# Circadian Desynchrony Promotes Metabolic Disruption in a Mouse Model of Shiftwork

**DOI:** 10.1371/journal.pone.0037150

**Published:** 2012-05-21

**Authors:** Johanna L. Barclay, Jana Husse, Brid Bode, Nadine Naujokat, Judit Meyer-Kovac, Sebastian M. Schmid, Hendrik Lehnert, Henrik Oster

**Affiliations:** 1 Max Planck Institute of Biophysical Chemistry, Göttingen, Germany; 2 Department of Internal Medicine I, University of Lübeck, Lübeck, Germany; Simon Fraser University, Canada

## Abstract

Shiftwork is associated with adverse metabolic pathophysiology, and the rising incidence of shiftwork in modern societies is thought to contribute to the worldwide increase in obesity and metabolic syndrome. The underlying mechanisms are largely unknown, but may involve direct physiological effects of nocturnal light exposure, or indirect consequences of perturbed endogenous circadian clocks. This study employs a two-week paradigm in mice to model the early molecular and physiological effects of shiftwork. Two weeks of timed sleep restriction has moderate effects on diurnal activity patterns, feeding behavior, and clock gene regulation in the circadian pacemaker of the suprachiasmatic nucleus. In contrast, microarray analyses reveal global disruption of diurnal liver transcriptome rhythms, enriched for pathways involved in glucose and lipid metabolism and correlating with first indications of altered metabolism. Although altered food timing itself is not sufficient to provoke these effects, stabilizing peripheral clocks by timed food access can restore molecular rhythms and metabolic function under sleep restriction conditions. This study suggests that peripheral circadian desynchrony marks an early event in the metabolic disruption associated with chronic shiftwork. Thus, strengthening the peripheral circadian system by minimizing food intake during night shifts may counteract the adverse physiological consequences frequently observed in human shift workers.

## Introduction

*Shiftwork* refers to a job schedule in which employees work hours other than the standard hours, e.g. in the early morning or during the night. Chronic shiftwork is correlated to increased body mass index (BMI) and risk of developing metabolic syndrome [1]–[5]. It further affects the secretion of endocrine factors such as melatonin, growth hormone, prolactin, leptin and glucocorticoids, all of which impinge on metabolic homeostasis [6]–[10]. It has been proposed that chronic sleep disruption may be causal in the incidence of metabolic dysregulation and obesity in shift workers [3], [7], [11]. In rodents, four weeks of enforced daytime activity causes altered diurnal rhythms of food uptake, blood glucose and neuronal activation [12], [13], suggesting an effect of sleep timing on circadian clock function. However, the molecular mechanisms underlying these observations remain unclear.

Circadian clocks provide a mechanism by which external time is interpreted and translated into time-of-day appropriate physiology, with the overall aim of promoting fitness and energy efficiency of an organism [14]. In mammals, circadian physiological rhythms are regulated by a hierarchical system of tissue clocks, with a master pacemaker situated in the suprachiasmatic nuclei (SCN) of the hypothalamus. The SCN receive external time information predominately in the form of light via the retinohypothalamic tract. They then transmit this information via neurohumoral signals to subsidiary oscillators in other regions of the brain and in the periphery. Peripheral clocks can also be directly entrained by food, independent of the SCN [15].

At the cellular level circadian clocks are organized into a system of interlocking transcriptional translational feedback loops (TTLs) [16]. The positive arm of the core TTL in mammals is formed by the transcriptional activators circadian locomotor output cycles kaput (CLOCK) and brain and muscle ARNT-like protein 1 (BMAL1 or ARNTL), which dimerize and bind to E-Box *cis*-elements on target gene promoters. Transcription of three period (*Per1-3*) and two cryptochrome genes (*Cry1* and *2*) is initiated by the CLOCK:BMAL1 complex. The resulting CRY and PER proteins inhibit CLOCK:BMAL1 activity, thus forming a negative feedback loop impinging on their own transcription. The clock machinery translates time information into physiologically meaningful signals by the regulation of hundreds of clock-controlled output genes (CCGs). While the nature of core clock genes is preserved among different tissues, CCGs are highly tissue-specific. It is estimated that up to 10% of the transcriptome of each cell is regulated in a circadian fashion [17]. In this way, physiological processes can be sequestered to the appropriate time of the day.

The rhythmic mammalian transcriptome of various tissues is enriched for genes involved in metabolic processes, indicating a major role for the clock in metabolic regulation [17]. In humans, circadian disruption correlates with metabolic dysfunction [5], [14], [16]. Genetic clock disruption in rodents has similar effects. *Bmal1* ^−/−^ mice show impaired glucose tolerance, gluconeogenesis, altered triglyceride rhythms and increased body fat [18], [19]. *Clock* ^Δ19^ mice display impaired gluconeogenesis in addition to hyperphagy and obesity [20], [21], and *Per2* mutant mice are also obese [22].

Given that sleep and clock dysfunction share the same metabolic endpoint, it was hypothesized that shiftwork-induced metabolic deregulation may be a direct result of circadian clock disturbance. This study demonstrates that two weeks of timed sleep restriction (TSR) leads to clock disruption and global diurnal desynchrony at the transcriptional level in the liver, while metabolic functions are still only moderately affected. Molecular and metabolic effects of TSR can be alleviated by timed food access, a strong synchronizer of peripheral clocks, emphasizing the therapeutic potential of behavioral chronotherapy to combat the metabolic consequences of shiftwork.

## Results

### TSR in Mice Results in Perturbations of Diurnal Behavioral Rhythms

**Figure 1:**
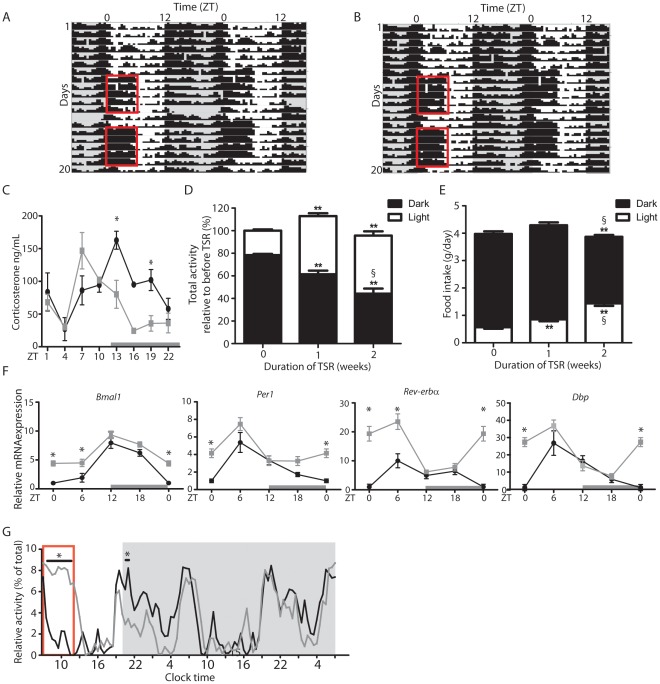
TSR results in perturbations of diurnal behavioral rhythms. (A and B) Representative activity recordings of TSR mice. Actograms are double-plotted. Grey shadings indicate dark phases, red boxes indicate period of TSR. (C) Plasma corticosterone in control and TSR mice on Day12 of TSR (n = 3−5). (D) Distribution of light and dark activity during TSR expressed as % of total activity of the same set of animals during control conditions (n = 5 cages of 4 mice each), * indicates p<0.05 and **p<0.01 relative to 0 weeks of TSR, § indicates p<0.05 relative to 1 week of TSR. (E) Food intake during light and dark phase during TSR, * indicates p<0.05 and **p<0.01 relative to 0 weeks of TSR (n = 10−23), § indicates p<0.05 relative to 1 week of TSR determined by 2-way ANOVA. (F) Quantitation of clock gene mRNA by ISH in the SCN of control and TSR mice expressed relative to controls at ZT0 (n = 3 at each time point). (G) Activity of control and TSR mice upon release into DD (n = 3 cages). Grey bars indicate dark phase, red box indicates last period of gentle handling. Control mice represented by black lines, TSR mice by grey lines; * indicates p<0.05 determined by 2-way ANOVA.

Young adult male C57Bl6/J mice were subjected to a two-week regime of TSR designed to mimic common human night shiftwork schedules. Mice were housed under 12 h light: 12 h dark (LD) conditions with food and water given *ad libitum* at all times. During TSR animals were kept awake during the first 6 hours of the light phase (*Zeitgeber* time (ZT) 0–6) on Days 1 through 5 and Days 8 through 12 using a ‘gentle handling’ approach designed to minimize stress effects and intervention by the experimenter [23], [24] (red boxes in Figure 1A and 1B). At all other times mice were left undisturbed. To assess stress levels in TSR mice plasma corticosterone was measured at 8 different times during the day. No overall increase in diurnal corticosterone release was observed, but corticosterone peak phase was found to be advanced by 6 h in TSR mice (Figure 1C). As expected, during TSR mice displayed increased activity from ZT0 to ZT6 relative to control animals (Figure 1A and 1B). In addition, activity during the dark phase gradually declined during the course of TSR (Figure 1D). In parallel, TSR animals developed altered feeding rhythms, with light phase food intake increasing relative to controls (Figure 1E). Of note, no overall changes in activity or food intake, and no significant effects on body weight were recorded during 2 weeks of TSR (Figure 1D, 1E and S1). On Day 12 central clock function was assessed by quantifying clock gene expression profiles in the SCN using ^35^S-UTP labeled ISH on sections. An upregulation of mRNA levels was observed in TSR animals in the early light phase (ZT1) for *Bmal1*, *Per1* and *Dbp*, and mid light phase (ZT7) for *Rev-erb*α (Figure 1F). However, overall diurnal rhythmicity and peak phasing of clock gene transcripts in the SCN was maintained. In line with this, when TSR mice were released into constant darkness (DD), thus avoiding photic masking effects on activity rhythms [25], activity profiles were virtually indistinguishable from control mice that had been allowed to sleep *ad libitum* prior to release into DD (Figure 1G).

### TSR Resets Liver Clocks

**Figure 2:**
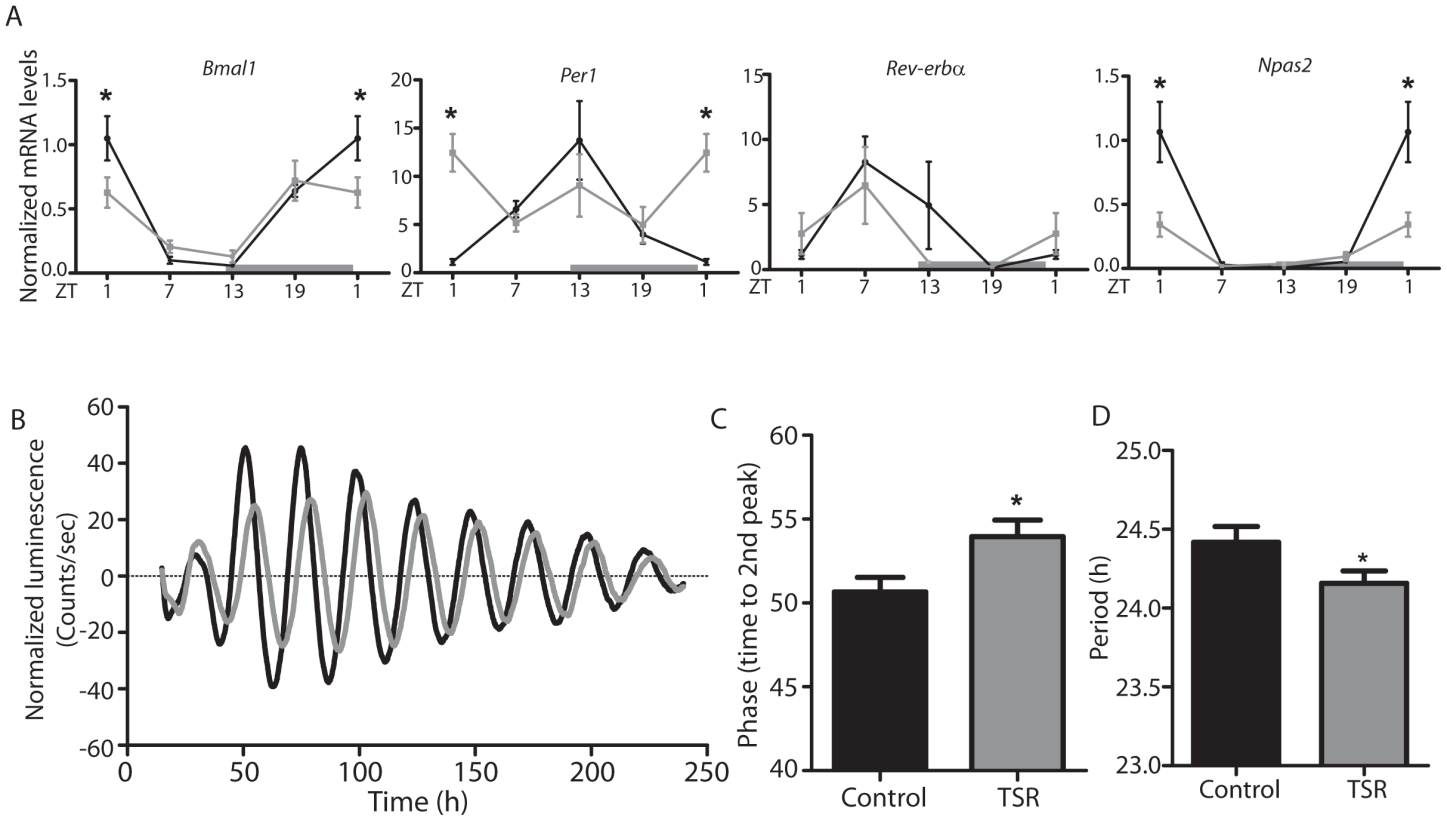
TSR resets the hepatic circadian clock. (A) qPCR analysis of diurnal clock gene mRNA profiles in the liver of control and TSR mice (n = 3 at each time point). (B) Luciferase activity in liver slice cultures from control and TSR *PER2::LUC* mice (n = 8) and (C) quantitation of phase and (D) period. Control mice are represented by black lines, TSR mice by grey lines; * indicates p<0.05 determined by 2-way ANOVA.

The marked effect of TSR on food intake and corticosterone rhythms prompted us to assess circadian clock function in the liver, a key peripheral tissue involved in energy metabolism. Diurnal clock gene expression profiles in the liver were analyzed using qPCR. A marked dampening of diurnal transcriptional rhythms was observed for *Bmal1*, *Per1* and *Npas2* with changes in expression peak times for *Bmal1* and *Per1*, while little effect was seen for *Rev-erbα* (Figure 2A). To examine the significance of these changes for hepatic clock function in the absence of external entrainment signals, liver slice cultures from TSR and control *PER2::LUC* circadian clock reporter mice were compared for phase and period. Slices from TSR animals showed delayed phasing and decreased period lengths of luciferase activity rhythms relative to control animals (Figure 2B–D).

### TSR Disrupts Transcriptome Rhythmicity in the Liver

**Figure 3:**
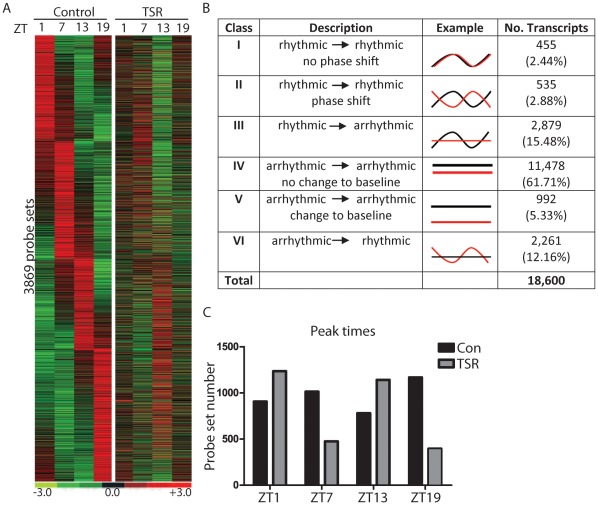
TSR causes global disruption of transcriptome rhythmicity in the liver. (A) Heat map illustrating diurnal expression of rhythmically expressed genes sorted for phase in control animals, and their corresponding expression profiles in TSR animals in liver (n = 3 at each time point). (B) Summary of transcriptional effects seen in liver following TSR. (C) Peak times of rhythmically expressed genes under control and TSR conditions. Black bars and lines indicate controls, grey bars and red lines indicate TSR.

Because of the strong effect of TSR on liver clock regulation we speculated that TSR would have a broad impact on transcriptome regulation in this tissue. To test this, cRNA preparations from liver samples harvested at 4 different time points from TSR and control mice were hybridized to whole genome expression microarrays (Affymetrix Mouse Gene 1.0 ST Arrays). Under control conditions, and in accordance with previous reports, we found a large number of genes with diurnal changes in expression levels (Figure 3A). After TSR the vast majority of previously rhythmic genes no longer showed diurnal variation and the normal phase relationship between different transcripts was lost (Figure 3A). When rhythmic genes from both treatment groups were sorted for expression peak time, profound changes in the overall distribution of transcriptional activity were observed. While expression peaks were evenly distributed along the day in the liver of control animals, a bimodal distribution pattern was observed in TSR animals, with most genes peaking in the early light phase or the early dark phase (ZT1 and ZT13, respectively; Figure 3B). Interestingly, although a general desynchrony of a formerly rhythmic transcriptome organization was observed during TSR, at the same time an additional array of changes in expression profiles for specific transcripts including phase shifts, alterations to baseline expression of non-rhythmic genes, and the induction of rhythmic gene expression in previously non-rhythmic genes was observed. TSR-induced changes in diurnal gene regulation are summarized and compared in Figure 3C. Full gene lists are provided in Table S1. In order to confirm that the array expression data was reliable, we directly compared our array results to the qPCR data on clock gene expression (Figure 2). A good correlation between both data sets was observed (Figure S2).

### Metabolic Gene Rhythmicity is Affected by TSR

**Figure 4:**
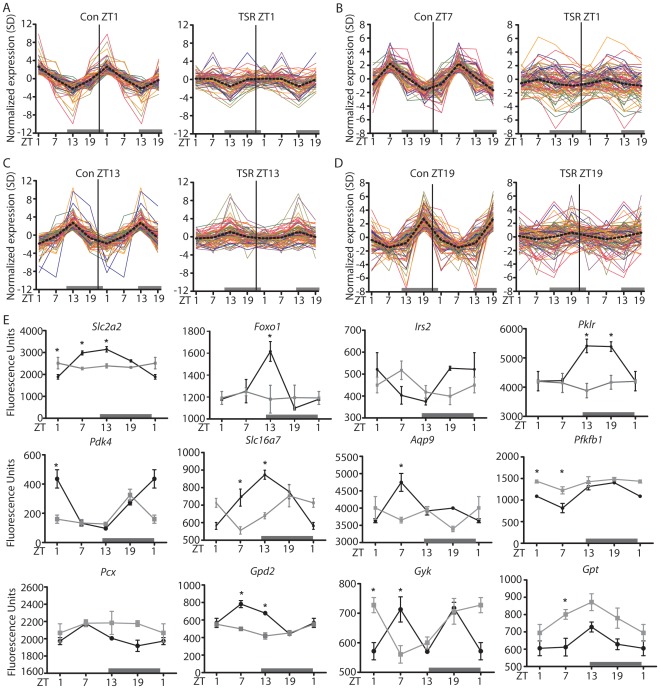
Metabolic gene rhythmicity in the liver is affected by shiftwork. Double plotted expression profiles of rhythmic genes associated with the GO terms ‘Carbohydrate Metabolism’, ‘Lipid Metabolism’ and ‘Amino Acid Metabolism’ sorted for peak time ((A) ZT1, (B) ZT7, (C) ZT13 and (D) ZT19) for both control and TSR groups. (E) Array expression profiles of selected metabolically relevant genes with altered expression profiles in the livers of TSR mice. Control mice are represented by black lines, TSR mice by grey lines; * indicates p<0.05 determined by 2-way ANOVA.

Previous studies in humans and rodents show that chronic shiftwork has profound effects on metabolism. Supporting this, gene ontology (GO) analysis of our array data revealed that the genes that lost rhythmicity following TSR were enriched for transcripts involved in metabolic processes (Table S2). To further investigate this phenomenon, expression profiles of all genes rhythmically expressed under control conditions and associated with metabolism (i.e. listed under the GO categories ‘Carbohydrate Metabolism’ - GO:0005975, ‘Lipid Metabolism’ - GO:0006629 or ‘Amino Acid Metabolism’ - GO:0006520) were compared. A profound loss of metabolic gene rhythmicity was seen for genes peaking at all time points (Figure 4A–D). Of note, no directional shift in expression peaks was observed for metabolic genes that were rhythmic both under control and TSR conditions (Figure S3), indicating that TSR had a disruptive, but not a phase resetting effect on liver metabolism.

**Figure 5:**
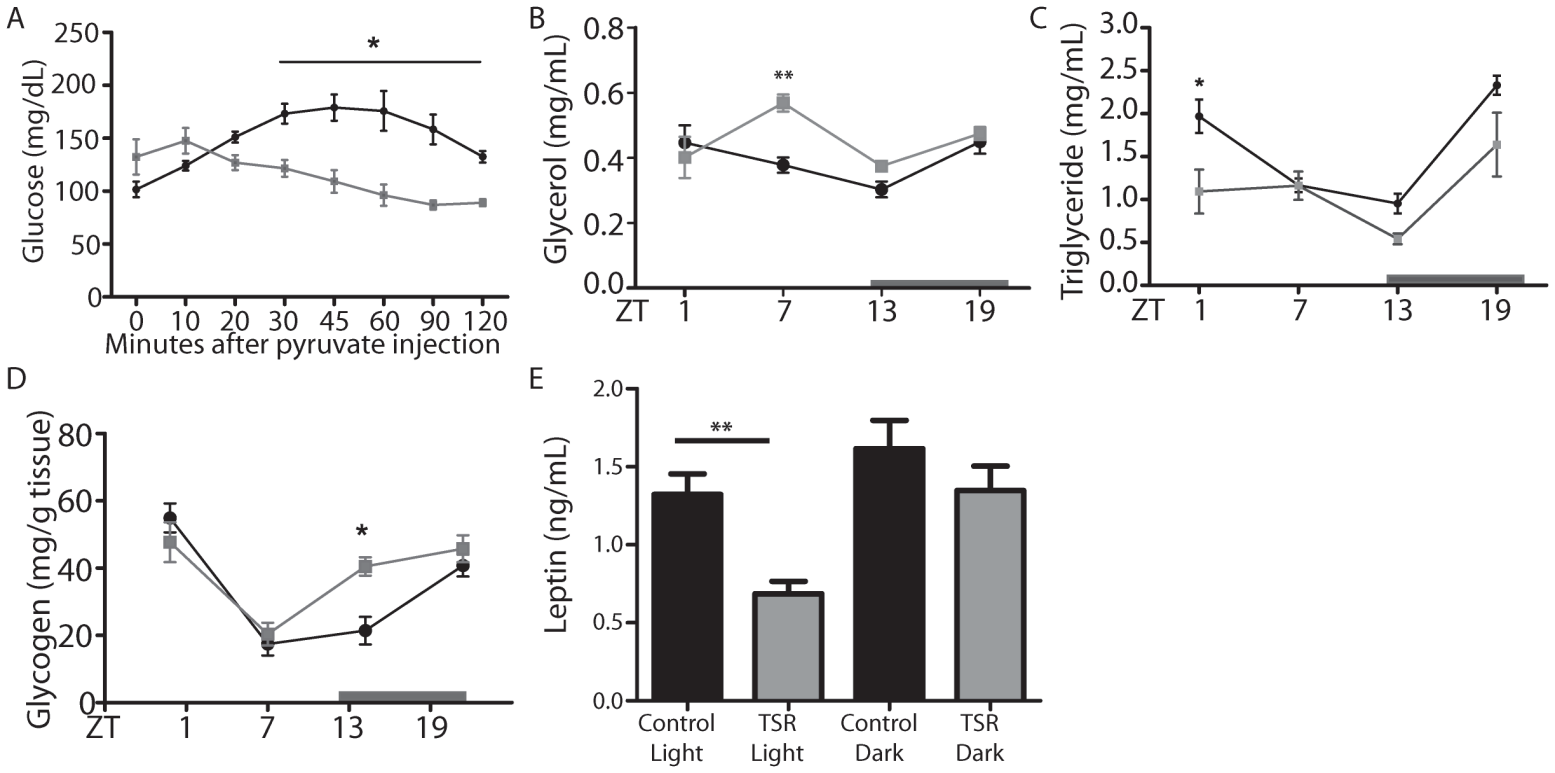
Metabolic effects of TSR. (A) Pyruvate tolerance test in control and TSR mice (n = 5). Circulating glycerol (B) and triglyceride (C) rhythms in control and TRS mice (n = 3 at each time point). (D) Liver glycogen levels in control and TSR mice (n = 3 at each time point) (E) Leptin levels during light and dark phase in control and TRS mice (n = 10). Control mice are represented by black lines, TSR mice by grey lines; * indicates p<0.05 and **p<0.01 determined by 2-way ANOVA.

Among the affected genes were a number of important regulators of carbohydrate metabolism (Figure 4E), such as the glucose transporter 2 *(Slc2a2*), glucose-responsive forkhead box O1 (*Foxo1)* and insulin receptor substrate 2 (*Irs2*). There appeared to be a particular emphasis on regulators of the glycolysis/gluconeogenesis pathway, such as liver pyruvate kinase (*Pklr*), pyruvate dehydrogenase kinase 4 (*Pdk4*), the pyruvate transporter *Slc16a7*, the glycerol transporter aquaporin 9 (*Aqp9*), fructose-2,6-biphosphatase 1 (*Pfkfb1*) and pyruvate carboxylase (*Pcx*). Glycerol phosphate dehydrogenase 2 (*Gpd2*) and glycerol kinase (*Gyk*), regulators of glycerol biosynthesis, and glutamic-pyruvate transaminase (*Gpt*), a regulator of pyruvate production from alanine, were also affected. Together, these findings suggested a marked effect of TSR on glucose metabolism, particularly gluconeogenesis. Supporting this, TSR animals showed profoundly impaired gluconeogenic capacity, determined by a pyruvate tolerance test (Figure 5A). Hepatic glycogen storage rhythms were also altered with increased levels at the beginning of the dark phase (ZT13) in TSR mice, and circulating glycerol was increased at ZT13, while diurnal triglyceride rhythms were dampened with lower levels at ZT1, indicating that TSR had further effects on lipid metabolism (Figure 5B–D). Finally, decreased leptin levels were observed in the light phase in TSR animals, correlating to increased food intake observed during this period (Figure 1E).

### Dark Phase Restricted Feeding Prevents TSR-induced Peripheral Clock and Metabolic Disruption

**Figure 6:**
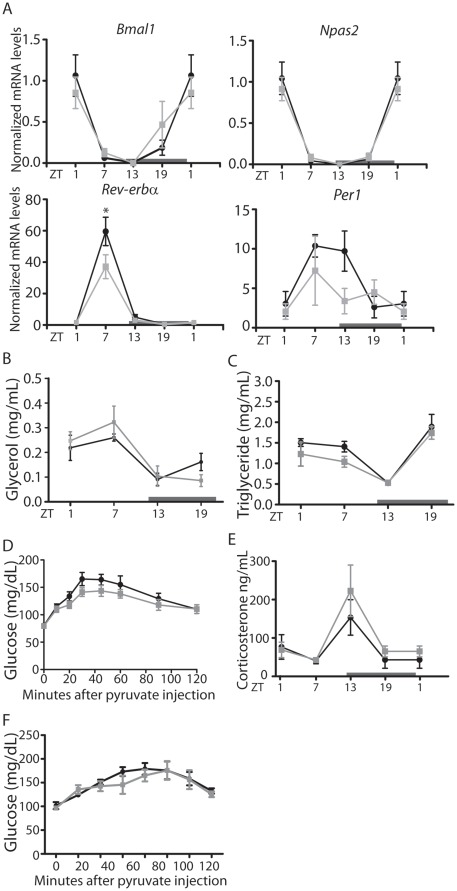
Dark phase feeding rescues TSR-induced disruption of peripheral clock gene expression and gluconeogenesis. (A) qPCR analysis of clock gene expression in the liver of control/dark phase fed (DF) and TSR/dark phase fed (DF-TSR) mice. (B) Plasma glycerol and (C) triglyceride rhythms in control/DF and DF-TSR mice. (D) Pyruvate tolerance test in control/DF and DF-TSR mice (n = 5). (E) Plasma corticosterone control/DF fed and DF-TSR mice. (F) Pyruvate tolerance test in non-TRS mice under a TSR-like feeding regimen (n = 5). (A-E) Control mice are represented by black lines, TSR mice by grey lines. (F) *Ad libitum* fed mice are represented by black lines, TSR-like fed mice are represented by grey lines; * indicates p<0.05 determined by 2-way ANOVA, n = 3 at each time point, unless stated otherwise.

Although 2 weeks of TSR did not result in significant changes in overall food intake, insulin sensitivity or weight gain as has been reported for human shift workers or for longer shiftwork-like protocols in rodents, the perturbations to glucose and lipid handling observed in this study could be considered as early signs of future metabolic dysfunction. Given the hepatic transcriptional desynchrony observed in TSR animals we speculated that stabilizing liver clock regulation during TSR would potentially prevent, or at least alleviate, the emergence of metabolic effects. Indeed, when food access was restricted to the dark phase during TSR (NF-TSR) marked effects on both the molecular and metabolic level were observed. In NF-TSR animal diurnal expression peak times for *Bmal1, Npas2* and *Rev-erb*α in the liver were undistinguishable from control animals, however, some dampening effect of TSR on *Rev-erbα* and *Per1* expression was preserved (Figure 6A, compare Figure 2A). Moreover, TSR perturbations to plasma glycerol and triglyceride profiles were rescued in NF-TSR mice (Figure 6B and 6C, compare Figure 5B and 5C) together with improved gluconeogenic potential (Figure 6D). Finally, the phase of plasma corticosterone rhythms was restored in NF-TSR mice (Figure 6E, compare Figure 1C). In summary, restricting food intake during TSR to the normal active phase rescued peripheral clock gene rhythmicity and subsequently improved glucose and lipid handling. This marked effect of feeding time on TSR effects prompted us to test if altered food intake rhythms may play a causative role in the metabolic pathophysiology of shiftwork [26]. To test this, mice were subjected to two weeks of timed feeding schedules mimicking the light/dark food intake pattern observed in the second week of TSR (Figure 1E), followed by a pyruvate tolerance test on the last day. These animals showed no changes to gluconeogenic potential (Figure 6F), indicating that alterations in food uptake rhythms are necessary, but not sufficient for the induction of metabolic effects of TSR.

## Discussion

The current study employs a 2 week schedule of TSR in mice to examine the early molecular and physiological responses to altered activity timing preceding the metabolic consequences of chronic shiftwork. Profound disruption in the diurnal regulation of core clock genes and CCGs was observed in liver, with a strong emphasis on metabolic transcripts, while only moderate effects were seen at the level of the circadian pacemaker of the SCN. The physiological significance of these changes was demonstrated by early alterations in lipid and glucose handling, while bodyweight regulation was not yet affected. Both peripheral clock gene rhythmicity and metabolic physiology were rescued by concurrent dark phase restricted food access, suggesting meal timing as a possible therapeutic intervention for the treatment of shiftwork-associated metabolic disorders.

### TSR Effects on SCN Clock Rhythms

Changes to core clock gene expression were evident following TSR. In the SCN, moderate effects on *Bmal1*, *Per1*, *Rev-erb*α and *Dbp* were observed, while overall rhythmicity was preserved. At the behavioral level, however, decreased activity was observed in the dark phase, accompanied by a shift of food intake to the light phase. These activity phase shifts appear to be acute effects of the TSR procedure, perhaps in response to increased light input, and alterations in activity rhythms ceased when animals were released into DD conditions after the end of TSR (Figure 1). Collectively this suggests that TSR has only a moderate effect on the central clock, despite an overall disruption of diurnal activity, although it is not possible to conclusively rule out changes to CCGs in the SCN. These findings correlate with a recent study of 4 weeks of enforced daytime activity in rats which showed no changes of PER1 protein expression in the SCN, but marked disruption of PER1 rhythms in other hypothalamic nuclei [13]. It would therefore be interesting to extend our analysis to CCGs in the SCN, and other clocks in the brain, or to analyze clock gene effects after longer periods of TSR. Given the late onset of metabolic disturbances in human shift workers it is tempting to speculate that SCN clock disruption may become more apparent at a later time point. In a combined review of six studies of permanent night shift workers describing melatonin rhythms – generally considered a reliable readout of the circadian pacemaker in humans – it was concluded that very few people ever show an adaptation of their internal clock to the altered activity schedule [27].

### TSR Causes Global Disruption of the Liver Clock and CCGs

The effect of TSR on clock gene expression in the liver was much more pronounced than in the SCN (Figure 2), with a consequent global disruption of normal transcriptome rhythmicity (Figure 3). Genes from Class III (“rhythmic to arrhythmic”) showed ontology enrichment for metabolic processes, correlating with a marked dampening of rhythms in metabolism-associated (Figure 4). TSR also impinges on the regulation of several non-rhythmic genes in which rhythmic expression was induced following TSR (Class VI). These genes may be directly affected by changes in food uptake [28] or activity [29]. If one assumes that rest and activity roughly corresponds to fasting and feeding, the strong increase in early light phase activity during TSR would indicate a profound increase in food intake during this time which would then drive gene expression rhythms. In this particular case this assumption would be misleading, as the increased activity can be largely attributed to exploration of the novel objects placed in the cage as part of the gentle handling protocol. This is supported by the observation that the effects of TSR on feeding rhythmicity are far less pronounced than those on locomotor activity (Figure 1D and 1E). Alternately these genes may be directly sleep-responsive and may therefore be classified as “acutely sleep (-loss) regulated”. In a study by Maret *et al.* which employed the same gentle handling method used in the current study, a number of genes specifically responsive to sleep-loss were identified for the brain as well as the liver [30], some of which encoded heat shock proteins which the authors hypothesized to be part of a tissue-independent stress response. A number of heat shock protein gene were also identified in the current study (Table S1). However, it should be noted that sleep was not directly measured in either the Maret study or ours - with the notable exception of during the TSR protocol when the animals were continually observed for wakefulness - and therefore any possible “sleep-related” changes are inferred from activity and must be very cautiously considered.

### TSR Effects on Carbohydrate Metabolism in Liver

Genes involved in carbohydrate metabolism were deregulated in the liver. Under normal conditions hepatic glucose uptake and glycolysis are enhanced during the active phase, corresponding with high *Slc2a2* and *Pklr* expression at ZT13/19 in our control animals. There was an emphasis on gluconeogenesis in the late part of the rest period at ZT7 and ZT13, indicated by high *Slc16a7* and *Aqp9* (substrate uptake), high *Pcx* (conversion of pyruvate to oxaloacetate), high *Gpd2* and *Gyk* (glycerol *de novo* biosynthesis), and low *PDK4* mRNA levels (inhibition of glycolysis). TSR resulted in most of these genes being reduced, in some cases remaining at basal expression levels throughout the day, indicating profound changes to diurnal carbohydrate utilization (Figure 4). Downregulation of hepatic *Scl2a2* has been previously reported in mice with genetic liver-specific clock disruption (*L-Bmal1^−/−^*) [18], which supports our hypothesis that alterations in liver clock regulation may underlie the molecular perturbations brought about by TSR. Molecular disruption could also be correlated with physiology, with TSR mice displaying severely retarded gluconeogenic potential as measured using a pyruvate tolerance test (PTT). This phenotype was observed previously in *Clock* ^Δ19^ and *Bmal1* ^−/−^ mice, suggesting a direct effect of the circadian clock on this process [21]. Liver-specific *L-Foxo1* ^−/−^ mice similarly display reduced hepatic glucose production [31], [32].

Circulating glycerol, a product of lipolysis and a substrate for gluconeogenesis, was high directly after the sleep restriction period (ZT7) following TSR (Figure 1), suggesting increased energy demands at this time, further emphasized by the impaired utilization of glycerol by the liver. Hepatic glycogen storage was increased at the beginning of the nocturnal active period (ZT13) following TSR. In the absence of gluconeogenic potential, despite adequate substrate availability, more emphasis is placed on glycogen. Once glycogen stores are depleted under resting, i.e. fasting, conditions (light phase), in the absence of efficient gluconeogenesis, increased food intake would be necessary to meet glucose demands. In TSR mice leptin is decreased at this time, relieving normal appetite suppression and coinciding with increased food consumption (Figure 1). Under sustained shiftwork conditions this would presumably promote hyperphagy [12]. In human shift workers – who, unlike rodents, have access to a range of food options – abnormal eating behavior with an increased tendency to consume high caloric carbohydrate-rich foods is reported [33]–[37]. In summary, the observed metabolic effects in TSR mice suggest a desynchronization of hepatic energy utilization, which under control conditions should employ glycogen breakdown at the beginning of the resting phase and move to gluconeogenesis when glycogen stores are depleted towards the end of the resting phase. In TSR glucose utilization from liver stores is altered and may promote carbohydrate craving and increased food intake during normal rest hours.

### Peripheral Clock Disruption Precedes Metabolic Disorders

Collectively, the observed molecular disruption seen in the liver can be directly correlated to changes in physiology. However, given the short duration of the model used, the full-blown metabolic phenotype characterized by obesity, insulin and leptin resistance seen in more chronic models of sleep restriction and in human shiftwork were not observed. Similarly for rodents, in which 4 weeks of night work results in hyperphagy and obesity, after only 2 weeks the effects on food intake are still relatively mild [12]. Therefore it may be concluded that TSR-induced clock disruption precedes metabolic pathophysiology. This is reminiscent of findings in leptin-deficient *ob/ob* mice, showing that circadian disruption precedes the development of obesity in these animals [38]. Additionally, clock disruption in humans can confound the effects of shiftwork. In a recent study by Gamble et al., it was shown that clock gene polymorphisms in nurses were correlated with adverse adaptation to shiftwork, culminating in increased alcohol and caffeine consumption and the likelihood to doze [39]. This scenario could be extended to rotating shiftwork conditions, which would presumably lead to chronic circadian desynchrony, and result in the most severe pathophysiology [40], [41]. These findings strongly support a causal role of clock desynchrony on metabolism following sleep disruption.

### Peripheral Clock Restoration Prevents Metabolic TSR Effects

In order to strengthen the link between the circadian clock and shiftwork-induced metabolic effects, a regime of normocaloric dark phase restricted feeding was employed concurrent to TSR to stabilize the peripheral clock machinery. Dark phase restricted feeding has been shown previously to reset clock gene rhythms in subsidiary oscillators, but not the SCN master clock [15], [28]. Further, Dark phase feeding stabilizes body temperature and glucose rhythms in a model of night work in rats [12]. Indeed, Dark phase feeding ameliorated the effects of TSR on clock gene rhythms in the liver (Figure 6). Concurrently, disrupted triglyceride, glycerol and corticosterone rhythms were rescued, as was gluconeogenic potential. Together, these findings suggest that TSR-induced metabolic resetting is a result of clock perturbation, and altered feeding patterns might, at least in part, underlie this phenomenon. Adverse effects of dark phase eating have been reported in studies of shift workers, resulting in decreased glucose and lipid tolerance [42]–[44]. However, merely enforcing an altered food consumption schedule to mimic that seen during TSR did not result in compromised gluconeogenic potential (Figure 6). Collectively these data suggest that alterations in feeding rhythmicity observed during TSR may promote, but are not sufficient to explain the metabolic pathophysiology of shiftwork. Nevertheless, imposing a strict dark phase feeding rhythm can be employed to reset peripheral clocks and alleviate metabolic perturbations.

### Conclusion

This study examines the early effects of extended timed sleep restriction as an experimental model of shiftwork on behavior, physiology and transcriptional regulation, with an emphasis on the circadian aspects of metabolism. While it is clear that causal interpretations from studies on complex interacting processes such as diurnal activities, feeding, sleep, metabolism, and circadian mechanisms are always limited, our liver transcriptome analyses indicate a direct effect of sleep timing on the molecular circadian machinery and on clock controlled genes involved in carbohydrate and lipid utilization. Importantly, disruption to circadian gene rhythmicity appears early compared to the adverse metabolic disturbances seen with chronic shiftwork in both rodents and humans, suggesting a causal role of clock disruption in the progression of shiftwork-induced metabolic syndrome. Resetting peripheral circadian clocks by nighttime feeding rescues physiological parameters altered during timed sleep restriction, implying that peripheral clocks may be attractive therapeutic targets for combating the metabolic effects of shiftwork.

## Materials and Methods

### Animals

Male C67Bl6/J mice, 10–14 weeks old, were entrained to a 12 h (50 lux) light: 12 h dark (LD) schedule with lights on (ZT0) at 7:00am. Activity measurements were recorded using custom-made infra-red detectors fitted to the roof of the cages and analyzed using ClockLab software (Actimetrics, Evanston, IL). Mice were housed in groups of 3–6 animals per cage, and kept under constant temperature (20.0+/−0.5°C) and humidity (50–60%) conditions with *ad libitum* access to standard chow (Ssniff V1126, Soest, Germany) and water. All animal experiments were approved by the Office for Consumer Protection and Food Safety (LAVES) of the State of Lower Saxony and executed in accordance with the German Law on Animal Welfare.

### Timed Sleep Restriction (TSR) and Feeding Schedules

During TSR mice were kept awake using a gentle handling method between ZT0 and ZT6 from Days 1 to 5, and Days 8 to 12 [23]. Briefly, animals were constantly watched and novel objects were introduced into the cages to induce alertness only when mice assumed a position suggestive of intended sleep. At all other times mice were able to sleep *ad libitum*. Control mice were kept under the same conditions in separate compartments, without intervention. Starting in the evening of Day 11, control and TSR mice were sacrificed by cervical dislocation at 6 hour intervals. In the dark phase, a 5 W safety red light was used to avoid light effects. Plasma, liver, epididymal adipose (WAT) and brains were collected and frozen. In the case of TSR with high light intensity, 500 lux light was used in the same LD schedule. In the case of TSR with dark phase restricted feeding (DF-TSR), food was removed from the cages at ZT0 (7:00am) on each day and replaced at ZT12 (7:00pm) on each day. In the case of altered food rhythmicity in control mice, mice were provided with 1.6 g of food at the start of the light/inactive phase and 2.2 g of food at the start of the dark/active phase. Each time food was given, any remaining uneaten food was removed to prevent hoarding.

### Pyruvate Tolerance Testing

Pyruvate tolerance tests were performed in the week prior to TSR, and on Day 12 of TSR. Mice were starved for 13 h overnight, and injected with 2 g/kg pyruvate i.p. at ZT1. Glucose measurements were taken from tail vein blood at indicated time points.

### Plasma Analysis

Leptin was determined using the Mouse Leptin ELISA Kit (Crystal Chem, Downers Grove, IL), and insulin was determined using the Ultrasensitive Mouse Insulin ELISA (Mercodia, Uppsala, Sweden). Circulating glycerol and triglycerides were determined using the Serum Triglyceride Determination Kit (Sigma, St. Louis, MO), and glycogen was determined using anthrone reagent as described previously [45]. Corticosterone was measured from plasma samples using the ImmuChem Double Antibody 125I-Radioimmunoassay Kit (MP Biomedicals, Solon, OH).

### In situ Hybridization

*In situ* hybridization using digoxigenin or ^35^S-labeled probes on frozen sections was performed as described [46]. Probe details are available on request.

### Quantitative Real-time PCR (qPCR)

Relative quantification of mRNA levels by qPCR was done as previously described [47]. Total RNA was extracted using Trizol reagent (Invitrogen, Carlsbad, CA), and cleaned of genomic DNA contamination using TURBO DNase (Ambion, Austin, TX) as per manufacturer’s instructions. cDNA synthesis was performed using Superscript III (Invitrogen) and random hexamer primers, and qPCR was performed using iQ SYBR Green Supermix (Bio-Rad, Hercules, CA) on an Bio-Rad C1000 Thermal Cycler and a CFX96 Real-Time system (95°C for 3 min, 40 cycles at 94°C for 15 sec, 60°C for 15 sec and 72°C for 20 sec, 95°C 10 sec, then 95°C for 10 sec followed by a melt curve from 65°C to 95°C at 0.5°C increments for 5 sec) Primer sequences are available on request. *Eef1a* was used as a reference gene, and data was expressed as relative quantitation (^ΔΔ^-CT method).

### Liver Slice Culture and Luciferase Measurements

Control and TSR heterozygous *PER2::LUC* (n = 6) [48] animals were scarified at ZT6 and 300 µm vibratome slices were prepared from agarose embedded liver lobes and cultured on Millicell culture membranes (PICMORG50, Millipore, Billerica, MA) in DMEM medium (high glucose, w/o L-glutamine), supplemented with 10 mM Hepes (pH 7.2), 2% B27, 25 units/ml penicillin, 25 µg/ml streptomycin, 352.5 mg/ml sodium carbonate, 2 mM L-glutamine and 0.1 mM luciferin (all from Invitrogen) in a Lumicycle luminometer (Actimetrics). For each animal 3 liver sections were analyzed.

### Microarray Analysis

Microarray experiments using liver and adipose tissue total RNA preparations were performed at the Transcriptome Analysis Lab (TAL) of the University of Göttingen, Germany. cRNA samples were hybridized to MoGene ST1.0 gene expression arrays (Affimetrix, Santa Clara, CA). Three chips per time point, condition and tissue were used. Gene expression was normalized using d-Chip Software [49] and rhythmic gene expression and expression phases were determined using CircWave Software (University of Groningen, The Netherlands) as described [50]. Rhythmic genes were grouped and heat maps were generated using d-Chip Software [49]. These sequence data have been submitted to the GEO databases under accession number GSE33381. Gene ontology analysis was performed using DAVID (NIAID, NIH) [51], [52].

### Statistical Analysis

Unless otherwise noted in the figure legend, all data are shown as mean +/− SEM. Statistical comparisons were performed using GraphPad Prism software (GraphPad, La Jolla, CA) for single gene expression profiles and physiological parameters or using the Bioconductor/R software package [53] for comparing multiple data sets from the microarray experiments. T-tests, one-way or two-way ANOVA with Bonferroni post-hoc test were used to compare single transcript profiles or physiological parameters. For large scale multiple comparisons two-tailed t-tests with Benjamini-Hochberg corrections were used [54]. P-values below 0.05 were considered significant.

## Supporting Information

Figure S1 **Body weight of mice during and following TSR (n  = 10).**
(TIF)

Figure S2 **Comparison of qPCR (data replotted from** **Figure 2** **) and array fluorescence measures (normalized to ZT1 in control animals) during control and TSR conditions in the liver.** Arrays are represented by the solid lines, qPCR data by the broken lines. * indicates p<0.05 determined by 2-way ANOVA (n  = 3 per time point and condition).
(TIF)

Figure S3 **Peak time phase relationships of metabolic genes that were rhythmic under both control and TSR conditions (Classes I and II) in the liver.** Peak times were assessed by sine wave fitting. Circles indicate number of genes per 20° wedge. The black arrow represents the normalization vector for all shifts/genes.
(TIF)

Table S1 **Full gene lists from** **Figure 3B** **, including Probe ID, Gene ID, and peak time in both control and TSR mice.**
(PDF)

Table S2 **Category 1 enriched GO processes (DAVID) of Class III TSR-regulated genes (** **Figure 3E** **) in the liver.**
(PDF)

## References

[1] Baron KG, Reid KJ, Kern AS, Zee PC (2011). Role of Sleep Timing in Caloric Intake and BMI.. Obesity.

[2] Karlsson B, Knutsson A, Lindahl B (2001). Is there an association between shift work and having a metabolic syndrome? Results from a population based study of 27,485 people.. Occup Environ Med.

[3] Fonken LK, Workman JL, Walton JC, Weil ZM, Morris JS (2010). Light at night increases body mass by shifting the time of food intake.. Proceedings of the National Academy of Sciences.

[4] Li Y, Sato Y, Yamaguchi N (2011). Shift work and the risk of metabolic syndrome: a nested case-control study.. Int J Occup Environ Health.

[5] Garaulet M, Ortega FB, Ruiz JR, Rey-Lopez JP, Beghin L (2011). Short sleep duration is associated with increased obesity markers in European adolescents: effect of physical activity and dietary habits..

[6] Rehman J-u, Brismar K, Holmbäck U, Åkerstedt T, Axelsson J (2010). Sleeping during the day: effects on the 24-h patterns of IGF-binding protein 1, insulin, glucose, cortisol, and growth hormone.. European Journal of Endocrinology.

[7] Arendt J (2010). Shift work: coping with the biological clock.. Occupational Medicine.

[8] Wu H, Zhao Z, Stone WS, Huang L, Zhuang J (2008). Effects of sleep restriction periods on serum cortisol levels in healthy men.. Brain Research Bulletin.

[9] Crispim CA, Waterhouse J, Damaso AR, Zimberg IZ, Padilha HG (2011). Hormonal appetite control is altered by shift work: a preliminary study.. Metabolism.

[10] Van Cauter E, Knutson KL (2008). Sleep and the epidemic of obesity in children and adults.. Eur J Endocrinol.

[11] Korkmaz A, Topal T, Tan D-X, Reiter R (2009). Role of melatonin in metabolic regulation.. Reviews in Endocrine & Metabolic Disorders.

[12] Salgado-Delgado R, Angeles-Castellanos M, Saderi N, Buijs RM, Escobar C (2010). Food Intake during the Normal Activity Phase Prevents Obesity and Circadian Desynchrony in a Rat Model of Night Work.. Endocrinology.

[13] Salgado-Delgado R, Nadia S, Angeles-Castellanos M, Buijs RM, Escobar C (2010). In a Rat Model of Night Work, Activity during the Normal Resting Phase Produces Desynchrony in the Hypothalamus.. Journal of Biological Rhythms.

[14] Bass J, Takahashi JS (2010). Circadian Integration of Metabolism and Energetics.. Science.

[15] Damiola F, Le Minh N, Preitner N, Kornmann B, Fleury-Olela F (2000). Restricted feeding uncouples circadian oscillators in peripheral tissues from the central pacemaker in the suprachiasmatic nucleus.. Genes Dev.

[16] Takahashi JS, Hong H-K, Ko CH, McDearmon EL (2008). The genetics of mammalian circadian order and disorder: implications for physiology and disease.. Nat Rev Genet.

[17] Panda S, Antoch MP, Miller BH, Su AI, Schook AB (2002). Coordinated Transcription of Key Pathways in the Mouse by the Circadian Clock.. Cell.

[18] Lamia KA, Storch KF, Weitz CJ (2008). Physiological significance of a peripheral tissue circadian clock.. Proc Natl Acad Sci U S A.

[19] Rudic R, McNamara P, Curtis A, Boston R, Panda S (2004). BMAL1 and CLOCK, two essential components of the circadian clock, are involved in glucose homeostasis.. PLoS Biol.

[20] Turek FW, Joshu C, Kohsaka A, Lin E, Ivanova G (2005). Obesity and Metabolic Syndrome in Circadian Clock Mutant Mice.. Science.

[21] Rudic RD, McNamara P, Curtis A-M, Boston RC, Panda S (2004). BMAL1 and CLOCK, Two Essential Components of the Circadian Clock, Are Involved in Glucose Homeostasis.. PLoS Biol.

[22] Yang S, Liu A, Weidenhammer A, Cooksey RC, McClain D (2009). The Role of mPer2 Clock Gene in Glucocorticoid and Feeding Rhythms.. Endocrinology.

[23] Tobler I, Borbely A, Groos G (1983). The effect of sleep deprivation on sleep in rats with suprachiasmatic lesions.. Neurosci Lett.

[24] Hasan S, Dauvilliers Y, Mongrain V, Franken P, Tafti M (2012). Age-related changes in sleep in inbred mice are genotype dependent.. Neurobiol Aging 33: 195.

[25] Mrosovsky N (1999). Masking: History, Definitions, and Measurement.. Chronobiology International.

[26] Arble DM, Bass J, Laposky AD, Vitaterna MH, Turek FW (2009). Circadian timing of food intake contributes to weight gain.. Obesity (Silver Spring).

[27] Folkard S (2008). Do Permanent Night Workers Show Circadian Adjustment? A Review Based on the Endogenous Melatonin Rhythm.. Chronobiology International.

[28] Vollmers C, Gill S, DiTacchio L, Pulivarthy SR, Le HD (2009). Time of feeding and the intrinsic circadian clock drive rhythms in hepatic gene expression.. Proc Natl Acad Sci U S A.

[29] Yamanaka Y, Honma S, Honma K-I (2008). Scheduled exposures to a novel environment with a running-wheel differentially accelerate re-entrainment of mice peripheral clocks to new light–dark cycles.. Genes to Cells.

[30] Maret S, Dorsaz S, Gurcel L, Pradervand S, Petit B (2007). Homer1a is a core brain molecular correlate of sleep loss.. Proceedings of the National Academy of Sciences.

[31] Puigserver P, Rhee J, Donovan J, Walkey CJ, Yoon JC (2003). Insulin-regulated hepatic gluconeogenesis through FOXO1-PGC-1[alpha] interaction.. Nature.

[32] Haeusler RA, Kaestner KH, Accili D (2010). FoxOs Function Synergistically to Promote Glucose Production.. Journal of Biological Chemistry.

[33] Persson M, MÅRtensson J (2006). Situations influencing habits in diet and exercise among nurses working night shift.. Journal of Nursing Management.

[34] Wong H, Wong MCS, Wong SYS, Lee A (2010). The association between shift duty and abnormal eating behavior among nurses working in a major hospital: A cross-sectional study.. International Journal of Nursing Studies.

[35] Morikawa Y, Miura K, Sasaki S, Yoshita K, Yoneyama S (2008). Evaluation of the Effects of Shift Work on Nutrient Intake: A Cross-sectional Study.. Journal of Occupational Health.

[36] Di Lorenzo L, De Pergola G, Zocchetti C, L’Abbate N, Basso A (2003). Effect of shift work on body mass index: results of a study performed in 319 glucose-tolerant men working in a Southern Italian industry.. Int J Obes Relat Metab Disord.

[37] Spiegel K, Tasali E, Penev P, Van Cauter E (2004). Brief communication: Sleep curtailment in healthy young men is associated with decreased leptin levels, elevated ghrelin levels, and increased hunger and appetite.. Annals of Internal Medicine.

[38] Ando H, Kumazaki M, Motosugi Y, Ushijima K, Maekawa T (2011). Impairment of Peripheral Circadian Clocks Precedes Metabolic Abnormalities in ob/ob Mice.. Endocrinology.

[39] Gamble KL, Motsinger-Reif AA, Hida A, Borsetti HM, Servick SV (2011). Shift Work in Nurses: Contribution of Phenotypes and Genotypes to Adaptation.. PLoS ONE.

[40] Sookoian S, Gemma C, Fernández Gianotti T, Burgueño A, Alvarez A (2007). Effects of rotating shift work on biomarkers of metabolic syndrome and inflammation.. Journal of Internal Medicine.

[41] Gibbs M, Hampton S, Morgan L, Arendt J (2007). Predicting Circadian Response to Abrupt Phase Shift: 6-Sulphatoxymelatonin Rhythms in Rotating Shift Workers Offshore.. Journal of Biological Rhythms.

[42] Al-Naimi S, Hampton SM, Richard P, Tzung C, Morgan LM (2004). Postprandial Metabolic Profiles Following Meals and Snacks Eaten during Simulated Night and Day Shift Work.. Chronobiology International.

[43] Lund J, Arendt J, Hampton S, English J, Morgan L (2001). Postprandial hormone and metabolic responses amongst shift workers in Antarctica.. Journal of Endocrinology.

[44] Ribeiro D, Hampton S, Morgan L, Deacon S, Arendt J (1998). Altered postprandial hormone and metabolic responses in a simulated shift work environment.. Journal of Endocrinology.

[45] Roe JH, Dailey RE (1966). Determination of glycogen with the anthrone reagent.. Analytical Biochemistry.

[46] Oster H, Baeriswyl S, van der Horst GTJ, Albrecht U (2003). Loss of circadian rhythmicity in aging mPer1−/− mCry2−/− mutant mice.. Genes & Development.

[47] Oster H, Damerow S, Kiessling S, Jakubcakova V, Abraham D (2006). The circadian rhythm of glucocorticoids is regulated by a gating mechanism residing in the adrenal cortical clock.. Cell Metabolism.

[48] Yoo S, Yamazaki S, Lowrey P, Shimomura K, Ko C (2004). PERIOD2::LUCIFERASE real-time reporting of circadian dynamics reveals persistent circadian oscillations in mouse peripheral tissues.. Proc Natl Acad Sci USA.

[49] Li C, Wong WH (2001). Model-based analysis of oligonucleotide arrays: Expression index computation and outlier detection.. Proceedings of the National Academy of Sciences.

[50] Oster H, Damerow S, Hut RA, Eichele G (2006). Transcriptional Profiling in the Adrenal Gland Reveals Circadian Regulation of Hormone Biosynthesis Genes and Nucleosome Assembly Genes.. Journal of Biological Rhythms.

[51] Huang DW, Sherman BT, Lempicki RA (2009). Bioinformatics enrichment tools: paths toward the comprehensive functional analysis of large gene lists.. Nucleic Acids Research.

[52] Huang DW, Sherman BT, Lempicki RA (2008). Systematic and integrative analysis of large gene lists using DAVID bioinformatics resources.. Nat Protocols.

[53] Gentleman R, Carey V, Bates D, Bolstad B, Dettling M (2004). Bioconductor: open software development for computational biology and bioinformatics.. Genome Biology.

[54] Hochberg Y, Benjamini Y (1990). More powerful procedures for multiple significance testing.. Statistics in Medicine.

