# Supplementary figures and images for: Circadian Desynchrony Promotes Metabolic Disruption in a Mouse Model of Shiftwork
Source: PLoS One. 2012 May 21;7(5):e37150. doi: 10.1371/journal.pone.0037150 (PMC3357388; doi:10.1371/journal.pone.0037150)

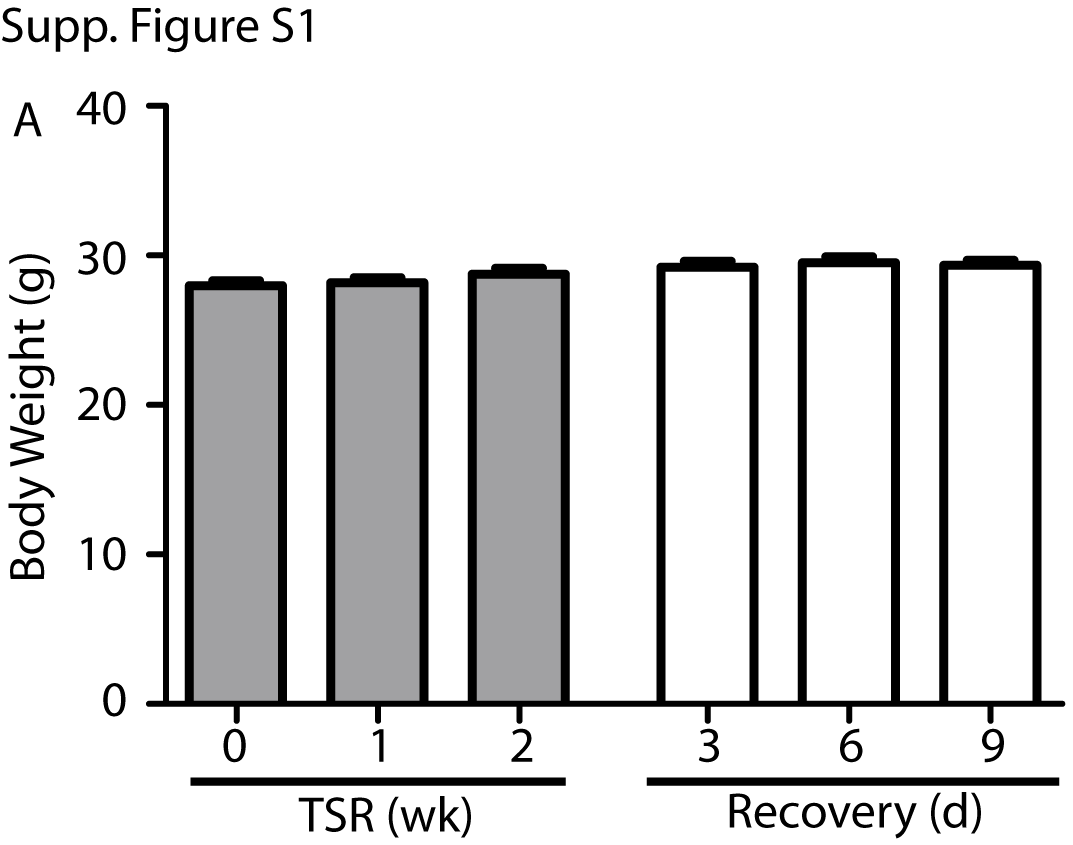

Supplement: Figure S1 — Body weight of mice during and following TSR (n = 10). (TIF) [file pone.0037150.s001.tif]

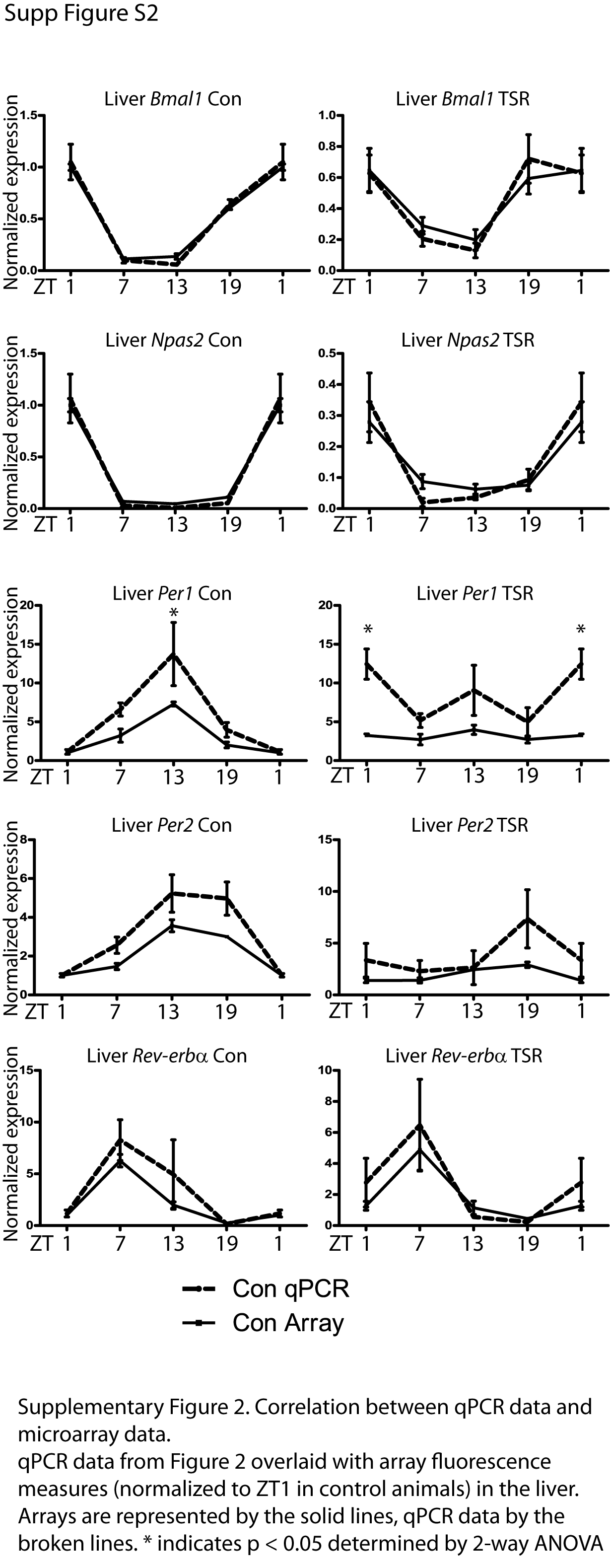

Supplement: Figure S2 — Comparison of qPCR (data replotted from Figure 2 ) and array fluorescence measures (normalized to ZT1 in control animals) during control and TSR conditions in the liver. Arrays are represented by the solid lines, qPCR data by the broken lines. * indicates p<0.05 determined by 2-way ANOVA (n = 3 per time point and condition). (TIF) [file pone.0037150.s002.tif]

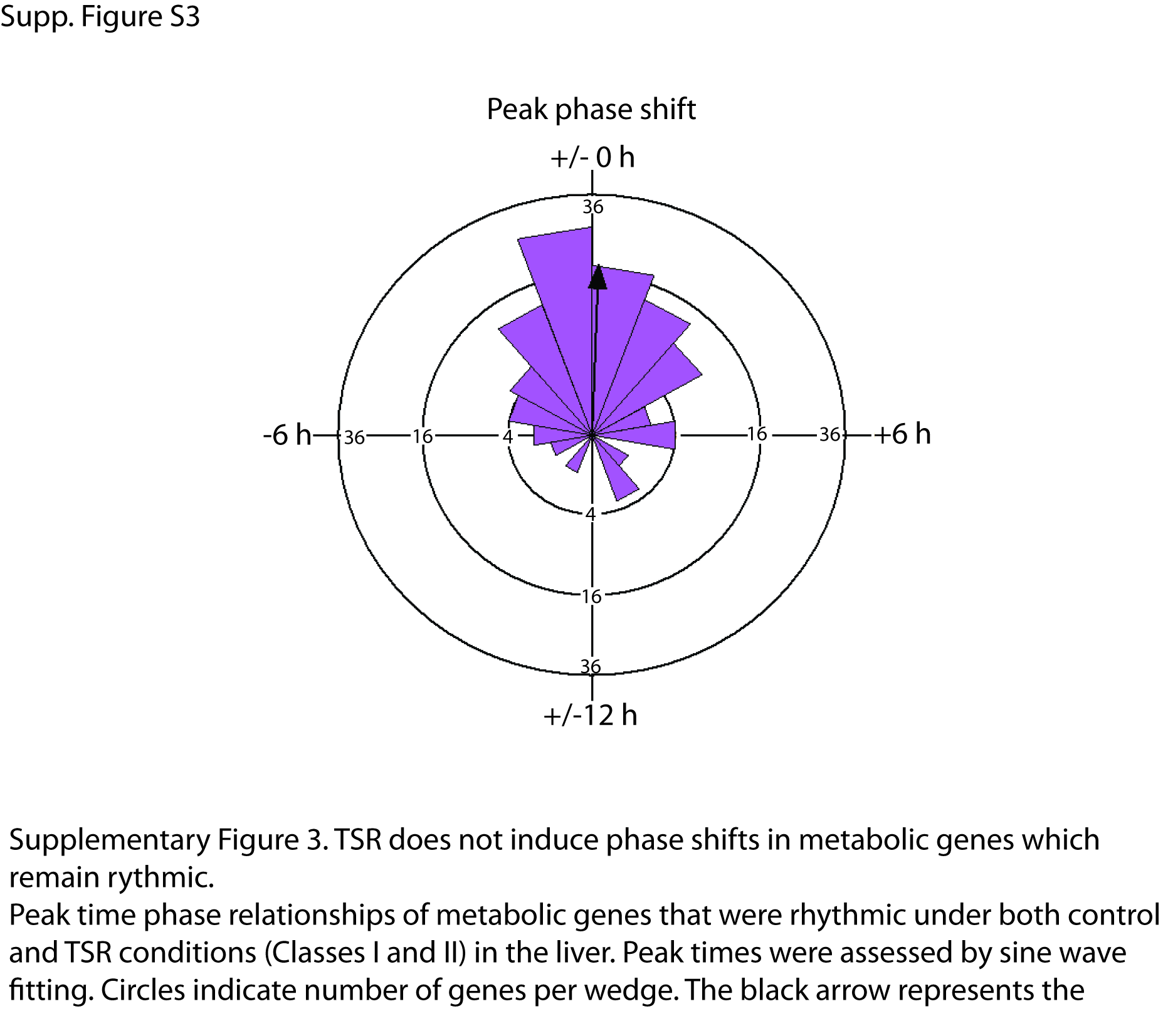

Supplement: Figure S3 — Peak time phase relationships of metabolic genes that were rhythmic under both control and TSR conditions (Classes I and II) in the liver. Peak times were assessed by sine wave fitting. Circles indicate number of genes per 20° wedge. The black arrow represents the normalization vector for all shifts/genes. (TIF) [file pone.0037150.s003.tif]
