# Supplementary material for: Circadian Desynchrony Promotes Metabolic Disruption in a Mouse Model of Shiftwork
Source: PLoS One. 2012 May 21;7(5):e37150. doi: 10.1371/journal.pone.0037150 (PMC3357388; doi:10.1371/journal.pone.0037150)
Supplement: Table S1 — Full gene lists from Figure 3B , including Probe ID, Gene ID, and peak time in both control and TSR mice. (PDF) [file pone.0037150.s004.pdf]

Supp Table 1 Complete list of genes in each catagory from Figure 3 for liver

Liver Class I			
Probe ID	**Gene ID**	**Con Peak**	**TSR Peak**
10344966	Ly96	1	1
10345183	Cdk10	13	13
10345492	Cnnm3	1	1
10345675	Npas2	1	1
10346298	Coq10b	13	13
10347748	Acsl3	19	19
10348000	2810459M11Rik	7	7
10348078		13	13
10349081	Phlpp1	7	7
10349512	Ubxn4	1	1
10349932	Etnk2	13	13
10350535	Tpr	1	1
10352548	Slc30a10	1	1
10353450	Gm4956	19	19
10353450	Gm4956	19	19
10354258	Uxs1	19	19
10354258	Uxs1	19	19
10355514	Tns1	13	13
10355554	Aamp	13	13
10355730	1810031K17Rik	7	7
10356084	Irs1	1	1
10356172	5033414K04Rik	1	1
10356406	Ngef	13	13
10357280	Insig2	1	1
10358607	Hmcn1	19	19
10359190	Fam20b	13	13
10359428	Aph1a	13	13
10359525	Bat2d	1	1
10360391	Ifi203	1	1
10361760	Timm8a1	13	13
10361846	Reps1	7	7
10361926	Map3k5	13	13
10362314	Ptprk	1	1
10363241	Gcc2	7	7
10363372	Pla2g12b	1	1
10363512	Sar1a	13	13
10364518	Ptbp1	7	7
10365116	Dohh	13	13
10365344	Tcp11l2	7	7
10365769	Hal	1	1
10366707	Avpr1a	1	1
10366956	Stat6	7	7
10366983	Tmem194	7	7
10367337	Rnf41	13	13
10367728	Map3k7ip2	1	1
10367768		1	1
10368495		1	1
10368681	Rfpl4b	13	13
10368806	Smpd2	13	13
10369290	Ddit4	19	19
10369290	Ddit4	19	19
10369379	Slc29a3	1	1
10369413	Sgpl1	19	19
10369413	Sgpl1	19	19
10370446	Trappc10	13	13
10371400	Cry1	19	19
10371400	Cry1	19	19
10372844	Rassf3	7	7
10373101	Pip4k2c	7	7
10373197	Inhbc	19	19
10373452	Gm129	13	13
10373890	Tbc1d10a	7	7
10375125	Ssbp1	1	1
10375634	Mapk9	1	1
10376144	Fnip1	7	7
10376733	Map2k3	13	13
10377751	Asgr1	13	13
10377927	Rnf167	7	7
10378385	Spata22	1	1
10379204	Poldip2	13	13
10380560	Zfp652	1	1
10381419	Nbr1	7	7
10382425	Gprc5c	7	7
10383395	Slc25a10	13	13
10383671	Drg1	13	13
10383982	Znrf3	7	7
10384051	Gck	13	13
10384811	Ccdc104	19	19
10384811	Ccdc104	19	19
10385747	Phf15	13	13
10385814	Leap2	19	19

10385814	Leap2	19	19
10386219	Zfp39	7	7
10386230	Rnf187	13	13
10386427	Flcn	7	7
10386495		7	7
10386850	Ncor1	1	1
10386934	Wsb2	13	13
10387180	Ndel1	13	13
10387922	Slc25a11	13	13
10388880	Tmem97	13	13
10388938	Wsb1	1	1
10389561	Dhx40	7	7
10390227	Ube2z	7	7
10390574	Fbxl20	7	7
10390691	Nr1d1	7	7
10392484	Abca8b	1	1
10394209	Dnmt3a	7	7
10394448	Pum2	7	7
10394593	Fam49a	1	1
10394627		1	1
10395259	Nampt	13	13
10396270	Dact1	13	13
10398299	AI132487	7	7
10399478	Lpin1	13	13
10399680	Cys1	13	13
10399801	Sntg2	7	7
10399897	Hbp1	7	7
10401805	Snw1	13	13
10402665	Cdc42bpb	19	19
10402766	Akt1	13	13
10404132	Cmah	1	1
10405781		13	13
10405894	Ptdss1	13	13
10407307	Mocs2	19	19
10407307	Mocs2	19	19
10409276		19	19
10409276		19	19
10410287	Zfp458	1	1
10410302	E130120F12Rik	1	1
10412100	Map3k1	1	1
10412298	Itga1	1	1
10413100	Myst4	1	1
10413640	Nek4	1	1
10414211	Mbl1	1	1
10414269		13	13
10415319	Irf9	7	7
10415574		1	1
10415725	Spata13	7	7
10416464	Zc3h13	1	1
10417167	Clybl	13	13
10417544	Acox2	1	1
10417734	Nr1d2	7	7
10417869	Anxa7	13	13
10419216	Gnpnat1	7	7
10419240	Ddhd1	1	1
10419288	Gch1	7	7
10419854	Slc7a8	1	1
10420114	Tgm1	13	13
10421197	Chmp7	7	7
10421697	9030625A04Rik	19	19
10423134	Zfr	1	1
10424833	Maf1	7	7
10425302	Gtpbp1	7	7
10425601	Tef	13	13
10427095	Tenc1	7	7
10427744	Rai14	1	1
10428998	Asap1	1	1
10429160	St3gal1	1	1
10430748	Rangap1	13	13
10431558	Odf3b	7	7
10431601	Arsa	7	7
10433057	Calcoco1	7	7
10434094	Klhl22	1	1
10434758	St6gal1	7	7
10435162	Lrch3	1	1
10436804	Mrap	19	19
10436804	Mrap	19	19
10436865	Ifngr2	7	7
10437451		7	7
10437992	Dnm1l	1	1
10438769	Cldn1	1	1
10439411	Nr1i2	13	13
10439854	Bbx	1	1
10440238	Nsun3	1	1

10443940	Zfp422-rs1	1	1
10444578		13	13
10444978	Gnal1	13	13
10445442	Gtpbp2	7	7
10445496	Yipf3	7	7
10446063	Tnfaip8l1	7	7
10446986	Crim1	13	13
10447361	Ttc7	13	13
10448195	3110048L19Rik	1	1
10448676	Slc9a3r2	13	13
10449386	D17Wsu92e	13	13
10449452	Fkbp5	13	13
10449999	Zfp101	1	1
10450369	Hspa1a	13	13
10450622	2610110G12Rik	1	1
10451421	Klhdc3	7	7
10451893	Stap2	13	13
10451918		13	13
10453276	Thada	1	1
10453747	Colec12	19	19
10454063	Rsl24d1	13	13
10454414	Pik3c3	7	7
10454741	Kdm3b	1	1
10455961	Iigp1	7	7
10456254	Nedd4l	1	1
10457508	Npc1	7	7
10457583		13	13
10457745	D030074E01Rik	1	1
10458164	Cdc23	1	1
10458731	Mcc	1	1
10458913	Cep120	7	7
10459772	Lipg	13	13
10460057		7	7
10460108	Gnpnat1	7	7
10460157	Cpt1a	7	7
10460221	Chka	1	1
10460732	Znhit2	13	13
10461808	Olfr1474	13	13
10462091	Klf9	7	7
10464425	Grk5	1	1
10465209	Mtvr2	13	13
10465215	Mtvr2	13	13
10465485	Esrra	13	13
10466304	Dtx4	1	1
10466745	Tjp2	1	1
10467153	Slc16a12	13	13
10467206	Ppp1r3c	1	1
10468762	4930506M07Rik	1	1
10468828	Eif3a	1	1
10470109	Fbxw5	1	1
10470427	Wdr5	13	13
10471912	Kynu	1	1
10471978	Epc2	7	7
10472097	Fmnl2	1	1
10473432	Tnks1bp1	13	13
10473919		19	19
10473919		19	19
10476033	Stk35	13	13
10478219	Plcg1	13	13
10481401	Zer1	7	7
10483163	Grb14	7	7
10483410	Abcb11	1	1
10483667	Cir1	7	7
10484389	Tfpi	1	1
10485466	Cat	1	1
10485514	Caprin1	1	1
10486029	Atpbd4	1	1
10486203	Ino80	7	7
10486819	Catsper2	1	1
10488797	Pxmp4	1	1
10489246	Mafb	13	13
10489484	Sdc4	7	7
10490777	Zfhx4	19	19
10490777	Zfhx4	19	19
10490826	Zbtb10	7	7
10491106	Pld1	1	1
10491564	4932438A13Rik	1	1
10491599	4932438A13Rik	1	1
10491605	4932438A13Rik	1	1
10491607	4932438A13Rik	1	1
10491611	4932438A13Rik	1	1
10491613	4932438A13Rik	1	1
10491621	4932438A13Rik	1	1
10491623	4932438A13Rik	1	1

10491625	4932438A13Rik	1	1
10491627	4932438A13Rik	1	1
10491629	4932438A13Rik	1	1
10492049	Fam48a	1	1
10492330	P2ry1	13	13
10492689	Pdgfc	1	1
10494001	Tdpoz4	13	13
10494312	Aph1a	13	13
10494832	Sike1	1	1
10495163	Tmem77	1	1
10495685	Arhgap29	1	1
10495873		13	13
10496023	Casp6	7	7
10496159	Ints12	1	1
10496324	Slc39a8	1	1
10498185	Tra2b	1	1
10498623	Kpna4	1	1
10499108	Glt28d2	1	1
10500272	Gm129	13	13
10500545	Hsd3b5	7	7
10500720	Slc22a15	19	19
10500720	Slc22a15	19	19
10501734	Palmd	7	7
10502224	Sgms2	7	7
10502284	Tet2	1	1
10503584	Coq3	7	7
10503711	Casp8ap2	1	1
10504582	1300002K09Rik	1	1
10505270	Slc31a2	1	1
10505461	Atp6v1g1	19	19
10506188	Pgm2	13	13
10506298	Leprot	7	7
10506470	1700024P16Rik	19	19
10506470	1700024P16Rik	19	19
10508454	Bsdc1	7	7
10508619	Pum1	7	7
10510270		1	1
10511665	Necab1	7	7
10512640	Gne	13	13
10512766	Trim14	1	1
10514298	Mrpl48	13	13
10515156	Minpp1	13	13
10516064	Mfsd2	13	13
10517328	Tmem50a	13	13
10517345	Srrm1	1	1
10517727	Klhdc7a	7	7
10518069	Efhd2	13	13
10518428	Clcn6	19	19
10518428	Clcn6	19	19
10519659	Anapc5	13	13
10519691	Anapc5	13	13
10520048	Mll5	1	1
10521174	Rnf4	7	7
10521702	4930431F12Rik	19	19
10521702	4930431F12Rik	19	19
10522802	Ythdc1	1	1
10524034	Idua	1	1
10524941	Fbxo21	7	7
10525086	Slc24a6	7	7
10525296	Brap	7	7
10525893	Aacs	13	13
10526356	Rhbdd2	13	13
10526363	Por	13	13
10526842	Zfp157	19	19
10526842	Zfp157	19	19
10527133	Wipi2	13	13
10527475	Zfp655	7	7
10527528	Smarce1	1	1
10528723	Mll3	1	1
10529758	Bod1l	1	1
10530733	Clock	1	1
10531323	G3bp2	1	1
10531529	Cnot6l	7	7
10531707	Lin54	7	7
10532133	Evi5	1	1
10532944	Mlec	1	1
10533285	Ptpn11	13	13
10535866	Ubl3	13	13
10537292	1810058I24Rik	13	13
10539111	Tmem150	19	19
10539111	Tmem150	19	19
10539486	Zfml	1	1
10539653	Tprkb	7	7
10540248	Mitf	1	1

10540812	Tatdn2	13	13
10541307	Usp18	7	7
10541446	Cpamd8	19	19
10541446	Cpamd8	19	19
10541545	Necap1	7	7
10541711	Phb2	13	13
10542112	Clec2h	7	7
10542200	Gabarapl1	7	7
10542477	Pik3c2g	19	19
10542477	Pik3c2g	19	19
10542522	Plekha5	1	1
10543879	Wdr91	1	1
10544089	Zc3hav1	7	7
10545065	Gprin3	7	7
10545308	Kdm3a	7	7
10545697	Dguok	7	7
10546450	Adamts9	1	1
10547521	Atp6v1e1	13	13
10548735	Dusp16	1	1
10551162	Atp5sl	13	13
10551173	Exosc5	13	13
10551226	Cyp2a4	13	13
10551250		19	19
10551250		19	19
10551282		13	13
10553092	Dbp	7	7
10554569	Fam103a1	13	13
10554714	Olfr291	13	13
10556169	Eif3f	13	13
10556463	Arntl	1	1
10558707	Ric8	13	13
10558723	Psmd13	13	13
10558921	Pnpla2	7	7
10558961	Tspan4	13	13
10560015	Rnf141	1	1
10560709	Pvr	7	7
10561008	Ceacam1	1	1
10561063	Bckdha	13	13
10562911	Tbc1d17	7	7
10563130	Aldh16a1	7	7
10564343	Tjp1	7	7
10564736	Polg	7	7
10565156	Homer2	13	13
10565341	Gm6155	13	13
10565775	Dgat2	13	13
10565846	Spcs2	1	1
10566926	Rnf141	1	1
10567335	Gde1	13	13
10568050	Aldoa	13	13
10568115	Mvp	13	13
10568436		7	7
10568785	Bnip3	13	13
10569646	Ccnd1	13	13
10569786	Pnpla6	19	19
10569786	Pnpla6	19	19
10569953	Snapc2	7	7
10570982	Fgfr1	13	13
10571444	Slc7a2	7	7
10571530		1	1
10571567	Sorbs2	1	1
10572130	Lpl	1	1
10573483	Prdx2	13	13
10574404	Setd6	13	13
10575029	6030452D12Rik	1	1
10575867	Mlycd	7	7
10576152	Trappc2l	13	13
10576258	Cdk10	13	13
10576610	Pard3	7	7
10576719		1	1
10577604	Agpat6	13	13
10579181	Rfxank	1	1
10580056	Lphn1	19	19
10580056	Lphn1	19	19
10580807	Kifc3	19	19
10580875	Gtl3	7	7
10581340	Ranbp10	7	7
10581560	Psmd7	1	1
10581625	2400003C14Rik	13	13
10582069	Sdr42e1	7	7
10582231	Fbxo31	13	13
10583573	Atg4d	13	13
10584634	Usp2	13	13
10584758		13	13
10584803	1500035H01Rik	7	7

10585417	Idh3a	13	13
10585467	C630028N24Rik	7	7
10586074		19	19
10586074		19	19
10586368	Clpx	1	1
10586724	Narg2	1	1
10587042	Rsl24d1	13	13
10588243	Ryk	13	13
10588429	Pik3r4	7	7
10588691	Hyal1	1	1
10589087	Prkar2a	19	19
10589087	Prkar2a	19	19
10590438	C730027P07Rik	1	1
10590635	Ccr5	1	1
10591263	Fbxl12	1	1
10591668	Rgl3	1	1
10592237	Ei24	1	1
10592289	Ccdc15	19	19
10592289	Ccdc15	19	19
10593966	Csk	7	7
10596318	Nudt16	7	7
10597531	Rbms3	1	1
10597758	Csrnp1	13	13
10598013	Ccr5	1	1
10598456	Otud5	7	7
10598586	Xk	1	1
10598798	Kdm6a	1	1
10599032	Wdr44	1	1
10599192	Lonrf3	19	19
10599192	Lonrf3	19	19
10601099	Med12	1	1
10601192	Taf1	1	1
10601303		1	1
10601588	3110007F17Rik	7	7
10601819	Armcx5	1	1
10602454	Fam120c	1	1
10602827	A830080D01Rik	1	1
10607774	Mospd2	1	1
Liver Class II			
Probe ID	**Gene ID**	**Con Peak**	**TSR Peak**
10344713		19	13
10345206		19	13
10346303	Hspe1	19	13
10347564	Dnajb2	1	7
10348321	Dgkd	7	13
10349316	Tmem185b	13	7
10349752	Elk4	7	1
10350349	Dennd1b	7	1
10350792	Tor1aip1	1	7
10351224	F5	13	1
10351269	4930455F23Rik	1	7
10351852	Crp	19	1
10352119	Pppde1	19	1
10352125	Gm9982	19	1
10352867	Plxna2	1	19
10353181	Lactb2	1	7
10353346	Pkhd1	7	1
10353849	4632411B12Rik	7	13
10355532	Tns1	19	13
10355567	Tmbim1	7	13
10356001	Cul3	7	1
10356593	Hes6	13	19
10356601	Per2	13	19
10356859	Dtymk	19	1
10357239	Tmem37	13	19
10357454	Dars	7	1
10357579	Mapkapk2	1	13
10357630	Srgap2	7	19
10358454	Rbm3	13	1
10359571	Fmo1	13	19
10360415	Grem2	13	7
10360589	Ahctf1	19	1
10361104	Ppp2r5a	7	19
10361869	Nhsl1	1	7
10361979	Bclaf1	7	1
10362052	Aldh8a1	7	19
10362171	Stx7	19	1
10362210	Med23	7	1
10362379	Echdc1	19	1
10362803	Cd164	7	1
10363281	Ranbp2	13	1
10363522	Tysnd1	19	13
10363706	Jmjd1c	1	19

10364049		7	19
10364683	Stk11	7	13
10365302	A230046K03Rik	19	1
10365545	Pah	19	13
10365658	Uhrf1bp1l	13	1
10365974	Dcn	1	19
10366238	Ppp1r12a	7	1
10367224	Stat2	1	19
10367600	Esr1	13	19
10367816		19	1
10368289	Enpp1	13	19
10368317	Enpp3	7	1
10368700	BC021785	19	1
10368888	Foxo3	13	7
10369909		1	19
10370376	Pfkl	19	13
10370931	Mknk2	13	7
10371356	Appl2	7	1
10371506	Stab2	1	19
10371830	Actr6	7	1
10371970	Amdhd1	1	7
10372177	Tmtc2	7	13
10372457	Tbc1d15	7	1
10372503	Lgr5	7	1
10372618	Frs2	7	1
10372656	Cpsf6	7	1
10372988	Slc16a7	13	19
10373223	Lrp1	7	13
10374455	Spred2	1	7
10374590	Xpo1	19	1
10375002	Cpeb4	7	1
10375529	Zfp62	19	1
10375820	Clk4	7	1
10376163	Rapgef6	7	1
10378914	Myo18a	13	7
10379262	Nf1	7	1
10379445	Zfp207	7	1
10379779	Ddx52	7	1
10380534	Spop	19	1
10380840	Crkrs	7	1
10381172	Stat5a	1	19
10382152	Helz	7	1
10382271	Arsg	7	1
10383758	Tug1	7	1
10384572	1110067D22Rik	1	19
10385325	Pttg1	7	13
10385353	Adra1b	1	19
10385822	Gm10447	19	1
10386473	Srebf1	13	19
10386624	Tmem11	7	13
10387557		19	13
10388377	Srr	19	1
10388451	Wdr81	1	7
10389797	Stxbp4	19	1
10390454	E130012A19Rik	19	13
10391286	Stat5b	1	7
10391828	C1ql1	19	13
10392318	Bptf	7	1
10392440	Slc16a6	19	1
10392601	Abca6	7	1
10392856	Nat9	19	13
10392894	Fads6	7	13
10393431	Tk1	13	19
10393904	Notum	7	13
10394173	Asxl2	7	1
10394288	Itsn2	7	1
10394783	1700030C10Rik	19	1
10394786	1700030C10Rik	19	1
10396346	1810048J11Rik	7	19
10396740	Gphn	7	1
10396950		19	13
10397085	Rbm25	13	19
10397518	Ahsa1	19	13
10398288	Yy1	7	13
10398578	Tecpr2	19	1
10398881	Gm5188	19	13
10399265	Ncoa1	7	1
10399354		19	1
10400006	Ahr	7	1
10400718	Sos2	7	1
10401289	Slc10a1	7	1
10401616	Mlh3	19	1
10402211	Fbln5	1	7
10402368	Serpina6	13	19

10402473	Clmn	13	7
10402542	Atg2b	7	1
10403558	Ero1lb	19	1
10403955	Hist1h2ao	1	13
10404045	Hist1h2ad	1	13
10404178	BC005537	19	13
10404612	Rreb1	7	1
10405911	Zfp759	19	1
10406482	Ccnh	19	1
10406614	Mtx3	7	1
10406681	Ap3b1	7	1
10406718	Wdr41	7	1
10407370	4833420G17Rik	7	1
10407993	Fusip1	19	1
10408111	Hist1h2ao	19	13
10408162	Zfp322a	19	1
10408251	Slc17a4	1	7
10408348	Mrs2	7	19
10408629	1300014I06Rik	1	19
10408656	Peci	7	13
10408915	Dtnbp1	7	13
10409204	Gm10784	19	13
10410273	Zfp708	19	1
10410364	6720487G11Rik	7	1
10410386	E430024C06Rik	19	1
10410388	E430024C06Rik	19	1
10410617	Pdcd6	7	13
10410644	Zfp72	19	1
10411899	2410002O22Rik	19	1
10412607	Abhd6	13	19
10413981	Gm626	19	13
10414449	Mudeng	7	1
10415052	Mmp14	1	19
10415911	Kif13b	19	1
10417124	B930095G15Rik	7	1
10418251	Appl1	19	1
10419223	Fermt2	1	19
10420390	Xpo4	13	1
10420497	Gm6907	13	1
10421672	1200011I18Rik	7	1
10422194	Rbm26	7	1
10423740		7	13
10423921	Oxr1	7	1
10424113	Mal2	19	1
10424467	Phf20l1	13	1
10424929	Adck5	1	7
10425207	H1f0	1	7
10426169	1300018J18Rik	7	13
10426921	Dazap2	7	1
10427235	Prr13	19	7
10427454	Card6	1	7
10427772	Tars	19	1
10428103	Stk3	7	1
10428619	Enpp2	13	19
10430649	Cbx7	7	13
10431856	Sfrs2ip	7	1
10433114	Itga5	1	19
10433311	Mgrn1	7	13
10433633	Mkl2	19	1
10433660	Bfar	7	1
10433910	Top3b	1	19
10434668	Tmem97	13	19
10435489	Ccdc58	7	1
10436209	Cblb	7	1
10436678	Gabpa	7	1
10436873	Son	7	1
10437224	Mx2	7	1
10437236	B230307C23Rik	19	1
10437330	Crebbp	1	7
10437664	Dexi	1	19
10437778	Parn	7	1
10438340	Comt1	1	7
10439762	Ahcy	19	13
10440770	Sfrs15	7	1
10441787		7	1
10442224	BC049807	19	1
10442341	Srrm2	7	1
10442625	Igfals	19	13
10443786	Pde9a	1	19
10444244	Tap1	1	7
10445607	Pex6	7	13
10448424	Pdpk1	7	1
10448506		19	1
10449225	Decr2	7	13

10449327	Lemd2	7	13
10449926	Zfp799	19	1
10450206	Rnf5	19	13
10450605	Tubb5	1	7
10453026	Prkd3	7	1
10453461	Fbxo11	7	1
10453544	Mettl4	1	7
10453636	Svil	1	19
10453759	Gm10554	19	1
10454105	Dsg1c	13	19
10454398	AW554918	7	1
10454447	Sap130	7	1
10454546	Map3k2	7	1
10454782	Egr1	1	7
10455588		19	13
10455852	Prrc1	1	7
10456296	Malt1	7	19
10457465	Esco1	7	1
10457780	Fam59a	13	7
10458098	Fam13b	19	1
10458843	Sema6a	1	19
10460544	Yif1a	13	7
10460591	Fibp	7	13
10461568	Prpf19	7	13
10461844	Gnaq	7	1
10461979	Aldh1a1	19	1
10462504	Minpp1	7	13
10463121	Zfp518	19	1
10463209	4933411K16Rik	7	19
10463224	Marveld1	13	19
10464070	Vti1a	7	19
10464084	Tcf7l2	7	1
10464415	Fam45a	19	1
10464560	Aldh3b1	19	13
10464572	Ndufv1	19	13
10465314	Capn1	13	7
10465474	Prdx5	19	13
10466288	Olfr1428	19	13
10466976	Gldc	19	1
10467368	Ctdspl2	7	1
10468253	Nt5c2	13	19
10468311	Sh3pxd2a	7	1
10468885	Zfp826	19	1
10468949	Dclre1c	7	1
10469335	Stam	7	1
10469425	Arl5b	13	1
10469475	Mllt10	7	1
10469772		7	1
10469867	Pnpla7	1	7
10471256	Nup214	7	1
10472162	Gpd2	7	1
10472538	Dhrs9	1	7
10473473	Olfr1022	19	13
10473582	Olfr1161	1	13
10473690	Fnbp4	7	1
10473981	Ambra1	7	1
10474006	Phf21a	1	19
10475335	Pdia3	19	1
10475405	Eif3j	19	1
10476314	Prnp	19	13
10476349	1110034G24Rik	13	7
10476834	Xrn2	7	1
10477649	Acss2	1	19
10477815	Phf20	19	13
10478447	Stk4	1	7
10478938	Hax1	19	7
10479047	Pck1	13	7
10479182		19	1
10479189	Gm14434	19	1
10479192		19	1
10479195		19	1
10479198	Gm14434	19	1
10479902	Dhtkd1	7	19
10480258	Nsun6	7	1
10480329	Dnajc1	19	1
10480725	BC029214	1	19
10481525	Tor1a	7	13
10482330	Atp6v1g1	19	13
10482448	Zeb2	1	19
10482868	Wdsub1	7	13
10482880	Baz2b	7	1
10483819	Ttc30b	1	7
10483822	Ttc30a2	1	7
10484402	Ctnnd1	7	1

10485357		19	13
10485656	Elp4	19	1
10487011	Gatm	1	19
10487277	Trpm7	7	1
10487945	Prei4	13	7
10488322	A230067G21Rik	7	1
10488469	Btbd1	19	1
10488816	Ahcy	19	13
10490259	Gm14434	19	1
10490262	Gm14434	19	1
10490265	Gm14391	19	1
10490268	Gm14434	19	1
10490273	100043387,00000000	19	1
10490291	Gm14434	19	1
10490690	Uckl1	7	1
10491597	4932438A13Rik	1	19
10491601	4932438A13Rik	19	1
10491603	4932438A13Rik	19	1
10491609	4932438A13Rik	19	1
10491615	4932438A13Rik	19	1
10491617	4932438A13Rik	19	1
10491619	4932438A13Rik	19	1
10492709	Accn5	19	13
10493292	2810403A07Rik	7	1
10493798	S100a16	19	13
10494536	Pdzk1	19	13
10496727	Ddah1	1	7
10498319	Serp1	19	1
10498477	E130311K13Rik	1	7
10499045	Trim2	1	19
10499128	Rnu73a	7	19
10499716	Ubap2l	7	1
10500610	Fam46c	13	7
10501567	Rnpc3	7	1
10501586	S1pr1	1	19
10501699	Agl	7	19
10501778	Ptbp2	7	1
10501795	Rwdd3	1	19
10503036	Sfrs11	7	1
10503359	C430048L16Rik	19	1
10503363	Rbm12b	19	1
10503926	Rars2	7	1
10504127	Ccl21a	19	7
10504154	Ccl21a	19	7
10504183	Ccl21a	19	7
10504755		19	13
10505008	Slc44a1	1	19
10505028	Slc44a1	19	1
10505044	Fktn	19	1
10508392	Rnf19b	13	7
10509063	Il22ra1	7	19
10509441	Ece1	7	19
10509601	Tmco4	7	13
10510129	Dhrs3	1	7
10510172	LOC100041490	1	7
10510256	2510039O18Rik	7	13
10510624	Klhl21	7	13
10511935	Pnrc1	1	19
10512308	Sigmar1	1	19
10512377	Ccl21a	19	7
10512886	Acnat2	1	19
10512892	Acnat1	19	1
10513362	Gm12528	13	19
10513551	Fkbp15	7	1
10513884	Tle1	7	1
10514791	Pcsk9	13	19
10516296	Thrap3	7	1
10516994	Fam76a	13	7
10517421	Pnrc2	19	1
10517689	Pqlc2	7	1
10518350		1	7
10518352		1	7
10518743	H6pd	1	7
10519324	Cdk6	7	1
10519951	Pion	7	1
10520010	Pmpcb	19	13
10520483	D5Wsu178e	19	1
10521057	Maea	1	13
10521068	4933407H18Rik	7	1
10521222	Add1	1	7
10522160	N4bp2	13	7
10523021	Slc4a4	7	1
10523579	Arhgap24	19	1
10524079		19	1

10524082	2310001H12Rik	19	1
10524369	Hps4	1	7
10525111	Ddx54	7	13
10525718		19	13
10526482	Polr2j	7	13
10527327	Bri3	13	7
10527575	Pan3	7	1
10528457	Orc5l	7	1
10529093	Gtf3c2	7	1
10529226	Rbks	13	19
10530225	Pds5a	7	1
10530492	Nfxl1	13	1
10531066	Ugt2a3	19	1
10531183	Adamts3	19	13
10531215	Ankrd17	7	1
10532301		19	1
10532305	4930522L14Rik	19	1
10532310	4930522L14Rik	19	1
10532582	Myo18b	19	13
10534842	Gnb2	7	13
10535739	Usp12	19	13
10535759	Lnx2	1	19
10535894	Hmgb1	7	1
10535956	Stard13	1	19
10536635	A430107O13Rik	7	1
10536818	Calu	19	1
10537102	Exoc4	7	1
10537375	Ubn2	7	1
10538253	Mpp6	19	1
10538269		19	1
10538459	Aqp1	7	13
10538526	Avl9	1	7
10539104	0610030E20Rik	1	7
10539606	Cct7	19	13
10539894	Mgll	13	19
10540493	Edem1	19	1
10540554	Setd5	7	1
10540727	Creld1	19	7
10541216	Kdm5a	7	1
10541441	Gm10319	1	19
10542172	Clec1b	7	1
10542378	Atf7ip	1	7
10542615	Slco1b2	7	1
10542885	2810474O19Rik	7	1
10542959	Bet1	19	1
10543017	Pdk4	1	19
10543551	Rbm28	19	1
10543785	AB041803	7	1
10544885	Fkbp14	19	1
10545409	Vamp8	7	13
10547404	Erc1	19	7
10547410	Erc1	19	1
10548030	Cd9	1	7
10548697		19	13
10548996	Slco1a4	7	1
10549256	Kras	19	1
10550003	Zfp606	19	1
10551752	4932431P20Rik	19	13
10552353		19	1
10552418	Etfb	19	13
10553354	Nav2	7	19
10555087		7	19
10555758	Olfr582	19	13
10555875	Olfr653	19	13
10556113	Rbm3	13	1
10556242		13	1
10556381		1	19
10556487	A630005I04Rik	1	19
10556528	Pde3b	19	1
10556701	Acsm5	7	19
10557206	Rbbp6	7	1
10557211	Rbbp6	7	1
10557233	Tnrc6a	7	1
10557831	Myst1	19	13
10559333	Shank2	1	19
10559341	Shank2	1	19
10560131	2810007J24Rik	7	1
10560181	Gltscr2	7	13
10560532	Lrrc68	13	7
10562605	AI987944	7	1
10563770	Csrp3	7	13
10563895		19	7
10564451	Mef2a	7	1
10564467	Lrrc28	13	19

10565178	Btbd1	19	1
10565479	I7Rn6	19	7
10565609	Thrsp	13	19
10565738	2210018M11Rik	7	1
10566132	Rhog	1	7
10567743	Nsmce1	1	13
10569024	Sigirr	19	13
10569848	Stxbp2	7	13
10570291	F10	19	13
10571266	Ppp2cb	7	13
10572146	Atp6v1b2	1	7
10572733	BC049349	7	1
10573867	Rbl2	7	1
10575153	Cyb5b	19	1
10575873	Osgin1	1	7
10576598	1810063B05Rik	13	19
10578264	Msr1	1	19
10578955		7	19
10579799	Tmem184c	19	1
10580771	Ciapin1	13	19
10580955		1	19
10582006	LOC100048095	19	13
10582474	Chmp1a	7	13
10582882		19	13
10582888		19	13
10582916		19	13
10583242	Sesn3	1	7
10583314	Taf1d	7	1
10583485	A230050P20Rik	1	19
10585358	Npat	7	1
10585572	Hmg20a	7	1
10585874	Hexa	7	13
10586920	Rfx7	1	19
10587266	Gclc	1	19
10587558	Dopey1	7	1
10587639	Nt5e	1	19
10587854	Slc9a9	1	19
10588154	Stag1	7	1
10588192	Msl2	7	1
10588223	Anapc13	1	13
10590563	Lars2	1	13
10590860	9030420J04Rik	19	1
10591035	5830418K08Rik	7	1
10591816	Dpy19l1	19	1
10593526	Atm	7	1
10594460	Dis3l	1	13
10594549	Gm10144	7	19
10595126	Fbxo9	7	1
10598389	Wdr45	7	13
10598626	Tspan7	1	13
10598837		7	19
10598956	Araf	7	19
10599369	Xiap	19	1
10599554	Rbmx2	19	13
10599826	F9	19	1
10600688	Map3k7ip3	7	1
10601312	Gm10454	19	1
10601616	Diap2	7	1
10602223	Alg13	7	1
10602501	Huwe1	7	1
10602772	Rps6ka3	7	1
10602964		7	19
10603469	Rbm3	13	1
10603708	Cask	19	1
10604424	Zfp280c	7	1
10604799	Atp11c	7	1
10605090	Idh3g	7	13
10606301	Magt1	19	1
10607002	Gm7091	19	13
10607560		19	13
10607862		19	13
10607950		1	19
Liver Class III			
Probe ID	**Gene ID**	**Con Peak**	**TSR Peak**
10344618		19	N/A
10344637	Atp6v1h	7	N/A
10344723	Rrs1	19	N/A
10344741		19	N/A
10344950	LOC675046	19	N/A
10344960	Tmem70	19	N/A
10345065	Gsta3	19	N/A
10345203	Paox	1	N/A
10345357	Imp4	7	N/A

10345368	D1Ertd448e	19	N/A
10345438		19	N/A
10345442	Hs6st1	13	N/A
10345482	Cnnm4	1	N/A
10345626	Eif5b	1	N/A
10345706	D1Bwg0212e	1	N/A
10345904	AI597479	19	N/A
10346191	Stat1	7	N/A
10346651	Bmpr2	1	N/A
10346747	Cyp20a1	1	N/A
10346762		19	N/A
10346780		13	N/A
10346838	Pard3b	1	N/A
10346941		7	N/A
10347033	Crygf	19	N/A
10347117	Cps1	19	N/A
10347254	Smarcal1	1	N/A
10347310	Pnkd	1	N/A
10347335	Slc11a1	19	N/A
10347774	Mrpl44	1	N/A
10347796	Rhbdd1	1	N/A
10347862	Mff	7	N/A
10347948	Sp100	1	N/A
10347980	Itm2c	1	N/A
10348076	Gm10200	1	N/A
10348087	Cops7b	7	N/A
10348096	Dis3l2	7	N/A
10348234	Neu2	19	N/A
10348277	Atg16l1	7	N/A
10348299	5830472F04Rik	19	N/A
10348653	Gpc1	13	N/A
10348702	Agxt	1	N/A
10348932	Zh2c2	13	N/A
10349206		19	N/A
10349431	Acmsd	1	N/A
10349453	Rab3gap1	7	N/A
10349559	Yod1	13	N/A
10349562	AA986860	13	N/A
10349569		13	N/A
10349580	Pigr	1	N/A
10349610	Lgtn	13	N/A
10349711	Slc41a1	7	N/A
10349724	Rab7l1	19	N/A
10349733	Nucks1	19	N/A
10349744	Slc45a3	19	N/A
10349782	Nuak2	13	N/A
10349793	Dstyk	19	N/A
10349876	Plekha6	7	N/A
10350003	Cyb5r1	7	N/A
10350099	Ptprv	19	N/A
10350113	Arl8a	13	N/A
10350188	Tmem9	13	N/A
10350331	Zfp281	13	N/A
10350489	Uchl5	19	N/A
10350594	Ivns1abp	13	N/A
10350733	Rgs16	7	N/A
10350766	Stx6	1	N/A
10350977	4930523C07Rik	7	N/A
10351047	Cenpl	7	N/A
10351163	Scyl3	7	N/A
10351259	Slc19a2	1	N/A
10351277	Nme7	7	N/A
10351345		19	N/A
10351347	Creg1	7	N/A
10351636	Refbp2	19	N/A
10351738	Pex19	7	N/A
10352234	Itpkb	1	N/A
10352242	Parp1	19	N/A
10352306	Pycr2	19	N/A
10352448	Dusp10	1	N/A
10352576	Esrrg	19	N/A
10352767	Nek2	1	N/A
10352827	A130010J15Rik	1	N/A
10352954		7	N/A
10353036	Gm10567	19	N/A
10353135	Ncoa2	7	N/A
10353167	Tram1	19	N/A
10353189	Gm5523	19	N/A
10353272	Stau2	7	N/A
10353296		19	N/A
10353309	Jph1	13	N/A
10353311	Jph1	13	N/A
10353438	Tram2	7	N/A
10353624	ND4L	19	N/A

10353626	ATP6	19	N/A
10353630	COX2	19	N/A
10353707	Ptp4a1	13	N/A
10353794	Fam168b	7	N/A
10353878	Ankrd23	19	N/A
10354085	Eif5b	7	N/A
10354168	Tbc1d8	13	N/A
10354233	Tgfbrap1	13	N/A
10354273		13	N/A
10354389	Slc39a10	1	N/A
10354404	Dnajb6	13	N/A
10354411		13	N/A
10354418		13	N/A
10354468		19	N/A
10354494	Nab1	7	N/A
10354647	Pgap1	7	N/A
10354649	Pgap1	7	N/A
10354868	Fam126b	7	N/A
10354897	Trak2	13	N/A
10354919	4930408G06Rik	19	N/A
10354979	Als2	19	N/A
10355037	Wdr12	19	N/A
10355050	Raph1	1	N/A
10355069	Ino80d	1	N/A
10355084	Ndufs1	19	N/A
10355214	Idh1	19	N/A
10355246	Acadl	13	N/A
10355278	Erbb4	13	N/A
10355536	Tns1	13	N/A
10355658	Fev	19	N/A
10355706	Ihh	13	N/A
10355806	Tuba4a	1	N/A
10355984	Serpine2	19	N/A
10356262	Gm7582	7	N/A
10356312	Ncl	13	N/A
10356351		13	N/A
10356568	Rab17	7	N/A
10356936	Hisppd1	19	N/A
10356999	Prdx2	19	N/A
10357003	Rnf152	7	N/A
10357051	Kdsr	7	N/A
10357064	Vps4b	1	N/A
10357158	Ralb	1	N/A
10357191	Ptpn4	7	N/A
10357220	Tmem177	1	N/A
10357249	Steap3	1	N/A
10357488	Cd55	1	N/A
10357535	Pfkfb2	13	N/A
10357676	Pctk3	13	N/A
10357870	Prelp	1	N/A
10357878	Adora1	7	N/A
10357948	Ppp1r12b	7	N/A
10358050	Timm17a	13	N/A
10358057	Shisa4	19	N/A
10358191	Camsap1l1	13	N/A
10358210	Nr5a2	7	N/A
10358259	Nek7	7	N/A
10358523	Hmcn1	19	N/A
10358567	Hmcn1	1	N/A
10358583	Hmcn1	1	N/A
10358599	Hmcn1	19	N/A
10358615	Hmcn1	19	N/A
10358726	Tsen15	19	N/A
10358733	Rgl1	1	N/A
10358754	Gm7278	19	N/A
10358849	Dhx9	13	N/A
10358894	Sord	19	N/A
10358999	Xpr1	7	N/A
10359506	Mettl13	13	N/A
10359582	Fmo2	13	N/A
10359677	Blzf1	13	N/A
10359689	Atp1b1	13	N/A
10359734	Dcaf6	7	N/A
10359859		19	N/A
10359861	Mgst3	19	N/A
10359929	Ddr2	1	N/A
10360003	Dusp12	19	N/A
10360026	Uqcrb	19	N/A
10360053	Pcp4l1	19	N/A
10360105	Usp21	1	N/A
10360382	Ifi204	1	N/A
10360540	4930527J03Rik	19	N/A
10360631	Cabc1	1	N/A
10360648	Psen2	19	N/A

10360806	Capn2	19	N/A
10361152	Gstp1	7	N/A
10361156	Rcor3	7	N/A
10361246	G0s2	13	N/A
10361323	Cnksr3	1	N/A
10361660	Lats1	7	N/A
10361680	BC013529	13	N/A
10361828	Cited2	1	N/A
10361882	Nhsl1	19	N/A
10361897	Ifngr1	1	N/A
10361956	Mtap7	1	N/A
10362138	Vnn1	7	N/A
10362294	Arhgap18	1	N/A
10362389		19	N/A
10362420	Trdn	19	N/A
10362615	Traf3ip2	1	N/A
10362701	Ddo	19	N/A
10362899	F830002L21Rik	19	N/A
10362902	Gm9034	19	N/A
10362922	Atg5	1	N/A
10362937	Gm6983	19	N/A
10362941	Prep	13	N/A
10363137	Nus1	13	N/A
10363346	Ankrd57	19	N/A
10363379	Cbara1	7	N/A
10363392	Dnajb12	1	N/A
10363498	Ppa1	19	N/A
10363541	Ass1	19	N/A
10363563	Slc25a16	19	N/A
10363629	Pbld	7	N/A
10363639	Atoh7	19	N/A
10363894	Ipmk	19	N/A
10363905	Zwint	13	N/A
10364091		19	N/A
10364222	Ftcd	7	N/A
10364702	Midn	7	N/A
10364752	Dazap1	13	N/A
10364824	Csnk1g2	13	N/A
10364916	3110056O03Rik	1	N/A
10365227	Ap3m1	13	N/A
10365230	Tdg	7	N/A
10365420	AI597468	1	N/A
10365578	Nup37	7	N/A
10365845	Fgd6	7	N/A
10365887	Ndufa12	19	N/A
10365926		19	N/A
10365966	Gm5426	13	N/A
10365971	Btg1	1	N/A
10366038	Galnt4	19	N/A
10366301		13	N/A
10366337	Nap1l1	19	N/A
10366476	Ptprb	1	N/A
10366667	Gns	13	N/A
10366705	Gm9081	19	N/A
10366848	B4galnt1	7	N/A
10366866	Dctn2	7	N/A
10366909	R3hdm2	1	N/A
10367076	Prim1	19	N/A
10367100	Ptges3	19	N/A
10367292	Cs	1	N/A
10367305	Ankrd52	13	N/A
10367400	Mmp19	19	N/A
10367544	Ctdsp2	7	N/A
10367717	BC013529	13	N/A
10367744		19	N/A
10367822	Rab32	1	N/A
10367960	Pex3	7	N/A
10368083	Ccdc28a	1	N/A
10368229	Tbpl1	19	N/A
10368277	Rps12	19	N/A
10368356	Akap7	7	N/A
10368475		19	N/A
10368486	Rnf146	1	N/A
10368527	Hint3	1	N/A
10368647	Dse	1	N/A
10368670	Amd1	13	N/A
10368675	Marcks	1	N/A
10368730	Bxdc1	13	N/A
10368748	Amd1	13	N/A
10368845	2410017P07Rik	1	N/A
10368883	Tdg	7	N/A
10369221	Dux	19	N/A
10369264	Oit3	1	N/A
10369276	Ccdc109a	1	N/A

10369630	Ddx21	13	N/A
10369647	Ddx50	19	N/A
10369806	1700040L02Rik	13	N/A
10369929		19	N/A
10369948	Cabin1	7	N/A
10370013	Gstt2	7	N/A
10370259	Col18a1	7	N/A
10370303	Adarb1	13	N/A
10370422	Pwp2	13	N/A
10370552	Ppap2c	7	N/A
10370665	Med16	13	N/A
10370681	C030046I01Rik	13	N/A
10370824	Mbd3	19	N/A
10370892	Rexo1	7	N/A
10371111	Tjp3	1	N/A
10371159	Fzr1	7	N/A
10371230	Gna11	13	N/A
10371256	Sirt6	7	N/A
10371271	Zfp781	19	N/A
10371796	Slc17a8	19	N/A
10371959	Elk3	1	N/A
10372005	Vezt	7	N/A
10372060	Cradd	1	N/A
10372130	4930430F08Rik	19	N/A
10372324	Syt1	13	N/A
10372478	Rab21	1	N/A
10372733	Cdc5l	13	N/A
10372735	Cdc5l	13	N/A
10372737	Cdc5l	13	N/A
10372739	Cdc5l	13	N/A
10372741	Cdc5l	13	N/A
10372743	Cdc5l	13	N/A
10372807	Msrb3	1	N/A
10372877	Xpot	13	N/A
10373143	Mbd6	7	N/A
10373192	Inhbe	7	N/A
10373467	Erbb3	1	N/A
10373515	Suox	1	N/A
10373521	Rab5b	7	N/A
10373530	Cdk2	7	N/A
10373618	Olfr771	19	N/A
10373622	Olfr773	19	N/A
10373690		13	N/A
10374106	Ykt6	13	N/A
10374181	Gm11971	19	N/A
10374223	Igfbp1	1	N/A
10374415	Ppp3r1	13	N/A
10374466	Rab1	19	N/A
10374558		19	N/A
10375145	Lcp2	1	N/A
10375214		19	N/A
10375234	Nudcd2	13	N/A
10375290	Slu7	1	N/A
10375343	Rnf145	13	N/A
10375358	Ebf1	19	N/A
10375382	Clint1	1	N/A
10375483	Gm12176	19	N/A
10375499	Snord96a	13	N/A
10375811		1	N/A
10375893	Sar1b	7	N/A
10375973	Taf13	19	N/A
10375980	Aff4	7	N/A
10376033	Kif3a	19	N/A
10376142	3230401D17Rik	19	N/A
10376292		1	N/A
10376312	Larp1	1	N/A
10376326	Igtp	7	N/A
10376444	Hist3h2ba	19	N/A
10376534	Mprip	7	N/A
10376557	Nt5m	1	N/A
10376596	4933439F18Rik	19	N/A
10377364	Rnf222	1	N/A
10377380	1500010J02Rik	1	N/A
10377431	Vamp2	7	N/A
10377439	Per1	13	N/A
10377508	Trappc1	13	N/A
10377603	Tmem102	19	N/A
10377612	Plscr3	1	N/A
10377652	Gps2	1	N/A
10377695	Phf23	13	N/A
10377826	Zmynd15	1	N/A
10377847	Gltpd2	1	N/A
10377889	Mink1	1	N/A
10378059	Txndc17	13	N/A

10378068	Xaf1	7	N/A
10378082	Ggt6	19	N/A
10378271	1200014J11Rik	7	N/A
10378341	Shpk	13	N/A
10378427	Olfr401	19	N/A
10378453	1300001I01Rik	1	N/A
10378568		7	N/A
10378579	Prpf8	7	N/A
10378739	Ywhae	13	N/A
10378833	Ssh2	7	N/A
10378848	Hsp90aa1	19	N/A
10378988	Phf12	7	N/A
10379013	Flot2	13	N/A
10379153	Aldoc	13	N/A
10379260	Gm11201	19	N/A
10379344	Gm10387	13	N/A
10379467	Psmd11	1	N/A
10379560	Zfp830	13	N/A
10379564	Lig3	19	N/A
10379727	Gm11428	1	N/A
10379820	Acaca	19	N/A
10379901	Bcas3	7	N/A
10379998	Trim37	19	N/A
10380059		1	N/A
10380061		1	N/A
10380063		1	N/A
10380065		1	N/A
10380087	Mtmr4	1	N/A
10380210	Sfrs1	19	N/A
10380244	1700106J16Rik	19	N/A
10380260	Trim25	7	N/A
10380289	Mmd	7	N/A
10380318		19	N/A
10380384	Ankrd40	19	N/A
10380403	Lrrc59	13	N/A
10380417		1	N/A
10380524	Slc35b1	19	N/A
10380566	Phospho1	1	N/A
10380571	Gngt2	1	N/A
10380634	Hoxb7	19	N/A
10380761	Socs7	13	N/A
10380823	Lasp1	1	N/A
10380927	Grb7	1	N/A
10380986	Psmd3	7	N/A
10381063	Wipf2	1	N/A
10381096	Igfbp4	13	N/A
10381170	Hspb9	19	N/A
10381416	Rnd2	19	N/A
10381445	Tmem106a	7	N/A
10381474	Arl4d	1	N/A
10381528	Nags	19	N/A
10381567	Tmub2	7	N/A
10381683	Acbd4	19	N/A
10381697	Hexim1	19	N/A
10381807	Lin52	7	N/A
10382010	Dcaf7	7	N/A
10382115	Ccdc45	19	N/A
10382136	1810010H24Rik	7	N/A
10382228	Axin2	7	N/A
10382243	Gna13	1	N/A
10382300	Map2k6	13	N/A
10382321	Kcnj2	1	N/A
10382328	Sox9	19	N/A
10382345	Cog1	7	N/A
10382538	Armc7	19	N/A
10382573	2310067B10Rik	1	N/A
10382756	Unk	7	N/A
10382797	Gm7367	7	N/A
10382844	Snord1c	7	N/A
10382888	2810008D09Rik	13	N/A
10382912	Sept9	1	N/A
10383133	Slc26a11	7	N/A
10383152		7	N/A
10383168		7	N/A
10383198		7	N/A
10383200		7	N/A
10383202		7	N/A
10383204		7	N/A
10383206		7	N/A
10383210		7	N/A
10383212		7	N/A
10383214	Rnf213	1	N/A
10383360	Ccdc137	13	N/A
10383434	Notum	1	N/A

10383532	Narf	7	N/A
10383765		7	N/A
10383867	Mtmr3	1	N/A
10383993	Ccdc117	19	N/A
10384004	2010005J08Rik	7	N/A
10384123	Ddx56	13	N/A
10384138	Tmed4	13	N/A
10384152	Purb	7	N/A
10384322	Hus1	19	N/A
10384378	Ddc	19	N/A
10384396		19	N/A
10384423		13	N/A
10384497		1	N/A
10384522	Actr2	19	N/A
10384539	Slc1a4	1	N/A
10384672	Ahsa2	19	N/A
10384766		19	N/A
10384883	4931440F15Rik	19	N/A
10385004	Mare	13	N/A
10385022	Efcab9	1	N/A
10385081		19	N/A
10385486	Trim41	13	N/A
10385500	Irgm1	1	N/A
10385504	Gm5431	1	N/A
10385513	9930111J21Rik	1	N/A
10385518	Tgtp	1	N/A
10385533	Tgtp	1	N/A
10385572	Sqstm1	1	N/A
10385599	Canx	19	N/A
10385665	Zfp354b	19	N/A
10385686	Hnrnpab	19	N/A
10385699	Rmnd5b	1	N/A
10385761	Ube2b	7	N/A
10385774	Olfr1371	19	N/A
10385966	Anxa6	19	N/A
10386058	Sparc	1	N/A
10386093	Snord1c	7	N/A
10386236	Hist3h2bb	19	N/A
10386400	Arih2	7	N/A
10386402	Zkscan17	7	N/A
10386543	Pabpc4	13	N/A
10386548	Pabpc4	13	N/A
10386636	Usp22	13	N/A
10386775	Prpsap2	1	N/A
10386824	Akap10	7	N/A
10387372	Kdm6b	13	N/A
10387648	Tmem102	1	N/A
10387890	Cxcl16	1	N/A
10388010	C1qbp	13	N/A
10388031		7	N/A
10388194	Spns2	1	N/A
10388254	Aspa	19	N/A
10388304		7	N/A
10388440	Serpinf2	7	N/A
10388684	Taok1	7	N/A
10388734	Eral1	7	N/A
10388749	Traf4	7	N/A
10388834	Slc13a2	19	N/A
10388902	Lgals9	1	N/A
10389087	Rffl	7	N/A
10389114	Nle1	13	N/A
10389134	Slfn9	7	N/A
10389222	Ccl6	1	N/A
10389229		19	N/A
10389238	Dusp14	19	N/A
10389261	Gm11437	19	N/A
10389581	Ypel2	1	N/A
10389680	Msi2	1	N/A
10389701	Akap1	7	N/A
10389786	Hlf	13	N/A
10389795	Stxbp4	19	N/A
10390308	Sp2	1	N/A
10390352	Kpnb1	19	N/A
10390635	1810046J19Rik	1	N/A
10390653	Med24	1	N/A
10390768	Smarce1	1	N/A
10391146	Acly	19	N/A
10391207	Dhx58	7	N/A
10391301	Stat3	7	N/A
10391410	Cntd1	19	N/A
10391431	Aarsd1	19	N/A
10391572	Lsm12	19	N/A
10391625	Ubtf	7	N/A
10391768	Eftud2	19	N/A

10392033		19	N/A
10392087	Ccdc47	19	N/A
10392207	Tex2	19	N/A
10392251	Ddx5	7	N/A
10392284	Kpna2	13	N/A
10392300	Bptf	1	N/A
10392347	Pitpnc1	7	N/A
10392388	Prkca	7	N/A
10392437	Gm11696	1	N/A
10392464	Fam20a	1	N/A
10392735	Cdc42ep4	7	N/A
10392936	Nt5c	19	N/A
10392970	Mif4gd	1	N/A
10393058	H3f3b	1	N/A
10393309	Prpsap1	13	N/A
10393544	Cyth1	7	N/A
10393620	Cbx4	13	N/A
10393774	Nploc4	13	N/A
10393970	Fasn	19	N/A
10394082	1110031I02Rik	19	N/A
10394186	Dtnb	7	N/A
10394538	Acaca	13	N/A
10394560	Smc6	7	N/A
10394690	E2f6	19	N/A
10394699	Rock2	13	N/A
10394735	Pdia6	19	N/A
10394770	Odc1	19	N/A
10394800	Bnip3l	13	N/A
10394846		19	N/A
10394862	Ddef2	1	N/A
10394988		19	N/A
10395058	Adi1	7	N/A
10395064	Tssc1	1	N/A
10395103	Pxdn	1	N/A
10395158	6030469F06Rik	19	N/A
10395198	Gm9359	1	N/A
10395293	Atxn7l1	1	N/A
10395457	Etv1	19	N/A
10395466	Dock4	7	N/A
10395532		19	N/A
10395932	Ctage5	19	N/A
10395976	Dnajb6	13	N/A
10395978	Gm527	1	N/A
10396141		19	N/A
10396161	Tmx1	19	N/A
10396476	Rhoj	1	N/A
10396511	Syne2	7	N/A
10396610	Mthfd1	1	N/A
10396671	Plekhg3	7	N/A
10396730		19	N/A
10396936	Smoc1	7	N/A
10397002	Sipa1l1	1	N/A
10397068	Rbm25	19	N/A
10397081	Rbm25	19	N/A
10397083	Rbm25	19	N/A
10397249	Lin52	7	N/A
10397281	Ylpm1	7	N/A
10397541	Gm5039	19	N/A
10397627		19	N/A
10397645	Gpr65	1	N/A
10397717		19	N/A
10397853	Rin3	1	N/A
10397866	Golga5	7	N/A
10398069	Serpina3m	19	N/A
10398398		19	N/A
10398400		19	N/A
10398414		13	N/A
10398601	Rcor1	1	N/A
10398695	Mark3	7	N/A
10398795	Aspg	7	N/A
10398972	Mta1	13	N/A
10398996	Crip2	1	N/A
10399146		13	N/A
10399198	Ncoa4	13	N/A
10399228	Gm6742	19	N/A
10399379	Pgk1	19	N/A
10399419		1	N/A
10399465	Fam84a	1	N/A
10399605	Adam17	19	N/A
10399677	Cox7a2l	7	N/A
10399696	Rnf144a	1	N/A
10399710	Rsad2	1	N/A
10400095		13	N/A
10400304	Egln3	1	N/A

10400405	Nfkbia	13	N/A
10400605	Rpl10l	19	N/A
10400844	Pygl	19	N/A
10400883	Timm9	13	N/A
10400892	Gm7985	19	N/A
10400941	Dhrs7	19	N/A
10400975	Trmt5	19	N/A
10401007	Ppp2r5e	13	N/A
10401050	Gm7862	19	N/A
10401128	Max	13	N/A
10401138	Atp6v1d	13	N/A
10401181	Rdh11	19	N/A
10401192	Zfyve26	7	N/A
10401244	Actn1	13	N/A
10401365		1	N/A
10401382	Numb	7	N/A
10401420		19	N/A
10401667	0610007P14Rik	7	N/A
10401713	Pomt2	13	N/A
10401891	Ston2	1	N/A
10401900	Sel1l	1	N/A
10401939	Lysmd1	13	N/A
10402066	Foxn3	13	N/A
10402096	Ttc7b	13	N/A
10402266		7	N/A
10402283	Itpk1	1	N/A
10402302	Btbd7	7	N/A
10402336	Ddx24	7	N/A
10402390	Serpina1b	19	N/A
10402399	Serpina1a	19	N/A
10402406	Serpina1c	19	N/A
10402409	Serpina1e	19	N/A
10402519	Atg2b	7	N/A
10402604		1	N/A
10402615	Hsp90aa1	19	N/A
10402730	Ppp1r13b	7	N/A
10402841	Brf1	7	N/A
10403003	LOC638560	19	N/A
10403076		19	N/A
10403108	Hmgn2	13	N/A
10403110	Gm10421	19	N/A
10403273	Asb13	19	N/A
10403291	Akr1c14	19	N/A
10403312	Akr1c19	13	N/A
10403428	Larp5	13	N/A
10403455	Dip2c	7	N/A
10403462	Dip2c	7	N/A
10403584	Nid1	1	N/A
10403664	B3galnt2	19	N/A
10403934	Isca1	13	N/A
10404016	V1ri5	19	N/A
10404033	Hist1h1d	19	N/A
10404049	Hist1h3g	1	N/A
10404051	Hist1h4d	19	N/A
10404059		19	N/A
10404097	Slc17a3	19	N/A
10404218	Gpld1	13	N/A
10404376	Agtr1a	7	N/A
10404380	Dusp22	7	N/A
10404422	Serpinb6b	1	N/A
10404429	Serpinb9	7	N/A
10404496	Nqo2	1	N/A
10404606	Ly86	1	N/A
10404649	Dsp	7	N/A
10404702	Gcnt2	13	N/A
10404717	Pak1ip1	19	N/A
10404941	Aof1	19	N/A
10404965	Rnf144b	1	N/A
10404975	Id4	19	N/A
10405236	Sfxn1	19	N/A
10405263	4732471D19Rik	13	N/A
10405511	Ddx46	7	N/A
10405693	Dapk1	1	N/A
10405733	Gm7036	19	N/A
10405741	Gm3338	19	N/A
10405743		13	N/A
10405783		13	N/A
10405822	Ccrk	19	N/A
10405890		19	N/A
10405948	BC048507	19	N/A
10405969		19	N/A
10405971	Nsun2	19	N/A
10406108	5430425J12Rik	19	N/A
10406141	Brd9	7	N/A

10406245		7	N/A
10406364	2210408I21Rik	7	N/A
10406407	Arrdc3	7	N/A
10406423	Mblac2	19	N/A
10406530	Tmem167	19	N/A
10406564	Acot12	7	N/A
10406581	Dhfr	13	N/A
10406598	Serinc5	7	N/A
10406626	Homer1	19	N/A
10406757	Col4a3bp	7	N/A
10406795	Gfm2	19	N/A
10407097	Pde4d	7	N/A
10407211	Ppap2a	1	N/A
10407390	Ptbp1	7	N/A
10407511		19	N/A
10407533		19	N/A
10407543	Gtpbp4	13	N/A
10407707	Gm10336	13	N/A
10407766	Lgals8	1	N/A
10407792	Gpr137b-ps	19	N/A
10407892	Cdc2l5	7	N/A
10408081	Hist1h1b	19	N/A
10408123	V1rh2	19	N/A
10408212	Hist1h2be	19	N/A
10408227	Hfe	1	N/A
10408455	Cdkal1	19	N/A
10408610	Tubb2a	1	N/A
10408616	Slc22a23	7	N/A
10408689	Nrn1	1	N/A
10408709	Ssr1	19	N/A
10408755	Muted	19	N/A
10408762	Eef1e1	19	N/A
10408932	Gm8513	19	N/A
10409265	Auh	7	N/A
10409278	Nfil3	1	N/A
10409322	Thoc3	7	N/A
10409338	Nop16	19	N/A
10409345	Cltb	13	N/A
10409414	Rab24	13	N/A
10409526	BC021381	7	N/A
10409643	Hnrnpa0	7	N/A
10409645	Ubqln1	1	N/A
10409660	Gkap1	13	N/A
10409799	Isca1	19	N/A
10409804	Zcchc6	7	N/A
10409966		19	N/A
10409978	6720457D02Rik	1	N/A
10409990	6720489N17Rik	19	N/A
10410244	Zfp640	19	N/A
10410248	Zfp640	19	N/A
10410259	Uqcrb	19	N/A
10410341	Zfp87	19	N/A
10410393	Mtrr	13	N/A
10410435	Pols	13	N/A
10410452	Srd5a1	19	N/A
10410508	Ndufs6	19	N/A
10410709	Rfesd	1	N/A
10410877	Polr3g	19	N/A
10411119	Papd4	7	N/A
10411147	Bhmt2	19	N/A
10411156	Scamp1	7	N/A
10411223	S100z	19	N/A
10411332	Hmgcr	13	N/A
10411595	Naip2	1	N/A
10411776		13	N/A
10411782	Pik3r1	19	N/A
10411853	Erbb2ip	7	N/A
10411927	Sdccag10	7	N/A
10412060	Gm8985	19	N/A
10412260	Fst	1	N/A
10412380		1	N/A
10412421	Zfp131	13	N/A
10412562	Flnb	13	N/A
10412624	Pxk	7	N/A
10412711	Uqcrb	19	N/A
10412741	Atxn7	7	N/A
10412919		19	N/A
10412943	Nudt13	1	N/A
10413059	Vcl	1	N/A
10413138	Vdac2	13	N/A
10413171		1	N/A
10413220	Gm10397	1	N/A
10413282	Fam116a	19	N/A
10413461	Erc2	19	N/A

10413530	Dcp1a	7	N/A
10413574	Sfmbt1	13	N/A
10413803	Btd	1	N/A
10413826	Oxnad1	1	N/A
10413839	Ncoa4	13	N/A
10413932	E130203B14Rik	1	N/A
10413977	Gm626	19	N/A
10414163	Lrit1	7	N/A
10414192	Mat1a	13	N/A
10414228	1700024B05Rik	19	N/A
10414234		19	N/A
10414250		19	N/A
10414475	Olfr738	19	N/A
10414514	Pnp1	1	N/A
10414527	Pnp1	1	N/A
10414537	Ang	13	N/A
10414661	Gm8586	19	N/A
10414790		19	N/A
10414900		19	N/A
10415021	Abhd4	1	N/A
10415030		7	N/A
10415065	Lrp10	7	N/A
10415282	Psme1	13	N/A
10415293	Rnf31	1	N/A
10415415	9130227C08Rik	1	N/A
10415444		19	N/A
10415513	Parp4	13	N/A
10415649	Zdhhc20	13	N/A
10415723		19	N/A
10415885	Sox7	19	N/A
10415888	Gm10867	19	N/A
10416044	Ccdc25	1	N/A
10416107	Gm10032	13	N/A
10416266	Ppp3cc	19	N/A
10416290	Reep4	1	N/A
10416332	B930095I24Rik	19	N/A
10416371	Lpar6	19	N/A
10416588	1300010F03Rik	7	N/A
10416705	Gm9264	19	N/A
10417000		19	N/A
10417013	Dnajc3	19	N/A
10417034	Dnajc3	19	N/A
10417053	Mbnl2	13	N/A
10417065	Rap2a	19	N/A
10417070		13	N/A
10417526	Dnase1l3	1	N/A
10417538		13	N/A
10417601	Ptma	13	N/A
10417617		19	N/A
10417676	Thoc7	19	N/A
10417689	Psmd6	1	N/A
10417759	Ube2e2	7	N/A
10417798	Kcnk5	13	N/A
10417950	Ndst2	7	N/A
10418004	Ap3m1	13	N/A
10418171	Zcchc24	13	N/A
10418185	D14Ertd449e	19	N/A
10418198	D14Ertd449e	19	N/A
10418210	D14Ertd449e	19	N/A
10418410	Prkcd	7	N/A
10418455	Itih1	1	N/A
10418480	Gnl3	13	N/A
10418720	Mettl6	7	N/A
10418747	Hacl1	19	N/A
10418905	Fam35a	13	N/A
10419073	Tspan14	1	N/A
10419136		13	N/A
10419198	Ero1l	13	N/A
10419261	Bmp4	1	N/A
10419542	Apex1	7	N/A
10419607		19	N/A
10419691	Tox4	1	N/A
10419759	Prmt5	19	N/A
10419803	4931414P19Rik	1	N/A
10419872		19	N/A
10419966	Zfhx2	1	N/A
10420165	Cideb	7	N/A
10420348	Zmym5	7	N/A
10420362	Gjb2	7	N/A
10420372		13	N/A
10420413	Lats2	13	N/A
10420516		19	N/A
10420591		19	N/A
10420631	Ebpl	1	N/A

10420659	6330409N04Rik	1	N/A
10420823	Hmbox1	1	N/A
10421003	Bnip3l	13	N/A
10421046	Dock5	7	N/A
10421172	Slc25a37	13	N/A
10421227	2610301G19Rik	7	N/A
10421269	Sorbs3	7	N/A
10421309	Slc39a14	13	N/A
10421577	Gm9779	19	N/A
10421817	Narg1l	13	N/A
10421906		19	N/A
10421908	Rps3a	19	N/A
10421917	Pabpc4	13	N/A
10422013	Klf12	1	N/A
10422164	Ednrb	19	N/A
10422396	Stk24	7	N/A
10422518	Tmtc4	19	N/A
10422598	Sepp1	19	N/A
10422728	Dab2	1	N/A
10423005	Ugt3a1	1	N/A
10423090	Amacr	7	N/A
10423271	Cdh18	19	N/A
10423333	Fam134b	7	N/A
10423363	Ank	13	N/A
10423568	5730407I07Rik	1	N/A
10423805	Atp6v1c1	13	N/A
10423941	Ttc35	13	N/A
10423963	Eny2	13	N/A
10424097	Med30	7	N/A
10424105	Colec10	1	N/A
10424140	Col14a1	1	N/A
10424213	Zhx2	1	N/A
10424250	Snrpd2	19	N/A
10424260	D15Ertd621e	7	N/A
10424363	Nsmce2	7	N/A
10424370	Trib1	13	N/A
10424557	Gm7125	19	N/A
10424577	Chrac1	13	N/A
10424674		19	N/A
10424676	Ly6e	1	N/A
10424695	Gpihbp1	1	N/A
10424705	2810039B14Rik	19	N/A
10424756	Mapk15	1	N/A
10424781	Grina	13	N/A
10424825	Cyc1	19	N/A
10425018	Rpl8	19	N/A
10425046	LOC626711	19	N/A
10425078	Mpst	1	N/A
10425116	Cdc42ep1	13	N/A
10425158	Pdxp	19	N/A
10425333	Apobec3	1	N/A
10425430	Tnrc6b	7	N/A
10425527	Ep300	1	N/A
10425611	Aco2	13	N/A
10425653	4930407I10Rik	19	N/A
10425905	Nup50	7	N/A
10426157	Trabd	13	N/A
10426301		7	N/A
10426451	Irak4	19	N/A
10426479	Ano6	1	N/A
10426550	Tmem106c	13	N/A
10426798	Smarcd1	1	N/A
10427015	Acvr1b	7	N/A
10427045	9430023L20Rik	19	N/A
10427049	6030408B16Rik	13	N/A
10427083	Eif4b	7	N/A
10427131	Soat2	7	N/A
10427162	Mfsd5	19	N/A
10427214	Sp1	1	N/A
10427241	Pcbp2	7	N/A
10427290	Hoxc8	19	N/A
10427334		19	N/A
10427665		19	N/A
10427742	Gm8174	19	N/A
10427881	Gm8341	19	N/A
10427883		19	N/A
10427895	Basp1	19	N/A
10427997	Ankrd33b	19	N/A
10428068	Tspyl5	19	N/A
10428171	Ankrd46	19	N/A
10428238	Ubr5	13	N/A
10428302	Klf10	13	N/A
10428328	Slc25a32	13	N/A
10428336	Ftl2	19	N/A

10428374	Gm5470	19	N/A
10428561	Rad21	7	N/A
10428698	Sntb1	1	N/A
10428736	Derl1	1	N/A
10428839	Gm5959	19	N/A
10428857	Mtss1	13	N/A
10428877	E430025E21Rik	7	N/A
10428918	D330050I23Rik	19	N/A
10429140	Ndrg1	1	N/A
10429216	Fam135b	19	N/A
10429298	Trappc9	1	N/A
10429674	Tsta3	13	N/A
10429772	Eppk1	7	N/A
10429843	Parp10	7	N/A
10429972	Cpsf1	1	N/A
10430105	C030006K11Rik	13	N/A
10430510	Tmem184b	7	N/A
10430596	Unc84b	7	N/A
10430669		19	N/A
10430723		19	N/A
10430739	Dnajb7	13	N/A
10430770	Tob2	13	N/A
10430834	Naga	13	N/A
10430871	Tdg	7	N/A
10430993	1700001L05Rik	13	N/A
10430997	Pacsin2	7	N/A
10431424	Plxnb2	1	N/A
10431486	Sbf1	7	N/A
10431528	Lmf2	1	N/A
10431546	Ncaph2	1	N/A
10431585	Chkb	7	N/A
10431612	Rabl2a	1	N/A
10431697	Abcd2	7	N/A
10431894	Slc38a2	7	N/A
10432101	Senp1	7	N/A
10432236	Rnd1	1	N/A
10432256	Arf3	13	N/A
10432636	BC004728	1	N/A
10433032	Atf7	7	N/A
10433034	Atf7	7	N/A
10433047		19	N/A
10433088	Cbx5	19	N/A
10433101	Gpr84	1	N/A
10433161	Marcksl1	19	N/A
10433199	Zfp263	13	N/A
10433274	Vasn	13	N/A
10433292	Hmox2	13	N/A
10433445	Abat	1	N/A
10433462	Pmm2	13	N/A
10433472	1810013L24Rik	7	N/A
10433478		19	N/A
10433639	Mkl2	1	N/A
10433672	Rrn3	13	N/A
10433717		19	N/A
10433776	Snai2	7	N/A
10433792		19	N/A
10434029	Lztr1	7	N/A
10434384	Ap2m1	13	N/A
10434629	Map3k13	19	N/A
10434675	Dnajb11	19	N/A
10434689	Ahsg	19	N/A
10434733	Eif4a2	1	N/A
10434778	Rtp4	1	N/A
10434782	Lpp	1	N/A
10434869	Ccdc50	7	N/A
10434934	Bdh1	7	N/A
10434942	Dlg1	7	N/A
10435024	Rnf168	19	N/A
10435057	Pcyt1a	19	N/A
10435305	Itgb5	19	N/A
10435543	Golgb1	7	N/A
10435617	Rabl3	19	N/A
10435626	Hgd	1	N/A
10435654	Lrrc58	13	N/A
10435676	Gsk3b	7	N/A
10435733	Igsf11	1	N/A
10435769	Zbtb20	13	N/A
10435787		7	N/A
10435821	Nat13	19	N/A
10435897	BC027231	13	N/A
10436037		19	N/A
10436239	2310061J03Rik	13	N/A
10436251		19	N/A
10436348	Tomm70a	13	N/A

10436500	Gbe1	13	N/A
10436550		19	N/A
10436608	Cxadr	19	N/A
10436666	Jam2	1	N/A
10436708	Usp16	7	N/A
10436800	1110008E08Rik	19	N/A
10436945	Slc5a3	19	N/A
10436983	Dopey2	19	N/A
10437023	Morc3	13	N/A
10437136	Dyrk1a	7	N/A
10437160	Ets2	13	N/A
10437239		19	N/A
10437332	Crebbp	1	N/A
10437364	Adcy9	7	N/A
10437499	Glyr1	7	N/A
10437582		19	N/A
10437639	Emp2	7	N/A
10437687		1	N/A
10437748	Gspt1	13	N/A
10437852	4921513D23Rik	19	N/A
10437963	Fam128b	7	N/A
10438017	Fgd4	19	N/A
10438071	Ppil2	1	N/A
10438198	Med15	7	N/A
10438328	D16H22S680E	13	N/A
10438423	Olfr165	19	N/A
10438460	Parl	19	N/A
10438527	Camk2n2	19	N/A
10438530	Clcn2	13	N/A
10438572	2510009E07Rik	19	N/A
10438575	Ehhadh	1	N/A
10438621	Tra2b	19	N/A
10438813		19	N/A
10439049	Pigx	13	N/A
10439130	Umps	19	N/A
10439239	Dirc2	1	N/A
10439249	Parp14	7	N/A
10439268	Dtx3l	7	N/A
10439404	Gm4324	1	N/A
10439409	BC031361	19	N/A
10439455	Adprh	1	N/A
10439498		7	N/A
10439532	Qtrtd1	13	N/A
10439701	Abhd10	7	N/A
10439895	Alcam	7	N/A
10439960	Cep97	19	N/A
10440158	Olfr191	19	N/A
10440178	Olfr204	19	N/A
10440186	Crybg3	19	N/A
10440246	Arl13b	1	N/A
10440284		13	N/A
10440300		19	N/A
10440406	Nrip1	1	N/A
10440427	D16Ertd472e	19	N/A
10440522	Adamts1	1	N/A
10440717	Krtap6-1	19	N/A
10440929	Gart	19	N/A
10441064	Dscr3	1	N/A
10441107	Psmg1	19	N/A
10441400	Arid1b	1	N/A
10441436	Snx9	13	N/A
10441509	Ppp1r2	13	N/A
10441797	Tcp1	19	N/A
10441864	Mllt4	7	N/A
10442037	Zfp97	1	N/A
10442057	Riok2	19	N/A
10442155	Ppp2r1a	1	N/A
10442219	Zfp52	19	N/A
10442493		1	N/A
10442549	Zfp598	13	N/A
10442596	Sepx1	13	N/A
10442606	4930528F23Rik	7	N/A
10442739	Unkl	1	N/A
10442752	0610007P22Rik	19	N/A
10442904	Jmjd8	19	N/A
10443007	EG240055	1	N/A
10443391	Mapk14	13	N/A
10443421	Brpf3	13	N/A
10443463	Cdkn1a	1	N/A
10443482	BC004004	1	N/A
10443527	Pim1	7	N/A
10443550	Rnf8	7	N/A
10443561	1300018I05Rik	1	N/A
10443589	Zfand3	7	N/A

10443596	Gm9874	1	N/A
10443898	Cyp4f15	1	N/A
10443918	C920016K16Rik	19	N/A
10444008	Zfp414	1	N/A
10444524		7	N/A
10444656		19	N/A
10444761	Bat1a	1	N/A
10444778		7	N/A
10444895	Flot1	13	N/A
10444911	Mdc1	1	N/A
10445033	Trim26	7	N/A
10445139	Olfr110	19	N/A
10445338	Enpp5	7	N/A
10445458	Xpo5	7	N/A
10445531	Zfp318	7	N/A
10445558	BC048355	19	N/A
10445674	Mrfap1	19	N/A
10445774	B430306N03Rik	7	N/A
10445796	AI314976	7	N/A
10445896		19	N/A
10445909	Kat2b	13	N/A
10445941	Gm4471	19	N/A
10445953	Emr4	1	N/A
10446166	Ndufa11	19	N/A
10446207	Clpp	19	N/A
10446235	Trip10	19	N/A
10446282	Emr1	1	N/A
10446334	Glcci1	7	N/A
10446376	Man2a1	13	N/A
10446402	Ppp4r1	13	N/A
10446581	Zfp161	19	N/A
10446656	Lpin2	13	N/A
10446693	Wdr43	13	N/A
10446713	Snord53	7	N/A
10446756	Ypel5	7	N/A
10446771	Lclat1	7	N/A
10446777	Ehd3	1	N/A
10446965	Rasgrp3	1	N/A
10447025	Ccdc75	19	N/A
10447128	Gm6594	19	N/A
10447239	Abcg8	7	N/A
10447341	Rhoq	7	N/A
10447354	Txndc14	19	N/A
10447417	Msh6	1	N/A
10447437	Klraq1	1	N/A
10447483	Nanp	7	N/A
10447486		19	N/A
10447904	Unc93a	7	N/A
10448004	Phf10	13	N/A
10448032	Gm10510	19	N/A
10448192	Gm6540	19	N/A
10448202	Tpm4	19	N/A
10448262	Zfp213	13	N/A
10448307	Tnfrsf12a	13	N/A
10448455	Atp6v0c	13	N/A
10448459	Tbc1d24	13	N/A
10448495	1600002H07Rik	13	N/A
10448700	Gfer	1	N/A
10448743	Fahd1	19	N/A
10448748	Nubp2	19	N/A
10448811	Cramp1l	7	N/A
10449471	Srpk1	13	N/A
10449644	Glo1	1	N/A
10449712	Cbs	1	N/A
10449815	Brd4	7	N/A
10449935	Zfp870	19	N/A
10449989	Zfp422	1	N/A
10449991	Zfp81	1	N/A
10450126	Brd2	13	N/A
10450296	Skiv2l	7	N/A
10450325	Cfb	19	N/A
10450435	Csnk2b	13	N/A
10450675	H2-T24	1	N/A
10450752	Trim39	19	N/A
10450923	Pgk2	19	N/A
10451004	Cd2ap	1	N/A
10451123	Slc29a1	1	N/A
10451395	Klc4	13	N/A
10451495	Ubr2	13	N/A
10451547	Gm16494	19	N/A
10451818	Sult1c2	13	N/A
10451879	Zfp119	19	N/A
10451932	Plin4	13	N/A
10451943	Plin5	13	N/A

10452000	Dpp9	1	N/A
10452030	Plin3	7	N/A
10452228	Khsrp	1	N/A
10452430	Fbxl17	7	N/A
10452442	AU016765	1	N/A
10452516	Ankrd12	13	N/A
10452815	Xdh	19	N/A
10452892	Fam98a	13	N/A
10452980	Eif2ak2	7	N/A
10453062	Atl2	13	N/A
10453139	Gm10190	19	N/A
10453178	Map4k3	7	N/A
10453260	Haao	1	N/A
10453318	Abcg5	7	N/A
10453334	Lrpprc	13	N/A
10453399	Srbd1	19	N/A
10453426	Atp6v1e2	19	N/A
10453429	Rhoq	13	N/A
10453512		13	N/A
10453573	Olfr63	19	N/A
10453575	Cul2	1	N/A
10453604	Bambi	19	N/A
10453738	Fzd8	7	N/A
10453918	3110002H16Rik	7	N/A
10454039	Impact	19	N/A
10454077	Taf4b	7	N/A
10454113	Dsg1a	13	N/A
10454192	Ttr	19	N/A
10454198	Rnf125	1	N/A
10454202	Rnf138	7	N/A
10454353	Mocos	1	N/A
10454514	Lims2	7	N/A
10454580	Bin1	19	N/A
10454606	Wdr36	19	N/A
10454851	Cxxc5	1	N/A
10454877	Pura	1	N/A
10454944	Eif4ebp3	13	N/A
10454953	Tmco6	7	N/A
10454966	Ik	1	N/A
10455069	Pcdhb6	13	N/A
10455259	Arhgap26	1	N/A
10455472	Dcp2	7	N/A
10455738	Snx2	7	N/A
10455752	Snx24	1	N/A
10455769	Csnk1g3	13	N/A
10455784	Gramd3	1	N/A
10455873	Slc12a2	19	N/A
10455948	Chsy3	1	N/A
10455974	Dctn4	1	N/A
10455989	Rbm22	7	N/A
10456046	Pdgfrb	1	N/A
10456071	Csf1r	1	N/A
10456361	Gm9926	19	N/A
10456492	D18Ertd653e	7	N/A
10456522	Tcf4	1	N/A
10456745	Smad7	13	N/A
10456793	Ier3ip1	19	N/A
10456970		1	N/A
10456974	Arf1	13	N/A
10457114	Bhmt	19	N/A
10457205	Crem	1	N/A
10457380		19	N/A
10457637		19	N/A
10457644	Cdh2	7	N/A
10457888	5730494M16Rik	19	N/A
10457938		19	N/A
10457948	Slc25a46	7	N/A
10458138	Brd8	7	N/A
10458213	Etf1	13	N/A
10458355	Apbb3	7	N/A
10458398	Hars	13	N/A
10458461	Hdac3	7	N/A
10458534	Pcdh1	7	N/A
10458547	Gnpda1	1	N/A
10458583	Yipf5	19	N/A
10458680	Eif3j	19	N/A
10458755	Ythdc2	1	N/A
10458882	LOC674866	19	N/A
10458956		19	N/A
10458960	Aldh7a1	19	N/A
10458992	C330018D20Rik	19	N/A
10459158	Hmgxb3	13	N/A
10459193	Ppargc1b	7	N/A
10459389		13	N/A

10459510		7	N/A
10459534	Afg3l2	13	N/A
10459604	4933403F05Rik	19	N/A
10459705	Smad4	1	N/A
10459799	1700003O11Rik	19	N/A
10459802	Gm9028	19	N/A
10459930	Ctdp1	7	N/A
10460196	1810055G02Rik	13	N/A
10460255	1700055N04Rik	19	N/A
10460259	Aldh3b2	19	N/A
10460263	Acy3	7	N/A
10460400	Pcx	7	N/A
10460582	AI837181	1	N/A
10460787	Atg2a	7	N/A
10461164	Wdr74	13	N/A
10461257	Ubxn1	7	N/A
10461305	B3gat3	1	N/A
10461423	Fads3	19	N/A
10461475	Cpsf7	7	N/A
10461526	Vwce	7	N/A
10461587	Ms4a4a	7	N/A
10461663	Mrpl16	1	N/A
10461728	Gm4952	7	N/A
10461909	BC016495	13	N/A
10461991	Zfand5	7	N/A
10461999	Fam108b	13	N/A
10462130	Fam122a	19	N/A
10462136	Cycs	19	N/A
10462214		1	N/A
10462237	Smarca2	13	N/A
10462621	I830012O16Rik	7	N/A
10462630	Pank1	7	N/A
10462822		7	N/A
10462918	Tmem20	13	N/A
10462922	Plce1	13	N/A
10462973	Hells	19	N/A
10463061	Gm5827	13	N/A
10463068		13	N/A
10463140	Lcor	19	N/A
10463211	Pi4k2a	1	N/A
10463308	Abcc2	7	N/A
10463343	Gm10768	13	N/A
10463355	Scd2	19	N/A
10463448	Peo1	13	N/A
10463486	Btrc	7	N/A
10463517	Pprc1	13	N/A
10463661	Trim8	13	N/A
10463739	Taf5	7	N/A
10463751	Pdcd11	13	N/A
10464045	Acsl5	19	N/A
10464218		7	N/A
10464240	Trub1	19	N/A
10464445	Gm5521	19	N/A
10464504	Lrp5	1	N/A
10464586	Gstp1	7	N/A
10464601		1	N/A
10464659	Rad9	1	N/A
10464672	Ssh3	7	N/A
10464728	Kdm2a	7	N/A
10464772	2010003K11Rik	13	N/A
10464819	Rbm14	13	N/A
10465054	Ccdc85b	19	N/A
10465150	Pcnxl3	7	N/A
10465226	Scyl1	13	N/A
10465374	Sac3d1	19	N/A
10465604	Stip1	19	N/A
10465638	Nat11	7	N/A
10465651	Mark2	7	N/A
10465683	Rab11b	7	N/A
10465895	Fads2	13	N/A
10465916	Gm98	1	N/A
10466008	Dak	13	N/A
10466104	Ccdc86	13	N/A
10466353		1	N/A
10466441	Vps13a	19	N/A
10466676	1110059E24Rik	13	N/A
10466935	Rln1	19	N/A
10466963	9930021J03Rik	1	N/A
10467003	Gm5972	13	N/A
10467041	Asah2	13	N/A
10467068	Sgms1	7	N/A
10467088		19	N/A
10467102	Rnls	1	N/A
10467216	Cpeb3	7	N/A

10467319	Rbp4	19	N/A
10467410	Cyp2c70	19	N/A
10467420		1	N/A
10467489	Ptp4a1	13	N/A
10467578	Pik3ap1	7	N/A
10467766	Loxl4	1	N/A
10467768	Loxl4	1	N/A
10467956	Bloc1s2	1	N/A
10467979	Scd1	19	N/A
10468081	Npm3	7	N/A
10468159	Ldb1	13	N/A
10468231	Arl3	1	N/A
10468292	Pdcd11	7	N/A
10468309	2310014D11Rik	1	N/A
10468533		19	N/A
10468691	Ablim1	1	N/A
10468722	Gfra1	13	N/A
10468795	Rab11fip2	13	N/A
10469046	Phyh	19	N/A
10469070	Nudt5	13	N/A
10469145	Kin	7	N/A
10469199		19	N/A
10469203		19	N/A
10469207		19	N/A
10469213		19	N/A
10469217		19	N/A
10469221		19	N/A
10469225		19	N/A
10469227		19	N/A
10469231		19	N/A
10469289	Il15ra	7	N/A
10469358	Mrc1	1	N/A
10469575		13	N/A
10469609	Gm13375	13	N/A
10469816	Il1rn	1	N/A
10469923	Entpd8	7	N/A
10469941		7	N/A
10470050	Abca2	1	N/A
10470268	Pmpca	13	N/A
10470446	Rxra	7	N/A
10470584	Tsc1	7	N/A
10470628	Ddx31	13	N/A
10470649	Ttf1	19	N/A
10470816	Gle1	7	N/A
10471045	Ppp2r4	1	N/A
10471108	Gpr107	7	N/A
10471154	Ass1	19	N/A
10471191	Exosc2	19	N/A
10471298	Bat2l	7	N/A
10471337	Pomt1	1	N/A
10471411	Ptges2	13	N/A
10471424	Fam102a	13	N/A
10471486	Eng	1	N/A
10471599	D730039F16Rik	19	N/A
10471675	Glo1	1	N/A
10471844	Nek6	1	N/A
10472095	Bloc1s2	1	N/A
10472199	Upp2	13	N/A
10472366	Scn2a1	1	N/A
10472501	Lass6	13	N/A
10472514	Nostrin	1	N/A
10472570	Ppig	1	N/A
10472846	Pdk1	19	N/A
10472893	B230120H23Rik	7	N/A
10472923	Ak3l1	13	N/A
10472933	Scrn3	19	N/A
10473008	Hnrnpa3	19	N/A
10473097	Plekha3	13	N/A
10473118	Ube2e3	7	N/A
10473224	Dusp19	1	N/A
10473356	Ube2l6	1	N/A
10473363	Timm10	13	N/A
10473367	Slc43a1	13	N/A
10473454	Olfr1008	19	N/A
10473483	Olfr1029	13	N/A
10473515		19	N/A
10473624	Olfr1257	1	N/A
10473760	Cugbp1	7	N/A
10473847	Acp2	1	N/A
10474028		7	N/A
10474064	Trp53i11	1	N/A
10474102		19	N/A
10474141	Slc1a2	13	N/A
10474181	Abtb2	1	N/A

10474199		19	N/A
10474201	Lmo2	1	N/A
10474411	Lin7c	13	N/A
10475199	Snap23	1	N/A
10475211	Haus2	7	N/A
10475226		19	N/A
10475247	Tmem62	1	N/A
10475264	Ccndbp1	7	N/A
10475293	Tubgcp4	1	N/A
10475350	Serf2	19	N/A
10475360	Mrpl51	13	N/A
10475362	Wdr76	19	N/A
10475437	Sord	19	N/A
10475623	Fbn1	19	N/A
10475718	Snrnp200	7	N/A
10475800	Adra2b	1	N/A
10475866	Bcl2l11	7	N/A
10475941	Zc3h6	7	N/A
10475957	Ttl	13	N/A
10475965	Polr1b	13	N/A
10475990	Slc20a1	7	N/A
10476093	Nop56	13	N/A
10476207	Atrn	7	N/A
10476319		19	N/A
10476668	Csrp2bp	19	N/A
10476759	Rin2	1	N/A
10476928	Gm14131	19	N/A
10476939	Gm4979	1	N/A
10477012	Fkbp1a	1	N/A
10477061	Srxn1	7	N/A
10477090	Tbc1d20	7	N/A
10477169	Id1	13	N/A
10477311	Asxl1	7	N/A
10477370		13	N/A
10477604	Itch	13	N/A
10477852	Gm14251	19	N/A
10477946	Rpn2	19	N/A
10477986	Nnat	1	N/A
10478196	Top1	13	N/A
10478389	Hnf4a	13	N/A
10478421	Kcnk15	19	N/A
10478748	Trp53rk	7	N/A
10478854	Slc9a8	7	N/A
10478890	Cebpb	13	N/A
10478928	Tshz2	7	N/A
10478936		19	N/A
10478962	2010011I20Rik	1	N/A
10479077	Vapb	7	N/A
10479099	Npepl1	7	N/A
10479154	Tubb1	1	N/A
10479176	Gm14391	19	N/A
10479203	Gm14434	19	N/A
10479217	Gm14434	13	N/A
10479221		13	N/A
10479228	Etohi1	19	N/A
10479297	Lsm14b	7	N/A
10479335	Osbpl2	13	N/A
10479411	Ogfr	13	N/A
10479458	2310003C23Rik	13	N/A
10479490	Arfgap1	7	N/A
10479639	Dnajc5	13	N/A
10479736	Polr3k	19	N/A
10479749	Rpp38	13	N/A
10479775	Hspa14	19	N/A
10479794	Prpf18	7	N/A
10479833	Optn	7	N/A
10480003	Itih2	7	N/A
10480035	Pfkfb3	13	N/A
10480347	Pip4k2a	1	N/A
10480381	Arhgap21	13	N/A
10480423	Abi1	7	N/A
10480459	Hnmt	13	N/A
10480601	A830007P12Rik	19	N/A
10480628		1	N/A
10481011	Pmpca	1	N/A
10481128	Med22	13	N/A
10481182	Fam163b	19	N/A
10481186	Sardh	19	N/A
10481210	Vav2	7	N/A
10481378		19	N/A
10481393	Zdhhc12	7	N/A
10481540	Fnbp1	7	N/A
10481577	Uck1	19	N/A
10481654	Fpgs	1	N/A

10481804	Ralgps1	19	N/A
10481868	Dnajb6	13	N/A
10481909	Fbxw2	7	N/A
10482004	AI182371	1	N/A
10482030	Stom	1	N/A
10482144	Rc3h2	7	N/A
10482200	Dennd1a	7	N/A
10482528	Neb	7	N/A
10482712	Stam2	7	N/A
10482793		7	N/A
10483023	Rbms1	7	N/A
10483025	Rbms1	7	N/A
10483110	Ifih1	7	N/A
10483178	Cobll1	7	N/A
10483559	Gm16510	19	N/A
10483679		19	N/A
10483768		19	N/A
10484249	Cwc22	1	N/A
10484256	4930401B11Rik	19	N/A
10484371	Calcrl	1	N/A
10484431	Txndc14	19	N/A
10484457	Clp1	19	N/A
10484541	Olfr154	13	N/A
10484586	Olfr1054	7	N/A
10484616	Olfr1082	19	N/A
10484640	Olfr1099	7	N/A
10484642	Olfr1100	19	N/A
10484658	Olfr1109	19	N/A
10484685	Olfr1135	13	N/A
10484888	Ptprj	1	N/A
10484894	Ptprj	1	N/A
10485042	Zfp408	7	N/A
10485170	Cry2	7	N/A
10485225	Ext2	1	N/A
10485277		19	N/A
10485282	Alkbh3	1	N/A
10485340	Itpa	1	N/A
10485344	Api5	19	N/A
10485562	Hipk3	7	N/A
10485582	Tcp11l1	1	N/A
10485607	Qser1	7	N/A
10485622	Qser1	7	N/A
10485635	Eif3m	13	N/A
10485811	Olfr1313	19	N/A
10486057	Rab5b	1	N/A
10486172	Fam82a2	19	N/A
10486201	Gm14207	1	N/A
10486396	Ehd4	19	N/A
10486710	Lcmt2	1	N/A
10487154	Secisbp2l	13	N/A
10487175	Cops2	1	N/A
10487433	Zfp661	19	N/A
10487748	4930402H24Rik	1	N/A
10487823		1	N/A
10487890	Erv3	13	N/A
10487906	Slc23a2	7	N/A
10488010	Hao1	19	N/A
10488048	Mkks	19	N/A
10488382	Cd93	1	N/A
10488441	Zfp120	1	N/A
10488550	Nanp	7	N/A
10488575	Psmf1	7	N/A
10488630	Defb23	19	N/A
10488722	Commd7	1	N/A
10488844	Ncoa6	1	N/A
10488879	Gss	7	N/A
10489041	Pdcd10	7	N/A
10489049		19	N/A
10489107	Samhd1	1	N/A
10489364	3230401D17Rik	1	N/A
10489368	Fitm2	7	N/A
10489620	Ncoa5	1	N/A
10489946	Dpm1	19	N/A
10490246	3100002L24Rik	19	N/A
10490250	3100002L24Rik	19	N/A
10490276	2210418O10Rik	19	N/A
10490294	Gm14434	19	N/A
10490299		19	N/A
10490352	Taf4a	1	N/A
10490467	Cables2	13	N/A
10490559	Chrna4	19	N/A
10490632	BC006779	1	N/A
10490838	Fabp5	1	N/A
10490946	Hsp90aa1	19	N/A

10490955	Mtfr1	7	N/A
10490970		19	N/A
10491038	Tbl1xr1	7	N/A
10491171	Slc2a2	13	N/A
10491300	Skil	19	N/A
10491455	Fxr1	7	N/A
10491595	4932438A13Rik	1	N/A
10491780	Hspa4l	19	N/A
10491820	3110057O12Rik	1	N/A
10491915	Ccrn4l	13	N/A
10491962	Foxo1	13	N/A
10492045	Hnrnpa3	19	N/A
10492091	Smad9	7	N/A
10492195	Tsc22d2	7	N/A
10492341	4631416L12Rik	13	N/A
10492668	4930589L23Rik	19	N/A
10492671	Ppid	19	N/A
10492735		1	N/A
10492755	Fga	1	N/A
10492860	Higd1a	19	N/A
10492864	Sh3d19	7	N/A
10492997	Etv3	19	N/A
10493009	Arhgef11	1	N/A
10493103	Isg20l2	13	N/A
10493203	0610031J06Rik	13	N/A
10493210	Smg5	13	N/A
10493259	Ssr2	19	N/A
10493309	Rit1	1	N/A
10493335		1	N/A
10493345		1	N/A
10493494	Efna3	1	N/A
10493565	Adar	7	N/A
10493585	Ube2q1	7	N/A
10493666	Nup210l	7	N/A
10493711	Crtc2	7	N/A
10493758	Gatad2b	1	N/A
10493984	Tchh	19	N/A
10494003	Tdpoz3	19	N/A
10494114	Selenbp1	19	N/A
10494137	Pi4kb	7	N/A
10494208		7	N/A
10494271	Ctss	1	N/A
10494322	Anp32e	19	N/A
10494351	Mtmr11	19	N/A
10494386	Hist1h2an	19	N/A
10494390	Hist2h2aa1	1	N/A
10494395	Hist2h2aa1	1	N/A
10494423	Hfe2	1	N/A
10494452	Rbm8a	19	N/A
10494551	Acp6	7	N/A
10494565	Fmo5	13	N/A
10494595	Notch2	1	N/A
10494664	Wars2	1	N/A
10495094	6530418L21Rik	1	N/A
10495134	Gm4248	19	N/A
10495285	Sort1	7	N/A
10495549	Dbt	19	N/A
10495651	Alg14	1	N/A
10495763	Gclm	7	N/A
10495781	Bcar3	19	N/A
10495933	4930422G04Rik	19	N/A
10495991		19	N/A
10495993	Elovl6	19	N/A
10496015	Pla2g12a	7	N/A
10496077	Agxt2l1	19	N/A
10496110	Papss1	19	N/A
10496251	Bdh2	13	N/A
10496338	Ppp3ca	7	N/A
10496387	Dnajb14	19	N/A
10496438	Adh1	19	N/A
10496492		7	N/A
10496569	Gbp6	7	N/A
10496605	Ccbl2	19	N/A
10496621	Gtf2b	7	N/A
10496748	Syde2	19	N/A
10496796	Ssx2ip	19	N/A
10496813	Ctbs	7	N/A
10496825	Uox	13	N/A
10496872	Eltd1	1	N/A
10497066	Zranb2	19	N/A
10497105	Lrrc40	7	N/A
10497186		19	N/A
10497337	Car1	13	N/A
10497379	Gm10742	1	N/A

10497390	Armc1	19	N/A
10497485		13	N/A
10497487		13	N/A
10497490	Naaladl2	13	N/A
10497501	Naaladl2	7	N/A
10497503		13	N/A
10497703	Mrpl47	13	N/A
10497964	Pgrmc2	1	N/A
10497971	Sclt1	7	N/A
10498018	Pcdh18	1	N/A
10498038	Elf2	7	N/A
10498313	Pgk1	19	N/A
10498367	P2ry13	1	N/A
10498401		19	N/A
10498407		19	N/A
10498413		13	N/A
10498593	Gm412	19	N/A
10498597	1110032F04Rik	19	N/A
10498599	Ift80	19	N/A
10498795	Zfp114	19	N/A
10498827	Fnip2	7	N/A
10498952	Gucy1a3	1	N/A
10498978	Lrat	1	N/A
10498990	Gm10710	19	N/A
10499091	Dear1	1	N/A
10499095	Fam160a1	1	N/A
10499121	Rps3a	19	N/A
10499130	Rnu73b	7	N/A
10499372	Slc25a44	13	N/A
10499431	Syt11	1	N/A
10499536	Efna1	1	N/A
10499655	Il6ra	13	N/A
10499705	Hax1	19	N/A
10499777	Ints3	1	N/A
10499839	Ilf2	7	N/A
10499948	Lce3a	7	N/A
10499971	Tdpoz1	19	N/A
10499975	Tdpoz1	19	N/A
10500042	Zfp687	7	N/A
10500054	Psmd4	1	N/A
10500283	Aph1a	1	N/A
10500304	Vps45	1	N/A
10500327	Hist2h3c2	19	N/A
10500329	Hist2h2aa1	1	N/A
10500445	Chd1l	13	N/A
10500685	Atp1a1	13	N/A
10500802	Atg4a	19	N/A
10500870	Gm5546	19	N/A
10500911	Mov10	7	N/A
10500948	Cttnbp2nl	1	N/A
10500960	Ddx20	7	N/A
10501046	Gm10673	19	N/A
10501063	Cd53	1	N/A
10501183	Eps8l3	19	N/A
10501199	Gstm7	1	N/A
10501235	Gstm4	1	N/A
10501282	Cyb561d1	7	N/A
10501485	Prmt6	19	N/A
10501608		13	N/A
10501661	Sfrs3	19	N/A
10502042	Alpk1	7	N/A
10502050	Alpk1	7	N/A
10502052	Alpk1	7	N/A
10502071	5730508B09Rik	19	N/A
10502146	Gar1	19	N/A
10502205	Hadh	13	N/A
10502214	Cyp2u1	7	N/A
10502417	2410002F23Rik	7	N/A
10502522	Hs2st1	1	N/A
10502655	Cyr61	13	N/A
10502732	Prkacb	13	N/A
10502766	Lphn2	1	N/A
10502776	Lphn2	1	N/A
10502949		1	N/A
10503023	Cth	1	N/A
10503123	Ubxn2b	1	N/A
10503172	Chd7	19	N/A
10503174	Chd7	13	N/A
10503178	Chd7	13	N/A
10503180	Chd7	19	N/A
10503186	Chd7	13	N/A
10503188	Chd7	13	N/A
10503190	Chd7	13	N/A
10503194	Chd7	19	N/A

10503196	Chd7	19	N/A
10503198	Chd7	13	N/A
10503200	Chd7	13	N/A
10503202	Chd7	13	N/A
10503210	Chd7	13	N/A
10503214	Chd7	19	N/A
10503218	Chd7	19	N/A
10503220	Chd7	19	N/A
10503222	Chd7	13	N/A
10503643	Ndufaf4	19	N/A
10503882	Rngtt	19	N/A
10503917	Akirin2	7	N/A
10503995	Dnaja1	13	N/A
10504106	Il11ra1	19	N/A
10504362	Rgp1	13	N/A
10504375	Npr2	13	N/A
10504466	Gm12504	19	N/A
10504615	E230008N13Rik	19	N/A
10504656		19	N/A
10504817	Tgfbr1	7	N/A
10504865	Invs	1	N/A
10504918	Zfp189	13	N/A
10505187	Ugcg	7	N/A
10505224	Snx30	7	N/A
10505240		19	N/A
10505747	Rraga	19	N/A
10505884		19	N/A
10505954	Tek	1	N/A
10506004	9530080O11Rik	19	N/A
10506154	Alg6	7	N/A
10506201	Ror1	1	N/A
10506213	Ube2u	1	N/A
10506269	Ak3l1	13	N/A
10506781	2010305A19Rik	7	N/A
10506786	Zcchc11	7	N/A
10506880	Kti12	13	N/A
10506893	Nrd1	19	N/A
10507011	Dmrta2	19	N/A
10507238	Lrrc41	1	N/A
10507347	Tesk2	13	N/A
10507394	Hectd3	7	N/A
10507500	Slc6a9	13	N/A
10507529	Med8	1	N/A
10507539	Elovl1	19	N/A
10507551	2610528J11Rik	1	N/A
10507657	Foxj3	7	N/A
10507699	Scmh1	1	N/A
10507775		19	N/A
10507851	Pabpc4	13	N/A
10508149	Tekt2	19	N/A
10508228	Zmym6	19	N/A
10508382	Ak2	19	N/A
10508608	Pef1	1	N/A
10508663	Laptm5	1	N/A
10508697	Sfrs4	13	N/A
10508707	Tmem200b	19	N/A
10508788	Ahdc1	1	N/A
10508800	LOC433762	13	N/A
10508883	1810019J16Rik	7	N/A
10508974	Pafah2	19	N/A
10509218	Zfp46	13	N/A
10509273	Wnt4	19	N/A
10509500	Hp1bp3	7	N/A
10509820	Rcc2	13	N/A
10509947	Arhgef19	19	N/A
10509965	Epha2	13	N/A
10510061	Pramef8	1	N/A
10510165	Cdv3	13	N/A
10510219		13	N/A
10510221		13	N/A
10510305	Mtor	7	N/A
10510391	Srm	13	N/A
10510814	BC046331	13	N/A
10511298	9430015G10Rik	19	N/A
10511375	Cyp7a1	7	N/A
10511382	Nsmaf	7	N/A
10511416	Tox	13	N/A
10511510	Ints8	19	N/A
10511661	Otud6b	13	N/A
10511703		7	N/A
10511865	Ptges3	19	N/A
10511881	Manea	7	N/A
10511886	Bhmt	19	N/A
10511892		1	N/A

10512024	Mobkl2b	7	N/A
10512061	Taf9	7	N/A
10512067	Ddx58	7	N/A
10512098	Aptx	7	N/A
10512251	AI464131	7	N/A
10512279	Cntfr	19	N/A
10512514	Tln1	1	N/A
10512574	Gba2	7	N/A
10512682		19	N/A
10512709	Mcart1	13	N/A
10512807	Gabbr2	7	N/A
10512851	Erp44	19	N/A
10512895	Baat	19	N/A
10512904	Aldob	19	N/A
10512935		13	N/A
10513141	Ptpn3	1	N/A
10513166	Ptpn3	1	N/A
10513190	D630039A03Rik	7	N/A
10513420	Mup7	19	N/A
10513428	Mup1	19	N/A
10513437	Mup1	19	N/A
10513455	Mup1	19	N/A
10513467	Mup1	19	N/A
10513472	Mup1	19	N/A
10513497	Mup1	19	N/A
10513504	Mup1	19	N/A
10513512		19	N/A
10513514	Mup5	19	N/A
10513521	Mup20	19	N/A
10513538	Mup21	19	N/A
10513608	Alad	13	N/A
10513803		19	N/A
10513955		19	N/A
10513957	Ptprd	19	N/A
10514133	Ttc39b	7	N/A
10514221	Plin2	7	N/A
10514329	Ifnz	19	N/A
10514338		19	N/A
10514588	I0C0044D17Rik	19	N/A
10514732	Slc35d1	13	N/A
10514912	Dio1	13	N/A
10515028	Zfyve9	13	N/A
10515051	Osbpl9	13	N/A
10515113		19	N/A
10515220		7	N/A
10515293	Llph	19	N/A
10515519	Atp6v0b	19	N/A
10515574	St3gal3	7	N/A
10515590	Kdm4a	1	N/A
10515613	Ptprf	1	N/A
10515692	BC059842	1	N/A
10515694	BC059842	1	N/A
10515698	BC059842	1	N/A
10515702	BC059842	1	N/A
10515704	BC059842	1	N/A
10515708	BC059842	1	N/A
10515712	BC059842	1	N/A
10515716	BC059842	1	N/A
10515729	BC059842	1	N/A
10515731	BC059842	1	N/A
10515733	BC059842	19	N/A
10515735	BC059842	1	N/A
10515737	BC059842	19	N/A
10515797	Tmem125	19	N/A
10515981		19	N/A
10516007	Zmpste24	19	N/A
10516303		7	N/A
10516427	Ncdn	13	N/A
10516466	Zmym1	1	N/A
10516507	Zscan20	19	N/A
10516520	Zfp362	7	N/A
10516551	S100pbp	7	N/A
10516678	Kpna6	13	N/A
10516706	Khdrbs1	7	N/A
10516823	Epb4.1	13	N/A
10516867	Gmeb1	1	N/A
10516884	Trnau1ap	13	N/A
10516908		19	N/A
10516966	BC013712	1	N/A
10516982		7	N/A
10517036	Wdtc1	7	N/A
10517081	Gm13213	7	N/A
10517083	Pigv	1	N/A
10517090	Arid1a	1	N/A

10517116	Rps6ka1	7	N/A
10517141		13	N/A
10517312	Tmem57	13	N/A
10517425	Lypla2	7	N/A
10517436	1110049F12Rik	1	N/A
10517513	C1qc	1	N/A
10517540	Zbtb40	19	N/A
10517587	Alpl	13	N/A
10517682	2310028O11Rik	19	N/A
10517892	D4Ertd22e	19	N/A
10517996	Plekhm2	7	N/A
10518019	Ddi2	7	N/A
10518226	Vps13d	13	N/A
10518300	Tnfrsf1b	13	N/A
10518344		13	N/A
10518385	Mfn2	13	N/A
10518473	Fbxo6	1	N/A
10518532	Tardbp	19	N/A
10518546	Pex14	7	N/A
10518585	Kif1b	7	N/A
10518642	Ube4b	7	N/A
10518774	Park7	19	N/A
10518781	Per3	13	N/A
10519177		19	N/A
10519488		1	N/A
10519642	Gm4959	19	N/A
10520080	Rint1	13	N/A
10520096	Klhl7	19	N/A
10520124	Sumo2	13	N/A
10520154	Abcb8	7	N/A
10520187	Slc4a2	13	N/A
10520250	Nub1	1	N/A
10520362	Insig1	19	N/A
10520371	Prr8	7	N/A
10520379	Prr8	7	N/A
10520390		19	N/A
10520513	4930471M23Rik	7	N/A
10520612	Khk	19	N/A
10520718	Snx17	13	N/A
10520734	Nrbp1	7	N/A
10520862	Fosl2	19	N/A
10521031	Ywhah	13	N/A
10521111	Fgfr3	7	N/A
10521182	BC037112	1	N/A
10521205	Sh3bp2	13	N/A
10521587		13	N/A
10521757		19	N/A
10521824	Sod3	1	N/A
10521830		13	N/A
10521832	Pi4k2b	19	N/A
10521907	1810013D10Rik	1	N/A
10521927	Tbc1d19	7	N/A
10521950	Stim2	13	N/A
10521966	Papd4	7	N/A
10521984	G6pd2	1	N/A
10522127	Klb	7	N/A
10522250	Tmem33	19	N/A
10522265	Slc30a9	13	N/A
10522301	Cmpk1	19	N/A
10522430	Dcun1d4	7	N/A
10522472	Fip1l1	1	N/A
10522589	Srd5a3	7	N/A
10522596	Tmem165	19	N/A
10522606	Exoc1	1	N/A
10522716	Polr2b	19	N/A
10523062	Alb	19	N/A
10523206	Uso1	19	N/A
10523245	Gm1381	13	N/A
10523255	Stbd1	7	N/A
10523281	Sept11	1	N/A
10523297	Ccng2	1	N/A
10523359	Cxcl13	1	N/A
10523451	Anxa3	1	N/A
10523481		1	N/A
10523727	Pkd2	19	N/A
10523758	Lrrc8b	13	N/A
10523901		7	N/A
10523903		19	N/A
10524004	Pcgf3	7	N/A
10524052	Fgfrl1	13	N/A
10524327	Mn1	7	N/A
10524345	Tpst2	19	N/A
10524460	Acacb	19	N/A
10524525	Ube3b	13	N/A

10524631	Oasl1	1	N/A
10524647	Sppl3	1	N/A
10524703	Pxn	7	N/A
10524723	Gcn1l1	1	N/A
10524973	2410131K14Rik	19	N/A
10524983	Med13l	7	N/A
10525016	Tbx3	7	N/A
10525187	Rpl6	13	N/A
10525313	Atxn2	7	N/A
10525374	Pptc7	13	N/A
10525439	P2rx4	7	N/A
10525473	Tmem120b	13	N/A
10525804		13	N/A
10525887	Bri3bp	1	N/A
10525942	Glt1d1	13	N/A
10526014	Sfrs8	7	N/A
10526069	Cct6a	19	N/A
10526098	Scand3	7	N/A
10526106	Vkorc1l1	13	N/A
10526113	Crcp	19	N/A
10526169	Caln1	13	N/A
10526181	Gatsl2	1	N/A
10526191	Gatsl2	1	N/A
10526232	Wbscr27	7	N/A
10526241	Cldn3	1	N/A
10526277	Mlxipl	1	N/A
10526302	Tbl2	19	N/A
10526487	Alkbh4	13	N/A
10526564	Ufsp1	13	N/A
10526566	Ephb4	7	N/A
10526656	Lrch4	1	N/A
10526687	2010007H12Rik	7	N/A
10526743	Cops6	19	N/A
10526772	Cnpy4	19	N/A
10526882	Heatr2	7	N/A
10526923	1110007L15Rik	13	N/A
10526972	Nudt1	1	N/A
10526977	Eif3b	19	N/A
10527026	AA881470	7	N/A
10527099		19	N/A
10527101	Foxk1	7	N/A
10527158	Fscn1	1	N/A
10527252	Eif2ak1	13	N/A
10527340	Trrap	7	N/A
10527423	Trrap	7	N/A
10527508	Cdk8	7	N/A
10527598	Pomp	13	N/A
10527624	Uspl1	13	N/A
10527646	BC028471	19	N/A
10528102	Crot	1	N/A
10528170	Cycs	19	N/A
10528238	Phtf2	13	N/A
10528385	Reln	1	N/A
10528507	Pus7	13	N/A
10528913	Gm5129	19	N/A
10529041	Preb	19	N/A
10529252	C330019G07Rik	1	N/A
10529264	Spon2	1	N/A
10529445	Lrpap1	13	N/A
10529547		13	N/A
10529567	D5Ertd579e	19	N/A
10529577	Cno	19	N/A
10529671	Slc2a9	7	N/A
10529797		13	N/A
10529873	Rab2a	19	N/A
10529875	Ldb2	1	N/A
10529895	Qdpr	1	N/A
10529957	Gpr125	1	N/A
10530017		19	N/A
10530194		19	N/A
10530692	Kdr	1	N/A
10530731	Srd5a3	19	N/A
10530806	Ppat	13	N/A
10530841	Igfbp7	1	N/A
10531100	Sult1d1	19	N/A
10531133	Grsf1	7	N/A
10531149	Gc	19	N/A
10531286	Vdac2	13	N/A
10531370	Naaa	1	N/A
10531560	Antxr2	19	N/A
10531796	Wdfy3	7	N/A
10531994	Mpa2l	1	N/A
10532308	Golga7	19	N/A
10532368	2410025L10Rik	7	N/A

10532472	Ulk1	7	N/A
10532584	Myo18b	19	N/A
10532669	2900026A02Rik	7	N/A
10532753	Coro1c	7	N/A
10532921	Sppl3	19	N/A
10532965	Rnf10	7	N/A
10532989	Gatc	1	N/A
10533304	Trafd1	7	N/A
10533323	Adam1a	13	N/A
10533345	Aldh2	7	N/A
10533751	Pitpnm2	13	N/A
10533929	Scarb1	1	N/A
10534120	Asl	13	N/A
10534301	Gm52	19	N/A
10534575	Prkrip1	19	N/A
10534921	Mepce	7	N/A
10535381	Actb	1	N/A
10535389	Rnf216	7	N/A
10535410		19	N/A
10535508	AU022870	13	N/A
10535559	Baiap2l1	13	N/A
10535586	Smurf1	7	N/A
10535776	Prhoxnb	13	N/A
10535841	Slc46a3	13	N/A
10535883	Katnal1	19	N/A
10535904	Hsph1	13	N/A
10535938	N4bp2l1	13	N/A
10536481	Gm3148	7	N/A
10536494	Cav2	13	N/A
10536593	Tsen15	19	N/A
10536743	6530409C15Rik	19	N/A
10536762	Snd1	19	N/A
10536931	Ahcyl2	19	N/A
10536994		19	N/A
10537062	Mest	1	N/A
10537227	Tmem140	1	N/A
10537316	Atp6v0c	13	N/A
10537347	Gm7504	19	N/A
10537437	Gm10244	19	N/A
10537728	Casp2	1	N/A
10537770	Zyx	13	N/A
10537834	Arhgef5	1	N/A
10537849	Arhgef5	1	N/A
10538100	Repin1	1	N/A
10538123	Gimap9	19	N/A
10538356	Chn2	13	N/A
10538394	Plekha8	13	N/A
10538413	Znrf2	1	N/A
10538588		19	N/A
10538640	Abcg2	19	N/A
10538753	Snx10	13	N/A
10538857	Serbp1	19	N/A
10538927	Amd1	13	N/A
10538932	Amd1	13	N/A
10538970	Krcc1	7	N/A
10539042	Polr1a	13	N/A
10539080	St3gal5	13	N/A
10539421	Mobkl1b	1	N/A
10539579	Gm5878	19	N/A
10539617	Alms1	1	N/A
10539649	Ptges3	19	N/A
10539861	Rpn1	19	N/A
10539975	V1ra7	19	N/A
10540034	Aldh1l1	19	N/A
10540072	Ppp1r2	13	N/A
10540075	Hdac11	7	N/A
10540191	Nr2c2	7	N/A
10540408	Itpr1	13	N/A
10540472	Bhlhe40	13	N/A
10540579	Mtmr14	7	N/A
10541034	Anubl1	19	N/A
10541071	8430408G22Rik	1	N/A
10541091	Gemin6	19	N/A
10541112	Lasp1	1	N/A
10541260	Cecr2	7	N/A
10541279	Bcl2l13	7	N/A
10541301	Tuba8	19	N/A
10541491	1700063H04Rik	19	N/A
10541771	Ing4	19	N/A
10541845	Nop2	13	N/A
10541885	Scnn1a	1	N/A
10541895	Tnfrsf1a	7	N/A
10542164	Clec12a	1	N/A
10542251	Tas2r116	13	N/A

10542302	Crebl2	19	N/A
10542340	8430419L09Rik	13	N/A
10542636	Pyroxd1	7	N/A
10542731	Rassf8	1	N/A
10542762	Gm6266	19	N/A
10542880	4833442J19Rik	19	N/A
10542911	Samd9l	1	N/A
10542949		19	N/A
10542953	Tfpi2	1	N/A
10543031	Slc25a13	13	N/A
10543226	2610001J05Rik	7	N/A
10543306	Tspan12	7	N/A
10543333	Aass	19	N/A
10543448	Wasl	7	N/A
10543524	Gcc1	19	N/A
10543650	Tnpo3	19	N/A
10543676	1700080G18Rik	19	N/A
10543737	Copg2	7	N/A
10543846	Slc35b4	19	N/A
10544133	Parp12	7	N/A
10544417	Epha1	7	N/A
10544525	Pdia4	1	N/A
10544547	Zfp777	7	N/A
10544629	Tra2a	19	N/A
10544906	Ggct	19	N/A
10545121	V1rc11	19	N/A
10545458	Tcf3	1	N/A
10545651	Ino80b	1	N/A
10545682		7	N/A
10545692	Tet3	7	N/A
10545720	Stambp	1	N/A
10545731	Clec4f	1	N/A
10545751	Tex261	13	N/A
10545760	Paip2b	19	N/A
10545835	1700040I03Rik	13	N/A
10546054		19	N/A
10546056	Rab43	13	N/A
10546294	Nup210	7	N/A
10546661	Foxp1	1	N/A
10546685	Eif4e3	13	N/A
10546760	Ddx3x	19	N/A
10546967	Sec13	13	N/A
10547251	Bms1	19	N/A
10547408	Erc1	19	N/A
10547436	Wnk1	1	N/A
10547531		13	N/A
10547621	Apobec1	1	N/A
10547769	Ptpn6	19	N/A
10547795	Rnu7	7	N/A
10547976		13	N/A
10548000	Ltbr	13	N/A
10548011	Plekhg6	7	N/A
10548038	Ntf3	19	N/A
10548194		19	N/A
10548207	Pzp	19	N/A
10548563		13	N/A
10548614	Tas2r114	19	N/A
10548701	Lrp6	7	N/A
10548808		1	N/A
10548815	Coq2	19	N/A
10548857	Hist4h4	19	N/A
10549079	Gys2	13	N/A
10549282	Itpr2	19	N/A
10549497	Fam60a	19	N/A
10549504	Dennd5b	7	N/A
10549506	Dennd5b	7	N/A
10549615	Leng8	7	N/A
10549653	Atp6v0c	13	N/A
10549700	Suv420h2	1	N/A
10549760	Zfp580	7	N/A
10549921	Vmn2r43	19	N/A
10549976	Gm4157	19	N/A
10549979	Tpm3	19	N/A
10550345	Strn4	7	N/A
10550826	V1rd20	13	N/A
10551069	Zfp526	7	N/A
10551197	Cyp2b10	19	N/A
10551423		7	N/A
10551469	Dyrk1b	7	N/A
10551487	Eid2b	1	N/A
10551554	Sars2	7	N/A
10551600	Hnrnpl	7	N/A
10552071	Tmem162	19	N/A
10552097		19	N/A

10552106		19	N/A
10552108		19	N/A
10552140	Cebpa	7	N/A
10552210	Ankrd27	13	N/A
10552240	Zfp507	19	N/A
10552311	Gm15470	19	N/A
10552358	C330019L16Rik	19	N/A
10552587	Klk1b21	1	N/A
10552656	Syt3	19	N/A
10552672	Aspdh	1	N/A
10552681	Josd2	1	N/A
10552715	2310016G11Rik	19	N/A
10552760	Pnkp	1	N/A
10552824	Rras	7	N/A
10553140	Tmem143	7	N/A
10553163	Nomo1	13	N/A
10553280	Gtf2h1	13	N/A
10553413	Prmt3	19	N/A
10553811		19	N/A
10553935	Tarsl2	19	N/A
10554057	Adamts17	19	N/A
10554081	Ttc23	19	N/A
10554156	Fam174b	19	N/A
10554475	Zfp592	1	N/A
10554549	Whamm	7	N/A
10554574	Tm6sf1	1	N/A
10554693	Stard5	19	N/A
10554752	Nox4	7	N/A
10554837	Eed	1	N/A
10555009		19	N/A
10555039	Kctd21	1	N/A
10555179	Prkrir	19	N/A
10555303	Pgm2l1	13	N/A
10555425	Fam168a	1	N/A
10555470	Arap1	1	N/A
10555548		19	N/A
10555590	Numa1	1	N/A
10555773	Olfr593	19	N/A
10556039	Olfr17	19	N/A
10556082	Ppfibp2	7	N/A
10556216	Ipo7	13	N/A
10556266	Wee1	13	N/A
10556658	9030624J02Rik	7	N/A
10556769	Acsm3	13	N/A
10556828	Anks4b	7	N/A
10556957	BC030336	13	N/A
10557009	Eef2k	13	N/A
10557035	Polr3e	13	N/A
10557148	Dctn5	1	N/A
10557213	Rbbp6	7	N/A
10557300	Aqp8	19	N/A
10557326	Il4ra	19	N/A
10557355	D430042O09Rik	1	N/A
10557357	D430042O09Rik	1	N/A
10557361	D430042O09Rik	7	N/A
10557374		1	N/A
10557394	D430042O09Rik	7	N/A
10557399	Sbk1	13	N/A
10557631	Prr14	1	N/A
10557644	Fbrs	1	N/A
10557655	Srcap	7	N/A
10557705	Phkg2	13	N/A
10557716	Rnf40	7	N/A
10557758	Setd1a	1	N/A
10557782	Hsd3b7	7	N/A
10557793	Stx4a	1	N/A
10557843	Fus	7	N/A
10558057	Brwd2	7	N/A
10558090	Tacc2	1	N/A
10558265	2310007H09Rik	7	N/A
10558297	2700050L05Rik	13	N/A
10558583	Zfp511	1	N/A
10558673	Cyp2e1	19	N/A
10558740	Gm15542	19	N/A
10558754	Athl1	7	N/A
10558880	Eps8l2	1	N/A
10558971	Ap2a2	1	N/A
10559310		19	N/A
10559436	Mboat7	1	N/A
10559516	Rdh13	13	N/A
10559606	Tmem86b	1	N/A
10559610	Saps1	7	N/A
10559708	Fiz1	7	N/A
10559762		19	N/A

10560023	Zfp551	19	N/A
10560091	V1rk1	19	N/A
10560160	Obox3	19	N/A
10560202	Gltscr1	1	N/A
10560260	Sae1	19	N/A
10560313		19	N/A
10560395		19	N/A
10560431	Foxa3	13	N/A
10560672	Cblc	1	N/A
10560818	Zfp111	19	N/A
10560826	Zfp109	1	N/A
10560993	Gsk3a	13	N/A
10561004	Erf	7	N/A
10561059	Gm7092	19	N/A
10561085	Hnrnpul1	1	N/A
10561323	Map3k10	19	N/A
10561525	Gm10648	19	N/A
10561527	Actn4	1	N/A
10561907	Sdhaf1	13	N/A
10562005	Wbp7	7	N/A
10562416	Cebpg	13	N/A
10562491	Pdcd5	19	N/A
10562548	C80913	7	N/A
10562563	Ccne1	13	N/A
10562578	Pop4	7	N/A
10562651		7	N/A
10562847	Nr1h2	7	N/A
10562905	Atf5	1	N/A
10562942	Med25	13	N/A
10563014	Prmt1	13	N/A
10563050	Prr12	1	N/A
10563110	Snord34	7	N/A
10563112	Snord33	1	N/A
10563253	Lin7b	19	N/A
10563295	Ftl1	19	N/A
10563314	Dhdh	7	N/A
10563362	Sphk2	1	N/A
10563641		19	N/A
10563768	Gm10628	1	N/A
10563816	Fancf	1	N/A
10563838	Nipa2	19	N/A
10563939		13	N/A
10563943		13	N/A
10564231	Ube2nl	19	N/A
10564290	Klf13	13	N/A
10564436	Asb7	7	N/A
10564527	Nr2f2	7	N/A
10564663	Tdg	7	N/A
10564805	Pex11a	13	N/A
10564809		13	N/A
10565002	Crtc3	1	N/A
10565072	Sec11a	19	N/A
10565081	Timm17a	13	N/A
10565152	9330120H11Rik	13	N/A
10565432		7	N/A
10565727	Tsku	13	N/A
10565811	Gm5775	7	N/A
10565819	Slco2b1	1	N/A
10565852	Rnf169	13	N/A
10565921		19	N/A
10565962	P2ry2	7	N/A
10566067	Rnf121	19	N/A
10566161	Trim68	1	N/A
10566168	Olfr33	13	N/A
10566190	Olfr575	1	N/A
10566229	Olfr624	7	N/A
10566326	Trim12	7	N/A
10566333	9230105E10Rik	7	N/A
10566346	9230105E10Rik	1	N/A
10566358	Trim30	1	N/A
10566366	AI451617	7	N/A
10566438	Fam160a2	7	N/A
10566488	Trim3	7	N/A
10566578	Gvin1	7	N/A
10566618	Olfr6	19	N/A
10566622		19	N/A
10566767	St5	1	N/A
10566877	Sbf2	7	N/A
10567043	Rras2	7	N/A
10567049	Copb1	7	N/A
10567213	LOC100047986	19	N/A
10567297	Itpripl2	1	N/A
10567316	Tmc7	13	N/A
10567423	Dcun1d3	13	N/A

10567496	Dnahc3	13	N/A
10567591	Usp31	13	N/A
10567608	Cog7	13	N/A
10567739	4933440M02Rik	1	N/A
10567797	Xpo6	7	N/A
10567903	Sh2b1	7	N/A
10568131	2900092E17Rik	19	N/A
10568252	Zfp689	19	N/A
10568296	Hsd3b7	1	N/A
10568432	Rnd2	19	N/A
10568480	Nsmce4a	7	N/A
10568521	2310057M21Rik	13	N/A
10568568	Oat	7	N/A
10568593	Mettl10	19	N/A
10568758	9430038I01Rik	1	N/A
10568780	Mapk1ip1	7	N/A
10568897	Tubgcp2	7	N/A
10568988	Bet1l	13	N/A
10569008	Cox8b	19	N/A
10569102	Irf7	13	N/A
10569149	Eps8l2	1	N/A
10569152	Pddc1	1	N/A
10569168	Slc25a22	7	N/A
10569265	Tollip	13	N/A
10569458		7	N/A
10569545	Nadsyn1	7	N/A
10569583	Ppfia1	19	N/A
10569611	Fadd	7	N/A
10569717		19	N/A
10569767	Zfp358	7	N/A
10569823	C330021F23Rik	1	N/A
10569830	2310057J16Rik	7	N/A
10569886	Trappc5	13	N/A
10569962	Ccl25	19	N/A
10570000	Gpi1	13	N/A
10570002	2410089E03Rik	7	N/A
10570144	Arhgef7	1	N/A
10570321	Cul4a	13	N/A
10570573	Agpat5	19	N/A
10570610	Defb4	19	N/A
10570837	Slc20a2	7	N/A
10571250	Hmgb1-rs17	19	N/A
10571302	Tmem66	19	N/A
10571415	Vps37a	1	N/A
10571621	Ufsp2	19	N/A
10571705	Irf2	7	N/A
10571715	Enpp6	19	N/A
10571737	AA386476	19	N/A
10571849	Fbxo8	1	N/A
10571984	Ddx60	7	N/A
10572095	Nat3	7	N/A
10572109	Ints10	1	N/A
10572180	Atp13a1	13	N/A
10572212	Gmip	7	N/A
10572282	Hapln4	1	N/A
10572419	Ell	7	N/A
10572533	Myo9b	7	N/A
10572786	Ap1m1	1	N/A
10572870	Hmgxb4	1	N/A
10572897	Hmox1	1	N/A
10572949	Nr3c2	7	N/A
10573112		19	N/A
10573261	Asf1b	19	N/A
10573344		19	N/A
10573519	Tnpo2	7	N/A
10573626	Gpt2	13	N/A
10573675	Lonp2	7	N/A
10573713	Heatr3	19	N/A
10573821		19	N/A
10573823	Chd9	19	N/A
10573865	LOC433762	13	N/A
10573893	Fto	7	N/A
10574023	Mt2	13	N/A
10574027	Mt1	13	N/A
10574087	Herpud1	13	N/A
10574137	Nlrc5	7	N/A
10574139	Nlrc5	7	N/A
10574145	Nlrc5	7	N/A
10574149	Nlrc5	7	N/A
10574157	Nlrc5	7	N/A
10574166	Cpne2	13	N/A
10574204	Arl2bp	7	N/A
10574436	Khdrbs1	13	N/A
10574438	Cdh5	1	N/A

10574595		19	N/A
10574617	Ces8	19	N/A
10574632	Cbfb	13	N/A
10574694	Elmo3	13	N/A
10574789	Fam65a	1	N/A
10574939		7	N/A
10574944	Dus2l	19	N/A
10574962	Nfatc3	1	N/A
10575052	Cdh1	1	N/A
10575120	Sntb2	1	N/A
10575144	Nip7	19	N/A
10575151	C630050I24Rik	19	N/A
10575184	Wwp2	7	N/A
10575249	Txnl4b	19	N/A
10575349	Tat	19	N/A
10575473	Gm9558	19	N/A
10575476	Vac14	13	N/A
10575578	4930402E16Rik	7	N/A
10575598	Znrf1	7	N/A
10575662	Mon1b	1	N/A
10575685	Nudt7	7	N/A
10575706	Wwox	7	N/A
10575750	Bcmo1	7	N/A
10575833	Hsd17b2	7	N/A
10576010	Gse1	13	N/A
10576090	Zfpm1	1	N/A
10576191	Spg7	19	N/A
10576274	Zfp276	13	N/A
10576305	Tcf25	13	N/A
10576354	Afg3l1	19	N/A
10576386	Rhou	1	N/A
10576659		19	N/A
10576696	Insr	7	N/A
10576774	Clec4g	1	N/A
10576873	Elavl1	1	N/A
10576901	Slc10a2	19	N/A
10576971	Irs2	19	N/A
10577517	Slc25a15	1	N/A
10577528	AI316807	7	N/A
10577641	1810011O10Rik	13	N/A
10577757	Adam9	19	N/A
10577808	Tacc1	19	N/A
10577838	Ddhd2	13	N/A
10577858	Bag4	7	N/A
10577882	Hgsnat	13	N/A
10578017	Gm9911	19	N/A
10578027	Mak16	13	N/A
10578109	Ubxn8	7	N/A
10578123	Rbpms	7	N/A
10578138	Dctn6	13	N/A
10578149	Leprotl1	1	N/A
10578155		19	N/A
10578222	Dlc1	1	N/A
10578320	Gm8423	1	N/A
10578391		1	N/A
10578427	F11	7	N/A
10578493	Tlr3	1	N/A
10578545		19	N/A
10578623	Wwc2	7	N/A
10578649	Odz3	7	N/A
10578679	Gm10675	19	N/A
10578685		19	N/A
10578703	Spcs3	19	N/A
10578757		1	N/A
10578810	Clcn3	19	N/A
10578922	Klhl2	19	N/A
10579199	Slc25a42	7	N/A
10579234	Upf1	13	N/A
10579331	Gdf15	19	N/A
10579479	Nr2f6	1	N/A
10579486	Ushbp1	19	N/A
10579649	Cib3	7	N/A
10579744	Large	7	N/A
10579769		19	N/A
10579852	Mmaa	19	N/A
10579925	Gab1	13	N/A
10579952	LOC675736	13	N/A
10579954		19	N/A
10579974	Tbc1d9	19	N/A
10579976	Elmod2	7	N/A
10580160	Mri1	13	N/A
10580210	Rad23a	13	N/A
10580370	Dnaja2	13	N/A
10580516	Gm6625	19	N/A

10580752	9330175E14Rik	7	N/A
10580885	Csnk2a2	7	N/A
10580957	Slc38a7	7	N/A
10581336	Gfod2	19	N/A
10581395	Slc12a4	13	N/A
10581523	Terf2	7	N/A
10581538	Nqo1	13	N/A
10581547	Nob1	13	N/A
10581813	Mlkl	7	N/A
10582209	Mthfsd	19	N/A
10582241	Zcchc14	13	N/A
10582295	Odc1	19	N/A
10582330	Rnf166	7	N/A
10582477	Spata2L	7	N/A
10582549		13	N/A
10582664	2310022B05Rik	1	N/A
10582694	Fam89a	19	N/A
10582708	Exoc8	19	N/A
10582811	Irf2bp2	1	N/A
10582814	Tomm20	13	N/A
10582896		19	N/A
10582958	Gucy1a2	1	N/A
10583254	Cwc15	1	N/A
10583326	Slc36a4	13	N/A
10583347	Chordc1	19	N/A
10583388	Olfr836	13	N/A
10583402	Zfp317	1	N/A
10583416	Gm7769	13	N/A
10583647	Dnm2	7	N/A
10583732	Ldlr	13	N/A
10583753		13	N/A
10583773	BC018242	19	N/A
10583920	Eepd1	7	N/A
10584057	Zbtb44	7	N/A
10584071	Prdm10	1	N/A
10584120		19	N/A
10584194	Srpr	19	N/A
10584200	Rpusd4	19	N/A
10584271	Tmem218	19	N/A
10584334	Siae	7	N/A
10584420	Olfr907	19	N/A
10584435	Vwa5a	7	N/A
10584576	Hspa8	13	N/A
10584578	Hspa8	13	N/A
10584580		13	N/A
10584589		1	N/A
10584712	Hyou1	1	N/A
10584741	Slc37a4	13	N/A
10584777	Ddx6	13	N/A
10584819		19	N/A
10584977	BC033915	7	N/A
10585129	Zw10	1	N/A
10585194	Il18	7	N/A
10585474	Psma4	7	N/A
10585533	Dnajb6	13	N/A
10585610	Ptpn9	1	N/A
10585625	Sin3a	7	N/A
10585699	Fabp5	1	N/A
10585721	Scamp2	1	N/A
10585842	Nptn	13	N/A
10585932	Pkm2	19	N/A
10586064	Anp32a	1	N/A
10586405	Spg21	7	N/A
10586458	Csnk1g1	7	N/A
10586484	Fam96a	19	N/A
10586491	Dapk2	1	N/A
10586700	Rora	19	N/A
10586759	Bnip2	19	N/A
10587211	Leo1	19	N/A
10587284	Elovl5	1	N/A
10587299	Ick	1	N/A
10587503	Sh3bgrl2	7	N/A
10587532	Gm5919	19	N/A
10587655	4930422I07Rik	19	N/A
10587746	Tmem41b	19	N/A
10587871	Paqr9	1	N/A
10588263	Slco2a1	1	N/A
10588294	Topbp1	19	N/A
10588357	Acad11	7	N/A
10588419	Aste1	13	N/A
10588683		7	N/A
10588826	Ip6k1	7	N/A
10588876	Nicn1	19	N/A
10588883	Amt	1	N/A

10588927	1700102P08Rik	19	N/A
10588975	Usp19	7	N/A
10589030	Qrich1	7	N/A
10589099	Ip6k2	1	N/A
10589420	Cdc25a	19	N/A
10589511	Scap	1	N/A
10589982	Azi2	7	N/A
10590149	Xylb	13	N/A
10590191	Wdr48	1	N/A
10590245	Slc25a38	19	N/A
10590343	Trak1	13	N/A
10590452	Abhd5	19	N/A
10590646		19	N/A
10590808	Yap1	7	N/A
10591009	Med17	7	N/A
10591110	Fat3	19	N/A
10591114	Fat3	19	N/A
10591127	Fat3	19	N/A
10591161	Zfp558	19	N/A
10591350	Angptl6	1	N/A
10591416		19	N/A
10591494	S1pr5	19	N/A
10591563	Kank2	7	N/A
10591643	Rab3d	13	N/A
10591658	LOC100049077	19	N/A
10591715	Zfp653	7	N/A
10591773		13	N/A
10592023	Aplp2	19	N/A
10592067	Fli1	1	N/A
10592084	St3gal4	13	N/A
10592114	Foxred1	19	N/A
10592160	Gm6762	1	N/A
10592342	Tbrg1	7	N/A
10592376	Olfr917	1	N/A
10592618	Tbcel	7	N/A
10592655	Arhgef12	7	N/A
10592719	Oaf	7	N/A
10592802	C2cd2l	13	N/A
10592919	Arcn1	19	N/A
10593103	Rnf214	1	N/A
10593159	Pafah1b2	19	N/A
10593213	Rbm7	7	N/A
10593225	Zbtb16	13	N/A
10593332	Bco2	19	N/A
10593413	2310030G06Rik	1	N/A
10593421	1110032A03Rik	7	N/A
10593483	Fdx1	13	N/A
10593646	Tnfaip8l3	19	N/A
10593776	Nrg4	13	N/A
10593878	Snx33	1	N/A
10593927	Scamp5	13	N/A
10594053	Pml	1	N/A
10594404	Smad3	7	N/A
10594501	Ptplad1	13	N/A
10594636	Ppp1r2	13	N/A
10594645	Rab8b	19	N/A
10594785	Rnf111	1	N/A
10594855	Cgnl1	13	N/A
10594988	Mapk6	7	N/A
10595404	Fam46a	13	N/A
10595680	Tbc1d2b	7	N/A
10596051	Tmem22	7	N/A
10596113		19	N/A
10596148	Trf	19	N/A
10596207	Uba5	19	N/A
10596433	Glyctk	19	N/A
10596442	Ppm1m	19	N/A
10596454	Alas1	13	N/A
10596481	Abhd14a	19	N/A
10596543	Rad54l2	13	N/A
10596545	Rad54l2	13	N/A
10596718	Slc38a3	7	N/A
10596737	Gnat1	7	N/A
10596815	Gmppb	7	N/A
10596931	Wdr6	7	N/A
10596951	Arih2	7	N/A
10597258	Tmie	1	N/A
10597352		19	N/A
10597413	Crtap	1	N/A
10597470	Cmtm8	7	N/A
10597493	Stt3b	19	N/A
10597518	Tgfbr2	13	N/A
10597573	Eif1	13	N/A
10597833	Sec22c	7	N/A

10597871	Higd1a	19	N/A
10597875	Cyp8b1	7	N/A
10597973	Lztfl1	7	N/A
10597978	Fyco1	7	N/A
10598018		19	N/A
10598020		19	N/A
10598025		19	N/A
10598027		19	N/A
10598029	ND1	19	N/A
10598034	ND2	19	N/A
10598036	COX1	19	N/A
10598038		19	N/A
10598043	Gm10925	19	N/A
10598049	COX3	19	N/A
10598053		19	N/A
10598055	ND3	19	N/A
10598057		19	N/A
10598059	ND4L	19	N/A
10598067		19	N/A
10598069		19	N/A
10598101	Maml2	19	N/A
10598111	Dhrsx	1	N/A
10598198	Mia3	19	N/A
10598409	Tcfe3	1	N/A
10598638	Mid1ip1	13	N/A
10599560		13	N/A
10599576	Ppdpf	1	N/A
10599612	Phf6	19	N/A
10599835		7	N/A
10599853	Ldoc1	19	N/A
10599925	Styx	7	N/A
10599997	Mtmr1	7	N/A
10600082	Nsdhl	7	N/A
10600148	DXBay18	19	N/A
10600169	Bgn	1	N/A
10600324		19	N/A
10600810	Gm9009	13	N/A
10600819	Zxdb	1	N/A
10600836	Msn	1	N/A
10600901	Ar	13	N/A
10600936	Efnb1	1	N/A
10601150		1	N/A
10601161	Gjb1	7	N/A
10601390	Pgk1	19	N/A
10601539		19	N/A
10601551	Gm16373	19	N/A
10601771	Armcx1	19	N/A
10601844	Bhlhb9	13	N/A
10601878	Tceal1	7	N/A
10602090	Atg4a	19	N/A
10602385	Pfkfb1	19	N/A
10602599	Smc1a	1	N/A
10602731	Gm8578	19	N/A
10602754		1	N/A
10603026	Ctps2	7	N/A
10603087	Pir	7	N/A
10603208	Mid1	1	N/A
10603354	Magix	1	N/A
10603387	Hdac6	7	N/A
10603567	Dynlt3	19	N/A
10603702	Llph	13	N/A
10603746	Maob	19	N/A
10604393	Gm9907	19	N/A
10604451	Enox2	7	N/A
10604614		19	N/A
10604653	Zfp36l3	19	N/A
10604671		19	N/A
10604687	Mmgt1	19	N/A
10604844		19	N/A
10604846	Gm6754	13	N/A
10604922	BC023829	19	N/A
10605195	Hcfc1	7	N/A
10605338	G6pdx	1	N/A
10605493	Prrg1	19	N/A
10605552		19	N/A
10605651		19	N/A
10605766	Maged1	19	N/A
10605820	Zc4h2	1	N/A
10605828	1700010D01Rik	19	N/A
10605848	Vsig4	1	N/A
10605919	Pja1	1	N/A
10605938	P2ry4	1	N/A
10606001	Snx12	1	N/A
10606016	Il2rg	1	N/A

10606315	Taf9b	7	N/A
10606436	Nsbp1	19	N/A
10606592	Tacc1	1	N/A
10606689	Timm8a1	13	N/A
10606924		19	N/A
10606936	Gm6322	19	N/A
10606989	Tsc22d3	19	N/A
10607089	Acsl4	19	N/A
10607250	Apex2	13	N/A
10607419		19	N/A
10607467	Sat1	7	N/A
10607619	Cdkl5	7	N/A
10607877	Prps2	7	N/A
10607910	Msl3	19	N/A
Liver Class IV			
Probe ID	**Gene ID**	**Con Peak**	**TSR Peak**
10344614	---	N/A	N/A
10344616	---	N/A	N/A
10344618	---	N/A	N/A
10344620	Gm10568	N/A	N/A
10344622	---	N/A	N/A
10344624	Lypla1	N/A	N/A
10344633	Tcea1	N/A	N/A
10344637	Atp6v1h	N/A	N/A
10344653	Oprk1	N/A	N/A
10344658	Rb1cc1	N/A	N/A
10344674	Fam150a	N/A	N/A
10344705	---	N/A	N/A
10344707	Pcmtd1	N/A	N/A
10344713	Ahcy	N/A	N/A
10344715	---	N/A	N/A
10344717	---	N/A	N/A
10344719	---	N/A	N/A
10344723	Rrs1	N/A	N/A
10344725	Adhfe1	N/A	N/A
10344741	Hnrnpa3	N/A	N/A
10344743	3110035E14Rik	N/A	N/A
10344789	Cspp1	N/A	N/A
10344797	Cspp1	N/A	N/A
10344799	Cspp1	N/A	N/A
10344801	Cspp1	N/A	N/A
10344803	Cspp1	N/A	N/A
10344805	Cspp1	N/A	N/A
10344807	Cspp1	N/A	N/A
10344809	Cspp1	N/A	N/A
10344811	Cspp1	N/A	N/A
10344813	Cspp1	N/A	N/A
10344815	Cspp1	N/A	N/A
10344817	Cspp1	N/A	N/A
10344819	Cspp1	N/A	N/A
10344821	Cspp1	N/A	N/A
10344835	---	N/A	N/A
10344837	Prex2	N/A	N/A
10344879	A830018L16Rik	N/A	N/A
10344895	---	N/A	N/A
10344897	Sulf1	N/A	N/A
10344922	---	N/A	N/A
10344931	Rpl5	N/A	N/A
10344933	---	N/A	N/A
10344935	Kcnb2	N/A	N/A
10344939	Terf1	N/A	N/A
10344950	Gm7634	N/A	N/A
10344952	Rdh10	N/A	N/A
10344966	Ly96	N/A	N/A
10344973	Gdap1	N/A	N/A
10344990	Crispld1	N/A	N/A
10345025	Iars	N/A	N/A
10345030	Mir133b	N/A	N/A
10345046	Efhc1	N/A	N/A
10345065	Gsta3	N/A	N/A
10345074	Cetn4	N/A	N/A
10345077	Khdc1a	N/A	N/A
10345087	Mir30a	N/A	N/A
10345089	Mir30c-2	N/A	N/A
10345091	B3gat2	N/A	N/A
10345099	1110058L19Rik	N/A	N/A
10345141	Lmbrd1	N/A	N/A
10345167	---	N/A	N/A
10345172	Gm9884	N/A	N/A
10345174	---	N/A	N/A
10345181	Gm6462	N/A	N/A
10345183	Cdk10	N/A	N/A
10345200	LOC100505265 /// Gm6489	N/A	N/A

10345203	Paox	N/A	N/A
10345206	---	N/A	N/A
10345212	Khdrbs2	N/A	N/A
10345224	---	N/A	N/A
10345226	---	N/A	N/A
10345228	1700001G17Rik	N/A	N/A
10345230	Rab23	N/A	N/A
10345241	Dst	N/A	N/A
10345350	---	N/A	N/A
10345357	Imp4	N/A	N/A
10345368	D1Ertd448e	N/A	N/A
10345387	Prss39	N/A	N/A
10345404	---	N/A	N/A
10345406	Arhgef4	N/A	N/A
10345409	Arhgef4	N/A	N/A
10345411	Arhgef4	N/A	N/A
10345423	Plekhb2	N/A	N/A
10345436	LOC280487	N/A	N/A
10345440	---	N/A	N/A
10345442	Hs6st1	N/A	N/A
10345482	Cnnm4	N/A	N/A
10345492	Cnnm3	N/A	N/A
10345504	Cox5b	N/A	N/A
10345527	---	N/A	N/A
10345546	Vwa3b	N/A	N/A
10345548	Vwa3b	N/A	N/A
10345550	Vwa3b	N/A	N/A
10345552	Vwa3b	N/A	N/A
10345554	Vwa3b	N/A	N/A
10345556	Vwa3b	N/A	N/A
10345580	Inpp4a	N/A	N/A
10345608	Unc50	N/A	N/A
10345616	Lipt1 /// Mitd1	N/A	N/A
10345620	Mrpl30	N/A	N/A
10345626	Eif5b	N/A	N/A
10345656	Nms	N/A	N/A
10345666	Pdcl3	N/A	N/A
10345698	Tbc1d8 /// Rpl31 /// Gm16382	N/A	N/A
10345704	---	N/A	N/A
10345706	D1Bwg0212e	N/A	N/A
10345762	Il1r1	N/A	N/A
10345777	Il1rl2	N/A	N/A
10345824	Il18rap	N/A	N/A
10345840	Slc9a4	N/A	N/A
10345875	---	N/A	N/A
10345877	Gm9915	N/A	N/A
10345879	Pou3f3	N/A	N/A
10345882	Mrps9	N/A	N/A
10345895	Gpr45	N/A	N/A
10345902	8430432A02Rik	N/A	N/A
10345904	AI597479	N/A	N/A
10345909	Rpl29 /// Gm8580 /// Rpl29-ps2	N/A	N/A
10345911	---	N/A	N/A
10345926	---	N/A	N/A
10345928	---	N/A	N/A
10345930	Tpp2	N/A	N/A
10345967	Bivm	N/A	N/A
10346000	Gulp1	N/A	N/A
10346067	---	N/A	N/A
10346069	---	N/A	N/A
10346072	---	N/A	N/A
10346074	Wdr75	N/A	N/A
10346107	Dnahc7b	N/A	N/A
10346109	Dnahc7b	N/A	N/A
10346114	---	N/A	N/A
10346137	---	N/A	N/A
10346139	---	N/A	N/A
10346150	Tmeff2	N/A	N/A
10346164	Sdpr	N/A	N/A
10346191	Stat1	N/A	N/A
10346222	LOC280487	N/A	N/A
10346224	Tmem194b	N/A	N/A
10346235	Hibch	N/A	N/A
10346255	Ormdl1	N/A	N/A
10346303	Hspe1 /// Hspe1-rs1	N/A	N/A
10346310	Mobkl3	N/A	N/A
10346321	Gm10561	N/A	N/A
10346328	---	N/A	N/A
10346330	Plcl1	N/A	N/A
10346337	1700066M21Rik	N/A	N/A
10346340	9430016H08Rik	N/A	N/A
10346348	Spats2l	N/A	N/A
10346365	Sgol2	N/A	N/A
10346374	Aox1	N/A	N/A

10346410	Aox3	N/A	N/A
10346523	Bzw1	N/A	N/A
10346533	Nif3l1 /// Ppil3	N/A	N/A
10346544	Ndufb3	N/A	N/A
10346549	Cbx3 /// Gm6901	N/A	N/A
10346551	Cflar	N/A	N/A
10346562	Cflar	N/A	N/A
10346564	Casp8	N/A	N/A
10346592	Als2cr4	N/A	N/A
10346607	Fzd7	N/A	N/A
10346634	Nop58	N/A	N/A
10346651	Bmpr2	N/A	N/A
10346668	Fam117b	N/A	N/A
10346678	Carf	N/A	N/A
10346695	Nbeal1	N/A	N/A
10346722	Nbeal1	N/A	N/A
10346747	Cyp20a1	N/A	N/A
10346762	---	N/A	N/A
10346764	Abi2	N/A	N/A
10346780	Eif4a1	N/A	N/A
10346808	Rpl17	N/A	N/A
10346810	Pard3b	N/A	N/A
10346838	Pard3b	N/A	N/A
10346840	---	N/A	N/A
10346843	Nrp2	N/A	N/A
10346867	Eef1b2	N/A	N/A
10346874	---	N/A	N/A
10346876	Snora41	N/A	N/A
10346878	Zdbf2	N/A	N/A
10346882	Adam23	N/A	N/A
10346914	Fastkd2	N/A	N/A
10346938	Gm13749	N/A	N/A
10346941	---	N/A	N/A
10346943	Creb1	N/A	N/A
10346960	Ccnyl1	N/A	N/A
10346970	Pikfyve	N/A	N/A
10347033	Crygf	N/A	N/A
10347036	Mtap2	N/A	N/A
10347058	---	N/A	N/A
10347060	Unc80	N/A	N/A
10347073	Unc80	N/A	N/A
10347106	Rpe	N/A	N/A
10347117	Cps1	N/A	N/A
10347186	---	N/A	N/A
10347188	Vwc2l	N/A	N/A
10347193	Atic	N/A	N/A
10347216	Rpl21 /// Gm5528	N/A	N/A
10347218	---	N/A	N/A
10347232	Xrcc5	N/A	N/A
10347254	Smarcal1	N/A	N/A
10347277	Igfbp2	N/A	N/A
10347324	Gm216	N/A	N/A
10347362	Mir26b	N/A	N/A
10347386	Rqcd1	N/A	N/A
10347398	Plcd4 /// Zfp142	N/A	N/A
10347417	Bcs1l	N/A	N/A
10347460	Ttll4	N/A	N/A
10347481	Cyp27a1	N/A	N/A
10347497	Wnt10a	N/A	N/A
10347503	Cdk5r2	N/A	N/A
10347508	Fam134a	N/A	N/A
10347521	Zfand2b	N/A	N/A
10347552	Stk16 /// Tuba4a	N/A	N/A
10347593	Speg	N/A	N/A
10347639	Gmppa	N/A	N/A
10347650	Accn4	N/A	N/A
10347662	Tmem198	N/A	N/A
10347669	Inha	N/A	N/A
10347672	Stk11ip	N/A	N/A
10347724	Slc4a3	N/A	N/A
10347726	LOC100504876	N/A	N/A
10347728	---	N/A	N/A
10347730	---	N/A	N/A
10347732	---	N/A	N/A
10347734	Sgpp2	N/A	N/A
10347748	Utp14b /// Acsl3	N/A	N/A
10347767	Kcne4	N/A	N/A
10347772	Gm10555	N/A	N/A
10347774	Mrpl44	N/A	N/A
10347779	9830004L10Rik	N/A	N/A
10347781	9430031J16Rik	N/A	N/A
10347790	---	N/A	N/A
10347792	---	N/A	N/A
10347796	Rhbdd1	N/A	N/A

10347860	---	N/A	N/A
10347862	Mff	N/A	N/A
10347873	Agfg1	N/A	N/A
10347910	Fbxo36	N/A	N/A
10347915	Gm7609 /// Csprs /// Gm7592	N/A	N/A
10347919	A530040E14Rik	N/A	N/A
10347925	Gm7609 /// Csprs /// Gm7592	N/A	N/A
10347928	Sp110	N/A	N/A
10347931	G530012D18Rik	N/A	N/A
10347933	Sp140 /// C130026I21Rik /// A530032D15Rik /// Arhgef12	N/A	N/A
10347968	Sp100	N/A	N/A
10347970	Cab39	N/A	N/A
10347980	Itm2c	N/A	N/A
10347988	4933407L21Rik	N/A	N/A
10347992	Spata3	N/A	N/A
10348000	2810459M11Rik	N/A	N/A
10348030	---	N/A	N/A
10348032	Armc9	N/A	N/A
10348062	B3gnt7	N/A	N/A
10348070	Ncl /// C130036L24Rik	N/A	N/A
10348072	---	N/A	N/A
10348076	Rpl30-ps6	N/A	N/A
10348087	Cops7b	N/A	N/A
10348096	Dis3l2	N/A	N/A
10348150	Chrnd	N/A	N/A
10348194	Efhd1	N/A	N/A
10348201	Gigyf2	N/A	N/A
10348234	Neu2	N/A	N/A
10348240	3110079O15Rik	N/A	N/A
10348244	Inpp5d	N/A	N/A
10348277	Atg16l1	N/A	N/A
10348299	5830472F04Rik	N/A	N/A
10348301	Sag	N/A	N/A
10348321	Dgkd	N/A	N/A
10348354	Ugt1a6a /// Ugt1a1 /// Ugt1a6b /// Ugt1a2 /// Ugt1a5 /// Ugt1a7c /// Ugt1a9 /// Ugt1a10	N/A	N/A
10348376	Heatr7b1	N/A	N/A
10348410	Spp2	N/A	N/A
10348420	---	N/A	N/A
10348424	Sh3bp4	N/A	N/A
10348432	Agap1	N/A	N/A
10348468	Gm9991	N/A	N/A
10348489	Prlh	N/A	N/A
10348493	Lrrfip1	N/A	N/A
10348547	Ube2f	N/A	N/A
10348556	---	N/A	N/A
10348558	Scly	N/A	N/A
10348570	Espnl	N/A	N/A
10348580	Klhl30	N/A	N/A
10348600	Traf3ip1	N/A	N/A
10348618	Asb1	N/A	N/A
10348632	Twist2	N/A	N/A
10348635	Olfr1413	N/A	N/A
10348637	Olfr1412	N/A	N/A
10348639	Olfr1411	N/A	N/A
10348641	Olfr1410	N/A	N/A
10348643	Olfr12	N/A	N/A
10348653	Gpc1	N/A	N/A
10348664	Mir149	N/A	N/A
10348666	Dusp28	N/A	N/A
10348670	Rnpepl1	N/A	N/A
10348682	Capn10	N/A	N/A
10348702	Agxt	N/A	N/A
10348739	Sned1	N/A	N/A
10348775	Ppp1r7	N/A	N/A
10348789	---	N/A	N/A
10348791	Ano7	N/A	N/A
10348817	Sep 02	N/A	N/A
10348829	Farp2 /// Stk25	N/A	N/A
10348858	Bok	N/A	N/A
10348864	Thap4 /// Gm10550	N/A	N/A
10348866	Atg4b	N/A	N/A
10348879	Ing5	N/A	N/A
10348889	D2hgdh	N/A	N/A
10348896	Gal3st2 /// Gm6086	N/A	N/A
10348902	Gal3st2 /// Gm6086	N/A	N/A
10348917	Fam174a	N/A	N/A
10348927	---	N/A	N/A
10348929	Hnrnpf	N/A	N/A
10348932	Gin1 /// Ppip5k2	N/A	N/A
10348945	---	N/A	N/A
10348963	Cntnap5b	N/A	N/A
10348996	---	N/A	N/A
10349014	Capza1	N/A	N/A
10349016	2310035C23Rik	N/A	N/A

10349049	Zfp706	N/A	N/A
10349065	Zcchc2	N/A	N/A
10349081	Phlpp1	N/A	N/A
10349100	---	N/A	N/A
10349108	Serpinb5	N/A	N/A
10349118	Serpinb12	N/A	N/A
10349174	Serpinb8	N/A	N/A
10349184	Cdh7	N/A	N/A
10349206	---	N/A	N/A
10349208	Cntnap5a	N/A	N/A
10349237	---	N/A	N/A
10349239	Mki67ip	N/A	N/A
10349249	Clasp1	N/A	N/A
10349314	---	N/A	N/A
10349316	Tmem185b	N/A	N/A
10349333	3110009E18Rik	N/A	N/A
10349340	C1ql2	N/A	N/A
10349348	Ccdc93	N/A	N/A
10349376	---	N/A	N/A
10349378	Dpp10	N/A	N/A
10349383	Slc35f5	N/A	N/A
10349401	Gpr39 /// Lypd1	N/A	N/A
10349404	Mgat5	N/A	N/A
10349427	---	N/A	N/A
10349429	---	N/A	N/A
10349442	Ccnt2	N/A	N/A
10349453	Rab3gap1	N/A	N/A
10349480	R3hdm1	N/A	N/A
10349510	Mir128-1	N/A	N/A
10349512	Ubxn4	N/A	N/A
10349527	---	N/A	N/A
10349569	---	N/A	N/A
10349580	Pigr	N/A	N/A
10349610	Lgtn	N/A	N/A
10349671	Slc26a9	N/A	N/A
10349694	Pm20d1	N/A	N/A
10349711	Slc41a1	N/A	N/A
10349724	Rab7l1	N/A	N/A
10349733	Nucks1	N/A	N/A
10349744	Slc45a3	N/A	N/A
10349752	Elk4	N/A	N/A
10349769	Mir135b	N/A	N/A
10349771	Gm7241	N/A	N/A
10349773	Klhdc8a	N/A	N/A
10349782	Nuak2	N/A	N/A
10349793	Dstyk	N/A	N/A
10349809	Rbbp5	N/A	N/A
10349821	Tmem81	N/A	N/A
10349826	Gm10538	N/A	N/A
10349828	Lrrn2	N/A	N/A
10349832	---	N/A	N/A
10349834	Pik3c2b	N/A	N/A
10349868	Ppp1r15b	N/A	N/A
10349872	---	N/A	N/A
10349876	Plekha6	N/A	N/A
10349904	Golt1a	N/A	N/A
10349913	Kiss1	N/A	N/A
10349922	Ren2	N/A	N/A
10349932	Etnk2	N/A	N/A
10349945	Gm10537	N/A	N/A
10349947	Fmod	N/A	N/A
10349953	Chit1	N/A	N/A
10349968	Chi3l1	N/A	N/A
10349980	Adora1	N/A	N/A
10350003	Cyb5r1	N/A	N/A
10350015	Adipor1	N/A	N/A
10350024	Klhl12	N/A	N/A
10350039	Rabif	N/A	N/A
10350044	Rpl29 /// Gm3550 /// Rpl29-ps2	N/A	N/A
10350046	Kdm5b	N/A	N/A
10350077	Syt2	N/A	N/A
10350090	Ube2t	N/A	N/A
10350099	Ptprv /// Gm10535	N/A	N/A
10350113	Arl8a	N/A	N/A
10350128	Nav1 /// Gm4793	N/A	N/A
10350136	Csrp1	N/A	N/A
10350146	Phlda3	N/A	N/A
10350173	Tnnt2	N/A	N/A
10350188	Tmem9	N/A	N/A
10350197	Ascl5-ps	N/A	N/A
10350247	Kif21b	N/A	N/A
10350286	Ddx59	N/A	N/A
10350329	Kif14	N/A	N/A
10350331	Zfp281	N/A	N/A

10350335	Hmbs	N/A	N/A
10350337	A130050O07Rik	N/A	N/A
10350341	Mir181b-1	N/A	N/A
10350349	Dennd1b	N/A	N/A
10350377	Zbtb41	N/A	N/A
10350425	F13b	N/A	N/A
10350438	Kcnt2	N/A	N/A
10350471	---	N/A	N/A
10350473	B3galt2	N/A	N/A
10350489	Uchl5	N/A	N/A
10350502	---	N/A	N/A
10350504	---	N/A	N/A
10350506	Fam5c	N/A	N/A
10350535	Tpr	N/A	N/A
10350590	Fam33a	N/A	N/A
10350592	3110040M04Rik	N/A	N/A
10350594	Ivns1abp	N/A	N/A
10350614	1190005F20Rik	N/A	N/A
10350646	Edem3	N/A	N/A
10350668	Glt25d2	N/A	N/A
10350684	Arpc5	N/A	N/A
10350697	Nmnat2	N/A	N/A
10350710	1700012A16Rik	N/A	N/A
10350723	---	N/A	N/A
10350725	Rgs8	N/A	N/A
10350733	Rgs16	N/A	N/A
10350742	Rnasel	N/A	N/A
10350749	Gm5531	N/A	N/A
10350751	Teddm1	N/A	N/A
10350753	Glul	N/A	N/A
10350758	A930039A15Rik	N/A	N/A
10350766	Stx6	N/A	N/A
10350777	Acbd6	N/A	N/A
10350790	Tor1aip1 /// Tor1aip2	N/A	N/A
10350792	Tor1aip2	N/A	N/A
10350800	Tor1aip2	N/A	N/A
10350806	Nphs2	N/A	N/A
10350816	Rpl35 /// Gm10269	N/A	N/A
10350819	Csnk2a1	N/A	N/A
10350823	Abl2	N/A	N/A
10350838	2810417H13Rik	N/A	N/A
10350846	Gm10531	N/A	N/A
10350864	Sec16b	N/A	N/A
10350894	---	N/A	N/A
10350896	Astn1	N/A	N/A
10350921	Mir488	N/A	N/A
10350923	Rfwd2	N/A	N/A
10350948	---	N/A	N/A
10350951	Tnr	N/A	N/A
10350975	Gm10530	N/A	N/A
10350977	4930523C07Rik /// 4930562F07Rik	N/A	N/A
10350985	Mrps14	N/A	N/A
10350990	Rpl23a /// Gm6177	N/A	N/A
10350992	Rc3h1	N/A	N/A
10351013	Rc3h1	N/A	N/A
10351015	Serpinc1	N/A	N/A
10351026	Gas5	N/A	N/A
10351035	Gas5	N/A	N/A
10351037	Gas5	N/A	N/A
10351039	Gas5	N/A	N/A
10351041	---	N/A	N/A
10351043	Snord47	N/A	N/A
10351045	---	N/A	N/A
10351056	Ankrd45	N/A	N/A
10351063	---	N/A	N/A
10351111	Mir214 /// Mir199a-2 /// Dnm3os	N/A	N/A
10351117	Dnm3 /// Gm10176	N/A	N/A
10351119	Vamp4	N/A	N/A
10351136	---	N/A	N/A
10351138	---	N/A	N/A
10351140	Kifap3	N/A	N/A
10351163	Scyl3	N/A	N/A
10351179	2810422O20Rik	N/A	N/A
10351224	F5	N/A	N/A
10351259	Slc19a2	N/A	N/A
10351269	4930455F23Rik	N/A	N/A
10351277	Nme7	N/A	N/A
10351293	Dpt	N/A	N/A
10351298	Gpr161	N/A	N/A
10351304	Brp44	N/A	N/A
10351345	---	N/A	N/A
10351347	Creg1	N/A	N/A
10351380	Ildr2	N/A	N/A
10351390	Tada1	N/A	N/A

10351400	Fam78b	N/A	N/A
10351404	Tmco1	N/A	N/A
10351414	Aldh9a1	N/A	N/A
10351430	Rxrg	N/A	N/A
10351455	Rgs5	N/A	N/A
10351463	Rgs5	N/A	N/A
10351471	Uhmk1	N/A	N/A
10351482	1700015E13Rik	N/A	N/A
10351487	Nos1ap /// Gm10174	N/A	N/A
10351489	---	N/A	N/A
10351500	---	N/A	N/A
10351502	Dusp12	N/A	N/A
10351504	---	N/A	N/A
10351507	Gm10522	N/A	N/A
10351515	Rnu1b1 /// Rnu1b6 /// Rnu1b2	N/A	N/A
10351517	1700009P17Rik	N/A	N/A
10351533	Nr1i3	N/A	N/A
10351546	Apoa2	N/A	N/A
10351551	Adamts4	N/A	N/A
10351574	Dedd	N/A	N/A
10351583	Pfdn2	N/A	N/A
10351623	F11r	N/A	N/A
10351636	Refbp2	N/A	N/A
10351644	LOC677008 /// Cd244	N/A	N/A
10351665	Ccdc72	N/A	N/A
10351689	Gm10521	N/A	N/A
10351703	Copa	N/A	N/A
10351781	Kcnj10	N/A	N/A
10351788	Pigm	N/A	N/A
10351852	Crp	N/A	N/A
10351857	Olfr16	N/A	N/A
10351859	---	N/A	N/A
10351861	Olfr218	N/A	N/A
10351863	Olfr1404	N/A	N/A
10351865	Olfr418-ps1	N/A	N/A
10351867	Aim2	N/A	N/A
10351880	E430029J22Rik	N/A	N/A
10351884	Olfr433	N/A	N/A
10351886	Olfr432	N/A	N/A
10351891	Olfr429	N/A	N/A
10351893	Olfr427	N/A	N/A
10351895	Olfr424	N/A	N/A
10351897	LOC100505119	N/A	N/A
10351903	Olfr420	N/A	N/A
10351959	1810030J14Rik	N/A	N/A
10351961	Olfr417	N/A	N/A
10351968	Olfr220	N/A	N/A
10351971	Fmn2	N/A	N/A
10351998	---	N/A	N/A
10352000	Kmo	N/A	N/A
10352064	---	N/A	N/A
10352092	Zfp238	N/A	N/A
10352104	Gm16432	N/A	N/A
10352110	Gm16432	N/A	N/A
10352119	Pppde1	N/A	N/A
10352125	Gm9982	N/A	N/A
10352127	Fam36a	N/A	N/A
10352131	---	N/A	N/A
10352133	Efcab2	N/A	N/A
10352143	Kif26b	N/A	N/A
10352150	---	N/A	N/A
10352152	Kif26b	N/A	N/A
10352166	Cnst	N/A	N/A
10352178	Sccpdh	N/A	N/A
10352192	Ahctf1 /// Gm10518	N/A	N/A
10352194	Cdc42bpa	N/A	N/A
10352234	Itpkb	N/A	N/A
10352242	Parp1	N/A	N/A
10352281	Acbd3	N/A	N/A
10352292	---	N/A	N/A
10352294	BC031781	N/A	N/A
10352306	Pycr2	N/A	N/A
10352314	Lefty1	N/A	N/A
10352320	Tmem63a	N/A	N/A
10352348	Cnih4	N/A	N/A
10352354	A430110L20Rik	N/A	N/A
10352356	Haus5	N/A	N/A
10352396	Trp53bp2	N/A	N/A
10352439	Susd4	N/A	N/A
10352457	Rpl21 /// Rpl21-ps1	N/A	N/A
10352459	Rab3gap2	N/A	N/A
10352497	Mir664 /// Snora36b	N/A	N/A
10352499	Mir194-1	N/A	N/A
10352503	Bpnt1	N/A	N/A

10352514	Eprs	N/A	N/A
10352554	---	N/A	N/A
10352562	Gpatch2	N/A	N/A
10352576	Esrrg	N/A	N/A
10352661	Ptpn14	N/A	N/A
10352703	Mfsd7b /// A230020J21Rik	N/A	N/A
10352709	Nsl1	N/A	N/A
10352722	---	N/A	N/A
10352725	Tmem206	N/A	N/A
10352735	Ints7	N/A	N/A
10352756	Lpgat1	N/A	N/A
10352767	Nek2	N/A	N/A
10352777	Slc30a1	N/A	N/A
10352792	---	N/A	N/A
10352794	Gm10516	N/A	N/A
10352798	Kcnh1	N/A	N/A
10352813	---	N/A	N/A
10352827	A130010J15Rik	N/A	N/A
10352829	A130010J15Rik /// Traf3ip3	N/A	N/A
10352864	---	N/A	N/A
10352905	Cd34	N/A	N/A
10352914	A330023F24Rik	N/A	N/A
10352916	A330023F24Rik /// Mir29b-2	N/A	N/A
10352918	A330023F24Rik /// Mir29c	N/A	N/A
10352920	Xkr4	N/A	N/A
10352926	---	N/A	N/A
10352928	Rp1	N/A	N/A
10352936	Sox17	N/A	N/A
10352945	---	N/A	N/A
10352947	Mrpl15	N/A	N/A
10352957	Rgs20	N/A	N/A
10352969	Npbwr1	N/A	N/A
10352971	Rb1cc1 /// 4732440D04Rik	N/A	N/A
10352980	Sntg1	N/A	N/A
10353002	---	N/A	N/A
10353004	Cks2	N/A	N/A
10353008	---	N/A	N/A
10353028	Vcpip1	N/A	N/A
10353032	Gm6195	N/A	N/A
10353034	Snord87	N/A	N/A
10353036	Gm10567	N/A	N/A
10353048	---	N/A	N/A
10353050	Cops5	N/A	N/A
10353061	---	N/A	N/A
10353064	Arfgef1	N/A	N/A
10353115	---	N/A	N/A
10353117	Slco5a1	N/A	N/A
10353135	Ncoa2	N/A	N/A
10353165	---	N/A	N/A
10353167	Tram1	N/A	N/A
10353181	Lactb2	N/A	N/A
10353189	Gm5523 /// Gm12070 /// Gapdh	N/A	N/A
10353219	Sumo2	N/A	N/A
10353221	Trpa1	N/A	N/A
10353250	Gapdh /// Gm2451	N/A	N/A
10353252	Gm106	N/A	N/A
10353258	---	N/A	N/A
10353272	Stau2	N/A	N/A
10353296	Tceb1	N/A	N/A
10353303	---	N/A	N/A
10353305	---	N/A	N/A
10353307	---	N/A	N/A
10353320	Crisp4	N/A	N/A
10353330	Defb44-ps	N/A	N/A
10353333	Defb18	N/A	N/A
10353343	---	N/A	N/A
10353413	---	N/A	N/A
10353438	Tram2	N/A	N/A
10353450	Gm4956	N/A	N/A
10353460	Kcnq5	N/A	N/A
10353475	Eif3m	N/A	N/A
10353477	---	N/A	N/A
10353479	Rims1	N/A	N/A
10353519	4933415F23Rik	N/A	N/A
10353524	Ogfrl1	N/A	N/A
10353533	B3gat2 /// Smap1	N/A	N/A
10353545	1110058L19Rik	N/A	N/A
10353549	Fam135a	N/A	N/A
10353574	Col19a1	N/A	N/A
10353624	ND4L	N/A	N/A
10353626	ATP6 /// Gm10925 /// ATP8	N/A	N/A
10353630	Prf1 /// COX2	N/A	N/A
10353632	Bai3	N/A	N/A
10353667	---	N/A	N/A

10353669	4931408C20Rik	N/A	N/A
10353672	---	N/A	N/A
10353674	Lsm5	N/A	N/A
10353676	Gm597	N/A	N/A
10353679	---	N/A	N/A
10353684	---	N/A	N/A
10353686	Gm9898	N/A	N/A
10353689	Phf3	N/A	N/A
10353707	Ptp4a1 /// Gm13363	N/A	N/A
10353727	---	N/A	N/A
10353729	Gm5415 /// 4930444G20Rik /// Gm9839	N/A	N/A
10353731	Gm5415 /// 4930444G20Rik /// Gm9839	N/A	N/A
10353733	Prim2	N/A	N/A
10353754	Zfp451	N/A	N/A
10353773	Gm15455	N/A	N/A
10353775	Bend6	N/A	N/A
10353783	Ccdc115	N/A	N/A
10353786	Prss40	N/A	N/A
10353792	1700101I19Rik	N/A	N/A
10353794	Fam168b	N/A	N/A
10353803	Uggt1	N/A	N/A
10353849	4632411B12Rik	N/A	N/A
10353871	Lman2l	N/A	N/A
10353878	Ankrd23 /// Ankrd39	N/A	N/A
10353891	Ankrd39 /// Ankrd23	N/A	N/A
10353934	Actr1b	N/A	N/A
10353947	Tmem131	N/A	N/A
10353989	---	N/A	N/A
10353991	Rpl12 /// Rpl12-ps1	N/A	N/A
10353993	6330578E17Rik	N/A	N/A
10354003	Mgat4a	N/A	N/A
10354019	2010300C02Rik	N/A	N/A
10354054	Lipt1 /// Mitd1	N/A	N/A
10354065	Lyg2	N/A	N/A
10354072	Lyg1	N/A	N/A
10354079	Txndc9	N/A	N/A
10354085	Rev1 /// Eif5b	N/A	N/A
10354111	Aff3	N/A	N/A
10354141	Lonrf2	N/A	N/A
10354157	Chst10	N/A	N/A
10354168	Tbc1d8	N/A	N/A
10354203	---	N/A	N/A
10354205	---	N/A	N/A
10354207	Creg2	N/A	N/A
10354216	Rfx8	N/A	N/A
10354220	Mfsd9	N/A	N/A
10354227	---	N/A	N/A
10354229	2610017I09Rik	N/A	N/A
10354233	Tgfbrap1	N/A	N/A
10354258	Uxs1	N/A	N/A
10354273	---	N/A	N/A
10354286	Kdelc1	N/A	N/A
10354307	Txn1	N/A	N/A
10354368	---	N/A	N/A
10354372	Myl6 /// Gm8894	N/A	N/A
10354374	Slc40a1	N/A	N/A
10354389	Slc39a10	N/A	N/A
10354400	---	N/A	N/A
10354404	Dnajb6	N/A	N/A
10354408	---	N/A	N/A
10354411	---	N/A	N/A
10354414	---	N/A	N/A
10354416	---	N/A	N/A
10354418	Obfc2a	N/A	N/A
10354432	Myo1b	N/A	N/A
10354468	---	N/A	N/A
10354470	Rpl15 /// Gm4294	N/A	N/A
10354472	Gls	N/A	N/A
10354494	Nab1	N/A	N/A
10354504	---	N/A	N/A
10354506	Mfsd6	N/A	N/A
10354542	Pms1	N/A	N/A
10354555	Asnsd1	N/A	N/A
10354563	Dnahc7b	N/A	N/A
10354576	Dnahc7a /// Dnahc7b	N/A	N/A
10354581	---	N/A	N/A
10354588	Stk17b	N/A	N/A
10354598	Hecw2	N/A	N/A
10354630	Gtf3c3	N/A	N/A
10354644	---	N/A	N/A
10354647	Pgap1	N/A	N/A
10354649	Pgap1	N/A	N/A
10354677	Ankrd44	N/A	N/A
10354704	Sf3b1	N/A	N/A

10354730	Coq10b	N/A	N/A
10354732	Hspd1	N/A	N/A
10354737	---	N/A	N/A
10354739	Atp5l	N/A	N/A
10354741	Rftn2 /// Gm10561	N/A	N/A
10354753	Boll	N/A	N/A
10354768	Akr1b3 /// Gm6644	N/A	N/A
10354777	Satb2	N/A	N/A
10354788	---	N/A	N/A
10354792	1110034B05Rik /// 9430016H08Rik	N/A	N/A
10354807	Kctd18	N/A	N/A
10354832	Nif3l1 /// Ppil3	N/A	N/A
10354845	Orc2	N/A	N/A
10354868	Fam126b	N/A	N/A
10354881	---	N/A	N/A
10354897	Trak2	N/A	N/A
10354916	---	N/A	N/A
10354919	---	N/A	N/A
10354938	Als2cr4	N/A	N/A
10354979	Als2 /// Mpp4	N/A	N/A
10355017	Sumo1	N/A	N/A
10355024	Ica1l	N/A	N/A
10355037	Wdr12	N/A	N/A
10355050	Raph1	N/A	N/A
10355069	Ino80d	N/A	N/A
10355084	Ndufs1	N/A	N/A
10355105	---	N/A	N/A
10355109	Gpr1	N/A	N/A
10355113	---	N/A	N/A
10355115	Prelid1	N/A	N/A
10355138	---	N/A	N/A
10355141	Klf7	N/A	N/A
10355147	Fam119a	N/A	N/A
10355152	Fzd5	N/A	N/A
10355173	Rpl10a /// Rpl10a-ps2	N/A	N/A
10355193	Crygc	N/A	N/A
10355197	---	N/A	N/A
10355199	Crygb	N/A	N/A
10355205	D630023F18Rik	N/A	N/A
10355214	Idh1	N/A	N/A
10355225	---	N/A	N/A
10355227	1110028C15Rik /// Rpe	N/A	N/A
10355246	Acadl	N/A	N/A
10355264	---	N/A	N/A
10355266	Lancl1	N/A	N/A
10355278	Erbb4	N/A	N/A
10355310	---	N/A	N/A
10355312	Ikzf2	N/A	N/A
10355325	Bard1	N/A	N/A
10355327	Bard1	N/A	N/A
10355341	---	N/A	N/A
10355403	Fn1	N/A	N/A
10355452	---	N/A	N/A
10355454	---	N/A	N/A
10355456	Mreg	N/A	N/A
10355474	March4	N/A	N/A
10355479	Ankar	N/A	N/A
10355500	Igfbp5	N/A	N/A
10355511	Tnp1	N/A	N/A
10355514	Tns1	N/A	N/A
10355528	Tns1	N/A	N/A
10355530	Tns1	N/A	N/A
10355532	Tns1	N/A	N/A
10355534	Tns1	N/A	N/A
10355550	Cxcr1	N/A	N/A
10355554	Aamp	N/A	N/A
10355567	Tmbim1	N/A	N/A
10355611	Zfp142 /// Plcd4	N/A	N/A
10355628	Rnf25	N/A	N/A
10355658	Fev	N/A	N/A
10355668	Mir375	N/A	N/A
10355670	Ccdc108	N/A	N/A
10355711	Nhej1	N/A	N/A
10355717	Slc23a3	N/A	N/A
10355730	1810031K17Rik	N/A	N/A
10355742	Abcb6	N/A	N/A
10355785	Glb1l /// Ankzf1	N/A	N/A
10355813	Ptprn	N/A	N/A
10355844	Dnpep	N/A	N/A
10355893	Epha4	N/A	N/A
10355914	---	N/A	N/A
10355931	Farsb /// Sgpp2	N/A	N/A
10355954	BC035947	N/A	N/A
10355958	Ube2v1	N/A	N/A

10355967	Ap1s3	N/A	N/A
10355974	Wdfy1	N/A	N/A
10355984	Serpine2	N/A	N/A
10355996	Slc25a5	N/A	N/A
10356001	Cul3	N/A	N/A
10356018	---	N/A	N/A
10356020	Dock10	N/A	N/A
10356082	Plscr1	N/A	N/A
10356084	Irs1	N/A	N/A
10356088	Col4a4	N/A	N/A
10356143	---	N/A	N/A
10356145	Slc19a3	N/A	N/A
10356154	Sphkap	N/A	N/A
10356170	---	N/A	N/A
10356172	Pid1	N/A	N/A
10356177	Dner	N/A	N/A
10356194	Trip12	N/A	N/A
10356262	Csprs /// Gm2619 /// Gm7592	N/A	N/A
10356267	A530032D15Rik	N/A	N/A
10356269	A530032D15Rik /// Sp140	N/A	N/A
10356271	A530032D15Rik	N/A	N/A
10356274	Csprs /// Gm7609 /// Gm7592	N/A	N/A
10356278	Sp110 /// C130026I21Rik /// LOC677525 /// LOC100041885	N/A	N/A
10356291	A530040E14Rik	N/A	N/A
10356312	Ncl	N/A	N/A
10356329	Snora75	N/A	N/A
10356331	---	N/A	N/A
10356333	Snord82	N/A	N/A
10356339	Pde6d	N/A	N/A
10356345	Nppc	N/A	N/A
10356351	Rpl6 /// Gm6807	N/A	N/A
10356379	Ecel1	N/A	N/A
10356401	---	N/A	N/A
10356403	Kcnj13	N/A	N/A
10356406	Ngef	N/A	N/A
10356457	Dnajb3	N/A	N/A
10356461	Hjurp /// 6430706D22Rik	N/A	N/A
10356475	Arl4c	N/A	N/A
10356482	---	N/A	N/A
10356498	Iqca	N/A	N/A
10356508	---	N/A	N/A
10356510	Iqca	N/A	N/A
10356512	Iqca	N/A	N/A
10356514	Iqca	N/A	N/A
10356577	Ilkap /// Fam132b	N/A	N/A
10356593	Hes6	N/A	N/A
10356626	---	N/A	N/A
10356628	Hdac4	N/A	N/A
10356657	Ndufa10	N/A	N/A
10356671	Olfr1416	N/A	N/A
10356675	Olfr1414	N/A	N/A
10356710	Capn10	N/A	N/A
10356712	Kif1a	N/A	N/A
10356762	LOC280487	N/A	N/A
10356764	2310007B03Rik	N/A	N/A
10356771	Sned1 /// Mterfd2	N/A	N/A
10356778	Rpl17	N/A	N/A
10356780	Pask	N/A	N/A
10356800	Hdlbp	N/A	N/A
10356859	Dtymk	N/A	N/A
10356873	---	N/A	N/A
10356876	---	N/A	N/A
10356878	---	N/A	N/A
10356880	St8sia4	N/A	N/A
10356886	Slco4c1	N/A	N/A
10356916	---	N/A	N/A
10356932	D1Ertd622e	N/A	N/A
10356936	Ppip5k2	N/A	N/A
10356968	Pam	N/A	N/A
10356995	---	N/A	N/A
10356997	---	N/A	N/A
10356999	Prdx2	N/A	N/A
10357001	---	N/A	N/A
10357003	Rnf152	N/A	N/A
10357008	Pign	N/A	N/A
10357043	Bcl2	N/A	N/A
10357064	Vps4b	N/A	N/A
10357103	Cdh19	N/A	N/A
10357115	Dsel	N/A	N/A
10357120	---	N/A	N/A
10357122	---	N/A	N/A
10357124	Tsn	N/A	N/A
10357133	---	N/A	N/A
10357155	Inhbb	N/A	N/A

10357158	Ralb	N/A	N/A
10357191	Ptpn4	N/A	N/A
10357220	Tmem177	N/A	N/A
10357242	Dbi	N/A	N/A
10357249	Steap3	N/A	N/A
10357288	Htr5b	N/A	N/A
10357292	Ddx18	N/A	N/A
10357298	LOC280487	N/A	N/A
10357300	Dpp10	N/A	N/A
10357328	---	N/A	N/A
10357332	Actr3	N/A	N/A
10357339	Gpr39 /// Lypd1	N/A	N/A
10357345	Nckap5	N/A	N/A
10357371	Tmem163	N/A	N/A
10357381	Ysk4	N/A	N/A
10357454	Dars	N/A	N/A
10357472	Cxcr4	N/A	N/A
10357478	Rpl31 /// Gm16382	N/A	N/A
10357514	---	N/A	N/A
10357516	C4bp	N/A	N/A
10357535	Pfkfb2	N/A	N/A
10357579	Mapkapk2	N/A	N/A
10357594	Rassf5	N/A	N/A
10357630	Srgap2	N/A	N/A
10357656	---	N/A	N/A
10357658	---	N/A	N/A
10357660	Mfsd4	N/A	N/A
10357696	Gm10188	N/A	N/A
10357698	Tmcc2	N/A	N/A
10357705	Cntn2	N/A	N/A
10357736	Nfasc	N/A	N/A
10357773	Mdm4	N/A	N/A
10357788	Ppp1r15b	N/A	N/A
10357833	Atp2b4	N/A	N/A
10357856	---	N/A	N/A
10357875	Btg2	N/A	N/A
10357878	Adora1	N/A	N/A
10357886	---	N/A	N/A
10357932	Ppp1r12b	N/A	N/A
10357944	Ppp1r12b	N/A	N/A
10357946	Ppp1r12b	N/A	N/A
10357948	Ppp1r12b	N/A	N/A
10357950	Ppp1r12b	N/A	N/A
10357952	Ppp1r12b	N/A	N/A
10357954	Ppp1r12b	N/A	N/A
10358023	Gpr37l1	N/A	N/A
10358038	Rnpep	N/A	N/A
10358050	Timm17a	N/A	N/A
10358057	Shisa4	N/A	N/A
10358064	Ipo9	N/A	N/A
10358091	Nav1	N/A	N/A
10358191	Camsap1l1	N/A	N/A
10358210	Nr5a2	N/A	N/A
10358259	Nek7	N/A	N/A
10358272	Lhx9	N/A	N/A
10358281	---	N/A	N/A
10358299	EG214403 /// Cfhr1	N/A	N/A
10358324	Cfhr3	N/A	N/A
10358330	BC026782	N/A	N/A
10358339	Cfh	N/A	N/A
10358357	Gm4845	N/A	N/A
10358359	Cdc73	N/A	N/A
10358379	Trove2	N/A	N/A
10358389	Rgs2	N/A	N/A
10358428	---	N/A	N/A
10358432	Zfp825	N/A	N/A
10358457	Bex4	N/A	N/A
10358459	BC003331 /// Tpr	N/A	N/A
10358476	Prg4	N/A	N/A
10358513	Hmcn1	N/A	N/A
10358515	Hmcn1	N/A	N/A
10358517	Hmcn1	N/A	N/A
10358519	Hmcn1	N/A	N/A
10358521	Hmcn1	N/A	N/A
10358523	Hmcn1	N/A	N/A
10358525	Hmcn1	N/A	N/A
10358527	Hmcn1	N/A	N/A
10358529	Hmcn1	N/A	N/A
10358531	Hmcn1	N/A	N/A
10358533	Hmcn1	N/A	N/A
10358535	Hmcn1	N/A	N/A
10358537	Hmcn1	N/A	N/A
10358539	Hmcn1	N/A	N/A
10358541	Hmcn1	N/A	N/A

10358543	Hmcn1	N/A	N/A
10358545	Hmcn1	N/A	N/A
10358547	Hmcn1	N/A	N/A
10358549	Hmcn1	N/A	N/A
10358553	Hmcn1	N/A	N/A
10358555	Hmcn1	N/A	N/A
10358557	Hmcn1	N/A	N/A
10358559	Hmcn1	N/A	N/A
10358561	Hmcn1	N/A	N/A
10358563	Hmcn1	N/A	N/A
10358565	Hmcn1	N/A	N/A
10358567	Hmcn1	N/A	N/A
10358569	Hmcn1	N/A	N/A
10358571	Hmcn1	N/A	N/A
10358573	Hmcn1	N/A	N/A
10358575	Hmcn1	N/A	N/A
10358577	Hmcn1	N/A	N/A
10358579	Hmcn1	N/A	N/A
10358581	Hmcn1	N/A	N/A
10358583	Hmcn1	N/A	N/A
10358585	Hmcn1	N/A	N/A
10358587	Hmcn1	N/A	N/A
10358589	Hmcn1	N/A	N/A
10358591	Hmcn1	N/A	N/A
10358593	Hmcn1	N/A	N/A
10358595	Hmcn1	N/A	N/A
10358597	Hmcn1	N/A	N/A
10358599	Hmcn1	N/A	N/A
10358601	Hmcn1	N/A	N/A
10358603	Hmcn1	N/A	N/A
10358607	Hmcn1	N/A	N/A
10358609	Hmcn1	N/A	N/A
10358611	Hmcn1	N/A	N/A
10358613	Hmcn1	N/A	N/A
10358615	Hmcn1	N/A	N/A
10358617	Hmcn1	N/A	N/A
10358619	Hmcn1	N/A	N/A
10358621	Hmcn1	N/A	N/A
10358623	Hmcn1	N/A	N/A
10358625	Hmcn1	N/A	N/A
10358627	Hmcn1	N/A	N/A
10358629	Hmcn1	N/A	N/A
10358631	Hmcn1	N/A	N/A
10358633	Hmcn1	N/A	N/A
10358635	Hmcn1	N/A	N/A
10358648	Hmcn1	N/A	N/A
10358650	Hmcn1	N/A	N/A
10358652	Hmcn1	N/A	N/A
10358654	Hmcn1	N/A	N/A
10358656	Hmcn1	N/A	N/A
10358658	Hmcn1	N/A	N/A
10358660	Hmcn1	N/A	N/A
10358662	Hmcn1	N/A	N/A
10358664	Hmcn1	N/A	N/A
10358666	Hmcn1	N/A	N/A
10358668	Hmcn1	N/A	N/A
10358670	Hmcn1	N/A	N/A
10358672	Hmcn1	N/A	N/A
10358675	Ivns1abp	N/A	N/A
10358677	1200016B10Rik	N/A	N/A
10358698	Rnf2	N/A	N/A
10358709	Cox7b	N/A	N/A
10358711	---	N/A	N/A
10358713	1700025G04Rik	N/A	N/A
10358717	1700025G04Rik	N/A	N/A
10358726	Tsen15	N/A	N/A
10358733	Rgl1	N/A	N/A
10358754	---	N/A	N/A
10358757	Smg7	N/A	N/A
10358816	Lamc1	N/A	N/A
10358849	Dhx9	N/A	N/A
10358892	F630010C12Rik	N/A	N/A
10358894	Sord	N/A	N/A
10358921	5830403L16Rik	N/A	N/A
10358926	---	N/A	N/A
10358928	Cacna1e	N/A	N/A
10358978	Ier5	N/A	N/A
10358982	Mr1	N/A	N/A
10358999	Xpr1	N/A	N/A
10359024	---	N/A	N/A
10359034	Qsox1	N/A	N/A
10359050	Cep350	N/A	N/A
10359078	Cep350	N/A	N/A
10359080	Cep350	N/A	N/A

10359082	Cep350	N/A	N/A
10359084	Cep350	N/A	N/A
10359086	Cep350	N/A	N/A
10359097	Tor1aip1	N/A	N/A
10359113	Fam163a	N/A	N/A
10359161	Soat1	N/A	N/A
10359181	Tor3a	N/A	N/A
10359190	Fam20b	N/A	N/A
10359201	Ralgps2	N/A	N/A
10359235	Rasal2	N/A	N/A
10359280	Pappa2	N/A	N/A
10359293	1600012P17Rik	N/A	N/A
10359297	Atp5h /// Gm4953 /// Gm5048	N/A	N/A
10359339	Rabgap1l	N/A	N/A
10359375	Gpr52	N/A	N/A
10359377	Zbtb37	N/A	N/A
10359386	Dars2	N/A	N/A
10359405	Klhl20	N/A	N/A
10359419	4930469G21Rik	N/A	N/A
10359422	Prdx6	N/A	N/A
10359428	Aph1a	N/A	N/A
10359446	AI848100	N/A	N/A
10359480	Dnm3	N/A	N/A
10359504	Dnm3os	N/A	N/A
10359518	Eif4e1b	N/A	N/A
10359525	Bat2l2	N/A	N/A
10359561	Fmo4	N/A	N/A
10359582	Fmo2	N/A	N/A
10359624	Prrx1	N/A	N/A
10359635	Gorab	N/A	N/A
10359642	LOC280487	N/A	N/A
10359644	Mettl11b	N/A	N/A
10359689	Atp1b1	N/A	N/A
10359734	Dcaf6	N/A	N/A
10359754	Mpzl1	N/A	N/A
10359770	Pou2f1	N/A	N/A
10359814	Ildr2	N/A	N/A
10359816	Pogk	N/A	N/A
10359826	Uqcr11	N/A	N/A
10359849	Uck2	N/A	N/A
10359851	Uck2	N/A	N/A
10359859	---	N/A	N/A
10359861	Mgst3	N/A	N/A
10359867	Lrrc52	N/A	N/A
10359870	Pbx1	N/A	N/A
10359888	Gm5265	N/A	N/A
10359908	Rgs4	N/A	N/A
10359917	Hsd17b7	N/A	N/A
10359929	Ddr2	N/A	N/A
10359948	Uap1	N/A	N/A
10359961	Uhmk1	N/A	N/A
10359970	Nos1ap	N/A	N/A
10359982	Atf6	N/A	N/A
10360001	---	N/A	N/A
10360012	Fcrlb	N/A	N/A
10360026	Uqcrb /// Gm5457	N/A	N/A
10360040	Fcgr3	N/A	N/A
10360046	Sdhc	N/A	N/A
10360053	Pcp4l1	N/A	N/A
10360058	Tomm40l	N/A	N/A
10360070	Fcer1g	N/A	N/A
10360076	Ndufs2	N/A	N/A
10360090	Ppox	N/A	N/A
10360105	Usp21	N/A	N/A
10360120	Ufc1	N/A	N/A
10360130	Nit1 /// Dedd	N/A	N/A
10360139	Klhdc9	N/A	N/A
10360145	B930036N10Rik	N/A	N/A
10360147	Refbp2	N/A	N/A
10360185	---	N/A	N/A
10360205	Ncstn /// Copa	N/A	N/A
10360225	Timm23	N/A	N/A
10360227	Pea15a	N/A	N/A
10360270	Atp1a2	N/A	N/A
10360321	Dusp23	N/A	N/A
10360324	Gm2710	N/A	N/A
10360332	---	N/A	N/A
10360336	Olfr1406	N/A	N/A
10360349	Cadm3	N/A	N/A
10360367	---	N/A	N/A
10360377	AI607873	N/A	N/A
10360391	Ifi203	N/A	N/A
10360410	Olfr231	N/A	N/A
10360415	Grem2	N/A	N/A

10360418	Rgs7	N/A	N/A
10360443	Fh1	N/A	N/A
10360454	Opn3	N/A	N/A
10360460	Chml	N/A	N/A
10360463	Pld5	N/A	N/A
10360479	Cep170	N/A	N/A
10360504	Mir350	N/A	N/A
10360506	Akt3	N/A	N/A
10360538	Pppde1	N/A	N/A
10360540	4930527J03Rik	N/A	N/A
10360542	AI503316 /// Fam36a	N/A	N/A
10360544	Hnrnpu	N/A	N/A
10360578	Rpl29 /// Gm3550 /// Rpl29-ps2	N/A	N/A
10360580	Tfb2m	N/A	N/A
10360589	Ahctf1	N/A	N/A
10360629	Rpl21	N/A	N/A
10360648	Psen2	N/A	N/A
10360662	Gm5069	N/A	N/A
10360664	6330403A02Rik /// Gm10001	N/A	N/A
10360679	H3f3a /// Gm12657	N/A	N/A
10360684	Ephx1	N/A	N/A
10360695	Nvl	N/A	N/A
10360737	Rpl35a	N/A	N/A
10360741	Ccdc121	N/A	N/A
10360745	Lbr	N/A	N/A
10360764	Enah	N/A	N/A
10360789	Degs1	N/A	N/A
10360794	Fbxo28	N/A	N/A
10360802	---	N/A	N/A
10360804	Gm10517	N/A	N/A
10360832	1700056E22Rik	N/A	N/A
10360840	Mosc1	N/A	N/A
10360848	Mosc2	N/A	N/A
10360858	C130074G19Rik	N/A	N/A
10360863	Mark1	N/A	N/A
10360884	Iars2	N/A	N/A
10360909	9630028B13Rik	N/A	N/A
10360912	---	N/A	N/A
10360914	Lyplal1	N/A	N/A
10360920	Tgfb2	N/A	N/A
10360934	Rrp15	N/A	N/A
10360957	Kctd3	N/A	N/A
10360972	Kcnk2	N/A	N/A
10360983	Pdcd5	N/A	N/A
10361021	---	N/A	N/A
10361023	Prox1	N/A	N/A
10361031	Rps6kc1	N/A	N/A
10361055	Vash2	N/A	N/A
10361063	---	N/A	N/A
10361065	Mfsd7b	N/A	N/A
10361075	Mfsd7b	N/A	N/A
10361078	Tatdn3	N/A	N/A
10361089	Fam71a	N/A	N/A
10361098	Nenf	N/A	N/A
10361104	Ppp2r5a /// Tmem206	N/A	N/A
10361129	---	N/A	N/A
10361131	---	N/A	N/A
10361133	---	N/A	N/A
10361135	Slc30a1 /// 1700034H15Rik	N/A	N/A
10361152	Gstp2 /// Gstp1	N/A	N/A
10361156	Rcor3	N/A	N/A
10361184	A730013G03Rik	N/A	N/A
10361186	Sertad4	N/A	N/A
10361191	Syt14	N/A	N/A
10361200	AA408296	N/A	N/A
10361234	Hsd11b1	N/A	N/A
10361246	G0s2	N/A	N/A
10361250	Camk1g	N/A	N/A
10361267	---	N/A	N/A
10361270	Cd46	N/A	N/A
10361323	Cnksr3	N/A	N/A
10361356	---	N/A	N/A
10361358	Rgs17	N/A	N/A
10361366	Mtrf1l	N/A	N/A
10361381	Syne1	N/A	N/A
10361507	---	N/A	N/A
10361509	Syne1	N/A	N/A
10361620	Rmnd1 /// Gm5512	N/A	N/A
10361640	---	N/A	N/A
10361642	Lrp11	N/A	N/A
10361651	Nup43	N/A	N/A
10361660	Lats1	N/A	N/A
10361669	Katna1	N/A	N/A
10361682	Ppil4	N/A	N/A

10361698	Zc3h12d	N/A	N/A
10361705	Sash1	N/A	N/A
10361708	---	N/A	N/A
10361710	Gm5177	N/A	N/A
10361748	Fbxo30	N/A	N/A
10361754	Epm2a	N/A	N/A
10361760	Timm8a1	N/A	N/A
10361763	---	N/A	N/A
10361767	Sf3b5	N/A	N/A
10361771	Plagl1	N/A	N/A
10361787	---	N/A	N/A
10361790	Fuca2	N/A	N/A
10361799	Adat2	N/A	N/A
10361807	Hivep2	N/A	N/A
10361824	---	N/A	N/A
10361846	Reps1	N/A	N/A
10361867	Gm10827	N/A	N/A
10361882	Nhsl1	N/A	N/A
10361887	Perp	N/A	N/A
10361892	---	N/A	N/A
10361897	Ifngr1	N/A	N/A
10361926	Map3k5	N/A	N/A
10361956	Mtap7	N/A	N/A
10361977	Gm6251	N/A	N/A
10361979	Bclaf1	N/A	N/A
10362003	F730021E23Rik	N/A	N/A
10362005	Ahi1	N/A	N/A
10362036	Hbs1l	N/A	N/A
10362052	Aldh8a1	N/A	N/A
10362062	1700021A07Rik	N/A	N/A
10362073	Sgk1	N/A	N/A
10362102	Gm10825	N/A	N/A
10362104	Slc2a12	N/A	N/A
10362111	---	N/A	N/A
10362113	Gm10824	N/A	N/A
10362115	1110021L09Rik	N/A	N/A
10362129	Vnn3	N/A	N/A
10362138	Vnn1	N/A	N/A
10362147	Taar1	N/A	N/A
10362156	Taar5	N/A	N/A
10362158	Taar7b	N/A	N/A
10362162	Taar7d	N/A	N/A
10362164	Taar7e	N/A	N/A
10362168	Taar8b	N/A	N/A
10362171	Stx7	N/A	N/A
10362208	---	N/A	N/A
10362210	Med23	N/A	N/A
10362243	---	N/A	N/A
10362271	---	N/A	N/A
10362273	Tmem200a /// Gm9767	N/A	N/A
10362294	Arhgap18	N/A	N/A
10362310	Ube2l3 /// LOC100502680 /// Gm10145	N/A	N/A
10362314	Ptprk	N/A	N/A
10362359	Pebp1	N/A	N/A
10362363	6330407J23Rik	N/A	N/A
10362372	9330159F19Rik	N/A	N/A
10362379	Echdc1	N/A	N/A
10362387	---	N/A	N/A
10362389	---	N/A	N/A
10362394	Hddc2	N/A	N/A
10362402	---	N/A	N/A
10362418	Trdn	N/A	N/A
10362420	Trdn	N/A	N/A
10362424	Trdn	N/A	N/A
10362426	Trdn	N/A	N/A
10362428	Trdn	N/A	N/A
10362432	Trdn	N/A	N/A
10362434	Trdn	N/A	N/A
10362440	Trdn	N/A	N/A
10362442	Trdn	N/A	N/A
10362446	Trdn	N/A	N/A
10362450	Trdn	N/A	N/A
10362462	Trdn	N/A	N/A
10362472	Rsph4a	N/A	N/A
10362487	Tspyl1	N/A	N/A
10362490	Tspyl4	N/A	N/A
10362495	Col10a1	N/A	N/A
10362511	Gstm3	N/A	N/A
10362513	Hs3st5	N/A	N/A
10362520	Hdac2	N/A	N/A
10362534	5930403N24Rik	N/A	N/A
10362536	---	N/A	N/A
10362581	Tube1	N/A	N/A
10362593	Npm1	N/A	N/A

10362596	Fyn	N/A	N/A
10362615	Traf3ip2	N/A	N/A
10362628	---	N/A	N/A
10362630	---	N/A	N/A
10362633	Rev3l	N/A	N/A
10362669	4930547M16Rik	N/A	N/A
10362674	Rnu3a	N/A	N/A
10362676	Cdk19	N/A	N/A
10362717	Wasf1	N/A	N/A
10362745	Akd1	N/A	N/A
10362758	Zbtb24	N/A	N/A
10362794	Ppil6 /// Smpd2	N/A	N/A
10362803	Cd164	N/A	N/A
10362823	Snx3	N/A	N/A
10362829	Ostm1	N/A	N/A
10362837	Sec63	N/A	N/A
10362861	Scml4	N/A	N/A
10362874	9030612E09Rik	N/A	N/A
10362876	Pdss2	N/A	N/A
10362886	Bend3	N/A	N/A
10362896	Cd24a	N/A	N/A
10362899	F830002L21Rik	N/A	N/A
10362902	---	N/A	N/A
10362904	Rtn4ip1	N/A	N/A
10362916	Aim1	N/A	N/A
10362922	Atg5	N/A	N/A
10362934	---	N/A	N/A
10362937	---	N/A	N/A
10362939	---	N/A	N/A
10362941	Prep	N/A	N/A
10362968	Bves	N/A	N/A
10363005	LOC280487	N/A	N/A
10363007	Ascc3	N/A	N/A
10363054	---	N/A	N/A
10363056	Sim1	N/A	N/A
10363130	Gopc	N/A	N/A
10363137	Nus1	N/A	N/A
10363144	Gm10674 /// Gm7360	N/A	N/A
10363146	Slc35f1	N/A	N/A
10363157	Pln	N/A	N/A
10363161	Tmem229b /// Gm5423	N/A	N/A
10363163	Asf1a	N/A	N/A
10363170	Msl3l2	N/A	N/A
10363173	Gja1	N/A	N/A
10363178	Npm1	N/A	N/A
10363181	Stard6 /// Gm9795	N/A	N/A
10363185	Gm9956	N/A	N/A
10363195	Hsf2	N/A	N/A
10363224	Fabp7	N/A	N/A
10363231	Smpdl3a	N/A	N/A
10363241	Gcc2	N/A	N/A
10363281	Ranbp2	N/A	N/A
10363331	---	N/A	N/A
10363333	Sh3rf3	N/A	N/A
10363346	Ankrd57	N/A	N/A
10363350	P4ha1	N/A	N/A
10363368	Timm23	N/A	N/A
10363372	Pla2g12b	N/A	N/A
10363377	Gm10322	N/A	N/A
10363379	Cbara1	N/A	N/A
10363392	Dnajb12	N/A	N/A
10363403	Ascc1	N/A	N/A
10363415	Spock2	N/A	N/A
10363430	Psap	N/A	N/A
10363445	4632428N05Rik	N/A	N/A
10363455	Pcbd1	N/A	N/A
10363479	Rpl27a /// Gm5908 /// Rpl27a-ps1 /// Gm6378 /// Gm5453	N/A	N/A
10363481	Nodal	N/A	N/A
10363486	Lrrc20	N/A	N/A
10363498	Ppa1	N/A	N/A
10363512	Sar1a	N/A	N/A
10363522	Tysnd1	N/A	N/A
10363528	Aifm2	N/A	N/A
10363541	Ass1 /// Gm5424	N/A	N/A
10363557	Supv3l1 /// 4930507D05Rik	N/A	N/A
10363559	2510003E04Rik	N/A	N/A
10363561	LOC280487	N/A	N/A
10363563	Slc25a16	N/A	N/A
10363575	Dna2	N/A	N/A
10363599	Rufy2	N/A	N/A
10363622	3110049J23Rik	N/A	N/A
10363629	Pbld	N/A	N/A
10363639	Atoh7	N/A	N/A
10363641	Herc4	N/A	N/A

10363667	Sirt1 /// Gm10114	N/A	N/A
10363696	D630028G08Rik	N/A	N/A
10363699	Rps6 /// Gm16409	N/A	N/A
10363701	---	N/A	N/A
10363703	Jmjd1c	N/A	N/A
10363706	Jmjd1c	N/A	N/A
10363741	Gm10797	N/A	N/A
10363773	Rhobtb1	N/A	N/A
10363786	Ank3	N/A	N/A
10363845	Ccdc6	N/A	N/A
10363860	Slc16a9	N/A	N/A
10363868	Fam13c /// Phyhipl	N/A	N/A
10363894	Ipmk	N/A	N/A
10363901	Etv5	N/A	N/A
10363903	Hbs1l	N/A	N/A
10363905	Zwint	N/A	N/A
10363915	Pcdh15	N/A	N/A
10363917	---	N/A	N/A
10363921	Pcdh15	N/A	N/A
10363970	Rab36	N/A	N/A
10363983	Bcr	N/A	N/A
10364009	Bcr	N/A	N/A
10364011	Specc1l	N/A	N/A
10364030	Adora2a	N/A	N/A
10364038	Upb1	N/A	N/A
10364049	---	N/A	N/A
10364051	Snrpd3	N/A	N/A
10364091	---	N/A	N/A
10364093	Derl3	N/A	N/A
10364102	Chchd10	N/A	N/A
10364109	Vpreb3	N/A	N/A
10364130	Zfp280b	N/A	N/A
10364149	S100b	N/A	N/A
10364153	Pcnt /// Gm9744	N/A	N/A
10364194	Lss	N/A	N/A
10364222	Ftcd	N/A	N/A
10364237	Gm10787	N/A	N/A
10364249	---	N/A	N/A
10364251	Pofut2	N/A	N/A
10364280	Pttg1ip	N/A	N/A
10364293	Ube2g2	N/A	N/A
10364315	LOC16697	N/A	N/A
10364317	---	N/A	N/A
10364319	---	N/A	N/A
10364324	---	N/A	N/A
10364326	Krtap10-10	N/A	N/A
10364328	Gm3238 /// Gm10318	N/A	N/A
10364338	1810043G02Rik	N/A	N/A
10364371	---	N/A	N/A
10364373	Lsm7	N/A	N/A
10364375	Cstb	N/A	N/A
10364379	---	N/A	N/A
10364381	Olfr1358	N/A	N/A
10364383	---	N/A	N/A
10364402	Slc1a6	N/A	N/A
10364413	Olfr1355	N/A	N/A
10364415	Olfr1355 /// Olfr8 /// Olfr1354	N/A	N/A
10364417	Olfr8	N/A	N/A
10364424	Olfr1352	N/A	N/A
10364435	Gm2889	N/A	N/A
10364451	Gm16517	N/A	N/A
10364455	Cdc34	N/A	N/A
10364468	Bsg	N/A	N/A
10364478	Hcn2	N/A	N/A
10364489	Fgf22	N/A	N/A
10364518	Ptbp1	N/A	N/A
10364542	Cfd	N/A	N/A
10364548	---	N/A	N/A
10364550	Kiss1r	N/A	N/A
10364571	Wdr18	N/A	N/A
10364683	Stk11	N/A	N/A
10364696	Atp5d	N/A	N/A
10364712	Cirbp	N/A	N/A
10364718	1600002K03Rik	N/A	N/A
10364766	Rps15	N/A	N/A
10364769	Apc2	N/A	N/A
10364784	Reep6	N/A	N/A
10364810	Onecut3	N/A	N/A
10364814	Scamp4 /// Adat3	N/A	N/A
10364824	Csnk1g2	N/A	N/A
10364856	Dot1l	N/A	N/A
10364888	Dot1l	N/A	N/A
10364890	Sf3a2	N/A	N/A
10364909	Oaz1 /// Gm9786	N/A	N/A

10364950	Gadd45b	N/A	N/A
10364971	Map2k2	N/A	N/A
10364984	Zbtb7a	N/A	N/A
10364990	Eef2	N/A	N/A
10365003	Snord37	N/A	N/A
10365005	Dapk3	N/A	N/A
10365015	Zfr2	N/A	N/A
10365056	Apba3	N/A	N/A
10365069	Pip5k1c	N/A	N/A
10365104	F630110N24Rik	N/A	N/A
10365116	Dohh	N/A	N/A
10365123	Dohh /// LOC100503793	N/A	N/A
10365125	Dohh /// LOC100503793	N/A	N/A
10365127	2210404O07Rik	N/A	N/A
10365132	Nfic	N/A	N/A
10365134	---	N/A	N/A
10365136	Aes	N/A	N/A
10365199	Gm10778	N/A	N/A
10365206	Gm8290	N/A	N/A
10365208	Zfp873	N/A	N/A
10365217	Gm10776	N/A	N/A
10365219	Gm4924	N/A	N/A
10365225	Gm4924	N/A	N/A
10365227	Ap3m1	N/A	N/A
10365230	Gm9855 /// Tdg /// Gm5806	N/A	N/A
10365242	Hcfc2	N/A	N/A
10365260	Txnrd1	N/A	N/A
10365286	Eid3	N/A	N/A
10365288	Taf10 /// Gm4799	N/A	N/A
10365290	Chst11	N/A	N/A
10365295	---	N/A	N/A
10365297	D10Wsu102e	N/A	N/A
10365302	A230046K03Rik	N/A	N/A
10365335	---	N/A	N/A
10365337	---	N/A	N/A
10365344	Tcp11l2	N/A	N/A
10365408	Ric8b	N/A	N/A
10365420	AI597468 /// Mterfd3	N/A	N/A
10365428	Btbd11	N/A	N/A
10365450	Pwp1	N/A	N/A
10365471	Fbxo7	N/A	N/A
10365482	Timp3	N/A	N/A
10365518	Nt5dc3	N/A	N/A
10365535	Tex18	N/A	N/A
10365543	Gm10764	N/A	N/A
10365545	Pah	N/A	N/A
10365559	Igf1	N/A	N/A
10365572	Tyms-ps	N/A	N/A
10365574	Pmch /// 4930547N16Rik	N/A	N/A
10365601	Gnptab	N/A	N/A
10365637	Arl1	N/A	N/A
10365658	Uhrf1bp1l	N/A	N/A
10365682	Anks1b	N/A	N/A
10365714	Sec61g	N/A	N/A
10365716	Ikbip	N/A	N/A
10365723	---	N/A	N/A
10365727	---	N/A	N/A
10365749	Lta4h	N/A	N/A
10365769	Hal	N/A	N/A
10365817	Ntn4	N/A	N/A
10365845	Fgd6	N/A	N/A
10365870	Nr2c1	N/A	N/A
10365887	Ndufa12	N/A	N/A
10365891	Tmcc3	N/A	N/A
10365918	Ube2n	N/A	N/A
10365926	4732465J04Rik	N/A	N/A
10365933	Eea1	N/A	N/A
10365966	Gm5426	N/A	N/A
10365974	Dcn	N/A	N/A
10365983	Lum	N/A	N/A
10366004	Atp2b1	N/A	N/A
10366026	Poc1b	N/A	N/A
10366038	Galnt4	N/A	N/A
10366043	Dusp6	N/A	N/A
10366052	Kitl	N/A	N/A
10366144	Mgat4c	N/A	N/A
10366153	Rassf9	N/A	N/A
10366161	---	N/A	N/A
10366163	Slc6a15	N/A	N/A
10366178	---	N/A	N/A
10366184	---	N/A	N/A
10366186	Ccdc59 /// BC067068	N/A	N/A
10366196	Ppfia2	N/A	N/A
10366229	Lin7a	N/A	N/A

10366238	Ppp1r12a	N/A	N/A
10366275	---	N/A	N/A
10366293	Csrp2	N/A	N/A
10366301	Rpl6 /// Gm6807	N/A	N/A
10366310	Osbpl8	N/A	N/A
10366335	Bbs10	N/A	N/A
10366337	Nap1l1	N/A	N/A
10366344	Gm5176	N/A	N/A
10366346	Phlda1	N/A	N/A
10366350	Krr1 /// Glipr1	N/A	N/A
10366391	Kcnc2	N/A	N/A
10366399	Atxn7l3b	N/A	N/A
10366401	---	N/A	N/A
10366403	---	N/A	N/A
10366405	---	N/A	N/A
10366407	Gm10752	N/A	N/A
10366409	Zfc3h1	N/A	N/A
10366457	Ptprr	N/A	N/A
10366512	---	N/A	N/A
10366514	Kcnmb4 /// 1700058G18Rik	N/A	N/A
10366517	Kcnmb4	N/A	N/A
10366519	Cnot2	N/A	N/A
10366522	Rpl29	N/A	N/A
10366539	Gm10747	N/A	N/A
10366544	Kifc5c-ps	N/A	N/A
10366546	Cpm	N/A	N/A
10366579	Cdc5l	N/A	N/A
10366581	Cdc5l	N/A	N/A
10366593	---	N/A	N/A
10366595	Dyrk2 /// 4932442E05Rik	N/A	N/A
10366630	Tmbim4	N/A	N/A
10366665	Rpl21 /// Gm16416 /// Gm8054	N/A	N/A
10366667	Gns	N/A	N/A
10366698	BC048403	N/A	N/A
10366705	---	N/A	N/A
10366712	Ppm1h	N/A	N/A
10366725	Fam19a2	N/A	N/A
10366735	4930503E24Rik	N/A	N/A
10366737	---	N/A	N/A
10366767	Ctdsp2 /// Mir26a-2	N/A	N/A
10366814	Cdk4	N/A	N/A
10366825	Agap2	N/A	N/A
10366846	Os9	N/A	N/A
10366864	F420014N23Rik	N/A	N/A
10366866	Dctn2	N/A	N/A
10366881	Ddit3 /// Mars	N/A	N/A
10366909	R3hdm2	N/A	N/A
10366956	Stat6	N/A	N/A
10366983	Tmem194	N/A	N/A
10367024	Tac2	N/A	N/A
10367033	Zbtb39	N/A	N/A
10367041	Rdh9	N/A	N/A
10367045	Rdh16	N/A	N/A
10367059	BC089597	N/A	N/A
10367066	Sdr9c7	N/A	N/A
10367073	Snrpd2 /// Gm5449 /// Gm10120	N/A	N/A
10367100	Ptges3	N/A	N/A
10367106	Atp5b	N/A	N/A
10367118	---	N/A	N/A
10367120	Mir677	N/A	N/A
10367122	Baz2a	N/A	N/A
10367154	Gls2	N/A	N/A
10367215	Apon	N/A	N/A
10367224	Stat2	N/A	N/A
10367292	Cs	N/A	N/A
10367305	Ankrd52	N/A	N/A
10367335	---	N/A	N/A
10367337	Rnf41	N/A	N/A
10367349	Smarcc2	N/A	N/A
10367422	Sarnp	N/A	N/A
10367434	---	N/A	N/A
10367436	Cd63	N/A	N/A
10367471	Olfr9	N/A	N/A
10367473	Olfr763	N/A	N/A
10367475	---	N/A	N/A
10367477	Olfr774	N/A	N/A
10367479	Olfr775	N/A	N/A
10367483	Olfr804 /// Olfr782 /// Olfr779	N/A	N/A
10367485	Olfr780	N/A	N/A
10367489	Olfr804 /// Olfr782 /// Olfr779	N/A	N/A
10367493	Olfr787	N/A	N/A
10367495	Olfr786	N/A	N/A
10367499	Olfr788	N/A	N/A
10367501	---	N/A	N/A

10367503	Olfr790	N/A	N/A
10367505	Olfr791	N/A	N/A
10367509	Olfr794	N/A	N/A
10367511	Olfr799	N/A	N/A
10367515	---	N/A	N/A
10367524	Olfr813	N/A	N/A
10367528	Olfr821	N/A	N/A
10367530	Olfr822	N/A	N/A
10367544	Ctdsp2	N/A	N/A
10367579	Gm3213	N/A	N/A
10367582	Vip	N/A	N/A
10367591	Myct1	N/A	N/A
10367598	Gm10097	N/A	N/A
10367600	Esr1	N/A	N/A
10367624	---	N/A	N/A
10367671	---	N/A	N/A
10367691	Iyd	N/A	N/A
10367697	Ppp1r14c	N/A	N/A
10367708	Pcmt1	N/A	N/A
10367717	BC013529	N/A	N/A
10367726	6530403G13Rik	N/A	N/A
10367728	Tab2	N/A	N/A
10367734	Ust	N/A	N/A
10367744	---	N/A	N/A
10367746	Sash1	N/A	N/A
10367768	---	N/A	N/A
10367770	Gm9930	N/A	N/A
10367772	Samd5	N/A	N/A
10367775	Stxbp5	N/A	N/A
10367803	---	N/A	N/A
10367816	9130014G24Rik	N/A	N/A
10367822	Rab32	N/A	N/A
10367828	Grm1 /// Gm10828	N/A	N/A
10367830	Grm1	N/A	N/A
10367843	Utrn	N/A	N/A
10367945	Phactr2	N/A	N/A
10367960	Pex3	N/A	N/A
10367973	Aig1	N/A	N/A
10368011	Vta1	N/A	N/A
10368025	Hspa8 /// LOC624853	N/A	N/A
10368027	Nmbr	N/A	N/A
10368041	3110003A17Rik	N/A	N/A
10368045	3110003A17Rik	N/A	N/A
10368050	Ect2l /// LOC100045792	N/A	N/A
10368052	Ect2l /// LOC100045792	N/A	N/A
10368054	Ect2l /// LOC100045792	N/A	N/A
10368056	Ect2l /// LOC100045792	N/A	N/A
10368058	Ect2l /// LOC100045792	N/A	N/A
10368060	Ect2l /// LOC100045792	N/A	N/A
10368062	Ect2l /// LOC100045792	N/A	N/A
10368064	Ect2l /// LOC100045792	N/A	N/A
10368066	Ect2l /// LOC100045792	N/A	N/A
10368068	Ect2l /// LOC100045792	N/A	N/A
10368075	Ect2l /// LOC100045792	N/A	N/A
10368077	Ect2l /// LOC100045792	N/A	N/A
10368079	Ect2l /// LOC100045792	N/A	N/A
10368083	Ccdc28a	N/A	N/A
10368090	---	N/A	N/A
10368101	D10Bwg1379e	N/A	N/A
10368159	Slc35d3	N/A	N/A
10368162	Pex7	N/A	N/A
10368173	4933406P04Rik	N/A	N/A
10368175	Pde7b	N/A	N/A
10368193	---	N/A	N/A
10368197	Gm10826	N/A	N/A
10368220	---	N/A	N/A
10368222	4930444G20Rik	N/A	N/A
10368227	Ube2q2	N/A	N/A
10368229	Tbpl1 /// Slc2a12	N/A	N/A
10368240	Tcf21	N/A	N/A
10368268	---	N/A	N/A
10368277	Rps12	N/A	N/A
10368279	Taar6	N/A	N/A
10368281	Taar7e /// Taar7f /// Taar7b /// Taar7a /// Taar7d	N/A	N/A
10368287	Taar9	N/A	N/A
10368289	Enpp1	N/A	N/A
10368317	Enpp3	N/A	N/A
10368343	Arg1	N/A	N/A
10368356	Akap7	N/A	N/A
10368370	---	N/A	N/A
10368380	L3mbtl3	N/A	N/A
10368473	---	N/A	N/A
10368475	4930519F09Rik	N/A	N/A
10368484	Gm9996	N/A	N/A

10368486	Rnf146 /// Echdc1	N/A	N/A
10368495	Rspo3	N/A	N/A
10368504	Rpl12	N/A	N/A
10368527	Hint3	N/A	N/A
10368566	Tpd52l1	N/A	N/A
10368575	Supt4h1 /// Gm3258	N/A	N/A
10368585	Nkain2	N/A	N/A
10368596	---	N/A	N/A
10368598	Clvs2	N/A	N/A
10368612	Gapdh	N/A	N/A
10368616	Zufsp	N/A	N/A
10368644	Fam26f	N/A	N/A
10368647	Dse	N/A	N/A
10368675	Marcks	N/A	N/A
10368681	Rfpl4b	N/A	N/A
10368683	1700025K23Rik	N/A	N/A
10368689	Wisp3	N/A	N/A
10368700	G630090E17Rik	N/A	N/A
10368711	---	N/A	N/A
10368713	AI317395	N/A	N/A
10368720	Slc16a10	N/A	N/A
10368730	Rpf2	N/A	N/A
10368739	Gtf3c6	N/A	N/A
10368762	Cdc40	N/A	N/A
10368780	Gpr6	N/A	N/A
10368782	Fig4	N/A	N/A
10368806	Smpd2	N/A	N/A
10368836	---	N/A	N/A
10368875	Armc2	N/A	N/A
10368877	Armc2	N/A	N/A
10368881	Armc2	N/A	N/A
10368883	Gm9855 /// Tdg /// Gm5806	N/A	N/A
10368886	Foxo3	N/A	N/A
10368888	Foxo3	N/A	N/A
10368893	Lace1	N/A	N/A
10368907	Nr2e1	N/A	N/A
10368918	Sobp	N/A	N/A
10368928	Rpl41	N/A	N/A
10368930	1700021F05Rik	N/A	N/A
10368935	Qrsl1	N/A	N/A
10368947	Aim1	N/A	N/A
10368979	---	N/A	N/A
10368981	Lin28b	N/A	N/A
10368990	---	N/A	N/A
10368992	---	N/A	N/A
10368997	C130030K03Rik	N/A	N/A
10368999	Grik2	N/A	N/A
10369086	Gopc	N/A	N/A
10369102	Gm9766	N/A	N/A
10369132	Fam184a	N/A	N/A
10369154	Man1a	N/A	N/A
10369171	9530009G21Rik	N/A	N/A
10369176	D630037F22Rik	N/A	N/A
10369206	Rps8 /// Gm11353 /// Rps8-ps1	N/A	N/A
10369210	Serinc1	N/A	N/A
10369221	4933403O03Rik /// Dux	N/A	N/A
10369223	4933403O03Rik /// Dux	N/A	N/A
10369225	4933403O03Rik /// Dux	N/A	N/A
10369232	Gm9919	N/A	N/A
10369234	---	N/A	N/A
10369250	---	N/A	N/A
10369264	Oit3	N/A	N/A
10369288	---	N/A	N/A
10369290	Ddit4	N/A	N/A
10369295	Anapc16	N/A	N/A
10369379	Slc29a3	N/A	N/A
10369388	Unc5b	N/A	N/A
10369413	Sgpl1	N/A	N/A
10369474	Eif4ebp2	N/A	N/A
10369479	Lrrc20 /// D830039M14Rik	N/A	N/A
10369481	H2afy2	N/A	N/A
10369525	2010107G23Rik	N/A	N/A
10369531	Tspan15	N/A	N/A
10369586	Supv3l1 /// 4930507D05Rik	N/A	N/A
10369604	Vps26a	N/A	N/A
10369620	2510003E04Rik	N/A	N/A
10369630	Ddx21	N/A	N/A
10369647	Ddx50	N/A	N/A
10369661	Ccar1	N/A	N/A
10369688	Tet1	N/A	N/A
10369690	Tet1	N/A	N/A
10369702	Tet1	N/A	N/A
10369704	Hnrnph3	N/A	N/A
10369735	Herc4 /// 1700120B22Rik	N/A	N/A

10369738	Sirt1	N/A	N/A
10369748	Gm7075	N/A	N/A
10369750	Gm10118	N/A	N/A
10369752	Lrrtm3	N/A	N/A
10369761	Reep3	N/A	N/A
10369774	Nrbf2	N/A	N/A
10369783	Zfp365	N/A	N/A
10369792	Arid5b	N/A	N/A
10369806	1700040L02Rik	N/A	N/A
10369825	---	N/A	N/A
10369828	---	N/A	N/A
10369835	Fam13c /// Phyhipl	N/A	N/A
10369842	Bicc1	N/A	N/A
10369867	Tfam	N/A	N/A
10369877	Ube2d1	N/A	N/A
10369885	Cisd1	N/A	N/A
10369890	---	N/A	N/A
10369892	---	N/A	N/A
10369894	1700049L16Rik	N/A	N/A
10369898	---	N/A	N/A
10369909	---	N/A	N/A
10369911	1110038D17Rik	N/A	N/A
10369920	AI646023	N/A	N/A
10369927	---	N/A	N/A
10369929	---	N/A	N/A
10369932	Susd2	N/A	N/A
10369948	Cabin1	N/A	N/A
10369989	Ddt	N/A	N/A
10369993	Gstt3	N/A	N/A
10370013	Gstt2	N/A	N/A
10370021	Mif	N/A	N/A
10370025	Smarcb1	N/A	N/A
10370037	Mmp11	N/A	N/A
10370070	---	N/A	N/A
10370072	Prmt2 /// Mir678	N/A	N/A
10370174	A130042E20Rik /// 2610028H24Rik /// Mcm3ap	N/A	N/A
10370242	Pcbp3	N/A	N/A
10370303	Adarb1	N/A	N/A
10370334	Lrrc3	N/A	N/A
10370376	Pfkl	N/A	N/A
10370413	D10Jhu81e	N/A	N/A
10370422	Pwp2	N/A	N/A
10370446	Trappc10	N/A	N/A
10370483	Rrp1	N/A	N/A
10370497	Pdxk	N/A	N/A
10370508	Gm9978	N/A	N/A
10370544	2610008E11Rik	N/A	N/A
10370552	Ppap2c	N/A	N/A
10370559	Mier2	N/A	N/A
10370584	C2cd4c	N/A	N/A
10370632	Rnf126	N/A	N/A
10370651	BC005764	N/A	N/A
10370681	C030046I01Rik	N/A	N/A
10370754	Dos	N/A	N/A
10370777	2310011J03Rik	N/A	N/A
10370818	Mex3d	N/A	N/A
10370824	Mbd3	N/A	N/A
10370833	Uqcr11	N/A	N/A
10370892	Rexo1	N/A	N/A
10370911	Klf16	N/A	N/A
10370914	Fam108a	N/A	N/A
10370920	Btbd2	N/A	N/A
10370931	Mknk2	N/A	N/A
10370946	Mobkl2a	N/A	N/A
10370950	Ap3d1	N/A	N/A
10370983	Plekhj1 /// Sf3a2	N/A	N/A
10370999	Lingo3	N/A	N/A
10371002	Lsm7	N/A	N/A
10371006	Timm13	N/A	N/A
10371037	Slc39a3	N/A	N/A
10371041	Sgta	N/A	N/A
10371067	Pias4	N/A	N/A
10371080	Dapk3 /// 4930442H23Rik	N/A	N/A
10371092	Atcay	N/A	N/A
10371107	Mrpl54	N/A	N/A
10371141	Hmg20b	N/A	N/A
10371159	Fzr1	N/A	N/A
10371176	Nfic	N/A	N/A
10371188	Celf5	N/A	N/A
10371201	Ncln	N/A	N/A
10371217	S1pr4	N/A	N/A
10371230	Gna11	N/A	N/A
10371256	Sirt6	N/A	N/A
10371271	Zfp781	N/A	N/A

10371277	B230315N10Rik	N/A	N/A
10371286	---	N/A	N/A
10371293	1190007I07Rik	N/A	N/A
10371296	Glt8d2 /// Tdg	N/A	N/A
10371307	Nfyb	N/A	N/A
10371319	Gm10773	N/A	N/A
10371332	Aldh1l2	N/A	N/A
10371356	Appl2	N/A	N/A
10371379	Nuak1	N/A	N/A
10371387	Ckap4	N/A	N/A
10371396	Mterfd3	N/A	N/A
10371400	Cry1	N/A	N/A
10371420	Prdm4	N/A	N/A
10371432	D10Wsu52e	N/A	N/A
10371464	Fbxo7	N/A	N/A
10371466	Syn3	N/A	N/A
10371482	Hsp90b1	N/A	N/A
10371578	Ascl1	N/A	N/A
10371589	Gm10763	N/A	N/A
10371607	Dram1	N/A	N/A
10371616	Chpt1	N/A	N/A
10371660	---	N/A	N/A
10371670	---	N/A	N/A
10371676	Utp20	N/A	N/A
10371770	Gas2l3	N/A	N/A
10371784	Nr1h4	N/A	N/A
10371811	Scyl2	N/A	N/A
10371842	Uhrf1bp1l	N/A	N/A
10371844	---	N/A	N/A
10371877	Slc25a3	N/A	N/A
10371888	Tmpo	N/A	N/A
10371904	1110012L19Rik	N/A	N/A
10371916	4930485B16Rik	N/A	N/A
10371942	4930485B16Rik	N/A	N/A
10371959	Cdk17 /// Elk3	N/A	N/A
10371970	Amdhd1	N/A	N/A
10372001	---	N/A	N/A
10372003	Mir331	N/A	N/A
10372005	Vezt	N/A	N/A
10372021	---	N/A	N/A
10372028	Plxnc1	N/A	N/A
10372060	Cradd	N/A	N/A
10372078	Mrpl42	N/A	N/A
10372082	Nudt4	N/A	N/A
10372091	3200002M19Rik /// Gm6843	N/A	N/A
10372094	---	N/A	N/A
10372102	LOC100504157	N/A	N/A
10372104	---	N/A	N/A
10372106	Gm10754 /// Epyc	N/A	N/A
10372108	C030005K15Rik	N/A	N/A
10372110	Phxr2	N/A	N/A
10372114	Gm8671	N/A	N/A
10372121	Tmtc3	N/A	N/A
10372130	4930430F08Rik /// Cep290	N/A	N/A
10372139	Nts	N/A	N/A
10372151	Lrriq1	N/A	N/A
10372177	Tmtc2	N/A	N/A
10372204	---	N/A	N/A
10372208	Acss3	N/A	N/A
10372324	Syt1	N/A	N/A
10372338	Gm5136	N/A	N/A
10372342	Nav3	N/A	N/A
10372383	Zdhhc17	N/A	N/A
10372385	Zdhhc17	N/A	N/A
10372417	Atxn7l3b	N/A	N/A
10372421	Trhde	N/A	N/A
10372441	---	N/A	N/A
10372457	Tbc1d15	N/A	N/A
10372478	Rab21	N/A	N/A
10372488	Tmem19	N/A	N/A
10372497	Thap2	N/A	N/A
10372503	Lgr5	N/A	N/A
10372534	Cnot2	N/A	N/A
10372583	Rab3ip	N/A	N/A
10372597	---	N/A	N/A
10372600	Cct2	N/A	N/A
10372618	Frs2	N/A	N/A
10372629	Yeats4	N/A	N/A
10372648	Lyz2	N/A	N/A
10372656	Cpsf6	N/A	N/A
10372716	Rap1b	N/A	N/A
10372726	Cdc5l	N/A	N/A
10372728	Cdc5l	N/A	N/A
10372733	Cdc5l	N/A	N/A

10372735	Cdc5l	N/A	N/A
10372737	Cdc5l	N/A	N/A
10372739	Cdc5l	N/A	N/A
10372741	Cdc5l	N/A	N/A
10372743	Cdc5l	N/A	N/A
10372745	Dyrk2	N/A	N/A
10372748	Gm10744	N/A	N/A
10372750	Cand1	N/A	N/A
10372805	Gm10743	N/A	N/A
10372815	Lemd3	N/A	N/A
10372831	Tbc1d30	N/A	N/A
10372844	Rassf3	N/A	N/A
10372853	Tbk1	N/A	N/A
10372877	Xpot	N/A	N/A
10372889	BC048403	N/A	N/A
10372891	Srgap1	N/A	N/A
10372917	Tmem5 /// Gm9079	N/A	N/A
10372926	Mirlet7i	N/A	N/A
10372965	Usp15	N/A	N/A
10372988	Slc16a7	N/A	N/A
10373000	Xrcc6bp1	N/A	N/A
10373009	Tsfm	N/A	N/A
10373021	March9	N/A	N/A
10373027	Tspan31 /// Cdk4	N/A	N/A
10373036	Os9	N/A	N/A
10373052	B4galnt1	N/A	N/A
10373093	Dtx3	N/A	N/A
10373101	Pip4k2c	N/A	N/A
10373113	Kif5a	N/A	N/A
10373143	Mbd6	N/A	N/A
10373157	Mars	N/A	N/A
10373179	Gli1	N/A	N/A
10373192	Inhbe	N/A	N/A
10373197	Inhbc	N/A	N/A
10373202	Shmt2	N/A	N/A
10373223	Lrp1	N/A	N/A
10373313	Nab2	N/A	N/A
10373334	Hsd17b6	N/A	N/A
10373340	Rbms2	N/A	N/A
10373355	Spryd4	N/A	N/A
10373365	Pan2	N/A	N/A
10373367	Coq10a	N/A	N/A
10373372	Coq10a	N/A	N/A
10373388	Obfc2b	N/A	N/A
10373396	Myl6	N/A	N/A
10373400	Myl6b	N/A	N/A
10373439	Zc3h10	N/A	N/A
10373454	Pa2g4	N/A	N/A
10373467	Erbb3	N/A	N/A
10373498	Rps26	N/A	N/A
10373502	Ikzf4	N/A	N/A
10373515	Suox	N/A	N/A
10373519	Rpl12	N/A	N/A
10373521	Rab5b	N/A	N/A
10373542	Dgka	N/A	N/A
10373569	Rpsa /// Rpsa-ps10	N/A	N/A
10373571	1110012D08Rik	N/A	N/A
10373577	Ormdl2 /// Dnajc14	N/A	N/A
10373583	Gdf11	N/A	N/A
10373588	Rdh5	N/A	N/A
10373594	Bloc1s1	N/A	N/A
10373600	Mettl7b	N/A	N/A
10373606	Olfr765	N/A	N/A
10373610	Olfr767	N/A	N/A
10373612	Olfr768	N/A	N/A
10373614	Olfr769	N/A	N/A
10373616	Olfr770	N/A	N/A
10373618	Olfr771	N/A	N/A
10373622	Olfr772 /// Olfr773	N/A	N/A
10373624	Olfr777 /// Olfr800	N/A	N/A
10373626	Olfr796	N/A	N/A
10373630	Olfr801	N/A	N/A
10373632	Olfr802	N/A	N/A
10373636	Olfr805	N/A	N/A
10373640	Olfr807	N/A	N/A
10373645	Olfr811	N/A	N/A
10373647	Olfr812	N/A	N/A
10373651	---	N/A	N/A
10373657	---	N/A	N/A
10373660	Olfr818	N/A	N/A
10373662	Olfr819 /// Olfr247	N/A	N/A
10373664	Olfr819 /// Olfr247	N/A	N/A
10373666	Olfr819 /// Olfr247	N/A	N/A
10373668	Olfr823 /// Olfr824	N/A	N/A

10373670	Olfr823 /// Olfr824	N/A	N/A
10373678	Olfr827	N/A	N/A
10373684	Vmn2r123	N/A	N/A
10373690	---	N/A	N/A
10373692	Vmn2r86 /// Vmn2r87	N/A	N/A
10373702	Pisd-ps3 /// Pisd-ps1	N/A	N/A
10373709	Eif4enif1	N/A	N/A
10373740	Pik3ip1	N/A	N/A
10373778	Morc2a	N/A	N/A
10373810	Dusp18	N/A	N/A
10373814	Pes1	N/A	N/A
10373832	---	N/A	N/A
10373834	Sec14l4	N/A	N/A
10373861	Rnf215	N/A	N/A
10373873	Sf3a1	N/A	N/A
10373890	Tbc1d10a	N/A	N/A
10373902	Gatsl3	N/A	N/A
10373924	Ascc2	N/A	N/A
10373944	Zmat5	N/A	N/A
10373950	Nipsnap1	N/A	N/A
10373964	Thoc5	N/A	N/A
10373986	Ap1b1 /// Nefh	N/A	N/A
10374035	Xbp1	N/A	N/A
10374068	Dbnl	N/A	N/A
10374083	Aebp1	N/A	N/A
10374115	Nudcd3	N/A	N/A
10374117	---	N/A	N/A
10374119	Ogdh	N/A	N/A
10374151	Zmiz2	N/A	N/A
10374175	Ppia	N/A	N/A
10374181	---	N/A	N/A
10374183	LOC280487	N/A	N/A
10374185	Ccm2	N/A	N/A
10374223	Igfbp1	N/A	N/A
10374228	Gm11992	N/A	N/A
10374313	---	N/A	N/A
10374348	---	N/A	N/A
10374350	---	N/A	N/A
10374352	LOC280487	N/A	N/A
10374354	---	N/A	N/A
10374356	Vstm2a	N/A	N/A
10374364	Akt2	N/A	N/A
10374366	Egfr	N/A	N/A
10374406	Cnrip1 /// Plek	N/A	N/A
10374413	---	N/A	N/A
10374415	Ppp3r1	N/A	N/A
10374426	Pno1	N/A	N/A
10374430	Wdr92	N/A	N/A
10374442	C1d	N/A	N/A
10374448	---	N/A	N/A
10374450	---	N/A	N/A
10374453	Glul	N/A	N/A
10374455	Spred2	N/A	N/A
10374464	Spred2	N/A	N/A
10374466	Rab1	N/A	N/A
10374476	Rps17 /// Gm12034	N/A	N/A
10374485	Peli1	N/A	N/A
10374529	AV249152	N/A	N/A
10374553	Tmem17	N/A	N/A
10374558	---	N/A	N/A
10374560	Zrsr1	N/A	N/A
10374564	Cct4	N/A	N/A
10374590	Xpo1	N/A	N/A
10374621	Usp34 /// Ahsa2	N/A	N/A
10374706	Pus10	N/A	N/A
10374727	Bcl11a	N/A	N/A
10374744	---	N/A	N/A
10374762	Vrk2 /// Gm6899	N/A	N/A
10374767	---	N/A	N/A
10374769	---	N/A	N/A
10374771	Mir216b	N/A	N/A
10374773	Mir216a	N/A	N/A
10374775	Mir217	N/A	N/A
10374777	Efemp1	N/A	N/A
10374793	Pnpt1	N/A	N/A
10374821	Smek2	N/A	N/A
10374840	---	N/A	N/A
10374842	Ccdc88a	N/A	N/A
10374880	Mtif2	N/A	N/A
10374908	Rtn4	N/A	N/A
10374929	4931440F15Rik	N/A	N/A
10374934	Psme4	N/A	N/A
10374998	Gpr75	N/A	N/A
10375038	Mpg /// Nprl3	N/A	N/A

10375051	Hba-a1 /// Hba-a2	N/A	N/A
10375058	Hba-a1 /// Hba-a2	N/A	N/A
10375065	Sh3pxd2b	N/A	N/A
10375079	Ubtd2	N/A	N/A
10375103	Fbxw11	N/A	N/A
10375121	C530030P08Rik	N/A	N/A
10375123	C530030P08Rik	N/A	N/A
10375125	Ssbp1	N/A	N/A
10375127	1700072H12Rik	N/A	N/A
10375145	Lcp2	N/A	N/A
10375167	Fam196b	N/A	N/A
10375214	Mir218-2	N/A	N/A
10375216	Pank3	N/A	N/A
10375225	Mir103-1	N/A	N/A
10375227	---	N/A	N/A
10375229	---	N/A	N/A
10375232	---	N/A	N/A
10375234	Nudcd2	N/A	N/A
10375240	Hspd1	N/A	N/A
10375242	---	N/A	N/A
10375245	Gabrb2	N/A	N/A
10375259	Gabrb2	N/A	N/A
10375261	Gabrb2	N/A	N/A
10375290	Slu7	N/A	N/A
10375322	4933415A04Rik	N/A	N/A
10375324	LOC280487	N/A	N/A
10375326	Pwwp2a	N/A	N/A
10375343	Rnf145	N/A	N/A
10375358	Ebf1	N/A	N/A
10375360	Ebf1	N/A	N/A
10375382	Clint1	N/A	N/A
10375432	C030019I05Rik /// Cyfip2	N/A	N/A
10375439	Med7	N/A	N/A
10375472	Timd4	N/A	N/A
10375483	---	N/A	N/A
10375485	---	N/A	N/A
10375487	Gnb2l1	N/A	N/A
10375497	---	N/A	N/A
10375499	Snord96a	N/A	N/A
10375501	Snord95	N/A	N/A
10375525	Olfr1395	N/A	N/A
10375527	Olfr1394	N/A	N/A
10375529	Zfp62	N/A	N/A
10375543	Olfr1393	N/A	N/A
10375545	Olfr1392	N/A	N/A
10375547	Olfr10	N/A	N/A
10375549	Olfr1391	N/A	N/A
10375551	Olfr1390	N/A	N/A
10375557	Olfr1388	N/A	N/A
10375559	---	N/A	N/A
10375561	Olfr1387	N/A	N/A
10375563	Olfr1393 /// Olfr1392 /// Olfr1386 /// Olfr1385	N/A	N/A
10375565	Olfr1392	N/A	N/A
10375570	Olfr1383	N/A	N/A
10375574	Olfr1373 /// Olfr1371 /// Olfr1380 /// Olfr1382 /// Olfr1381	N/A	N/A
10375576	Olfr1373 /// Olfr1371 /// Olfr1380 /// Olfr1382 /// Olfr1381	N/A	N/A
10375634	Mapk9	N/A	N/A
10375667	Rnf130	N/A	N/A
10375677	Mir340	N/A	N/A
10375679	Tbc1d9b	N/A	N/A
10375704	Sqstm1	N/A	N/A
10375713	Mgat4b	N/A	N/A
10375730	---	N/A	N/A
10375732	Cby3	N/A	N/A
10375735	Hnrnph1	N/A	N/A
10375787	Olfr1378	N/A	N/A
10375791	Olfr1378 /// Olfr51 /// Olfr351	N/A	N/A
10375793	Olfr51	N/A	N/A
10375795	Olfr54	N/A	N/A
10375797	Olfr54	N/A	N/A
10375799	Zfp354a	N/A	N/A
10375811	---	N/A	N/A
10375815	---	N/A	N/A
10375820	Clk4	N/A	N/A
10375864	Agxt2l2 /// Hnrnpab	N/A	N/A
10375880	Nhp2	N/A	N/A
10375893	Sar1b	N/A	N/A
10375901	---	N/A	N/A
10375903	Cdkn2aipnl	N/A	N/A
10375909	Cdkl3	N/A	N/A
10375926	Ppp2ca	N/A	N/A
10375941	Vdac1	N/A	N/A
10375956	Fstl4	N/A	N/A
10375973	Taf13	N/A	N/A

10375980	Aff4	N/A	N/A
10376002	---	N/A	N/A
10376007	Shroom1	N/A	N/A
10376017	Ankrd43 /// Gm9945	N/A	N/A
10376021	Sep 08	N/A	N/A
10376033	Kif3a	N/A	N/A
10376052	---	N/A	N/A
10376060	Irf1	N/A	N/A
10376074	P4ha2	N/A	N/A
10376092	---	N/A	N/A
10376142	3230401D17Rik	N/A	N/A
10376144	Fnip1	N/A	N/A
10376163	Rapgef6	N/A	N/A
10376201	Gpx3	N/A	N/A
10376208	Gm2a	N/A	N/A
10376216	Slc36a1	N/A	N/A
10376239	Gm12238	N/A	N/A
10376241	Zic5	N/A	N/A
10376245	Gria1	N/A	N/A
10376263	Mfap3	N/A	N/A
10376269	Galnt10	N/A	N/A
10376283	---	N/A	N/A
10376285	Sap30l	N/A	N/A
10376292	Larp1	N/A	N/A
10376312	Larp1	N/A	N/A
10376314	Cnot8	N/A	N/A
10376324	Gm12250	N/A	N/A
10376326	Igtp /// Irgm2	N/A	N/A
10376339	Zfp692-ps	N/A	N/A
10376358	Sh3bp5l	N/A	N/A
10376374	---	N/A	N/A
10376392	Olfr325	N/A	N/A
10376394	Olfr324	N/A	N/A
10376404	Olfr320	N/A	N/A
10376406	Olfr319	N/A	N/A
10376408	Olfr316	N/A	N/A
10376410	Olfr315	N/A	N/A
10376412	Olfr314	N/A	N/A
10376414	Olfr313	N/A	N/A
10376416	Olfr312 /// Olfr313	N/A	N/A
10376418	Olfr311	N/A	N/A
10376420	Gm12258	N/A	N/A
10376425	Gm12258	N/A	N/A
10376427	2810021J22Rik	N/A	N/A
10376444	Hist3h2bb-ps /// Hist3h2ba	N/A	N/A
10376446	Trim17 /// Hist3h2a	N/A	N/A
10376455	Hist3h2a /// Trim17	N/A	N/A
10376461	Trim11	N/A	N/A
10376474	Mrpl55	N/A	N/A
10376482	2310033P09Rik	N/A	N/A
10376496	Jmjd4	N/A	N/A
10376532	Olfr225	N/A	N/A
10376534	Mprip	N/A	N/A
10376555	Cops3	N/A	N/A
10376557	Nt5m	N/A	N/A
10376564	Med9	N/A	N/A
10376577	---	N/A	N/A
10376579	Lrrc48	N/A	N/A
10376596	4933439F18Rik	N/A	N/A
10376685	Alkbh5	N/A	N/A
10376715	Smcr7	N/A	N/A
10376721	Smcr8	N/A	N/A
10376726	Dhrs7b	N/A	N/A
10376733	Map2k3	N/A	N/A
10376839	Ttc19	N/A	N/A
10376864	Ubb /// Gm1821	N/A	N/A
10376885	Snord49b	N/A	N/A
10376887	Snord49a	N/A	N/A
10376897	---	N/A	N/A
10376929	Fam18b	N/A	N/A
10376950	Pmp22	N/A	N/A
10376956	Hs3st3a1	N/A	N/A
10376986	AU040829	N/A	N/A
10376998	Pirt	N/A	N/A
10377010	Sco1	N/A	N/A
10377215	Gas7	N/A	N/A
10377253	---	N/A	N/A
10377255	Stx8	N/A	N/A
10377308	Mfsd6l	N/A	N/A
10377319	Myh10	N/A	N/A
10377380	1500010J02Rik	N/A	N/A
10377416	2310047M10Rik	N/A	N/A
10377418	Tmem107	N/A	N/A
10377429	Snord118	N/A	N/A

10377431	Vamp2	N/A	N/A
10377508	Trappc1	N/A	N/A
10377534	A030009H04Rik	N/A	N/A
10377537	Chd3	N/A	N/A
10377541	Lsmd1 /// Tmem88	N/A	N/A
10377547	Kdm6b	N/A	N/A
10377550	Trp53	N/A	N/A
10377560	Sat2	N/A	N/A
10377569	Fxr2	N/A	N/A
10377593	Zbtb4	N/A	N/A
10377603	Tmem102 /// G630025P09Rik	N/A	N/A
10377605	1810027O10Rik	N/A	N/A
10377681	Dullard /// Rai12	N/A	N/A
10377695	Phf23	N/A	N/A
10377751	Asgr1	N/A	N/A
10377763	Asgr2	N/A	N/A
10377802	Mir195	N/A	N/A
10377822	Med11	N/A	N/A
10377841	Tm4sf5	N/A	N/A
10377847	Gltpd2	N/A	N/A
10377889	Mink1	N/A	N/A
10377927	Rnf167	N/A	N/A
10377953	Kif1c	N/A	N/A
10377982	Kif1c	N/A	N/A
10377984	Zfp3	N/A	N/A
10377987	Rabep1	N/A	N/A
10378013	Rpain	N/A	N/A
10378024	Mis12	N/A	N/A
10378038	Wscd1	N/A	N/A
10378059	Txndc17	N/A	N/A
10378065	Med31 /// 4930563E22Rik	N/A	N/A
10378068	Xaf1	N/A	N/A
10378082	Ggt6	N/A	N/A
10378088	Mybbp1a	N/A	N/A
10378114	Ube2g1	N/A	N/A
10378126	Ankfy1	N/A	N/A
10378154	Zzef1	N/A	N/A
10378214	---	N/A	N/A
10378253	Camkk1	N/A	N/A
10378271	1200014J11Rik	N/A	N/A
10378334	Tax1bp3	N/A	N/A
10378341	Shpk	N/A	N/A
10378385	Spata22 /// Olfr20	N/A	N/A
10378395	Olfr20	N/A	N/A
10378397	Olfr376	N/A	N/A
10378399	Olfr384	N/A	N/A
10378401	---	N/A	N/A
10378407	Olfr390	N/A	N/A
10378415	---	N/A	N/A
10378427	Olfr401	N/A	N/A
10378429	Olfr402	N/A	N/A
10378432	Olfr403 /// Olfr43	N/A	N/A
10378434	Olfr59 /// Olfr406-ps	N/A	N/A
10378436	Olfr59	N/A	N/A
10378438	---	N/A	N/A
10378443	Olfr412	N/A	N/A
10378445	E130309D14Rik	N/A	N/A
10378453	1300001I01Rik	N/A	N/A
10378482	Mett10d	N/A	N/A
10378497	Mnt	N/A	N/A
10378508	Tsr1 /// Srr	N/A	N/A
10378523	Smg6	N/A	N/A
10378547	Mir132	N/A	N/A
10378568	Mir22 /// 2210403K04Rik	N/A	N/A
10378570	---	N/A	N/A
10378572	Tlcd2	N/A	N/A
10378579	Prpf8	N/A	N/A
10378668	Pitpna	N/A	N/A
10378681	Inpp5k	N/A	N/A
10378732	Crk	N/A	N/A
10378739	Ywhae	N/A	N/A
10378749	1700016K19Rik	N/A	N/A
10378763	Gemin4	N/A	N/A
10378774	Timm22	N/A	N/A
10378781	---	N/A	N/A
10378783	Rpl36 /// Gm13611	N/A	N/A
10378785	Bhlha9	N/A	N/A
10378833	Ssh2	N/A	N/A
10378848	Hsp90aa1	N/A	N/A
10378857	Coro6	N/A	N/A
10378870	Git1	N/A	N/A
10378964	Sez6	N/A	N/A
10378988	Phf12	N/A	N/A
10379006	Dhrs13	N/A	N/A

10379013	Flot2	N/A	N/A
10379028	Mir451	N/A	N/A
10379030	BC017647	N/A	N/A
10379034	Tlcd1	N/A	N/A
10379060	Proca1	N/A	N/A
10379068	Sdf2 /// Supt6h	N/A	N/A
10379075	2610507B11Rik	N/A	N/A
10379117	BC030499	N/A	N/A
10379153	Aldoc	N/A	N/A
10379163	Pigs	N/A	N/A
10379184	Slc46a1	N/A	N/A
10379190	Vtn	N/A	N/A
10379200	Sebox	N/A	N/A
10379204	Poldip2	N/A	N/A
10379215	Ift20	N/A	N/A
10379260	Ksr1	N/A	N/A
10379262	Nf1	N/A	N/A
10379321	Rab11fip4	N/A	N/A
10379338	---	N/A	N/A
10379340	Mir193	N/A	N/A
10379342	Mir365-2	N/A	N/A
10379344	Gm10387	N/A	N/A
10379346	Suz12	N/A	N/A
10379363	Atad5	N/A	N/A
10379387	---	N/A	N/A
10379389	Adap2	N/A	N/A
10379401	Rnf135	N/A	N/A
10379410	Rhot1	N/A	N/A
10379436	Rhbdl3	N/A	N/A
10379445	Zfp207	N/A	N/A
10379467	Psmd11	N/A	N/A
10379482	Cdk5r1	N/A	N/A
10379489	Tmem98	N/A	N/A
10379505	---	N/A	N/A
10379518	Ccl7	N/A	N/A
10379543	Tmem132e	N/A	N/A
10379557	Gm11426	N/A	N/A
10379560	Zfp830	N/A	N/A
10379564	Lig3 /// Rffl	N/A	N/A
10379627	---	N/A	N/A
10379630	Slfn2	N/A	N/A
10379646	Slfn3	N/A	N/A
10379652	Snord7 /// AI450353	N/A	N/A
10379654	Ap2b1	N/A	N/A
10379689	Taf15	N/A	N/A
10379727	Gm11428	N/A	N/A
10379736	1100001G20Rik	N/A	N/A
10379740	Heatr6	N/A	N/A
10379795	Synrg	N/A	N/A
10379836	Mrm1	N/A	N/A
10379838	Gm10374	N/A	N/A
10379866	Car4	N/A	N/A
10379875	Rpl13	N/A	N/A
10379889	Appbp2 /// D630032N06Rik	N/A	N/A
10379953	4632419I22Rik	N/A	N/A
10379957	Rnft1	N/A	N/A
10379981	Ptrh2	N/A	N/A
10379987	---	N/A	N/A
10379989	Fam33a	N/A	N/A
10379998	Trim37	N/A	N/A
10380059	Rnu3b4 /// Rnu3b2 /// Rnu3b1 /// Rnu3b3	N/A	N/A
10380061	Rnu3b4 /// Rnu3b2 /// Rnu3b1 /// Rnu3b3	N/A	N/A
10380063	Rnu3b4 /// Rnu3b2 /// Rnu3b1 /// Rnu3b3	N/A	N/A
10380065	Rnu3b4 /// Rnu3b2 /// Rnu3b1 /// Rnu3b3	N/A	N/A
10380109	Hsf5	N/A	N/A
10380116	Rnf43	N/A	N/A
10380129	Supt4h1 /// Gm3258	N/A	N/A
10380135	Mir142	N/A	N/A
10380210	Srsf1	N/A	N/A
10380219	Vezf1	N/A	N/A
10380226	Cuedc1	N/A	N/A
10380238	Mrps23	N/A	N/A
10380244	1700106J16Rik	N/A	N/A
10380248	Gm15698	N/A	N/A
10380250	2210409E12Rik /// Gm10367	N/A	N/A
10380252	Coil	N/A	N/A
10380260	Trim25	N/A	N/A
10380289	Mmd	N/A	N/A
10380297	Cox11 /// Tom1l1	N/A	N/A
10380303	Car10	N/A	N/A
10380318	---	N/A	N/A
10380341	Spag9	N/A	N/A
10380381	Tob1	N/A	N/A
10380384	Ankrd40	N/A	N/A

10380403	Lrrc59	N/A	N/A
10380411	Mrpl27	N/A	N/A
10380415	---	N/A	N/A
10380417	---	N/A	N/A
10380472	---	N/A	N/A
10380474	Hils1	N/A	N/A
10380477	Ppp1r9b	N/A	N/A
10380489	Samd14	N/A	N/A
10380514	Fam117a	N/A	N/A
10380524	Slc35b1	N/A	N/A
10380534	Spop	N/A	N/A
10380549	---	N/A	N/A
10380551	Phb	N/A	N/A
10380558	B130006D01Rik	N/A	N/A
10380560	Zfp652	N/A	N/A
10380579	---	N/A	N/A
10380620	Mir196a-1	N/A	N/A
10380634	Hoxb7	N/A	N/A
10380646	Mir10a	N/A	N/A
10380670	Rpl17 /// Gm10268 /// Rpl17-ps3	N/A	N/A
10380699	Copz2	N/A	N/A
10380710	Mir152	N/A	N/A
10380719	Sp6	N/A	N/A
10380739	Osbpl7	N/A	N/A
10380751	Mrpl45	N/A	N/A
10380761	Socs7	N/A	N/A
10380773	Arhgap23	N/A	N/A
10380793	Mllt6	N/A	N/A
10380815	Psmb3	N/A	N/A
10380819	Cwc25 /// 2810474C18Rik	N/A	N/A
10380821	Atp5l	N/A	N/A
10380830	B230217C12Rik	N/A	N/A
10380840	Cdk12	N/A	N/A
10380859	Cdk12	N/A	N/A
10380862	Ppp1r1b	N/A	N/A
10380871	Stard3	N/A	N/A
10380927	Grb7	N/A	N/A
10380954	Gm12	N/A	N/A
10380986	Psmd3	N/A	N/A
10381006	Thra /// Nr1d1	N/A	N/A
10381018	Msl1	N/A	N/A
10381049	Rapgefl1	N/A	N/A
10381063	Wipf2	N/A	N/A
10381082	Rara	N/A	N/A
10381096	Igfbp4	N/A	N/A
10381101	Gm11559	N/A	N/A
10381103	Krtap9-1 /// Gm11567 /// Gm11559 /// Gm11568	N/A	N/A
10381105	Gm11567	N/A	N/A
10381107	Krtap31-1	N/A	N/A
10381109	Gm11565	N/A	N/A
10381111	---	N/A	N/A
10381115	Eif1	N/A	N/A
10381118	Gast	N/A	N/A
10381122	Fkbp10 /// Nt5c3l	N/A	N/A
10381133	Klhl10	N/A	N/A
10381154	Cnp	N/A	N/A
10381170	Hspb9	N/A	N/A
10381172	Stat5a	N/A	N/A
10381187	Atp6v0a1	N/A	N/A
10381211	Naglu	N/A	N/A
10381226	Coasy	N/A	N/A
10381238	Mlx	N/A	N/A
10381250	Tubg1	N/A	N/A
10381260	Tubg2	N/A	N/A
10381272	Cntnap1	N/A	N/A
10381304	Vps25	N/A	N/A
10381379	---	N/A	N/A
10381381	---	N/A	N/A
10381383	---	N/A	N/A
10381385	---	N/A	N/A
10381387	G6pc	N/A	N/A
10381395	Rundc1	N/A	N/A
10381402	Rpl27	N/A	N/A
10381408	Ifi35	N/A	N/A
10381416	Rnd2	N/A	N/A
10381419	Nbr1	N/A	N/A
10381445	Tmem106a	N/A	N/A
10381458	Rnu2	N/A	N/A
10381460	Rnu2	N/A	N/A
10381462	Rdm1	N/A	N/A
10381470	Rnu2	N/A	N/A
10381472	Rnu2	N/A	N/A
10381474	Arl4d	N/A	N/A
10381477	Dhx8	N/A	N/A

10381502	---	N/A	N/A
10381526	Ppih	N/A	N/A
10381528	Nags	N/A	N/A
10381567	Tmub2	N/A	N/A
10381574	Rundc3a	N/A	N/A
10381588	Grn	N/A	N/A
10381601	Mdk	N/A	N/A
10381617	---	N/A	N/A
10381647	---	N/A	N/A
10381649	Higd1b	N/A	N/A
10381664	Kif18b	N/A	N/A
10381666	Dcakd	N/A	N/A
10381668	Nmt1	N/A	N/A
10381681	---	N/A	N/A
10381695	---	N/A	N/A
10381697	Hexim1	N/A	N/A
10381702	Hexim2	N/A	N/A
10381736	Rprml	N/A	N/A
10381744	Arf2	N/A	N/A
10381776	Mapt	N/A	N/A
10381807	Lin52 /// Gm7020	N/A	N/A
10381860	Mettl2	N/A	N/A
10381870	Gm10842	N/A	N/A
10381872	Tlk2	N/A	N/A
10381930	---	N/A	N/A
10381934	Tanc2	N/A	N/A
10381939	Tanc2	N/A	N/A
10381960	Cyb561 /// Gm9910	N/A	N/A
10382010	Dcaf7	N/A	N/A
10382022	Taco1	N/A	N/A
10382028	Map3k3	N/A	N/A
10382047	Gm10840	N/A	N/A
10382049	Ddx42	N/A	N/A
10382069	Psmc5	N/A	N/A
10382104	Snord104	N/A	N/A
10382106	Gm885	N/A	N/A
10382115	Ccdc45	N/A	N/A
10382136	1810010H24Rik	N/A	N/A
10382139	Psmd12	N/A	N/A
10382152	Helz	N/A	N/A
10382189	Apoh	N/A	N/A
10382198	---	N/A	N/A
10382200	Ccdc46	N/A	N/A
10382228	Axin2	N/A	N/A
10382243	Gna13	N/A	N/A
10382257	Amz2	N/A	N/A
10382284	Prkar1a	N/A	N/A
10382316	Kcnj16	N/A	N/A
10382328	Sox9	N/A	N/A
10382336	---	N/A	N/A
10382339	Gm16487	N/A	N/A
10382341	Sstr2	N/A	N/A
10382360	D11Wsu47e	N/A	N/A
10382367	---	N/A	N/A
10382369	Rpl38	N/A	N/A
10382376	Ttyh2	N/A	N/A
10382425	Gprc5c	N/A	N/A
10382435	Gprc5c	N/A	N/A
10382462	Slc9a3r1	N/A	N/A
10382470	Tmem104	N/A	N/A
10382502	Cdr2l	N/A	N/A
10382508	Ict1	N/A	N/A
10382516	Kctd2	N/A	N/A
10382538	Armc7	N/A	N/A
10382565	Mrps7	N/A	N/A
10382571	---	N/A	N/A
10382573	2310067B10Rik	N/A	N/A
10382698	1110017F19Rik	N/A	N/A
10382701	Sap30bp	N/A	N/A
10382756	Unk	N/A	N/A
10382774	2310004N24Rik /// Acox1	N/A	N/A
10382795	---	N/A	N/A
10382797	Fam100b /// Gm7367	N/A	N/A
10382844	Snord1b /// Snord1c	N/A	N/A
10382848	1110005A03Rik	N/A	N/A
10382852	Mfsd11	N/A	N/A
10382888	---	N/A	N/A
10382912	Sep 09	N/A	N/A
10382932	2900041M22Rik	N/A	N/A
10382935	Tnrc6c	N/A	N/A
10382980	Syngr2	N/A	N/A
10382985	Afmid /// Tk1	N/A	N/A
10383010	Socs3	N/A	N/A
10383012	Pgs1	N/A	N/A

10383023	Gm10099	N/A	N/A
10383025	C1qtnf1	N/A	N/A
10383088	Gaa	N/A	N/A
10383133	Slc26a11	N/A	N/A
10383152	Rnf213	N/A	N/A
10383168	Rnf213	N/A	N/A
10383192	Rnf213	N/A	N/A
10383194	Rnf213	N/A	N/A
10383196	Rnf213	N/A	N/A
10383198	Rnf213	N/A	N/A
10383200	Rnf213	N/A	N/A
10383202	Rnf213	N/A	N/A
10383204	Rnf213	N/A	N/A
10383206	Rnf213	N/A	N/A
10383208	Rnf213	N/A	N/A
10383210	Rnf213	N/A	N/A
10383212	Rnf213	N/A	N/A
10383214	Rnf213	N/A	N/A
10383233	Rnf213	N/A	N/A
10383245	Rptor	N/A	N/A
10383289	Baiap2	N/A	N/A
10383309	Slc38a10	N/A	N/A
10383311	Gm9734	N/A	N/A
10383342	0610009L18Rik	N/A	N/A
10383351	Gm9981	N/A	N/A
10383358	---	N/A	N/A
10383360	Ccdc137	N/A	N/A
10383365	Hgs	N/A	N/A
10383389	Mrpl12	N/A	N/A
10383395	Slc25a10	N/A	N/A
10383409	Gcgr	N/A	N/A
10383429	Npb	N/A	N/A
10383434	Notum	N/A	N/A
10383436	Aspscr1	N/A	N/A
10383453	Lrrc45	N/A	N/A
10383472	Rac3	N/A	N/A
10383479	Hmga1-rs1 /// Hmga1	N/A	N/A
10383485	Gps1	N/A	N/A
10383514	Uts2r	N/A	N/A
10383518	Hexdc	N/A	N/A
10383532	Narf	N/A	N/A
10383545	Foxk2	N/A	N/A
10383556	Fn3krp	N/A	N/A
10383564	Fn3k	N/A	N/A
10383575	Tbcd	N/A	N/A
10383671	Drg1	N/A	N/A
10383681	Patz1 /// Gm12592	N/A	N/A
10383684	Limk2	N/A	N/A
10383708	Rnf185	N/A	N/A
10383717	Inpp5j	N/A	N/A
10383731	Smtn	N/A	N/A
10383754	---	N/A	N/A
10383756	Ifitm2	N/A	N/A
10383758	Tug1	N/A	N/A
10383763	Gm10216	N/A	N/A
10383765	---	N/A	N/A
10383767	Osbp2	N/A	N/A
10383791	Slc35e4	N/A	N/A
10383799	Tcn2	N/A	N/A
10383810	Pes1 /// 4930556J24Rik	N/A	N/A
10383819	Sec14l2	N/A	N/A
10383850	---	N/A	N/A
10383867	Mtmr3	N/A	N/A
10383891	Cabp7	N/A	N/A
10383920	Nefh	N/A	N/A
10383930	Gm10278	N/A	N/A
10383943	Ewsr1	N/A	N/A
10383982	Znrf3	N/A	N/A
10383991	---	N/A	N/A
10383993	Ccdc117	N/A	N/A
10383999	Mrps24	N/A	N/A
10384004	Urgcp	N/A	N/A
10384020	Polm	N/A	N/A
10384032	Pold2	N/A	N/A
10384051	Gck	N/A	N/A
10384064	Camk2b	N/A	N/A
10384092	Nudcd3	N/A	N/A
10384100	Rps15a /// Gm11968	N/A	N/A
10384123	Ddx56	N/A	N/A
10384138	Tmed4	N/A	N/A
10384145	H2afv	N/A	N/A
10384150	Purb	N/A	N/A
10384152	Purb	N/A	N/A
10384179	---	N/A	N/A

10384192	Tbrg4	N/A	N/A
10384208	---	N/A	N/A
10384210	---	N/A	N/A
10384219	---	N/A	N/A
10384221	---	N/A	N/A
10384223	Igfbp3	N/A	N/A
10384229	---	N/A	N/A
10384233	Tns3	N/A	N/A
10384322	Hus1	N/A	N/A
10384349	Polr2c	N/A	N/A
10384363	---	N/A	N/A
10384370	---	N/A	N/A
10384378	Ddc	N/A	N/A
10384396	---	N/A	N/A
10384398	Grb10	N/A	N/A
10384423	Cobl	N/A	N/A
10384448	Sec61g	N/A	N/A
10384452	Ublcp1	N/A	N/A
10384454	---	N/A	N/A
10384456	---	N/A	N/A
10384474	Pno1	N/A	N/A
10384483	---	N/A	N/A
10384486	Etaa1	N/A	N/A
10384493	Gapdh	N/A	N/A
10384495	---	N/A	N/A
10384502	---	N/A	N/A
10384522	Actr2	N/A	N/A
10384529	Cep68	N/A	N/A
10384539	Slc1a4	N/A	N/A
10384552	Gapdh /// Gm2606 /// Gm2451 /// Gm10293	N/A	N/A
10384555	Aftph	N/A	N/A
10384566	---	N/A	N/A
10384577	---	N/A	N/A
10384579	Ugp2	N/A	N/A
10384603	Mdh1	N/A	N/A
10384652	---	N/A	N/A
10384654	---	N/A	N/A
10384672	Ahsa2 /// Usp34	N/A	N/A
10384691	0610010F05Rik	N/A	N/A
10384715	LOC100134990	N/A	N/A
10384717	Pex13	N/A	N/A
10384725	Rel	N/A	N/A
10384737	Papolg	N/A	N/A
10384762	A830031A19Rik	N/A	N/A
10384766	---	N/A	N/A
10384768	Gm10466	N/A	N/A
10384776	5730522E02Rik	N/A	N/A
10384778	---	N/A	N/A
10384780	Fancl	N/A	N/A
10384782	Vrk2 /// Fancl	N/A	N/A
10384795	Cyb5d1 /// Gm6685	N/A	N/A
10384797	Ccdc85a	N/A	N/A
10384808	Smek2 /// A630052C17Rik	N/A	N/A
10384811	Ccdc104	N/A	N/A
10384829	Rps27a	N/A	N/A
10384838	Eml6 /// Rtn4	N/A	N/A
10384883	4931440F15Rik	N/A	N/A
10384885	Spnb2	N/A	N/A
10384936	Acyp2	N/A	N/A
10384940	Erlec1	N/A	N/A
10385004	Nprl3	N/A	N/A
10385022	Efcab9	N/A	N/A
10385032	---	N/A	N/A
10385034	Rpsa	N/A	N/A
10385036	Fgf18	N/A	N/A
10385043	Npm1	N/A	N/A
10385052	Ranbp17	N/A	N/A
10385081	Anp32-ps	N/A	N/A
10385096	Kcnip1	N/A	N/A
10385114	Foxi1	N/A	N/A
10385153	Ccdc99	N/A	N/A
10385155	Fbll1	N/A	N/A
10385159	Rars	N/A	N/A
10385175	Wwc1	N/A	N/A
10385203	Odz2	N/A	N/A
10385234	---	N/A	N/A
10385236	Akr1b3 /// Gm6644	N/A	N/A
10385239	Mat2b	N/A	N/A
10385271	Ccng1	N/A	N/A
10385281	---	N/A	N/A
10385283	Gabrg2	N/A	N/A
10385297	Gabra1	N/A	N/A
10385335	---	N/A	N/A
10385343	Ttc1	N/A	N/A

10385361	Ublcp1	N/A	N/A
10385365	Lsm11	N/A	N/A
10385375	Thg1l	N/A	N/A
10385382	Sft2d1	N/A	N/A
10385384	Nipal4	N/A	N/A
10385391	Cyfip2	N/A	N/A
10385455	Timd2	N/A	N/A
10385466	Sgcd	N/A	N/A
10385477	---	N/A	N/A
10385484	Ddx5	N/A	N/A
10385486	Trim41	N/A	N/A
10385495	Cdk2ap1	N/A	N/A
10385500	Irgm1	N/A	N/A
10385511	Psme2 /// Psme2b-ps	N/A	N/A
10385513	9930111J21Rik2 /// 9930111J21Rik1 /// Gm5431	N/A	N/A
10385518	Tgtp1 /// Tgtp2 /// Gm12185	N/A	N/A
10385523	---	N/A	N/A
10385526	9930111J21Rik2 /// Gm5431 /// 9930111J21Rik1	N/A	N/A
10385540	Olfr1396	N/A	N/A
10385555	1700024J04Rik	N/A	N/A
10385557	Cnot6	N/A	N/A
10385570	Rpl30	N/A	N/A
10385572	Sqstm1	N/A	N/A
10385591	Maml1	N/A	N/A
10385599	Canx	N/A	N/A
10385616	Rufy1	N/A	N/A
10385635	Zfp354c	N/A	N/A
10385647	Zfp454	N/A	N/A
10385656	Zfp2	N/A	N/A
10385665	Zfp354b	N/A	N/A
10385686	Hnrnpab	N/A	N/A
10385699	Rmnd5b	N/A	N/A
10385716	0610009B22Rik	N/A	N/A
10385719	Sec24a	N/A	N/A
10385747	Phf15	N/A	N/A
10385761	Ube2b	N/A	N/A
10385768	Olfr1373 /// Olfr1371 /// Olfr1380 /// Olfr1382 /// Olfr1381	N/A	N/A
10385774	Olfr1373 /// Olfr1371 /// Olfr1380 /// Olfr1382 /// Olfr1381	N/A	N/A
10385790	Hspa4	N/A	N/A
10385814	Leap2	N/A	N/A
10385818	Uqcrq	N/A	N/A
10385822	Gm10447	N/A	N/A
10385824	Gm9837	N/A	N/A
10385870	Irf1	N/A	N/A
10385893	Slc22a4	N/A	N/A
10385903	Pdlim4	N/A	N/A
10385926	Cdc42se2	N/A	N/A
10385966	Anxa6	N/A	N/A
10385993	Anxa6	N/A	N/A
10386058	Sparc	N/A	N/A
10386070	Atox1	N/A	N/A
10386086	Nmur2	N/A	N/A
10386093	Snord1b /// Snord1c	N/A	N/A
10386095	Fam114a2	N/A	N/A
10386123	A430005L14Rik	N/A	N/A
10386171	Ndufs3	N/A	N/A
10386178	Olfr30	N/A	N/A
10386180	---	N/A	N/A
10386185	Olfr330 /// Olfr325 /// Olfr329-ps /// Olfr331	N/A	N/A
10386187	Rpl24	N/A	N/A
10386189	Olfr330	N/A	N/A
10386191	Olfr330 /// Olfr325 /// Olfr329-ps /// Olfr328	N/A	N/A
10386193	Olfr328	N/A	N/A
10386207	Olfr318	N/A	N/A
10386209	Olfr317	N/A	N/A
10386219	Zfp39	N/A	N/A
10386230	Rnf187	N/A	N/A
10386236	Hist3h2bb-ps	N/A	N/A
10386344	A230051G13Rik	N/A	N/A
10386349	4930543L23Rik	N/A	N/A
10386359	Guk1	N/A	N/A
10386368	2310033P09Rik	N/A	N/A
10386370	Arf1	N/A	N/A
10386388	Snap47	N/A	N/A
10386394	Zfp867	N/A	N/A
10386400	Arih2	N/A	N/A
10386402	Zkscan17	N/A	N/A
10386414	Zkscan17	N/A	N/A
10386416	Olfr222	N/A	N/A
10386418	---	N/A	N/A
10386423	Pld6	N/A	N/A
10386427	Flcn	N/A	N/A
10386442	Cops3	N/A	N/A
10386455	Rasd1	N/A	N/A

10386460	Pemt	N/A	N/A
10386470	---	N/A	N/A
10386473	Srebf1	N/A	N/A
10386495	Tom1l2	N/A	N/A
10386518	Atpaf2	N/A	N/A
10386527	---	N/A	N/A
10386529	---	N/A	N/A
10386531	---	N/A	N/A
10386533	---	N/A	N/A
10386535	---	N/A	N/A
10386537	---	N/A	N/A
10386539	---	N/A	N/A
10386541	---	N/A	N/A
10386543	Pabpc4	N/A	N/A
10386546	---	N/A	N/A
10386548	Pabpc4	N/A	N/A
10386551	Flii	N/A	N/A
10386628	Gm16515	N/A	N/A
10386633	Gm16516	N/A	N/A
10386636	Usp22	N/A	N/A
10386683	Slc47a1	N/A	N/A
10386703	Gm12271	N/A	N/A
10386705	Rnf112	N/A	N/A
10386723	Mapk7	N/A	N/A
10386741	---	N/A	N/A
10386743	Epn2	N/A	N/A
10386756	---	N/A	N/A
10386775	Prpsap2	N/A	N/A
10386789	Ulk2	N/A	N/A
10386821	---	N/A	N/A
10386824	Akap10	N/A	N/A
10386844	Zswim7	N/A	N/A
10386850	Ncor1	N/A	N/A
10386909	Cenpv	N/A	N/A
10386916	BC046404	N/A	N/A
10386921	Zfp287	N/A	N/A
10386934	Wsb2	N/A	N/A
10386937	Zfp286	N/A	N/A
10386947	Gm10291	N/A	N/A
10386951	Hs3st3b1	N/A	N/A
10386955	Cox10	N/A	N/A
10386963	Hs3st3a1 /// F930015N05Rik	N/A	N/A
10386965	AU040829	N/A	N/A
10386990	---	N/A	N/A
10386992	1700086D15Rik	N/A	N/A
10387014	Map2k4	N/A	N/A
10387100	Shisa6	N/A	N/A
10387180	Ndel1	N/A	N/A
10387194	Odf4	N/A	N/A
10387219	Rangrf /// Slc25a35	N/A	N/A
10387251	2310047M10Rik /// 9330160F10Rik	N/A	N/A
10387255	Hes7	N/A	N/A
10387316	Chd3	N/A	N/A
10387363	Cyb5d1 /// Gm6685	N/A	N/A
10387368	Tmem88 /// Lsmd1	N/A	N/A
10387372	Kdm6b	N/A	N/A
10387505	Atp1b2	N/A	N/A
10387514	---	N/A	N/A
10387525	Mpdu1	N/A	N/A
10387536	Cd68	N/A	N/A
10387545	Eif4a1	N/A	N/A
10387557	---	N/A	N/A
10387559	Senp3	N/A	N/A
10387588	Polr2a	N/A	N/A
10387648	Tmem102 /// G630025P09Rik	N/A	N/A
10387659	Nlgn2	N/A	N/A
10387757	Rai12	N/A	N/A
10387768	Dvl2 /// Acadvl	N/A	N/A
10387871	Pelp1	N/A	N/A
10387903	Vmo1	N/A	N/A
10387907	Pld2	N/A	N/A
10387922	Slc25a11	N/A	N/A
10387932	Pfn1	N/A	N/A
10387936	Spag7	N/A	N/A
10387971	Inca1	N/A	N/A
10387983	---	N/A	N/A
10387992	Nup88	N/A	N/A
10388010	C1qbp	N/A	N/A
10388018	Dhx33	N/A	N/A
10388033	Derl2	N/A	N/A
10388042	6330403K07Rik	N/A	N/A
10388045	---	N/A	N/A
10388109	Pitpnm3	N/A	N/A
10388132	4933427D14Rik	N/A	N/A

10388154	Med31	N/A	N/A
10388160	Slc13a5	N/A	N/A
10388225	---	N/A	N/A
10388227	Cyb5d2	N/A	N/A
10388238	Tmem93	N/A	N/A
10388241	Ctns	N/A	N/A
10388254	Aspa	N/A	N/A
10388265	Olfr1	N/A	N/A
10388267	Olfr378	N/A	N/A
10388272	Olfr380	N/A	N/A
10388274	Olfr381	N/A	N/A
10388276	Olfr382	N/A	N/A
10388280	Olfr385	N/A	N/A
10388284	Olfr389	N/A	N/A
10388286	Olfr391-ps	N/A	N/A
10388290	Olfr393	N/A	N/A
10388292	Olfr394	N/A	N/A
10388294	Olfr395	N/A	N/A
10388298	Olfr139	N/A	N/A
10388300	Olfr399	N/A	N/A
10388304	---	N/A	N/A
10388306	Olfr410	N/A	N/A
10388308	Olfr411	N/A	N/A
10388310	Rap1gap2	N/A	N/A
10388337	Pafah1b1	N/A	N/A
10388352	Sgsm2	N/A	N/A
10388377	Srr	N/A	N/A
10388392	Dph1 /// Ovca2	N/A	N/A
10388430	Serpinf1	N/A	N/A
10388440	Serpinf2	N/A	N/A
10388451	Wdr81	N/A	N/A
10388461	Cox7c /// Gm10012	N/A	N/A
10388465	Doc2b	N/A	N/A
10388476	Rph3al	N/A	N/A
10388488	Fam101b	N/A	N/A
10388492	Vps53	N/A	N/A
10388517	Gemin4	N/A	N/A
10388520	Glod4	N/A	N/A
10388532	Nxn	N/A	N/A
10388545	Abr	N/A	N/A
10388579	Gosr1	N/A	N/A
10388591	Cpd	N/A	N/A
10388613	Ccdc55	N/A	N/A
10388623	Mir423	N/A	N/A
10388648	Ankrd13b	N/A	N/A
10388679	Abhd15 /// Gm10392	N/A	N/A
10388682	Taok1	N/A	N/A
10388684	Taok1	N/A	N/A
10388716	Gm10277	N/A	N/A
10388718	Pipox	N/A	N/A
10388732	Dhrs13	N/A	N/A
10388734	Eral1	N/A	N/A
10388745	Lsm6 /// Gm10043	N/A	N/A
10388747	BC017647 /// Gm10003	N/A	N/A
10388749	Traf4	N/A	N/A
10388784	Rpl23a	N/A	N/A
10388786	Supt6h	N/A	N/A
10388847	Sarm1	N/A	N/A
10388861	Tmem199	N/A	N/A
10388869	Tnfaip1	N/A	N/A
10388880	Tmem97	N/A	N/A
10388884	Nlk	N/A	N/A
10388896	---	N/A	N/A
10388902	Lgals9	N/A	N/A
10388914	Ksr1	N/A	N/A
10388938	Wsb1	N/A	N/A
10388952	Gm9964	N/A	N/A
10388954	Omg	N/A	N/A
10388958	Evi2a /// Evi2b	N/A	N/A
10388971	Utp6	N/A	N/A
10388994	Gm10386	N/A	N/A
10388996	Crlf3	N/A	N/A
10389005	1110002N22Rik	N/A	N/A
10389010	5730455P16Rik	N/A	N/A
10389022	Myo1d	N/A	N/A
10389025	Myo1d	N/A	N/A
10389047	Accn1	N/A	N/A
10389062	1700071K01Rik	N/A	N/A
10389087	Rffl	N/A	N/A
10389099	Rad51l3	N/A	N/A
10389114	Nle1	N/A	N/A
10389127	Unc45b	N/A	N/A
10389162	Rpl12	N/A	N/A
10389164	Pex12	N/A	N/A

10389170	Gas2l2	N/A	N/A
10389207	Ccl5	N/A	N/A
10389214	Ccl9	N/A	N/A
10389222	Ccl6	N/A	N/A
10389229	Rpl9	N/A	N/A
10389238	Dusp14	N/A	N/A
10389245	Tada2a	N/A	N/A
10389261	Gm11437	N/A	N/A
10389269	Aatf	N/A	N/A
10389283	Lhx1	N/A	N/A
10389293	Mrm1	N/A	N/A
10389300	Dhrs11	N/A	N/A
10389308	Ggnbp2	N/A	N/A
10389339	Usp32	N/A	N/A
10389373	Appbp2	N/A	N/A
10389387	Ppm1d	N/A	N/A
10389389	Gm10372	N/A	N/A
10389421	Ints2	N/A	N/A
10389451	Med13	N/A	N/A
10389482	---	N/A	N/A
10389484	Rps6kb1	N/A	N/A
10389505	Gm9975	N/A	N/A
10389522	Rpl13a /// Rpl13a-ps1	N/A	N/A
10389524	---	N/A	N/A
10389526	Cltc	N/A	N/A
10389561	Dhx40	N/A	N/A
10389590	Gdpd1	N/A	N/A
10389601	1200011M11Rik	N/A	N/A
10389617	Ppm1e	N/A	N/A
10389625	---	N/A	N/A
10389668	Olfr462	N/A	N/A
10389670	Olfr463	N/A	N/A
10389672	Olfr464	N/A	N/A
10389674	Dynll2	N/A	N/A
10389680	Msi2	N/A	N/A
10389699	---	N/A	N/A
10389701	Akap1	N/A	N/A
10389717	---	N/A	N/A
10389719	Scpep1	N/A	N/A
10389736	2210409E12Rik /// Gm10367	N/A	N/A
10389738	Dgke	N/A	N/A
10389752	Nog	N/A	N/A
10389754	4932411E22Rik	N/A	N/A
10389759	Ankfn1	N/A	N/A
10389775	Pctp	N/A	N/A
10389783	Gm10276	N/A	N/A
10389795	Stxbp4	N/A	N/A
10389797	Stxbp4	N/A	N/A
10389816	Tom1l1 /// Cox11	N/A	N/A
10389858	Dscaml1 /// Nme2	N/A	N/A
10389875	Rpl27	N/A	N/A
10389877	Wfikkn2	N/A	N/A
10389882	Luc7l3	N/A	N/A
10389894	Abcc3	N/A	N/A
10390022	Rsad1	N/A	N/A
10390032	Acsf2	N/A	N/A
10390103	Pdk2	N/A	N/A
10390153	Myst2	N/A	N/A
10390184	Gm9796	N/A	N/A
10390209	Igf2bp1	N/A	N/A
10390227	Ube2z	N/A	N/A
10390237	Atp5g1	N/A	N/A
10390244	---	N/A	N/A
10390252	---	N/A	N/A
10390256	Gm11529	N/A	N/A
10390258	Snx11	N/A	N/A
10390269	Gm11517	N/A	N/A
10390271	Nfe2l1	N/A	N/A
10390283	Cdk5rap3	N/A	N/A
10390299	Pnpo	N/A	N/A
10390319	Lrrc46	N/A	N/A
10390336	Tbkbp1	N/A	N/A
10390381	Npepps	N/A	N/A
10390426	4933428G20Rik /// Arhgap23	N/A	N/A
10390430	Srcin1	N/A	N/A
10390454	E130012A19Rik	N/A	N/A
10390473	Pip4k2b	N/A	N/A
10390502	Rpl23	N/A	N/A
10390505	Snora21	N/A	N/A
10390574	Fbxl20	N/A	N/A
10390595	Med1	N/A	N/A
10390635	1810046J19Rik	N/A	N/A
10390653	Med24	N/A	N/A
10390691	Nr1d1 /// Thra	N/A	N/A

10390705	Gjd3	N/A	N/A
10390746	---	N/A	N/A
10390763	Ccr7	N/A	N/A
10390780	Krt222	N/A	N/A
10390831	Krt10	N/A	N/A
10390895	Krtap3-1	N/A	N/A
10390897	Krtap1-5	N/A	N/A
10390907	Krtap1-3	N/A	N/A
10390909	Krtap9-3	N/A	N/A
10390911	Gm11562 /// Gm11938 /// Krtap2-4	N/A	N/A
10390913	Gm11562 /// Gm11938 /// Krtap2-4	N/A	N/A
10390915	Gm11562 /// Gm11938 /// Krtap2-4	N/A	N/A
10390917	Gm11562 /// Gm11938 /// Krtap2-4	N/A	N/A
10390919	Krtap4-1	N/A	N/A
10390934	Krtap4-8 /// Krtap4-6 /// Gm11564 /// Gm11563 /// Gm11595	N/A	N/A
10390939	Gm11569 /// Gm11554 /// Krtap4-13	N/A	N/A
10390945	Gm11569 /// Gm11554 /// Krtap4-13	N/A	N/A
10390954	---	N/A	N/A
10390956	Krtap4-16	N/A	N/A
10390961	Krtap17-1	N/A	N/A
10391084	Hap1	N/A	N/A
10391100	Atp5g1	N/A	N/A
10391103	Jup	N/A	N/A
10391119	1110036O03Rik	N/A	N/A
10391130	Nt5c3l	N/A	N/A
10391143	Klhl11	N/A	N/A
10391146	Acly	N/A	N/A
10391178	Dnajc7	N/A	N/A
10391221	Kat2a	N/A	N/A
10391242	Rab5c	N/A	N/A
10391277	Ghdc	N/A	N/A
10391286	Stat5b	N/A	N/A
10391301	Stat3	N/A	N/A
10391407	Ccdc56	N/A	N/A
10391410	Becn1 /// Cntd1	N/A	N/A
10391454	Vat1	N/A	N/A
10391488	Rnu2	N/A	N/A
10391513	Dusp3	N/A	N/A
10391518	Mpp3	N/A	N/A
10391555	Ppy	N/A	N/A
10391567	Tmem101	N/A	N/A
10391572	Lsm12	N/A	N/A
10391577	Hdac5	N/A	N/A
10391610	---	N/A	N/A
10391625	Ubtf	N/A	N/A
10391732	Gpatch8	N/A	N/A
10391742	Gpatch8	N/A	N/A
10391744	Gpatch8	N/A	N/A
10391746	Gpatch8	N/A	N/A
10391750	Gpatch8	N/A	N/A
10391755	Ccdc43	N/A	N/A
10391768	Eftud2	N/A	N/A
10391798	Gfap	N/A	N/A
10391828	C1ql1	N/A	N/A
10391914	Sh3d20 /// Arhgap27	N/A	N/A
10391957	Wnt9b	N/A	N/A
10391963	Nsf	N/A	N/A
10391985	Taf1d	N/A	N/A
10391987	Gm10844	N/A	N/A
10391990	1700081L11Rik	N/A	N/A
10392010	1700081L11Rik	N/A	N/A
10392012	Cdc27	N/A	N/A
10392033	---	N/A	N/A
10392056	Cyb561	N/A	N/A
10392070	Strada	N/A	N/A
10392087	Ccdc47	N/A	N/A
10392096	---	N/A	N/A
10392098	Ftsj3	N/A	N/A
10392177	Icam2	N/A	N/A
10392183	Ern1	N/A	N/A
10392207	Tex2	N/A	N/A
10392251	Ddx5	N/A	N/A
10392259	Smurf2	N/A	N/A
10392261	Smurf2	N/A	N/A
10392284	Kpna2	N/A	N/A
10392300	Bptf	N/A	N/A
10392318	Bptf	N/A	N/A
10392328	Nol11	N/A	N/A
10392347	Pitpnc1	N/A	N/A
10392369	Cacng4	N/A	N/A
10392374	Cacng5	N/A	N/A
10392388	Prkca	N/A	N/A
10392410	Gm10838	N/A	N/A
10392413	---	N/A	N/A

10392415	Rgs9	N/A	N/A
10392437	Gm11696 /// Gna13	N/A	N/A
10392449	Wipi1	N/A	N/A
10392464	Fam20a	N/A	N/A
10392484	Abca8b	N/A	N/A
10392522	Abca8a	N/A	N/A
10392601	Abca6	N/A	N/A
10392642	Abca5	N/A	N/A
10392683	Gm10836	N/A	N/A
10392685	BC006965	N/A	N/A
10392699	---	N/A	N/A
10392701	Slc39a11	N/A	N/A
10392721	Cpsf4l	N/A	N/A
10392735	Cdc42ep4	N/A	N/A
10392787	4932435O22Rik	N/A	N/A
10392791	Btbd17	N/A	N/A
10392825	Clm3 /// Gm11711	N/A	N/A
10392834	Clm3 /// Gm11711	N/A	N/A
10392856	Nat9	N/A	N/A
10392881	Fdxr	N/A	N/A
10392894	Fads6	N/A	N/A
10392910	C630004H02Rik	N/A	N/A
10392930	Atp5h /// Gm4953 /// Gm5048	N/A	N/A
10392936	Nt5c	N/A	N/A
10392943	Hn1	N/A	N/A
10392947	Sumo2 /// Mir684-1 /// Mir684-2	N/A	N/A
10392953	Gga3	N/A	N/A
10392970	Mif4gd	N/A	N/A
10392983	Slc25a19	N/A	N/A
10392996	Rpl21 /// Gm6813 /// Gm9104 /// Rpl21-ps4 /// Rpl21-ps7 /// Gm16416 /// Rpl21-ps12 /// Rpl21-ps10 /// Gm8054	N/A	N/A
10393045	B230344G16Rik /// Itgb4	N/A	N/A
10393058	H3f3b	N/A	N/A
10393098	Wbp2	N/A	N/A
10393125	Mrpl38	N/A	N/A
10393166	St6galnac2 /// 1810032O08Rik	N/A	N/A
10393177	Acox1	N/A	N/A
10393222	Srp68	N/A	N/A
10393239	---	N/A	N/A
10393241	Exoc7	N/A	N/A
10393264	---	N/A	N/A
10393272	Rnf157	N/A	N/A
10393309	Prpsap1	N/A	N/A
10393320	Ube2o	N/A	N/A
10393379	Mxra7	N/A	N/A
10393395	Srsf2	N/A	N/A
10393404	LOC100505199	N/A	N/A
10393406	---	N/A	N/A
10393431	Tk1	N/A	N/A
10393509	Usp36	N/A	N/A
10393559	Timp2	N/A	N/A
10393569	Ddc8	N/A	N/A
10393573	Lgals3bp	N/A	N/A
10393594	D11Bwg0517e	N/A	N/A
10393642	Eif4a3	N/A	N/A
10393662	Nptx1	N/A	N/A
10393668	Aatk	N/A	N/A
10393685	Mir338	N/A	N/A
10393714	2410002I01Rik	N/A	N/A
10393728	Slc38a10	N/A	N/A
10393749	2810410L24Rik	N/A	N/A
10393754	Actg1	N/A	N/A
10393761	2310003H01Rik	N/A	N/A
10393774	Nploc4	N/A	N/A
10393801	1810049H13Rik	N/A	N/A
10393814	Fam195b	N/A	N/A
10393823	P4hb	N/A	N/A
10393836	Arhgdia	N/A	N/A
10393851	Pcyt2	N/A	N/A
10393866	Sirt7	N/A	N/A
10393877	---	N/A	N/A
10393879	---	N/A	N/A
10393917	Stra13	N/A	N/A
10393926	Dcxr	N/A	N/A
10393944	Rfng	N/A	N/A
10393955	Dus1l	N/A	N/A
10393970	Fasn	N/A	N/A
10394040	Csnk1d	N/A	N/A
10394080	---	N/A	N/A
10394093	BC017643	N/A	N/A
10394102	Wdr45l	N/A	N/A
10394119	Zfp750	N/A	N/A
10394142	Hmgb1	N/A	N/A
10394158	Kif3c /// 1110002L01Rik	N/A	N/A
10394173	Asxl2	N/A	N/A

10394186	Dtnb	N/A	N/A
10394209	Dnmt3a	N/A	N/A
10394238	---	N/A	N/A
10394258	Adcy3 /// Cenpo	N/A	N/A
10394283	2410017P09Rik /// Cenpo	N/A	N/A
10394286	2410017P09Rik	N/A	N/A
10394288	Itsn2	N/A	N/A
10394344	Gm6682	N/A	N/A
10394353	0610009D07Rik	N/A	N/A
10394366	Atad2b	N/A	N/A
10394381	Atad2b	N/A	N/A
10394389	2810032G03Rik	N/A	N/A
10394392	Nfyc	N/A	N/A
10394394	Apob	N/A	N/A
10394429	1110057K04Rik	N/A	N/A
10394448	Pum2	N/A	N/A
10394471	Sdc1	N/A	N/A
10394498	Wdr35	N/A	N/A
10394527	Ttc32	N/A	N/A
10394532	Ube2f /// Gm5434	N/A	N/A
10394538	Acaca	N/A	N/A
10394555	Rdh14	N/A	N/A
10394558	Rpl29 /// Gm3550	N/A	N/A
10394591	Rpl15 /// Gm4294	N/A	N/A
10394593	Fam49a	N/A	N/A
10394609	Rpl36 /// Gm13611 /// Gm5614 /// Gm5745	N/A	N/A
10394611	Nbas	N/A	N/A
10394625	Nbas	N/A	N/A
10394627	Nbas	N/A	N/A
10394671	Gm16497	N/A	N/A
10394674	Socs2 /// Gm9847	N/A	N/A
10394676	---	N/A	N/A
10394678	---	N/A	N/A
10394680	---	N/A	N/A
10394682	---	N/A	N/A
10394685	Ntsr2	N/A	N/A
10394731	---	N/A	N/A
10394733	Rps23	N/A	N/A
10394735	Pdia6	N/A	N/A
10394749	Nol10	N/A	N/A
10394770	Odc1	N/A	N/A
10394776	---	N/A	N/A
10394778	Hpcal1	N/A	N/A
10394789	B430203G13Rik	N/A	N/A
10394791	---	N/A	N/A
10394798	---	N/A	N/A
10394805	2410018L13Rik	N/A	N/A
10394812	---	N/A	N/A
10394819	---	N/A	N/A
10394821	---	N/A	N/A
10394823	---	N/A	N/A
10394827	---	N/A	N/A
10394829	---	N/A	N/A
10394831	Gm5784	N/A	N/A
10394833	---	N/A	N/A
10394837	LOC100043371 /// 2410018L13Rik	N/A	N/A
10394843	---	N/A	N/A
10394846	LOC100043371	N/A	N/A
10394850	---	N/A	N/A
10394858	---	N/A	N/A
10394860	B230354O11Rik	N/A	N/A
10394892	Cpsf3	N/A	N/A
10394912	---	N/A	N/A
10394922	---	N/A	N/A
10394926	---	N/A	N/A
10394934	---	N/A	N/A
10394936	---	N/A	N/A
10394938	---	N/A	N/A
10394940	---	N/A	N/A
10394942	Taf1b	N/A	N/A
10394954	Grhl1	N/A	N/A
10394978	Rrm2	N/A	N/A
10394988	---	N/A	N/A
10394990	Mboat2	N/A	N/A
10395005	Kidins220	N/A	N/A
10395049	Rnaseh1	N/A	N/A
10395058	Adi1	N/A	N/A
10395064	Tssc1	N/A	N/A
10395074	Myt1l	N/A	N/A
10395103	Pxdn	N/A	N/A
10395129	Tmem18	N/A	N/A
10395155	Fam110c	N/A	N/A
10395198	---	N/A	N/A
10395225	Gm10473	N/A	N/A

10395227	Hbp1 /// Cog5	N/A	N/A
10395250	---	N/A	N/A
10395252	2010109K11Rik	N/A	N/A
10395257	---	N/A	N/A
10395259	Nampt	N/A	N/A
10395273	Gdap10	N/A	N/A
10395275	LOC280487	N/A	N/A
10395287	Atxn7l1	N/A	N/A
10395312	Twistnb	N/A	N/A
10395328	Snx13	N/A	N/A
10395376	Ankmy2	N/A	N/A
10395389	Sostdc1	N/A	N/A
10395394	Ispd	N/A	N/A
10395414	Tmem195	N/A	N/A
10395428	Dgkb	N/A	N/A
10395457	Etv1	N/A	N/A
10395463	Ifrd1 /// Gm7008	N/A	N/A
10395466	Dock4	N/A	N/A
10395520	Immp2l	N/A	N/A
10395534	Gm2027	N/A	N/A
10395538	Pnpla8	N/A	N/A
10395553	Nrcam	N/A	N/A
10395594	---	N/A	N/A
10395596	Foxg1 /// 3110039M20Rik	N/A	N/A
10395604	Gm9804	N/A	N/A
10395606	Gm10468	N/A	N/A
10395608	---	N/A	N/A
10395610	Gm9808	N/A	N/A
10395672	Ap4s1	N/A	N/A
10395682	Gm5785	N/A	N/A
10395684	Nubpl	N/A	N/A
10395692	Arhgap5	N/A	N/A
10395702	Akap6	N/A	N/A
10395719	Npas3	N/A	N/A
10395733	Npas3	N/A	N/A
10395737	Rpl21 /// Gm7555	N/A	N/A
10395739	Srp54c /// Srp54a /// Srp54b	N/A	N/A
10395788	Srp54c /// Srp54a /// Srp54b	N/A	N/A
10395805	Fam177a /// 1700047I17Rik2	N/A	N/A
10395807	1110008L16Rik	N/A	N/A
10395816	Psma6	N/A	N/A
10395825	Aldoart2	N/A	N/A
10395827	Insm2	N/A	N/A
10395829	Ralgapa1	N/A	N/A
10395831	Brms1l	N/A	N/A
10395845	---	N/A	N/A
10395847	---	N/A	N/A
10395908	---	N/A	N/A
10395910	Pnn	N/A	N/A
10395922	Rps2 /// Rps2-ps6 /// Gm5921 /// Gm6139	N/A	N/A
10395925	Mia2	N/A	N/A
10395932	Ctage5	N/A	N/A
10395953	---	N/A	N/A
10395961	Lrfn5	N/A	N/A
10395971	---	N/A	N/A
10395976	Dnajb6	N/A	N/A
10395978	Gm527	N/A	N/A
10395984	Fam179b	N/A	N/A
10396008	Prpf39 /// Fkbp3	N/A	N/A
10396030	Fancm	N/A	N/A
10396059	Rpl17 /// Gm10268 /// Rpl17-ps3	N/A	N/A
10396064	Txndc9	N/A	N/A
10396066	---	N/A	N/A
10396074	Mgat2	N/A	N/A
10396076	1110034A24Rik /// 9330151L19Rik	N/A	N/A
10396094	Klhdc2	N/A	N/A
10396108	Arf6	N/A	N/A
10396110	---	N/A	N/A
10396112	Atp5s	N/A	N/A
10396121	4930512B01Rik	N/A	N/A
10396125	Atl1	N/A	N/A
10396141	---	N/A	N/A
10396143	Gm3086	N/A	N/A
10396146	Nin /// Gm9997	N/A	N/A
10396161	Tmx1	N/A	N/A
10396170	Frmd6	N/A	N/A
10396175	Gm10457	N/A	N/A
10396177	Actr10	N/A	N/A
10396193	Psma3	N/A	N/A
10396205	Arid4a	N/A	N/A
10396270	Dact1	N/A	N/A
10396278	Daam1	N/A	N/A
10396306	Jkamp	N/A	N/A
10396346	1810048J11Rik	N/A	N/A

10396358	Ppm1a	N/A	N/A
10396367	Six6	N/A	N/A
10396383	Slc38a6	N/A	N/A
10396419	---	N/A	N/A
10396421	Hif1a	N/A	N/A
10396442	Snapc1	N/A	N/A
10396466	1700086L19Rik	N/A	N/A
10396472	Dbpht2	N/A	N/A
10396606	Syne2	N/A	N/A
10396608	Syne2	N/A	N/A
10396610	Mthfd1	N/A	N/A
10396640	Akap5	N/A	N/A
10396652	Hspa2	N/A	N/A
10396656	Gm70	N/A	N/A
10396669	Gm10451	N/A	N/A
10396694	Churc1	N/A	N/A
10396699	Fntb	N/A	N/A
10396712	Fut8	N/A	N/A
10396730	---	N/A	N/A
10396740	Gphn	N/A	N/A
10396778	Mpp5	N/A	N/A
10396795	Eif2s1	N/A	N/A
10396867	Exd2	N/A	N/A
10396877	Galntl1	N/A	N/A
10396896	Slc39a9	N/A	N/A
10396919	4933426M11Rik	N/A	N/A
10396926	Srsf5	N/A	N/A
10396936	Smoc1	N/A	N/A
10396950	---	N/A	N/A
10396952	Ttc9	N/A	N/A
10396956	Pcnx	N/A	N/A
10397000	---	N/A	N/A
10397002	Sipa1l1	N/A	N/A
10397054	Dcaf4	N/A	N/A
10397068	Rbm25	N/A	N/A
10397081	Rbm25	N/A	N/A
10397083	Rbm25	N/A	N/A
10397085	Rbm25	N/A	N/A
10397094	Psen1	N/A	N/A
10397143	2410016O06Rik	N/A	N/A
10397145	Acot2	N/A	N/A
10397172	Acot6	N/A	N/A
10397189	Ptgr2	N/A	N/A
10397201	Zfp410	N/A	N/A
10397230	2900006K08Rik	N/A	N/A
10397249	Lin52 /// Gm7020	N/A	N/A
10397267	Isca2	N/A	N/A
10397275	Ltbp2 /// D030025P21Rik	N/A	N/A
10397281	Ylpm1	N/A	N/A
10397311	Dlst	N/A	N/A
10397332	Eif2b2	N/A	N/A
10397342	Fam164c	N/A	N/A
10397416	1700019E19Rik	N/A	N/A
10397428	1700020O03Rik	N/A	N/A
10397450	Vash1	N/A	N/A
10397476	2310044G17Rik	N/A	N/A
10397482	Tmem63c	N/A	N/A
10397507	Gstz1 /// Pomt2	N/A	N/A
10397518	Ahsa1	N/A	N/A
10397528	Rpl21	N/A	N/A
10397536	---	N/A	N/A
10397538	---	N/A	N/A
10397541	Gm8300 /// Gm2022 /// Gm2056 /// Gm16368	N/A	N/A
10397543	Eif1a /// Gm8300 /// Gm2022 /// Gm16368	N/A	N/A
10397555	Gm8587	N/A	N/A
10397559	---	N/A	N/A
10397575	Nrxn3	N/A	N/A
10397602	---	N/A	N/A
10397627	---	N/A	N/A
10397633	Flrt2	N/A	N/A
10397639	---	N/A	N/A
10397642	---	N/A	N/A
10397645	Gpr65	N/A	N/A
10397666	Zc3h14	N/A	N/A
10397683	Ttc8	N/A	N/A
10397699	Rpl30	N/A	N/A
10397701	---	N/A	N/A
10397703	3300002A11Rik	N/A	N/A
10397708	---	N/A	N/A
10397715	---	N/A	N/A
10397717	---	N/A	N/A
10397719	Tdp1	N/A	N/A
10397741	Psmc1	N/A	N/A
10397750	---	N/A	N/A

10397752	Calm1	N/A	N/A
10397761	Gm10432	N/A	N/A
10397763	9030617O03Rik	N/A	N/A
10397780	Ccdc88c	N/A	N/A
10397784	Smek1 /// D130020L05Rik	N/A	N/A
10397786	Kif4b	N/A	N/A
10397788	Kif4b	N/A	N/A
10397790	---	N/A	N/A
10397818	Cpsf2	N/A	N/A
10397835	Slc24a4	N/A	N/A
10397866	Golga5	N/A	N/A
10397882	Chga	N/A	N/A
10397891	D230037D09Rik	N/A	N/A
10397895	Ubr7	N/A	N/A
10397912	Unc79	N/A	N/A
10397966	Otub2	N/A	N/A
10397975	Ifi27l1	N/A	N/A
10397984	Ppp4r4	N/A	N/A
10398011	Serpina4-ps1	N/A	N/A
10398069	Serpina3m	N/A	N/A
10398075	Serpina3n	N/A	N/A
10398080	Gm10000	N/A	N/A
10398085	Glrx5	N/A	N/A
10398095	Tcl1b1	N/A	N/A
10398130	Ak7	N/A	N/A
10398147	Papola	N/A	N/A
10398173	Vrk1	N/A	N/A
10398193	3110018I06Rik	N/A	N/A
10398195	Ccnk	N/A	N/A
10398224	Cyp46a1	N/A	N/A
10398286	Mir342	N/A	N/A
10398288	Yy1	N/A	N/A
10398297	Mir345	N/A	N/A
10398299	Slc25a47	N/A	N/A
10398306	Wdr25	N/A	N/A
10398315	---	N/A	N/A
10398319	Dlk1	N/A	N/A
10398326	Meg3	N/A	N/A
10398332	Mir673	N/A	N/A
10398334	Mir337	N/A	N/A
10398336	Mir540	N/A	N/A
10398338	Mir665	N/A	N/A
10398340	Rtl1 /// Mir431	N/A	N/A
10398342	Rtl1 /// Mir433 /// 6430411K18Rik	N/A	N/A
10398344	Rtl1 /// Mir127	N/A	N/A
10398346	Rtl1 /// Mir434	N/A	N/A
10398348	Rtl1 /// Mir136	N/A	N/A
10398350	Mir341	N/A	N/A
10398352	Mir370	N/A	N/A
10398354	---	N/A	N/A
10398356	---	N/A	N/A
10398358	---	N/A	N/A
10398360	---	N/A	N/A
10398362	AF357355	N/A	N/A
10398364	---	N/A	N/A
10398366	---	N/A	N/A
10398368	---	N/A	N/A
10398370	---	N/A	N/A
10398372	---	N/A	N/A
10398374	AF357341	N/A	N/A
10398376	---	N/A	N/A
10398378	---	N/A	N/A
10398380	---	N/A	N/A
10398382	Mir379	N/A	N/A
10398384	Mir411	N/A	N/A
10398386	Mir299	N/A	N/A
10398388	Mir380	N/A	N/A
10398390	Mir323	N/A	N/A
10398392	Mir329	N/A	N/A
10398394	Mir494	N/A	N/A
10398396	Mir679	N/A	N/A
10398398	Mir666	N/A	N/A
10398400	Mir543	N/A	N/A
10398402	Mir495	N/A	N/A
10398404	Mir667	N/A	N/A
10398406	Mir376c	N/A	N/A
10398408	Mir376b	N/A	N/A
10398412	Mir300	N/A	N/A
10398414	Mir381	N/A	N/A
10398416	Mir487b	N/A	N/A
10398418	Mir539	N/A	N/A
10398420	Mir382	N/A	N/A
10398422	Mir134	N/A	N/A
10398424	Mir668	N/A	N/A

10398426	Mir485	N/A	N/A
10398428	Mir154	N/A	N/A
10398430	Mir496	N/A	N/A
10398434	Mir541	N/A	N/A
10398436	Mir409	N/A	N/A
10398438	Mir412	N/A	N/A
10398440	Mir369	N/A	N/A
10398442	Mir410 /// Mirg	N/A	N/A
10398444	---	N/A	N/A
10398451	Rps25 /// Gm4963	N/A	N/A
10398453	Ppp2r5c	N/A	N/A
10398455	Ppp2r5c	N/A	N/A
10398459	Ppp2r5c	N/A	N/A
10398461	Ppp2r5c	N/A	N/A
10398483	Dync1h1	N/A	N/A
10398578	Tecpr2	N/A	N/A
10398599	Rps19 /// Rps19-ps3 /// Gm6636	N/A	N/A
10398601	Rcor1	N/A	N/A
10398615	Gm10158	N/A	N/A
10398678	Eif5	N/A	N/A
10398693	Snora28	N/A	N/A
10398695	Mark3	N/A	N/A
10398717	Trmt61a	N/A	N/A
10398721	2810002N01Rik	N/A	N/A
10398727	Klc1	N/A	N/A
10398751	Zfyve21	N/A	N/A
10398795	Aspg	N/A	N/A
10398859	Adssl1	N/A	N/A
10398874	Siva1	N/A	N/A
10398881	Zbtb42	N/A	N/A
10398885	AW555464	N/A	N/A
10398929	Btbd6	N/A	N/A
10398934	Brf1	N/A	N/A
10398972	Mta1	N/A	N/A
10399005	Crip1	N/A	N/A
10399027	Adam6a	N/A	N/A
10399029	---	N/A	N/A
10399034	---	N/A	N/A
10399036	Uevld	N/A	N/A
10399038	Zfp386	N/A	N/A
10399046	Vipr2	N/A	N/A
10399061	Esyt2	N/A	N/A
10399121	Ptprn2	N/A	N/A
10399146	Mir153	N/A	N/A
10399198	Ncoa4 /// Gm6768	N/A	N/A
10399208	Tmem196	N/A	N/A
10399212	Tmem196	N/A	N/A
10399214	Rab10	N/A	N/A
10399224	1110002L01Rik /// Kif3c	N/A	N/A
10399228	---	N/A	N/A
10399232	---	N/A	N/A
10399234	Efr3b	N/A	N/A
10399254	Cenpo /// Adcy3	N/A	N/A
10399265	Ncoa1	N/A	N/A
10399290	4930417G10Rik	N/A	N/A
10399299	A830093I24Rik	N/A	N/A
10399337	Klhl29	N/A	N/A
10399352	---	N/A	N/A
10399354	---	N/A	N/A
10399357	Gdf7	N/A	N/A
10399376	---	N/A	N/A
10399379	Pgk1	N/A	N/A
10399383	Kcns3	N/A	N/A
10399387	Msgn1	N/A	N/A
10399389	1700034J04Rik	N/A	N/A
10399407	Vsnl1	N/A	N/A
10399415	---	N/A	N/A
10399419	Tubb2b /// Tubb2a-ps2	N/A	N/A
10399426	---	N/A	N/A
10399428	Snord118	N/A	N/A
10399430	Ddx1	N/A	N/A
10399457	Akr1b3 /// Gm6644	N/A	N/A
10399459	---	N/A	N/A
10399461	---	N/A	N/A
10399463	---	N/A	N/A
10399465	Fam84a	N/A	N/A
10399470	Trib2	N/A	N/A
10399555	Kcnf1	N/A	N/A
10399575	---	N/A	N/A
10399579	1700030C10Rik	N/A	N/A
10399581	---	N/A	N/A
10399584	G730007D18Rik	N/A	N/A
10399586	Zfp125	N/A	N/A
10399588	Zfp125	N/A	N/A

10399591	---	N/A	N/A
10399593	Itgb1bp1	N/A	N/A
10399605	Adam17	N/A	N/A
10399629	Ywhaq	N/A	N/A
10399632	F630048H11Rik	N/A	N/A
10399634	LOC100043371 /// 6030426L16Rik	N/A	N/A
10399636	Mrto4	N/A	N/A
10399640	LOC100043371	N/A	N/A
10399642	---	N/A	N/A
10399655	Gm4425	N/A	N/A
10399657	---	N/A	N/A
10399659	---	N/A	N/A
10399661	Gm10330	N/A	N/A
10399663	LOC100043371	N/A	N/A
10399666	LOC100043371 /// 9030624G23Rik	N/A	N/A
10399671	Hpcal1 /// 2410018L13Rik	N/A	N/A
10399675	---	N/A	N/A
10399677	Cox7a2l	N/A	N/A
10399689	---	N/A	N/A
10399691	Id2	N/A	N/A
10399696	Rnf144a	N/A	N/A
10399710	Rsad2	N/A	N/A
10399720	---	N/A	N/A
10399722	Gm9866	N/A	N/A
10399725	Sox11	N/A	N/A
10399760	Rps7 /// Gm9493	N/A	N/A
10399768	Ttc15	N/A	N/A
10399820	Acp1	N/A	N/A
10399823	---	N/A	N/A
10399825	Dld	N/A	N/A
10399841	Cbll1	N/A	N/A
10399852	---	N/A	N/A
10399892	Gpr22	N/A	N/A
10399897	Hbp1 /// Cog5	N/A	N/A
10399941	---	N/A	N/A
10399943	Cdhr3	N/A	N/A
10399973	Hdac9 /// LOC100504541	N/A	N/A
10400002	4921508M14Rik	N/A	N/A
10400004	Mir680-3	N/A	N/A
10400006	Ahr	N/A	N/A
10400023	Tspan13	N/A	N/A
10400052	Ispd	N/A	N/A
10400054	---	N/A	N/A
10400095	Ifrd1 /// Gm7008	N/A	N/A
10400109	Zfp277	N/A	N/A
10400124	---	N/A	N/A
10400131	Gm10165	N/A	N/A
10400135	---	N/A	N/A
10400137	Dnajb9	N/A	N/A
10400141	Zbed4 /// 4930529C04Rik	N/A	N/A
10400143	Stxbp6	N/A	N/A
10400153	Hmgb1	N/A	N/A
10400155	Nova1	N/A	N/A
10400157	Nova1	N/A	N/A
10400165	Tfb2m	N/A	N/A
10400170	Prkd1	N/A	N/A
10400189	---	N/A	N/A
10400191	Strn3	N/A	N/A
10400210	Hectd1	N/A	N/A
10400293	6530401N04Rik	N/A	N/A
10400299	Gpr33	N/A	N/A
10400302	---	N/A	N/A
10400304	Egln3	N/A	N/A
10400319	Gm10465	N/A	N/A
10400321	1110002B05Rik	N/A	N/A
10400324	---	N/A	N/A
10400334	Rpl31 /// Gm16382	N/A	N/A
10400336	Snx6	N/A	N/A
10400350	Cfl2	N/A	N/A
10400391	Polr2h	N/A	N/A
10400393	Fam177a /// 1700047I17Rik2 /// Gm9802	N/A	N/A
10400395	Ppp2r3c	N/A	N/A
10400405	Nfkbia	N/A	N/A
10400413	Ralgapa1	N/A	N/A
10400460	Mbip	N/A	N/A
10400470	Cox6c	N/A	N/A
10400504	Foxa1	N/A	N/A
10400508	Rpl26-ps2	N/A	N/A
10400510	Clec14a	N/A	N/A
10400515	Sec23a	N/A	N/A
10400538	Trappc6b	N/A	N/A
10400544	Rpl21	N/A	N/A
10400546	Fbxo33	N/A	N/A
10400555	---	N/A	N/A

10400557	---	N/A	N/A
10400559	---	N/A	N/A
10400562	---	N/A	N/A
10400570	---	N/A	N/A
10400572	Klhl28	N/A	N/A
10400581	Fkbp3 /// Prpf39	N/A	N/A
10400605	Rpl10l	N/A	N/A
10400607	Mdga2	N/A	N/A
10400609	Mdga2	N/A	N/A
10400628	---	N/A	N/A
10400630	---	N/A	N/A
10400635	Rps29	N/A	N/A
10400639	1110034A24Rik /// Rpl36al	N/A	N/A
10400642	1110034A24Rik /// 9330151L19Rik	N/A	N/A
10400704	---	N/A	N/A
10400706	Arf6 /// Gm9887	N/A	N/A
10400708	---	N/A	N/A
10400710	Gm71	N/A	N/A
10400718	Sos2	N/A	N/A
10400740	---	N/A	N/A
10400748	Cdkl1	N/A	N/A
10400760	Mir681	N/A	N/A
10400762	Map4k5	N/A	N/A
10400803	---	N/A	N/A
10400844	Pygl	N/A	N/A
10400866	Trim9	N/A	N/A
10400883	Timm9	N/A	N/A
10400892	---	N/A	N/A
10400894	Gpr135	N/A	N/A
10400896	2810055F11Rik	N/A	N/A
10400926	Rtn1	N/A	N/A
10400941	Dhrs7	N/A	N/A
10400948	4930447C04Rik	N/A	N/A
10400971	Six4	N/A	N/A
10400981	D830013O20Rik	N/A	N/A
10400984	Tmem30b	N/A	N/A
10400989	Kcnh5	N/A	N/A
10401007	Ppp2r5e	N/A	N/A
10401023	Wdr89 /// Rplp2	N/A	N/A
10401028	Sgpp1	N/A	N/A
10401050	---	N/A	N/A
10401063	Zbtb25	N/A	N/A
10401114	Rab15	N/A	N/A
10401128	Max	N/A	N/A
10401136	---	N/A	N/A
10401138	Atp6v1d	N/A	N/A
10401166	Pigh /// Plekhh1	N/A	N/A
10401172	Vti1b /// Arg2	N/A	N/A
10401244	Actn1	N/A	N/A
10401267	Dcaf5	N/A	N/A
10401278	Erh	N/A	N/A
10401283	---	N/A	N/A
10401286	Gm1568	N/A	N/A
10401289	Slc10a1	N/A	N/A
10401296	Slc8a3	N/A	N/A
10401309	Cox16	N/A	N/A
10401317	Gm4787	N/A	N/A
10401320	Adam4	N/A	N/A
10401322	Synj2bp /// Cox16	N/A	N/A
10401330	Adam21	N/A	N/A
10401343	Map3k9	N/A	N/A
10401359	Dpf3	N/A	N/A
10401365	Zfyve1	N/A	N/A
10401382	Numb	N/A	N/A
10401416	2410016O06Rik	N/A	N/A
10401418	4732463B04Rik	N/A	N/A
10401420	---	N/A	N/A
10401422	Pnma1	N/A	N/A
10401428	C130039O16Rik	N/A	N/A
10401441	Gm5436	N/A	N/A
10401443	Fam161b	N/A	N/A
10401454	Entpd5	N/A	N/A
10401473	Aldh6a1	N/A	N/A
10401511	Tmem90a	N/A	N/A
10401564	1110018G07Rik	N/A	N/A
10401595	Rps6kl1	N/A	N/A
10401616	Mlh3	N/A	N/A
10401637	Nek9	N/A	N/A
10401667	0610007P14Rik	N/A	N/A
10401684	Angel1	N/A	N/A
10401695	pl21 /// Gm6813 /// Gm9104 /// Rpl21-ps4 /// Rpl21-ps7 /// Gm16416 /// Rpl21-ps12 /// Rpl21-ps14 /// Rpl21-ps10 /// Gm80	N/A	N/A
10401698	6430527G18Rik	N/A	N/A
10401702	Zdhhc22	N/A	N/A
10401705	Zdhhc22	N/A	N/A

10401708	Ngb	N/A	N/A
10401713	Pomt2	N/A	N/A
10401737	Tmed8	N/A	N/A
10401743	4933437F05Rik	N/A	N/A
10401753	Vipar	N/A	N/A
10401781	Sptlc2	N/A	N/A
10401795	Nrp /// Alkbh1	N/A	N/A
10401803	---	N/A	N/A
10401805	Snw1	N/A	N/A
10401820	Gm16381	N/A	N/A
10401822	Gm4027 /// BB287469 /// Gm5662	N/A	N/A
10401829	BB287469 /// Eif1a /// Gm4027 /// Gm8300 /// Gm2022 /// Gm16368	N/A	N/A
10401882	Gtf2a1	N/A	N/A
10401891	Ston2	N/A	N/A
10401924	Rpl31 /// Rpl31-ps1 /// Gm16382	N/A	N/A
10401931	---	N/A	N/A
10401933	---	N/A	N/A
10401935	BC005685	N/A	N/A
10401937	---	N/A	N/A
10401939	Lysmd1	N/A	N/A
10401948	---	N/A	N/A
10401956	---	N/A	N/A
10401968	Galc	N/A	N/A
10401987	Kcnk10	N/A	N/A
10401997	Ptpn21	N/A	N/A
10402020	Eml5	N/A	N/A
10402061	Eml5	N/A	N/A
10402063	Foxn3	N/A	N/A
10402066	Foxn3	N/A	N/A
10402096	Ttc7b	N/A	N/A
10402117	Rps6ka5	N/A	N/A
10402136	Gpr68	N/A	N/A
10402140	Gpr68 /// Gm10431	N/A	N/A
10402179	Smek1	N/A	N/A
10402211	Fbln5	N/A	N/A
10402225	Trip11	N/A	N/A
10402262	Gm2695	N/A	N/A
10402266	---	N/A	N/A
10402268	Lgmn	N/A	N/A
10402283	Itpk1	N/A	N/A
10402294	Moap1 /// LOC100233175	N/A	N/A
10402302	Btbd7	N/A	N/A
10402314	Rps2 /// Rps2-ps6	N/A	N/A
10402316	Rpl29	N/A	N/A
10402334	9330161L09Rik	N/A	N/A
10402336	Ddx24	N/A	N/A
10402360	Serpina10	N/A	N/A
10402368	Serpina6	N/A	N/A
10402390	Serpina1a /// Serpina1b	N/A	N/A
10402394	Serpina1d	N/A	N/A
10402399	Serpina1e /// Serpina1d /// Serpina1c /// Serpina1b /// Serpina1a	N/A	N/A
10402406	Serpina1c	N/A	N/A
10402409	Serpina1a /// Serpina1b /// Serpina1c /// Serpina1e	N/A	N/A
10402415	Serpina11	N/A	N/A
10402440	Gsc	N/A	N/A
10402444	Dicer1	N/A	N/A
10402473	Clmn	N/A	N/A
10402512	Scarna13	N/A	N/A
10402519	Atg2b	N/A	N/A
10402542	Atg2b	N/A	N/A
10402554	Bcl11b	N/A	N/A
10402560	A130014H13Rik	N/A	N/A
10402570	Gm10427	N/A	N/A
10402572	Ccdc85c	N/A	N/A
10402579	Slc25a29	N/A	N/A
10402585	Wars	N/A	N/A
10402598	Begain	N/A	N/A
10402604	---	N/A	N/A
10402606	Rtl1 /// Mir431 /// 6430411K18Rik	N/A	N/A
10402615	Hsp90aa1	N/A	N/A
10402630	Stk30	N/A	N/A
10402648	Brp44l	N/A	N/A
10402650	Cinp	N/A	N/A
10402665	Cdc42bpb	N/A	N/A
10402703	1200009I06Rik /// Gm10425	N/A	N/A
10402705	Gm266	N/A	N/A
10402721	Xrcc3	N/A	N/A
10402730	Ppp1r13b	N/A	N/A
10402752	2010107E04Rik	N/A	N/A
10402761	Tmem179	N/A	N/A
10402766	Akt1	N/A	N/A
10402783	Ahnak2	N/A	N/A
10402835	Nudt14	N/A	N/A
10402841	Brf1	N/A	N/A

10402986	---	N/A	N/A
10402988	---	N/A	N/A
10402991	Igh-VX24 /// Gm16970	N/A	N/A
10403013	---	N/A	N/A
10403018	AI324046 /// IghmAC38.205.12	N/A	N/A
10403021	---	N/A	N/A
10403034	LOC100046275	N/A	N/A
10403043	Ighv1-72	N/A	N/A
10403046	AI324046	N/A	N/A
10403048	Ighv1-72 /// LOC631518	N/A	N/A
10403052	---	N/A	N/A
10403054	LOC435333	N/A	N/A
10403063	LOC100046275	N/A	N/A
10403071	---	N/A	N/A
10403076	---	N/A	N/A
10403079	LOC435333	N/A	N/A
10403108	Hmgn2	N/A	N/A
10403110	Gm10421	N/A	N/A
10403193	Sp4	N/A	N/A
10403229	Itgb8	N/A	N/A
10403244	---	N/A	N/A
10403246	---	N/A	N/A
10403258	Gdi2	N/A	N/A
10403281	Calm4	N/A	N/A
10403291	Akr1c14	N/A	N/A
10403303	Akr1c13	N/A	N/A
10403312	Akr1c19	N/A	N/A
10403322	Akr1c6	N/A	N/A
10403346	Rpl29 /// Gm3550 /// Rpl29-ps2	N/A	N/A
10403348	Gm5444	N/A	N/A
10403361	Pitrm1 /// Pfkp	N/A	N/A
10403392	---	N/A	N/A
10403394	---	N/A	N/A
10403396	Adarb2	N/A	N/A
10403413	Idi1	N/A	N/A
10403428	Larp4b	N/A	N/A
10403453	Dip2c	N/A	N/A
10403455	Dip2c	N/A	N/A
10403462	Dip2c	N/A	N/A
10403464	Dip2c	N/A	N/A
10403466	Dip2c	N/A	N/A
10403508	---	N/A	N/A
10403511	Heatr1	N/A	N/A
10403577	---	N/A	N/A
10403584	Nid1	N/A	N/A
10403604	Lyst	N/A	N/A
10403664	B3galnt2 /// Tbce	N/A	N/A
10403680	Arid4b	N/A	N/A
10403706	Psma2	N/A	N/A
10403716	AW209491	N/A	N/A
10403743	Inhba	N/A	N/A
10403748	---	N/A	N/A
10403754	Bat4	N/A	N/A
10403756	1600012F09Rik	N/A	N/A
10403761	---	N/A	N/A
10403765	Vps41	N/A	N/A
10403796	Amph	N/A	N/A
10403834	Sfrp4	N/A	N/A
10403842	Elmo1	N/A	N/A
10403897	---	N/A	N/A
10403899	Olfr263-ps1	N/A	N/A
10403903	Trim27	N/A	N/A
10403911	Gpx6	N/A	N/A
10403917	Olfr1367	N/A	N/A
10403929	Zkscan4 /// Gm10065	N/A	N/A
10403934	Isca1 /// AK157302	N/A	N/A
10403936	Olfr1359 /// Olfr1360	N/A	N/A
10403941	Hist1h3a /// Hist1h3i /// Hist1h3g /// Hist1h3h /// Hist1h3b /// Hist1h3d /// Hist1h3e	N/A	N/A
10403943	Hist1h2bm	N/A	N/A
10403948	Hist1h2bg	N/A	N/A
10403955	Hist1h2ai /// Hist1h2ad /// Hist1h2af /// Hist1h2ah /// Gm11276 /// Hist1h2ao /// Hist1h2an /// Hist1h2ak	N/A	N/A
10403957	Hist1h4m /// Hist1h4b /// Hist1h4f /// Hist1h4a /// Hist1h4i /// Gm11275	N/A	N/A
10403959	Gm11277	N/A	N/A
10403973	Pom121l2	N/A	N/A
10403978	Hist1h2bk	N/A	N/A
10403980	Gm11277	N/A	N/A
10403982	Vmn1r188	N/A	N/A
10403984	Vmn1r194	N/A	N/A
10403986	Vmn1r195	N/A	N/A
10403988	Vmn1r191 /// Vmn1r196 /// Vmn1r188 /// Vmn1r214 /// Vmn1r203 /// Vmn1r219 /// Vmn1r189	N/A	N/A
10403992	Vmn1r198	N/A	N/A
10403996	Vmn1r200	N/A	N/A
10403998	Vmn1r-ps103	N/A	N/A
10404002	Vmn1r203	N/A	N/A

10404004	Vmn1r223 /// Vmn1r204 /// Vmn1r193	N/A	N/A
10404008	Vmn1r213	N/A	N/A
10404010	Vmn1r214	N/A	N/A
10404014	Vmn1r216	N/A	N/A
10404016	Vmn1r218	N/A	N/A
10404018	Vmn1r219	N/A	N/A
10404020	Vmn1r221 /// Vmn1r-ps103 /// Vmn1r189	N/A	N/A
10404022	Vmn1r223 /// Vmn1r204 /// Vmn1r190-ps	N/A	N/A
10404024	Hist1h4h	N/A	N/A
10404026	Gm11276 /// Hist1h2ao /// Hist1h2ai /// Hist1h2ad /// Hist1h2af /// Hist1h2ah /// Hist1h2an /// Hist1h2ak	N/A	N/A
10404028	Hist1h3i /// Hist1h3g /// Hist1h3h /// Hist1h3b /// Hist1h3d /// Hist1h3e	N/A	N/A
10404033	Hist1h1d	N/A	N/A
10404036	Hist1h2bg	N/A	N/A
10404045	1276 /// Hist1h2ao /// Hist1h2ai /// Hist1h2ad /// Hist1h2af /// Hist1h2ah /// Hist1h2ae /// Hist1h2ag /// Hist1h2an /// Hist1	N/A	N/A
10404049	Hist1h3i /// Hist1h3h /// Hist1h3b /// Hist1h3d /// Hist1h3e	N/A	N/A
10404051	Hist1h4d	N/A	N/A
10404053	Hist1h2bc	N/A	N/A
10404061	Hist1h2bb	N/A	N/A
10404063	Hist1h2ab	N/A	N/A
10404065	Hist1h3i /// Hist1h3c /// Hist1h3h /// Hist1h3b /// Hist1h3d /// Hist1h3e /// Hist2h3b	N/A	N/A
10404067	Hist1h4m /// Hist1h4c /// Hist1h4a /// Hist1h4b /// Hist1h4f /// Gm11275	N/A	N/A
10404069	Hist1h1a	N/A	N/A
10404077	Slc17a2	N/A	N/A
10404097	Slc17a3	N/A	N/A
10404127	Hist1h2aa	N/A	N/A
10404129	Lrrc16a /// 4933427I18Rik	N/A	N/A
10404132	Cmah	N/A	N/A
10404152	Fam65b	N/A	N/A
10404187	Tdp2	N/A	N/A
10404195	D130043K22Rik	N/A	N/A
10404218	Gpld1	N/A	N/A
10404250	Dcdc2a	N/A	N/A
10404262	Rps8 /// Gm11353 /// Rps8-ps1	N/A	N/A
10404354	Rps18 /// Gm10260	N/A	N/A
10404376	Agtr1a	N/A	N/A
10404380	Dusp22	N/A	N/A
10404400	Gm5447	N/A	N/A
10404402	Foxq1	N/A	N/A
10404404	Foxf2	N/A	N/A
10404422	Serpinb6b	N/A	N/A
10404429	Serpinb9	N/A	N/A
10404447	Serpinb1b	N/A	N/A
10404496	Nqo2	N/A	N/A
10404506	Ripk1	N/A	N/A
10404521	Bphl	N/A	N/A
10404538	Prpf4b	N/A	N/A
10404575	---	N/A	N/A
10404593	---	N/A	N/A
10404595	Ppp1r3g	N/A	N/A
10404597	Fars2	N/A	N/A
10404606	Ly86	N/A	N/A
10404612	Rreb1	N/A	N/A
10404630	Riok1	N/A	N/A
10404649	Dsp	N/A	N/A
10404700	Ubxn2a	N/A	N/A
10404702	Gcnt2	N/A	N/A
10404717	Pak1ip1	N/A	N/A
10404731	Tmem14c	N/A	N/A
10404763	Tmem170b	N/A	N/A
10404772	Gm10790	N/A	N/A
10404774	Hivep1	N/A	N/A
10404792	Phactr1	N/A	N/A
10404815	Sirt5	N/A	N/A
10404827	Nol7	N/A	N/A
10404836	Rnf182	N/A	N/A
10404840	Cd83	N/A	N/A
10404870	---	N/A	N/A
10404872	Hspb1 /// Gm9817	N/A	N/A
10404874	Mylip	N/A	N/A
10404885	Gmpr	N/A	N/A
10404895	5033430I15Rik	N/A	N/A
10404913	Cap2	N/A	N/A
10404928	C78339	N/A	N/A
10404937	---	N/A	N/A
10404939	2010001K21Rik	N/A	N/A
10404941	Kdm1b	N/A	N/A
10404965	Rnf144b	N/A	N/A
10404975	Id4	N/A	N/A
10404996	Ninj1	N/A	N/A
10405001	Bicd2	N/A	N/A
10405074	Nol8	N/A	N/A
10405094	Iars	N/A	N/A
10405119	Gm270	N/A	N/A
10405121	2310081J21Rik	N/A	N/A

10405123	2310081J21Rik	N/A	N/A
10405154	Gm906 /// Gm904 /// Gm8765	N/A	N/A
10405158	Pus7	N/A	N/A
10405163	Spin1	N/A	N/A
10405174	Nxnl2	N/A	N/A
10405185	Cks2	N/A	N/A
10405189	---	N/A	N/A
10405211	Gadd45g	N/A	N/A
10405234	Snrpd2 /// Gm5449 /// Gm10120	N/A	N/A
10405236	Sfxn1	N/A	N/A
10405263	4732471D19Rik	N/A	N/A
10405280	Arl10	N/A	N/A
10405291	Faf2	N/A	N/A
10405355	Unc5a	N/A	N/A
10405372	Zfp346	N/A	N/A
10405380	Fgfr4	N/A	N/A
10405427	Prelid1 /// Rab24	N/A	N/A
10405462	BC038268 /// Pfn3	N/A	N/A
10405483	Prr7	N/A	N/A
10405504	Caml	N/A	N/A
10405535	B230219D22Rik	N/A	N/A
10405539	Txndc15	N/A	N/A
10405545	Pcbd2	N/A	N/A
10405566	Slc25a48	N/A	N/A
10405585	Gm10781	N/A	N/A
10405587	Tgfbi	N/A	N/A
10405605	Smad5	N/A	N/A
10405619	5133401N09Rik	N/A	N/A
10405626	Rpl17 /// Gm10268 /// Rpl17-ps3	N/A	N/A
10405633	Ntrk2	N/A	N/A
10405662	Naa35	N/A	N/A
10405725	---	N/A	N/A
10405727	2410127L17Rik	N/A	N/A
10405729	Selk	N/A	N/A
10405731	4933434E20Rik	N/A	N/A
10405733	6720457D02Rik /// Zfp808	N/A	N/A
10405739	Taf1d	N/A	N/A
10405741	---	N/A	N/A
10405745	---	N/A	N/A
10405749	---	N/A	N/A
10405751	Mir713	N/A	N/A
10405753	Me1	N/A	N/A
10405755	---	N/A	N/A
10405779	Mir23b	N/A	N/A
10405781	Mir27b	N/A	N/A
10405783	Mir24-1	N/A	N/A
10405785	0610007P08Rik	N/A	N/A
10405804	0610007P08Rik	N/A	N/A
10405811	Habp4	N/A	N/A
10405820	1810034E14Rik	N/A	N/A
10405822	Cdk20	N/A	N/A
10405833	---	N/A	N/A
10405835	---	N/A	N/A
10405847	---	N/A	N/A
10405849	Olfr466	N/A	N/A
10405851	Gm10775	N/A	N/A
10405853	Zfp369	N/A	N/A
10405866	Gm10139	N/A	N/A
10405868	Cbx3 /// Gm6901	N/A	N/A
10405870	Vmn2r122	N/A	N/A
10405874	Cbx3 /// Gm6901	N/A	N/A
10405876	Gm10324	N/A	N/A
10405885	---	N/A	N/A
10405888	Cbx3 /// Gm6901	N/A	N/A
10405890	Rpl9 /// Gm5451	N/A	N/A
10405894	Ptdss1	N/A	N/A
10405916	Zfp87	N/A	N/A
10405927	Zfp455	N/A	N/A
10405929	F630042J09Rik	N/A	N/A
10405935	Rplp0	N/A	N/A
10405938	Zfp273	N/A	N/A
10405942	Gm10037	N/A	N/A
10405948	BC048507	N/A	N/A
10405964	1700001L19Rik	N/A	N/A
10405969	---	N/A	N/A
10405994	Med10	N/A	N/A
10405999	8030423J24Rik	N/A	N/A
10406003	---	N/A	N/A
10406031	Lpcat1	N/A	N/A
10406067	Clptm1l	N/A	N/A
10406086	Tert	N/A	N/A
10406108	---	N/A	N/A
10406111	Slc12a7	N/A	N/A
10406139	---	N/A	N/A

10406141	Brd9	N/A	N/A
10406171	Tppp	N/A	N/A
10406198	Ftl2 /// Ftl1 /// Mir692-1	N/A	N/A
10406203	Stoml2	N/A	N/A
10406205	Erap1	N/A	N/A
10406226	---	N/A	N/A
10406229	Pcsk1	N/A	N/A
10406245	---	N/A	N/A
10406248	---	N/A	N/A
10406254	Ell2	N/A	N/A
10406270	Glrx	N/A	N/A
10406276	---	N/A	N/A
10406278	Rps2 /// Rps2-ps6 /// Gm5921 /// Gm6139	N/A	N/A
10406287	Ttc37	N/A	N/A
10406332	---	N/A	N/A
10406334	Mctp1	N/A	N/A
10406364	2210408I21Rik	N/A	N/A
10406385	Fam172a	N/A	N/A
10406399	Pou5f2	N/A	N/A
10406405	---	N/A	N/A
10406407	Arrdc3	N/A	N/A
10406417	Actg1	N/A	N/A
10406419	Lysmd3	N/A	N/A
10406423	Mblac2	N/A	N/A
10406452	Gm10759	N/A	N/A
10406456	AY512938	N/A	N/A
10406459	Ndufc1	N/A	N/A
10406461	Mir9-2	N/A	N/A
10406464	---	N/A	N/A
10406466	Tmem161b	N/A	N/A
10406482	Ccnh	N/A	N/A
10406499	Rpl21	N/A	N/A
10406501	---	N/A	N/A
10406504	Edil3	N/A	N/A
10406530	Tmem167	N/A	N/A
10406536	Tmem167	N/A	N/A
10406538	---	N/A	N/A
10406546	---	N/A	N/A
10406551	Ssbp2	N/A	N/A
10406564	Acot12	N/A	N/A
10406581	Msh3 /// Dhfr	N/A	N/A
10406598	Serinc5	N/A	N/A
10406622	Gm4814 /// Cmya5	N/A	N/A
10406626	Homer1	N/A	N/A
10406646	Dmgdh	N/A	N/A
10406663	Arsb	N/A	N/A
10406672	Arsb	N/A	N/A
10406681	Ap3b1	N/A	N/A
10406710	Tbca	N/A	N/A
10406714	Otp	N/A	N/A
10406718	Wdr41	N/A	N/A
10406733	Zbed3	N/A	N/A
10406736	F2rl2	N/A	N/A
10406740	---	N/A	N/A
10406742	Poc5	N/A	N/A
10406757	Col4a3bp	N/A	N/A
10406777	Gcnt4	N/A	N/A
10406782	Fam169a	N/A	N/A
10406795	Gfm2	N/A	N/A
10406817	Enc1	N/A	N/A
10406823	---	N/A	N/A
10406843	Btf3	N/A	N/A
10406852	Cnn3	N/A	N/A
10406854	Rpl27a /// Gm5908 /// Rpl27a-ps1 /// Gm5453	N/A	N/A
10406856	Gm807	N/A	N/A
10406859	Zfp366	N/A	N/A
10406865	Mrps27	N/A	N/A
10406877	Serf1	N/A	N/A
10406881	Smn1	N/A	N/A
10406898	Taf9	N/A	N/A
10406934	Etv1 /// Gm5454	N/A	N/A
10406939	Rpl21 /// Rpl21-ps7 /// Gm16416	N/A	N/A
10406941	Sgtb	N/A	N/A
10406953	Trim23	N/A	N/A
10407034	Htr1a	N/A	N/A
10407040	Esco1	N/A	N/A
10407042	Dimt1	N/A	N/A
10407049	3830408C21Rik	N/A	N/A
10407051	---	N/A	N/A
10407072	Elovl7	N/A	N/A
10407097	Pde4d	N/A	N/A
10407120	Rps3a /// Gm9000	N/A	N/A
10407122	---	N/A	N/A
10407124	AI452195	N/A	N/A

10407126	Plk2	N/A	N/A
10407159	Ankrd55	N/A	N/A
10407173	Il6st	N/A	N/A
10407192	Slc38a9	N/A	N/A
10407209	Slc38a9	N/A	N/A
10407211	Ppap2a	N/A	N/A
10407251	Ccno	N/A	N/A
10407274	Mir449c	N/A	N/A
10407276	Mir449a	N/A	N/A
10407286	BC067074	N/A	N/A
10407291	Arl15	N/A	N/A
10407307	Mocs2	N/A	N/A
10407316	Pelo /// Gm10734	N/A	N/A
10407319	Rpl34 /// Gm6404 /// Gm10154	N/A	N/A
10407325	Hmgb1	N/A	N/A
10407327	Emb	N/A	N/A
10407337	Hcn1	N/A	N/A
10407346	---	N/A	N/A
10407348	Mrps30	N/A	N/A
10407356	Nnt /// Gm9777	N/A	N/A
10407358	Paip1	N/A	N/A
10407370	4833420G17Rik /// Gm9752	N/A	N/A
10407387	Gm7120	N/A	N/A
10407390	Ptbp1	N/A	N/A
10407392	BC016423	N/A	N/A
10407445	Akr1c12	N/A	N/A
10407454	Gm9973	N/A	N/A
10407456	Akr1c20	N/A	N/A
10407467	Akr1e1	N/A	N/A
10407481	Pfkp	N/A	N/A
10407509	---	N/A	N/A
10407511	---	N/A	N/A
10407513	Wdr37	N/A	N/A
10407533	---	N/A	N/A
10407535	Rpl10a /// Rpl10a-ps2	N/A	N/A
10407543	Gtpbp4	N/A	N/A
10407563	---	N/A	N/A
10407568	---	N/A	N/A
10407570	Zmynd11	N/A	N/A
10407591	Chrm3	N/A	N/A
10407598	Ryr2	N/A	N/A
10407707	Gm10336	N/A	N/A
10407766	Lgals8	N/A	N/A
10407792	Gpr137b-ps /// Gpr137b	N/A	N/A
10407801	Gm10811	N/A	N/A
10407809	---	N/A	N/A
10407811	Hnrnpf	N/A	N/A
10407814	Tbce	N/A	N/A
10407833	Ggps1	N/A	N/A
10407841	Hecw1	N/A	N/A
10407870	Mrpl32	N/A	N/A
10407892	Cdk13	N/A	N/A
10407907	Rala	N/A	N/A
10407912	---	N/A	N/A
10407916	1600012F09Rik	N/A	N/A
10407921	Vdac3	N/A	N/A
10407924	Bat4	N/A	N/A
10407926	Vdac3	N/A	N/A
10407929	Pou6f2	N/A	N/A
10407946	Stard3nl	N/A	N/A
10407955	Epdr1	N/A	N/A
10407995	Olfr1370	N/A	N/A
10408006	---	N/A	N/A
10408008	---	N/A	N/A
10408032	Zfp187	N/A	N/A
10408047	Zfp389	N/A	N/A
10408049	Zfp192	N/A	N/A
10408056	Olfr1366	N/A	N/A
10408058	Olfr1535	N/A	N/A
10408060	Olfr1364	N/A	N/A
10408062	Olfr1362	N/A	N/A
10408066	Olfr1361	N/A	N/A
10408068	Olfr1359 /// Olfr1360	N/A	N/A
10408070	Hist1h2bc	N/A	N/A
10408072	Hist1h2ai	N/A	N/A
10408074	Hist1h4m	N/A	N/A
10408081	Hist1h1b	N/A	N/A
10408083	Hist1h3i /// Hist1h3g /// Hist1h3h /// Hist1h3b /// Hist1h3d /// Hist1h3e	N/A	N/A
10408085	Gm11276 /// Hist1h2ao /// Hist1h2ai /// Hist1h2ad /// Hist1h2af /// Hist1h2ah /// Hist1h2ag /// Hist1h2an /// Hist1h2ak	N/A	N/A
10408087	Gm11277	N/A	N/A
10408092	Hist1h4i /// Hist1h4m /// Hist1h4f /// Hist1h4a /// Hist1h4b /// Gm11275	N/A	N/A
10408094	Hist1h2ai /// Hist1h2ad /// Hist1h2af /// Hist1h2ah /// Gm11276 /// Hist1h2ao /// Hist1h2an /// Hist1h2ak	N/A	N/A
10408111	Hist1h2ai /// Gm11276 /// Hist1h2ao /// Hist1h2af /// Hist1h2ah /// Hist1h2an /// Hist1h2ad /// Hist1h2ak	N/A	N/A
10408113	Hist1h4i	N/A	N/A

10408121	---	N/A	N/A
10408123	Vmn1r189	N/A	N/A
10408125	Vmn1r190-ps	N/A	N/A
10408130	Vmn1r191	N/A	N/A
10408132	Vmn1r192	N/A	N/A
10408134	Vmn1r193	N/A	N/A
10408138	Vmn1r202	N/A	N/A
10408142	Vmn1r206	N/A	N/A
10408144	Vmn1r208	N/A	N/A
10408146	Vmn1r208	N/A	N/A
10408148	Vmn1r206 /// Vmn1r207-ps /// Vmn1r209	N/A	N/A
10408152	Vmn1r211	N/A	N/A
10408154	Vmn1r212	N/A	N/A
10408156	Vmn1r217	N/A	N/A
10408158	Vmn1r220	N/A	N/A
10408162	Zfp322a	N/A	N/A
10408168	Abt1	N/A	N/A
10408173	---	N/A	N/A
10408200	Hist1h4f	N/A	N/A
10408202	Hist1h3f /// Hist1h3i /// Hist1h3h /// Hist1h3b /// Hist1h3d /// Hist1h3e	N/A	N/A
10408210	Hist1h2bf	N/A	N/A
10408212	Hist1h1e /// Hist1h2be	N/A	N/A
10408223	Hist1h2bc	N/A	N/A
10408225	Hist1h4c	N/A	N/A
10408227	Hfe	N/A	N/A
10408239	Hist1h3f /// Hist1h3i /// Hist1h3c /// Hist1h3b /// Hist1h3d /// Hist1h3e	N/A	N/A
10408243	Hist1h4m /// Hist1h4a /// Hist1h4f /// Hist1h4b /// Gm11275	N/A	N/A
10408249	---	N/A	N/A
10408266	Hist1h2ba	N/A	N/A
10408280	Lrrc16a	N/A	N/A
10408329	---	N/A	N/A
10408331	Acot13	N/A	N/A
10408335	Aldh5a1	N/A	N/A
10408346	Gm9983	N/A	N/A
10408348	Mrs2	N/A	N/A
10408359	Nrsn1	N/A	N/A
10408450	Sox4	N/A	N/A
10408455	Cdkal1	N/A	N/A
10408477	E2f3	N/A	N/A
10408485	---	N/A	N/A
10408487	Uqcrfs1	N/A	N/A
10408490	Exoc2	N/A	N/A
10408519	Hus1b	N/A	N/A
10408523	Foxq1	N/A	N/A
10408529	---	N/A	N/A
10408531	Gmds	N/A	N/A
10408541	---	N/A	N/A
10408555	Wrnip1	N/A	N/A
10408572	---	N/A	N/A
10408574	---	N/A	N/A
10408600	Serpinb6a	N/A	N/A
10408610	Tubb2a	N/A	N/A
10408613	Tubb2b	N/A	N/A
10408616	Slc22a23	N/A	N/A
10408629	1300014I06Rik	N/A	N/A
10408636	---	N/A	N/A
10408656	Peci	N/A	N/A
10408677	Lyrm4	N/A	N/A
10408684	Tmed10	N/A	N/A
10408687	Hmgb1	N/A	N/A
10408689	Nrn1	N/A	N/A
10408709	Ssr1	N/A	N/A
10408739	Dsp /// Gm10129	N/A	N/A
10408741	Txndc5	N/A	N/A
10408762	Eef1e1	N/A	N/A
10408787	---	N/A	N/A
10408810	---	N/A	N/A
10408812	Mak	N/A	N/A
10408838	Elovl2	N/A	N/A
10408848	---	N/A	N/A
10408850	Nedd9	N/A	N/A
10408870	Tbc1d7	N/A	N/A
10408879	Gfod1	N/A	N/A
10408882	Ranbp9	N/A	N/A
10408898	---	N/A	N/A
10408900	---	N/A	N/A
10408915	Dtnbp1	N/A	N/A
10408926	---	N/A	N/A
10408928	Hspb1 /// Gm9817	N/A	N/A
10408932	---	N/A	N/A
10408935	Gm10786	N/A	N/A
10408937	Atxn1	N/A	N/A
10408947	Gm10113	N/A	N/A
10408950	---	N/A	N/A

10408952	Nup153	N/A	N/A
10409014	---	N/A	N/A
10409016	Nhlrc1	N/A	N/A
10409059	Mirlet7d	N/A	N/A
10409061	Mirlet7f-1	N/A	N/A
10409063	Mirlet7a-1	N/A	N/A
10409065	Ptpdc1	N/A	N/A
10409076	Phf2	N/A	N/A
10409099	Fam120a	N/A	N/A
10409118	Wnk2	N/A	N/A
10409200	Gapdh /// Gm7251	N/A	N/A
10409202	---	N/A	N/A
10409204	---	N/A	N/A
10409206	---	N/A	N/A
10409208	Gm906 /// Gm904 /// Gm8765	N/A	N/A
10409212	---	N/A	N/A
10409214	Mir683-2 /// Mir683-1	N/A	N/A
10409216	Mir683-2 /// Mir683-1	N/A	N/A
10409218	2310081J21Rik	N/A	N/A
10409220	Hist2h2aa1 /// Hist2h2aa2 /// Hist2h3c1 /// Hist2h2ac	N/A	N/A
10409222	Shc3	N/A	N/A
10409236	---	N/A	N/A
10409240	Sema4d	N/A	N/A
10409259	---	N/A	N/A
10409261	Diras2	N/A	N/A
10409294	Sptlc1	N/A	N/A
10409319	Drd1a	N/A	N/A
10409322	Thoc3	N/A	N/A
10409330	4833439L19Rik	N/A	N/A
10409338	Nop16	N/A	N/A
10409345	Cltb	N/A	N/A
10409352	Rnf44	N/A	N/A
10409365	Gprin1	N/A	N/A
10409369	Sncb	N/A	N/A
10409414	Rab24	N/A	N/A
10409434	Lman2	N/A	N/A
10409445	BC038268 /// Pfn3	N/A	N/A
10409449	F12	N/A	N/A
10409464	Dbn1	N/A	N/A
10409486	Pdlim7	N/A	N/A
10409508	Ddx41	N/A	N/A
10409526	Fam193b	N/A	N/A
10409539	---	N/A	N/A
10409541	---	N/A	N/A
10409543	---	N/A	N/A
10409557	H2afy	N/A	N/A
10409592	Lect2	N/A	N/A
10409599	---	N/A	N/A
10409602	Trpc7	N/A	N/A
10409616	Spock1	N/A	N/A
10409629	Klhl3	N/A	N/A
10409643	Hnrnpa0	N/A	N/A
10409660	Gkap1	N/A	N/A
10409684	2210016F16Rik	N/A	N/A
10409689	Hnrnpk	N/A	N/A
10409709	Mir7-1	N/A	N/A
10409711	---	N/A	N/A
10409737	Agtpbp1	N/A	N/A
10409799	Isca1 /// AK157302	N/A	N/A
10409804	Zcchc6	N/A	N/A
10409966	Mir713	N/A	N/A
10409968	Rps18	N/A	N/A
10409978	6720457D02Rik /// Gm3604 /// Zfp808 /// 6720489N17Rik	N/A	N/A
10409986	4933434E20Rik	N/A	N/A
10409988	Taf1d	N/A	N/A
10409990	6720489N17Rik	N/A	N/A
10409992	---	N/A	N/A
10409994	Gm5665	N/A	N/A
10410039	Ptch1	N/A	N/A
10410063	Rpl10a /// Rpl10a-ps2	N/A	N/A
10410078	Slc35d2	N/A	N/A
10410099	Cdc14b	N/A	N/A
10410115	1110018J18Rik	N/A	N/A
10410124	Ctsl	N/A	N/A
10410134	---	N/A	N/A
10410138	Cntnap3	N/A	N/A
10410148	Cntnap3	N/A	N/A
10410150	Cntnap3	N/A	N/A
10410164	Cntnap3	N/A	N/A
10410166	Cntnap3	N/A	N/A
10410173	Hiatl1	N/A	N/A
10410204	---	N/A	N/A
10410207	Cbx3 /// Gm6901	N/A	N/A
10410209	Rybp	N/A	N/A

10410235	---	N/A	N/A
10410252	---	N/A	N/A
10410259	Uqcrb /// Gm5457	N/A	N/A
10410264	Mterfd1 /// Ptdss1	N/A	N/A
10410273	Zfp708	N/A	N/A
10410287	Zfp458	N/A	N/A
10410291	Zfp457	N/A	N/A
10410295	Zfp595	N/A	N/A
10410309	---	N/A	N/A
10410328	Zfp874b	N/A	N/A
10410332	Zfp58	N/A	N/A
10410341	Zfp87	N/A	N/A
10410345	Zfp748	N/A	N/A
10410351	AA987161	N/A	N/A
10410355	A530054K11Rik	N/A	N/A
10410362	Zfp738	N/A	N/A
10410364	Zfp738	N/A	N/A
10410375	Zfp85-rs1	N/A	N/A
10410386	E430024C06Rik	N/A	N/A
10410388	E430024C06Rik	N/A	N/A
10410390	---	N/A	N/A
10410393	Mtrr	N/A	N/A
10410408	Adcy2	N/A	N/A
10410435	Papd7	N/A	N/A
10410449	A530095I07Rik	N/A	N/A
10410452	Srd5a1	N/A	N/A
10410460	Ube2ql1	N/A	N/A
10410465	BC018507	N/A	N/A
10410475	---	N/A	N/A
10410477	Adamts16	N/A	N/A
10410506	Rpl9 /// Gm5451	N/A	N/A
10410508	Ndufs6	N/A	N/A
10410590	Exoc3	N/A	N/A
10410617	Pdcd6	N/A	N/A
10410625	Sdha	N/A	N/A
10410641	Lrrc14b	N/A	N/A
10410644	Zfp72	N/A	N/A
10410650	Zfp825	N/A	N/A
10410654	---	N/A	N/A
10410656	Cast	N/A	N/A
10410687	---	N/A	N/A
10410695	Rhobtb3	N/A	N/A
10410709	Rfesd	N/A	N/A
10410721	Arsk	N/A	N/A
10410741	Mctp1 /// Gm10760	N/A	N/A
10410743	Ankrd32	N/A	N/A
10410756	Ankrd32	N/A	N/A
10410766	Nr2f1	N/A	N/A
10410772	Rpl21	N/A	N/A
10410774	---	N/A	N/A
10410776	---	N/A	N/A
10410877	Polr3g	N/A	N/A
10410887	Rps2	N/A	N/A
10410889	---	N/A	N/A
10410892	Rasa1	N/A	N/A
10410919	---	N/A	N/A
10410921	Cox7c /// Gm10012	N/A	N/A
10410925	---	N/A	N/A
10410927	LOC280487	N/A	N/A
10410929	Agk	N/A	N/A
10410931	Vcan	N/A	N/A
10410947	Xrcc4	N/A	N/A
10410959	Atg10	N/A	N/A
10410970	Hnrnpk	N/A	N/A
10410973	4833422C13Rik	N/A	N/A
10410975	Zcchc9	N/A	N/A
10410995	Rasgrf2	N/A	N/A
10411052	Fam151b	N/A	N/A
10411059	Zfyve16	N/A	N/A
10411105	Gm10753	N/A	N/A
10411119	Papd4	N/A	N/A
10411126	Jmy	N/A	N/A
10411142	Bhmt	N/A	N/A
10411147	Bhmt2	N/A	N/A
10411156	Scamp1	N/A	N/A
10411167	---	N/A	N/A
10411169	Gm9776	N/A	N/A
10411171	Pde8b	N/A	N/A
10411215	Crhbp	N/A	N/A
10411229	F2r	N/A	N/A
10411235	Iqgap2	N/A	N/A
10411272	---	N/A	N/A
10411274	Sv2c	N/A	N/A
10411287	Btf3l4	N/A	N/A

10411363	Nsa2	N/A	N/A
10411373	Hexb	N/A	N/A
10411390	---	N/A	N/A
10411393	Rps18 /// Gm10260	N/A	N/A
10411432	Utp15	N/A	N/A
10411452	Gapdh /// Gm2451	N/A	N/A
10411454	Sec61b	N/A	N/A
10411459	Tmem171	N/A	N/A
10411464	Fcho2	N/A	N/A
10411502	1700024P04Rik	N/A	N/A
10411506	---	N/A	N/A
10411508	Ptcd2	N/A	N/A
10411519	Mtap1b	N/A	N/A
10411527	Cartpt	N/A	N/A
10411532	Mccc2	N/A	N/A
10411552	Bdp1	N/A	N/A
10411593	Serf1	N/A	N/A
10411609	---	N/A	N/A
10411709	Cdk7	N/A	N/A
10411711	Cdk7	N/A	N/A
10411722	Mrps36	N/A	N/A
10411774	---	N/A	N/A
10411776	H3f3a /// Gm12657	N/A	N/A
10411780	---	N/A	N/A
10411782	Pik3r1	N/A	N/A
10411839	Srsf12	N/A	N/A
10411853	Erbb2ip	N/A	N/A
10411882	Nln	N/A	N/A
10411899	2410002O22Rik	N/A	N/A
10411915	Ppwd1	N/A	N/A
10411927	Cwc27	N/A	N/A
10411945	Fam159b	N/A	N/A
10411949	Rgs7bp	N/A	N/A
10411958	Rnf180	N/A	N/A
10411970	Dph3b-ps	N/A	N/A
10411972	---	N/A	N/A
10411974	Ipo11	N/A	N/A
10412011	Kif2a	N/A	N/A
10412036	Apoo /// Apoo-ps	N/A	N/A
10412038	Zswim6	N/A	N/A
10412054	Ndufaf2	N/A	N/A
10412060	---	N/A	N/A
10412062	---	N/A	N/A
10412064	---	N/A	N/A
10412066	Rab3c	N/A	N/A
10412076	Gemin8	N/A	N/A
10412082	Gpbp1	N/A	N/A
10412098	Rpl41	N/A	N/A
10412100	Map3k1	N/A	N/A
10412123	Ncf2	N/A	N/A
10412177	Skiv2l2	N/A	N/A
10412205	Gm10735	N/A	N/A
10412207	Gpx8	N/A	N/A
10412227	Snx18	N/A	N/A
10412231	Hspb3	N/A	N/A
10412251	Ndufs4	N/A	N/A
10412258	---	N/A	N/A
10412296	---	N/A	N/A
10412298	Itga1	N/A	N/A
10412328	Pelo	N/A	N/A
10412335	Isl1	N/A	N/A
10412345	Parp8	N/A	N/A
10412376	Emb	N/A	N/A
10412378	H3f3a	N/A	N/A
10412380	---	N/A	N/A
10412385	Mrps30	N/A	N/A
10412392	Gm10732	N/A	N/A
10412394	Nnt	N/A	N/A
10412414	4833420G17Rik /// Gm9752	N/A	N/A
10412417	3110070M22Rik /// Gm7120	N/A	N/A
10412466	Hmgcs1	N/A	N/A
10412481	2410127L17Rik	N/A	N/A
10412491	---	N/A	N/A
10412517	Gm3002 /// Gm10021 /// Gm3512	N/A	N/A
10412537	Gm3373	N/A	N/A
10412543	m1973 /// LOC100041151 /// Gm5797 /// Gm5458 /// Gm3047 /// Gm3187 /// Gm3366 /// Gm10413 /// Gm10338 /// Gm80[illegible]	N/A	N/A
10412549	D830030K20Rik /// Gm3033 /// Gm3278 /// LOC100504200 /// Gm8050 /// Gm1973	N/A	N/A
10412559	Slbp	N/A	N/A
10412562	Flnb	N/A	N/A
10412607	Abhd6	N/A	N/A
10412616	Rpp14	N/A	N/A
10412624	Pxk	N/A	N/A
10412650	Kctd6	N/A	N/A
10412655	2610318M16Rik	N/A	N/A

10412657	---	N/A	N/A
10412659	---	N/A	N/A
10412661	---	N/A	N/A
10412663	---	N/A	N/A
10412665	Rpl21-ps4	N/A	N/A
10412667	Ptprg	N/A	N/A
10412699	Gm10404	N/A	N/A
10412701	3830406C13Rik	N/A	N/A
10412711	Uqcrb /// Gm5457	N/A	N/A
10412729	Sntn	N/A	N/A
10412741	Atxn7	N/A	N/A
10412755	---	N/A	N/A
10412760	Il3ra	N/A	N/A
10412771	---	N/A	N/A
10412773	Slc4a7	N/A	N/A
10412805	Nek10	N/A	N/A
10412823	Lrrc3b	N/A	N/A
10412826	Gm10403	N/A	N/A
10412830	Ngly1	N/A	N/A
10412844	Top2b	N/A	N/A
10412882	Thrb	N/A	N/A
10412897	---	N/A	N/A
10412907	---	N/A	N/A
10412909	Fdft1	N/A	N/A
10412911	---	N/A	N/A
10412919	---	N/A	N/A
10412943	Nudt13	N/A	N/A
10412960	Fam149b	N/A	N/A
10412978	1810062O18Rik	N/A	N/A
10412981	Sec24c	N/A	N/A
10413008	Fut11	N/A	N/A
10413012	Fut11	N/A	N/A
10413014	Chchd1	N/A	N/A
10413018	2310021P13Rik	N/A	N/A
10413059	Vcl	N/A	N/A
10413086	Adk	N/A	N/A
10413100	Myst4	N/A	N/A
10413136	---	N/A	N/A
10413138	Vdac2	N/A	N/A
10413148	Comtd1	N/A	N/A
10413161	Cnot1	N/A	N/A
10413174	Rps24 /// Gm6030	N/A	N/A
10413183	Gm10398	N/A	N/A
10413185	Zmiz1	N/A	N/A
10413216	4931406H21Rik	N/A	N/A
10413220	Zmiz1 /// Gm10397	N/A	N/A
10413222	Ppif	N/A	N/A
10413229	Anxa11	N/A	N/A
10413238	Cphx	N/A	N/A
10413243	Gm10394 /// Duxbl	N/A	N/A
10413250	Cphx	N/A	N/A
10413255	Gm10394 /// Duxbl	N/A	N/A
10413260	Cphx	N/A	N/A
10413265	Gm10394 /// Duxbl	N/A	N/A
10413280	---	N/A	N/A
10413282	Fam116a	N/A	N/A
10413304	Arf4	N/A	N/A
10413314	Pde12	N/A	N/A
10413316	---	N/A	N/A
10413333	Dnahc12	N/A	N/A
10413416	Il17rd	N/A	N/A
10413419	Arhgef3	N/A	N/A
10413434	D14Abb1e	N/A	N/A
10413461	Erc2	N/A	N/A
10413482	Wnt5a	N/A	N/A
10413492	Lrtm1	N/A	N/A
10413502	Actr8	N/A	N/A
10413517	Chdh	N/A	N/A
10413530	Dcp1a	N/A	N/A
10413559	Rft1	N/A	N/A
10413574	Sfmbt1	N/A	N/A
10413596	Sfmbt1	N/A	N/A
10413598	Tmem110	N/A	N/A
10413615	Itih4	N/A	N/A
10413657	Glt8d1	N/A	N/A
10413670	Pbrm1	N/A	N/A
10413695	Pbrm1	N/A	N/A
10413697	Pbrm1	N/A	N/A
10413710	Nt5dc2 /// Stab1	N/A	N/A
10413726	Tnnc1	N/A	N/A
10413752	Bap1	N/A	N/A
10413771	Capn7	N/A	N/A
10413795	Eaf1	N/A	N/A
10413803	Btd	N/A	N/A

10413808	---	N/A	N/A
10413813	Galntl2	N/A	N/A
10413826	Oxnad1	N/A	N/A
10413839	Ncoa4 /// Gm6768	N/A	N/A
10413853	Parg	N/A	N/A
10413874	Ogdhl	N/A	N/A
10413897	Ercc6	N/A	N/A
10413928	1810011H11Rik	N/A	N/A
10413930	Fam170b	N/A	N/A
10413949	Gm6340	N/A	N/A
10413977	Gm626	N/A	N/A
10413979	Gm626	N/A	N/A
10413981	Gm626	N/A	N/A
10413983	Gm626	N/A	N/A
10413985	Gm626	N/A	N/A
10413987	Gm626	N/A	N/A
10413989	Gm626	N/A	N/A
10413991	Gm626	N/A	N/A
10413993	Gm626	N/A	N/A
10413995	Gm626	N/A	N/A
10413997	Gm626	N/A	N/A
10413999	Gm626	N/A	N/A
10414001	Gm626	N/A	N/A
10414003	Gm626	N/A	N/A
10414025	Gdf10	N/A	N/A
10414031	Gdf2	N/A	N/A
10414093	Glud1	N/A	N/A
10414113	Wapal	N/A	N/A
10414137	Grid1	N/A	N/A
10414154	Mir346	N/A	N/A
10414161	---	N/A	N/A
10414163	Lrit1	N/A	N/A
10414173	---	N/A	N/A
10414175	---	N/A	N/A
10414192	Mat1a	N/A	N/A
10414211	Mbl1	N/A	N/A
10414218	1700049E17Rik1	N/A	N/A
10414228	Gm5622 /// Gm5930 /// Gm3371	N/A	N/A
10414234	Gm5622 /// Gm5930 /// Gm3371	N/A	N/A
10414241	Gm5930 /// Gm3371	N/A	N/A
10414245	Gm3161 /// 1700049E17Rik1 /// 1700049E17Rik2 /// 4930503E14Rik /// Gm7233 /// Gm8032	N/A	N/A
10414256	Rbmxrt	N/A	N/A
10414260	Ang5	N/A	N/A
10414262	Ear2 /// BC151093	N/A	N/A
10414265	BC061237	N/A	N/A
10414278	Gpr137c	N/A	N/A
10414288	Psmc6	N/A	N/A
10414313	Ubb /// Gm1821	N/A	N/A
10414325	Cgrrf1	N/A	N/A
10414333	Samd4	N/A	N/A
10414355	Mapk1ip1l	N/A	N/A
10414366	Fbxo34	N/A	N/A
10414372	Gm6616	N/A	N/A
10414374	Ktn1	N/A	N/A
10414417	Peli2	N/A	N/A
10414431	---	N/A	N/A
10414433	6720456H20Rik	N/A	N/A
10414449	Mudeng	N/A	N/A
10414460	Naa30	N/A	N/A
10414475	Olfr738	N/A	N/A
10414479	Olfr740	N/A	N/A
10414481	Olfr741	N/A	N/A
10414485	Olfr265	N/A	N/A
10414487	Olfr744	N/A	N/A
10414491	Olfr746	N/A	N/A
10414493	Olfr748	N/A	N/A
10414514	Pnp	N/A	N/A
10414522	Apex1 /// Tmem55b	N/A	N/A
10414527	Pnp /// Pnp2	N/A	N/A
10414537	Rnase4 /// Ang	N/A	N/A
10414551	Ear5	N/A	N/A
10414553	Ear14 /// Ear4	N/A	N/A
10414594	Mett11d1	N/A	N/A
10414612	Slc39a2	N/A	N/A
10414651	G630016D24Rik	N/A	N/A
10414659	Snord58b	N/A	N/A
10414661	---	N/A	N/A
10414693	Tox4	N/A	N/A
10414697	---	N/A	N/A
10414700	Olfr1509	N/A	N/A
10414703	Gm10886	N/A	N/A
10414706	Gm8635	N/A	N/A
10414711	Gm8639	N/A	N/A
10414717	Gm5772	N/A	N/A

10414720	---	N/A	N/A
10414723	Gm13955	N/A	N/A
10414726	---	N/A	N/A
10414728	Gm7174	N/A	N/A
10414734	Gm13959	N/A	N/A
10414736	---	N/A	N/A
10414738	LOC547323	N/A	N/A
10414741	Gm8736 /// Gm8800	N/A	N/A
10414744	Gm13970	N/A	N/A
10414751	Trav13n-1 /// Trav13-3 /// Gm13978 /// Gm6033 /// Gm17006	N/A	N/A
10414757	Gm13979 /// Gm16591	N/A	N/A
10414767	Rps19 /// Rps19-ps3 /// Gm6636	N/A	N/A
10414769	Tcra-V8	N/A	N/A
10414772	Tcra-V8 /// Gm10890	N/A	N/A
10414775	Trav13d-4 /// Gm6033 /// Gm17006	N/A	N/A
10414781	Gm10891 /// Gm13926	N/A	N/A
10414784	Gm26	N/A	N/A
10414790	---	N/A	N/A
10414793	C920008G01Rik	N/A	N/A
10414796	---	N/A	N/A
10414805	Trav13d-4	N/A	N/A
10414814	Gm1220 /// Gm13969	N/A	N/A
10414863	Gm13979 /// Gm16591	N/A	N/A
10414874	Rps19 /// Rps19-ps3 /// Gm6636	N/A	N/A
10414876	Tcra-V8	N/A	N/A
10414879	Tcra-V8 /// Gm10890	N/A	N/A
10414882	Trav13d-4 /// Gm6033 /// Gm17006	N/A	N/A
10414888	Gm10891 /// Gm13926	N/A	N/A
10414891	A130082M07Rik /// LOC547323	N/A	N/A
10414894	Gm26	N/A	N/A
10414900	---	N/A	N/A
10414903	Gm10893 /// C920008G01Rik	N/A	N/A
10414906	Gm13978	N/A	N/A
10414911	Trav13n-1 /// Trav13-3 /// Gm13978 /// Gm17006	N/A	N/A
10414917	---	N/A	N/A
10414923	Gm13955	N/A	N/A
10414926	---	N/A	N/A
10414929	Gm13949 /// Gm7174 /// Gm13959	N/A	N/A
10414932	---	N/A	N/A
10414934	LOC547323	N/A	N/A
10414955	A130082M07Rik /// LOC547323	N/A	N/A
10414960	Trav13d-4 /// Gm6033 /// Gm17006	N/A	N/A
10414963	Gm13926	N/A	N/A
10414967	Gm17006	N/A	N/A
10414978	Gm13894	N/A	N/A
10414981	Gm13893	N/A	N/A
10414984	Gm13892	N/A	N/A
10415011	---	N/A	N/A
10415013	---	N/A	N/A
10415017	---	N/A	N/A
10415019	---	N/A	N/A
10415021	Abhd4	N/A	N/A
10415030	Oxa1l	N/A	N/A
10415052	Mmp14	N/A	N/A
10415065	Lrp10	N/A	N/A
10415074	Rem2	N/A	N/A
10415081	---	N/A	N/A
10415090	Cdh24	N/A	N/A
10415092	4930579G18Rik	N/A	N/A
10415095	Acin1 /// 1700123O20Rik	N/A	N/A
10415119	Pabpn1	N/A	N/A
10415132	Cmtm5	N/A	N/A
10415228	Cpne6	N/A	N/A
10415262	Dcaf11	N/A	N/A
10415279	Fitm1	N/A	N/A
10415282	Psme1	N/A	N/A
10415293	Rnf31	N/A	N/A
10415319	Irf9	N/A	N/A
10415363	Gmpr2	N/A	N/A
10415377	2610027L16Rik	N/A	N/A
10415388	Ltb4r2	N/A	N/A
10415408	Nynrin	N/A	N/A
10415411	Nynrin	N/A	N/A
10415413	Nynrin	N/A	N/A
10415444	---	N/A	N/A
10415570	Pspc1 /// Gm10034	N/A	N/A
10415572	2410022M11Rik	N/A	N/A
10415574	Ccni	N/A	N/A
10415576	Zmym2	N/A	N/A
10415604	Gjb2 /// Gm10871	N/A	N/A
10415608	Ift88	N/A	N/A
10415640	Rpl12 /// Snora65	N/A	N/A
10415642	Sap18	N/A	N/A
10415645	Mrp63	N/A	N/A

10415649	Zdhhc20	N/A	N/A
10415660	Rpl13	N/A	N/A
10415678	Cab39l	N/A	N/A
10415693	Shisa2	N/A	N/A
10415700	Mtmr6	N/A	N/A
10415721	---	N/A	N/A
10415723	---	N/A	N/A
10415725	Spata13	N/A	N/A
10415778	Arl11	N/A	N/A
10415784	Trim13	N/A	N/A
10415787	Kcnrg	N/A	N/A
10415804	---	N/A	N/A
10415806	---	N/A	N/A
10415818	Wdfy2	N/A	N/A
10415835	Defb43	N/A	N/A
10415844	Ctsb /// Fdft1	N/A	N/A
10415866	Xkr6	N/A	N/A
10415873	Mir598	N/A	N/A
10415885	Sox7	N/A	N/A
10415888	Gm10867	N/A	N/A
10415911	Kif13b	N/A	N/A
10415955	---	N/A	N/A
10415957	---	N/A	N/A
10415960	Ints9	N/A	N/A
10415991	Zfp395	N/A	N/A
10416004	Zfp395	N/A	N/A
10416044	Ccdc25	N/A	N/A
10416057	Clu	N/A	N/A
10416069	Timm23	N/A	N/A
10416082	Trim35	N/A	N/A
10416090	Stmn4	N/A	N/A
10416107	Gm10032	N/A	N/A
10416110	Gm5464	N/A	N/A
10416112	Pnma2	N/A	N/A
10416122	---	N/A	N/A
10416124	---	N/A	N/A
10416153	Gm10861	N/A	N/A
10416155	Kctd9	N/A	N/A
10416169	Gnrh1	N/A	N/A
10416175	Nefl	N/A	N/A
10416181	Stc1	N/A	N/A
10416187	Rps2 /// Rps2-ps6	N/A	N/A
10416251	Egr3	N/A	N/A
10416256	Bin3	N/A	N/A
10416269	---	N/A	N/A
10416271	Mir320	N/A	N/A
10416273	Phyhip	N/A	N/A
10416279	Lgi3	N/A	N/A
10416290	Reep4	N/A	N/A
10416332	Fam160b2	N/A	N/A
10416340	Gfra2	N/A	N/A
10416353	---	N/A	N/A
10416355	Rcbtb2	N/A	N/A
10416371	Lpar6	N/A	N/A
10416379	Sucla2	N/A	N/A
10416406	Htr2a	N/A	N/A
10416411	Esd	N/A	N/A
10416419	Gm10847	N/A	N/A
10416421	Lrch1	N/A	N/A
10416437	Lcp1	N/A	N/A
10416464	Zc3h13	N/A	N/A
10416485	Siah3	N/A	N/A
10416496	Tpt1 /// Tpt1p	N/A	N/A
10416503	Snora31	N/A	N/A
10416510	Nufip1	N/A	N/A
10416522	Tsc22d1	N/A	N/A
10416531	---	N/A	N/A
10416533	Ccdc122	N/A	N/A
10416541	Enox1	N/A	N/A
10416588	1300010F03Rik	N/A	N/A
10416653	Kbtbd7	N/A	N/A
10416655	Gm5465	N/A	N/A
10416657	Elf1	N/A	N/A
10416666	Sugt1	N/A	N/A
10416680	Pcdh8 /// Gm9748	N/A	N/A
10416696	LOC280487	N/A	N/A
10416698	LOC280487	N/A	N/A
10416700	Pcdh17	N/A	N/A
10416725	---	N/A	N/A
10416730	---	N/A	N/A
10416732	Snora30	N/A	N/A
10416734	---	N/A	N/A
10416785	---	N/A	N/A
10416791	---	N/A	N/A

10416793	Uchl3	N/A	N/A
10416835	Rpl17 /// Gm10268 /// Rpl17-ps3	N/A	N/A
10416843	Cln5	N/A	N/A
10416848	---	N/A	N/A
10416887	Slain1	N/A	N/A
10416915	Rpl41	N/A	N/A
10416917	Trim52	N/A	N/A
10416919	Gm6219 /// Gm6280	N/A	N/A
10416923	---	N/A	N/A
10416925	---	N/A	N/A
10416927	---	N/A	N/A
10416929	Gm6219 /// Gm6280	N/A	N/A
10416931	Slitrk5	N/A	N/A
10416938	Gm4822	N/A	N/A
10416940	Tpm3	N/A	N/A
10416942	---	N/A	N/A
10416948	Mir17	N/A	N/A
10416950	Mir18	N/A	N/A
10416956	Mir19b-1	N/A	N/A
10416958	Mir92-1 /// Mir17hg	N/A	N/A
10416960	Gpc5	N/A	N/A
10416974	Gpc6	N/A	N/A
10416989	Gpr180	N/A	N/A
10417000	---	N/A	N/A
10417002	A830021K08Rik	N/A	N/A
10417004	Dzip1 /// Cldn10	N/A	N/A
10417013	Dnajc3	N/A	N/A
10417027	Cldn10	N/A	N/A
10417034	Dnajc3	N/A	N/A
10417048	Hs6st3	N/A	N/A
10417065	Rap2a	N/A	N/A
10417068	---	N/A	N/A
10417070	Ipo5	N/A	N/A
10417095	Farp1	N/A	N/A
10417124	B930095G15Rik	N/A	N/A
10417128	---	N/A	N/A
10417130	Ubac2	N/A	N/A
10417147	Tm9sf2	N/A	N/A
10417167	Clybl	N/A	N/A
10417181	Gm10837	N/A	N/A
10417183	Pcca	N/A	N/A
10417208	Tmtc4	N/A	N/A
10417235	Gm3373	N/A	N/A
10417245	Gm1973	N/A	N/A
10417253	Gm1973 /// Gm10338	N/A	N/A
10417258	Gm8348	N/A	N/A
10417264	Gm3373	N/A	N/A
10417273	Esd	N/A	N/A
10417275	---	N/A	N/A
10417281	Gm1973	N/A	N/A
10417302	Gm3373	N/A	N/A
10417315	Gm3373	N/A	N/A
10417319	D830030K20Rik /// Gm3033 /// Gm3030 /// Gm3278 /// LOC100504200 /// Gm8050 /// Gm1973	N/A	N/A
10417359	Gm3373	N/A	N/A
10417366	Gm10406 /// ENSMUSG00000068790 /// Gm8348 /// Gm2897 /// Gm10409	N/A	N/A
10417371	Gm3696 /// Gm2897	N/A	N/A
10417403	1700110I01Rik /// Gm8160	N/A	N/A
10417411	Gm3373	N/A	N/A
10417421	Gm3468	N/A	N/A
10417446	4930555G01Rik /// Gm2897 /// Gm3099	N/A	N/A
10417452	4930555G01Rik /// Gm2897 /// Gm3099	N/A	N/A
10417461	Gm3558	N/A	N/A
10417492	Gm5458	N/A	N/A
10417498	1700110I01Rik /// Gm8160	N/A	N/A
10417504	Gm1973 /// LOC100041151 /// Gm5797 /// Gm3149 /// Gm10338 /// Gm5458	N/A	N/A
10417526	Dnase1l3	N/A	N/A
10417538	Pdhb	N/A	N/A
10417544	Acox2	N/A	N/A
10417561	Fam107a	N/A	N/A
10417599	---	N/A	N/A
10417617	Gapdh /// Gm3839 /// Gm16374 /// Gm2606 /// Gm4609 /// Gm3200 /// Gm2451 /// Gm10293	N/A	N/A
10417628	Cadps	N/A	N/A
10417676	Thoc7	N/A	N/A
10417698	Olfr720	N/A	N/A
10417700	---	N/A	N/A
10417702	---	N/A	N/A
10417704	Lrrc3b	N/A	N/A
10417708	Oxsm	N/A	N/A
10417728	---	N/A	N/A
10417730	---	N/A	N/A
10417732	---	N/A	N/A
10417745	Rpl15 /// Gm4294	N/A	N/A
10417749	Ube2e1 /// Nkiras1	N/A	N/A
10417757	Vps4b	N/A	N/A

10417759	Ube2e2	N/A	N/A
10417769	Gm3696	N/A	N/A
10417773	Gm5458 /// Gm5797 /// Gm3047 /// Gm8159 /// Gm3453 /// Gm3476 /// Gm3149 /// Gm3642	N/A	N/A
10417778	2700060E02Rik /// Nid2	N/A	N/A
10417787	Gng2	N/A	N/A
10417794	1810063B07Rik	N/A	N/A
10417798	Kcnk5	N/A	N/A
10417813	Ecd	N/A	N/A
10417829	Dnajc9	N/A	N/A
10417836	Mrps16	N/A	N/A
10417869	Anxa7	N/A	N/A
10417887	Zmynd17	N/A	N/A
10417895	Ppp3cb	N/A	N/A
10417912	Usp54	N/A	N/A
10417920	Usp54	N/A	N/A
10417946	Sec24c /// 6230400D17Rik	N/A	N/A
10417950	Ndst2	N/A	N/A
10417972	Camk2g	N/A	N/A
10418002	---	N/A	N/A
10418004	Ap3m1	N/A	N/A
10418053	Kcnma1	N/A	N/A
10418096	Dlg5	N/A	N/A
10418129	---	N/A	N/A
10418131	Polr3a	N/A	N/A
10418167	Ppif /// Ppifos	N/A	N/A
10418169	1700054O19Rik	N/A	N/A
10418171	Zcchc24	N/A	N/A
10418177	Anxa11 /// Gm9872	N/A	N/A
10418185	D14Ertd449e	N/A	N/A
10418198	D14Ertd449e	N/A	N/A
10418210	D14Ertd449e	N/A	N/A
10418218	Slmap	N/A	N/A
10418244	Fam116a	N/A	N/A
10418247	Pde12	N/A	N/A
10418251	Appl1	N/A	N/A
10418275	Ccdc66 /// D14Abb1e	N/A	N/A
10418295	---	N/A	N/A
10418300	Cacna2d3	N/A	N/A
10418339	Gm10245	N/A	N/A
10418434	Itih3	N/A	N/A
10418455	Itih1	N/A	N/A
10418480	Gnl3	N/A	N/A
10418498	Rpl7a	N/A	N/A
10418500	2010107H07Rik	N/A	N/A
10418578	Nisch	N/A	N/A
10418604	Phf7	N/A	N/A
10418718	---	N/A	N/A
10418747	Hacl1	N/A	N/A
10418766	Ankrd28	N/A	N/A
10418796	Dph3	N/A	N/A
10418804	Timm23	N/A	N/A
10418835	Slc18a3	N/A	N/A
10418846	3425401B19Rik	N/A	N/A
10418879	Mapk8	N/A	N/A
10418895	Zfp488	N/A	N/A
10418901	Gprin2	N/A	N/A
10418903	Gprin2	N/A	N/A
10418905	Fam35a	N/A	N/A
10418917	2200001I15Rik	N/A	N/A
10418921	Sncg	N/A	N/A
10418927	Bmpr1a	N/A	N/A
10418982	4930596D02Rik	N/A	N/A
10418989	---	N/A	N/A
10418991	Gcap14	N/A	N/A
10419034	2610528A11Rik	N/A	N/A
10419038	Ghitm	N/A	N/A
10419049	Nrg3	N/A	N/A
10419060	---	N/A	N/A
10419073	Tspan14	N/A	N/A
10419089	Dydc2	N/A	N/A
10419108	Gm9890	N/A	N/A
10419116	1700001F09Rik /// Gm10375 /// Gm3543 /// Gm3676	N/A	N/A
10419119	Gm8165	N/A	N/A
10419122	Gm8165	N/A	N/A
10419125	1700091H14Rik /// Gm8005	N/A	N/A
10419136	Cdv3	N/A	N/A
10419154	Ear2	N/A	N/A
10419156	Ear2	N/A	N/A
10419160	---	N/A	N/A
10419167	Ptgdr	N/A	N/A
10419170	Txndc16	N/A	N/A
10419198	Ero1l	N/A	N/A
10419216	Gnpnat1 /// Styx	N/A	N/A
10419240	Ddhd1	N/A	N/A

10419261	Bmp4	N/A	N/A
10419267	Cnih	N/A	N/A
10419274	Gmfb	N/A	N/A
10419284	Gm10101	N/A	N/A
10419286	---	N/A	N/A
10419288	Gch1	N/A	N/A
10419296	Wdhd1	N/A	N/A
10419321	---	N/A	N/A
10419343	Atg14	N/A	N/A
10419354	Map1lc3b	N/A	N/A
10419366	Gm534	N/A	N/A
10419368	---	N/A	N/A
10419370	Exoc5	N/A	N/A
10419390	---	N/A	N/A
10419399	Slc35f4	N/A	N/A
10419414	---	N/A	N/A
10419416	3632451O06Rik	N/A	N/A
10419429	Olfr724 /// Olfr723	N/A	N/A
10419435	Olfr725	N/A	N/A
10419438	Olfr726	N/A	N/A
10419440	Olfr728	N/A	N/A
10419442	Olfr729	N/A	N/A
10419444	Olfr730	N/A	N/A
10419448	Olfr732	N/A	N/A
10419450	Olfr733	N/A	N/A
10419452	Olfr734	N/A	N/A
10419454	Olfr735	N/A	N/A
10419460	Ttc5	N/A	N/A
10419469	---	N/A	N/A
10419532	Osgep	N/A	N/A
10419542	Tmem55b /// Apex1	N/A	N/A
10419557	Rnase11	N/A	N/A
10419559	Rnase12	N/A	N/A
10419561	Olfr750	N/A	N/A
10419563	Rnase1	N/A	N/A
10419566	Ang2	N/A	N/A
10419578	Ndrg2	N/A	N/A
10419594	Rnase13	N/A	N/A
10419596	Zfp219	N/A	N/A
10419604	Olfr221	N/A	N/A
10419607	Hnrnpc	N/A	N/A
10419611	Supt16h /// Rpgrip1	N/A	N/A
10419638	Chd8	N/A	N/A
10419674	Snord8	N/A	N/A
10419676	Rab2b	N/A	N/A
10419691	Mettl3 /// Tox4	N/A	N/A
10419711	Psma3	N/A	N/A
10419713	Olfr1512	N/A	N/A
10419715	Olfr1510 /// Olfr1511	N/A	N/A
10419717	Olfr1510	N/A	N/A
10419729	Nrbf2	N/A	N/A
10419731	Nrbf2	N/A	N/A
10419736	Dad1	N/A	N/A
10419742	Olfr49	N/A	N/A
10419757	Gm10366	N/A	N/A
10419759	Prmt5	N/A	N/A
10419779	Haus4	N/A	N/A
10419803	4931414P19Rik	N/A	N/A
10419810	Psmb5	N/A	N/A
10419814	Cdh24	N/A	N/A
10419825	Acin1	N/A	N/A
10419850	Cebpe	N/A	N/A
10419854	Slc7a8	N/A	N/A
10419867	Homez	N/A	N/A
10419872	Ppp1r3e	N/A	N/A
10419874	Slc22a17	N/A	N/A
10419932	Mir208a	N/A	N/A
10419966	Zfhx2 /// Zfhx2as	N/A	N/A
10420011	Myl6 /// Gm8894	N/A	N/A
10420019	Gm10876	N/A	N/A
10420021	Dcaf11 /// A730061H03Rik	N/A	N/A
10420023	Fam158a	N/A	N/A
10420030	Psme2	N/A	N/A
10420080	Mdp1	N/A	N/A
10420089	Nedd8	N/A	N/A
10420114	Tgm1	N/A	N/A
10420131	Rabggta	N/A	N/A
10420155	Dhrs1	N/A	N/A
10420165	Cideb	N/A	N/A
10420216	Sdr39u1	N/A	N/A
10420237	Ube2j2 /// Gm5801	N/A	N/A
10420316	Atp12a /// Gm10873	N/A	N/A
10420338	Pspc1 /// Gm10034	N/A	N/A
10420348	Zmym5	N/A	N/A

10420359	Gja3	N/A	N/A
10420362	Gjb2 /// Gm10871	N/A	N/A
10420366	Gjb6	N/A	N/A
10420372	Cryl1	N/A	N/A
10420383	AU040096	N/A	N/A
10420385	N6amt2	N/A	N/A
10420390	Xpo4	N/A	N/A
10420413	Lats2	N/A	N/A
10420437	Zdhhc20	N/A	N/A
10420439	Zdhhc20	N/A	N/A
10420457	Efha1	N/A	N/A
10420470	---	N/A	N/A
10420503	Setdb2	N/A	N/A
10420516	Cdadc1	N/A	N/A
10420532	Atp8a2	N/A	N/A
10420572	Nupl1 /// Mir719	N/A	N/A
10420589	Fam123a /// Gm10235	N/A	N/A
10420591	---	N/A	N/A
10420594	C1qtnf9	N/A	N/A
10420614	Sacs	N/A	N/A
10420628	---	N/A	N/A
10420631	Ebpl	N/A	N/A
10420637	Kpna3	N/A	N/A
10420659	6330409N04Rik	N/A	N/A
10420666	Mir16-1	N/A	N/A
10420668	Mir15a	N/A	N/A
10420670	Dleu2	N/A	N/A
10420672	Dleu7	N/A	N/A
10420724	Defb30	N/A	N/A
10420730	Fdft1 /// Ctsb	N/A	N/A
10420747	Gata4	N/A	N/A
10420785	Mtmr9	N/A	N/A
10420787	Mtmr9	N/A	N/A
10420823	Hmbox1	N/A	N/A
10420837	Extl3	N/A	N/A
10420846	Fzd3	N/A	N/A
10420860	Elp3	N/A	N/A
10420889	1110020C17Rik	N/A	N/A
10420899	Gulo	N/A	N/A
10420935	Ephx2	N/A	N/A
10420957	Ptk2b	N/A	N/A
10420986	Rpl21-ps10	N/A	N/A
10420988	Dpysl2	N/A	N/A
10421026	Ebf2 /// Gm10860	N/A	N/A
10421046	Dock5	N/A	N/A
10421172	Slc25a37	N/A	N/A
10421184	Loxl2 /// Gm10325	N/A	N/A
10421197	Chmp7	N/A	N/A
10421212	---	N/A	N/A
10421214	Rhobtb2	N/A	N/A
10421227	2610301G19Rik	N/A	N/A
10421269	Sorbs3	N/A	N/A
10421309	Slc39a14	N/A	N/A
10421351	Polr3d	N/A	N/A
10421361	Bmp1	N/A	N/A
10421418	Epb4.9	N/A	N/A
10421486	F630201L12Rik	N/A	N/A
10421488	Fndc3a	N/A	N/A
10421515	---	N/A	N/A
10421524	---	N/A	N/A
10421526	Rb1	N/A	N/A
10421555	Mir687	N/A	N/A
10421557	Itm2b	N/A	N/A
10421571	Rps6 /// Rps6-ps2 /// Gm16409	N/A	N/A
10421573	Cbx3 /// Gm6901	N/A	N/A
10421577	Gm9779	N/A	N/A
10421581	Lrch1	N/A	N/A
10421622	---	N/A	N/A
10421624	Cog3	N/A	N/A
10421648	Slc25a30	N/A	N/A
10421661	Gtf2f2	N/A	N/A
10421670	---	N/A	N/A
10421672	1200011I18Rik	N/A	N/A
10421683	Rps2 /// Rps2-ps6	N/A	N/A
10421694	---	N/A	N/A
10421697	9030625A04Rik	N/A	N/A
10421723	Dnajc15	N/A	N/A
10421749	Akap11	N/A	N/A
10421758	Akap11	N/A	N/A
10421768	Akap11	N/A	N/A
10421774	Dgkh	N/A	N/A
10421817	Naa16	N/A	N/A
10421840	Wbp4	N/A	N/A
10421863	Pcdh8	N/A	N/A

10421870	Gm10845	N/A	N/A
10421873	---	N/A	N/A
10421875	---	N/A	N/A
10421906	---	N/A	N/A
10421911	Pcdh20	N/A	N/A
10421917	Pabpc4	N/A	N/A
10421920	---	N/A	N/A
10421922	---	N/A	N/A
10421924	Pcdh9	N/A	N/A
10421930	---	N/A	N/A
10421932	Pcdh9	N/A	N/A
10421934	Klhl1	N/A	N/A
10421970	---	N/A	N/A
10421972	Rpl36a	N/A	N/A
10421981	Dis3 /// Pibf1	N/A	N/A
10422005	---	N/A	N/A
10422007	---	N/A	N/A
10422009	---	N/A	N/A
10422011	---	N/A	N/A
10422013	Klf12	N/A	N/A
10422022	Gm10843	N/A	N/A
10422024	---	N/A	N/A
10422026	1700110M21Rik	N/A	N/A
10422028	Tbc1d4	N/A	N/A
10422052	Commd6	N/A	N/A
10422055	Gm9922	N/A	N/A
10422057	Rpl7a /// Gm16477 /// Rpl7a-ps10	N/A	N/A
10422075	Mycbp2	N/A	N/A
10422161	Gm10293	N/A	N/A
10422164	Ednrb	N/A	N/A
10422176	D130079A08Rik	N/A	N/A
10422185	Rnf219	N/A	N/A
10422194	Rbm26	N/A	N/A
10422225	---	N/A	N/A
10422227	Spry2	N/A	N/A
10422232	Gm6219 /// Gm6280	N/A	N/A
10422234	---	N/A	N/A
10422236	---	N/A	N/A
10422238	Rpl27a-ps2	N/A	N/A
10422240	Slitrk1	N/A	N/A
10422244	Slitrk6	N/A	N/A
10422247	LOC280487	N/A	N/A
10422272	Sox21	N/A	N/A
10422321	Dzip1	N/A	N/A
10422348	Uggt2	N/A	N/A
10422393	---	N/A	N/A
10422396	Stk24	N/A	N/A
10422436	Dock9	N/A	N/A
10422493	Gpr18	N/A	N/A
10422496	Gpr183	N/A	N/A
10422504	Zic5	N/A	N/A
10422509	---	N/A	N/A
10422512	A2ld1	N/A	N/A
10422518	Tmtc4	N/A	N/A
10422537	Nalcn	N/A	N/A
10422585	Fgf14	N/A	N/A
10422598	Sepp1	N/A	N/A
10422606	Fbxo4	N/A	N/A
10422608	Oxct1	N/A	N/A
10422635	C6	N/A	N/A
10422655	Gapdh	N/A	N/A
10422699	---	N/A	N/A
10422701	Rpl37	N/A	N/A
10422707	Prkaa1	N/A	N/A
10422718	Ttc33	N/A	N/A
10422725	---	N/A	N/A
10422728	Dab2	N/A	N/A
10422748	C9	N/A	N/A
10422776	Phb	N/A	N/A
10422781	Rictor	N/A	N/A
10422822	Lifr	N/A	N/A
10422854	Nup155	N/A	N/A
10422892	2410089E03Rik	N/A	N/A
10422912	2410089E03Rik	N/A	N/A
10422942	---	N/A	N/A
10422944	---	N/A	N/A
10422962	1110020G09Rik	N/A	N/A
10422980	Lmbrd2	N/A	N/A
10423002	Ugt3a2 /// Ugt3a1	N/A	N/A
10423005	Ugt3a1	N/A	N/A
10423011	Ugt3a2	N/A	N/A
10423030	Prlr	N/A	N/A
10423049	Prlr	N/A	N/A
10423053	Agxt2 /// Prlr	N/A	N/A

10423068	Rad1	N/A	N/A
10423078	Gm10389	N/A	N/A
10423090	Amacr	N/A	N/A
10423134	Zfr	N/A	N/A
10423172	Golph3	N/A	N/A
10423180	Gapdh /// Gm12070 /// Gm16374 /// Gm2606 /// Gm4609 /// Gm3200 /// Gm2451 /// Gm10293	N/A	N/A
10423185	Rnasen	N/A	N/A
10423228	Nsa2	N/A	N/A
10423243	Cdh10	N/A	N/A
10423258	Cdh12	N/A	N/A
10423271	---	N/A	N/A
10423274	Cdh18	N/A	N/A
10423287	Cdh18	N/A	N/A
10423289	---	N/A	N/A
10423291	Gm5468	N/A	N/A
10423293	Myo10	N/A	N/A
10423333	Fam134b	N/A	N/A
10423346	Zfp622	N/A	N/A
10423355	March11	N/A	N/A
10423358	March11	N/A	N/A
10423361	Rps2 /// Rps2-ps6 /// Gm5921 /// Gm6139	N/A	N/A
10423363	Ank	N/A	N/A
10423379	Tiaf2	N/A	N/A
10423383	---	N/A	N/A
10423386	Gm9891	N/A	N/A
10423471	Ctnnd2	N/A	N/A
10423498	Dap	N/A	N/A
10423503	Gm6624	N/A	N/A
10423505	Cmbl	N/A	N/A
10423518	Tas2r119	N/A	N/A
10423520	Sema5a	N/A	N/A
10423548	Sdc2	N/A	N/A
10423556	Pgcp	N/A	N/A
10423568	5730407I07Rik	N/A	N/A
10423570	---	N/A	N/A
10423575	---	N/A	N/A
10423577	Mtdh	N/A	N/A
10423593	Laptm4b	N/A	N/A
10423599	Matn2	N/A	N/A
10423663	Vps13b	N/A	N/A
10423731	---	N/A	N/A
10423733	---	N/A	N/A
10423740	---	N/A	N/A
10423742	Polr2k	N/A	N/A
10423768	Gm10385	N/A	N/A
10423791	4930447A16Rik	N/A	N/A
10423796	---	N/A	N/A
10423803	---	N/A	N/A
10423805	Atp6v1c1	N/A	N/A
10423821	Baalc	N/A	N/A
10423842	Dcaf13 /// Slc25a32	N/A	N/A
10423855	Rims2	N/A	N/A
10423917	---	N/A	N/A
10423919	4930555K19Rik	N/A	N/A
10423921	Oxr1	N/A	N/A
10423941	Ttc35	N/A	N/A
10423951	---	N/A	N/A
10423953	Trhr	N/A	N/A
10423963	Nudcd1 /// Eny2	N/A	N/A
10424060	A930017M01Rik	N/A	N/A
10424062	A930017M01Rik	N/A	N/A
10424070	Ewsr1	N/A	N/A
10424075	---	N/A	N/A
10424097	Med30	N/A	N/A
10424102	Gm7489	N/A	N/A
10424105	Colec10	N/A	N/A
10424113	Mal2	N/A	N/A
10424119	Nov	N/A	N/A
10424126	Depdc6	N/A	N/A
10424140	Col14a1	N/A	N/A
10424211	Gm10370	N/A	N/A
10424213	Zhx2	N/A	N/A
10424221	Wdr67	N/A	N/A
10424250	Snrpd2 /// Gm10120	N/A	N/A
10424252	Wdyhv1	N/A	N/A
10424260	D15Ertd621e	N/A	N/A
10424335	Rnf139	N/A	N/A
10424340	Ndufb9	N/A	N/A
10424345	Gm5045	N/A	N/A
10424347	---	N/A	N/A
10424349	Sqle	N/A	N/A
10424363	Nsmce2	N/A	N/A
10424370	Trib1	N/A	N/A
10424377	LOC280487	N/A	N/A

10424379	Srsf3	N/A	N/A
10424381	---	N/A	N/A
10424398	Gm10368	N/A	N/A
10424400	Myc	N/A	N/A
10424404	Pvt1	N/A	N/A
10424411	Tsg101	N/A	N/A
10424413	Hmgb1	N/A	N/A
10424416	---	N/A	N/A
10424437	---	N/A	N/A
10424439	Efr3a	N/A	N/A
10424467	Phf20l1	N/A	N/A
10424485	Phf20l1	N/A	N/A
10424553	Gm6813 /// Gm9104 /// Rpl21-ps4 /// Rpl21-ps7 /// Gm16415 /// Gm16416 /// Rpl21-ps12 /// Rpl21-ps14 /// Rpl21-ps10 ///	N/A	N/A
10424555	---	N/A	N/A
10424557	Gm7125	N/A	N/A
10424559	Khdrbs3	N/A	N/A
10424573	---	N/A	N/A
10424582	---	N/A	N/A
10424622	---	N/A	N/A
10424624	Bai1	N/A	N/A
10424660	4933427E11Rik /// Jrk	N/A	N/A
10424667	4930572J05Rik	N/A	N/A
10424674	---	N/A	N/A
10424676	Ly6e	N/A	N/A
10424683	Ly6g	N/A	N/A
10424686	BC025446	N/A	N/A
10424695	Gpihbp1	N/A	N/A
10424779	Cks2	N/A	N/A
10424781	Grina	N/A	N/A
10424810	Gpaa1	N/A	N/A
10424823	Rps6 /// Gm16409	N/A	N/A
10424825	Cyc1	N/A	N/A
10424833	Maf1	N/A	N/A
10424853	Brp16	N/A	N/A
10424894	Heatr7a	N/A	N/A
10424905	Scx	N/A	N/A
10424909	Hsf1	N/A	N/A
10424922	Gpr172b	N/A	N/A
10424929	Adck5	N/A	N/A
10424945	Kifc2	N/A	N/A
10424965	Ppp1r16a	N/A	N/A
10424979	Gpt	N/A	N/A
10425000	Lrrc14	N/A	N/A
10425012	Zfp7	N/A	N/A
10425016	Commd5	N/A	N/A
10425018	Rpl8	N/A	N/A
10425024	1110038F14Rik	N/A	N/A
10425046	---	N/A	N/A
10425049	Apol9a /// Apol9b	N/A	N/A
10425076	---	N/A	N/A
10425078	Mpst	N/A	N/A
10425082	Kctd17	N/A	N/A
10425109	Elfn2 /// 1700041B01Rik	N/A	N/A
10425114	Tmed2	N/A	N/A
10425116	Cdc42ep1	N/A	N/A
10425120	Gga1	N/A	N/A
10425158	Pdxp	N/A	N/A
10425161	Lgals1	N/A	N/A
10425166	Nol12	N/A	N/A
10425207	H1f0	N/A	N/A
10425211	Gcat	N/A	N/A
10425223	Galr3 /// Gcat	N/A	N/A
10425226	Eif3l	N/A	N/A
10425257	Polr2f	N/A	N/A
10425263	Gm10864	N/A	N/A
10425265	---	N/A	N/A
10425293	Cby1	N/A	N/A
10425299	Tomm22	N/A	N/A
10425302	Gtpbp1	N/A	N/A
10425317	---	N/A	N/A
10425319	Gm10856	N/A	N/A
10425321	Apobec3	N/A	N/A
10425333	Apobec3	N/A	N/A
10425335	Syngr1	N/A	N/A
10425341	Tab1	N/A	N/A
10425357	Smcr7l	N/A	N/A
10425430	Tnrc6b	N/A	N/A
10425461	Adsl	N/A	N/A
10425477	Sgsm3	N/A	N/A
10425501	---	N/A	N/A
10425507	Mchr1	N/A	N/A
10425511	Xpnpep3	N/A	N/A
10425522	Rbx1	N/A	N/A
10425527	Ep300	N/A	N/A

10425559	L3mbtl2	N/A	N/A
10425578	Zc3h7b	N/A	N/A
10425611	Aco2	N/A	N/A
10425623	Csdc2	N/A	N/A
10425630	1700029P11Rik	N/A	N/A
10425651	4930407I10Rik	N/A	N/A
10425653	4930407I10Rik	N/A	N/A
10425695	Srebf2	N/A	N/A
10425723	1500009C09Rik	N/A	N/A
10425755	---	N/A	N/A
10425757	1500032L24Rik	N/A	N/A
10425761	---	N/A	N/A
10425799	Rnu12	N/A	N/A
10425801	Bik	N/A	N/A
10425808	Tspo	N/A	N/A
10425814	Mpped1	N/A	N/A
10425822	Pnpla3	N/A	N/A
10425834	Samm50	N/A	N/A
10425852	Parvb	N/A	N/A
10425880	Prr5	N/A	N/A
10425903	Gm4609 /// Gm2451	N/A	N/A
10425905	Nup50	N/A	N/A
10425966	Atxn10	N/A	N/A
10425981	Gm4825	N/A	N/A
10425983	Mirlet7c-2	N/A	N/A
10425985	Mirlet7b	N/A	N/A
10425987	Ppara	N/A	N/A
10425999	Ttc38	N/A	N/A
10426065	Tbc1d22a	N/A	N/A
10426081	Fam19a5	N/A	N/A
10426093	Zbed4 /// 4930529C04Rik	N/A	N/A
10426098	Creld2	N/A	N/A
10426150	Panx2	N/A	N/A
10426157	Trabd	N/A	N/A
10426169	1300018J18Rik /// Tubgcp6	N/A	N/A
10426180	Ppp6r2	N/A	N/A
10426225	Ncaph2	N/A	N/A
10426240	Klhdc7b	N/A	N/A
10426242	Chkb	N/A	N/A
10426244	Mapk8ip2	N/A	N/A
10426261	Shank3	N/A	N/A
10426284	Acr	N/A	N/A
10426292	Alg10b	N/A	N/A
10426296	Gm10839	N/A	N/A
10426298	---	N/A	N/A
10426301	Tcea1	N/A	N/A
10426368	Lrrk2	N/A	N/A
10426383	Muc19	N/A	N/A
10426397	Cntn1	N/A	N/A
10426425	Pdzrn4	N/A	N/A
10426435	Gapdh /// Gm12070 /// Gm2606 /// Gm4609 /// Gm10290 /// Gm10291	N/A	N/A
10426437	Rn7sk	N/A	N/A
10426439	Pphln1	N/A	N/A
10426451	Irak4	N/A	N/A
10426467	Tmem117	N/A	N/A
10426477	Snrpc	N/A	N/A
10426479	Ano6	N/A	N/A
10426500	---	N/A	N/A
10426507	Arid2	N/A	N/A
10426535	Fam113b /// Amigo2	N/A	N/A
10426542	Rapgef3 /// Gm10835	N/A	N/A
10426544	Slc48a1	N/A	N/A
10426550	Tmem106c	N/A	N/A
10426557	Pfkm	N/A	N/A
10426581	AI836003	N/A	N/A
10426592	Olfr282	N/A	N/A
10426598	---	N/A	N/A
10426601	Olfr234	N/A	N/A
10426603	Ccnt1 /// 9330020H09Rik	N/A	N/A
10426627	Ccdc65	N/A	N/A
10426643	Prkag1	N/A	N/A
10426645	B430209F14Rik	N/A	N/A
10426648	Rpl36 /// Gm13611 /// Gm8973	N/A	N/A
10426650	Tuba1c /// Gm6682 /// Tuba1b /// Gm5620	N/A	N/A
10426685	Dnajc22	N/A	N/A
10426689	Spats2	N/A	N/A
10426751	Tmbim6	N/A	N/A
10426767	Aqp5	N/A	N/A
10426798	Smarcd1	N/A	N/A
10426812	Gpd1	N/A	N/A
10426822	2310016M24Rik	N/A	N/A
10426827	Larp4	N/A	N/A
10426835	Dip2b	N/A	N/A
10426875	Atf1	N/A	N/A

10426889	Sec61b	N/A	N/A
10426891	Mettl7a1	N/A	N/A
10426909	Letmd1	N/A	N/A
10426921	Dazap2	N/A	N/A
10426955	Scn8a	N/A	N/A
10427015	Acvr1b	N/A	N/A
10427035	Nr4a1	N/A	N/A
10427045	9430023L20Rik	N/A	N/A
10427049	6030408B16Rik	N/A	N/A
10427075	Krt18	N/A	N/A
10427083	Eif4b	N/A	N/A
10427095	Tenc1	N/A	N/A
10427125	Igfbp6	N/A	N/A
10427148	Zfp740	N/A	N/A
10427159	Gm9918	N/A	N/A
10427162	Mfsd5	N/A	N/A
10427199	Pfdn5	N/A	N/A
10427205	Myg1	N/A	N/A
10427214	Sp1	N/A	N/A
10427235	Prr13	N/A	N/A
10427241	Pcbp2 /// Map3k12	N/A	N/A
10427253	Map3k12	N/A	N/A
10427255	Tarbp2	N/A	N/A
10427266	Rpl39	N/A	N/A
10427284	Mir196a-2	N/A	N/A
10427301	Mir615	N/A	N/A
10427310	Hnrnpa1 /// Gm10052 /// Gm5643	N/A	N/A
10427323	Copz1	N/A	N/A
10427334	Mir148b	N/A	N/A
10427369	Pde1b	N/A	N/A
10427389	LOC280487	N/A	N/A
10427399	Ccdc152	N/A	N/A
10427420	Fbxo4	N/A	N/A
10427428	AW549877	N/A	N/A
10427454	Card6	N/A	N/A
10427459	---	N/A	N/A
10427468	---	N/A	N/A
10427492	Gm10050	N/A	N/A
10427494	---	N/A	N/A
10427521	Wdr70	N/A	N/A
10427538	Nipbl	N/A	N/A
10427590	Slc1a3	N/A	N/A
10427603	---	N/A	N/A
10427622	---	N/A	N/A
10427624	Ugt3a2 /// Ugt3a1	N/A	N/A
10427653	Spef2	N/A	N/A
10427655	Spef2	N/A	N/A
10427657	Spef2	N/A	N/A
10427659	Spef2	N/A	N/A
10427661	Spef2	N/A	N/A
10427663	Spef2	N/A	N/A
10427665	Spef2	N/A	N/A
10427667	Spef2	N/A	N/A
10427669	Spef2	N/A	N/A
10427673	---	N/A	N/A
10427675	Spef2	N/A	N/A
10427677	Spef2	N/A	N/A
10427679	Spef2	N/A	N/A
10427681	Spef2	N/A	N/A
10427683	Spef2	N/A	N/A
10427685	Spef2	N/A	N/A
10427687	Spef2	N/A	N/A
10427689	Spef2	N/A	N/A
10427693	Spef2	N/A	N/A
10427704	Dnajc21	N/A	N/A
10427718	Brix1	N/A	N/A
10427742	---	N/A	N/A
10427744	Rai14	N/A	N/A
10427766	Rxfp3	N/A	N/A
10427772	Tars	N/A	N/A
10427796	Npr3	N/A	N/A
10427807	Sub1	N/A	N/A
10427845	F830212C03Rik	N/A	N/A
10427847	LOC669751	N/A	N/A
10427849	6030458C11Rik	N/A	N/A
10427862	Cdh6	N/A	N/A
10427877	---	N/A	N/A
10427879	---	N/A	N/A
10427881	---	N/A	N/A
10427883	Acot10	N/A	N/A
10427885	Gm10052 /// Hnrnpa1 /// Gm5803	N/A	N/A
10427888	---	N/A	N/A
10427895	Basp1	N/A	N/A
10427898	Fbxl7	N/A	N/A

10427904	Fbxl7	N/A	N/A
10427906	D130046C19Rik	N/A	N/A
10427908	Gm9948	N/A	N/A
10427918	Fam105a	N/A	N/A
10427928	Trio	N/A	N/A
10427991	Trio	N/A	N/A
10427993	---	N/A	N/A
10427995	---	N/A	N/A
10427997	Ankrd33b	N/A	N/A
10428002	Ankrd33b	N/A	N/A
10428004	Ankrd33b	N/A	N/A
10428012	Ropn1l	N/A	N/A
10428018	Ube2v2 /// LOC635992	N/A	N/A
10428020	March6	N/A	N/A
10428066	1700084J12Rik	N/A	N/A
10428068	Tspyl5	N/A	N/A
10428074	Rpl30	N/A	N/A
10428081	Hrsp12	N/A	N/A
10428089	Nipal2	N/A	N/A
10428103	Stk3	N/A	N/A
10428117	Mir599	N/A	N/A
10428157	Rnf19a	N/A	N/A
10428169	Rpl7a	N/A	N/A
10428171	Ankrd46	N/A	N/A
10428192	Pabpc1	N/A	N/A
10428204	Ywhaz	N/A	N/A
10428211	Zfp706	N/A	N/A
10428215	---	N/A	N/A
10428222	Ncald	N/A	N/A
10428232	Rrm2b	N/A	N/A
10428302	Klf10	N/A	N/A
10428308	G930009F23Rik	N/A	N/A
10428326	---	N/A	N/A
10428328	Slc25a32	N/A	N/A
10428336	Ftl2 /// Ftl1	N/A	N/A
10428338	Dpys	N/A	N/A
10428366	Gm10383	N/A	N/A
10428368	---	N/A	N/A
10428374	---	N/A	N/A
10428376	Angpt1	N/A	N/A
10428396	---	N/A	N/A
10428398	Eif3e	N/A	N/A
10428405	Gm10373	N/A	N/A
10428407	Tmem74	N/A	N/A
10428412	Nudcd1	N/A	N/A
10428426	Sybu	N/A	N/A
10428439	---	N/A	N/A
10428443	Kcnv1	N/A	N/A
10428449	---	N/A	N/A
10428451	---	N/A	N/A
10428453	Csmd3	N/A	N/A
10428509	Csmd3	N/A	N/A
10428511	Csmd3	N/A	N/A
10428513	Csmd3	N/A	N/A
10428515	Csmd3	N/A	N/A
10428517	Csmd3	N/A	N/A
10428522	Csmd3	N/A	N/A
10428534	Trps1	N/A	N/A
10428536	Trps1	N/A	N/A
10428554	Eif3h	N/A	N/A
10428561	Rad21	N/A	N/A
10428579	Ext1	N/A	N/A
10428594	Samd12	N/A	N/A
10428602	---	N/A	N/A
10428613	Gm5215	N/A	N/A
10428648	Taf2	N/A	N/A
10428682	---	N/A	N/A
10428690	Mrpl13	N/A	N/A
10428698	Sntb1	N/A	N/A
10428736	Derl1	N/A	N/A
10428744	9130401M01Rik	N/A	N/A
10428753	---	N/A	N/A
10428755	Zhx1	N/A	N/A
10428796	Fbxo32	N/A	N/A
10428809	Klhl38	N/A	N/A
10428825	---	N/A	N/A
10428827	Tmem65	N/A	N/A
10428837	---	N/A	N/A
10428839	Gm5959	N/A	N/A
10428842	Tatdn1 /// Rnf139	N/A	N/A
10428857	Mtss1	N/A	N/A
10428877	E430025E21Rik	N/A	N/A
10428908	---	N/A	N/A
10428910	4933412E24Rik	N/A	N/A

10428918	9930014A18Rik /// Fam84b	N/A	N/A
10428931	---	N/A	N/A
10428933	H2afy3	N/A	N/A
10428983	Fam49b	N/A	N/A
10429029	Adcy8	N/A	N/A
10429081	Snrpc	N/A	N/A
10429083	Kcnq3	N/A	N/A
10429100	Lrrc6	N/A	N/A
10429140	Ndrg1	N/A	N/A
10429160	St3gal1	N/A	N/A
10429176	St3gal1	N/A	N/A
10429178	Zfat	N/A	N/A
10429197	Mir30b	N/A	N/A
10429199	Mir30d	N/A	N/A
10429201	Nfu1	N/A	N/A
10429203	Fam135b	N/A	N/A
10429216	Fam135b	N/A	N/A
10429218	Fam135b	N/A	N/A
10429220	Fam135b	N/A	N/A
10429222	Fam135b	N/A	N/A
10429295	Kcnk9	N/A	N/A
10429298	Trappc9	N/A	N/A
10429325	---	N/A	N/A
10429327	Eif2c2	N/A	N/A
10429329	Eif2c2	N/A	N/A
10429385	Mir151	N/A	N/A
10429387	Ptplb	N/A	N/A
10429389	Rpl17 /// Gm10268 /// Rpl17-ps3	N/A	N/A
10429391	Slc45a4	N/A	N/A
10429400	---	N/A	N/A
10429407	---	N/A	N/A
10429424	---	N/A	N/A
10429426	Gm628	N/A	N/A
10429428	Gm628	N/A	N/A
10429440	Gm628	N/A	N/A
10429442	Gm628	N/A	N/A
10429444	Gm628	N/A	N/A
10429446	Gm628	N/A	N/A
10429460	7120482A17Rik	N/A	N/A
10429491	Arc	N/A	N/A
10429495	Jrk	N/A	N/A
10429506	Lypd2	N/A	N/A
10429515	Lynx1	N/A	N/A
10429520	Ly6d	N/A	N/A
10429564	Ly6a	N/A	N/A
10429568	Ly6c2 /// Ly6c1	N/A	N/A
10429588	9030619P08Rik	N/A	N/A
10429607	Top1mt	N/A	N/A
10429638	---	N/A	N/A
10429641	Naprt1	N/A	N/A
10429666	Pycrl	N/A	N/A
10429674	Tsta3	N/A	N/A
10429739	Puf60	N/A	N/A
10429754	Nrbp2	N/A	N/A
10429772	Eppk1	N/A	N/A
10429856	Spatc1 /// Gm10872	N/A	N/A
10429859	Oplah /// Gm10345	N/A	N/A
10429885	Sharpin	N/A	N/A
10429926	Dgat1	N/A	N/A
10429944	Scrt1	N/A	N/A
10429949	Rpl29	N/A	N/A
10429957	Fbxl6	N/A	N/A
10429968	---	N/A	N/A
10429970	Adck5 /// Gm10869	N/A	N/A
10430020	Vps28	N/A	N/A
10430058	Cyhr1	N/A	N/A
10430105	C030006K11Rik /// Lrrc24	N/A	N/A
10430113	Arhgap39	N/A	N/A
10430127	Zfp251	N/A	N/A
10430132	Zfp647	N/A	N/A
10430145	Rbfox2	N/A	N/A
10430166	Apol7a	N/A	N/A
10430174	Apol9a /// Apol9b	N/A	N/A
10430195	Apol8	N/A	N/A
10430201	Myh9	N/A	N/A
10430245	---	N/A	N/A
10430247	Txn2	N/A	N/A
10430255	Foxred2	N/A	N/A
10430265	Eif3d	N/A	N/A
10430280	Cacng2	N/A	N/A
10430282	Cacng2	N/A	N/A
10430289	Ift27	N/A	N/A
10430319	Tst	N/A	N/A
10430324	Tmprss6	N/A	N/A

10430370	---	N/A	N/A
10430372	Rac2	N/A	N/A
10430382	---	N/A	N/A
10430384	Elfn2	N/A	N/A
10430431	Gm10866	N/A	N/A
10430433	---	N/A	N/A
10430436	Gm10865	N/A	N/A
10430438	Ankrd54	N/A	N/A
10430447	1700088E04Rik /// Micall1	N/A	N/A
10430458	Sox10	N/A	N/A
10430489	Pla2g6	N/A	N/A
10430510	Tmem184b	N/A	N/A
10430533	Kcnj4	N/A	N/A
10430536	Ddx17	N/A	N/A
10430572	---	N/A	N/A
10430574	4933432B09Rik	N/A	N/A
10430593	Josd1	N/A	N/A
10430596	Sun2	N/A	N/A
10430618	Dnalc4	N/A	N/A
10430626	Nptxr /// Npcd /// Cbx6	N/A	N/A
10430645	D730005E14Rik	N/A	N/A
10430647	D730005E14Rik	N/A	N/A
10430649	Cbx7	N/A	N/A
10430660	Pdgfb	N/A	N/A
10430669	Rpl3	N/A	N/A
10430679	---	N/A	N/A
10430693	Mkl1	N/A	N/A
10430711	Slc25a17	N/A	N/A
10430723	---	N/A	N/A
10430725	St13	N/A	N/A
10430739	Dnajb7	N/A	N/A
10430743	---	N/A	N/A
10430745	Chadl	N/A	N/A
10430748	Rangap1	N/A	N/A
10430768	---	N/A	N/A
10430778	Phf5a	N/A	N/A
10430783	Polr3h	N/A	N/A
10430804	Pppde2	N/A	N/A
10430811	Nhp2l1	N/A	N/A
10430818	Tnfrsf13c	N/A	N/A
10430834	Naga	N/A	N/A
10430851	Cyp2d22	N/A	N/A
10430866	Cyp2d10	N/A	N/A
10430871	Gm9855 /// Tdg /// Gm5806	N/A	N/A
10430883	Cyp2d13	N/A	N/A
10430892	Pcdhb11 /// Cyp2d37-ps	N/A	N/A
10430909	Cyp2d26	N/A	N/A
10430920	Tcf20	N/A	N/A
10430929	Tbrg3	N/A	N/A
10430941	Rrp7a /// Serhl	N/A	N/A
10430945	Poldip3	N/A	N/A
10430956	Cyb5r3	N/A	N/A
10430974	Arfgap3	N/A	N/A
10430993	1700001L05Rik	N/A	N/A
10430997	Pacsin2	N/A	N/A
10431014	Vkorc1l1	N/A	N/A
10431017	Ttll1	N/A	N/A
10431030	Mcat	N/A	N/A
10431035	Ttll12	N/A	N/A
10431147	Ldoc1l	N/A	N/A
10431170	5031439G07Rik	N/A	N/A
10431208	---	N/A	N/A
10431266	Cerk	N/A	N/A
10431282	2810001A02Rik	N/A	N/A
10431284	Brd1	N/A	N/A
10431326	Mlc1	N/A	N/A
10431352	Tubgcp6	N/A	N/A
10431393	Mapk12	N/A	N/A
10431410	Mapk11	N/A	N/A
10431424	Plxnb2	N/A	N/A
10431463	Fam116b	N/A	N/A
10431486	Sbf1	N/A	N/A
10431528	Lmf2	N/A	N/A
10431558	Odf3b	N/A	N/A
10431585	Chkb /// BC090627	N/A	N/A
10431601	Arsa	N/A	N/A
10431612	Rabl2	N/A	N/A
10431625	Syt10	N/A	N/A
10431635	---	N/A	N/A
10431656	Rpl31 /// Gm16382	N/A	N/A
10431659	Kif21a	N/A	N/A
10431697	Abcd2	N/A	N/A
10431711	Slc2a13	N/A	N/A
10431722	Gxylt1	N/A	N/A

10431732	Zcrb1	N/A	N/A
10431802	Twf1	N/A	N/A
10431812	Nell2	N/A	N/A
10431839	Dbx2	N/A	N/A
10431845	Ano6	N/A	N/A
10431856	Srsf2ip	N/A	N/A
10431872	Slc38a1	N/A	N/A
10431874	Slc38a1	N/A	N/A
10431912	---	N/A	N/A
10431915	Slc38a4	N/A	N/A
10431935	Amigo2 /// Fam113b	N/A	N/A
10431943	---	N/A	N/A
10431946	5830453K13Rik	N/A	N/A
10431948	Rpap3	N/A	N/A
10432006	Hdac7	N/A	N/A
10432101	Senp1	N/A	N/A
10432122	Asb8	N/A	N/A
10432133	Olfr286	N/A	N/A
10432150	Olfr257 /// Olfr285	N/A	N/A
10432152	Olfr284	N/A	N/A
10432162	2310037I24Rik	N/A	N/A
10432176	Snora34	N/A	N/A
10432178	Snora2b	N/A	N/A
10432180	Ccnt1	N/A	N/A
10432215	Ddx23	N/A	N/A
10432236	Rnd1	N/A	N/A
10432256	Arf3	N/A	N/A
10432263	---	N/A	N/A
10432278	Ddn	N/A	N/A
10432294	Mll2	N/A	N/A
10432298	Mll2	N/A	N/A
10432398	Tuba1b /// Gm6682 /// Gm5620	N/A	N/A
10432404	Tuba1a	N/A	N/A
10432408	C1ql4	N/A	N/A
10432429	---	N/A	N/A
10432471	---	N/A	N/A
10432492	Faim2	N/A	N/A
10432509	Uxt	N/A	N/A
10432527	Lass5	N/A	N/A
10432540	Lima1	N/A	N/A
10432556	---	N/A	N/A
10432558	LOC100046692	N/A	N/A
10432571	2310068J16Rik	N/A	N/A
10432573	Slc11a2	N/A	N/A
10432593	Csrnp2	N/A	N/A
10432601	Tcfcp2	N/A	N/A
10432619	Pou6f1	N/A	N/A
10432636	Smagp	N/A	N/A
10432675	I730030J21Rik	N/A	N/A
10432918	Krt8	N/A	N/A
10432939	Csad	N/A	N/A
10432986	Aaas	N/A	N/A
10433003	Sp7	N/A	N/A
10433008	Map3k12	N/A	N/A
10433027	Npff	N/A	N/A
10433032	Atf7	N/A	N/A
10433034	Atf7	N/A	N/A
10433047	---	N/A	N/A
10433049	Atp5g2	N/A	N/A
10433055	Mir688	N/A	N/A
10433057	Calcoco1	N/A	N/A
10433073	Gm10831	N/A	N/A
10433088	Cbx5	N/A	N/A
10433163	Ppp1r1a	N/A	N/A
10433177	---	N/A	N/A
10433193	Olfr161	N/A	N/A
10433195	---	N/A	N/A
10433197	---	N/A	N/A
10433212	Olfr15	N/A	N/A
10433214	Zfp174	N/A	N/A
10433219	Nat15	N/A	N/A
10433228	Cluap1	N/A	N/A
10433262	Rpl7a /// Gm5766	N/A	N/A
10433264	Glis2	N/A	N/A
10433274	Vasn	N/A	N/A
10433278	Dnaja3	N/A	N/A
10433292	Hmox2 /// 5730403B10Rik	N/A	N/A
10433300	---	N/A	N/A
10433311	Mgrn1	N/A	N/A
10433331	Nudt16l1	N/A	N/A
10433340	4930451G09Rik	N/A	N/A
10433352	Ubn1	N/A	N/A
10433389	Fam86 /// Alg1	N/A	N/A
10433403	Rbfox1	N/A	N/A

10433428	---	N/A	N/A
10433431	---	N/A	N/A
10433433	BC024814	N/A	N/A
10433445	Abat	N/A	N/A
10433462	Pmm2	N/A	N/A
10433472	1810013L24Rik	N/A	N/A
10433478	---	N/A	N/A
10433492	Atf7ip2	N/A	N/A
10433494	Nubp1	N/A	N/A
10433536	Clec16a	N/A	N/A
10433562	Rpl30 /// Gm12191 /// Gm6570 /// Gm5481 /// Gm6109	N/A	N/A
10433573	---	N/A	N/A
10433575	Gm10832	N/A	N/A
10433578	Snn	N/A	N/A
10433589	Rundc2a	N/A	N/A
10433597	Snx29	N/A	N/A
10433618	Shisa9	N/A	N/A
10433620	Ercc4	N/A	N/A
10433639	Mkl2	N/A	N/A
10433656	Mkl2	N/A	N/A
10433658	Mir365-1	N/A	N/A
10433660	Bfar /// 3110001I22Rik	N/A	N/A
10433672	Rrn3	N/A	N/A
10433691	Ntan1 /// Pdxdc1	N/A	N/A
10433702	Mpv17l	N/A	N/A
10433709	2900011O08Rik	N/A	N/A
10433719	Mir484	N/A	N/A
10433721	Nde1	N/A	N/A
10433735	Abcc1	N/A	N/A
10433776	Snai2	N/A	N/A
10433782	Efcab1	N/A	N/A
10433792	---	N/A	N/A
10433797	Prkdc	N/A	N/A
10433885	Cebpd	N/A	N/A
10433887	Pkp2	N/A	N/A
10433902	Rpl30 /// Gm12191 /// Gm5481	N/A	N/A
10433904	Yars2	N/A	N/A
10433910	Top3b	N/A	N/A
10433929	Ppm1f	N/A	N/A
10433937	---	N/A	N/A
10433940	Mapk1	N/A	N/A
10433971	Rimbp3	N/A	N/A
10433988	Serpind1	N/A	N/A
10433996	Snap29	N/A	N/A
10434003	Crkl	N/A	N/A
10434007	Aifm3	N/A	N/A
10434029	Lztr1	N/A	N/A
10434089	Ccdc74a	N/A	N/A
10434094	Klhl22	N/A	N/A
10434105	Scarf2	N/A	N/A
10434117	B830017H08Rik	N/A	N/A
10434121	Tssk1	N/A	N/A
10434124	Tssk2	N/A	N/A
10434128	Vpreb2	N/A	N/A
10434131	AA066038	N/A	N/A
10434133	Dgcr6	N/A	N/A
10434165	Arvcf	N/A	N/A
10434189	---	N/A	N/A
10434191	Txnrd2	N/A	N/A
10434224	Gnb1l	N/A	N/A
10434226	---	N/A	N/A
10434229	Cldn5	N/A	N/A
10434233	Ufd1l	N/A	N/A
10434248	2510002D24Rik	N/A	N/A
10434252	Hira	N/A	N/A
10434281	LOC635992	N/A	N/A
10434283	---	N/A	N/A
10434285	---	N/A	N/A
10434287	Olfr166	N/A	N/A
10434289	---	N/A	N/A
10434302	Klhl24	N/A	N/A
10434313	Yeats2	N/A	N/A
10434348	Eif2b5	N/A	N/A
10434366	Dvl3	N/A	N/A
10434384	Ap2m1	N/A	N/A
10434396	Abcf3	N/A	N/A
10434418	Vwa5b2	N/A	N/A
10434446	Ece2	N/A	N/A
10434481	Eif4g1	N/A	N/A
10434516	Fam131a	N/A	N/A
10434523	Polr2h	N/A	N/A
10434552	---	N/A	N/A
10434556	---	N/A	N/A
10434577	Vps8	N/A	N/A

10434629	Map3k13	N/A	N/A
10434643	Psmb3	N/A	N/A
10434645	Senp2	N/A	N/A
10434664	Ndufa11	N/A	N/A
10434666	---	N/A	N/A
10434668	Tmem97	N/A	N/A
10434670	---	N/A	N/A
10434672	Gng5 /// Gm3150	N/A	N/A
10434675	Dnajb11	N/A	N/A
10434689	Ahsg	N/A	N/A
10434698	Fetub	N/A	N/A
10434709	Hrg	N/A	N/A
10434717	---	N/A	N/A
10434719	Kng1	N/A	N/A
10434733	Eif4a2	N/A	N/A
10434741	---	N/A	N/A
10434743	---	N/A	N/A
10434745	---	N/A	N/A
10434754	BC106179	N/A	N/A
10434758	St6gal1	N/A	N/A
10434778	Rtp4	N/A	N/A
10434782	Lpp	N/A	N/A
10434799	---	N/A	N/A
10434802	Lpp	N/A	N/A
10434804	Mir28	N/A	N/A
10434806	Lpp	N/A	N/A
10434835	Leprel1	N/A	N/A
10434845	Il1rap	N/A	N/A
10434869	Ccdc50	N/A	N/A
10434878	Gm10823	N/A	N/A
10434880	Hrasls	N/A	N/A
10434888	Opa1	N/A	N/A
10434925	Hes1	N/A	N/A
10434930	1700025H01Rik	N/A	N/A
10434932	Fam43a	N/A	N/A
10434934	Bdh1	N/A	N/A
10434942	Dlg1	N/A	N/A
10434993	Pigz	N/A	N/A
10434998	Ncbp2	N/A	N/A
10435004	1500031L02Rik	N/A	N/A
10435015	Wdr53	N/A	N/A
10435019	2310010M20Rik	N/A	N/A
10435024	Rnf168	N/A	N/A
10435031	Ubxn7	N/A	N/A
10435057	Pcyt1a	N/A	N/A
10435094	Tnk2	N/A	N/A
10435149	Fyttd1	N/A	N/A
10435162	Lrch3	N/A	N/A
10435185	---	N/A	N/A
10435187	Rpl35a /// Ino80 /// Gm10247	N/A	N/A
10435212	Osbpl11	N/A	N/A
10435226	Snx4	N/A	N/A
10435237	Zfp148	N/A	N/A
10435266	Heg1	N/A	N/A
10435271	Heg1	N/A	N/A
10435305	Itgb5	N/A	N/A
10435345	Mylk	N/A	N/A
10435383	Ptplb	N/A	N/A
10435388	Adcy5	N/A	N/A
10435413	---	N/A	N/A
10435453	Gm10237	N/A	N/A
10435455	Rpl35a /// Ino80 /// Gm10247	N/A	N/A
10435457	Parp9 /// Dtx3l	N/A	N/A
10435470	Kpna1	N/A	N/A
10435487	Wdr5b	N/A	N/A
10435489	Ccdc58	N/A	N/A
10435543	Golgb1	N/A	N/A
10435565	Hcls1	N/A	N/A
10435626	Hgd	N/A	N/A
10435641	Fstl1	N/A	N/A
10435661	Gpr156	N/A	N/A
10435674	---	N/A	N/A
10435676	Gsk3b	N/A	N/A
10435693	Cox17	N/A	N/A
10435712	Cd80	N/A	N/A
10435714	Tmem39a	N/A	N/A
10435724	B4galt4	N/A	N/A
10435743	---	N/A	N/A
10435745	Gm16498	N/A	N/A
10435748	D930030D11Rik	N/A	N/A
10435765	---	N/A	N/A
10435767	---	N/A	N/A
10435784	Ndufs5 /// BC002163	N/A	N/A
10435787	---	N/A	N/A

10435789	Zbtb20	N/A	N/A
10435791	Mir568 /// Zbtb20	N/A	N/A
10435793	Drd3	N/A	N/A
10435821	Naa50	N/A	N/A
10435832	Gm608	N/A	N/A
10435841	Ccdc52	N/A	N/A
10435897	BC027231	N/A	N/A
10435961	Gm10808	N/A	N/A
10435963	Atg3	N/A	N/A
10435991	---	N/A	N/A
10436034	---	N/A	N/A
10436037	---	N/A	N/A
10436041	---	N/A	N/A
10436046	---	N/A	N/A
10436048	Prdx1	N/A	N/A
10436169	Ift57	N/A	N/A
10436182	Cd47	N/A	N/A
10436196	Cd47	N/A	N/A
10436198	---	N/A	N/A
10436200	---	N/A	N/A
10436203	Gm4416	N/A	N/A
10436209	Cblb	N/A	N/A
10436232	Rpl24	N/A	N/A
10436239	2310061J03Rik /// Zbtb11	N/A	N/A
10436348	Tomm70a	N/A	N/A
10436369	Filip1l	N/A	N/A
10436372	Dcbld2 /// St3gal6	N/A	N/A
10436392	Cpox	N/A	N/A
10436402	Cldnd1	N/A	N/A
10436412	Olfr195	N/A	N/A
10436426	Orai2	N/A	N/A
10436428	Mina /// Crybg3	N/A	N/A
10436442	Fam60a	N/A	N/A
10436444	---	N/A	N/A
10436446	Gps1	N/A	N/A
10436449	---	N/A	N/A
10436456	Pros1	N/A	N/A
10436471	Cggbp1	N/A	N/A
10436498	---	N/A	N/A
10436500	Gbe1	N/A	N/A
10436517	---	N/A	N/A
10436519	Robo1	N/A	N/A
10436550	---	N/A	N/A
10436561	Usp25	N/A	N/A
10436588	2810055G20Rik	N/A	N/A
10436590	2810055G20Rik	N/A	N/A
10436594	---	N/A	N/A
10436596	2810055G20Rik	N/A	N/A
10436598	2810055G20Rik	N/A	N/A
10436600	Mir99a	N/A	N/A
10436602	Mirlet7c-1	N/A	N/A
10436606	---	N/A	N/A
10436608	Cxadr	N/A	N/A
10436634	Gm16509	N/A	N/A
10436636	Ncam2	N/A	N/A
10436658	7120432I05Rik	N/A	N/A
10436662	Mir155	N/A	N/A
10436664	---	N/A	N/A
10436666	Jam2	N/A	N/A
10436678	Gabpa	N/A	N/A
10436692	Gm10791	N/A	N/A
10436694	Rplp0	N/A	N/A
10436696	---	N/A	N/A
10436708	Usp16	N/A	N/A
10436727	ORF63	N/A	N/A
10436746	Krtap13-1	N/A	N/A
10436748	2310034C09Rik	N/A	N/A
10436750	Gm5965	N/A	N/A
10436758	Gm10229	N/A	N/A
10436760	Krtap6-1	N/A	N/A
10436762	---	N/A	N/A
10436764	Krtap6-3	N/A	N/A
10436766	Krtap20-2 /// LOC100048551 /// 1110057P08Rik	N/A	N/A
10436768	Gm10061	N/A	N/A
10436773	Gm9789 /// Gm7735	N/A	N/A
10436775	1110057P08Rik	N/A	N/A
10436779	---	N/A	N/A
10436781	Gm10789	N/A	N/A
10436783	Sod1	N/A	N/A
10436788	Hunk	N/A	N/A
10436800	1110008E08Rik	N/A	N/A
10436802	---	N/A	N/A
10436804	Mrap	N/A	N/A
10436823	Olig2	N/A	N/A

10436826	---	N/A	N/A
10436828	Olig1	N/A	N/A
10436830	Ifnar2	N/A	N/A
10436841	Il10rb /// Ifnar2	N/A	N/A
10436849	Ifnar1	N/A	N/A
10436873	Son	N/A	N/A
10436890	Gm10785	N/A	N/A
10436892	Itsn1	N/A	N/A
10436941	Mrps6 /// Slc5a3	N/A	N/A
10436945	Slc5a3	N/A	N/A
10436947	Kcne2	N/A	N/A
10436951	Fam165b	N/A	N/A
10436958	Clic6	N/A	N/A
10436967	Cbr1	N/A	N/A
10436976	---	N/A	N/A
10436983	Dopey2	N/A	N/A
10437023	Morc3	N/A	N/A
10437080	Ttc3	N/A	N/A
10437132	---	N/A	N/A
10437134	Dyrk1a	N/A	N/A
10437136	Dyrk1a	N/A	N/A
10437149	---	N/A	N/A
10437160	Ets2	N/A	N/A
10437171	---	N/A	N/A
10437174	Wrb	N/A	N/A
10437191	B3galt5	N/A	N/A
10437205	Pcp4	N/A	N/A
10437210	Bace2	N/A	N/A
10437222	Hnrnpa3	N/A	N/A
10437236	B230307C23Rik	N/A	N/A
10437239	---	N/A	N/A
10437257	Zfp597	N/A	N/A
10437270	Cluap1	N/A	N/A
10437292	Btbd12	N/A	N/A
10437311	Trap1	N/A	N/A
10437332	Crebbp	N/A	N/A
10437376	Srl	N/A	N/A
10437384	Tcfap4	N/A	N/A
10437392	Pam16	N/A	N/A
10437443	5730403B10Rik /// Hmox2	N/A	N/A
10437451	Fam100a	N/A	N/A
10437454	Anks3	N/A	N/A
10437499	Glyr1	N/A	N/A
10437573	---	N/A	N/A
10437580	---	N/A	N/A
10437582	---	N/A	N/A
10437594	Usp7	N/A	N/A
10437627	Rpl35a /// Ino80 /// Gm10247	N/A	N/A
10437629	Grin2a	N/A	N/A
10437639	Emp2	N/A	N/A
10437655	Fam18a /// Nubp1	N/A	N/A
10437664	Dexi	N/A	N/A
10437677	Prm3	N/A	N/A
10437684	Prm1	N/A	N/A
10437687	Litaf	N/A	N/A
10437695	Litaf /// Gm9861	N/A	N/A
10437698	Txndc11	N/A	N/A
10437712	Zc3h7a	N/A	N/A
10437735	---	N/A	N/A
10437737	Rsl1d1	N/A	N/A
10437748	Gspt1	N/A	N/A
10437765	Cpped1	N/A	N/A
10437773	---	N/A	N/A
10437775	2310015D24Rik	N/A	N/A
10437813	---	N/A	N/A
10437817	Pdxdc1	N/A	N/A
10437846	Ifitm7	N/A	N/A
10437852	4921513D23Rik	N/A	N/A
10437928	0610037P05Rik	N/A	N/A
10437934	Rpsa /// Rpsa-ps10	N/A	N/A
10437940	---	N/A	N/A
10437942	Ube2v2	N/A	N/A
10437945	Mcm4	N/A	N/A
10437963	Mzt2	N/A	N/A
10437992	Dnm1l	N/A	N/A
10438040	Olfr19	N/A	N/A
10438049	Spag6	N/A	N/A
10438060	Igll1	N/A	N/A
10438069	Ypel1	N/A	N/A
10438071	Ppil2	N/A	N/A
10438094	Mir130b	N/A	N/A
10438096	Mir301b	N/A	N/A
10438112	Pi4ka	N/A	N/A
10438169	---	N/A	N/A

10438178	4930451C15Rik	N/A	N/A
10438189	Slc7a4	N/A	N/A
10438232	Dgcr2	N/A	N/A
10438245	Dgcr14	N/A	N/A
10438262	Slc25a1	N/A	N/A
10438272	Prodh	N/A	N/A
10438293	Zdhhc8	N/A	N/A
10438308	Ranbp1	N/A	N/A
10438313	Dgcr8 /// Trmt2a	N/A	N/A
10438328	D16H22S680E	N/A	N/A
10438338	Mir185	N/A	N/A
10438340	Comt1	N/A	N/A
10438358	Gp1bb /// Sept5	N/A	N/A
10438376	Rps2 /// Rps2-ps6 /// Gm5921 /// Gm6139	N/A	N/A
10438400	Mrpl40	N/A	N/A
10438421	Olfr164	N/A	N/A
10438423	Olfr165	N/A	N/A
10438425	Olfr167	N/A	N/A
10438427	Olfr168	N/A	N/A
10438429	Olfr170 /// Olfr171 /// Olfr169	N/A	N/A
10438431	Olfr170	N/A	N/A
10438433	Olfr171	N/A	N/A
10438442	A930003A15Rik	N/A	N/A
10438454	Sumo2	N/A	N/A
10438460	Parl	N/A	N/A
10438478	Abcc5	N/A	N/A
10438515	Gm15760	N/A	N/A
10438517	Alg3	N/A	N/A
10438527	Camk2n2	N/A	N/A
10438530	Clcn2	N/A	N/A
10438564	Rps10	N/A	N/A
10438570	Magef1	N/A	N/A
10438572	2510009E07Rik	N/A	N/A
10438575	Ehhadh	N/A	N/A
10438583	Rpl12	N/A	N/A
10438585	Tmem41a	N/A	N/A
10438621	Tra2b	N/A	N/A
10438639	Dgkg	N/A	N/A
10438666	---	N/A	N/A
10438672	Tbccd1	N/A	N/A
10438681	Kng2	N/A	N/A
10438708	Masp1	N/A	N/A
10438726	---	N/A	N/A
10438730	Sst	N/A	N/A
10438738	Bcl6	N/A	N/A
10438753	Leprel1	N/A	N/A
10438801	Fgf12	N/A	N/A
10438813	Mir690	N/A	N/A
10438815	1600021P15Rik	N/A	N/A
10438822	Atp13a5	N/A	N/A
10438854	Atp13a4	N/A	N/A
10438889	9530020O07Rik	N/A	N/A
10438899	Cpn2	N/A	N/A
10438904	Lrrc15	N/A	N/A
10438907	Gp5	N/A	N/A
10438909	Atp13a3	N/A	N/A
10438911	Atp13a3	N/A	N/A
10438942	Tmem44	N/A	N/A
10438959	Lsg1	N/A	N/A
10438975	AI480653	N/A	N/A
10438980	Acap2	N/A	N/A
10439005	Ppp1r2	N/A	N/A
10439009	Apod	N/A	N/A
10439016	Gm5415 /// 4930444G20Rik /// AF366264 /// Gm9839	N/A	N/A
10439018	0610012G03Rik	N/A	N/A
10439021	Senp5	N/A	N/A
10439032	Pak2	N/A	N/A
10439063	Fbxo45	N/A	N/A
10439080	---	N/A	N/A
10439084	Gm10818	N/A	N/A
10439092	1700021K19Rik	N/A	N/A
10439114	Iqcg	N/A	N/A
10439126	---	N/A	N/A
10439130	Umps	N/A	N/A
10439138	Kalrn	N/A	N/A
10439204	Rpl15 /// Gm4294	N/A	N/A
10439206	---	N/A	N/A
10439208	Sec22a	N/A	N/A
10439216	---	N/A	N/A
10439218	Pdia5	N/A	N/A
10439237	Rps21	N/A	N/A
10439239	Dirc2	N/A	N/A
10439268	Dtx3l /// Parp9	N/A	N/A
10439285	Gm6815	N/A	N/A

10439321	Slc15a2	N/A	N/A
10439357	Fbxo40	N/A	N/A
10439362	Stxbp5l	N/A	N/A
10439392	Gtf2e1	N/A	N/A
10439398	Ndufb4	N/A	N/A
10439402	Rpl9 /// Gm5451	N/A	N/A
10439404	---	N/A	N/A
10439409	BC031361	N/A	N/A
10439411	Nr1i2	N/A	N/A
10439424	4932425I24Rik	N/A	N/A
10439442	Pla1a	N/A	N/A
10439455	Adprh	N/A	N/A
10439463	4930455C21Rik	N/A	N/A
10439471	Poglut1	N/A	N/A
10439483	Arhgap31	N/A	N/A
10439498	---	N/A	N/A
10439520	---	N/A	N/A
10439523	---	N/A	N/A
10439527	Tigit	N/A	N/A
10439532	Qtrtd1	N/A	N/A
10439542	Zdhhc23	N/A	N/A
10439547	Gramd1c	N/A	N/A
10439566	Atp6v1a	N/A	N/A
10439634	Gtpbp8	N/A	N/A
10439642	Slc35a5	N/A	N/A
10439651	Cd200	N/A	N/A
10439695	Tagln3	N/A	N/A
10439701	Abhd10	N/A	N/A
10439710	Phldb2	N/A	N/A
10439762	Ahcy	N/A	N/A
10439766	Pvrl3	N/A	N/A
10439788	---	N/A	N/A
10439798	Dzip3	N/A	N/A
10439830	---	N/A	N/A
10439835	Gm4802	N/A	N/A
10439854	Bbx	N/A	N/A
10439874	Ccdc54	N/A	N/A
10439878	Psmc1	N/A	N/A
10439885	---	N/A	N/A
10439887	---	N/A	N/A
10439889	---	N/A	N/A
10439891	---	N/A	N/A
10439895	Alcam	N/A	N/A
10439915	---	N/A	N/A
10439932	---	N/A	N/A
10439934	---	N/A	N/A
10439955	Fam55c	N/A	N/A
10439960	Cep97	N/A	N/A
10439974	---	N/A	N/A
10439976	2310061J03Rik	N/A	N/A
10439985	Rg9mtd1	N/A	N/A
10439989	---	N/A	N/A
10440037	Nit2	N/A	N/A
10440050	Tbc1d23	N/A	N/A
10440077	2610528E23Rik	N/A	N/A
10440097	---	N/A	N/A
10440099	St3gal6 /// Dcbld2	N/A	N/A
10440118	---	N/A	N/A
10440131	Gpr15	N/A	N/A
10440136	Olfr173	N/A	N/A
10440149	Olfr186	N/A	N/A
10440160	Olfr192	N/A	N/A
10440164	Olfr203 /// Olfr194	N/A	N/A
10440168	Olfr197	N/A	N/A
10440172	Olfr199	N/A	N/A
10440174	Olfr201	N/A	N/A
10440176	Olfr202	N/A	N/A
10440180	Olfr205 /// Olfr204	N/A	N/A
10440182	Olfr206	N/A	N/A
10440184	Olfr208 /// Olfr207 /// Olfr209	N/A	N/A
10440186	Crybg3 /// Mina	N/A	N/A
10440206	Arl6	N/A	N/A
10440216	Epha6	N/A	N/A
10440238	Nsun3	N/A	N/A
10440246	Arl13b	N/A	N/A
10440279	Csnka2ip	N/A	N/A
10440284	4930453N24Rik	N/A	N/A
10440288	Zfp654	N/A	N/A
10440292	---	N/A	N/A
10440300	---	N/A	N/A
10440302	Htr1f	N/A	N/A
10440307	Chmp2b	N/A	N/A
10440314	Cadm2	N/A	N/A
10440329	9330155M09Rik	N/A	N/A

10440338	4930423O20Rik	N/A	N/A
10440340	---	N/A	N/A
10440342	---	N/A	N/A
10440388	Hspa13	N/A	N/A
10440406	Nrip1	N/A	N/A
10440414	Fau	N/A	N/A
10440417	9430053O09Rik	N/A	N/A
10440425	---	N/A	N/A
10440427	D16Ertd472e	N/A	N/A
10440465	---	N/A	N/A
10440467	Rpl21 /// Rpl21-ps5	N/A	N/A
10440469	---	N/A	N/A
10440471	Mrpl39	N/A	N/A
10440491	App	N/A	N/A
10440513	Cyyr1	N/A	N/A
10440534	Adamts5	N/A	N/A
10440543	Ltn1	N/A	N/A
10440550	Ltn1	N/A	N/A
10440552	Ltn1	N/A	N/A
10440554	Ltn1	N/A	N/A
10440556	Ltn1	N/A	N/A
10440558	Ltn1	N/A	N/A
10440560	Ltn1	N/A	N/A
10440562	Ltn1	N/A	N/A
10440564	Ltn1	N/A	N/A
10440566	Ltn1	N/A	N/A
10440568	Ltn1	N/A	N/A
10440570	Ltn1	N/A	N/A
10440572	Ltn1	N/A	N/A
10440574	Ltn1	N/A	N/A
10440578	Ltn1	N/A	N/A
10440591	---	N/A	N/A
10440600	Cct8	N/A	N/A
10440617	---	N/A	N/A
10440619	---	N/A	N/A
10440621	Grik1	N/A	N/A
10440643	Cldn17	N/A	N/A
10440651	Krtap24-1	N/A	N/A
10440653	---	N/A	N/A
10440655	2310079G19Rik	N/A	N/A
10440664	2310061N02Rik	N/A	N/A
10440690	Krtap16-4	N/A	N/A
10440698	---	N/A	N/A
10440700	AY026312	N/A	N/A
10440708	---	N/A	N/A
10440711	Gm10228	N/A	N/A
10440717	Gm10229 /// Krtap16-8	N/A	N/A
10440719	---	N/A	N/A
10440727	Krtap8-1	N/A	N/A
10440734	---	N/A	N/A
10440738	Tiam1	N/A	N/A
10440770	Srsf15	N/A	N/A
10440794	2610039C10Rik	N/A	N/A
10440840	1110004E09Rik	N/A	N/A
10440849	Synj1	N/A	N/A
10440881	Gcfc1	N/A	N/A
10440903	4932438H23Rik	N/A	N/A
10440909	---	N/A	N/A
10440914	Gm9881	N/A	N/A
10440916	---	N/A	N/A
10440918	Tmem50b	N/A	N/A
10440926	Dnajc28	N/A	N/A
10440929	Gart	N/A	N/A
10440953	Donson	N/A	N/A
10440964	Cryzl1	N/A	N/A
10440993	Rcan1	N/A	N/A
10441017	Setd4	N/A	N/A
10441038	Hlcs	N/A	N/A
10441053	---	N/A	N/A
10441064	Dscr3	N/A	N/A
10441091	6530402D11Rik	N/A	N/A
10441107	Psmg1	N/A	N/A
10441115	Brwd1	N/A	N/A
10441195	Dscam	N/A	N/A
10441231	Bace2	N/A	N/A
10441313	C2cd2	N/A	N/A
10441330	Zfp295	N/A	N/A
10441342	Synj2bp /// Cox16	N/A	N/A
10441344	Rbm16	N/A	N/A
10441359	---	N/A	N/A
10441392	Cldn20	N/A	N/A
10441394	---	N/A	N/A
10441396	Ppia	N/A	N/A
10441398	---	N/A	N/A

10441400	Arid1b	N/A	N/A
10441422	Zdhhc14	N/A	N/A
10441436	Snx9	N/A	N/A
10441489	Gtf2h5	N/A	N/A
10441494	Tulp4	N/A	N/A
10441497	Tulp4	N/A	N/A
10441509	Ppp1r2 /// Gm5972	N/A	N/A
10441554	---	N/A	N/A
10441556	Gm1604b	N/A	N/A
10441565	Rps6ka2	N/A	N/A
10441610	Rsph3a /// Rsph3b	N/A	N/A
10441620	Fgfr1op	N/A	N/A
10441642	Brp44l	N/A	N/A
10441646	Sft2d1	N/A	N/A
10441657	Prr18	N/A	N/A
10441680	Pde10a	N/A	N/A
10441740	Agpat4	N/A	N/A
10441751	---	N/A	N/A
10441753	Plg	N/A	N/A
10441787	Airn	N/A	N/A
10441791	Airn	N/A	N/A
10441797	Tcp1	N/A	N/A
10441811	---	N/A	N/A
10441813	Snora20	N/A	N/A
10441856	Gm10512	N/A	N/A
10441862	Smok2b	N/A	N/A
10441864	Mllt4	N/A	N/A
10441917	---	N/A	N/A
10441923	Gm3435 /// 2210404J11Rik /// 9030025P20Rik	N/A	N/A
10441933	2210404J11Rik /// Gm3435 /// 9030025P20Rik	N/A	N/A
10441952	Gm3435 /// 2210404J11Rik /// 9030025P20Rik	N/A	N/A
10441954	2210404J11Rik	N/A	N/A
10441956	Fam120b	N/A	N/A
10441973	Tbp	N/A	N/A
10441987	Chd1	N/A	N/A
10442025	Gm9943	N/A	N/A
10442027	---	N/A	N/A
10442030	---	N/A	N/A
10442037	Zfp97 /// BC018101	N/A	N/A
10442052	Gm6712	N/A	N/A
10442057	Riok2	N/A	N/A
10442069	Lix1	N/A	N/A
10442081	Mir99b	N/A	N/A
10442083	Mirlet7e	N/A	N/A
10442085	Mir125a	N/A	N/A
10442087	Ncrna00085	N/A	N/A
10442104	Fpr-rs4	N/A	N/A
10442120	---	N/A	N/A
10442123	---	N/A	N/A
10442139	Vmn1r224	N/A	N/A
10442141	Vmn1r225	N/A	N/A
10442143	Gm5145	N/A	N/A
10442145	Vmn1r226	N/A	N/A
10442149	Vmn1r229	N/A	N/A
10442151	Vmn1r230	N/A	N/A
10442153	---	N/A	N/A
10442155	Ppp2r1a	N/A	N/A
10442172	Zfp160	N/A	N/A
10442219	Zfp52	N/A	N/A
10442224	BC049807	N/A	N/A
10442236	Gm10509 /// 3110052M02Rik	N/A	N/A
10442238	Zfp51 /// 3110052M02Rik	N/A	N/A
10442240	Zfp760	N/A	N/A
10442250	Zfp229	N/A	N/A
10442254	---	N/A	N/A
10442256	---	N/A	N/A
10442258	4930432O21Rik	N/A	N/A
10442262	Zfp758	N/A	N/A
10442270	1300003B13Rik	N/A	N/A
10442285	---	N/A	N/A
10442292	Vmn2r113	N/A	N/A
10442300	---	N/A	N/A
10442321	Thoc6 /// Hcfc1r1	N/A	N/A
10442341	Srrm2	N/A	N/A
10442370	Dcpp1 /// Dcpp2	N/A	N/A
10442373	Dcpp2	N/A	N/A
10442391	---	N/A	N/A
10442393	Ntn3 /// BC028777 /// 1600002H07Rik	N/A	N/A
10442396	Abca3	N/A	N/A
10442435	Rnps1	N/A	N/A
10442454	Pgp	N/A	N/A
10442458	9930021D14Rik /// Mlst8	N/A	N/A
10442468	Caskin1	N/A	N/A
10442493	---	N/A	N/A

10442495	Pkd1	N/A	N/A
10442549	Zfp598	N/A	N/A
10442580	Rps2	N/A	N/A
10442596	Sepx1	N/A	N/A
10442606	4930528F23Rik	N/A	N/A
10442616	Hagh	N/A	N/A
10442625	Igfals	N/A	N/A
10442629	Spsb3	N/A	N/A
10442643	Nme3 /// Mrps34	N/A	N/A
10442649	Mapk8ip3	N/A	N/A
10442736	BC003965	N/A	N/A
10442739	Unkl	N/A	N/A
10442769	---	N/A	N/A
10442816	Lmf1	N/A	N/A
10442827	---	N/A	N/A
10442829	Gng13 /// Chtf18	N/A	N/A
10442834	Rpusd1	N/A	N/A
10442887	Fbxl16	N/A	N/A
10442904	Jmjd8 /// Stub1	N/A	N/A
10442914	0610011F06Rik	N/A	N/A
10442922	Cox7c /// Gm10012	N/A	N/A
10442932	Tmem8	N/A	N/A
10442948	Mrpl28	N/A	N/A
10442986	Luc7l	N/A	N/A
10443007	Neurl1B	N/A	N/A
10443009	Ergic1	N/A	N/A
10443027	A930001N09Rik	N/A	N/A
10443039	Bnip1	N/A	N/A
10443063	Phf1	N/A	N/A
10443080	Syngap1	N/A	N/A
10443087	Syngap1	N/A	N/A
10443089	Syngap1	N/A	N/A
10443091	Syngap1	N/A	N/A
10443095	Syngap1	N/A	N/A
10443108	Syngap1	N/A	N/A
10443110	Syngap1	N/A	N/A
10443117	Bak1	N/A	N/A
10443120	Ggnbp1	N/A	N/A
10443191	Gm10505	N/A	N/A
10443193	Rpl30 /// Gm12191	N/A	N/A
10443199	AI413582 /// C130040N14Rik	N/A	N/A
10443201	Pacsin1	N/A	N/A
10443221	Uhrf1bp1	N/A	N/A
10443344	---	N/A	N/A
10443360	Rpl10a /// Rpl10a-ps2	N/A	N/A
10443367	E230001N04Rik	N/A	N/A
10443378	Gm749	N/A	N/A
10443391	Mapk14	N/A	N/A
10443421	Brpf3	N/A	N/A
10443449	Kctd20	N/A	N/A
10443459	Srsf3	N/A	N/A
10443482	BC004004	N/A	N/A
10443492	pl21 /// Gm6813 /// Gm9104 /// Rpl21-ps4 /// Rpl21-ps7 /// Gm16416 /// Rpl21-ps12 /// Rpl21-ps14 /// Rpl21-ps10 /// Gm80	N/A	N/A
10443527	Pim1	N/A	N/A
10443535	Tbc1d22b	N/A	N/A
10443550	Rnf8	N/A	N/A
10443561	Ftsjd2	N/A	N/A
10443589	Zfand3	N/A	N/A
10443596	Gm9874	N/A	N/A
10443690	Glp1r	N/A	N/A
10443764	Slc37a1	N/A	N/A
10443808	Ndufv3	N/A	N/A
10443814	---	N/A	N/A
10443817	Pknox1	N/A	N/A
10443836	Rrp1b	N/A	N/A
10443852	A530088E08Rik /// Rasal3	N/A	N/A
10443898	Cyp4f15	N/A	N/A
10443918	C920016K16Rik	N/A	N/A
10443940	Zfp955a /// Zfp955b	N/A	N/A
10443946	Actl9	N/A	N/A
10444008	Zfp414	N/A	N/A
10444041	Ndufa7	N/A	N/A
10444066	Zbtb22	N/A	N/A
10444068	Tapbp	N/A	N/A
10444079	Rgl2	N/A	N/A
10444114	Vps52	N/A	N/A
10444135	AA388235	N/A	N/A
10444244	Tap1	N/A	N/A
10444291	H2-Ab1	N/A	N/A
10444394	Pbx2	N/A	N/A
10444420	Egfl8 /// Agpat1	N/A	N/A
10444431	Prrt1	N/A	N/A
10444436	Fkbpl	N/A	N/A
10444439	Atf6b	N/A	N/A

10444496	Dom3z	N/A	N/A
10444524	Ehmt2	N/A	N/A
10444589	Hspa1b	N/A	N/A
10444595	Lsm2	N/A	N/A
10444656	---	N/A	N/A
10444658	Clic1	N/A	N/A
10444685	Bat5	N/A	N/A
10444713	Bat4	N/A	N/A
10444717	D17H6S53E	N/A	N/A
10444756	Atp6v1g2	N/A	N/A
10444761	Bat1a	N/A	N/A
10444778	---	N/A	N/A
10444780	H2-L /// H2-D1	N/A	N/A
10444800	Nhp2l1	N/A	N/A
10444802	H2-Q2 /// H2-Q1	N/A	N/A
10444810	Gm6571 /// H2-Q1	N/A	N/A
10444814	H2-gs10 /// H2-Q5	N/A	N/A
10444821	H2-Q8	N/A	N/A
10444824	LOC68395 /// H2-Q6	N/A	N/A
10444830	H2-Q7 /// H2-Q6 /// H2-Q8	N/A	N/A
10444841	H2-Q10	N/A	N/A
10444883	Cdsn	N/A	N/A
10444890	Ier3	N/A	N/A
10444895	Flot1	N/A	N/A
10444911	Mdc1	N/A	N/A
10444927	Nrm	N/A	N/A
10444936	Dhx16	N/A	N/A
10444957	Ppp1r10	N/A	N/A
10444978	Gnl1	N/A	N/A
10444995	---	N/A	N/A
10445006	Akirin1 /// Gm6623	N/A	N/A
10445008	Lsm5	N/A	N/A
10445033	Trim26	N/A	N/A
10445061	Rnf39	N/A	N/A
10445067	Znrd1as	N/A	N/A
10445071	Zfp57	N/A	N/A
10445078	Gabbr1	N/A	N/A
10445107	Gm4831	N/A	N/A
10445117	Olfr96	N/A	N/A
10445135	Olfr108	N/A	N/A
10445137	Olfr109	N/A	N/A
10445139	Olfr110	N/A	N/A
10445141	Olfr111	N/A	N/A
10445151	Olfr120	N/A	N/A
10445156	Olfr123	N/A	N/A
10445165	Olfr127	N/A	N/A
10445172	Olfr130	N/A	N/A
10445183	Olfr136	N/A	N/A
10445185	Rn18s	N/A	N/A
10445214	Mut /// Cenpq	N/A	N/A
10445229	---	N/A	N/A
10445232	3110082D06Rik	N/A	N/A
10445235	Gm9753	N/A	N/A
10445237	3110082D06Rik	N/A	N/A
10445239	Gm5978	N/A	N/A
10445241	Tnfrsf21	N/A	N/A
10445251	Gpr110	N/A	N/A
10445268	Gpr116	N/A	N/A
10445291	---	N/A	N/A
10445293	Pla2g7	N/A	N/A
10445323	---	N/A	N/A
10445325	Rcan2	N/A	N/A
10445338	Enpp5	N/A	N/A
10445347	Clic5	N/A	N/A
10445360	Supt3h	N/A	N/A
10445373	B230354K17Rik	N/A	N/A
10445376	Rpl21	N/A	N/A
10445407	Tcte1 /// Tmem151b	N/A	N/A
10445428	---	N/A	N/A
10445430	Mrpl14	N/A	N/A
10445434	Mrps18a	N/A	N/A
10445442	Gtpbp2	N/A	N/A
10445458	Xpo5	N/A	N/A
10445494	Mir693	N/A	N/A
10445496	Yipf3	N/A	N/A
10445531	Zfp318	N/A	N/A
10445542	---	N/A	N/A
10445544	Crip3	N/A	N/A
10445558	BC048355	N/A	N/A
10445565	Mrpl2	N/A	N/A
10445601	Mea1	N/A	N/A
10445607	Pex6	N/A	N/A
10445627	2310039H08Rik	N/A	N/A
10445638	Ubr2	N/A	N/A

10445640	Trerf1	N/A	N/A
10445664	Guca1b	N/A	N/A
10445670	Mrps10	N/A	N/A
10445674	Mrfap1	N/A	N/A
10445678	AI661453 /// 1700001C19Rik	N/A	N/A
10445695	Med20	N/A	N/A
10445702	Usp49	N/A	N/A
10445741	---	N/A	N/A
10445781	Trem2	N/A	N/A
10445796	AI314976	N/A	N/A
10445824	---	N/A	N/A
10445826	Mocs1	N/A	N/A
10445867	Plcl2	N/A	N/A
10445875	Btg3 /// Gm7334	N/A	N/A
10445877	Gm16489	N/A	N/A
10445879	Kcnh8	N/A	N/A
10445891	---	N/A	N/A
10445894	Erh	N/A	N/A
10445898	Rab5a	N/A	N/A
10445909	Kat2b	N/A	N/A
10445941	---	N/A	N/A
10445944	St6gal2	N/A	N/A
10445972	BC011426	N/A	N/A
10446001	Fsd1	N/A	N/A
10446013	Mpnd	N/A	N/A
10446063	Tnfaip8l1	N/A	N/A
10446066	A230051N06Rik	N/A	N/A
10446069	Mir7b	N/A	N/A
10446071	Fem1a	N/A	N/A
10446084	Kdm4b	N/A	N/A
10446131	Rpl36 /// Gm13611 /// Gm5745	N/A	N/A
10446149	Ranbp3	N/A	N/A
10446166	Ndufa11	N/A	N/A
10446172	Dus3l	N/A	N/A
10446207	Clpp	N/A	N/A
10446214	Alkbh7	N/A	N/A
10446309	Cntnap5c	N/A	N/A
10446312	Cntnap5c	N/A	N/A
10446334	Glcci1	N/A	N/A
10446341	---	N/A	N/A
10446344	---	N/A	N/A
10446351	Fert2	N/A	N/A
10446376	Man2a1	N/A	N/A
10446423	5430411C19Rik	N/A	N/A
10446425	---	N/A	N/A
10446427	ORF19	N/A	N/A
10446549	Tmem200c	N/A	N/A
10446553	Epb4.1l3	N/A	N/A
10446581	Zfp161	N/A	N/A
10446587	C030034I22Rik	N/A	N/A
10446592	Dlgap1	N/A	N/A
10446594	---	N/A	N/A
10446596	Dlgap1	N/A	N/A
10446613	Gm16519	N/A	N/A
10446615	Rps24 /// Gm6030	N/A	N/A
10446617	---	N/A	N/A
10446656	Lpin2	N/A	N/A
10446713	Snord53	N/A	N/A
10446739	Clip4	N/A	N/A
10446756	Ypel5	N/A	N/A
10446763	Lbh	N/A	N/A
10446771	Lclat1	N/A	N/A
10446777	Ehd3	N/A	N/A
10446785	Spast	N/A	N/A
10446804	Slc30a6	N/A	N/A
10446821	Rps6 /// Gm16409	N/A	N/A
10446833	Birc6	N/A	N/A
10446907	Ttc27	N/A	N/A
10446965	Rasgrp3	N/A	N/A
10446984	---	N/A	N/A
10446986	Crim1	N/A	N/A
10447004	Hdac1	N/A	N/A
10447023	Heatr5b	N/A	N/A
10447025	Ccdc75	N/A	N/A
10447036	---	N/A	N/A
10447038	1110001A16Rik /// Cebpz	N/A	N/A
10447056	Qpct	N/A	N/A
10447065	Fam82a1	N/A	N/A
10447079	---	N/A	N/A
10447082	Cyp1b1	N/A	N/A
10447084	Galm	N/A	N/A
10447097	Gemin6	N/A	N/A
10447100	Morn2 /// Dhx57	N/A	N/A
10447118	Gm9959	N/A	N/A

10447120	Tmem178	N/A	N/A
10447125	---	N/A	N/A
10447128	Gm6594	N/A	N/A
10447130	Pkdcc	N/A	N/A
10447139	Rpl21 /// Gm6813 /// Rpl21-ps4 /// Rpl21-ps7 /// Gm16416	N/A	N/A
10447167	Mta3	N/A	N/A
10447188	Mta3	N/A	N/A
10447222	---	N/A	N/A
10447224	Dync2li1	N/A	N/A
10447239	Abcg8	N/A	N/A
10447264	Prepl /// Slc3a1	N/A	N/A
10447275	1700106N22Rik	N/A	N/A
10447286	Rpl31	N/A	N/A
10447294	Prkce	N/A	N/A
10447315	---	N/A	N/A
10447317	Epas1	N/A	N/A
10447337	1700090G07Rik	N/A	N/A
10447341	Rhoq /// Pigf	N/A	N/A
10447349	Cript	N/A	N/A
10447354	Tmx2	N/A	N/A
10447356	Socs5	N/A	N/A
10447361	Ttc7	N/A	N/A
10447395	Msh2	N/A	N/A
10447412	---	N/A	N/A
10447415	---	N/A	N/A
10447431	Foxn2	N/A	N/A
10447437	Klraq1	N/A	N/A
10447461	Ston1	N/A	N/A
10447477	Gm10493	N/A	N/A
10447480	Nrxn1 /// Gm10308	N/A	N/A
10447483	Nanp	N/A	N/A
10447486	---	N/A	N/A
10447490	Pja2	N/A	N/A
10447502	Adcyap1	N/A	N/A
10447513	---	N/A	N/A
10447515	U2af1	N/A	N/A
10447517	Pisd-ps2	N/A	N/A
10447521	Tfb1m /// Tiam2	N/A	N/A
10447551	5730437N04Rik	N/A	N/A
10447557	Ldhal6b	N/A	N/A
10447564	Zdhhc14	N/A	N/A
10447566	Atp5g2	N/A	N/A
10447569	Serac1 /// Synj2	N/A	N/A
10447589	Fbl	N/A	N/A
10447591	Ftl2 /// Ftl1	N/A	N/A
10447594	Dynlt1a /// Dynlt1b /// Dynlt1f /// Dynlt1c /// Dynlt1e	N/A	N/A
10447617	Mir692-1	N/A	N/A
10447619	Rsph3a /// Rsph3b	N/A	N/A
10447629	Tagap /// Tagap1	N/A	N/A
10447634	Unc93a /// Gm9992	N/A	N/A
10447643	---	N/A	N/A
10447645	5830477G23Rik	N/A	N/A
10447647	Gpr31c	N/A	N/A
10447668	Gm1604b /// Gm1604A	N/A	N/A
10447688	---	N/A	N/A
10447691	---	N/A	N/A
10447693	---	N/A	N/A
10447695	---	N/A	N/A
10447697	6530411M01Rik	N/A	N/A
10447699	---	N/A	N/A
10447702	Ppih /// Gm10069	N/A	N/A
10447708	Qk	N/A	N/A
10447729	Qk	N/A	N/A
10447740	Gm10513	N/A	N/A
10447786	Slc22a1	N/A	N/A
10447799	Igf2r	N/A	N/A
10447880	Mrpl18	N/A	N/A
10447897	Wtap	N/A	N/A
10447902	Gpr31c	N/A	N/A
10447915	---	N/A	N/A
10447919	---	N/A	N/A
10447921	---	N/A	N/A
10447933	LOC100505091	N/A	N/A
10447935	Gm7168	N/A	N/A
10448004	Phf10 /// 1600012H06Rik	N/A	N/A
10448030	---	N/A	N/A
10448032	Gm10510	N/A	N/A
10448055	Pdcd2	N/A	N/A
10448062	---	N/A	N/A
10448079	---	N/A	N/A
10448089	Oaz1 /// Gm9786	N/A	N/A
10448094	Lnpep	N/A	N/A
10448117	Has1	N/A	N/A
10448127	---	N/A	N/A

10448168	Fpr-rs3	N/A	N/A
10448178	Vmn1r232	N/A	N/A
10448180	Vmn1r233	N/A	N/A
10448182	Mir703	N/A	N/A
10448184	4930515G13Rik	N/A	N/A
10448192	---	N/A	N/A
10448195	3110048L19Rik	N/A	N/A
10448202	Tpm4	N/A	N/A
10448212	---	N/A	N/A
10448214	3110048L19Rik /// 6330416L07Rik	N/A	N/A
10448224	Vmn2r111	N/A	N/A
10448230	LOC280487	N/A	N/A
10448232	Gm16386	N/A	N/A
10448235	A630033E08Rik	N/A	N/A
10448247	Zfp40	N/A	N/A
10448257	Gm5146	N/A	N/A
10448262	Zfp213	N/A	N/A
10448369	Flywch1	N/A	N/A
10448380	Tceb2	N/A	N/A
10448424	Pdpk1	N/A	N/A
10448455	Atp6v0c	N/A	N/A
10448459	Tbc1d24	N/A	N/A
10448495	1600002H07Rik	N/A	N/A
10448557	Abca3	N/A	N/A
10448582	Mlst8	N/A	N/A
10448593	Traf7	N/A	N/A
10448631	Tsc2	N/A	N/A
10448694	Syngr3	N/A	N/A
10448700	Gfer /// Dst	N/A	N/A
10448707	Tbl3	N/A	N/A
10448743	Fahd1	N/A	N/A
10448748	Nubp2	N/A	N/A
10448755	Eme2 /// Mapk8ip3	N/A	N/A
10448765	Mapk8ip3	N/A	N/A
10448836	Tmem204	N/A	N/A
10448865	Gnptg	N/A	N/A
10448878	Baiap3	N/A	N/A
10448912	Ube2i	N/A	N/A
10448973	---	N/A	N/A
10448998	Mslnl	N/A	N/A
10449018	Haghl /// Narfl	N/A	N/A
10449034	Fam173a	N/A	N/A
10449051	Stub1 /// Jmjd8	N/A	N/A
10449142	Fam195a	N/A	N/A
10449148	Wfikkn1	N/A	N/A
10449152	Rab40c	N/A	N/A
10449191	Solh	N/A	N/A
10449207	Rab11fip3	N/A	N/A
10449225	Decr2	N/A	N/A
10449258	Arhgdig	N/A	N/A
10449266	Itfg3	N/A	N/A
10449284	Dusp1	N/A	N/A
10449289	1700049J03Rik	N/A	N/A
10449292	Nkx2-5	N/A	N/A
10449295	Cuta	N/A	N/A
10449303	Bak1	N/A	N/A
10449312	---	N/A	N/A
10449315	2900010M23Rik	N/A	N/A
10449327	Lemd2	N/A	N/A
10449343	Grm4	N/A	N/A
10449356	AI413582	N/A	N/A
10449363	Nudt3 /// Anks1	N/A	N/A
10449370	Rps10	N/A	N/A
10449394	Taf11	N/A	N/A
10449415	C230013L11Rik	N/A	N/A
10449471	Srpk1	N/A	N/A
10449523	Pxt1	N/A	N/A
10449527	Stk38	N/A	N/A
10449545	---	N/A	N/A
10449547	---	N/A	N/A
10449549	Rpl35a /// Ino80 /// Gm10247	N/A	N/A
10449551	Cpne5	N/A	N/A
10449581	Mtch1	N/A	N/A
10449596	---	N/A	N/A
10449598	Tmem217	N/A	N/A
10449602	1110021J02Rik	N/A	N/A
10449631	Btbd9	N/A	N/A
10449644	Glo1	N/A	N/A
10449652	Dnahc8 /// Gm9937	N/A	N/A
10449654	Gm10503	N/A	N/A
10449657	Tff3	N/A	N/A
10449697	Gm9902	N/A	N/A
10449712	Cbs	N/A	N/A
10449731	U2af1	N/A	N/A

10449741	Sik1	N/A	N/A
10449815	Brd4	N/A	N/A
10449839	Akap8	N/A	N/A
10449854	Akap8l	N/A	N/A
10449873	Wiz	N/A	N/A
10449920	Zfp811	N/A	N/A
10449926	Zfp799	N/A	N/A
10449955	Cyp4f13	N/A	N/A
10449971	Zfp763	N/A	N/A
10449977	4921501E09Rik	N/A	N/A
10449979	Morc2b	N/A	N/A
10449989	Zfp422	N/A	N/A
10449999	Zfp101	N/A	N/A
10450006	Hnrnpm /// March2	N/A	N/A
10450055	H2-Ke2	N/A	N/A
10450059	B3galt4	N/A	N/A
10450063	Rps18 /// Gm10260	N/A	N/A
10450089	Ring1	N/A	N/A
10450101	Mir219-1	N/A	N/A
10450103	H2-Ke6	N/A	N/A
10450116	Slc39a7	N/A	N/A
10450126	Brd2	N/A	N/A
10450154	H2-Aa	N/A	N/A
10450189	Btnl5	N/A	N/A
10450197	Btnl5	N/A	N/A
10450206	Rnf5	N/A	N/A
10450224	---	N/A	N/A
10450226	Ppt2	N/A	N/A
10450242	C4a /// C4b	N/A	N/A
10450265	Stk19	N/A	N/A
10450325	Cfb	N/A	N/A
10450344	C2	N/A	N/A
10450363	Snord52	N/A	N/A
10450365	---	N/A	N/A
10450367	Hspa1b /// Hspa1a	N/A	N/A
10450369	Hspa1a	N/A	N/A
10450372	Hspa1l	N/A	N/A
10450435	Csnk2b	N/A	N/A
10450444	Apom	N/A	N/A
10450451	Bat2	N/A	N/A
10450482	---	N/A	N/A
10450514	Nfkbil1	N/A	N/A
10450519	Tcf19	N/A	N/A
10450579	Ddr1	N/A	N/A
10450603	---	N/A	N/A
10450605	Tubb5	N/A	N/A
10450614	2310061I04Rik	N/A	N/A
10450622	Atat1	N/A	N/A
10450640	Mrps18b	N/A	N/A
10450646	Rbx1	N/A	N/A
10450648	Abcf1	N/A	N/A
10450669	Prr3	N/A	N/A
10450675	H2-T24	N/A	N/A
10450694	H2-T22	N/A	N/A
10450733	H2-t9 /// EG547347	N/A	N/A
10450744	---	N/A	N/A
10450752	Trim39	N/A	N/A
10450762	H2-M10.2	N/A	N/A
10450777	---	N/A	N/A
10450796	Armcx5	N/A	N/A
10450798	---	N/A	N/A
10450814	Ppp1r11	N/A	N/A
10450843	H2-M5	N/A	N/A
10450852	Olfr90	N/A	N/A
10450854	Olfr91	N/A	N/A
10450856	Olfr92	N/A	N/A
10450858	Olfr93	N/A	N/A
10450860	Olfr90 /// Olfr92	N/A	N/A
10450862	Olfr94	N/A	N/A
10450864	Olfr95	N/A	N/A
10450870	---	N/A	N/A
10450874	Olfr101	N/A	N/A
10450876	Olfr100 /// Olfr102	N/A	N/A
10450878	Olfr103	N/A	N/A
10450895	Olfr114	N/A	N/A
10450900	Olfr116	N/A	N/A
10450902	Olfr117	N/A	N/A
10450904	Scoc	N/A	N/A
10450906	Olfr127	N/A	N/A
10450910	Olfr131	N/A	N/A
10450912	Olfr132	N/A	N/A
10450914	Olfr137	N/A	N/A
10450920	AY036118	N/A	N/A
10451004	Cd2ap	N/A	N/A

10451039	Slc25a27	N/A	N/A
10451052	Gm10498	N/A	N/A
10451077	---	N/A	N/A
10451079	4930564C03Rik	N/A	N/A
10451081	Ccdc94	N/A	N/A
10451110	Hsp90ab1	N/A	N/A
10451123	Slc29a1	N/A	N/A
10451142	Gm7325	N/A	N/A
10451167	Tmem63b	N/A	N/A
10451193	E030047D23Rik	N/A	N/A
10451198	Vegfa	N/A	N/A
10451225	Polh	N/A	N/A
10451238	Polr1c	N/A	N/A
10451291	Slc22a7	N/A	N/A
10451303	Ttbk1	N/A	N/A
10451363	Srf	N/A	N/A
10451395	Klc4	N/A	N/A
10451413	Rrp36	N/A	N/A
10451421	Klhdc3	N/A	N/A
10451434	Ppp2r5d	N/A	N/A
10451451	Gnmt	N/A	N/A
10451458	Cnpy3	N/A	N/A
10451472	Rpl7l1	N/A	N/A
10451481	BC032203	N/A	N/A
10451493	A330017A19Rik	N/A	N/A
10451495	Ubr2	N/A	N/A
10451547	---	N/A	N/A
10451549	Gm4945	N/A	N/A
10451559	AI661453 /// 1700001C19Rik	N/A	N/A
10451580	Bysl	N/A	N/A
10451591	Tomm6 /// Prickle4	N/A	N/A
10451597	Prickle4	N/A	N/A
10451611	---	N/A	N/A
10451650	Nfya	N/A	N/A
10451675	---	N/A	N/A
10451677	---	N/A	N/A
10451708	4930556A20Rik	N/A	N/A
10451710	Rftn1	N/A	N/A
10451736	Tbc1d5	N/A	N/A
10451761	Tbc1d5	N/A	N/A
10451763	Satb1	N/A	N/A
10451784	Kcnh8	N/A	N/A
10451879	Zfp119a	N/A	N/A
10451884	Rpl21 /// Rpl21-ps4 /// Rpl21-ps6	N/A	N/A
10451893	Stap2	N/A	N/A
10451907	Sh3gl1	N/A	N/A
10451932	Plin4	N/A	N/A
10451943	Plin5	N/A	N/A
10451953	Lrg1	N/A	N/A
10451955	Sema6b	N/A	N/A
10451974	Sema6b	N/A	N/A
10451993	D17Wsu104e	N/A	N/A
10452022	Ticam1	N/A	N/A
10452030	Plin3	N/A	N/A
10452043	Arrdc5	N/A	N/A
10452047	Ptprs	N/A	N/A
10452085	Safb2	N/A	N/A
10452087	Safb2	N/A	N/A
10452110	2410015M20Rik	N/A	N/A
10452118	Lonp1	N/A	N/A
10452188	Mllt1	N/A	N/A
10452213	Gtf2f1	N/A	N/A
10452228	Khsrp	N/A	N/A
10452257	Slc25a23	N/A	N/A
10452316	C3	N/A	N/A
10452356	Gpr108	N/A	N/A
10452384	Rpl7a /// Rpl7a-ps3 /// Rpl7a-ps5 /// Gm5619 /// Gm16477 /// Rpl7a-ps10	N/A	N/A
10452404	Nudt12	N/A	N/A
10452415	Gapdh /// Gm2451	N/A	N/A
10452417	---	N/A	N/A
10452419	Efna5	N/A	N/A
10452427	---	N/A	N/A
10452430	Fbxl17	N/A	N/A
10452440	A930002H24Rik	N/A	N/A
10452442	AU016765	N/A	N/A
10452468	Mrps10	N/A	N/A
10452470	Vapa	N/A	N/A
10452480	Txndc2	N/A	N/A
10452485	Rab31	N/A	N/A
10452496	Ralbp1	N/A	N/A
10452508	Twsg1	N/A	N/A
10452516	Ankrd12	N/A	N/A
10452525	Ndufv2	N/A	N/A
10452535	---	N/A	N/A

10452538	1110012J17Rik	N/A	N/A
10452556	Rab12	N/A	N/A
10452571	Ptprm	N/A	N/A
10452639	Myl12b	N/A	N/A
10452643	2900073G15Rik	N/A	N/A
10452658	Smchd1	N/A	N/A
10452701	Smchd1	N/A	N/A
10452721	Trmt61b	N/A	N/A
10452766	---	N/A	N/A
10452768	---	N/A	N/A
10452815	Xdh	N/A	N/A
10452854	Srd5a2	N/A	N/A
10452867	Dpy30	N/A	N/A
10452874	Atp6v1f	N/A	N/A
10452890	Tceb2	N/A	N/A
10452892	Fam98a	N/A	N/A
10452901	---	N/A	N/A
10452903	---	N/A	N/A
10452907	Fez2	N/A	N/A
10452918	Strn	N/A	N/A
10452935	Heatr5b	N/A	N/A
10452937	Heatr5b	N/A	N/A
10452978	Gm6548	N/A	N/A
10452980	Eif2ak2	N/A	N/A
10453006	Cebpz	N/A	N/A
10453026	Prkd3	N/A	N/A
10453047	---	N/A	N/A
10453049	Cdc42ep3	N/A	N/A
10453055	Gm10494	N/A	N/A
10453062	Atl2	N/A	N/A
10453082	Hnrpll	N/A	N/A
10453102	Srsf7	N/A	N/A
10453114	Dhx57 /// Morn2	N/A	N/A
10453139	Gm10190	N/A	N/A
10453141	Sos1	N/A	N/A
10453178	Map4k3	N/A	N/A
10453214	Nutf2 /// Nutf2-ps1	N/A	N/A
10453231	Slc8a1	N/A	N/A
10453233	Slc8a1	N/A	N/A
10453248	---	N/A	N/A
10453250	---	N/A	N/A
10453252	Hmgb1	N/A	N/A
10453254	Cox7a2l	N/A	N/A
10453260	Haao	N/A	N/A
10453272	Zfp36l2	N/A	N/A
10453276	Thada	N/A	N/A
10453318	Abcg5	N/A	N/A
10453334	Lrpprc	N/A	N/A
10453373	Prepl /// Slc3a1	N/A	N/A
10453390	Six3os1	N/A	N/A
10453399	Srbd1	N/A	N/A
10453423	Gm10309	N/A	N/A
10453451	Calm2	N/A	N/A
10453456	Kcnk12	N/A	N/A
10453459	Pnkd	N/A	N/A
10453461	Fbxo11	N/A	N/A
10453485	---	N/A	N/A
10453512	Kpna2	N/A	N/A
10453518	Nrxn1	N/A	N/A
10453542	---	N/A	N/A
10453544	Mettl4	N/A	N/A
10453553	LOC100038746	N/A	N/A
10453555	2610044O15Rik	N/A	N/A
10453560	---	N/A	N/A
10453562	Sft2d1	N/A	N/A
10453573	Olfr63	N/A	N/A
10453600	---	N/A	N/A
10453602	---	N/A	N/A
10453604	Bambi	N/A	N/A
10453616	Mtpap	N/A	N/A
10453627	---	N/A	N/A
10453629	9430020K01Rik	N/A	N/A
10453632	Rpl7a /// Rpl7a-ps3 /// Rpl7a-ps5 /// Gm5619 /// Gm16477	N/A	N/A
10453634	Gm10556	N/A	N/A
10453676	---	N/A	N/A
10453678	Zeb1	N/A	N/A
10453688	---	N/A	N/A
10453690	Rpl27	N/A	N/A
10453692	Vps24	N/A	N/A
10453705	Rab18	N/A	N/A
10453715	---	N/A	N/A
10453717	Fabp5l2	N/A	N/A
10453732	---	N/A	N/A
10453734	---	N/A	N/A

10453736	Unkl	N/A	N/A
10453738	Fzd8	N/A	N/A
10453747	Colec12	N/A	N/A
10453759	Gm10554	N/A	N/A
10453761	---	N/A	N/A
10453764	---	N/A	N/A
10453797	Greb1l	N/A	N/A
10453811	Greb1l	N/A	N/A
10453825	Snrpd1	N/A	N/A
10453830	Mib1	N/A	N/A
10453855	---	N/A	N/A
10453857	Gata6	N/A	N/A
10453887	Cables1	N/A	N/A
10453900	Riok3	N/A	N/A
10453918	3110002H16Rik	N/A	N/A
10454015	Ttc39c	N/A	N/A
10454039	Impact	N/A	N/A
10454057	---	N/A	N/A
10454059	---	N/A	N/A
10454061	---	N/A	N/A
10454063	Rsl24d1	N/A	N/A
10454077	Taf4b	N/A	N/A
10454093	Mrpl27	N/A	N/A
10454095	---	N/A	N/A
10454097	Rpl11 /// Gm7589 /// Gm10288 /// Gm10036	N/A	N/A
10454099	Morf4l1	N/A	N/A
10454103	---	N/A	N/A
10454105	Dsg1c	N/A	N/A
10454192	Ttr	N/A	N/A
10454202	Rnf138	N/A	N/A
10454229	Klhl14	N/A	N/A
10454235	Asxl3	N/A	N/A
10454252	---	N/A	N/A
10454254	Dtna	N/A	N/A
10454286	Mapre2	N/A	N/A
10454296	Rpl19	N/A	N/A
10454298	Zfp397	N/A	N/A
10454306	Zfp35	N/A	N/A
10454310	Galnt1	N/A	N/A
10454332	Elp2	N/A	N/A
10454353	Mocos	N/A	N/A
10454398	AW554918	N/A	N/A
10454411	Hnrnpa1 /// Gm10052 /// Gm5643	N/A	N/A
10454414	Pik3c3	N/A	N/A
10454441	Syt4	N/A	N/A
10454445	Slc25a46	N/A	N/A
10454447	Sap130	N/A	N/A
10454469	Ammecr1l	N/A	N/A
10454478	Polr2d	N/A	N/A
10454512	Sft2d3	N/A	N/A
10454514	Lims2 /// Gpr17	N/A	N/A
10454525	Iws1	N/A	N/A
10454543	---	N/A	N/A
10454546	Map3k2	N/A	N/A
10454564	Ercc3	N/A	N/A
10454580	Bin1	N/A	N/A
10454606	Wdr36	N/A	N/A
10454632	Camk4	N/A	N/A
10454647	Gm10549	N/A	N/A
10454651	---	N/A	N/A
10454653	---	N/A	N/A
10454655	Apc	N/A	N/A
10454731	---	N/A	N/A
10454733	Rpl29 /// Gm3550	N/A	N/A
10454735	Fam53c	N/A	N/A
10454741	Kdm3b	N/A	N/A
10454771	Reep2	N/A	N/A
10454786	Ctnna1	N/A	N/A
10454805	Uba52 /// Gm5239	N/A	N/A
10454807	Snora74a	N/A	N/A
10454809	Matr3	N/A	N/A
10454828	Pnet-ps	N/A	N/A
10454831	Paip2	N/A	N/A
10454836	1700066B19Rik	N/A	N/A
10454840	LOC546711	N/A	N/A
10454842	Ube2d2	N/A	N/A
10454851	Cxxc5	N/A	N/A
10454856	Psd2	N/A	N/A
10454877	Pura	N/A	N/A
10454881	0610010O12Rik	N/A	N/A
10454912	Ankhd1	N/A	N/A
10454944	Ankhd1 /// Eif4ebp3	N/A	N/A
10454953	Tmco6	N/A	N/A
10454966	Ik	N/A	N/A

10454984	Wdr55 /// Dnd1	N/A	N/A
10455007	Zmat2	N/A	N/A
10455017	Gm6756	N/A	N/A
10455019	Pcdha4-g	N/A	N/A
10455048	Pcdhb1	N/A	N/A
10455050	Pcdhb2	N/A	N/A
10455061	Pcdhb4	N/A	N/A
10455065	Pcdhb5	N/A	N/A
10455069	Pcdhb6	N/A	N/A
10455071	Pcdhb7	N/A	N/A
10455078	Pcdhb8	N/A	N/A
10455080	Pcdhb9	N/A	N/A
10455088	Pcdhb11	N/A	N/A
10455092	Pcdhb12	N/A	N/A
10455094	Pcdhb13	N/A	N/A
10455098	Pcdhb14	N/A	N/A
10455104	Pcdhb15	N/A	N/A
10455108	Pcdhb16	N/A	N/A
10455112	Pcdhb17	N/A	N/A
10455118	Pcdhb18	N/A	N/A
10455123	Pcdhb19	N/A	N/A
10455135	Pcdhb21	N/A	N/A
10455139	Pcdhb22	N/A	N/A
10455146	3222401L13Rik	N/A	N/A
10455148	Pcdhgc5	N/A	N/A
10455199	Rell2 /// Fchsd1	N/A	N/A
10455210	Rpl17 /// Gm10268 /// Rpl17-ps3	N/A	N/A
10455212	0610009O20Rik	N/A	N/A
10455227	Rnf14	N/A	N/A
10455238	Ndfip1	N/A	N/A
10455249	9630014M24Rik	N/A	N/A
10455259	Arhgap26	N/A	N/A
10455292	2900055J20Rik	N/A	N/A
10455294	Kctd16	N/A	N/A
10455297	Gm4838	N/A	N/A
10455310	---	N/A	N/A
10455312	Rbm27	N/A	N/A
10455338	pl21 /// Gm6813 /// Gm9104 /// Rpl21-ps4 /// Rpl21-ps7 /// Gm16415 /// Rpl21-ps12 /// Rpl21-ps14 /// Rpl21-ps10 /// Gm80	N/A	N/A
10455346	Tcerg1	N/A	N/A
10455372	C330007P06Rik	N/A	N/A
10455472	Dcp2	N/A	N/A
10455483	Ythdc2	N/A	N/A
10455514	Kcnn2	N/A	N/A
10455531	1700018A14Rik	N/A	N/A
10455533	Eif1a	N/A	N/A
10455542	Ap3s1	N/A	N/A
10455578	Commd10	N/A	N/A
10455588	Hspe1 /// Hspe1-rs1	N/A	N/A
10455599	---	N/A	N/A
10455602	Dmxl1	N/A	N/A
10455647	Tnfaip8	N/A	N/A
10455653	---	N/A	N/A
10455656	Hsd17b4	N/A	N/A
10455687	Prr16	N/A	N/A
10455691	Ftmt	N/A	N/A
10455695	Srfbp1	N/A	N/A
10455705	---	N/A	N/A
10455719	Gykl1	N/A	N/A
10455721	Sncaip	N/A	N/A
10455738	Snx2	N/A	N/A
10455752	Snx24	N/A	N/A
10455769	Csnk1g3	N/A	N/A
10455780	Gapdh	N/A	N/A
10455782	---	N/A	N/A
10455784	Gramd3	N/A	N/A
10455809	1700065I17Rik	N/A	N/A
10455824	Gm10536	N/A	N/A
10455852	Prrc1	N/A	N/A
10455863	Ctxn3	N/A	N/A
10455873	Slc12a2	N/A	N/A
10455912	Isoc1	N/A	N/A
10455942	A730017C20Rik	N/A	N/A
10455948	Chsy3	N/A	N/A
10455954	Gm4951	N/A	N/A
10455957	---	N/A	N/A
10455959	---	N/A	N/A
10455961	Iigp1	N/A	N/A
10455967	2610318N02Rik	N/A	N/A
10455970	BC023105	N/A	N/A
10455974	Dctn4	N/A	N/A
10456001	Rps14	N/A	N/A
10456005	Cd74	N/A	N/A
10456021	Camk2a	N/A	N/A
10456046	Pdgfrb	N/A	N/A

10456120	Csnk1a1	N/A	N/A
10456136	Il17b	N/A	N/A
10456158	Gm9949	N/A	N/A
10456161	Htr4	N/A	N/A
10456184	Apcdd1	N/A	N/A
10456194	Napg	N/A	N/A
10456206	Wdr7	N/A	N/A
10456237	St8sia3	N/A	N/A
10456289	---	N/A	N/A
10456291	Mir122a	N/A	N/A
10456293	Alpk2 /// F730048M01Rik	N/A	N/A
10456296	Malt1	N/A	N/A
10456317	Zfp532	N/A	N/A
10456346	Sec11c	N/A	N/A
10456353	Grp	N/A	N/A
10456361	Gm9926	N/A	N/A
10456363	Gnal	N/A	N/A
10456378	Chmp1b	N/A	N/A
10456383	Impa2	N/A	N/A
10456405	Slmo1	N/A	N/A
10456414	Psmg2	N/A	N/A
10456423	Seh1l	N/A	N/A
10456435	---	N/A	N/A
10456437	---	N/A	N/A
10456486	---	N/A	N/A
10456488	Cep192	N/A	N/A
10456490	Cep192	N/A	N/A
10456492	D18Ertd653e	N/A	N/A
10456501	Rnmt	N/A	N/A
10456519	---	N/A	N/A
10456522	Tcf4	N/A	N/A
10456556	Stard6 /// Gm9795	N/A	N/A
10456579	Mex3c	N/A	N/A
10456585	---	N/A	N/A
10456587	Mro	N/A	N/A
10456599	Gm9925	N/A	N/A
10456601	Cxxc1	N/A	N/A
10456619	---	N/A	N/A
10456622	Mbd1	N/A	N/A
10456650	---	N/A	N/A
10456699	Acaa2	N/A	N/A
10456709	Rpl17 /// Gm10268 /// Rpl17-ps3	N/A	N/A
10456717	Snord58b	N/A	N/A
10456719	---	N/A	N/A
10456721	---	N/A	N/A
10456723	BC031181	N/A	N/A
10456727	Dym	N/A	N/A
10456745	Smad7	N/A	N/A
10456754	Gm10532	N/A	N/A
10456756	Zbtb7c	N/A	N/A
10456812	Hdhd2	N/A	N/A
10456814	Pias2	N/A	N/A
10456836	St8sia5	N/A	N/A
10456887	Loxhd1	N/A	N/A
10456891	Atp5a1	N/A	N/A
10456904	Pstpip2	N/A	N/A
10456922	5430411K18Rik	N/A	N/A
10456970	---	N/A	N/A
10456972	Gm10265	N/A	N/A
10456974	Arf1	N/A	N/A
10456976	Gm10527 /// Gm10526	N/A	N/A
10456978	Gm10527 /// Gm10526	N/A	N/A
10456980	Gm10527 /// Gm10526	N/A	N/A
10456982	Gm10527 /// Gm10526	N/A	N/A
10456984	Gm10527 /// Gm10526	N/A	N/A
10456986	Gm10527 /// Gm10526	N/A	N/A
10457007	Pqlc1	N/A	N/A
10457020	---	N/A	N/A
10457022	Mbp	N/A	N/A
10457038	Gm10524	N/A	N/A
10457040	Zfp516	N/A	N/A
10457048	Gm10523	N/A	N/A
10457054	Zadh2	N/A	N/A
10457071	Cyb5	N/A	N/A
10457091	Neto1	N/A	N/A
10457106	Cbln2	N/A	N/A
10457114	Bhmt /// Bhmt2	N/A	N/A
10457183	Tmx3	N/A	N/A
10457203	---	N/A	N/A
10457205	Crem	N/A	N/A
10457221	---	N/A	N/A
10457223	2610005L07Rik /// 6820431F20Rik	N/A	N/A
10457225	Map3k8	N/A	N/A
10457243	Gm10125	N/A	N/A

10457250	Arhgap12	N/A	N/A
10457273	Kif5b	N/A	N/A
10457302	Epc1	N/A	N/A
10457323	Mkx	N/A	N/A
10457331	Mzt1	N/A	N/A
10457357	Mpp7	N/A	N/A
10457359	Mpp7	N/A	N/A
10457378	Gm9993	N/A	N/A
10457380	---	N/A	N/A
10457385	Ccny	N/A	N/A
10457400	---	N/A	N/A
10457407	---	N/A	N/A
10457409	Usp14 /// Thoc1	N/A	N/A
10457429	Rock1	N/A	N/A
10457463	Nutf2 /// Nutf2-ps1	N/A	N/A
10457475	Abhd3	N/A	N/A
10457485	Mir133a-1	N/A	N/A
10457487	Mir1-2	N/A	N/A
10457489	6030446N20Rik	N/A	N/A
10457508	Npc1	N/A	N/A
10457536	Ankrd29	N/A	N/A
10457583	---	N/A	N/A
10457587	Zfp521	N/A	N/A
10457598	Ss18	N/A	N/A
10457606	Kctd1	N/A	N/A
10457637	---	N/A	N/A
10457640	S100a11	N/A	N/A
10457644	Cdh2	N/A	N/A
10457663	---	N/A	N/A
10457665	4921533I20Rik	N/A	N/A
10457667	LOC280487	N/A	N/A
10457686	Dsc2	N/A	N/A
10457729	---	N/A	N/A
10457731	Rpl35 /// Gm10269	N/A	N/A
10457733	B4galt6	N/A	N/A
10457745	D030074E01Rik	N/A	N/A
10457776	---	N/A	N/A
10457778	Gm10551	N/A	N/A
10457780	Fam59a	N/A	N/A
10457820	Nol4	N/A	N/A
10457834	Nol4	N/A	N/A
10457836	Nol4	N/A	N/A
10457838	Zscan30	N/A	N/A
10457844	Zfp191 /// Zf12	N/A	N/A
10457853	Ino80c	N/A	N/A
10457862	Rprd1a	N/A	N/A
10457872	Slc39a6	N/A	N/A
10457884	Gm9955	N/A	N/A
10457886	Gm9960	N/A	N/A
10457888	5730494M16Rik	N/A	N/A
10457895	Celf4	N/A	N/A
10457916	---	N/A	N/A
10457918	---	N/A	N/A
10457920	Rps25 /// Gm4963	N/A	N/A
10457922	Rpl29	N/A	N/A
10457924	Bloc1s1	N/A	N/A
10457927	---	N/A	N/A
10457929	Rit2	N/A	N/A
10457938	---	N/A	N/A
10457942	Syt4	N/A	N/A
10457948	Slc25a46	N/A	N/A
10457959	Sft2d3	N/A	N/A
10457963	Gpr17	N/A	N/A
10458016	Proc	N/A	N/A
10458044	Osgep	N/A	N/A
10458046	D0H4S114	N/A	N/A
10458084	---	N/A	N/A
10458086	---	N/A	N/A
10458088	Gm10548	N/A	N/A
10458090	Reep5	N/A	N/A
10458098	Fam13b /// Pkd2l2	N/A	N/A
10458122	Nme5	N/A	N/A
10458138	Brd8	N/A	N/A
10458164	Cdc23 /// Kif20a	N/A	N/A
10458213	Etf1	N/A	N/A
10458226	Hspa9	N/A	N/A
10458247	Lrrtm2	N/A	N/A
10458251	Sil1	N/A	N/A
10458262	Slc23a1	N/A	N/A
10458283	Gm1614	N/A	N/A
10458285	Spata24	N/A	N/A
10458293	Dnajc18	N/A	N/A
10458323	Nrg2	N/A	N/A
10458334	Pfdn1	N/A	N/A

10458340	Hbegf	N/A	N/A
10458355	Apbb3 /// Slc35a4	N/A	N/A
10458382	Cd14	N/A	N/A
10458386	Ndufa2	N/A	N/A
10458415	Gm10545	N/A	N/A
10458417	Gm10544	N/A	N/A
10458419	Slc25a2	N/A	N/A
10458424	Taf7	N/A	N/A
10458428	Uxt	N/A	N/A
10458461	Hdac3	N/A	N/A
10458534	Pcdh1	N/A	N/A
10458538	0610009O20Rik /// 1700086O06Rik	N/A	N/A
10458547	Gnpda1 /// Gm8615	N/A	N/A
10458560	Fgf1	N/A	N/A
10458569	Nr3c1	N/A	N/A
10458581	Gm10008	N/A	N/A
10458583	Yipf5	N/A	N/A
10458607	Lars	N/A	N/A
10458645	Ppp2r2b	N/A	N/A
10458661	---	N/A	N/A
10458663	Dpysl3	N/A	N/A
10458680	Eif3j	N/A	N/A
10458685	Jakmip2	N/A	N/A
10458731	Mcc	N/A	N/A
10458755	Ythdc2	N/A	N/A
10458757	Morf4l1	N/A	N/A
10458760	---	N/A	N/A
10458762	---	N/A	N/A
10458764	---	N/A	N/A
10458782	Pggt1b	N/A	N/A
10458784	Pggt1b	N/A	N/A
10458794	Ccdc112	N/A	N/A
10458804	---	N/A	N/A
10458823	Tmed7	N/A	N/A
10458828	Cdo1	N/A	N/A
10458834	Atg12	N/A	N/A
10458841	Ube2l3 /// Gm10145	N/A	N/A
10458843	Sema6a	N/A	N/A
10458870	---	N/A	N/A
10458875	Dtwd2	N/A	N/A
10458882	---	N/A	N/A
10458913	Cep120	N/A	N/A
10458938	---	N/A	N/A
10458940	Zfp608	N/A	N/A
10458956	---	N/A	N/A
10458958	---	N/A	N/A
10458960	Aldh7a1 /// Phax	N/A	N/A
10458983	March3	N/A	N/A
10458992	C330018D20Rik	N/A	N/A
10459066	Gm4841	N/A	N/A
10459089	Ndst1	N/A	N/A
10459138	Slc6a7	N/A	N/A
10459158	Hmgxb3	N/A	N/A
10459183	Slc26a2	N/A	N/A
10459208	Mir378	N/A	N/A
10459225	Mir145	N/A	N/A
10459227	Mir143	N/A	N/A
10459229	Pcyox1l	N/A	N/A
10459236	Grpel2	N/A	N/A
10459292	Gm9876	N/A	N/A
10459294	---	N/A	N/A
10459296	Fbxo38	N/A	N/A
10459375	Txnl1	N/A	N/A
10459387	Nsa2	N/A	N/A
10459391	Fech	N/A	N/A
10459405	Nars	N/A	N/A
10459421	Atp8b1	N/A	N/A
10459452	A330084C13Rik	N/A	N/A
10459467	---	N/A	N/A
10459481	Lman1	N/A	N/A
10459510	Mir694	N/A	N/A
10459512	Mc4r	N/A	N/A
10459518	Mppe1	N/A	N/A
10459552	Spire1	N/A	N/A
10459576	Cep76	N/A	N/A
10459590	Ptpn2	N/A	N/A
10459602	Ptpn2	N/A	N/A
10459604	4933403F05Rik	N/A	N/A
10459614	4930546C10Rik	N/A	N/A
10459618	---	N/A	N/A
10459620	Rab27b	N/A	N/A
10459637	---	N/A	N/A
10459643	4930503L19Rik	N/A	N/A
10459655	Poli	N/A	N/A

10459669	Rpl5	N/A	N/A
10459671	Dcc	N/A	N/A
10459730	Me2	N/A	N/A
10459747	Mapk4	N/A	N/A
10459766	Scarna17	N/A	N/A
10459770	---	N/A	N/A
10459785	Gm672	N/A	N/A
10459799	1700003O11Rik	N/A	N/A
10459802	---	N/A	N/A
10459804	Katnal2	N/A	N/A
10459823	St8sia5 /// Gm7276	N/A	N/A
10459827	8030462N17Rik /// Rnf165	N/A	N/A
10459835	---	N/A	N/A
10459837	8030462N17Rik	N/A	N/A
10459844	Haus1	N/A	N/A
10459866	Slc14a1	N/A	N/A
10459905	Setbp1	N/A	N/A
10459912	Adnp2	N/A	N/A
10459918	1110032A13Rik	N/A	N/A
10459925	Pqlc1	N/A	N/A
10459927	Kcng2	N/A	N/A
10459962	Atp9b	N/A	N/A
10460008	---	N/A	N/A
10460010	Galr1	N/A	N/A
10460016	Rpl21 /// Gm9104 /// Rpl21-ps7 /// Gm16416 /// Rpl21-ps12 /// Rpl21-ps14 /// Rpl21-ps10 /// Gm8054	N/A	N/A
10460018	Zfp236	N/A	N/A
10460055	---	N/A	N/A
10460057	Tshz1	N/A	N/A
10460070	Zfp407	N/A	N/A
10460085	Cndp2	N/A	N/A
10460097	Fam69c	N/A	N/A
10460100	---	N/A	N/A
10460102	1700034H14Rik	N/A	N/A
10460106	---	N/A	N/A
10460108	Gnpnat1	N/A	N/A
10460112	---	N/A	N/A
10460114	---	N/A	N/A
10460118	Socs6	N/A	N/A
10460123	9330132A10Rik	N/A	N/A
10460127	Dok6	N/A	N/A
10460146	---	N/A	N/A
10460149	---	N/A	N/A
10460151	Mrpl21	N/A	N/A
10460157	Cpt1a	N/A	N/A
10460194	---	N/A	N/A
10460202	Suv420h1	N/A	N/A
10460237	Unc93b1	N/A	N/A
10460253	Aldh3b2	N/A	N/A
10460255	1700055N04Rik	N/A	N/A
10460257	1700055N04Rik	N/A	N/A
10460259	Aldh3b2	N/A	N/A
10460312	Cdk2ap2	N/A	N/A
10460344	Tmem134	N/A	N/A
10460359	Coro1b	N/A	N/A
10460376	Ppp1ca	N/A	N/A
10460392	Pold4	N/A	N/A
10460400	Pcx	N/A	N/A
10460423	Spnb3	N/A	N/A
10460461	Rbm4b	N/A	N/A
10460466	Ccdc87	N/A	N/A
10460468	Ctsf	N/A	N/A
10460485	Zdhhc24	N/A	N/A
10460490	Dpp3	N/A	N/A
10460492	Mrpl11	N/A	N/A
10460498	Slc29a2	N/A	N/A
10460517	Brms1	N/A	N/A
10460541	Cd248	N/A	N/A
10460544	Yif1a	N/A	N/A
10460554	Klc2 /// Gm10817	N/A	N/A
10460556	Gal3st3	N/A	N/A
10460573	Eif1ad	N/A	N/A
10460580	Sart1 /// D330050I16Rik	N/A	N/A
10460582	AI837181	N/A	N/A
10460591	Fibp	N/A	N/A
10460603	Efemp2	N/A	N/A
10460616	Cfl1	N/A	N/A
10460623	Gm962	N/A	N/A
10460626	Rnaseh2c	N/A	N/A
10460645	Map3k11	N/A	N/A
10460693	Gm9783 /// Frmd8	N/A	N/A
10460696	Slc25a45	N/A	N/A
10460704	Gm10814	N/A	N/A
10460706	Syvn1	N/A	N/A
10460726	Fau	N/A	N/A

10460732	Znhit2-ps	N/A	N/A
10460735	BC048609 /// 1110014N23Rik	N/A	N/A
10460765	2400001E08Rik	N/A	N/A
10460767	Batf2	N/A	N/A
10460787	Atg2a	N/A	N/A
10460829	Mir194-2	N/A	N/A
10460831	Mir192	N/A	N/A
10460833	Ehd1	N/A	N/A
10460879	Men1	N/A	N/A
10460891	Map4k2	N/A	N/A
10460926	Sf1	N/A	N/A
10460947	Pygm	N/A	N/A
10460987	Nrxn2	N/A	N/A
10461012	Prdx5 /// Trmt112	N/A	N/A
10461022	Ppp1r14b	N/A	N/A
10461028	Trpt1	N/A	N/A
10461051	5730409K12Rik	N/A	N/A
10461055	---	N/A	N/A
10461078	Atl3	N/A	N/A
10461108	Iscu	N/A	N/A
10461115	Slc22a8	N/A	N/A
10461143	Chrm1	N/A	N/A
10461150	---	N/A	N/A
10461152	Snhg1	N/A	N/A
10461154	Snhg1	N/A	N/A
10461156	Snhg1	N/A	N/A
10461158	Snhg1	N/A	N/A
10461160	---	N/A	N/A
10461162	Snord22	N/A	N/A
10461164	Wdr74	N/A	N/A
10461176	Stx5a	N/A	N/A
10461191	Nxf1	N/A	N/A
10461214	Tmem223	N/A	N/A
10461220	Hnrnpul2	N/A	N/A
10461237	Bscl2	N/A	N/A
10461274	Ints5	N/A	N/A
10461277	Ganab	N/A	N/A
10461305	B3gat3	N/A	N/A
10461334	Mta2 /// Gm10353	N/A	N/A
10461354	Tut1	N/A	N/A
10461365	Eef1g	N/A	N/A
10461389	Rps7 /// Gm9493	N/A	N/A
10461391	Pcna	N/A	N/A
10461400	---	N/A	N/A
10461402	Fth1	N/A	N/A
10461408	Rab3il1	N/A	N/A
10461423	Fads3	N/A	N/A
10461439	Fads1	N/A	N/A
10461452	Fen1	N/A	N/A
10461459	Syt7	N/A	N/A
10461475	Cpsf7 /// Tmem216	N/A	N/A
10461487	Cybasc3	N/A	N/A
10461526	Vwce	N/A	N/A
10461547	Vps37c	N/A	N/A
10461568	Prpf19	N/A	N/A
10461583	Gpr44 /// Ccdc86	N/A	N/A
10461640	---	N/A	N/A
10461642	Scarna17	N/A	N/A
10461663	Mrpl16	N/A	N/A
10461671	Patl1	N/A	N/A
10461690	Osbp	N/A	N/A
10461705	Olfr1431	N/A	N/A
10461707	Olfr235	N/A	N/A
10461711	Olfr1434 /// Olfr1433	N/A	N/A
10461713	Olfr1440	N/A	N/A
10461715	Olfr1441	N/A	N/A
10461717	Pfpl	N/A	N/A
10461721	Mpeg1	N/A	N/A
10461728	Gm4952	N/A	N/A
10461735	Glyat	N/A	N/A
10461758	Keg1	N/A	N/A
10461765	Lpxn	N/A	N/A
10461780	Olfr1445	N/A	N/A
10461782	Rmnd1 /// Gm5512	N/A	N/A
10461784	Olfr1449	N/A	N/A
10461786	Olfr1450	N/A	N/A
10461788	Olfr1451	N/A	N/A
10461794	Olfr1461	N/A	N/A
10461800	Olfr1466	N/A	N/A
10461802	Olfr1467	N/A	N/A
10461804	Olfr1469	N/A	N/A
10461814	Olfr1480	N/A	N/A
10461816	Olfr1484	N/A	N/A
10461822	Olfr1490	N/A	N/A

10461824	Olfr1491	N/A	N/A
10461830	Olfr1495	N/A	N/A
10461832	Olfr1496	N/A	N/A
10461834	Olfr1502 /// Olfr1505	N/A	N/A
10461840	Olfr1505	N/A	N/A
10461842	---	N/A	N/A
10461844	Gnaq	N/A	N/A
10461867	Vps13a	N/A	N/A
10461898	Rfk	N/A	N/A
10461904	---	N/A	N/A
10461906	---	N/A	N/A
10461909	BC016495	N/A	N/A
10461921	2410127L17Rik	N/A	N/A
10461930	D030056L22Rik	N/A	N/A
10461979	Aldh1a1	N/A	N/A
10462035	Ldhb	N/A	N/A
10462039	Trpm3	N/A	N/A
10462081	Nsa2	N/A	N/A
10462084	Mir204	N/A	N/A
10462086	---	N/A	N/A
10462088	---	N/A	N/A
10462096	---	N/A	N/A
10462100	Sarnp	N/A	N/A
10462102	Ptar1	N/A	N/A
10462111	Gm9938	N/A	N/A
10462113	Apba1	N/A	N/A
10462130	Fam122a	N/A	N/A
10462136	Cycs	N/A	N/A
10462193	Ndufb4	N/A	N/A
10462195	Kank1	N/A	N/A
10462214	---	N/A	N/A
10462281	Vldlr	N/A	N/A
10462309	C030016D13Rik	N/A	N/A
10462313	Slc1a1	N/A	N/A
10462330	Ppapdc2 /// 4430402I18Rik	N/A	N/A
10462333	Cdc37l1	N/A	N/A
10462343	Gm9895	N/A	N/A
10462357	Mir101b	N/A	N/A
10462359	Gm9883	N/A	N/A
10462363	Jak2	N/A	N/A
10462390	Cd274	N/A	N/A
10462442	Il33	N/A	N/A
10462454	Uhrf2	N/A	N/A
10462473	Mbl2	N/A	N/A
10462484	A1cf	N/A	N/A
10462499	---	N/A	N/A
10462501	2700046G09Rik	N/A	N/A
10462504	Minpp1	N/A	N/A
10462507	Papss2	N/A	N/A
10462521	Pten	N/A	N/A
10462535	Pten	N/A	N/A
10462539	Nudt15	N/A	N/A
10462587	Stambpl1	N/A	N/A
10462603	Fas	N/A	N/A
10462618	Ifit3	N/A	N/A
10462621	I830012O16Rik	N/A	N/A
10462623	Ifit1	N/A	N/A
10462630	Pank1	N/A	N/A
10462683	Pcgf5	N/A	N/A
10462697	---	N/A	N/A
10462699	---	N/A	N/A
10462702	Hectd2	N/A	N/A
10462724	Tnks2	N/A	N/A
10462752	Btaf1	N/A	N/A
10462818	Hhex	N/A	N/A
10462822	Exoc6	N/A	N/A
10462853	Cyp26a1	N/A	N/A
10462861	---	N/A	N/A
10462879	---	N/A	N/A
10462912	Lgi1	N/A	N/A
10462918	Tmem20	N/A	N/A
10462957	Tbc1d12	N/A	N/A
10463037	Cyp2c39	N/A	N/A
10463043	Cyp2c37	N/A	N/A
10463051	Cyp2c50	N/A	N/A
10463064	Gm4609 /// Gm2451	N/A	N/A
10463066	Tctn3	N/A	N/A
10463068	Ptp4a1 /// Gm13363	N/A	N/A
10463070	Entpd1	N/A	N/A
10463112	Ccnj	N/A	N/A
10463121	Zfp518a	N/A	N/A
10463138	Dppa2 /// Gm9788	N/A	N/A
10463140	Lcor	N/A	N/A
10463153	Morf4l1	N/A	N/A

10463155	Gm340	N/A	N/A
10463164	Frat1	N/A	N/A
10463168	Pgam1	N/A	N/A
10463173	Zdhhc16	N/A	N/A
10463185	Ubtd1	N/A	N/A
10463200	Dhdpsl	N/A	N/A
10463209	4933411K16Rik	N/A	N/A
10463224	Marveld1	N/A	N/A
10463227	Gm6937	N/A	N/A
10463229	Zfyve27	N/A	N/A
10463242	Golga7b	N/A	N/A
10463254	D19Ertd386e	N/A	N/A
10463263	Lztfl1	N/A	N/A
10463265	Cnnm1	N/A	N/A
10463282	Entpd7	N/A	N/A
10463308	Abcc2	N/A	N/A
10463343	Gm10768	N/A	N/A
10463345	1700084K02Rik	N/A	N/A
10463355	Scd2	N/A	N/A
10463380	Hif1an	N/A	N/A
10463402	---	N/A	N/A
10463404	Fam178a	N/A	N/A
10463410	Fam178a	N/A	N/A
10463428	---	N/A	N/A
10463430	Sema4g	N/A	N/A
10463448	Peo1	N/A	N/A
10463457	Lzts2	N/A	N/A
10463486	Btrc	N/A	N/A
10463505	Dpcd	N/A	N/A
10463513	9130011E15Rik /// 4930505N22Rik	N/A	N/A
10463515	Hps6	N/A	N/A
10463535	Nolc1	N/A	N/A
10463557	Gbf1	N/A	N/A
10463632	Tmem180	N/A	N/A
10463643	---	N/A	N/A
10463645	Sufu	N/A	N/A
10463661	Trim8 /// Gm9798	N/A	N/A
10463687	D19Wsu162e	N/A	N/A
10463695	2010012O05Rik	N/A	N/A
10463704	As3mt	N/A	N/A
10463716	Cnnm2	N/A	N/A
10463729	Nt5c2	N/A	N/A
10463732	Ina	N/A	N/A
10463737	Ina	N/A	N/A
10463739	Taf5	N/A	N/A
10463799	---	N/A	N/A
10463803	Slk	N/A	N/A
10463836	Gsto1	N/A	N/A
10463875	Sorcs3	N/A	N/A
10463904	---	N/A	N/A
10463911	Add3	N/A	N/A
10463930	Mxi1	N/A	N/A
10463951	Smc3	N/A	N/A
10463997	Pdcd4	N/A	N/A
10464013	---	N/A	N/A
10464015	Shoc2	N/A	N/A
10464030	Adra2a	N/A	N/A
10464045	Acsl5	N/A	N/A
10464070	Vti1a	N/A	N/A
10464084	Tcf7l2	N/A	N/A
10464107	Ppnr	N/A	N/A
10464110	D730002M21Rik	N/A	N/A
10464113	Habp2	N/A	N/A
10464153	Nhlrc2	N/A	N/A
10464167	Adrb1	N/A	N/A
10464169	A630007B06Rik /// 1700010L13Rik	N/A	N/A
10464218	Fam160b1	N/A	N/A
10464251	Atrnl1	N/A	N/A
10464283	Gm10007	N/A	N/A
10464285	---	N/A	N/A
10464363	4930442E04Rik	N/A	N/A
10464370	Slc18a2	N/A	N/A
10464388	Rps12 /// Gm10063 /// Rps12-ps2	N/A	N/A
10464407	4933412A08Rik /// D19Ertd737e	N/A	N/A
10464409	Nanos1	N/A	N/A
10464413	Gm10751	N/A	N/A
10464415	Fam45a	N/A	N/A
10464443	---	N/A	N/A
10464445	---	N/A	N/A
10464469	Rpl7a /// Gm16477	N/A	N/A
10464471	Gal	N/A	N/A
10464479	Ppp6r3	N/A	N/A
10464504	Lrp5	N/A	N/A
10464529	Tcirg1	N/A	N/A

10464551	Ndufs8	N/A	N/A
10464569	4833408A19Rik	N/A	N/A
10464583	Gstp1	N/A	N/A
10464586	Gstp2 /// Gstp1	N/A	N/A
10464594	BC021614	N/A	N/A
10464601	---	N/A	N/A
10464603	Aip	N/A	N/A
10464642	Carns1	N/A	N/A
10464659	Rad9	N/A	N/A
10464672	Ssh3	N/A	N/A
10464688	Ankrd13d	N/A	N/A
10464704	Adrbk1	N/A	N/A
10464728	Kdm2a	N/A	N/A
10464754	Rhod	N/A	N/A
10464761	Syt12	N/A	N/A
10464772	2010003K11Rik	N/A	N/A
10464775	Lrfn4	N/A	N/A
10464783	Rce1	N/A	N/A
10464811	---	N/A	N/A
10464813	Rbm4	N/A	N/A
10464819	Rbm14	N/A	N/A
10464825	Ccs	N/A	N/A
10464858	Bbs1	N/A	N/A
10464877	Dpp3	N/A	N/A
10464896	Peli3	N/A	N/A
10464914	Tmem151a	N/A	N/A
10464917	Cnih2	N/A	N/A
10464924	Rab1b	N/A	N/A
10464932	Klc2	N/A	N/A
10464949	Pacs1	N/A	N/A
10464974	Sf3b2	N/A	N/A
10464997	---	N/A	N/A
10465005	Banf1	N/A	N/A
10465043	Gm6293	N/A	N/A
10465054	Ccdc85b /// Fosl1	N/A	N/A
10465072	Mus81	N/A	N/A
10465089	Snx32	N/A	N/A
10465103	1700020D05Rik	N/A	N/A
10465114	Kat5	N/A	N/A
10465150	Pcnxl3	N/A	N/A
10465209	Mtvr2 /// Sssca1	N/A	N/A
10465215	Sssca1 /// Mtvr2	N/A	N/A
10465220	Sssca1	N/A	N/A
10465224	Gm10815	N/A	N/A
10465226	Scyl1	N/A	N/A
10465244	Malat1	N/A	N/A
10465260	Tigd3	N/A	N/A
10465263	Dpf2	N/A	N/A
10465314	Capn1	N/A	N/A
10465336	Mrpl49	N/A	N/A
10465342	Tm7sf2	N/A	N/A
10465354	1110014N23Rik	N/A	N/A
10465366	Zfpl1	N/A	N/A
10465379	Snx15	N/A	N/A
10465388	Arl2	N/A	N/A
10465395	Ppp2r5b	N/A	N/A
10465424	Nrxn2	N/A	N/A
10465474	Prdx5 /// Trmt112	N/A	N/A
10465500	Kcnk4	N/A	N/A
10465508	Bad /// Gpr137	N/A	N/A
10465553	Fkbp2	N/A	N/A
10465580	Nudt22	N/A	N/A
10465604	Stip1	N/A	N/A
10465619	Flrt1	N/A	N/A
10465625	Otub1	N/A	N/A
10465633	Cox8a	N/A	N/A
10465638	Naa40	N/A	N/A
10465649	Mark2	N/A	N/A
10465651	Mark2	N/A	N/A
10465673	AI846148	N/A	N/A
10465683	Rab11b	N/A	N/A
10465686	Rtn3	N/A	N/A
10465740	---	N/A	N/A
10465742	C730048C13Rik /// Gm5631 /// D630002G06Rik	N/A	N/A
10465764	C730048C13Rik	N/A	N/A
10465772	Slc3a2	N/A	N/A
10465790	Tmem179b /// Taf6l	N/A	N/A
10465804	Polr2g	N/A	N/A
10465812	Ttc9c	N/A	N/A
10465820	Gng3	N/A	N/A
10465831	5730408K05Rik	N/A	N/A
10465833	Rom1	N/A	N/A
10465838	Mta2	N/A	N/A
10465853	Stxbp3a /// Stxbp3b	N/A	N/A

10465895	Fads2	N/A	N/A
10465910	---	N/A	N/A
10465916	Gm98	N/A	N/A
10465943	Dagla	N/A	N/A
10465963	Lrrc10b	N/A	N/A
10465965	4930579J09Rik	N/A	N/A
10465980	Sdhaf2	N/A	N/A
10465990	Tmem216 /// Cpsf7	N/A	N/A
10466000	Tmem138	N/A	N/A
10466008	Dak	N/A	N/A
10466073	Gm10802	N/A	N/A
10466075	Tmem132a	N/A	N/A
10466087	Tmem109	N/A	N/A
10466104	Ccdc86 /// Gpr44	N/A	N/A
10466120	Ms4a15	N/A	N/A
10466127	AW112010	N/A	N/A
10466248	Stx3	N/A	N/A
10466266	Olfr1417	N/A	N/A
10466268	Olfr1418	N/A	N/A
10466270	---	N/A	N/A
10466272	Olfr1419	N/A	N/A
10466274	---	N/A	N/A
10466276	Olfr1423	N/A	N/A
10466280	Olfr1426 /// Olfr1424 /// Olfr1425	N/A	N/A
10466286	Olfr1427	N/A	N/A
10466288	Olfr1428	N/A	N/A
10466290	Olfr76	N/A	N/A
10466292	---	N/A	N/A
10466294	---	N/A	N/A
10466296	Olfr262	N/A	N/A
10466300	Olfr1436 /// Olfr1437	N/A	N/A
10466302	---	N/A	N/A
10466314	---	N/A	N/A
10466317	Zfp91 /// U05342 /// Cntf	N/A	N/A
10466344	---	N/A	N/A
10466347	Olfr1457	N/A	N/A
10466351	Olfr1459	N/A	N/A
10466353	---	N/A	N/A
10466355	Olfr1465	N/A	N/A
10466361	Olfr1497	N/A	N/A
10466366	Olfr1499	N/A	N/A
10466372	Olfr1504	N/A	N/A
10466374	Tle4	N/A	N/A
10466402	Eif4a1	N/A	N/A
10466404	---	N/A	N/A
10466406	---	N/A	N/A
10466439	Pebp1	N/A	N/A
10466441	Vps13a	N/A	N/A
10466528	Rfk	N/A	N/A
10466571	---	N/A	N/A
10466573	Ostf1	N/A	N/A
10466587	Rorb	N/A	N/A
10466604	---	N/A	N/A
10466620	---	N/A	N/A
10466622	C730037M02Rik	N/A	N/A
10466624	Aldh1a7	N/A	N/A
10466659	Gda	N/A	N/A
10466676	1110059E24Rik	N/A	N/A
10466680	Klf9 /// Gm9971	N/A	N/A
10466728	1700028P14Rik	N/A	N/A
10466735	Fam189a2	N/A	N/A
10466779	Pip5k1b	N/A	N/A
10466798	---	N/A	N/A
10466818	Cbwd1	N/A	N/A
10466835	Snora19	N/A	N/A
10466837	---	N/A	N/A
10466839	---	N/A	N/A
10466843	Gapdh /// Gm10293	N/A	N/A
10466846	---	N/A	N/A
10466848	D19Bwg1357e	N/A	N/A
10466865	Rfx3	N/A	N/A
10466886	Glis3	N/A	N/A
10466888	Glis3	N/A	N/A
10466920	Slc1a1	N/A	N/A
10466923	Rpl26 /// Gm10136 /// Gm15772	N/A	N/A
10466925	Ak3	N/A	N/A
10466935	Rln1	N/A	N/A
10466947	Ermp1	N/A	N/A
10466970	9930021J03Rik	N/A	N/A
10466972	Ranbp6	N/A	N/A
10466976	Gldc	N/A	N/A
10467001	---	N/A	N/A
10467003	Ppp1r2 /// Gm5972	N/A	N/A
10467013	Prkg1	N/A	N/A

10467038	Gm6642	N/A	N/A
10467041	Asah2	N/A	N/A
10467068	Sgms1	N/A	N/A
10467088	Rpl9 /// Gm5451	N/A	N/A
10467102	Rnls	N/A	N/A
10467110	Lipo1	N/A	N/A
10467134	---	N/A	N/A
10467139	Lipa	N/A	N/A
10467153	Slc16a12	N/A	N/A
10467162	Pank1	N/A	N/A
10467173	Mir107	N/A	N/A
10467175	A830019P07Rik	N/A	N/A
10467203	Pcgf5	N/A	N/A
10467216	Cpeb3	N/A	N/A
10467230	Ide	N/A	N/A
10467256	Rpl10	N/A	N/A
10467319	Rbp4	N/A	N/A
10467342	Gm9886	N/A	N/A
10467344	Noc3l	N/A	N/A
10467368	Ctdspl2	N/A	N/A
10467372	Cyp2c38	N/A	N/A
10467380	Cyp2c67	N/A	N/A
10467385	Cyp2c68	N/A	N/A
10467390	Cyp2c40 /// Cyp2c67 /// Cyp2c68 /// Cyp2c69	N/A	N/A
10467400	Cyp2c54	N/A	N/A
10467410	Cyp2c70	N/A	N/A
10467420	Pdlim1	N/A	N/A
10467468	3010085J16Rik	N/A	N/A
10467470	Aldh18a1	N/A	N/A
10467489	Ptp4a1 /// Gm13363	N/A	N/A
10467493	Tctn3	N/A	N/A
10467529	Opalin	N/A	N/A
10467560	Tm9sf3	N/A	N/A
10467578	Pik3ap1	N/A	N/A
10467599	Slit1	N/A	N/A
10467650	Frat2	N/A	N/A
10467697	Mms19	N/A	N/A
10467730	Morn4	N/A	N/A
10467739	Avpi1	N/A	N/A
10467744	Sfrp5	N/A	N/A
10467838	---	N/A	N/A
10467840	C130021O09Rik	N/A	N/A
10467842	Got1	N/A	N/A
10467852	Slc25a28	N/A	N/A
10467859	Cox15	N/A	N/A
10467871	Dnmbp	N/A	N/A
10467887	Cpn1	N/A	N/A
10467897	Cyp2c44	N/A	N/A
10467907	Erlin1	N/A	N/A
10467921	Chuk	N/A	N/A
10467941	Cwf19l1	N/A	N/A
10467956	Bloc1s2	N/A	N/A
10467979	Scd1	N/A	N/A
10468016	Ndufb8	N/A	N/A
10468022	Mrpl43 /// Sema4g	N/A	N/A
10468030	Pdzd7	N/A	N/A
10468037	Pdzd7	N/A	N/A
10468046	Lbx1	N/A	N/A
10468059	Fbxw4	N/A	N/A
10468081	Npm3 /// Npm3-ps1	N/A	N/A
10468089	Mgea5 /// E330018D03Rik	N/A	N/A
10468131	9130011E15Rik	N/A	N/A
10468159	Ldb1	N/A	N/A
10468180	Psd	N/A	N/A
10468200	Cuedc2 /// Fbxl15	N/A	N/A
10468213	2310034G01Rik	N/A	N/A
10468217	Actr1a	N/A	N/A
10468229	Trim8 /// Gm9798	N/A	N/A
10468231	Arl3	N/A	N/A
10468239	Cyp17a1	N/A	N/A
10468249	Cnnm2 /// Gm10199	N/A	N/A
10468251	---	N/A	N/A
10468253	Nt5c2	N/A	N/A
10468275	Pcgf6	N/A	N/A
10468287	Usmg5	N/A	N/A
10468292	Pdcd11	N/A	N/A
10468307	---	N/A	N/A
10468311	Sh3pxd2a	N/A	N/A
10468329	Obfc1	N/A	N/A
10468413	D19Ertd652e	N/A	N/A
10468415	D19Ertd652e	N/A	N/A
10468417	D19Ertd652e	N/A	N/A
10468419	D19Ertd652e	N/A	N/A
10468448	---	N/A	N/A

10468450	Rpl13a-ps1	N/A	N/A
10468452	Sorcs1	N/A	N/A
10468485	---	N/A	N/A
10468487	LOC280487	N/A	N/A
10468489	Xpnpep1	N/A	N/A
10468517	Mxi1	N/A	N/A
10468525	5830416P10Rik	N/A	N/A
10468527	5830416P10Rik	N/A	N/A
10468531	Nutf2 /// Nutf2-ps1	N/A	N/A
10468533	Gpam	N/A	N/A
10468560	Gm10755	N/A	N/A
10468584	Zdhhc6	N/A	N/A
10468639	Dclre1a	N/A	N/A
10468651	---	N/A	N/A
10468653	A630007B06Rik	N/A	N/A
10468668	Afap1l2	N/A	N/A
10468691	Ablim1	N/A	N/A
10468722	Gfra1	N/A	N/A
10468746	Hspa12a	N/A	N/A
10468762	4930506M07Rik	N/A	N/A
10468783	Vax1	N/A	N/A
10468789	Pdzd8	N/A	N/A
10468795	Rab11fip2	N/A	N/A
10468802	D19Ertd737e	N/A	N/A
10468810	Prlhr	N/A	N/A
10468816	2700078E11Rik	N/A	N/A
10468828	Eif3a	N/A	N/A
10468851	Snora19	N/A	N/A
10468853	Sfxn4	N/A	N/A
10468869	Prdx3	N/A	N/A
10468877	---	N/A	N/A
10468881	Zfp826	N/A	N/A
10468885	Zfp826 /// BC029127	N/A	N/A
10468891	Gm7102	N/A	N/A
10468893	Csf2ra	N/A	N/A
10468909	Disp1	N/A	N/A
10468929	Nmt2	N/A	N/A
10468974	Cdnf	N/A	N/A
10468988	---	N/A	N/A
10468990	Parl	N/A	N/A
10468992	Frmd4a	N/A	N/A
10469018	Gm10862	N/A	N/A
10469035	Sephs1	N/A	N/A
10469046	Phyh	N/A	N/A
10469056	Gm13194	N/A	N/A
10469058	Ucma	N/A	N/A
10469066	Ccdc3	N/A	N/A
10469070	Nudt5 /// Cdc123 /// Gm13199	N/A	N/A
10469081	Gm10859	N/A	N/A
10469083	Upf2	N/A	N/A
10469108	Gm10857	N/A	N/A
10469110	Usp6nl	N/A	N/A
10469127	LOC280487	N/A	N/A
10469138	4930412O13Rik	N/A	N/A
10469140	---	N/A	N/A
10469143	Taf3	N/A	N/A
10469145	Kin	N/A	N/A
10469151	Itih5	N/A	N/A
10469167	Sfmbt2	N/A	N/A
10469195	Mir669b	N/A	N/A
10469199	Mir467b /// Mir467d	N/A	N/A
10469203	Mir467b	N/A	N/A
10469207	Mir467b	N/A	N/A
10469213	Mir467b	N/A	N/A
10469217	Mir467b	N/A	N/A
10469221	Mir467b /// Mir467d	N/A	N/A
10469225	Mir467b	N/A	N/A
10469227	Mir467b	N/A	N/A
10469231	Mir467b	N/A	N/A
10469237	Mir466	N/A	N/A
10469239	Mir669c	N/A	N/A
10469243	Mir297b	N/A	N/A
10469247	Gm10852	N/A	N/A
10469276	Gm10851	N/A	N/A
10469289	Il15ra	N/A	N/A
10469300	Ankrd16	N/A	N/A
10469320	Gm9875	N/A	N/A
10469322	Vim	N/A	N/A
10469335	Stam	N/A	N/A
10469389	Slc39a12	N/A	N/A
10469404	Cacnb2	N/A	N/A
10469425	Arl5b	N/A	N/A
10469457	Plxdc2	N/A	N/A
10469503	---	N/A	N/A

10469505	Commd3	N/A	N/A
10469514	Bmi1	N/A	N/A
10469536	---	N/A	N/A
10469538	4930426L09Rik	N/A	N/A
10469559	Msrb2	N/A	N/A
10469571	Otud1	N/A	N/A
10469575	Ptp4a1 /// Gm13363	N/A	N/A
10469577	Gm16495	N/A	N/A
10469579	---	N/A	N/A
10469611	C130046B21Rik	N/A	N/A
10469613	Thnsl1	N/A	N/A
10469622	Gpr158	N/A	N/A
10469635	Gm10846	N/A	N/A
10469672	Gad2	N/A	N/A
10469693	---	N/A	N/A
10469720	Acbd5	N/A	N/A
10469732	Yme1l1	N/A	N/A
10469772	Ube2d3	N/A	N/A
10469849	Zmynd19	N/A	N/A
10469856	Wdr85 /// Mrpl41	N/A	N/A
10469904	Gm6813 /// Gm9104 /// Rpl21-ps4 /// Rpl21-ps7 /// Gm16415 /// Gm16416 /// Rpl21-ps12 /// Rpl21-ps14 /// Rpl21-ps10 ///	N/A	N/A
10469906	Nelf	N/A	N/A
10469923	Entpd8	N/A	N/A
10469941	---	N/A	N/A
10469951	Rnf208	N/A	N/A
10469957	Tprn	N/A	N/A
10469965	Anapc2	N/A	N/A
10469979	Tmem210	N/A	N/A
10469984	Lrrc26	N/A	N/A
10469987	Man1b1	N/A	N/A
10470027	Npdc1	N/A	N/A
10470050	Abca2	N/A	N/A
10470109	Fbxw5	N/A	N/A
10470119	Edf1	N/A	N/A
10470182	Bmyc	N/A	N/A
10470214	Kcnt1	N/A	N/A
10470248	Gpsm1	N/A	N/A
10470268	Pmpca	N/A	N/A
10470283	Egfl7	N/A	N/A
10470298	Fam69b	N/A	N/A
10470305	Rpl7a	N/A	N/A
10470314	---	N/A	N/A
10470316	---	N/A	N/A
10470318	---	N/A	N/A
10470320	---	N/A	N/A
10470322	Surf2	N/A	N/A
10470381	5930434B04Rik	N/A	N/A
10470388	5930434B04Rik	N/A	N/A
10470390	---	N/A	N/A
10470427	Wdr5	N/A	N/A
10470444	---	N/A	N/A
10470446	Rxra	N/A	N/A
10470529	Olfm1	N/A	N/A
10470543	Gm347	N/A	N/A
10470555	Gbgt1	N/A	N/A
10470562	Gm10134	N/A	N/A
10470564	Ralgds	N/A	N/A
10470584	Tsc1	N/A	N/A
10470647	---	N/A	N/A
10470665	Setx	N/A	N/A
10470696	Med27	N/A	N/A
10470741	Coq4 /// Trub2	N/A	N/A
10470751	Slc27a4	N/A	N/A
10470768	Urm1	N/A	N/A
10470775	Cercam	N/A	N/A
10470788	Odf2	N/A	N/A
10470816	Gle1	N/A	N/A
10470834	Spna2	N/A	N/A
10470893	BC085271 /// Set	N/A	N/A
10470905	Set	N/A	N/A
10470907	Set	N/A	N/A
10470909	---	N/A	N/A
10470911	---	N/A	N/A
10470936	Tbc1d13	N/A	N/A
10470948	Slc39a1	N/A	N/A
10470950	Endog	N/A	N/A
10470954	Lrrc8a	N/A	N/A
10470959	Phyhd1	N/A	N/A
10471018	Fam73b	N/A	N/A
10471036	Dolpp1	N/A	N/A
10471045	Ppp2r4	N/A	N/A
10471058	Cstad	N/A	N/A
10471062	Mettl11a	N/A	N/A
10471080	Usp20	N/A	N/A

10471108	Gpr107	N/A	N/A
10471129	Ncs1	N/A	N/A
10471154	Ass1 /// Gm5424	N/A	N/A
10471171	Fubp3	N/A	N/A
10471191	Exosc2	N/A	N/A
10471201	Abl1	N/A	N/A
10471247	Aif1l	N/A	N/A
10471256	Nup214	N/A	N/A
10471294	Ppapdc3	N/A	N/A
10471298	Bat2l	N/A	N/A
10471333	---	N/A	N/A
10471335	---	N/A	N/A
10471337	Pomt1	N/A	N/A
10471358	Rpl36 /// Gm13611 /// Gm5745	N/A	N/A
10471385	Mir199b	N/A	N/A
10471387	Ciz1	N/A	N/A
10471411	Ptges2	N/A	N/A
10471419	Naif1	N/A	N/A
10471438	Dpm2	N/A	N/A
10471474	Ak1	N/A	N/A
10471486	Eng	N/A	N/A
10471503	Taf1d	N/A	N/A
10471519	Tor2a	N/A	N/A
10471533	---	N/A	N/A
10471535	Fam129b	N/A	N/A
10471555	Angptl2	N/A	N/A
10471567	C130021I20Rik /// LOC100504399	N/A	N/A
10471569	---	N/A	N/A
10471571	Mapkap1	N/A	N/A
10471586	Hspa5	N/A	N/A
10471597	Gm6422	N/A	N/A
10471655	Gsn	N/A	N/A
10471675	Glo1	N/A	N/A
10471677	Dab2ip	N/A	N/A
10471701	Gm10829	N/A	N/A
10471715	Mrrf	N/A	N/A
10471739	Olfr338	N/A	N/A
10471743	Olfr340	N/A	N/A
10471745	Olfr342	N/A	N/A
10471747	Olfr344	N/A	N/A
10471753	Olfr347	N/A	N/A
10471759	Olfr350	N/A	N/A
10471761	Olfr352	N/A	N/A
10471763	Olfr354	N/A	N/A
10471766	---	N/A	N/A
10471768	Olfr356	N/A	N/A
10471770	Olfr357	N/A	N/A
10471772	Olfr360	N/A	N/A
10471774	Olfr365	N/A	N/A
10471778	Olfr366	N/A	N/A
10471780	Olfr367-ps	N/A	N/A
10471782	Olfr368	N/A	N/A
10471784	Rabgap1	N/A	N/A
10471814	Gpr21	N/A	N/A
10471842	---	N/A	N/A
10471844	Nek6	N/A	N/A
10471878	Mir181a-2	N/A	N/A
10471880	Mir181b-2	N/A	N/A
10471904	Arpc5l	N/A	N/A
10471909	---	N/A	N/A
10471945	Zeb2	N/A	N/A
10471949	---	N/A	N/A
10471951	---	N/A	N/A
10471953	Acvr2a	N/A	N/A
10471967	Mbd5	N/A	N/A
10471978	Epc2	N/A	N/A
10471994	Kif5c	N/A	N/A
10472034	Lypd6	N/A	N/A
10472040	Gm13498	N/A	N/A
10472042	---	N/A	N/A
10472044	---	N/A	N/A
10472050	Tnfaip6	N/A	N/A
10472058	Rif1	N/A	N/A
10472095	Bloc1s2	N/A	N/A
10472097	Fmnl2	N/A	N/A
10472128	Arl6ip6	N/A	N/A
10472134	---	N/A	N/A
10472153	---	N/A	N/A
10472155	Kcnj3	N/A	N/A
10472162	Gpd2	N/A	N/A
10472195	Nudt15	N/A	N/A
10472197	---	N/A	N/A
10472212	Pkp4	N/A	N/A
10472275	---	N/A	N/A

10472277	March7	N/A	N/A
10472289	Tank	N/A	N/A
10472300	Psmd14	N/A	N/A
10472321	Slc4a10	N/A	N/A
10472350	Gca	N/A	N/A
10472364	Scn2a1	N/A	N/A
10472366	Scn2a1	N/A	N/A
10472368	Scn2a1	N/A	N/A
10472370	Scn2a1	N/A	N/A
10472372	Scn9a /// Scn2a1	N/A	N/A
10472374	Scn9a /// Scn3a /// Scn2a1	N/A	N/A
10472376	Scn2a1	N/A	N/A
10472378	Scn2a1	N/A	N/A
10472380	Scn2a1	N/A	N/A
10472382	Scn2a1	N/A	N/A
10472384	Scn2a1	N/A	N/A
10472386	Scn2a1	N/A	N/A
10472396	Scn2a1	N/A	N/A
10472398	Scn2a1	N/A	N/A
10472400	Scn2a1	N/A	N/A
10472402	Scn2a1	N/A	N/A
10472408	Csrnp3	N/A	N/A
10472418	Scn9a	N/A	N/A
10472436	B3galt1	N/A	N/A
10472440	Tax1bp3	N/A	N/A
10472467	---	N/A	N/A
10472499	---	N/A	N/A
10472501	Lass6	N/A	N/A
10472514	Nostrin	N/A	N/A
10472549	Bbs5	N/A	N/A
10472570	Ppig	N/A	N/A
10472605	Ssb	N/A	N/A
10472621	Ubr3	N/A	N/A
10472630	Ubr3	N/A	N/A
10472686	Rpl9 /// Gm5451	N/A	N/A
10472707	Gad1	N/A	N/A
10472724	Gorasp2	N/A	N/A
10472757	Cybrd1	N/A	N/A
10472764	Dync1i2	N/A	N/A
10472782	Hat1	N/A	N/A
10472846	Pdk1	N/A	N/A
10472860	Rapgef4	N/A	N/A
10472923	Ak4	N/A	N/A
10472925	---	N/A	N/A
10472930	Sp9	N/A	N/A
10472933	Scrn3	N/A	N/A
10472942	Gm10822	N/A	N/A
10472944	---	N/A	N/A
10472946	Hoxd13	N/A	N/A
10472958	Hoxd10	N/A	N/A
10472994	Mtx2	N/A	N/A
10473006	Rpl21 /// Gm6813 /// Gm9104 /// Rpl21-ps4 /// Rpl21-ps7 /// Gm16416 /// Rpl21-ps12 /// Rpl21-ps10 /// Gm8054	N/A	N/A
10473008	Hnrnpa3	N/A	N/A
10473022	Plp2	N/A	N/A
10473024	Agps	N/A	N/A
10473058	Osbpl6	N/A	N/A
10473097	Plekha3	N/A	N/A
10473107	---	N/A	N/A
10473118	Ube2e3	N/A	N/A
10473158	---	N/A	N/A
10473160	Ssfa2	N/A	N/A
10473190	Dnajc10	N/A	N/A
10473217	---	N/A	N/A
10473219	---	N/A	N/A
10473224	Dusp19	N/A	N/A
10473230	Nup35	N/A	N/A
10473240	Eno1 /// Gm5506	N/A	N/A
10473244	Zfp804a	N/A	N/A
10473250	Mrpl18	N/A	N/A
10473256	Fsip2	N/A	N/A
10473270	---	N/A	N/A
10473272	Zc3h15	N/A	N/A
10473281	Itgav	N/A	N/A
10473312	Fam171b	N/A	N/A
10473322	Cwc22	N/A	N/A
10473325	Cwc22	N/A	N/A
10473328	Cwc22	N/A	N/A
10473331	Cwc22	N/A	N/A
10473334	Cwc22	N/A	N/A
10473337	Cwc22	N/A	N/A
10473340	Cwc22	N/A	N/A
10473343	Med19 /// Zdhhc5	N/A	N/A
10473349	Ypel4	N/A	N/A
10473356	Ube2l6	N/A	N/A

10473363	Timm10	N/A	N/A
10473384	Slc43a3	N/A	N/A
10473414	Ssrp1	N/A	N/A
10473444	Aplnr	N/A	N/A
10473446	Olfr996	N/A	N/A
10473450	Olfr998	N/A	N/A
10473452	---	N/A	N/A
10473454	Olfr1008	N/A	N/A
10473456	Olfr1009	N/A	N/A
10473458	Olfr1010	N/A	N/A
10473462	Olfr1014	N/A	N/A
10473464	Olfr1015	N/A	N/A
10473469	Olfr1020	N/A	N/A
10473471	---	N/A	N/A
10473473	Olfr1022	N/A	N/A
10473479	Olfr1026	N/A	N/A
10473481	Olfr1028	N/A	N/A
10473483	Olfr1029	N/A	N/A
10473485	Olfr1030	N/A	N/A
10473489	Olfr1032	N/A	N/A
10473491	Olfr1033	N/A	N/A
10473494	Olfr1036 /// Olfr1034	N/A	N/A
10473496	Olfr1036	N/A	N/A
10473505	Olfr1076	N/A	N/A
10473507	Olfr1093	N/A	N/A
10473511	Olfr1102	N/A	N/A
10473513	Olfr1112	N/A	N/A
10473515	Olfr1113	N/A	N/A
10473517	---	N/A	N/A
10473519	Olfr1115	N/A	N/A
10473523	Olfr1118-ps /// Olfr1116-ps	N/A	N/A
10473525	Olfr1118-ps	N/A	N/A
10473528	Olfr1120	N/A	N/A
10473537	Olfr1123	N/A	N/A
10473539	Olfr1124	N/A	N/A
10473541	Olfr1126	N/A	N/A
10473547	Srp9	N/A	N/A
10473551	Olfr153	N/A	N/A
10473553	Olfr1126 /// Olfr1129 /// Olfr1130	N/A	N/A
10473557	Olfr1131	N/A	N/A
10473562	Olfr152	N/A	N/A
10473564	Olfr1143	N/A	N/A
10473566	Olfr1145	N/A	N/A
10473568	---	N/A	N/A
10473570	Olfr1148	N/A	N/A
10473572	---	N/A	N/A
10473574	Olfr1151	N/A	N/A
10473578	Olfr1153	N/A	N/A
10473580	Olfr1158	N/A	N/A
10473584	---	N/A	N/A
10473586	Olfr1168	N/A	N/A
10473592	Olfr1183	N/A	N/A
10473594	Olfr1184	N/A	N/A
10473596	---	N/A	N/A
10473600	Olfr1188	N/A	N/A
10473602	Olfr1189	N/A	N/A
10473606	---	N/A	N/A
10473608	Olfr1193	N/A	N/A
10473610	Olfr1206 /// Olfr1201 /// Olfr1203	N/A	N/A
10473612	Olfr1202	N/A	N/A
10473614	Olfr1203	N/A	N/A
10473616	Olfr1204	N/A	N/A
10473622	Olfr1255	N/A	N/A
10473630	Olfr1260	N/A	N/A
10473639	Olfr1263	N/A	N/A
10473641	Olfr1265	N/A	N/A
10473648	Olfr1274-ps	N/A	N/A
10473650	Nup160	N/A	N/A
10473690	Fnbp4	N/A	N/A
10473737	Mtch2	N/A	N/A
10473760	Celf1	N/A	N/A
10473793	Psmc3	N/A	N/A
10473809	Sfpi1	N/A	N/A
10473847	Acp2 /// Ddb2	N/A	N/A
10473874	Arfgap2 /// 1110051M20Rik	N/A	N/A
10473919	Ckap5	N/A	N/A
10473965	Arhgap1	N/A	N/A
10473976	Harbi1	N/A	N/A
10473981	Ambra1	N/A	N/A
10474002	Chrm4	N/A	N/A
10474004	Dgkz /// Gm9821	N/A	N/A
10474006	Phf21a	N/A	N/A
10474028	Pex16	N/A	N/A
10474041	1700029I15Rik	N/A	N/A

10474045	Chst1	N/A	N/A
10474048	Syt13	N/A	N/A
10474064	Trp53i11	N/A	N/A
10474073	---	N/A	N/A
10474077	---	N/A	N/A
10474079	Gm10803	N/A	N/A
10474093	2810002D19Rik /// Ttc17	N/A	N/A
10474096	Lrrc4c	N/A	N/A
10474102	---	N/A	N/A
10474112	Traf6	N/A	N/A
10474123	Commd9	N/A	N/A
10474129	Pamr1	N/A	N/A
10474141	Slc1a2	N/A	N/A
10474169	---	N/A	N/A
10474181	Abtb2	N/A	N/A
10474199	---	N/A	N/A
10474201	Lmo2	N/A	N/A
10474207	Fbxo3	N/A	N/A
10474221	Gm10912	N/A	N/A
10474223	Cd59b	N/A	N/A
10474229	Cd59a /// Cd59b	N/A	N/A
10474234	A930018P22Rik	N/A	N/A
10474239	Gapdh	N/A	N/A
10474241	---	N/A	N/A
10474269	Gm6306	N/A	N/A
10474307	0610012H03Rik	N/A	N/A
10474333	Elp4	N/A	N/A
10474339	Gm10795	N/A	N/A
10474355	Dcdc5	N/A	N/A
10474369	C130023O10Rik	N/A	N/A
10474371	Gm16488	N/A	N/A
10474373	Kcna4	N/A	N/A
10474379	Hadhb	N/A	N/A
10474411	Lin7c	N/A	N/A
10474419	Lgr4	N/A	N/A
10474477	---	N/A	N/A
10474479	Olfr1276	N/A	N/A
10474481	Olfr1278	N/A	N/A
10474483	Olfr1279	N/A	N/A
10474492	Olfr1284	N/A	N/A
10474497	Olfr1280	N/A	N/A
10474499	Olfr1288	N/A	N/A
10474504	Olfr1289	N/A	N/A
10474514	Olfr1301	N/A	N/A
10474518	---	N/A	N/A
10474520	Tdh	N/A	N/A
10474524	Olfr1318 /// Olfr1317	N/A	N/A
10474526	Lpcat4	N/A	N/A
10474541	Nop10	N/A	N/A
10474545	Slc12a6	N/A	N/A
10474573	Slc12a6	N/A	N/A
10474575	Slc12a6	N/A	N/A
10474619	Fmn1	N/A	N/A
10474642	BC052040	N/A	N/A
10474669	Rpl27a /// Gm5908 /// Gm6378	N/A	N/A
10474671	Spred1	N/A	N/A
10474683	Mir674	N/A	N/A
10474685	---	N/A	N/A
10474687	---	N/A	N/A
10474689	Fam98b	N/A	N/A
10474725	Eif2ak4	N/A	N/A
10474793	Pak6	N/A	N/A
10474807	5430417L22Rik	N/A	N/A
10474814	Disp2	N/A	N/A
10474836	Ivd	N/A	N/A
10474867	---	N/A	N/A
10474870	Rpusd2	N/A	N/A
10474915	Gchfr	N/A	N/A
10474919	Gm14137	N/A	N/A
10474950	Vps18	N/A	N/A
10474977	1500003O03Rik	N/A	N/A
10474998	---	N/A	N/A
10475000	Rtf1	N/A	N/A
10475027	Tyro3	N/A	N/A
10475051	Mga	N/A	N/A
10475080	Mapkbp1	N/A	N/A
10475199	Snap23	N/A	N/A
10475211	Haus2	N/A	N/A
10475218	Stard9	N/A	N/A
10475226	Stard9	N/A	N/A
10475245	Ubr1	N/A	N/A
10475247	Tmem62	N/A	N/A
10475262	---	N/A	N/A
10475264	Ccndbp1	N/A	N/A

10475280	Adal	N/A	N/A
10475293	Tubgcp4	N/A	N/A
10475314	Mtap1a	N/A	N/A
10475324	Ckmt1	N/A	N/A
10475335	Pdia3	N/A	N/A
10475350	Serinc4 /// Serf2 /// 2310003F16Rik	N/A	N/A
10475360	Mrpl51	N/A	N/A
10475378	Casc4	N/A	N/A
10475394	Ctdspl2	N/A	N/A
10475405	Eif3j	N/A	N/A
10475414	B2m	N/A	N/A
10475435	Rps12 /// Gm10063 /// Rps12-ps2	N/A	N/A
10475437	Sord	N/A	N/A
10475514	---	N/A	N/A
10475525	Pldn	N/A	N/A
10475532	Sqrdl	N/A	N/A
10475544	Sema6d	N/A	N/A
10475578	Ctxn2	N/A	N/A
10475623	Fbn1 /// Gm9913	N/A	N/A
10475625	Eid1	N/A	N/A
10475630	Galk2	N/A	N/A
10475648	Dtwd1	N/A	N/A
10475653	Slc27a2	N/A	N/A
10475665	Usp8	N/A	N/A
10475708	Blvra	N/A	N/A
10475718	Snrnp200 /// Ciao1	N/A	N/A
10475767	Tmem127	N/A	N/A
10475772	Stard7	N/A	N/A
10475800	Adra2b	N/A	N/A
10475830	Mrps5	N/A	N/A
10475843	Gm10765	N/A	N/A
10475866	Bcl2l11	N/A	N/A
10475890	Mertk	N/A	N/A
10475910	---	N/A	N/A
10475912	Tmem87b	N/A	N/A
10475941	Zc3h6	N/A	N/A
10475957	Ttl	N/A	N/A
10475965	Polr1b	N/A	N/A
10475981	Chchd5	N/A	N/A
10475987	Setd3	N/A	N/A
10475990	Slc20a1	N/A	N/A
10476021	Sirpa	N/A	N/A
10476033	Stk35	N/A	N/A
10476093	Nop56	N/A	N/A
10476102	---	N/A	N/A
10476104	---	N/A	N/A
10476106	Snord57	N/A	N/A
10476130	1700020A23Rik	N/A	N/A
10476133	4933425O20Rik	N/A	N/A
10476136	Vps16	N/A	N/A
10476163	Ptpra	N/A	N/A
10476189	Mrps26	N/A	N/A
10476192	Oxt	N/A	N/A
10476197	Itpa	N/A	N/A
10476207	Atrn	N/A	N/A
10476270	2310035K24Rik	N/A	N/A
10476276	Mavs	N/A	N/A
10476297	Mir103-2	N/A	N/A
10476299	---	N/A	N/A
10476312	4930425F17Rik	N/A	N/A
10476314	Prnp /// Prnd	N/A	N/A
10476319	Prnd	N/A	N/A
10476326	Cds2	N/A	N/A
10476347	AU019990	N/A	N/A
10476349	1110034G24Rik	N/A	N/A
10476353	---	N/A	N/A
10476355	Chgb	N/A	N/A
10476383	Crls1	N/A	N/A
10476395	Bmp2	N/A	N/A
10476399	LOC280487	N/A	N/A
10476401	Plcb1	N/A	N/A
10476441	Rpl29 /// Gm3550 /// Rpl29-ps2	N/A	N/A
10476443	Plcb4	N/A	N/A
10476482	6330527O06Rik	N/A	N/A
10476497	Ankrd5	N/A	N/A
10476512	Snap25	N/A	N/A
10476526	2210009G21Rik	N/A	N/A
10476538	Btbd3	N/A	N/A
10476560	Ism1	N/A	N/A
10476582	Macrod2	N/A	N/A
10476588	Macrod2	N/A	N/A
10476590	Macrod2	N/A	N/A
10476592	Macrod2	N/A	N/A
10476594	Macrod2	N/A	N/A

10476612	---	N/A	N/A
10476614	---	N/A	N/A
10476633	Pcsk2	N/A	N/A
10476648	Dstn	N/A	N/A
10476658	---	N/A	N/A
10476668	Csrp2bp /// Pet117	N/A	N/A
10476680	Zfp133 /// LOC668917	N/A	N/A
10476689	LOC668917	N/A	N/A
10476691	Polr3f	N/A	N/A
10476702	Sec23b	N/A	N/A
10476725	Gm561	N/A	N/A
10476728	Dtd1	N/A	N/A
10476735	---	N/A	N/A
10476740	Slc24a3	N/A	N/A
10476775	Naa20	N/A	N/A
10476782	4930529M08Rik	N/A	N/A
10476791	4930529M08Rik	N/A	N/A
10476793	4930529M08Rik	N/A	N/A
10476795	4930529M08Rik	N/A	N/A
10476817	Atpaf1	N/A	N/A
10476819	Plk1s1	N/A	N/A
10476834	Xrn2	N/A	N/A
10476868	---	N/A	N/A
10476872	---	N/A	N/A
10476880	---	N/A	N/A
10476882	---	N/A	N/A
10476886	Sstr4	N/A	N/A
10476889	Nxt1	N/A	N/A
10476893	Gzf1	N/A	N/A
10476931	Cst10	N/A	N/A
10476935	Tmem90b	N/A	N/A
10476939	Gm4979 /// 3300002I08Rik /// Zfp442	N/A	N/A
10476941	---	N/A	N/A
10476952	Entpd6	N/A	N/A
10476969	Pygb	N/A	N/A
10476998	---	N/A	N/A
10477001	---	N/A	N/A
10477004	---	N/A	N/A
10477006	Nsfl1c	N/A	N/A
10477012	Fkbp1a	N/A	N/A
10477058	Scrt2	N/A	N/A
10477073	Csnk2a1	N/A	N/A
10477090	Tbc1d20	N/A	N/A
10477100	---	N/A	N/A
10477103	Nrsn2 /// Gm14164	N/A	N/A
10477120	---	N/A	N/A
10477122	Defb20	N/A	N/A
10477129	Defb21	N/A	N/A
10477147	H13	N/A	N/A
10477167	Mcts2	N/A	N/A
10477176	---	N/A	N/A
10477233	Xkr7	N/A	N/A
10477264	Tm9sf4	N/A	N/A
10477286	Pofut1	N/A	N/A
10477311	Asxl1	N/A	N/A
10477353	Mapre1	N/A	N/A
10477370	Tomm20	N/A	N/A
10477543	Cbfa2t2	N/A	N/A
10477555	1700007I08Rik	N/A	N/A
10477557	Zfp341	N/A	N/A
10477572	Chmp4b	N/A	N/A
10477583	a /// Raly	N/A	N/A
10477604	Itch	N/A	N/A
10477630	Dynlrb1	N/A	N/A
10477637	Map1lc3a	N/A	N/A
10477642	Mir695	N/A	N/A
10477644	Trp53inp2	N/A	N/A
10477649	Acss2	N/A	N/A
10477715	Mir499	N/A	N/A
10477777	Ergic3	N/A	N/A
10477808	Romo1	N/A	N/A
10477813	Rbm39	N/A	N/A
10477815	Phf20	N/A	N/A
10477852	---	N/A	N/A
10477854	Epb4.1l1	N/A	N/A
10477894	---	N/A	N/A
10477929	1110008F13Rik /// Sla2	N/A	N/A
10477942	Rbl1	N/A	N/A
10477944	---	N/A	N/A
10477946	Rpn2	N/A	N/A
10477966	Manbal	N/A	N/A
10477970	Src	N/A	N/A
10477986	Nnat	N/A	N/A
10477991	---	N/A	N/A

10477994	Ctnnbl1	N/A	N/A
10478012	Ube2i	N/A	N/A
10478014	Vstm2l	N/A	N/A
10478022	Rprd1b	N/A	N/A
10478066	Snhg11	N/A	N/A
10478073	---	N/A	N/A
10478075	---	N/A	N/A
10478124	Slc32a1	N/A	N/A
10478133	Actr5	N/A	N/A
10478145	Ppp1r16b	N/A	N/A
10478169	Dhx35	N/A	N/A
10478196	Top1	N/A	N/A
10478281	---	N/A	N/A
10478285	9430021M05Rik	N/A	N/A
10478289	---	N/A	N/A
10478291	Srsf6	N/A	N/A
10478326	Sgk2	N/A	N/A
10478341	Ift52	N/A	N/A
10478364	Tox2	N/A	N/A
10478374	Gdap1l1	N/A	N/A
10478389	Hnf4a	N/A	N/A
10478401	Ttpal	N/A	N/A
10478407	0610039K10Rik /// Serinc3	N/A	N/A
10478421	Kcnk15	N/A	N/A
10478424	Ywhab	N/A	N/A
10478447	Stk4	N/A	N/A
10478487	Sys1	N/A	N/A
10478508	Pigt	N/A	N/A
10478523	---	N/A	N/A
10478533	---	N/A	N/A
10478540	Wfdc10	N/A	N/A
10478560	Dnttip1	N/A	N/A
10478587	Zswim3 /// Acot8	N/A	N/A
10478590	Zswim1	N/A	N/A
10478594	Ctsa	N/A	N/A
10478615	Pcif1	N/A	N/A
10478647	Slc12a5	N/A	N/A
10478718	Ncoa3	N/A	N/A
10478744	---	N/A	N/A
10478746	LOC280487	N/A	N/A
10478772	Arfgef2	N/A	N/A
10478776	Arfgef2	N/A	N/A
10478778	Arfgef2	N/A	N/A
10478799	Cse1l	N/A	N/A
10478825	Ddx27	N/A	N/A
10478847	1500012F01Rik /// Znfx1	N/A	N/A
10478854	Slc9a8	N/A	N/A
10478890	Cebpb	N/A	N/A
10478897	Ptpn1	N/A	N/A
10478907	Pard6b	N/A	N/A
10478922	---	N/A	N/A
10478924	Gm9873	N/A	N/A
10478926	1700101G07Rik	N/A	N/A
10478928	Tshz2	N/A	N/A
10478936	---	N/A	N/A
10478938	Hax1	N/A	N/A
10478943	Pfdn4 /// Cyp24a1	N/A	N/A
10478949	Dok5	N/A	N/A
10478959	Mc3r	N/A	N/A
10478962	2010011I20Rik	N/A	N/A
10478967	Cstf1	N/A	N/A
10478983	2410001C21Rik	N/A	N/A
10478994	1700029J11Rik	N/A	N/A
10479047	Pck1	N/A	N/A
10479063	Rab22a	N/A	N/A
10479074	1700010B08Rik	N/A	N/A
10479087	Stx16	N/A	N/A
10479099	Npepl1	N/A	N/A
10479112	Gnas	N/A	N/A
10479136	Th1l	N/A	N/A
10479154	Tubb1	N/A	N/A
10479159	Zfp831	N/A	N/A
10479172	---	N/A	N/A
10479174	Rps8 /// Gm11353 /// Rps8-ps1	N/A	N/A
10479176	Gm6710	N/A	N/A
10479182	0610010B08Rik	N/A	N/A
10479185	---	N/A	N/A
10479187	Rps8 /// Gm11353 /// Rps8-ps1	N/A	N/A
10479189	0610010B08Rik /// Gm14430	N/A	N/A
10479192	0610010B08Rik /// Gm14430 /// Gm14434	N/A	N/A
10479195	100043387 /// 0610010B08Rik	N/A	N/A
10479198	0610010B08Rik	N/A	N/A
10479203	Gm14322 /// 2210418O10Rik /// Gm14403 /// LOC664987	N/A	N/A
10479215	Rps8 /// Gm11353 /// Rps8-ps1	N/A	N/A

10479217	Gm8898 /// Gm14431 /// Gm14430 /// Gm6710 /// Gm14420	N/A	N/A
10479221	Gm14403	N/A	N/A
10479228	Etohi1	N/A	N/A
10479230	Phactr3	N/A	N/A
10479247	9030418K01Rik	N/A	N/A
10479268	Wtap	N/A	N/A
10479274	Cdh4	N/A	N/A
10479294	---	N/A	N/A
10479297	Lsm14b	N/A	N/A
10479311	Ss18l1	N/A	N/A
10479324	Gtpbp5	N/A	N/A
10479335	Osbpl2	N/A	N/A
10479351	Adrm1	N/A	N/A
10479362	Rps21	N/A	N/A
10479375	Mir1-1	N/A	N/A
10479397	Ntsr1	N/A	N/A
10479411	Ogfr	N/A	N/A
10479458	2310003C23Rik	N/A	N/A
10479490	Arfgap1	N/A	N/A
10479510	9230112E08Rik /// Col20a1	N/A	N/A
10479556	BC051628	N/A	N/A
10479560	Rtel1	N/A	N/A
10479607	Lime1	N/A	N/A
10479615	---	N/A	N/A
10479625	BC050777	N/A	N/A
10479627	Tpd52l2	N/A	N/A
10479639	Dnajc5	N/A	N/A
10479649	Prpf6	N/A	N/A
10479672	Tcea2	N/A	N/A
10479685	Oprl1	N/A	N/A
10479698	Myt1	N/A	N/A
10479726	Pcmtd2	N/A	N/A
10479736	Polr3k	N/A	N/A
10479740	---	N/A	N/A
10479747	---	N/A	N/A
10479749	Rpp38	N/A	N/A
10479775	Hspa14	N/A	N/A
10479792	---	N/A	N/A
10479794	Prpf18	N/A	N/A
10479807	---	N/A	N/A
10479833	Optn	N/A	N/A
10479869	Cdc123	N/A	N/A
10479884	Nudt5 /// Gm13199	N/A	N/A
10479887	Sec61a2	N/A	N/A
10479902	Dhtkd1	N/A	N/A
10479938	Echdc3	N/A	N/A
10479948	Celf2	N/A	N/A
10479950	Celf2	N/A	N/A
10479971	Gm10855	N/A	N/A
10479973	Gm10115	N/A	N/A
10479975	---	N/A	N/A
10479979	Slc25a36	N/A	N/A
10479988	Taf3	N/A	N/A
10479996	Atp5c1	N/A	N/A
10480003	Itih2	N/A	N/A
10480032	Gapdh /// Gm16374 /// Gm2606 /// Gm4609 /// Gm3200 /// Gm2451 /// Gm10293	N/A	N/A
10480057	Rbm17	N/A	N/A
10480064	Fbxo18	N/A	N/A
10480087	---	N/A	N/A
10480121	Fam188a	N/A	N/A
10480139	C1ql3 /// Pter	N/A	N/A
10480145	Rsu1	N/A	N/A
10480238	St8sia6	N/A	N/A
10480254	Cacnb2	N/A	N/A
10480256	Gm10848	N/A	N/A
10480273	---	N/A	N/A
10480275	Nebl	N/A	N/A
10480284	Nebl	N/A	N/A
10480286	Nebl	N/A	N/A
10480314	---	N/A	N/A
10480321	A930004D18Rik	N/A	N/A
10480324	2810030E01Rik	N/A	N/A
10480329	Dnajc1	N/A	N/A
10480345	---	N/A	N/A
10480347	Pip4k2a	N/A	N/A
10480379	Mrps5	N/A	N/A
10480381	Arhgap21	N/A	N/A
10480414	Enkur	N/A	N/A
10480421	Pdss1	N/A	N/A
10480423	Abi1	N/A	N/A
10480445	Spopl	N/A	N/A
10480459	Hnmt	N/A	N/A
10480492	Cacna1b	N/A	N/A
10480570	Arrdc1	N/A	N/A

10480579	Mrpl41 /// Wdr85	N/A	N/A
10480583	---	N/A	N/A
10480601	A830007P12Rik	N/A	N/A
10480605	Cobra1	N/A	N/A
10480628	Tubb2c	N/A	N/A
10480646	Gm757	N/A	N/A
10480649	2310002J15Rik	N/A	N/A
10480652	Ndor1	N/A	N/A
10480672	Ssna1	N/A	N/A
10480676	Grin1	N/A	N/A
10480699	Dpp7	N/A	N/A
10480714	Uap1l1	N/A	N/A
10480734	Ptgds	N/A	N/A
10480751	C8g	N/A	N/A
10480808	Gm996	N/A	N/A
10480813	B230208H17Rik	N/A	N/A
10480842	Tmem141	N/A	N/A
10480878	Camsap1	N/A	N/A
10480901	Nacc2	N/A	N/A
10480921	Qsox2	N/A	N/A
10480937	C030048H21Rik	N/A	N/A
10480939	4932418E24Rik	N/A	N/A
10480999	Sdccag3	N/A	N/A
10481011	Inpp5e /// Pmpca	N/A	N/A
10481023	Sec16a	N/A	N/A
10481101	Snhg7 /// Snora43	N/A	N/A
10481111	---	N/A	N/A
10481122	Surf6	N/A	N/A
10481128	Med22	N/A	N/A
10481147	Surf4	N/A	N/A
10481164	Slc2a6	N/A	N/A
10481182	Fam163b	N/A	N/A
10481186	Sardh	N/A	N/A
10481259	Col5a1	N/A	N/A
10481272	1700007K13Rik	N/A	N/A
10481291	Gtf3c5	N/A	N/A
10481320	Gtf3c4	N/A	N/A
10481337	1700101E01Rik	N/A	N/A
10481344	Gapdh /// Gm2606	N/A	N/A
10481366	Rapgef1	N/A	N/A
10481368	Trub2 /// Coq4	N/A	N/A
10481378	Mir219-2	N/A	N/A
10481380	Cercam	N/A	N/A
10481383	Wdr34	N/A	N/A
10481393	Zdhhc12	N/A	N/A
10481401	Zer1	N/A	N/A
10481420	D2Wsu81e /// Endog	N/A	N/A
10481435	Ccbl1	N/A	N/A
10481451	1700084E18Rik /// Lrrc8a	N/A	N/A
10481453	Dolk	N/A	N/A
10481496	---	N/A	N/A
10481508	Asb6	N/A	N/A
10481518	Ptges	N/A	N/A
10481525	Tor1a	N/A	N/A
10481540	Fnbp1	N/A	N/A
10481566	Fibcd1	N/A	N/A
10481574	Fam78a	N/A	N/A
10481577	Uck1	N/A	N/A
10481585	2900010J23Rik	N/A	N/A
10481592	Dnm1	N/A	N/A
10481619	---	N/A	N/A
10481627	Lcn2	N/A	N/A
10481649	9430097D07Rik	N/A	N/A
10481654	Fpgs	N/A	N/A
10481670	Cdk9	N/A	N/A
10481678	6330409D20Rik	N/A	N/A
10481711	Stxbp1	N/A	N/A
10481772	Garnl3	N/A	N/A
10481804	Ralgps1	N/A	N/A
10481827	Zbtb34	N/A	N/A
10481830	Zbtb43	N/A	N/A
10481845	Fam125b	N/A	N/A
10481857	Pbx3	N/A	N/A
10481868	Dnajb6	N/A	N/A
10481870	Gapvd1	N/A	N/A
10481900	Rabepk	N/A	N/A
10481909	Fbxw2	N/A	N/A
10481920	Psmd5	N/A	N/A
10481962	Hc	N/A	N/A
10482004	AI182371	N/A	N/A
10482017	Rab14	N/A	N/A
10482030	Stom	N/A	N/A
10482073	---	N/A	N/A
10482075	Ttll11	N/A	N/A

10482109	Rbm18 /// Mrrf	N/A	N/A
10482117	Olfr341	N/A	N/A
10482119	---	N/A	N/A
10482121	Olfr3	N/A	N/A
10482123	Olfr351	N/A	N/A
10482125	Olfr353	N/A	N/A
10482127	Olfr355	N/A	N/A
10482129	Olfr358	N/A	N/A
10482131	Olfr360	N/A	N/A
10482133	Olfr361	N/A	N/A
10482135	Olfr362	N/A	N/A
10482139	Pdcl	N/A	N/A
10482144	Rc3h2	N/A	N/A
10482167	Zbtb6	N/A	N/A
10482172	Zbtb26 /// Zbtb6	N/A	N/A
10482177	Strbp	N/A	N/A
10482181	Strbp	N/A	N/A
10482200	Dennd1a	N/A	N/A
10482229	Psmb7	N/A	N/A
10482267	Rpl35 /// Gm10269	N/A	N/A
10482301	Scai	N/A	N/A
10482323	Ppp6c	N/A	N/A
10482330	Atp6v1g1	N/A	N/A
10482334	---	N/A	N/A
10482336	Lrp1b	N/A	N/A
10482432	---	N/A	N/A
10482434	Gtdc1	N/A	N/A
10482448	Zeb2	N/A	N/A
10482467	Orc4	N/A	N/A
10482484	---	N/A	N/A
10482486	Mmadhc	N/A	N/A
10482500	Rnd3	N/A	N/A
10482507	Ppia	N/A	N/A
10482517	Nmi	N/A	N/A
10482687	Arl5a	N/A	N/A
10482695	Cacnb4	N/A	N/A
10482712	Stam2	N/A	N/A
10482731	Prpf40a	N/A	N/A
10482762	Idi1	N/A	N/A
10482766	Rprm	N/A	N/A
10482788	Gpd2	N/A	N/A
10482791	---	N/A	N/A
10482793	---	N/A	N/A
10482795	Ermn	N/A	N/A
10482814	Acvr1c	N/A	N/A
10482846	Ccdc148	N/A	N/A
10482863	---	N/A	N/A
10482866	Tanc1	N/A	N/A
10482918	Gm13570	N/A	N/A
10482920	Cd302	N/A	N/A
10483023	Rbms1	N/A	N/A
10483025	Rbms1	N/A	N/A
10483046	Dpp4	N/A	N/A
10483110	Ifih1	N/A	N/A
10483131	Kcnh7	N/A	N/A
10483150	Fign	N/A	N/A
10483161	LOC280487	N/A	N/A
10483163	Grb14	N/A	N/A
10483215	Scn3a /// Scn1a	N/A	N/A
10483228	Scn3a	N/A	N/A
10483246	---	N/A	N/A
10483299	Scn1a	N/A	N/A
10483322	---	N/A	N/A
10483324	Scn9a	N/A	N/A
10483326	Scn9a	N/A	N/A
10483379	F830016D02Rik	N/A	N/A
10483381	Stk39	N/A	N/A
10483410	Abcb11	N/A	N/A
10483521	Fastkd1	N/A	N/A
10483536	4930578N16Rik	N/A	N/A
10483546	---	N/A	N/A
10483548	Mettl5 /// Ssb	N/A	N/A
10483559	---	N/A	N/A
10483563	Tlk1	N/A	N/A
10483604	Slc25a12	N/A	N/A
10483624	Dlx1as	N/A	N/A
10483631	Gm13637	N/A	N/A
10483633	Sp3	N/A	N/A
10483646	Sp3	N/A	N/A
10483648	Ola1	N/A	N/A
10483665	Sp9	N/A	N/A
10483667	Cir1	N/A	N/A
10483679	Gpr155	N/A	N/A
10483698	Wipf1	N/A	N/A

10483719	Chn1	N/A	N/A
10483737	Atf2	N/A	N/A
10483756	Atp5g3	N/A	N/A
10483761	---	N/A	N/A
10483766	---	N/A	N/A
10483768	---	N/A	N/A
10483770	Lnp	N/A	N/A
10483786	Mrpl23	N/A	N/A
10483806	Rps6 /// Gm16409	N/A	N/A
10483809	Nfe2l2	N/A	N/A
10483817	6030499A19Rik	N/A	N/A
10483819	Ttc30b	N/A	N/A
10483822	Ttc30b /// Ttc30a2 /// Ttc30a1	N/A	N/A
10483824	Ttc30a1	N/A	N/A
10483828	Pde11a	N/A	N/A
10483856	Prkra	N/A	N/A
10483865	Fkbp7	N/A	N/A
10484190	Ttn	N/A	N/A
10484197	Ccdc141	N/A	N/A
10484205	Ccdc141	N/A	N/A
10484227	Sestd1	N/A	N/A
10484256	4930401B11Rik	N/A	N/A
10484258	---	N/A	N/A
10484283	Pde1a	N/A	N/A
10484318	Nckap1	N/A	N/A
10484351	Sumo2	N/A	N/A
10484353	---	N/A	N/A
10484355	LOC280487	N/A	N/A
10484357	Rps4x	N/A	N/A
10484371	Calcrl	N/A	N/A
10484389	Tfpi	N/A	N/A
10484402	Ctnnd1	N/A	N/A
10484425	2700094K13Rik	N/A	N/A
10484431	Tmx2	N/A	N/A
10484457	Clp1	N/A	N/A
10484461	Mir130a	N/A	N/A
10484463	Serping1	N/A	N/A
10484486	P2rx3	N/A	N/A
10484503	Lrrc55	N/A	N/A
10484508	Olfr987	N/A	N/A
10484510	Olfr988	N/A	N/A
10484516	Olfr994	N/A	N/A
10484539	Olfr1006	N/A	N/A
10484543	Olfr1006	N/A	N/A
10484547	Olfr1016	N/A	N/A
10484549	Olfr1019	N/A	N/A
10484553	Olfr1024	N/A	N/A
10484559	Olfr1040	N/A	N/A
10484561	Olfr1042	N/A	N/A
10484563	Olfr1043	N/A	N/A
10484565	Olfr1044	N/A	N/A
10484567	Olfr52	N/A	N/A
10484569	Olfr1045	N/A	N/A
10484571	Olfr1046	N/A	N/A
10484584	---	N/A	N/A
10484586	Olfr1054	N/A	N/A
10484590	Olfr1056	N/A	N/A
10484592	Olfr1057	N/A	N/A
10484602	Olfr1065 /// Olfr1058	N/A	N/A
10484604	Olfr1065	N/A	N/A
10484606	Olfr1066	N/A	N/A
10484608	Olfr228	N/A	N/A
10484610	Olfr1077-ps1	N/A	N/A
10484614	Olfr1080	N/A	N/A
10484616	Olfr1082	N/A	N/A
10484618	Olfr1083	N/A	N/A
10484624	Olfr1086	N/A	N/A
10484626	Olfr1087	N/A	N/A
10484628	Olfr1089	N/A	N/A
10484630	Olfr1090	N/A	N/A
10484632	Olfr141	N/A	N/A
10484640	Olfr1099	N/A	N/A
10484642	Olfr1100	N/A	N/A
10484644	Olfr1101	N/A	N/A
10484646	---	N/A	N/A
10484648	Olfr1104	N/A	N/A
10484650	Olfr1105	N/A	N/A
10484652	Olfr1106	N/A	N/A
10484667	---	N/A	N/A
10484679	Olfr1132	N/A	N/A
10484681	Olfr1133	N/A	N/A
10484685	Olfr1135	N/A	N/A
10484687	Olfr1136	N/A	N/A
10484689	Olfr1137	N/A	N/A

10484691	Olfr1138	N/A	N/A
10484693	Olfr1141	N/A	N/A
10484695	---	N/A	N/A
10484697	Olfr1154	N/A	N/A
10484699	Olfr1155	N/A	N/A
10484704	Olfr1157	N/A	N/A
10484706	Olfr74	N/A	N/A
10484710	Olfr73	N/A	N/A
10484712	Olfr1163 /// Olfr1162	N/A	N/A
10484716	Olfr1164	N/A	N/A
10484718	Olfr1165-ps	N/A	N/A
10484726	Olfr1167 /// Olfr1170 /// Olfr1166	N/A	N/A
10484731	Olfr1173	N/A	N/A
10484733	Olfr1174-ps	N/A	N/A
10484735	Olfr1175-ps	N/A	N/A
10484739	Olfr1179	N/A	N/A
10484745	Olfr1181	N/A	N/A
10484747	Olfr1182	N/A	N/A
10484752	Olfr1195	N/A	N/A
10484754	Olfr1196 /// Olfr1197	N/A	N/A
10484756	Olfr1196 /// Olfr1197 /// Olfr1200	N/A	N/A
10484762	Olfr1197 /// Olfr1200	N/A	N/A
10484764	Olfr1208	N/A	N/A
10484766	Olfr1209	N/A	N/A
10484775	Olfr1215	N/A	N/A
10484779	Olfr1217	N/A	N/A
10484781	Olfr1218	N/A	N/A
10484783	Olfr1219	N/A	N/A
10484791	Olfr1222	N/A	N/A
10484793	Olfr1223	N/A	N/A
10484799	Olfr1225	N/A	N/A
10484801	Olfr1226	N/A	N/A
10484805	Olfr1229	N/A	N/A
10484807	Olfr1230	N/A	N/A
10484809	Olfr1231	N/A	N/A
10484813	Olfr1233	N/A	N/A
10484816	Olfr1234	N/A	N/A
10484818	Olfr1238	N/A	N/A
10484820	Olfr1239	N/A	N/A
10484822	Olfr1240	N/A	N/A
10484824	Olfr1241	N/A	N/A
10484839	Olfr1249	N/A	N/A
10484846	Olfr1252	N/A	N/A
10484852	Olfr1256	N/A	N/A
10484854	Olfr48	N/A	N/A
10484856	Olfr1259	N/A	N/A
10484861	Olfr140	N/A	N/A
10484863	Olfr1269 /// Olfr1265	N/A	N/A
10484865	---	N/A	N/A
10484867	Olfr1269	N/A	N/A
10484880	Olfr142	N/A	N/A
10484882	Olfr1271	N/A	N/A
10484884	Olfr1272	N/A	N/A
10484894	Ptprj	N/A	N/A
10484912	Ndufs3	N/A	N/A
10484920	Ptpmt1	N/A	N/A
10484925	Gm13777	N/A	N/A
10484927	Slc39a13	N/A	N/A
10484987	Nr1h3	N/A	N/A
10485027	F2	N/A	N/A
10485042	Zfp408	N/A	N/A
10485048	Atg13	N/A	N/A
10485068	---	N/A	N/A
10485115	Dgkz	N/A	N/A
10485117	Creb3l1	N/A	N/A
10485151	Mapk8ip1	N/A	N/A
10485170	Cry2	N/A	N/A
10485183	Slc35c1	N/A	N/A
10485191	Prdm11	N/A	N/A
10485198	Tspan18	N/A	N/A
10485213	Cd82	N/A	N/A
10485225	Ext2	N/A	N/A
10485277	---	N/A	N/A
10485280	Gm13889	N/A	N/A
10485282	Alkbh3	N/A	N/A
10485294	Hsd17b12	N/A	N/A
10485307	Mir129-2	N/A	N/A
10485309	---	N/A	N/A
10485312	Mir670	N/A	N/A
10485314	Ttc17	N/A	N/A
10485340	Itpa	N/A	N/A
10485342	2810002D19Rik /// Ttc17	N/A	N/A
10485344	Api5	N/A	N/A
10485355	LOC280487	N/A	N/A

10485357	---	N/A	N/A
10485378	Prr5l	N/A	N/A
10485388	Ldlrad3	N/A	N/A
10485395	Trim44	N/A	N/A
10485402	Fjx1	N/A	N/A
10485466	Cat	N/A	N/A
10485546	D430041D05Rik	N/A	N/A
10485550	D430041D05Rik	N/A	N/A
10485562	Hipk3	N/A	N/A
10485580	Cstf3	N/A	N/A
10485582	Tcp11l1	N/A	N/A
10485594	Pin1l	N/A	N/A
10485597	Depdc7	N/A	N/A
10485622	Qser1	N/A	N/A
10485633	Gm10796	N/A	N/A
10485635	Eif3m	N/A	N/A
10485641	---	N/A	N/A
10485643	AI314831	N/A	N/A
10485645	Rcn1	N/A	N/A
10485654	Rpl10	N/A	N/A
10485656	Elp4	N/A	N/A
10485667	Dnajc24	N/A	N/A
10485674	2700007P21Rik	N/A	N/A
10485685	Rpl35a /// Ino80 /// Gm10247	N/A	N/A
10485687	Mett5d1	N/A	N/A
10485698	---	N/A	N/A
10485700	Bbox1	N/A	N/A
10485711	Fibin	N/A	N/A
10485745	Ano3	N/A	N/A
10485767	Olfr1277	N/A	N/A
10485769	Olfr1282	N/A	N/A
10485771	Olfr1286	N/A	N/A
10485777	Olfr1294	N/A	N/A
10485790	Olfr1303	N/A	N/A
10485792	Gm13931	N/A	N/A
10485794	Olfr1305	N/A	N/A
10485798	Olfr1307	N/A	N/A
10485800	Olfr1308	N/A	N/A
10485802	Olfr1309	N/A	N/A
10485807	Olfr1311	N/A	N/A
10485809	Olfr1312	N/A	N/A
10485811	Olfr1313	N/A	N/A
10485813	Olfr1314	N/A	N/A
10485828	Slc12a6	N/A	N/A
10485830	Tmem85	N/A	N/A
10485836	Chrm5	N/A	N/A
10485840	Ryr3	N/A	N/A
10485955	Scg5	N/A	N/A
10485979	Gjd2	N/A	N/A
10485989	Aqr	N/A	N/A
10486026	Zfp770	N/A	N/A
10486041	Meis2	N/A	N/A
10486057	Rab5b	N/A	N/A
10486059	---	N/A	N/A
10486061	Rasgrp1	N/A	N/A
10486102	Gpr176	N/A	N/A
10486107	Srp14	N/A	N/A
10486112	Bmf	N/A	N/A
10486154	A430105I19Rik	N/A	N/A
10486166	Ccdc32	N/A	N/A
10486172	Fam82a2	N/A	N/A
10486185	Dnajc17	N/A	N/A
10486197	Rhov	N/A	N/A
10486201	Gm14207	N/A	N/A
10486203	Ino80	N/A	N/A
10486284	Ndufaf1	N/A	N/A
10486290	---	N/A	N/A
10486320	6330405D24Rik	N/A	N/A
10486322	6330405D24Rik	N/A	N/A
10486396	Ehd4	N/A	N/A
10486403	Pla2g4e	N/A	N/A
10486469	Vps39	N/A	N/A
10486522	Zfp106	N/A	N/A
10486552	Lrrc57	N/A	N/A
10486562	---	N/A	N/A
10486595	Ttbk2	N/A	N/A
10486616	Ubr1	N/A	N/A
10486710	Lcmt2	N/A	N/A
10486712	Zscan29	N/A	N/A
10486722	Trp53bp1	N/A	N/A
10486858	Mfap1b /// Mfap1a	N/A	N/A
10486867	Mfap1b /// Mfap1a	N/A	N/A
10486875	Frmd5	N/A	N/A
10486898	Spg11	N/A	N/A

10486954	Dppa4	N/A	N/A
10487011	Gatm	N/A	N/A
10487021	Slc30a4	N/A	N/A
10487033	Myef2	N/A	N/A
10487154	Secisbp2l	N/A	N/A
10487175	Cops2	N/A	N/A
10487267	Polr2l /// Usp50	N/A	N/A
10487277	Trpm7	N/A	N/A
10487321	2010106G01Rik	N/A	N/A
10487359	Itpripl1	N/A	N/A
10487371	Ciao1	N/A	N/A
10487380	Gm10766	N/A	N/A
10487392	Kcnip3	N/A	N/A
10487433	Zfp661	N/A	N/A
10487476	1500011K16Rik	N/A	N/A
10487506	Gm14005	N/A	N/A
10487508	Gm14005	N/A	N/A
10487510	---	N/A	N/A
10487513	Anapc1	N/A	N/A
10487575	Gm10762	N/A	N/A
10487588	Il1a	N/A	N/A
10487595	---	N/A	N/A
10487613	Pdyn	N/A	N/A
10487619	Gm10183	N/A	N/A
10487629	Idh3b	N/A	N/A
10487643	Rpl27a /// Gm5908 /// Rpl27a-ps1 /// Gm6378 /// Gm5453	N/A	N/A
10487685	Avp	N/A	N/A
10487690	Ubox5 /// Fastkd5	N/A	N/A
10487700	Prosapip1	N/A	N/A
10487711	Ddrgk1	N/A	N/A
10487748	4930402H24Rik	N/A	N/A
10487785	A730017L22Rik	N/A	N/A
10487871	Cenpb	N/A	N/A
10487894	Rassf2	N/A	N/A
10487906	Slc23a2	N/A	N/A
10487925	Rpl18	N/A	N/A
10487927	5730494N06Rik	N/A	N/A
10487930	Pcna	N/A	N/A
10487937	Prokr2	N/A	N/A
10487943	Gm10757	N/A	N/A
10488010	Hao1	N/A	N/A
10488020	Tmx4	N/A	N/A
10488029	Zfand1	N/A	N/A
10488031	---	N/A	N/A
10488033	Pak7	N/A	N/A
10488046	---	N/A	N/A
10488048	Mkks /// 2210009G21Rik	N/A	N/A
10488090	Tasp1	N/A	N/A
10488106	---	N/A	N/A
10488108	Esf1	N/A	N/A
10488145	---	N/A	N/A
10488154	---	N/A	N/A
10488195	Rrbp1	N/A	N/A
10488231	Rps2 /// Rps2-ps6 /// Gm5921 /// Gm6139	N/A	N/A
10488233	---	N/A	N/A
10488254	---	N/A	N/A
10488264	Calcoco2	N/A	N/A
10488266	6330439K17Rik	N/A	N/A
10488291	Rbbp9	N/A	N/A
10488299	Gm14092	N/A	N/A
10488303	Crnkl1	N/A	N/A
10488322	Ralgapa2	N/A	N/A
10488366	Nkx2-2	N/A	N/A
10488372	---	N/A	N/A
10488378	Thbd	N/A	N/A
10488382	Cd93	N/A	N/A
10488387	Napb	N/A	N/A
10488415	Cst3	N/A	N/A
10488430	Gm10750	N/A	N/A
10488437	C530025M09Rik	N/A	N/A
10488439	---	N/A	N/A
10488449	---	N/A	N/A
10488451	3300002I08Rik	N/A	N/A
10488456	---	N/A	N/A
10488469	Btbd1	N/A	N/A
10488472	2310001A20Rik	N/A	N/A
10488482	Acss1	N/A	N/A
10488507	Abhd12	N/A	N/A
10488565	Gm14160	N/A	N/A
10488575	Psmf1	N/A	N/A
10488594	Rbck1	N/A	N/A
10488617	Nrsn2 /// Gm14164	N/A	N/A
10488623	Sox12	N/A	N/A
10488626	Zcchc3	N/A	N/A

10488630	Defb23	N/A	N/A
10488652	Defb25	N/A	N/A
10488673	Foxs1	N/A	N/A
10488687	Pdrg1	N/A	N/A
10488693	Tspyl3	N/A	N/A
10488697	Plagl2	N/A	N/A
10488709	8430427H17Rik	N/A	N/A
10488722	Commd7	N/A	N/A
10488748	Cdk5rap1	N/A	N/A
10488762	Snta1	N/A	N/A
10488771	Necab3	N/A	N/A
10488797	Pxmp4	N/A	N/A
10488802	Zfp341	N/A	N/A
10488816	Ahcy	N/A	N/A
10488830	Pigu	N/A	N/A
10488844	Ncoa6	N/A	N/A
10488860	---	N/A	N/A
10488862	Ggt7	N/A	N/A
10488879	Gss	N/A	N/A
10488892	Trpc4ap	N/A	N/A
10488912	Edem2	N/A	N/A
10488944	Uqcc	N/A	N/A
10488959	6430550D23Rik	N/A	N/A
10488982	Rbm12 /// Cpne1 /// Nfs1	N/A	N/A
10489004	Nfs1	N/A	N/A
10489018	Rbm39	N/A	N/A
10489038	Scand1	N/A	N/A
10489041	Pdcd10	N/A	N/A
10489043	2900097C17Rik /// Gm7292	N/A	N/A
10489049	Rpl9 /// Gm5451	N/A	N/A
10489051	5730471H19Rik	N/A	N/A
10489053	4930518I15Rik	N/A	N/A
10489065	Ndrg3	N/A	N/A
10489092	9830001H06Rik	N/A	N/A
10489186	Rpl35a	N/A	N/A
10489195	Tti1	N/A	N/A
10489204	Tgm2	N/A	N/A
10489235	9430008C03Rik	N/A	N/A
10489237	---	N/A	N/A
10489239	---	N/A	N/A
10489246	Mafb	N/A	N/A
10489253	Zhx3	N/A	N/A
10489303	---	N/A	N/A
10489305	Ptprt	N/A	N/A
10489341	---	N/A	N/A
10489346	---	N/A	N/A
10489355	Jph2	N/A	N/A
10489364	3230401D17Rik	N/A	N/A
10489368	Fitm2 /// 2310001K24Rik	N/A	N/A
10489372	0610008F07Rik	N/A	N/A
10489377	Serinc3	N/A	N/A
10489406	Rims4	N/A	N/A
10489413	Tomm34	N/A	N/A
10489440	Wfdc15a	N/A	N/A
10489484	Sdc4	N/A	N/A
10489498	Dbndd2 /// Gm10725	N/A	N/A
10489532	Wfdc11	N/A	N/A
10489542	Wfdc3	N/A	N/A
10489553	Acot8	N/A	N/A
10489566	Ctsa /// Neurl2	N/A	N/A
10489569	Pltp /// Ctsa	N/A	N/A
10489589	Pcif1 /// 4930445K14Rik	N/A	N/A
10489620	Ncoa5	N/A	N/A
10489629	Cdh22	N/A	N/A
10489660	Elmo2	N/A	N/A
10489694	Zfp334	N/A	N/A
10489701	4833422F24Rik	N/A	N/A
10489705	Slc13a3	N/A	N/A
10489719	2810408M09Rik	N/A	N/A
10489721	LOC280487	N/A	N/A
10489723	Zmynd8	N/A	N/A
10489759	Sulf2	N/A	N/A
10489782	---	N/A	N/A
10489784	Prex1	N/A	N/A
10489829	---	N/A	N/A
10489831	Stau1	N/A	N/A
10489870	---	N/A	N/A
10489872	Kcnb1	N/A	N/A
10489891	B4galt5	N/A	N/A
10489904	Spata2	N/A	N/A
10489912	Tmem189	N/A	N/A
10489936	Adnp /// Dpm1	N/A	N/A
10489946	Dpm1 /// Adnp	N/A	N/A
10489983	Gm10724	N/A	N/A

10489985	Atp9a	N/A	N/A
10490045	---	N/A	N/A
10490053	Zfp217	N/A	N/A
10490078	Sumo1	N/A	N/A
10490097	Cbln4	N/A	N/A
10490126	Rps29	N/A	N/A
10490129	Bmp7	N/A	N/A
10490150	Zbp1	N/A	N/A
10490159	Pmepa1	N/A	N/A
10490169	Ppp4r1l-ps	N/A	N/A
10490192	---	N/A	N/A
10490194	---	N/A	N/A
10490199	Mir296	N/A	N/A
10490201	Mir298	N/A	N/A
10490203	Gnas	N/A	N/A
10490212	Ctsz	N/A	N/A
10490221	Atp5e	N/A	N/A
10490225	Slmo2	N/A	N/A
10490244	Gm2049	N/A	N/A
10490246	Gm14326 /// Gm14399	N/A	N/A
10490248	---	N/A	N/A
10490250	Gm6710 /// LOC627901 /// 0610010B08Rik /// Gm14295 /// Gm14391 /// Gm14399	N/A	N/A
10490256	Rps8 /// Gm11353 /// Rps8-ps1	N/A	N/A
10490259	100043387 /// 0610010B08Rik	N/A	N/A
10490262	0610010B08Rik /// Gm14430	N/A	N/A
10490265	0610010B08Rik /// LOC66376 /// Gm2004	N/A	N/A
10490268	0610010B08Rik	N/A	N/A
10490271	Gm2049	N/A	N/A
10490273	100043387 /// 0610010B08Rik	N/A	N/A
10490276	Gm14322	N/A	N/A
10490287	Gm14407	N/A	N/A
10490289	---	N/A	N/A
10490291	0610010B08Rik	N/A	N/A
10490294	2810021G02Rik /// Gm14430 /// Gm6710 /// Gm14326 /// Gm14403 /// Gm14434 /// Gm14391 /// Etohi1	N/A	N/A
10490350	Ppp1r3d	N/A	N/A
10490352	Taf4a	N/A	N/A
10490370	Psma7	N/A	N/A
10490378	Hrh3	N/A	N/A
10490467	Cables2	N/A	N/A
10490544	Ythdf1	N/A	N/A
10490551	Nkain4	N/A	N/A
10490559	Chrna4	N/A	N/A
10490569	Kcnq2	N/A	N/A
10490602	Eef1a2	N/A	N/A
10490632	BC006779	N/A	N/A
10490653	Gmeb2	N/A	N/A
10490663	Stmn3	N/A	N/A
10490665	Stmn3	N/A	N/A
10490672	Arfrp1	N/A	N/A
10490706	Znf512b	N/A	N/A
10490724	Samd10	N/A	N/A
10490731	Sox18	N/A	N/A
10490734	---	N/A	N/A
10490736	Rgs19	N/A	N/A
10490751	---	N/A	N/A
10490768	Rps24-ps2	N/A	N/A
10490773	Hnrnph2	N/A	N/A
10490775	Gm10748	N/A	N/A
10490777	Zfhx4	N/A	N/A
10490794	Pkia	N/A	N/A
10490813	---	N/A	N/A
10490818	Stmn2	N/A	N/A
10490824	Rpl29	N/A	N/A
10490826	Zbtb10	N/A	N/A
10490833	---	N/A	N/A
10490838	Fabp5 /// Fabp5l2	N/A	N/A
10490845	Chmp4c	N/A	N/A
10490852	---	N/A	N/A
10490854	Gm13051 /// Zfp534 /// Gm13151 /// Gm13235	N/A	N/A
10490856	Ralyl	N/A	N/A
10490872	Lrrcc1	N/A	N/A
10490894	E2f5	N/A	N/A
10490913	Car3	N/A	N/A
10490923	Car2	N/A	N/A
10490931	Ythdf3	N/A	N/A
10490942	Mir124a-2	N/A	N/A
10490944	Cypt12	N/A	N/A
10490946	Hsp90aa1	N/A	N/A
10490953	---	N/A	N/A
10490955	Mtfr1	N/A	N/A
10490982	BC002189	N/A	N/A
10490984	---	N/A	N/A
10490986	4632415L05Rik	N/A	N/A
10490989	Cp	N/A	N/A

10491036	Nudt21	N/A	N/A
10491038	Tbl1xr1	N/A	N/A
10491056	Tbl1xr1	N/A	N/A
10491060	---	N/A	N/A
10491062	Rpl27a /// Gm5908 /// Rpl27a-ps1 /// Gm5453	N/A	N/A
10491064	---	N/A	N/A
10491066	Nlgn1 /// A830092H15Rik	N/A	N/A
10491081	---	N/A	N/A
10491083	Nceh1	N/A	N/A
10491091	Tnfsf10	N/A	N/A
10491136	Tnik	N/A	N/A
10491171	Slc2a2	N/A	N/A
10491182	Eif5a2	N/A	N/A
10491191	Rpl22l1 /// LOC100042049	N/A	N/A
10491212	Egfem1	N/A	N/A
10491229	Mir551b	N/A	N/A
10491231	Mynn	N/A	N/A
10491261	Sec62	N/A	N/A
10491279	Prkci	N/A	N/A
10491300	Skil	N/A	N/A
10491313	Cldn11	N/A	N/A
10491319	Kcnmb2	N/A	N/A
10491329	Zmat3 /// 4930429B21Rik	N/A	N/A
10491331	Pik3ca	N/A	N/A
10491354	Zfp639	N/A	N/A
10491363	Mfn1	N/A	N/A
10491385	Actl6a	N/A	N/A
10491406	Ndufb5	N/A	N/A
10491414	Usp13	N/A	N/A
10491436	---	N/A	N/A
10491438	Ttc14	N/A	N/A
10491455	Fxr1 /// Dnajc19	N/A	N/A
10491477	Sox2	N/A	N/A
10491484	---	N/A	N/A
10491486	Atp11b	N/A	N/A
10491518	---	N/A	N/A
10491520	---	N/A	N/A
10491522	A330050B17Rik /// Mccc1	N/A	N/A
10491526	Acad9	N/A	N/A
10491547	1810062G17Rik	N/A	N/A
10491551	Exosc9	N/A	N/A
10491595	4932438A13Rik	N/A	N/A
10491597	4932438A13Rik	N/A	N/A
10491599	4932438A13Rik	N/A	N/A
10491603	4932438A13Rik	N/A	N/A
10491605	4932438A13Rik	N/A	N/A
10491615	4932438A13Rik	N/A	N/A
10491617	4932438A13Rik	N/A	N/A
10491619	4932438A13Rik	N/A	N/A
10491623	4932438A13Rik	N/A	N/A
10491695	Bbs12	N/A	N/A
10491704	Spata5	N/A	N/A
10491730	Rps23 /// Gm8618	N/A	N/A
10491732	Fat4	N/A	N/A
10491751	---	N/A	N/A
10491753	Intu	N/A	N/A
10491780	Hspa4l	N/A	N/A
10491825	3110057O12Rik	N/A	N/A
10491835	Larp1b	N/A	N/A
10491846	---	N/A	N/A
10491858	---	N/A	N/A
10491877	D3Ertd751e	N/A	N/A
10491883	Acp1	N/A	N/A
10491885	Pcdh10	N/A	N/A
10491897	---	N/A	N/A
10491920	Elf2	N/A	N/A
10491922	Naa15	N/A	N/A
10491958	Gm10729	N/A	N/A
10491960	---	N/A	N/A
10491962	Foxo1	N/A	N/A
10491967	Gapdh /// Gm4609 /// Gm10293	N/A	N/A
10491993	Stoml3	N/A	N/A
10492002	Gm10727	N/A	N/A
10492004	---	N/A	N/A
10492006	Trpc4	N/A	N/A
10492045	Hnrnpa3	N/A	N/A
10492091	Smad9	N/A	N/A
10492102	Spg20	N/A	N/A
10492136	Dclk1	N/A	N/A
10492165	---	N/A	N/A
10492169	Mab21l1	N/A	N/A
10492172	---	N/A	N/A
10492174	Tm4sf4	N/A	N/A
10492180	Rnf13	N/A	N/A

10492193	---	N/A	N/A
10492195	Tsc22d2	N/A	N/A
10492205	Eif2a	N/A	N/A
10492220	2810407C02Rik	N/A	N/A
10492231	Med12l	N/A	N/A
10492300	Aadac	N/A	N/A
10492310	Mbnl1	N/A	N/A
10492325	Ube2v1	N/A	N/A
10492330	P2ry1	N/A	N/A
10492335	Rap2b	N/A	N/A
10492341	4631416L12Rik	N/A	N/A
10492355	Mme	N/A	N/A
10492381	Gmps	N/A	N/A
10492396	Vmn2r1	N/A	N/A
10492424	---	N/A	N/A
10492426	Acsf2	N/A	N/A
10492428	Tiparp /// 4931440P22Rik	N/A	N/A
10492442	Lekr1	N/A	N/A
10492452	---	N/A	N/A
10492454	---	N/A	N/A
10492456	Rsrc1	N/A	N/A
10492480	Gfm1	N/A	N/A
10492499	Mfsd1	N/A	N/A
10492516	Iqcj-schip1 /// Iqcj	N/A	N/A
10492522	Schip1 /// Iqcj-schip1	N/A	N/A
10492536	---	N/A	N/A
10492538	Gm10723	N/A	N/A
10492556	1110032F04Rik	N/A	N/A
10492582	Mir15b	N/A	N/A
10492584	Mir16-2	N/A	N/A
10492590	Ppm1l	N/A	N/A
10492598	Nmd3	N/A	N/A
10492625	---	N/A	N/A
10492628	Serpini1	N/A	N/A
10492640	Fstl5	N/A	N/A
10492658	Gapdh /// Gm2606 /// Gm4609 /// Gm2451 /// Gm10290 /// Gm10291	N/A	N/A
10492660	---	N/A	N/A
10492668	4930589L23Rik	N/A	N/A
10492671	Ppid	N/A	N/A
10492679	4930579G24Rik	N/A	N/A
10492699	Ctso	N/A	N/A
10492709	Accn5	N/A	N/A
10492720	Mtap9	N/A	N/A
10492735	Fgg	N/A	N/A
10492748	Fga	N/A	N/A
10492755	Fga	N/A	N/A
10492757	Plrg1	N/A	N/A
10492774	Dchs2	N/A	N/A
10492813	Tigd4	N/A	N/A
10492824	---	N/A	N/A
10492846	Pet112l	N/A	N/A
10492860	Higd1a	N/A	N/A
10492864	Sh3d19	N/A	N/A
10492888	---	N/A	N/A
10492890	Lrba	N/A	N/A
10492951	---	N/A	N/A
10492953	---	N/A	N/A
10492955	---	N/A	N/A
10492964	Cd5l	N/A	N/A
10492997	Etv3	N/A	N/A
10493009	Arhgef11 /// 4933430H15Rik	N/A	N/A
10493086	Hdgf	N/A	N/A
10493094	Mrpl24	N/A	N/A
10493103	Isg20l2	N/A	N/A
10493120	Gpatch4	N/A	N/A
10493177	Mef2d	N/A	N/A
10493189	Mef2d	N/A	N/A
10493191	Mir9-1	N/A	N/A
10493193	Cct3	N/A	N/A
10493203	0610031J06Rik	N/A	N/A
10493210	Smg5	N/A	N/A
10493235	Paqr6	N/A	N/A
10493243	Rpl21	N/A	N/A
10493247	Ubqln4	N/A	N/A
10493259	Ssr2	N/A	N/A
10493267	Arhgef2	N/A	N/A
10493292	2810403A07Rik	N/A	N/A
10493307	---	N/A	N/A
10493309	Rit1	N/A	N/A
10493317	Gon4l	N/A	N/A
10493335	5830417I10Rik /// Gon4l	N/A	N/A
10493343	---	N/A	N/A
10493345	---	N/A	N/A
10493347	Ash1l	N/A	N/A

10493377	---	N/A	N/A
10493380	---	N/A	N/A
10493394	Clk2	N/A	N/A
10493409	Scamp3	N/A	N/A
10493421	Fam189b	N/A	N/A
10493435	Gba	N/A	N/A
10493484	Krtcap2	N/A	N/A
10493490	Dpm3	N/A	N/A
10493494	Efna3	N/A	N/A
10493496	Adam15	N/A	N/A
10493498	Dcst1	N/A	N/A
10493519	Shc1	N/A	N/A
10493530	Pygo2	N/A	N/A
10493555	Kcnn3	N/A	N/A
10493565	Adar	N/A	N/A
10493585	Ube2q1	N/A	N/A
10493604	She	N/A	N/A
10493612	4933434E20Rik /// 1700094D03Rik	N/A	N/A
10493626	1700094D03Rik /// 4933434E20Rik	N/A	N/A
10493631	Mir190b	N/A	N/A
10493660	Nup210l	N/A	N/A
10493662	Nup210l	N/A	N/A
10493664	Nup210l	N/A	N/A
10493666	Nup210l	N/A	N/A
10493668	Nup210l	N/A	N/A
10493688	Nup210l	N/A	N/A
10493690	Nup210l	N/A	N/A
10493703	Jtb	N/A	N/A
10493709	Slc39a1	N/A	N/A
10493711	Crtc2	N/A	N/A
10493758	Gatad2b	N/A	N/A
10493789	S100a13	N/A	N/A
10493798	S100a16	N/A	N/A
10493809	S100a2	N/A	N/A
10493812	S100a4	N/A	N/A
10493856	Sprr2a3 /// Sprr2a1	N/A	N/A
10493858	Sprr2a3 /// Sprr2a1	N/A	N/A
10493889	Lce1c	N/A	N/A
10493891	Ywhaz	N/A	N/A
10493894	---	N/A	N/A
10493900	Lce3c	N/A	N/A
10493903	Lce3c /// Lce3b /// Lce3f /// Lce3a	N/A	N/A
10493984	Tchh	N/A	N/A
10493990	S100a11	N/A	N/A
10493999	Tdpoz2 /// Gm5773	N/A	N/A
10494001	Tdpoz4 /// Gm4858	N/A	N/A
10494003	Tdpoz3	N/A	N/A
10494005	Gm10696	N/A	N/A
10494023	Rorc	N/A	N/A
10494039	Lingo4	N/A	N/A
10494043	Tdrkh	N/A	N/A
10494060	Mrpl9	N/A	N/A
10494069	Celf3	N/A	N/A
10494085	Selenbp2	N/A	N/A
10494092	Pogz	N/A	N/A
10494114	Selenbp1 /// Selenbp2	N/A	N/A
10494122	Rfx5	N/A	N/A
10494137	Pi4kb	N/A	N/A
10494151	---	N/A	N/A
10494153	Vps72	N/A	N/A
10494170	Lysmd1	N/A	N/A
10494200	Cdc42se1	N/A	N/A
10494227	Lass2	N/A	N/A
10494238	Arnt	N/A	N/A
10494288	Golph3l	N/A	N/A
10494296	Rps10	N/A	N/A
10494299	Ensa	N/A	N/A
10494306	Mcl1	N/A	N/A
10494312	Aph1a /// Car14	N/A	N/A
10494322	Anp32e	N/A	N/A
10494332	---	N/A	N/A
10494335	Otud7b	N/A	N/A
10494351	Mtmr11	N/A	N/A
10494372	Sv2a	N/A	N/A
10494386	Hist2h2ab	N/A	N/A
10494388	Hist2h2be	N/A	N/A
10494390	Hist2h2aa1 /// Hist2h2aa2 /// Hist2h3c1 /// Hist2h2ac	N/A	N/A
10494395	Hist2h2aa1 /// Hist2h2aa2 /// Hist2h3b /// Hist2h3c2-ps /// Hist2h3c1 /// Hist1h3b /// Hist2h2ac	N/A	N/A
10494402	Hist2h3b /// Hist2h3c2-ps /// Hist2h3c1 /// Hist1h3b	N/A	N/A
10494405	Hist2h3c2-ps /// Hist2h3b /// Hist2h3c1 /// Hist1h3b	N/A	N/A
10494407	Hist2h2bb	N/A	N/A
10494411	Rnu1b1 /// Rnu1b6 /// Rnu1b2	N/A	N/A
10494413	Rnu1b1 /// Rnu1b6 /// Rnu1b2	N/A	N/A
10494415	---	N/A	N/A

10494417	Rnu1b1 /// Rnu1b6 /// Rnu1b2	N/A	N/A
10494421	Rnu1b1 /// Rnu1b6 /// Rnu1b2	N/A	N/A
10494423	Hfe2	N/A	N/A
10494428	Txnip	N/A	N/A
10494441	Ankrd34a	N/A	N/A
10494445	Lix1l	N/A	N/A
10494452	Rbm8a	N/A	N/A
10494460	Pex11b	N/A	N/A
10494509	Pias3	N/A	N/A
10494527	Rnf115	N/A	N/A
10494536	Pdzk1 /// Gpr89	N/A	N/A
10494548	Gja5	N/A	N/A
10494551	Acp6	N/A	N/A
10494565	Fmo5	N/A	N/A
10494574	Prkab2	N/A	N/A
10494595	Notch2	N/A	N/A
10494643	Hmgcs2	N/A	N/A
10494655	Zfp697	N/A	N/A
10494662	Ywhah	N/A	N/A
10494664	Wars2	N/A	N/A
10494735	Gdap2	N/A	N/A
10494751	---	N/A	N/A
10494753	---	N/A	N/A
10494769	Trim45	N/A	N/A
10494781	Igsf3	N/A	N/A
10494817	Ngf	N/A	N/A
10494821	Tspan2	N/A	N/A
10494832	Sike1	N/A	N/A
10494839	Csde1	N/A	N/A
10494910	Bcas2	N/A	N/A
10494924	Trim33	N/A	N/A
10494957	Ap4b1	N/A	N/A
10495001	Rsbn1	N/A	N/A
10495012	Phtf1	N/A	N/A
10495059	St7l	N/A	N/A
10495083	Kcnd3	N/A	N/A
10495094	6530418L21Rik	N/A	N/A
10495107	Adora3	N/A	N/A
10495111	Wdr77	N/A	N/A
10495120	Ovgp1	N/A	N/A
10495134	---	N/A	N/A
10495147	Dennd2d /// 2010016I18Rik	N/A	N/A
10495163	Dram2	N/A	N/A
10495173	4933421E11Rik	N/A	N/A
10495183	Kcna3	N/A	N/A
10495186	AI504432	N/A	N/A
10495193	Kcna2	N/A	N/A
10495197	Gm10672	N/A	N/A
10495199	Kcna10	N/A	N/A
10495243	Gstm5 /// 4933431E20Rik	N/A	N/A
10495252	Romo1	N/A	N/A
10495257	Ampd2 /// Gm12500	N/A	N/A
10495270	Amigo1	N/A	N/A
10495285	Sort1	N/A	N/A
10495330	---	N/A	N/A
10495332	1700013F07Rik	N/A	N/A
10495340	Taf13	N/A	N/A
10495343	Wdr47	N/A	N/A
10495359	Clcc1	N/A	N/A
10495405	Slc25a24	N/A	N/A
10495518	Olfm3	N/A	N/A
10495528	Gm9889	N/A	N/A
10495530	Dph5	N/A	N/A
10495549	Dbt	N/A	N/A
10495574	Sass6 /// Ccdc76	N/A	N/A
10495592	---	N/A	N/A
10495594	---	N/A	N/A
10495596	Frrs1	N/A	N/A
10495613	4833424O15Rik	N/A	N/A
10495621	Mir137	N/A	N/A
10495623	Gm9916	N/A	N/A
10495625	Dpyd	N/A	N/A
10495649	Gm10652	N/A	N/A
10495651	Alg14	N/A	N/A
10495657	Alg14	N/A	N/A
10495659	Cnn3	N/A	N/A
10495675	F3	N/A	N/A
10495763	Gclm	N/A	N/A
10495773	Dnttip2	N/A	N/A
10495781	Bcar3	N/A	N/A
10495820	Fabp2	N/A	N/A
10495830	Sec24d	N/A	N/A
10495867	Gm9372	N/A	N/A
10495869	Tram1l1	N/A	N/A

10495876	---	N/A	N/A
10495878	Ndst4	N/A	N/A
10495891	Arsj	N/A	N/A
10495896	Camk2d	N/A	N/A
10495929	Mir302d	N/A	N/A
10495933	4930422G04Rik	N/A	N/A
10495935	4930422G04Rik	N/A	N/A
10495964	Neurog2	N/A	N/A
10495972	Gm10650	N/A	N/A
10495974	---	N/A	N/A
10495989	Rpl7a /// Gm5619 /// Gm16477 /// Rpl7a-ps10	N/A	N/A
10495991	---	N/A	N/A
10496001	Cfi	N/A	N/A
10496077	Agxt2l1	N/A	N/A
10496110	Papss1	N/A	N/A
10496130	Tbck	N/A	N/A
10496159	Ints12	N/A	N/A
10496167	LOC280487	N/A	N/A
10496169	Ppa2	N/A	N/A
10496192	Tacr3	N/A	N/A
10496200	---	N/A	N/A
10496202	---	N/A	N/A
10496251	Bdh2	N/A	N/A
10496302	Manba	N/A	N/A
10496322	Gm9799	N/A	N/A
10496324	Slc39a8	N/A	N/A
10496336	LOC280487	N/A	N/A
10496338	Ppp3ca	N/A	N/A
10496357	---	N/A	N/A
10496359	Emcn	N/A	N/A
10496379	H2afz	N/A	N/A
10496387	Dnajb14	N/A	N/A
10496397	Mapksp1	N/A	N/A
10496417	Rg9mtd2	N/A	N/A
10496425	Adh7	N/A	N/A
10496438	Adh1	N/A	N/A
10496455	---	N/A	N/A
10496462	Adh6-ps1	N/A	N/A
10496466	Adh4	N/A	N/A
10496475	Adh5	N/A	N/A
10496490	Mir1956 /// Eif4e	N/A	N/A
10496494	Tspan5	N/A	N/A
10496519	Unc5c	N/A	N/A
10496569	Gbp6	N/A	N/A
10496605	Ccbl2	N/A	N/A
10496626	Gapdh /// Gm16374 /// Gm2606 /// Gm4609 /// Gm3200 /// Gm2451 /// Gm10293	N/A	N/A
10496629	Sep 15	N/A	N/A
10496638	Odf2l	N/A	N/A
10496715	Znhit6	N/A	N/A
10496727	Ddah1	N/A	N/A
10496735	Rpl36a	N/A	N/A
10496796	Ssx2ip	N/A	N/A
10496813	Ctbs /// Spata1	N/A	N/A
10496822	Gng5	N/A	N/A
10496825	Uox	N/A	N/A
10496835	Hmgb1	N/A	N/A
10496837	Ttll7	N/A	N/A
10496854	Ttll7	N/A	N/A
10496862	---	N/A	N/A
10496872	Eltd1	N/A	N/A
10496888	---	N/A	N/A
10496890	Gipc2	N/A	N/A
10496892	Fubp1	N/A	N/A
10496917	---	N/A	N/A
10496919	Usp33	N/A	N/A
10496956	Pigk	N/A	N/A
10497001	Cryz	N/A	N/A
10497012	4922501L14Rik	N/A	N/A
10497031	---	N/A	N/A
10497045	---	N/A	N/A
10497051	Negr1	N/A	N/A
10497064	---	N/A	N/A
10497066	Zranb2	N/A	N/A
10497077	Mir186	N/A	N/A
10497086	---	N/A	N/A
10497090	Ankrd13c	N/A	N/A
10497105	Lrrc40	N/A	N/A
10497149	Wls	N/A	N/A
10497167	---	N/A	N/A
10497169	---	N/A	N/A
10497173	Pxmp3	N/A	N/A
10497186	---	N/A	N/A
10497197	5330432E05Rik	N/A	N/A
10497199	Tmem189 /// Gm6194	N/A	N/A

10497201	---	N/A	N/A
10497203	Hey1	N/A	N/A
10497222	Zfp704	N/A	N/A
10497237	Pag1	N/A	N/A
10497248	---	N/A	N/A
10497250	---	N/A	N/A
10497285	Impa1	N/A	N/A
10497296	Slc10a5	N/A	N/A
10497300	Zfand1	N/A	N/A
10497309	Snx16	N/A	N/A
10497321	Pgam1	N/A	N/A
10497327	LOC280487	N/A	N/A
10497335	1810022K09Rik	N/A	N/A
10497337	Car1	N/A	N/A
10497356	Sirpb1a /// LOC100038947 /// Sirpb1b	N/A	N/A
10497376	---	N/A	N/A
10497381	Cyp7b1	N/A	N/A
10497390	Armc1	N/A	N/A
10497399	Pde7a	N/A	N/A
10497421	Hps3	N/A	N/A
10497441	Gyg	N/A	N/A
10497481	2810416G20Rik	N/A	N/A
10497483	Hmgb1	N/A	N/A
10497485	---	N/A	N/A
10497487	---	N/A	N/A
10497490	Naaladl2	N/A	N/A
10497501	Naaladl2	N/A	N/A
10497503	Kpna2	N/A	N/A
10497505	Nlgn1	N/A	N/A
10497580	---	N/A	N/A
10497582	Tmem212	N/A	N/A
10497587	1600012F09Rik	N/A	N/A
10497608	Mecom	N/A	N/A
10497613	Gm10258	N/A	N/A
10497615	1600017P15Rik	N/A	N/A
10497644	Sec62	N/A	N/A
10497646	Phc3	N/A	N/A
10497648	Phc3	N/A	N/A
10497663	Slc7a14	N/A	N/A
10497673	Zmat3	N/A	N/A
10497689	Gnb4	N/A	N/A
10497703	Mrpl47	N/A	N/A
10497711	Ndufb5 /// Mrpl47	N/A	N/A
10497713	Pex5l	N/A	N/A
10497731	Ccdc39 /// Ttc14	N/A	N/A
10497752	Carhsp1	N/A	N/A
10497754	Dnajc19	N/A	N/A
10497773	Mccc1	N/A	N/A
10497817	Anxa5	N/A	N/A
10497842	Bbs7	N/A	N/A
10497862	Trpc3	N/A	N/A
10497894	Cetn4	N/A	N/A
10497899	---	N/A	N/A
10497918	pl21 /// Gm6813 /// Gm9104 /// Rpl21-ps4 /// Rpl21-ps7 /// Gm16416 /// Rpl21-ps12 /// Rpl21-ps14 /// Rpl21-ps10 /// Gm80	N/A	N/A
10497920	Ankrd50	N/A	N/A
10497929	---	N/A	N/A
10497931	Cflar	N/A	N/A
10497958	1700034I23Rik	N/A	N/A
10497964	Pgrmc2	N/A	N/A
10497968	Gm16508	N/A	N/A
10497971	Sclt1	N/A	N/A
10497994	Pabpc4l	N/A	N/A
10497996	Ikzf5	N/A	N/A
10497998	---	N/A	N/A
10498018	Pcdh18	N/A	N/A
10498038	Elf2	N/A	N/A
10498058	Ndufc1	N/A	N/A
10498064	Setd7	N/A	N/A
10498093	---	N/A	N/A
10498095	Cog6	N/A	N/A
10498117	Rpl29	N/A	N/A
10498146	Nhlrc3	N/A	N/A
10498158	Gm10726	N/A	N/A
10498160	Ufm1	N/A	N/A
10498166	---	N/A	N/A
10498168	Exosc8	N/A	N/A
10498180	Rfxap	N/A	N/A
10498185	Tra2b	N/A	N/A
10498187	6030405A18Rik	N/A	N/A
10498210	Nbea	N/A	N/A
10498273	Tm4sf1	N/A	N/A
10498284	Wwtr1	N/A	N/A
10498296	Commd2	N/A	N/A
10498302	Gm410	N/A	N/A

10498307	---	N/A	N/A
10498309	Pfn2	N/A	N/A
10498313	Pgk1	N/A	N/A
10498319	Serp1	N/A	N/A
10498332	Siah2	N/A	N/A
10498343	---	N/A	N/A
10498350	P2ry14	N/A	N/A
10498357	F630111L10Rik	N/A	N/A
10498367	P2ry13	N/A	N/A
10498379	Igsf10	N/A	N/A
10498383	Igsf10	N/A	N/A
10498386	Igsf10	N/A	N/A
10498401	---	N/A	N/A
10498403	---	N/A	N/A
10498405	Gapdh /// Gm12070	N/A	N/A
10498413	9330121J05Rik	N/A	N/A
10498415	Dhx36	N/A	N/A
10498441	Gpr149	N/A	N/A
10498446	---	N/A	N/A
10498477	E130311K13Rik	N/A	N/A
10498485	Slc33a1	N/A	N/A
10498519	Ssr3	N/A	N/A
10498526	4931440P22Rik	N/A	N/A
10498531	Ccnl1	N/A	N/A
10498566	---	N/A	N/A
10498576	Lxn	N/A	N/A
10498584	Rarres1	N/A	N/A
10498591	---	N/A	N/A
10498593	---	N/A	N/A
10498595	Snrpd2 /// Gm5449 /// Gm10120	N/A	N/A
10498597	1110032F04Rik	N/A	N/A
10498599	Ift80	N/A	N/A
10498620	Trim59	N/A	N/A
10498623	Kpna4	N/A	N/A
10498647	B3galnt1	N/A	N/A
10498651	Rpl32-ps	N/A	N/A
10498653	1110032A04Rik	N/A	N/A
10498707	Slitrk3	N/A	N/A
10498710	Bche	N/A	N/A
10498720	Zbbx	N/A	N/A
10498763	Wdr49	N/A	N/A
10498771	Pdcd10	N/A	N/A
10498795	Zfp108 /// Zfp114 /// Zfp111 /// Zfp109	N/A	N/A
10498800	Ube2d2	N/A	N/A
10498802	Rapgef2	N/A	N/A
10498827	Fnip2	N/A	N/A
10498837	Etfdh	N/A	N/A
10498885	Gria2	N/A	N/A
10498907	Glrb	N/A	N/A
10498919	Gm9989	N/A	N/A
10498921	Tdo2	N/A	N/A
10498952	Gucy1a3	N/A	N/A
10498965	Npy2r	N/A	N/A
10498978	Lrat	N/A	N/A
10498981	Fgb	N/A	N/A
10498990	Gm10710	N/A	N/A
10499045	Trim2	N/A	N/A
10499080	Arfip1	N/A	N/A
10499089	---	N/A	N/A
10499091	Fbxw7 /// Dear1	N/A	N/A
10499093	---	N/A	N/A
10499095	Fam160a1	N/A	N/A
10499121	Rps3a /// Gm9000 /// Gm10119	N/A	N/A
10499128	Rnu73a	N/A	N/A
10499130	Rnu73b	N/A	N/A
10499138	Dclk2	N/A	N/A
10499160	Cd1d1	N/A	N/A
10499187	Ube2l3 /// Gm10145	N/A	N/A
10499198	Rpl30 /// Gm12191 /// Gm6570 /// Gm6109	N/A	N/A
10499265	Prcc	N/A	N/A
10499273	Mrpl24	N/A	N/A
10499285	Bcan	N/A	N/A
10499309	Apoa1bp	N/A	N/A
10499342	1700021C14Rik /// Rhbg	N/A	N/A
10499358	Bglap /// Bglap-rs1 /// Bglap2	N/A	N/A
10499363	Bglap	N/A	N/A
10499366	Pmf1	N/A	N/A
10499372	Slc25a44	N/A	N/A
10499425	2700060E02Rik	N/A	N/A
10499427	Rxfp4	N/A	N/A
10499429	Gm10253	N/A	N/A
10499431	Syt11	N/A	N/A
10499438	Msto1	N/A	N/A
10499454	Dap3	N/A	N/A

10499470	Rusc1	N/A	N/A
10499491	Mir720	N/A	N/A
10499493	Hcn3	N/A	N/A
10499504	Mtx1	N/A	N/A
10499512	Mir92b	N/A	N/A
10499529	Rag1ap1	N/A	N/A
10499536	Efna1	N/A	N/A
10499545	Efna3	N/A	N/A
10499560	Adam15	N/A	N/A
10499620	Lenep /// Flad1	N/A	N/A
10499639	Cks1b	N/A	N/A
10499643	Chrnb2	N/A	N/A
10499652	4632404H12Rik	N/A	N/A
10499655	Il6ra	N/A	N/A
10499703	---	N/A	N/A
10499705	Hax1	N/A	N/A
10499716	Ubap2l	N/A	N/A
10499748	Rps27 /// Gm9846	N/A	N/A
10499766	Slc27a3	N/A	N/A
10499777	Ints3	N/A	N/A
10499839	Snapin /// Ilf2	N/A	N/A
10499854	S100a1	N/A	N/A
10499859	---	N/A	N/A
10499861	S100a9	N/A	N/A
10499870	Lor	N/A	N/A
10499876	Lelp1	N/A	N/A
10499879	Rpl3	N/A	N/A
10499881	Rpl3	N/A	N/A
10499883	Adrm1	N/A	N/A
10499902	Sprr4	N/A	N/A
10499904	Ivl	N/A	N/A
10499909	Lce6a	N/A	N/A
10499927	Lce1f	N/A	N/A
10499930	Lce1g	N/A	N/A
10499935	Lce1i	N/A	N/A
10499937	---	N/A	N/A
10499939	Lce1k	N/A	N/A
10499948	Lce3a	N/A	N/A
10499950	---	N/A	N/A
10499952	Crct1	N/A	N/A
10499961	Tdpoz2	N/A	N/A
10499967	---	N/A	N/A
10499969	Gm10696	N/A	N/A
10499971	Tdpoz1 /// Gm9125	N/A	N/A
10499973	Gm10697 /// Tdpoz5	N/A	N/A
10499975	Tdpoz1 /// Gm9125	N/A	N/A
10499977	Gm10697 /// Tdpoz5	N/A	N/A
10499979	Gm10696	N/A	N/A
10499996	Snx27	N/A	N/A
10500009	Rpl31 /// Gm16382	N/A	N/A
10500011	Tuft1	N/A	N/A
10500034	Psmb4	N/A	N/A
10500042	Zfp687	N/A	N/A
10500054	Psmd4	N/A	N/A
10500069	Pip5k1a	N/A	N/A
10500089	---	N/A	N/A
10500091	Scnm1	N/A	N/A
10500100	Tnfaip8l2	N/A	N/A
10500103	Gabpb2	N/A	N/A
10500114	Mllt11	N/A	N/A
10500133	Prune	N/A	N/A
10500155	Gm10691	N/A	N/A
10500157	Setdb1	N/A	N/A
10500181	---	N/A	N/A
10500204	Ecm1	N/A	N/A
10500218	Tars2	N/A	N/A
10500237	Rprd2	N/A	N/A
10500283	Aph1a /// Car14	N/A	N/A
10500293	---	N/A	N/A
10500304	Vps45	N/A	N/A
10500321	Bola1	N/A	N/A
10500329	Hist2h2aa1 /// Hist2h2aa2 /// Hist2h3c1 /// Hist2h2ac	N/A	N/A
10500333	Hist2h4	N/A	N/A
10500343	Rnu1b1 /// Rnu1b6 /// Rnu1b2	N/A	N/A
10500345	Terc	N/A	N/A
10500356	Rnu1b1 /// Rnu1b6 /// Rnu1b2	N/A	N/A
10500358	Rnu1b1 /// Rnu1b6 /// Rnu1b2	N/A	N/A
10500360	---	N/A	N/A
10500362	Polr3gl	N/A	N/A
10500371	6330549D23Rik	N/A	N/A
10500376	Rpl21	N/A	N/A
10500388	Polr3c	N/A	N/A
10500404	---	N/A	N/A
10500412	Gpr89	N/A	N/A

10500429	Gja8	N/A	N/A
10500434	Bcl9	N/A	N/A
10500443	Olfr1402	N/A	N/A
10500445	Chd1l	N/A	N/A
10500469	Pde4dip	N/A	N/A
10500527	LOC280487	N/A	N/A
10500534	Hsd3b4 /// Hsd3b5	N/A	N/A
10500539	Hsd3b4 /// Hsd3b5	N/A	N/A
10500545	Hsd3b5	N/A	N/A
10500547	Hsd3b2	N/A	N/A
10500555	Hsd3b3	N/A	N/A
10500580	---	N/A	N/A
10500610	Fam46c	N/A	N/A
10500614	Man1a2	N/A	N/A
10500666	Ptgfrn	N/A	N/A
10500683	Rangrf /// Gm10355	N/A	N/A
10500720	Slc22a15	N/A	N/A
10500736	Vangl1	N/A	N/A
10500796	---	N/A	N/A
10500798	---	N/A	N/A
10500802	Atg4a /// Syt6	N/A	N/A
10500808	Olfml3	N/A	N/A
10500813	Hipk1	N/A	N/A
10500837	Dclre1b	N/A	N/A
10500845	A130049A11Rik	N/A	N/A
10500847	Magi3	N/A	N/A
10500870	Gm5546	N/A	N/A
10500876	Lrig2	N/A	N/A
10500896	H3f3b	N/A	N/A
10500911	Mov10	N/A	N/A
10500928	Capza1	N/A	N/A
10500948	Cttnbp2nl	N/A	N/A
10500957	Kcnd3	N/A	N/A
10500960	Ddx20	N/A	N/A
10500976	Rap1a	N/A	N/A
10500990	Atp5f1	N/A	N/A
10501046	Gm10673	N/A	N/A
10501048	2010016I18Rik /// Dennd2d	N/A	N/A
10501051	Cept1 /// Dram2	N/A	N/A
10501072	Olfr266	N/A	N/A
10501091	Rbm15	N/A	N/A
10501096	---	N/A	N/A
10501104	Slc6a17	N/A	N/A
10501121	Fam40a	N/A	N/A
10501143	Ahcyl1	N/A	N/A
10501183	Eps8l3 /// 4933431E20Rik	N/A	N/A
10501199	Gstm7	N/A	N/A
10501208	Gstm6	N/A	N/A
10501218	Gstm3	N/A	N/A
10501222	Gstm2	N/A	N/A
10501229	Gstm1	N/A	N/A
10501235	Gstm4	N/A	N/A
10501244	Ampd2	N/A	N/A
10501282	Cyb561d1	N/A	N/A
10501286	Atxn7l2	N/A	N/A
10501313	---	N/A	N/A
10501315	Mybphl	N/A	N/A
10501319	Celsr2	N/A	N/A
10501358	Sars	N/A	N/A
10501372	---	N/A	N/A
10501374	5330417C22Rik	N/A	N/A
10501397	Tmem167b	N/A	N/A
10501420	Stxbp3a	N/A	N/A
10501454	4921515J06Rik /// Gm9857	N/A	N/A
10501456	Fam102b	N/A	N/A
10501468	Ntng1	N/A	N/A
10501483	Ntng1	N/A	N/A
10501485	Prmt6	N/A	N/A
10501489	Hectd1	N/A	N/A
10501492	---	N/A	N/A
10501494	Amy2a5 /// Amy2b /// Amy2a4	N/A	N/A
10501500	Amy2a5 /// Amy2b /// Amy2a4	N/A	N/A
10501511	Amy2a5 /// Amy2b /// Amy2a4	N/A	N/A
10501522	Amy2a5 /// Amy2b /// Amy2a4	N/A	N/A
10501533	Amy2a5 /// Amy2b /// Amy2a4	N/A	N/A
10501555	Amy1	N/A	N/A
10501567	Rnpc3	N/A	N/A
10501591	A930005H10Rik	N/A	N/A
10501593	Slc30a7	N/A	N/A
10501649	Rtcd1	N/A	N/A
10501661	Srsf3	N/A	N/A
10501676	Hiat1	N/A	N/A
10501690	Slc35a3	N/A	N/A
10501734	Palmd	N/A	N/A

10501742	Rpl7a-ps3	N/A	N/A
10501754	D3Bwg0562e	N/A	N/A
10501775	---	N/A	N/A
10501778	Ptbp2	N/A	N/A
10501795	Rwdd3	N/A	N/A
10501802	Tmem56	N/A	N/A
10501811	Slc44a3	N/A	N/A
10501832	Abcd3	N/A	N/A
10501857	Gapdh /// Gm16374 /// Gm2606 /// Gm4609 /// Gm3200 /// Gm2451 /// Gm10293	N/A	N/A
10501860	Fnbp1l	N/A	N/A
10501879	Usp53	N/A	N/A
10501903	Synpo2	N/A	N/A
10501922	Snhg8 /// Snora24	N/A	N/A
10501924	Ndst3	N/A	N/A
10501963	Ugt8a	N/A	N/A
10501971	Ank2	N/A	N/A
10502050	Alpk1	N/A	N/A
10502052	Alpk1	N/A	N/A
10502058	Ap1ar	N/A	N/A
10502076	---	N/A	N/A
10502079	---	N/A	N/A
10502081	Enpep	N/A	N/A
10502146	Gar1	N/A	N/A
10502165	Sec24b	N/A	N/A
10502191	Ostc	N/A	N/A
10502196	Rpl34	N/A	N/A
10502199	---	N/A	N/A
10502205	Hadh	N/A	N/A
10502214	Cyp2u1	N/A	N/A
10502224	Sgms2	N/A	N/A
10502232	Aimp1	N/A	N/A
10502284	Tet2	N/A	N/A
10502329	Cisd2	N/A	N/A
10502333	Gm16500	N/A	N/A
10502375	Mttp	N/A	N/A
10502405	Metap1	N/A	N/A
10502417	2410002F23Rik	N/A	N/A
10502419	Rap1gds1	N/A	N/A
10502438	Gm4862	N/A	N/A
10502440	---	N/A	N/A
10502469	Pdlim5	N/A	N/A
10502482	---	N/A	N/A
10502484	Pkn2	N/A	N/A
10502510	Lmo4	N/A	N/A
10502522	Hs2st1	N/A	N/A
10502535	---	N/A	N/A
10502655	Cyr61	N/A	N/A
10502682	---	N/A	N/A
10502686	Dnase2b	N/A	N/A
10502714	Rpf1	N/A	N/A
10502732	Prkacb	N/A	N/A
10502748	Lphn2	N/A	N/A
10502766	Lphn2	N/A	N/A
10502770	Lphn2	N/A	N/A
10502772	Lphn2	N/A	N/A
10502774	Lphn2	N/A	N/A
10502778	Lphn2	N/A	N/A
10502780	Lphn2	N/A	N/A
10502783	Gm9912	N/A	N/A
10502785	Gm10287	N/A	N/A
10502787	Rpsa /// Rpsa-ps10	N/A	N/A
10502791	Ifi44	N/A	N/A
10502823	Dnajb4	N/A	N/A
10502845	Fam73a	N/A	N/A
10502863	Ak5	N/A	N/A
10502881	St6galnac5	N/A	N/A
10502890	St6galnac3	N/A	N/A
10502898	Ube2l3 /// Gm10145	N/A	N/A
10502900	5730460C07Rik	N/A	N/A
10502934	Rabggtb	N/A	N/A
10502949	---	N/A	N/A
10502951	Acadm	N/A	N/A
10502961	Lhx8	N/A	N/A
10502973	Tyw3	N/A	N/A
10503008	---	N/A	N/A
10503019	4930566N20Rik	N/A	N/A
10503023	Cth	N/A	N/A
10503036	Srsf11	N/A	N/A
10503054	Lrrc7	N/A	N/A
10503085	Tgs1	N/A	N/A
10503098	Lyn	N/A	N/A
10503107	6330407A03Rik	N/A	N/A
10503116	LOC100036540	N/A	N/A
10503118	Fam110b	N/A	N/A

10503123	Ubxn2b	N/A	N/A
10503134	Sdcbp	N/A	N/A
10503145	---	N/A	N/A
10503148	---	N/A	N/A
10503161	Chd7	N/A	N/A
10503166	Chd7	N/A	N/A
10503168	Chd7	N/A	N/A
10503170	Chd7	N/A	N/A
10503172	Chd7	N/A	N/A
10503174	Chd7	N/A	N/A
10503176	Chd7	N/A	N/A
10503178	Chd7	N/A	N/A
10503180	Chd7	N/A	N/A
10503182	Chd7	N/A	N/A
10503184	Chd7	N/A	N/A
10503186	Chd7	N/A	N/A
10503188	Chd7	N/A	N/A
10503192	Chd7	N/A	N/A
10503194	Chd7	N/A	N/A
10503196	Chd7	N/A	N/A
10503198	Chd7	N/A	N/A
10503200	Chd7	N/A	N/A
10503202	Chd7	N/A	N/A
10503204	Chd7	N/A	N/A
10503206	Chd7	N/A	N/A
10503208	Chd7	N/A	N/A
10503210	Chd7	N/A	N/A
10503212	Chd7	N/A	N/A
10503214	Chd7	N/A	N/A
10503216	Chd7	N/A	N/A
10503218	Chd7	N/A	N/A
10503222	Chd7	N/A	N/A
10503232	---	N/A	N/A
10503234	Asph /// Clvs1	N/A	N/A
10503244	---	N/A	N/A
10503251	2610301B20Rik	N/A	N/A
10503257	---	N/A	N/A
10503259	Trp53inp1	N/A	N/A
10503281	Gxylt1	N/A	N/A
10503303	1110037F02Rik	N/A	N/A
10503305	1110037F02Rik	N/A	N/A
10503359	C430048L16Rik	N/A	N/A
10503361	---	N/A	N/A
10503363	Rbm12b	N/A	N/A
10503370	Hnrnpa3 /// Gm6793	N/A	N/A
10503374	---	N/A	N/A
10503376	Gm11818	N/A	N/A
10503382	Runx1t1	N/A	N/A
10503399	Myl6 /// Gm8894	N/A	N/A
10503401	Tmem55a	N/A	N/A
10503410	Tmem64	N/A	N/A
10503416	Calb1	N/A	N/A
10503448	Mmp16	N/A	N/A
10503508	Ggh	N/A	N/A
10503520	Ttpa	N/A	N/A
10503523	Ggh	N/A	N/A
10503534	Ccnc	N/A	N/A
10503551	Usp45	N/A	N/A
10503570	Sfrs18	N/A	N/A
10503584	Coq3	N/A	N/A
10503593	6230409E13Rik	N/A	N/A
10503613	---	N/A	N/A
10503615	---	N/A	N/A
10503643	Ndufaf4	N/A	N/A
10503647	Gpr63	N/A	N/A
10503650	---	N/A	N/A
10503654	---	N/A	N/A
10503656	---	N/A	N/A
10503659	Epha7	N/A	N/A
10503680	Map3k7	N/A	N/A
10503695	Bach2	N/A	N/A
10503709	D130062J21Rik	N/A	N/A
10503711	Casp8ap2	N/A	N/A
10503723	Mdn1	N/A	N/A
10503828	Lyrm2	N/A	N/A
10503833	Rplp1	N/A	N/A
10503835	Rragd	N/A	N/A
10503845	Ube2j1	N/A	N/A
10503876	Srsf13b	N/A	N/A
10503882	Rngtt	N/A	N/A
10503902	Cnr1	N/A	N/A
10503911	Polr1d	N/A	N/A
10503915	Chchd2	N/A	N/A
10503917	Akirin2	N/A	N/A

10503952	Ifnk	N/A	N/A
10503955	---	N/A	N/A
10503963	---	N/A	N/A
10503966	Aco1	N/A	N/A
10503989	Ndufb6	N/A	N/A
10503995	Dnaja1	N/A	N/A
10504000	Dnaja1 /// Mir207	N/A	N/A
10504008	Chmp5	N/A	N/A
10504017	Nfx1	N/A	N/A
10504047	Ube2r2	N/A	N/A
10504054	---	N/A	N/A
10504056	Ubap1	N/A	N/A
10504064	Nudt2	N/A	N/A
10504072	Dnaic1	N/A	N/A
10504094	Galt	N/A	N/A
10504106	Il11ra2 /// Gm2002 /// Il11ra1 /// Gm13305	N/A	N/A
10504121	Gm3893	N/A	N/A
10504123	Gm3893	N/A	N/A
10504125	Gm3893	N/A	N/A
10504127	Ccl21c	N/A	N/A
10504132	Ccl19 /// Gm13309 /// Gm2442 /// LOC100043921	N/A	N/A
10504137	4933409K07Rik /// Gm10590	N/A	N/A
10504139	Gm3893 /// 4933409K07Rik /// 4930578G10Rik /// Gm5859 /// Gm7819 /// Gm10590	N/A	N/A
10504148	Gm3893 /// 4933409K07Rik /// Gm5859 /// Gm7819 /// Gm10590 /// LOC100503421	N/A	N/A
10504154	Ccl21c	N/A	N/A
10504159	Ccl19 /// Gm13309 /// Gm2442 /// LOC100043921	N/A	N/A
10504164	Ccl27a /// Gm13306	N/A	N/A
10504169	4933409K07Rik /// Gm7819 /// Gm3893 /// Gm10590 /// LOC100503421	N/A	N/A
10504172	4933409K07Rik /// 4930578G10Rik /// Gm7819 /// Gm3893 /// Gm10590	N/A	N/A
10504178	Gm3893 /// 4933409K07Rik /// Gm7819 /// Gm10590	N/A	N/A
10504183	Ccl21c	N/A	N/A
10504188	Ccl19 /// Gm13309 /// Gm2442 /// LOC100043921	N/A	N/A
10504194	Ccl27a /// Gm13306	N/A	N/A
10504199	---	N/A	N/A
10504201	4933409K07Rik /// Gm10590	N/A	N/A
10504218	Dnajb5	N/A	N/A
10504316	Tesk1	N/A	N/A
10504329	Ccdc107	N/A	N/A
10504349	Creb3 /// Gba2	N/A	N/A
10504373	Gm12472	N/A	N/A
10504398	Serf2	N/A	N/A
10504402	Tmem8b	N/A	N/A
10504417	Hrct1	N/A	N/A
10504450	Glipr2	N/A	N/A
10504456	Ccin	N/A	N/A
10504458	Clta	N/A	N/A
10504466	---	N/A	N/A
10504499	Zcchc7	N/A	N/A
10504504	Grhpr	N/A	N/A
10504514	1700055D18Rik	N/A	N/A
10504534	Frmpd1	N/A	N/A
10504551	Rg9mtd3	N/A	N/A
10504562	Exosc3	N/A	N/A
10504564	Dcaf10	N/A	N/A
10504582	1300002K09Rik	N/A	N/A
10504606	Aldh1b1	N/A	N/A
10504609	---	N/A	N/A
10504611	E230008N13Rik	N/A	N/A
10504615	E230008N13Rik	N/A	N/A
10504617	E230008N13Rik	N/A	N/A
10504619	E230008N13Rik	N/A	N/A
10504621	E230008N13Rik	N/A	N/A
10504630	E230008N13Rik	N/A	N/A
10504632	E230008N13Rik	N/A	N/A
10504634	E230008N13Rik	N/A	N/A
10504636	E230008N13Rik	N/A	N/A
10504638	E230008N13Rik	N/A	N/A
10504640	E230008N13Rik	N/A	N/A
10504642	E230008N13Rik	N/A	N/A
10504644	E230008N13Rik	N/A	N/A
10504646	E230008N13Rik	N/A	N/A
10504648	E230008N13Rik	N/A	N/A
10504650	E230008N13Rik	N/A	N/A
10504652	E230008N13Rik	N/A	N/A
10504654	E230008N13Rik	N/A	N/A
10504656	E230008N13Rik	N/A	N/A
10504658	E230008N13Rik	N/A	N/A
10504660	E230008N13Rik	N/A	N/A
10504662	E230008N13Rik	N/A	N/A
10504664	E230008N13Rik	N/A	N/A
10504666	E230008N13Rik	N/A	N/A
10504668	E230008N13Rik	N/A	N/A
10504670	E230008N13Rik	N/A	N/A
10504672	Tdrd7	N/A	N/A

10504690	---	N/A	N/A
10504692	Tmod1 /// Tstd2	N/A	N/A
10504703	Ncbp1	N/A	N/A
10504728	Foxe1	N/A	N/A
10504751	---	N/A	N/A
10504753	LOC641050	N/A	N/A
10504755	---	N/A	N/A
10504757	BC005685	N/A	N/A
10504759	---	N/A	N/A
10504761	LOC641050	N/A	N/A
10504817	Tgfbr1	N/A	N/A
10504849	Stx17	N/A	N/A
10504865	Invs	N/A	N/A
10504891	Tmeff1	N/A	N/A
10504902	Murc	N/A	N/A
10504905	E130309F12Rik	N/A	N/A
10504912	---	N/A	N/A
10504914	---	N/A	N/A
10504916	---	N/A	N/A
10504926	Rnf20	N/A	N/A
10504955	---	N/A	N/A
10504986	---	N/A	N/A
10504988	Olfr275	N/A	N/A
10504990	Olfr270	N/A	N/A
10505028	Slc44a1	N/A	N/A
10505030	Fsd1l	N/A	N/A
10505044	Fktn	N/A	N/A
10505064	Tmem38b	N/A	N/A
10505071	Tmem38b	N/A	N/A
10505073	Zfp462	N/A	N/A
10505090	Rpl29	N/A	N/A
10505092	Rad23b	N/A	N/A
10505105	Actl7b	N/A	N/A
10505107	Actl7a	N/A	N/A
10505109	BC026590	N/A	N/A
10505120	Palm2 /// Akap2	N/A	N/A
10505143	Palm2 /// Akap2	N/A	N/A
10505163	Zkscan16	N/A	N/A
10505187	Ugcg	N/A	N/A
10505198	Rod1	N/A	N/A
10505200	Hsdl2	N/A	N/A
10505224	Snx30	N/A	N/A
10505237	---	N/A	N/A
10505240	---	N/A	N/A
10505246	---	N/A	N/A
10505249	---	N/A	N/A
10505252	---	N/A	N/A
10505254	---	N/A	N/A
10505258	---	N/A	N/A
10505261	---	N/A	N/A
10505266	---	N/A	N/A
10505268	---	N/A	N/A
10505276	Slc31a1	N/A	N/A
10505282	Prpf4	N/A	N/A
10505436	Mir455	N/A	N/A
10505438	Orm1	N/A	N/A
10505445	Orm3	N/A	N/A
10505451	Orm2	N/A	N/A
10505457	---	N/A	N/A
10505461	Atp6v1g1	N/A	N/A
10505489	Pappa	N/A	N/A
10505515	---	N/A	N/A
10505526	Rps2 /// Rps2-ps6	N/A	N/A
10505528	---	N/A	N/A
10505532	---	N/A	N/A
10505568	Frmd3	N/A	N/A
10505587	Kdm4c	N/A	N/A
10505612	---	N/A	N/A
10505623	D4Bwg0951e	N/A	N/A
10505630	Snapc3	N/A	N/A
10505705	Sh3gl2	N/A	N/A
10505747	Rraga	N/A	N/A
10505779	Acer2	N/A	N/A
10505788	Acer2	N/A	N/A
10505791	BC057079	N/A	N/A
10505837	Mir491	N/A	N/A
10505839	Klhl9	N/A	N/A
10505841	Gm13290 /// Gm13271 /// Gm13275	N/A	N/A
10505843	Gm13271 /// Gm13275	N/A	N/A
10505845	Gm13290	N/A	N/A
10505848	Gm13291	N/A	N/A
10505852	Gm13292	N/A	N/A
10505856	Gm13293	N/A	N/A
10505858	Gm13294	N/A	N/A

10505862	Gm13295	N/A	N/A
10505864	Gm13296	N/A	N/A
10505866	Gm13297	N/A	N/A
10505868	Gm13298	N/A	N/A
10505870	Gm13299	N/A	N/A
10505872	Gm13300	N/A	N/A
10505874	Gm13301	N/A	N/A
10505879	Ifna7	N/A	N/A
10505884	Ifna6	N/A	N/A
10505888	Ifna5	N/A	N/A
10505890	Ifna4	N/A	N/A
10505892	Ifna12	N/A	N/A
10505917	---	N/A	N/A
10505922	---	N/A	N/A
10505925	---	N/A	N/A
10505927	---	N/A	N/A
10505954	Tek	N/A	N/A
10505978	---	N/A	N/A
10505980	Fggy	N/A	N/A
10505982	Fggy	N/A	N/A
10505994	Fggy	N/A	N/A
10505996	Fggy	N/A	N/A
10505998	Fggy	N/A	N/A
10506004	Hook1	N/A	N/A
10506029	---	N/A	N/A
10506031	Nfia	N/A	N/A
10506050	Nfia	N/A	N/A
10506052	---	N/A	N/A
10506058	Inadl	N/A	N/A
10506108	---	N/A	N/A
10506125	Angptl3	N/A	N/A
10506134	Atg4c	N/A	N/A
10506146	Gm10305	N/A	N/A
10506148	Gm12689	N/A	N/A
10506186	---	N/A	N/A
10506188	Pgm2	N/A	N/A
10506201	Ror1	N/A	N/A
10506254	Raver2	N/A	N/A
10506267	---	N/A	N/A
10506269	Ak4	N/A	N/A
10506274	Dnajc6	N/A	N/A
10506296	---	N/A	N/A
10506298	Leprot	N/A	N/A
10506330	---	N/A	N/A
10506335	Pde4b	N/A	N/A
10506360	Sgip1	N/A	N/A
10506397	Mier1	N/A	N/A
10506415	Oma1	N/A	N/A
10506424	Actg1	N/A	N/A
10506427	---	N/A	N/A
10506431	Gm10304	N/A	N/A
10506433	Dab1	N/A	N/A
10506452	AY512949	N/A	N/A
10506454	C8b	N/A	N/A
10506488	Ppap2b	N/A	N/A
10506496	---	N/A	N/A
10506500	Usp24	N/A	N/A
10506569	Usp24	N/A	N/A
10506571	Dhcr24	N/A	N/A
10506603	Ssbp3	N/A	N/A
10506643	Tmem59	N/A	N/A
10506658	Hspb11	N/A	N/A
10506668	Yipf1	N/A	N/A
10506680	Tmem48	N/A	N/A
10506714	Lrp8	N/A	N/A
10506736	Magoh	N/A	N/A
10506767	Echdc2	N/A	N/A
10506781	2010305A19Rik	N/A	N/A
10506786	Zcchc11	N/A	N/A
10506820	---	N/A	N/A
10506870	Txndc12	N/A	N/A
10506880	Kti12	N/A	N/A
10506893	Nrd1 /// Osbpl9	N/A	N/A
10506939	Eps15	N/A	N/A
10506989	Faf1	N/A	N/A
10507015	Agbl4	N/A	N/A
10507032	Bend5	N/A	N/A
10507099	Gm12824	N/A	N/A
10507101	Gm12824	N/A	N/A
10507110	---	N/A	N/A
10507137	Pdzk1ip1	N/A	N/A
10507143	Cyp4a12a	N/A	N/A
10507152	Cyp4a12b	N/A	N/A
10507160	---	N/A	N/A

10507171	Cyp4a31	N/A	N/A
10507177	Cyp4a10 /// Cyp4a31 /// Cyp4a32	N/A	N/A
10507190	4732418C07Rik	N/A	N/A
10507203	Atpaf1	N/A	N/A
10507213	Mobkl2c	N/A	N/A
10507218	Mknk1	N/A	N/A
10507231	Kncn	N/A	N/A
10507236	---	N/A	N/A
10507238	Lrrc41	N/A	N/A
10507250	Pomgnt1	N/A	N/A
10507273	Pik3r3	N/A	N/A
10507284	---	N/A	N/A
10507299	Gpbp1l1	N/A	N/A
10507328	Prdx1	N/A	N/A
10507347	Tesk2 /// Toe1	N/A	N/A
10507379	Zswim5	N/A	N/A
10507394	Hectd3	N/A	N/A
10507418	Eif2b3	N/A	N/A
10507431	---	N/A	N/A
10507471	---	N/A	N/A
10507473	---	N/A	N/A
10507475	---	N/A	N/A
10507484	Eri3	N/A	N/A
10507500	Slc6a9 /// Ccdc24	N/A	N/A
10507518	---	N/A	N/A
10507520	Hyi	N/A	N/A
10507529	Med8	N/A	N/A
10507539	Elovl1	N/A	N/A
10507555	---	N/A	N/A
10507557	Ebna1bp2 /// D4Ertd617e	N/A	N/A
10507567	Olfr62	N/A	N/A
10507574	Olfr1339	N/A	N/A
10507576	Olfr1331	N/A	N/A
10507578	Olfr1330	N/A	N/A
10507594	Slc2a1	N/A	N/A
10507606	Ccdc23	N/A	N/A
10507655	---	N/A	N/A
10507657	Foxj3	N/A	N/A
10507671	Guca2a	N/A	N/A
10507675	Hivep3	N/A	N/A
10507677	Hivep3	N/A	N/A
10507699	Scmh1	N/A	N/A
10507731	Rims3	N/A	N/A
10507773	---	N/A	N/A
10507775	---	N/A	N/A
10507784	Ppt1	N/A	N/A
10507804	Trit1	N/A	N/A
10507824	Oxct2b /// Oxct2a	N/A	N/A
10507826	Hpcal4	N/A	N/A
10507833	Nt5c1a	N/A	N/A
10507840	Heyl	N/A	N/A
10507851	Pabpc4	N/A	N/A
10507870	---	N/A	N/A
10507872	---	N/A	N/A
10507885	Mycbp	N/A	N/A
10507903	---	N/A	N/A
10507905	Pou3f1	N/A	N/A
10507914	Sf3a3	N/A	N/A
10507931	Mir697	N/A	N/A
10507961	Mtf1	N/A	N/A
10507983	Epha10	N/A	N/A
10507990	Epha10	N/A	N/A
10508012	Rspo1	N/A	N/A
10508019	Gnl2	N/A	N/A
10508042	Meaf6	N/A	N/A
10508052	Grik3	N/A	N/A
10508069	Ftl2 /// Ftl1 /// Mir692-1	N/A	N/A
10508089	Mrps15	N/A	N/A
10508099	Oscp1	N/A	N/A
10508112	Lsm10	N/A	N/A
10508115	Stk40	N/A	N/A
10508135	Trappc3	N/A	N/A
10508149	Tekt2	N/A	N/A
10508178	5730409E04Rik	N/A	N/A
10508190	AU040320	N/A	N/A
10508217	Sfpq	N/A	N/A
10508228	Zmym6 /// Gm12942	N/A	N/A
10508247	---	N/A	N/A
10508249	Gm12942	N/A	N/A
10508253	Dlgap3	N/A	N/A
10508269	BC003266	N/A	N/A
10508351	Phc2	N/A	N/A
10508376	Trim62	N/A	N/A
10508382	Ak2	N/A	N/A

10508392	Rnf19b	N/A	N/A
10508412	Fndc5	N/A	N/A
10508420	Yars	N/A	N/A
10508452	---	N/A	N/A
10508454	Bsdc1	N/A	N/A
10508465	Marcksl1	N/A	N/A
10508468	2510006D16Rik	N/A	N/A
10508477	---	N/A	N/A
10508479	Ptp4a2	N/A	N/A
10508486	LOC100505143	N/A	N/A
10508488	---	N/A	N/A
10508490	Srsf5	N/A	N/A
10508500	Bai2	N/A	N/A
10508608	Pef1	N/A	N/A
10508614	Fabp3	N/A	N/A
10508645	Snord85	N/A	N/A
10508647	---	N/A	N/A
10508649	---	N/A	N/A
10508663	Laptm5	N/A	N/A
10508686	Mecr	N/A	N/A
10508697	Srsf4	N/A	N/A
10508707	Tmem200b	N/A	N/A
10508709	Gm10300	N/A	N/A
10508711	Taf12	N/A	N/A
10508719	Snora16a	N/A	N/A
10508721	Snora44	N/A	N/A
10508723	Snora61	N/A	N/A
10508725	---	N/A	N/A
10508727	Dnajc8	N/A	N/A
10508734	Ptafr	N/A	N/A
10508737	Eya3	N/A	N/A
10508770	Gm13033	N/A	N/A
10508788	Ahdc1	N/A	N/A
10508797	Gm10151	N/A	N/A
10508800	Gm3579	N/A	N/A
10508805	Wasf2	N/A	N/A
10508860	Slc9a1	N/A	N/A
10508879	Fam46b	N/A	N/A
10508883	1810019J16Rik	N/A	N/A
10508887	Nr0b2	N/A	N/A
10508901	Gpn2	N/A	N/A
10508907	Lin28a	N/A	N/A
10508972	Gm5589	N/A	N/A
10508974	Pafah2	N/A	N/A
10508986	Stmn1	N/A	N/A
10508992	Paqr7	N/A	N/A
10508996	2610002D18Rik	N/A	N/A
10509000	---	N/A	N/A
10509014	D4Wsu53e	N/A	N/A
10509023	Syf2	N/A	N/A
10509063	Il22ra1	N/A	N/A
10509071	9130219A07Rik	N/A	N/A
10509127	Fuca1	N/A	N/A
10509137	Hmgcl	N/A	N/A
10509161	---	N/A	N/A
10509163	Id3	N/A	N/A
10509204	Tcea3	N/A	N/A
10509228	Hnrnpr	N/A	N/A
10509244	6030445D17Rik	N/A	N/A
10509246	Luzp1	N/A	N/A
10509253	4930549C01Rik	N/A	N/A
10509273	Wnt4	N/A	N/A
10509275	Rpl31 /// Gm16382	N/A	N/A
10509379	Usp48	N/A	N/A
10509441	Ece1	N/A	N/A
10509463	Eif4g3	N/A	N/A
10509500	Hp1bp3	N/A	N/A
10509542	Ddost	N/A	N/A
10509555	---	N/A	N/A
10509557	AB041806	N/A	N/A
10509560	Rpl38	N/A	N/A
10509562	Mul1	N/A	N/A
10509568	Camk2n1	N/A	N/A
10509571	Pla2g2c /// Ubxn10	N/A	N/A
10509596	Rnf186	N/A	N/A
10509601	Tmco4	N/A	N/A
10509620	Capzb	N/A	N/A
10509635	Akr7a5	N/A	N/A
10509645	C230096C10Rik /// Ubr4	N/A	N/A
10509790	Aldh4a1	N/A	N/A
10509820	Rcc2	N/A	N/A
10509856	---	N/A	N/A
10509868	Atp13a2	N/A	N/A
10509901	Mfap2	N/A	N/A

10509927	D4Ertd22e /// 4921514A10Rik	N/A	N/A
10509930	Fbxo42	N/A	N/A
10509941	6330545A04Rik	N/A	N/A
10509947	Arhgef19	N/A	N/A
10509965	Epha2	N/A	N/A
10509985	Fam131c	N/A	N/A
10510019	B330016D10Rik	N/A	N/A
10510022	Tmem82 /// AI507597	N/A	N/A
10510025	Agmat	N/A	N/A
10510047	Gm10565	N/A	N/A
10510061	Pramef8	N/A	N/A
10510068	BC080695 /// Gm13057 /// Gm13109 /// Gm13043 /// Gm13119 /// Gm13040 /// Gm13101	N/A	N/A
10510072	BC080695 /// Gm13057 /// Gm13101 /// Gm13109 /// Gm13043 /// Gm13119 /// Gm13040	N/A	N/A
10510117	---	N/A	N/A
10510125	Mcrs1 /// LOC100505102	N/A	N/A
10510127	Gm13177	N/A	N/A
10510129	Dhrs3	N/A	N/A
10510142	Smarca5 /// Vmn2r-ps14	N/A	N/A
10510162	Ppp2r5a	N/A	N/A
10510165	Gm13238	N/A	N/A
10510167	Gm13235	N/A	N/A
10510170	Gm13235	N/A	N/A
10510172	Hmgb2	N/A	N/A
10510176	Smarca5	N/A	N/A
10510194	Hmgb1	N/A	N/A
10510197	Ppp2r5a	N/A	N/A
10510201	Rex2 /// Gm13138 /// Gm13152 /// Gm13235 /// Zfp600 /// Gm13242 /// Gm13151 /// 2610305D13Rik	N/A	N/A
10510212	Hmgb1	N/A	N/A
10510215	Gm13139	N/A	N/A
10510219	Cdv3 /// Gm13238	N/A	N/A
10510221	Gm3579	N/A	N/A
10510225	Gm13235	N/A	N/A
10510227	Hmgb1	N/A	N/A
10510239	Gm16503	N/A	N/A
10510252	---	N/A	N/A
10510254	Fv1	N/A	N/A
10510256	2510039O18Rik	N/A	N/A
10510305	Mtor	N/A	N/A
10510365	Exosc10	N/A	N/A
10510391	Srm	N/A	N/A
10510399	Masp2 /// Tardbp	N/A	N/A
10510445	---	N/A	N/A
10510447	Casz1	N/A	N/A
10510452	Dffa	N/A	N/A
10510462	Trmt112	N/A	N/A
10510464	Lzic	N/A	N/A
10510482	Clstn1	N/A	N/A
10510505	---	N/A	N/A
10510507	Mir34a	N/A	N/A
10510546	Eno1	N/A	N/A
10510552	Rere	N/A	N/A
10510574	Errfi1	N/A	N/A
10510592	---	N/A	N/A
10510602	9230110K08Rik	N/A	N/A
10510604	Dnajc11	N/A	N/A
10510624	Klhl21	N/A	N/A
10510629	Nol9	N/A	N/A
10510687	Acot7	N/A	N/A
10510700	Gpr153	N/A	N/A
10510720	Rpl22	N/A	N/A
10510725	Chd5	N/A	N/A
10510809	A430005L14Rik	N/A	N/A
10510814	BC046331	N/A	N/A
10510836	Lrrc47 /// 1190007F08Rik	N/A	N/A
10510844	Wdr8	N/A	N/A
10510859	Megf6	N/A	N/A
10510861	Megf6	N/A	N/A
10510872	---	N/A	N/A
10510874	Megf6	N/A	N/A
10510876	Megf6	N/A	N/A
10510878	Megf6	N/A	N/A
10510880	Megf6	N/A	N/A
10510882	Megf6	N/A	N/A
10510884	Megf6	N/A	N/A
10510908	---	N/A	N/A
10510910	Prdm16	N/A	N/A
10510957	Pank4	N/A	N/A
10510980	---	N/A	N/A
10511007	2610002J02Rik	N/A	N/A
10511014	---	N/A	N/A
10511017	2010015L04Rik	N/A	N/A
10511069	Gnb1	N/A	N/A
10511084	Nadk	N/A	N/A
10511099	Slc35e2	N/A	N/A

10511113	Cdk11b	N/A	N/A
10511136	B930041F14Rik	N/A	N/A
10511139	Ssu72	N/A	N/A
10511146	2610204G22Rik	N/A	N/A
10511149	Mrpl20	N/A	N/A
10511175	Aurkaip1	N/A	N/A
10511180	Mxra8	N/A	N/A
10511190	Dvl1	N/A	N/A
10511207	Cpsf3l /// Pusl1	N/A	N/A
10511226	Acap3	N/A	N/A
10511252	Ube2j2	N/A	N/A
10511269	Sdf4	N/A	N/A
10511290	Tnfrsf18	N/A	N/A
10511298	9430015G10Rik	N/A	N/A
10511309	9930005F22Rik /// LOC100045778	N/A	N/A
10511311	Tmem68	N/A	N/A
10511325	Rps20	N/A	N/A
10511333	Plag1	N/A	N/A
10511342	Uba52 /// Gm5239 /// Gm7866	N/A	N/A
10511363	Penk	N/A	N/A
10511366	---	N/A	N/A
10511375	Cyp7a1	N/A	N/A
10511382	Nsmaf	N/A	N/A
10511414	---	N/A	N/A
10511416	Tox	N/A	N/A
10511429	Car8	N/A	N/A
10511442	---	N/A	N/A
10511444	Gm11810	N/A	N/A
10511484	---	N/A	N/A
10511490	---	N/A	N/A
10511496	---	N/A	N/A
10511498	Plekhf2	N/A	N/A
10511510	Ints8	N/A	N/A
10511541	Dpy19l4	N/A	N/A
10511580	Pdp1	N/A	N/A
10511586	Gm10604	N/A	N/A
10511588	Tmem67	N/A	N/A
10511617	Fam92a	N/A	N/A
10511629	Rpl26 /// Gm10136 /// Gm15772	N/A	N/A
10511661	Otud6b	N/A	N/A
10511665	Necab1	N/A	N/A
10511679	Decr1	N/A	N/A
10511692	Nbn	N/A	N/A
10511694	Osgin2	N/A	N/A
10511719	---	N/A	N/A
10511721	---	N/A	N/A
10511723	---	N/A	N/A
10511725	Cyb5r4	N/A	N/A
10511739	Cpne3	N/A	N/A
10511779	Atp6v0d2	N/A	N/A
10511789	Nkain3	N/A	N/A
10511803	2610029I01Rik	N/A	N/A
10511808	Sfrs18 /// 4930528A17Rik	N/A	N/A
10511810	Pou3f2	N/A	N/A
10511812	Pou3f2	N/A	N/A
10511814	---	N/A	N/A
10511817	Klhl32	N/A	N/A
10511819	Klhl32	N/A	N/A
10511843	1810074P20Rik	N/A	N/A
10511865	Ptges3	N/A	N/A
10511868	---	N/A	N/A
10511870	Fut9	N/A	N/A
10511877	---	N/A	N/A
10511879	---	N/A	N/A
10511881	Manea	N/A	N/A
10511886	Bhmt	N/A	N/A
10511888	---	N/A	N/A
10511890	---	N/A	N/A
10511892	---	N/A	N/A
10511894	---	N/A	N/A
10511896	BC024582	N/A	N/A
10511950	---	N/A	N/A
10511952	Orc3	N/A	N/A
10511975	Slc35a1	N/A	N/A
10511984	1700003M02Rik	N/A	N/A
10512011	Zfp292	N/A	N/A
10512022	Mobkl2b	N/A	N/A
10512024	Mobkl2b	N/A	N/A
10512030	3110043O21Rik	N/A	N/A
10512044	Lingo2	N/A	N/A
10512057	---	N/A	N/A
10512059	Lingo2	N/A	N/A
10512061	Taf9	N/A	N/A
10512063	---	N/A	N/A

10512065	---	N/A	N/A
10512067	Ddx58	N/A	N/A
10512088	Topors	N/A	N/A
10512093	Ndufb6	N/A	N/A
10512098	Aptx	N/A	N/A
10512111	Smu1	N/A	N/A
10512125	Psenen	N/A	N/A
10512129	B4galt1	N/A	N/A
10512136	Bag1	N/A	N/A
10512165	Nol6	N/A	N/A
10512195	Ubap2	N/A	N/A
10512226	Dcaf12	N/A	N/A
10512254	1110017D15Rik	N/A	N/A
10512265	2310028H24Rik	N/A	N/A
10512274	Enho	N/A	N/A
10512279	Cntfr	N/A	N/A
10512291	Dctn3	N/A	N/A
10512308	Sigmar1	N/A	N/A
10512315	Ccl27a /// Gm13306	N/A	N/A
10512322	Ccl19 /// Gm13309 /// Gm2442 /// LOC100043921	N/A	N/A
10512327	4933409K07Rik /// Gm7819 /// Gm13308 /// Gm3893 /// Ccl27a /// Gm10590	N/A	N/A
10512332	4930466F19Rik	N/A	N/A
10512350	4933409K07Rik /// Gm10590	N/A	N/A
10512352	4933409K07Rik /// Gm10590	N/A	N/A
10512354	4930466F19Rik	N/A	N/A
10512372	Ccl19 /// Gm13309 /// Gm2442 /// LOC100043921	N/A	N/A
10512377	Ccl21c /// Gm10591 /// Ccl21b /// Gm13304 /// Ccl21a /// Gm1987	N/A	N/A
10512384	BC049635	N/A	N/A
10512391	Vcp	N/A	N/A
10512431	Pigo	N/A	N/A
10512443	Stoml2	N/A	N/A
10512463	LOC280487	N/A	N/A
10512487	Rmrp	N/A	N/A
10512514	Tln1	N/A	N/A
10512574	Gba2	N/A	N/A
10512594	Rgp1 /// Msmp	N/A	N/A
10512626	Olfr71	N/A	N/A
10512628	Olfr159	N/A	N/A
10512630	Olfr155	N/A	N/A
10512637	Olfr157	N/A	N/A
10512640	Gne	N/A	N/A
10512653	---	N/A	N/A
10512655	Rnf38	N/A	N/A
10512680	Aurkc	N/A	N/A
10512688	Fbxo10	N/A	N/A
10512704	Exosc3	N/A	N/A
10512714	Shb	N/A	N/A
10512728	Tstd2 /// Tmod1	N/A	N/A
10512739	Xpa /// Ncbp1	N/A	N/A
10512766	Trim14	N/A	N/A
10512807	Gabbr2	N/A	N/A
10512827	Gm568	N/A	N/A
10512830	Anks6	N/A	N/A
10512847	Alg2	N/A	N/A
10512851	Erp44	N/A	N/A
10512867	Tex10 /// Invs	N/A	N/A
10512884	---	N/A	N/A
10512892	Acnat1	N/A	N/A
10512901	Mrpl50	N/A	N/A
10512904	Aldob	N/A	N/A
10512919	Grin3a	N/A	N/A
10512937	Rnu1b1 /// Rnu1b6 /// Rnu1b2	N/A	N/A
10512939	2610030H06Rik	N/A	N/A
10512945	Olfr272	N/A	N/A
10512947	---	N/A	N/A
10512949	Abca1	N/A	N/A
10513002	Gm12471	N/A	N/A
10513004	Gm10588	N/A	N/A
10513006	---	N/A	N/A
10513020	Ikbkap	N/A	N/A
10513061	Ctnnal1	N/A	N/A
10513082	D730040F13Rik	N/A	N/A
10513101	Mir32	N/A	N/A
10513103	6430704M03Rik	N/A	N/A
10513139	Ptpn3	N/A	N/A
10513141	Ptpn3	N/A	N/A
10513143	Ptpn3	N/A	N/A
10513145	Ptpn3	N/A	N/A
10513152	Ptpn3	N/A	N/A
10513154	Ptpn3	N/A	N/A
10513156	Ptpn3	N/A	N/A
10513158	Ptpn3	N/A	N/A
10513160	Ptpn3	N/A	N/A
10513162	Ptpn3	N/A	N/A

10513164	Ptpn3	N/A	N/A
10513181	Gapdh /// Gm16374 /// Gm2606 /// Gm4609 /// Gm3200 /// Gm2451 /// Gm10293	N/A	N/A
10513186	Gm12538	N/A	N/A
10513188	---	N/A	N/A
10513190	D630039A03Rik	N/A	N/A
10513266	Olfr267	N/A	N/A
10513268	AI314180	N/A	N/A
10513360	---	N/A	N/A
10513362	Susd1	N/A	N/A
10513381	Rod1	N/A	N/A
10513404	---	N/A	N/A
10513420	Mup7	N/A	N/A
10513428	Mup7	N/A	N/A
10513437	Mup1	N/A	N/A
10513455	Mup7	N/A	N/A
10513467	Mup3 /	N/A	N/A
10513472	Mup7	N/A	N/A
10513497	Mup1	N/A	N/A
10513504	Mup1	N/A	N/A
10513512	up1 /// Mup7 /// Mup10 /// Mup19 /// Mup2 /// LOC100048884 /// Mup9 /// LOC100048885 /// Mup11 /// Mup12 /// Mup	N/A	N/A
10513521	Mup20	N/A	N/A
10513529	Mup3	N/A	N/A
10513536	---	N/A	N/A
10513538	Mup21	N/A	N/A
10513544	Zfp37	N/A	N/A
10513551	Fkbp15	N/A	N/A
10513583	Cdc26	N/A	N/A
10513592	Wdr31	N/A	N/A
10513622	Pole3	N/A	N/A
10513630	Ambp	N/A	N/A
10513692	Whrn	N/A	N/A
10513737	Rpl17 /// Gm10268 /// Rpl17-ps3	N/A	N/A
10513774	---	N/A	N/A
10513776	Astn2	N/A	N/A
10513803	---	N/A	N/A
10513805	Dbc1	N/A	N/A
10513818	Stmn1	N/A	N/A
10513822	---	N/A	N/A
10513867	---	N/A	N/A
10513869	Megf9	N/A	N/A
10513878	Rps18 /// Gm10260	N/A	N/A
10513884	Tle1	N/A	N/A
10513912	Aldoart1	N/A	N/A
10513952	3110001D03Rik	N/A	N/A
10513955	---	N/A	N/A
10513957	Ptprd	N/A	N/A
10514000	Mpdz	N/A	N/A
10514047	---	N/A	N/A
10514049	Nfib	N/A	N/A
10514054	Nfib	N/A	N/A
10514072	Zdhhc21	N/A	N/A
10514128	Ttc39b	N/A	N/A
10514133	Ttc39b	N/A	N/A
10514158	Psip1	N/A	N/A
10514173	Rpl34 /// Gm6404 /// Gm10154	N/A	N/A
10514175	---	N/A	N/A
10514193	---	N/A	N/A
10514201	Haus6	N/A	N/A
10514219	Scarna8	N/A	N/A
10514233	Rps6 /// Gm16409	N/A	N/A
10514240	Slc24a2	N/A	N/A
10514255	Mllt3	N/A	N/A
10514289	Ifna12	N/A	N/A
10514296	Ifna9	N/A	N/A
10514298	Mrpl48	N/A	N/A
10514313	Gm13280	N/A	N/A
10514315	Ifna2	N/A	N/A
10514317	Ifnab	N/A	N/A
10514319	Klhl9	N/A	N/A
10514323	LOC100037413	N/A	N/A
10514325	Gm13290 /// Gm13275 /// Ifnz	N/A	N/A
10514327	Gm13290 /// Gm13275 /// Ifnz	N/A	N/A
10514329	Gm13290 /// Gm13275 /// Ifnz	N/A	N/A
10514331	Gm13290 /// Gm13275 /// Ifnz	N/A	N/A
10514333	4930553M12Rik	N/A	N/A
10514338	Mir31	N/A	N/A
10514347	Cdkn2b	N/A	N/A
10514350	---	N/A	N/A
10514352	Elavl2	N/A	N/A
10514368	Gapdh /// Gm12070 /// Gm16374 /// Gm2606 /// Gm4609 /// Gm3200 /// Gm2451 /// Gm10293	N/A	N/A
10514374	---	N/A	N/A
10514383	Larp7	N/A	N/A
10514390	---	N/A	N/A
10514392	Tusc1	N/A	N/A

10514396	---	N/A	N/A
10514398	5830433M19Rik /// Gm10306	N/A	N/A
10514405	Plaa	N/A	N/A
10514441	Mysm1	N/A	N/A
10514461	---	N/A	N/A
10514464	---	N/A	N/A
10514466	Jun	N/A	N/A
10514473	---	N/A	N/A
10514475	Hook1	N/A	N/A
10514491	Cyp2j12-ps	N/A	N/A
10514510	Cyp2j6	N/A	N/A
10514520	Cyp2j9	N/A	N/A
10514532	Cyp2j5	N/A	N/A
10514556	Gm10192	N/A	N/A
10514558	---	N/A	N/A
10514568	Tm2d1	N/A	N/A
10514576	Kank4	N/A	N/A
10514590	Dock7	N/A	N/A
10514645	---	N/A	N/A
10514647	---	N/A	N/A
10514658	Ccdc50	N/A	N/A
10514662	Gm10577	N/A	N/A
10514664	Gm10576	N/A	N/A
10514668	Jak1	N/A	N/A
10514697	Mir101a	N/A	N/A
10514706	---	N/A	N/A
10514713	Wdr78	N/A	N/A
10514732	Slc35d1	N/A	N/A
10514763	C8a	N/A	N/A
10514779	Prkaa2	N/A	N/A
10514789	---	N/A	N/A
10514791	Pcsk9	N/A	N/A
10514815	---	N/A	N/A
10514830	Ttc4	N/A	N/A
10514865	Acot11	N/A	N/A
10514884	Mrpl37	N/A	N/A
10514892	2210012G02Rik	N/A	N/A
10514896	2210012G02Rik	N/A	N/A
10514902	Lrrc42	N/A	N/A
10514912	Dio1	N/A	N/A
10514924	Tomm22	N/A	N/A
10514926	B230314M03Rik	N/A	N/A
10514933	Cpt2	N/A	N/A
10514956	Scp2	N/A	N/A
10514971	Zyg11a	N/A	N/A
10514985	Zyg11b	N/A	N/A
10515003	Fam159a	N/A	N/A
10515012	Prpf38a	N/A	N/A
10515026	Cc2d1b	N/A	N/A
10515028	Zfyve9	N/A	N/A
10515046	Btf3l4	N/A	N/A
10515049	Kti12	N/A	N/A
10515051	Osbpl9	N/A	N/A
10515086	---	N/A	N/A
10515113	Hnrnpa3	N/A	N/A
10515132	---	N/A	N/A
10515142	---	N/A	N/A
10515154	Rpl3	N/A	N/A
10515156	Minpp1	N/A	N/A
10515164	Cmpk1	N/A	N/A
10515168	Cyp4x1	N/A	N/A
10515187	Cyp4a14	N/A	N/A
10515211	4930544O15Rik	N/A	N/A
10515220	Faah	N/A	N/A
10515253	Uqcrh	N/A	N/A
10515277	1520402A15Rik /// Pomgnt1	N/A	N/A
10515280	---	N/A	N/A
10515293	Llph	N/A	N/A
10515295	Mast2	N/A	N/A
10515326	Tmem69	N/A	N/A
10515335	Ccdc17 /// C530005A16Rik	N/A	N/A
10515337	Nasp	N/A	N/A
10515363	Mmachc	N/A	N/A
10515378	Hpdl	N/A	N/A
10515383	Rpl36 /// Gm13611 /// Gm5745	N/A	N/A
10515396	Btbd19	N/A	N/A
10515399	Plk3	N/A	N/A
10515416	Rps8 /// Gm5121 /// Gm11353 /// Rps8-ps1	N/A	N/A
10515423	---	N/A	N/A
10515425	Snord38a	N/A	N/A
10515427	---	N/A	N/A
10515429	Snord55	N/A	N/A
10515452	Gm1661	N/A	N/A
10515461	Rnf220	N/A	N/A

10515481	Dmap1	N/A	N/A
10515493	Rhoa	N/A	N/A
10515519	Atp6v0b	N/A	N/A
10515528	Dph2	N/A	N/A
10515536	Ipo13	N/A	N/A
10515574	St3gal3	N/A	N/A
10515613	Ptprf	N/A	N/A
10515688	Szt2	N/A	N/A
10515690	Szt2	N/A	N/A
10515692	Szt2	N/A	N/A
10515694	Szt2	N/A	N/A
10515696	Szt2	N/A	N/A
10515698	Szt2	N/A	N/A
10515704	Szt2	N/A	N/A
10515706	Szt2	N/A	N/A
10515708	Szt2	N/A	N/A
10515710	Szt2	N/A	N/A
10515712	Szt2	N/A	N/A
10515714	Szt2	N/A	N/A
10515716	Szt2	N/A	N/A
10515729	Szt2	N/A	N/A
10515731	Szt2	N/A	N/A
10515733	Szt2	N/A	N/A
10515735	Szt2	N/A	N/A
10515737	Szt2	N/A	N/A
10515771	Tie1	N/A	N/A
10515803	Wdr65	N/A	N/A
10515822	Olfr1342	N/A	N/A
10515824	Olfr1338	N/A	N/A
10515826	Olfr1328	N/A	N/A
10515828	Olfr1328 /// Olfr1335	N/A	N/A
10515830	Olfr1333	N/A	N/A
10515832	Olfr1329	N/A	N/A
10515834	Olfr1328	N/A	N/A
10515839	---	N/A	N/A
10515844	Zfp691	N/A	N/A
10515861	4930538K18Rik	N/A	N/A
10515870	AU022252	N/A	N/A
10515878	Ybx1	N/A	N/A
10515884	Ppih /// Gm10069	N/A	N/A
10515924	Rimkla	N/A	N/A
10515930	AA415398	N/A	N/A
10515943	Ctps	N/A	N/A
10515979	Mir30c-1	N/A	N/A
10515981	Mir30e	N/A	N/A
10515983	Dem1	N/A	N/A
10515994	Smap2	N/A	N/A
10516007	Zmpste24	N/A	N/A
10516020	---	N/A	N/A
10516022	---	N/A	N/A
10516024	Tmco2	N/A	N/A
10516027	Rlf	N/A	N/A
10516051	Cap1	N/A	N/A
10516079	Oxct2b	N/A	N/A
10516101	Oxct2a	N/A	N/A
10516103	Macf1	N/A	N/A
10516209	Rps2	N/A	N/A
10516211	Ndufs5	N/A	N/A
10516219	Gm10572	N/A	N/A
10516221	---	N/A	N/A
10516225	Rragc /// 4930535I16Rik	N/A	N/A
10516227	3100002H09Rik	N/A	N/A
10516229	Utp11l	N/A	N/A
10516238	1110065P20Rik	N/A	N/A
10516241	Maneal	N/A	N/A
10516259	Dnali1	N/A	N/A
10516275	Rpl28	N/A	N/A
10516296	Thrap3	N/A	N/A
10516303	---	N/A	N/A
10516305	Mtap7d1	N/A	N/A
10516325	Adprhl2	N/A	N/A
10516335	Tekt2	N/A	N/A
10516348	Eif2c3	N/A	N/A
10516371	Eif2c1	N/A	N/A
10516393	Eif2c4	N/A	N/A
10516427	Ncdn	N/A	N/A
10516435	Zmym4	N/A	N/A
10516466	Zmym1	N/A	N/A
10516479	Rps28	N/A	N/A
10516481	Gja4	N/A	N/A
10516495	CK137956	N/A	N/A
10516507	Zscan20	N/A	N/A
10516518	Tlr12	N/A	N/A
10516520	Zfp362	N/A	N/A

10516529	Adc	N/A	N/A
10516544	Hpca	N/A	N/A
10516551	S100pbp	N/A	N/A
10516576	Rbbp4 /// Sync	N/A	N/A
10516590	Zbtb8a	N/A	N/A
10516602	Tssk3	N/A	N/A
10516605	Hdac1	N/A	N/A
10516637	Fam167b	N/A	N/A
10516640	Eif3i	N/A	N/A
10516652	Iqcc	N/A	N/A
10516666	Txlna	N/A	N/A
10516695	Tmem39b	N/A	N/A
10516706	Khdrbs1	N/A	N/A
10516721	---	N/A	N/A
10516723	Hcrtr1	N/A	N/A
10516735	Tinagl1	N/A	N/A
10516765	Serinc2	N/A	N/A
10516776	Gm10570 /// Fabp3	N/A	N/A
10516778	Zcchc17	N/A	N/A
10516823	Epb4.1	N/A	N/A
10516859	Ythdf2	N/A	N/A
10516867	Gmeb1	N/A	N/A
10516906	Snora73b	N/A	N/A
10516908	Snora73a	N/A	N/A
10516910	Phactr4	N/A	N/A
10516956	Smpdl3b	N/A	N/A
10516966	BC013712	N/A	N/A
10516982	Stx12	N/A	N/A
10517005	Gpr3	N/A	N/A
10517028	Tmem222	N/A	N/A
10517036	Wdtc1	N/A	N/A
10517053	Trnp1	N/A	N/A
10517058	1810019J16Rik	N/A	N/A
10517067	Sfn	N/A	N/A
10517070	Zdhhc18	N/A	N/A
10517083	Pigv	N/A	N/A
10517090	Arid1a	N/A	N/A
10517114	---	N/A	N/A
10517141	Hmgn2 /// Gm6724	N/A	N/A
10517147	Dhdds	N/A	N/A
10517165	Cd52	N/A	N/A
10517169	Sh3bgrl3	N/A	N/A
10517236	Zfp593	N/A	N/A
10517240	Grrp1	N/A	N/A
10517250	Extl1	N/A	N/A
10517263	Fam54b	N/A	N/A
10517274	Sepn1	N/A	N/A
10517301	Ldlrap1	N/A	N/A
10517328	Tmem50a	N/A	N/A
10517336	Clic4	N/A	N/A
10517343	---	N/A	N/A
10517345	Srrm1	N/A	N/A
10517371	---	N/A	N/A
10517373	Rcan3 /// Mir700	N/A	N/A
10517383	Nipal3	N/A	N/A
10517419	---	N/A	N/A
10517436	1110049F12Rik	N/A	N/A
10517443	Tceb3	N/A	N/A
10517463	Luzp1	N/A	N/A
10517465	Kdm1a	N/A	N/A
10517486	---	N/A	N/A
10517508	C1qb	N/A	N/A
10517513	C1qc	N/A	N/A
10517517	C1qa	N/A	N/A
10517540	Zbtb40	N/A	N/A
10517559	Cdc42	N/A	N/A
10517566	---	N/A	N/A
10517587	Alpl	N/A	N/A
10517600	Pink1	N/A	N/A
10517609	Cda	N/A	N/A
10517614	Fam43b /// AB041806	N/A	N/A
10517616	Vwa5b1	N/A	N/A
10517664	---	N/A	N/A
10517673	Htr6	N/A	N/A
10517677	Nbl1	N/A	N/A
10517682	2310028O11Rik	N/A	N/A
10517687	Gm16287	N/A	N/A
10517689	Pqlc2	N/A	N/A
10517706	Mrto4	N/A	N/A
10517727	Klhdc7a	N/A	N/A
10517731	Igsf21	N/A	N/A
10517742	Gm9867	N/A	N/A
10517892	D4Ertd22e	N/A	N/A
10517899	C630004L07Rik	N/A	N/A

10517948	Spen	N/A	N/A
10517996	Plekhm2	N/A	N/A
10518019	Ddi2 /// Rsc1a1	N/A	N/A
10518031	Dnajc16	N/A	N/A
10518075	Fhad1	N/A	N/A
10518132	Prdm2	N/A	N/A
10518145	Prdm2	N/A	N/A
10518147	Pdpn	N/A	N/A
10518163	---	N/A	N/A
10518167	Trappc2	N/A	N/A
10518207	---	N/A	N/A
10518216	Gm9944	N/A	N/A
10518224	Gm13178	N/A	N/A
10518226	Vps13d	N/A	N/A
10518228	Vps13d	N/A	N/A
10518300	Tnfrsf1b	N/A	N/A
10518327	---	N/A	N/A
10518329	Rpl28	N/A	N/A
10518331	---	N/A	N/A
10518333	---	N/A	N/A
10518335	1700029I01Rik /// Gm13251 /// Gm13139 /// Gm13151 /// Zfp534 /// Gm13235	N/A	N/A
10518341	Ppp2r5a	N/A	N/A
10518344	Cdv3 /// Gm13238	N/A	N/A
10518346	1700029I01Rik /// Gm13251 /// Gm13139 /// Gm13051	N/A	N/A
10518350	Hmgb2	N/A	N/A
10518352	Hmgb2	N/A	N/A
10518354	---	N/A	N/A
10518356	Rpl29	N/A	N/A
10518358	2610305D13Rik	N/A	N/A
10518361	Smarca5 /// Vmn2r-ps14	N/A	N/A
10518364	Rps19-ps3	N/A	N/A
10518366	2810408P10Rik	N/A	N/A
10518385	Mfn2	N/A	N/A
10518408	Plod1	N/A	N/A
10518428	Clcn6	N/A	N/A
10518453	Chchd2	N/A	N/A
10518484	Fbxo44	N/A	N/A
10518492	Ptchd2	N/A	N/A
10518520	Ubiad1	N/A	N/A
10518524	Ubiad1	N/A	N/A
10518532	Tardbp /// Masp2	N/A	N/A
10518546	Pex14	N/A	N/A
10518557	Cort	N/A	N/A
10518568	---	N/A	N/A
10518570	Pgd	N/A	N/A
10518585	Kif1b	N/A	N/A
10518642	Ube4b	N/A	N/A
10518714	Tmem201	N/A	N/A
10518726	Slc25a33	N/A	N/A
10518761	---	N/A	N/A
10518763	Slc45a1	N/A	N/A
10518774	Park7	N/A	N/A
10518805	Vamp3	N/A	N/A
10518812	Camta1	N/A	N/A
10518833	Camta1	N/A	N/A
10518835	Camta1	N/A	N/A
10518837	Camta1	N/A	N/A
10518927	Kcnab2	N/A	N/A
10518947	Ajap1	N/A	N/A
10518955	BC049688	N/A	N/A
10518967	1190007F08Rik	N/A	N/A
10519007	Tprgl	N/A	N/A
10519028	Prdm16	N/A	N/A
10519046	5930403L14Rik	N/A	N/A
10519052	2810405K02Rik	N/A	N/A
10519060	Tnfrsf14	N/A	N/A
10519096	Rer1	N/A	N/A
10519105	Ski	N/A	N/A
10519117	Prkcz	N/A	N/A
10519136	---	N/A	N/A
10519138	Gm10563	N/A	N/A
10519151	Mib2	N/A	N/A
10519177	---	N/A	N/A
10519179	1500002C15Rik	N/A	N/A
10519181	---	N/A	N/A
10519196	Vwa1	N/A	N/A
10519203	Tmem88b	N/A	N/A
10519207	Ccnl2	N/A	N/A
10519209	Mxra8 /// Gm10562	N/A	N/A
10519211	Tas1r3	N/A	N/A
10519219	Gltpd1	N/A	N/A
10519224	Pusl1	N/A	N/A
10519234	B3galt6	N/A	N/A
10519239	Gm10560	N/A	N/A

10519266	Mir200a	N/A	N/A
10519268	Mir200b	N/A	N/A
10519270	Agrn	N/A	N/A
10519321	Rpl26 /// Gm10136 /// Gm15772	N/A	N/A
10519324	Cdk6	N/A	N/A
10519333	Fam133b	N/A	N/A
10519344	---	N/A	N/A
10519346	1700109H08Rik /// C030048B08Rik	N/A	N/A
10519354	Pex1 /// C030048B08Rik	N/A	N/A
10519392	Krit1	N/A	N/A
10519420	Akap9	N/A	N/A
10519475	---	N/A	N/A
10519477	---	N/A	N/A
10519482	Gm8773	N/A	N/A
10519484	Steap2	N/A	N/A
10519486	---	N/A	N/A
10519488	Tubb2c	N/A	N/A
10519495	---	N/A	N/A
10519497	Steap4	N/A	N/A
10519504	Sri	N/A	N/A
10519514	---	N/A	N/A
10519516	Slc25a40	N/A	N/A
10519578	Abcb4	N/A	N/A
10519607	4930420K17Rik	N/A	N/A
10519612	9330182L06Rik	N/A	N/A
10519640	Gm10482	N/A	N/A
10519642	---	N/A	N/A
10519655	---	N/A	N/A
10519657	---	N/A	N/A
10519659	---	N/A	N/A
10519667	---	N/A	N/A
10519669	---	N/A	N/A
10519675	---	N/A	N/A
10519684	---	N/A	N/A
10519686	---	N/A	N/A
10519691	---	N/A	N/A
10519693	Sema3d	N/A	N/A
10519711	---	N/A	N/A
10519717	Sema3a	N/A	N/A
10519770	Pclo	N/A	N/A
10519805	Gm9758 /// Speer4e /// Speer4d /// Speer4c	N/A	N/A
10519811	Speer7-ps1 /// Speer8-ps1	N/A	N/A
10519815	Cacna2d1	N/A	N/A
10519855	Cacna2d1	N/A	N/A
10519857	Hgf	N/A	N/A
10519886	Sema3c	N/A	N/A
10519913	Magi2	N/A	N/A
10519940	---	N/A	N/A
10519942	Magi2	N/A	N/A
10519945	Tmem60	N/A	N/A
10519949	A630072M18Rik	N/A	N/A
10519983	Fgl2	N/A	N/A
10519988	Fam185a	N/A	N/A
10520010	Pmpcb	N/A	N/A
10520025	Psmc2	N/A	N/A
10520043	Lhfpl3	N/A	N/A
10520046	LOC100503803	N/A	N/A
10520048	Mll5	N/A	N/A
10520080	Rint1	N/A	N/A
10520096	Klhl7	N/A	N/A
10520109	---	N/A	N/A
10520111	Nupl2	N/A	N/A
10520121	---	N/A	N/A
10520124	Sumo2	N/A	N/A
10520187	Slc4a2	N/A	N/A
10520211	Agap3	N/A	N/A
10520232	---	N/A	N/A
10520247	1700022A21Rik	N/A	N/A
10520250	Nub1	N/A	N/A
10520268	1500035N22Rik	N/A	N/A
10520271	2900005J15Rik	N/A	N/A
10520288	Galnt11	N/A	N/A
10520301	Cct8l1	N/A	N/A
10520304	Actr3b	N/A	N/A
10520318	Dpp6	N/A	N/A
10520351	Dpp6	N/A	N/A
10520355	Htr5a	N/A	N/A
10520360	LOC100504484 /// LOC100503803	N/A	N/A
10520362	Insig1	N/A	N/A
10520371	Rbm33	N/A	N/A
10520379	Rbm33	N/A	N/A
10520388	Rbm33	N/A	N/A
10520390	Gapdh /// Gm4609 /// Gm10290 /// Gm10291	N/A	N/A
10520392	Rnf32	N/A	N/A

10520419	Ube3c	N/A	N/A
10520450	Gm10469	N/A	N/A
10520459	Fam59b	N/A	N/A
10520467	Hadhb	N/A	N/A
10520499	Gm1060	N/A	N/A
10520501	1700001C02Rik	N/A	N/A
10520506	Kcnk3	N/A	N/A
10520513	4930471M23Rik	N/A	N/A
10520521	Cenpa	N/A	N/A
10520527	Dpysl5	N/A	N/A
10520544	Mapre3	N/A	N/A
10520553	Tmem214	N/A	N/A
10520612	Khk	N/A	N/A
10520633	Tcf23	N/A	N/A
10520648	---	N/A	N/A
10520696	Gm9924	N/A	N/A
10520718	Snx17	N/A	N/A
10520734	Nrbp1	N/A	N/A
10520763	Gckr	N/A	N/A
10520782	Zfp512	N/A	N/A
10520815	Slc4a1ap	N/A	N/A
10520833	Mrpl33	N/A	N/A
10520842	Bre	N/A	N/A
10520860	---	N/A	N/A
10520862	Fosl2	N/A	N/A
10520867	---	N/A	N/A
10520940	Plb1	N/A	N/A
10520942	Plb1	N/A	N/A
10520944	Plb1	N/A	N/A
10520946	Plb1	N/A	N/A
10520948	Plb1	N/A	N/A
10520950	Pdlim1	N/A	N/A
10520952	Ppp1cb	N/A	N/A
10520965	Yes1 /// Gm10461	N/A	N/A
10520982	Depdc5	N/A	N/A
10521031	Ywhah	N/A	N/A
10521057	Maea	N/A	N/A
10521085	Gm9903	N/A	N/A
10521088	Slbp /// Gm10459	N/A	N/A
10521111	Fgfr3	N/A	N/A
10521134	Rps29	N/A	N/A
10521136	Whsc1	N/A	N/A
10521168	Nat8l	N/A	N/A
10521174	Rnf4	N/A	N/A
10521182	Fam193a	N/A	N/A
10521222	Add1	N/A	N/A
10521261	Htt	N/A	N/A
10521331	A930005I04Rik	N/A	N/A
10521337	Rgs12	N/A	N/A
10521356	Hgfac	N/A	N/A
10521383	Adra2c	N/A	N/A
10521389	---	N/A	N/A
10521391	Acox3	N/A	N/A
10521415	Ablim2	N/A	N/A
10521440	Afap1	N/A	N/A
10521459	Psapl1	N/A	N/A
10521461	Grpel1	N/A	N/A
10521467	Ccdc96	N/A	N/A
10521469	Mrfap1	N/A	N/A
10521471	Ppp2r2c	N/A	N/A
10521481	Jakmip1	N/A	N/A
10521495	Gm1043	N/A	N/A
10521498	Crmp1	N/A	N/A
10521543	Stx18	N/A	N/A
10521583	Drd5	N/A	N/A
10521585	---	N/A	N/A
10521587	Dnaja1	N/A	N/A
10521589	Cir1	N/A	N/A
10521600	---	N/A	N/A
10521602	Cpeb2	N/A	N/A
10521622	---	N/A	N/A
10521626	Cc2d2a	N/A	N/A
10521690	Ppih /// Gm10069	N/A	N/A
10521693	---	N/A	N/A
10521696	Prom1 /// Gm16401	N/A	N/A
10521698	---	N/A	N/A
10521700	Gm10208	N/A	N/A
10521702	4930431F12Rik	N/A	N/A
10521705	Clrn2	N/A	N/A
10521709	Lap3	N/A	N/A
10521725	Med28	N/A	N/A
10521755	4930449I04Rik	N/A	N/A
10521757	---	N/A	N/A
10521759	Slit2	N/A	N/A

10521796	Mir218-1	N/A	N/A
10521811	---	N/A	N/A
10521813	Nsfl1c	N/A	N/A
10521822	---	N/A	N/A
10521824	Sod3	N/A	N/A
10521830	Slc35a4	N/A	N/A
10521832	Pi4k2b	N/A	N/A
10521863	Anapc4	N/A	N/A
10521907	1810013D10Rik	N/A	N/A
10521913	Rbpj	N/A	N/A
10521927	Tbc1d19	N/A	N/A
10521964	Gm10440	N/A	N/A
10521966	Papd4	N/A	N/A
10521969	Immp1l	N/A	N/A
10521972	Pcdh7	N/A	N/A
10521979	---	N/A	N/A
10521982	Cbfa2t2	N/A	N/A
10521984	G6pd2	N/A	N/A
10521986	Dthd1	N/A	N/A
10521995	3110047P20Rik	N/A	N/A
10522004	0610040J01Rik	N/A	N/A
10522009	Pgm1	N/A	N/A
10522024	Tbc1d1	N/A	N/A
10522051	Klf3	N/A	N/A
10522060	Fam114a1	N/A	N/A
10522075	Klhl5	N/A	N/A
10522127	Klb	N/A	N/A
10522149	Ube2k	N/A	N/A
10522160	N4bp2	N/A	N/A
10522182	Rhoh	N/A	N/A
10522208	Uchl1	N/A	N/A
10522217	Limch1	N/A	N/A
10522248	---	N/A	N/A
10522250	Tmem33	N/A	N/A
10522265	Slc30a9	N/A	N/A
10522285	C330024D21Rik	N/A	N/A
10522301	Cmpk1	N/A	N/A
10522303	Guf1	N/A	N/A
10522324	Gabrb1	N/A	N/A
10522366	---	N/A	N/A
10522378	Slain2	N/A	N/A
10522388	Slc10a4	N/A	N/A
10522396	Ociad1	N/A	N/A
10522409	---	N/A	N/A
10522430	Dcun1d4	N/A	N/A
10522445	Spata18	N/A	N/A
10522462	Usp46	N/A	N/A
10522465	Snora26 /// 2700023E23Rik	N/A	N/A
10522467	Rasl11b	N/A	N/A
10522500	Gsx2	N/A	N/A
10522503	Pdgfra	N/A	N/A
10522530	Kit	N/A	N/A
10522554	Gm16367 /// D5Ertd577e /// E330014E10Rik /// Gm16427 /// Gm7792 /// Gm3106	N/A	N/A
10522558	LOC624931 /// EG665031 /// LOC625240 /// Gm7682	N/A	N/A
10522569	Gm16367 /// D5Ertd577e /// E330014E10Rik /// Gm16427 /// Gm7792 /// Gm3106	N/A	N/A
10522573	Gm16367 /// D5Ertd577e /// E330014E10Rik /// Gm16427 /// Gm7792 /// Gm3106	N/A	N/A
10522577	D5Ertd577e /// E330014E10Rik /// Gm3286	N/A	N/A
10522581	D5Ertd577e /// Gm16367 /// E330014E10Rik /// Gm16427 /// Gm7792 /// Gm3106	N/A	N/A
10522585	LOC624931 /// EG665031 /// LOC625240 /// Gm7682	N/A	N/A
10522589	Srd5a3	N/A	N/A
10522596	Tmem165	N/A	N/A
10522604	Gm7467	N/A	N/A
10522606	Exoc1	N/A	N/A
10522653	Gm10430	N/A	N/A
10522655	---	N/A	N/A
10522658	C530008M17Rik	N/A	N/A
10522661	C530008M17Rik	N/A	N/A
10522666	---	N/A	N/A
10522668	Paics	N/A	N/A
10522676	Srp72	N/A	N/A
10522712	Rest	N/A	N/A
10522742	LOC280487	N/A	N/A
10522744	Mthfr	N/A	N/A
10522749	Lphn3	N/A	N/A
10522782	Rpl7 /// Gm5045	N/A	N/A
10522784	Hmgn2	N/A	N/A
10522786	---	N/A	N/A
10522800	---	N/A	N/A
10522802	Ythdc1	N/A	N/A
10522819	Ugt2b35	N/A	N/A
10522873	Gm7337	N/A	N/A
10522902	BC051076	N/A	N/A
10522944	---	N/A	N/A
10522973	Utp3	N/A	N/A

10522976	Rufy3	N/A	N/A
10523021	Slc4a4	N/A	N/A
10523056	---	N/A	N/A
10523058	Eif5a	N/A	N/A
10523060	Gm9958	N/A	N/A
10523062	Alb	N/A	N/A
10523095	Afm	N/A	N/A
10523111	5830473C10Rik	N/A	N/A
10523151	Cxcl1	N/A	N/A
10523161	Mthfd2l	N/A	N/A
10523190	Parm1	N/A	N/A
10523195	Thap6	N/A	N/A
10523204	Cdkl2 /// Gm9793	N/A	N/A
10523206	Uso1	N/A	N/A
10523245	Fam47e	N/A	N/A
10523255	Stbd1	N/A	N/A
10523260	Shroom3	N/A	N/A
10523275	---	N/A	N/A
10523277	Ankrd56	N/A	N/A
10523279	---	N/A	N/A
10523281	Sep 11	N/A	N/A
10523312	Gm16367 /// D5Ertd577e /// E330014E10Rik /// Gm16427 /// Gm7792 /// Gm3106	N/A	N/A
10523337	LOC624931 /// EG665031 /// LOC625240 /// Gm7682	N/A	N/A
10523350	D5Ertd577e /// E330014E10Rik /// Gm3286	N/A	N/A
10523354	Gapdh /// Gm2606 /// Gm10293	N/A	N/A
10523357	---	N/A	N/A
10523359	Cxcl13	N/A	N/A
10523365	Mrpl1	N/A	N/A
10523468	Bmp2k	N/A	N/A
10523479	Slc25a5	N/A	N/A
10523481	---	N/A	N/A
10523483	Prdm8	N/A	N/A
10523511	Prkg2	N/A	N/A
10523518	Enoph1	N/A	N/A
10523529	Cops4	N/A	N/A
10523541	Mrps18c	N/A	N/A
10523577	---	N/A	N/A
10523647	Aff1	N/A	N/A
10523670	Aff1	N/A	N/A
10523672	---	N/A	N/A
10523674	Nudt9	N/A	N/A
10523717	Spp1	N/A	N/A
10523727	Pkd2	N/A	N/A
10523750	D930016D06Rik	N/A	N/A
10523752	Txlng	N/A	N/A
10523754	---	N/A	N/A
10523756	---	N/A	N/A
10523758	Lrrc8b	N/A	N/A
10523766	Lrrc8c	N/A	N/A
10523785	Zfp326	N/A	N/A
10523800	---	N/A	N/A
10523843	---	N/A	N/A
10523853	Lpcat2b	N/A	N/A
10523856	---	N/A	N/A
10523865	1700028K03Rik /// A830010M20Rik	N/A	N/A
10523880	Rpap2	N/A	N/A
10523891	1700013N18Rik	N/A	N/A
10523901	---	N/A	N/A
10523903	---	N/A	N/A
10523905	Mtf2	N/A	N/A
10523955	Dr1	N/A	N/A
10523960	Pigg	N/A	N/A
10523974	Gm10419	N/A	N/A
10523977	Gm10419	N/A	N/A
10524004	Pcgf3	N/A	N/A
10524018	Rpl34 /// Gm6404 /// Gm10154	N/A	N/A
10524020	Tmem175	N/A	N/A
10524034	Idua	N/A	N/A
10524077	---	N/A	N/A
10524079	2310001H12Rik	N/A	N/A
10524082	2310001H12Rik /// LOC100044193	N/A	N/A
10524105	Chfr	N/A	N/A
10524124	Golga3	N/A	N/A
10524150	Ankle2	N/A	N/A
10524227	Fbrsl1	N/A	N/A
10524234	Galnt9	N/A	N/A
10524264	---	N/A	N/A
10524284	Ttc28	N/A	N/A
10524308	Mir701	N/A	N/A
10524310	Ttc28	N/A	N/A
10524312	Ttc28	N/A	N/A
10524325	Gm10405	N/A	N/A
10524327	Mn1	N/A	N/A
10524331	C130026L21Rik	N/A	N/A

10524338	Crybb1	N/A	N/A
10524369	Hps4	N/A	N/A
10524384	Gm6588	N/A	N/A
10524394	Mir469	N/A	N/A
10524396	Aym1	N/A	N/A
10524398	Wscd2	N/A	N/A
10524411	Ficd	N/A	N/A
10524436	Usp30	N/A	N/A
10524460	Acacb	N/A	N/A
10524525	Ube3b	N/A	N/A
10524555	Mvk	N/A	N/A
10524568	BC057022	N/A	N/A
10524588	Ankrd13a /// 4930515G01Rik	N/A	N/A
10524621	Oasl2	N/A	N/A
10524631	Oasl1	N/A	N/A
10524640	2210016L21Rik /// Hnf1a	N/A	N/A
10524647	Sppl3	N/A	N/A
10524668	Coq5	N/A	N/A
10524676	Srsf9	N/A	N/A
10524681	Triap1	N/A	N/A
10524684	Msi1	N/A	N/A
10524698	Pla2g1b	N/A	N/A
10524703	Pxn	N/A	N/A
10524718	Rplp0	N/A	N/A
10524723	Gcn1l1	N/A	N/A
10524781	Rab35	N/A	N/A
10524842	4930569F06Rik	N/A	N/A
10524844	Taok3	N/A	N/A
10524874	Vsig10	N/A	N/A
10524876	Vsig10	N/A	N/A
10524878	Vsig10	N/A	N/A
10524889	Ksr2	N/A	N/A
10524909	Nos1	N/A	N/A
10524941	Fbxo21	N/A	N/A
10524965	Fbxw8 /// Gm9754	N/A	N/A
10524969	Hrk	N/A	N/A
10524973	2410131K14Rik	N/A	N/A
10524983	Med13l	N/A	N/A
10525014	---	N/A	N/A
10525016	Tbx3	N/A	N/A
10525041	---	N/A	N/A
10525076	Sds /// Plbd2	N/A	N/A
10525086	Slc24a6	N/A	N/A
10525111	Ddx54	N/A	N/A
10525187	Rpl6 /// Gm6807	N/A	N/A
10525195	Gm15800	N/A	N/A
10525210	Gm15800	N/A	N/A
10525236	Gm15800	N/A	N/A
10525271	Naa25	N/A	N/A
10525296	Brap	N/A	N/A
10525313	Atxn2	N/A	N/A
10525336	Fam109a	N/A	N/A
10525352	Ppp1cc	N/A	N/A
10525374	Pptc7	N/A	N/A
10525381	Vps29	N/A	N/A
10525387	Gpn3	N/A	N/A
10525397	Arpc3	N/A	N/A
10525406	Anapc7	N/A	N/A
10525439	P2rx4	N/A	N/A
10525452	Rnf34	N/A	N/A
10525460	A930024E05Rik	N/A	N/A
10525487	4932422M17Rik	N/A	N/A
10525489	Setd1b	N/A	N/A
10525495	Setd1b	N/A	N/A
10525506	Psmd9	N/A	N/A
10525542	Bcl7a	N/A	N/A
10525553	---	N/A	N/A
10525657	Denr	N/A	N/A
10525711	Ogfod2	N/A	N/A
10525726	2810006K23Rik	N/A	N/A
10525731	---	N/A	N/A
10525741	Snrnp35	N/A	N/A
10525744	Tmed2	N/A	N/A
10525766	Gtf2h3	N/A	N/A
10525780	Tctn2	N/A	N/A
10525804	Atp6v0a2	N/A	N/A
10525829	---	N/A	N/A
10525831	---	N/A	N/A
10525833	Atp6v0a2	N/A	N/A
10525835	Dnahc10	N/A	N/A
10525837	Dnahc10	N/A	N/A
10525854	Dnahc10	N/A	N/A
10525872	Zfp664	N/A	N/A
10525877	Zfp664	N/A	N/A

10525880	Fam101a	N/A	N/A
10525885	Ubc /// Gm10382	N/A	N/A
10525887	Bri3bp	N/A	N/A
10525893	Aacs	N/A	N/A
10525916	Tmem132b	N/A	N/A
10525921	Tmem132b	N/A	N/A
10525923	Tmem132b	N/A	N/A
10525929	---	N/A	N/A
10525932	Tmem132c	N/A	N/A
10525942	Glt1d1	N/A	N/A
10525983	Ran	N/A	N/A
10526014	Sfswap	N/A	N/A
10526038	Mmp17	N/A	N/A
10526049	Mrps17	N/A	N/A
10526055	Gbas	N/A	N/A
10526069	Cct6a	N/A	N/A
10526085	---	N/A	N/A
10526087	Sumf2 /// Phkg1	N/A	N/A
10526098	Scand3	N/A	N/A
10526106	Vkorc1l1	N/A	N/A
10526120	Tpst1	N/A	N/A
10526133	Rabgef1	N/A	N/A
10526145	0610007L01Rik	N/A	N/A
10526181	Gatsl2	N/A	N/A
10526191	Gatsl2	N/A	N/A
10526193	Wbscr16	N/A	N/A
10526211	---	N/A	N/A
10526213	---	N/A	N/A
10526229	Gm10369	N/A	N/A
10526232	Wbscr27	N/A	N/A
10526241	Cldn3	N/A	N/A
10526274	Dnajc30 /// Wbscr22	N/A	N/A
10526277	Mlxipl	N/A	N/A
10526302	Tbl2	N/A	N/A
10526311	Bcl7b	N/A	N/A
10526319	Baz1b	N/A	N/A
10526345	Nsun5	N/A	N/A
10526356	Rhbdd2	N/A	N/A
10526381	Mdh2	N/A	N/A
10526391	Srrm3	N/A	N/A
10526410	Hspb1 /// Gm9817	N/A	N/A
10526482	Polr2j	N/A	N/A
10526487	Alkbh4	N/A	N/A
10526502	Rabl5	N/A	N/A
10526508	Fis1	N/A	N/A
10526520	Plod3	N/A	N/A
10526553	Vgf	N/A	N/A
10526559	Ache	N/A	N/A
10526564	Ufsp1	N/A	N/A
10526566	Ephb4	N/A	N/A
10526587	Gigyf1	N/A	N/A
10526614	Actl6b	N/A	N/A
10526630	Trfr2	N/A	N/A
10526654	---	N/A	N/A
10526656	Lrch4 /// Lrch4-sap25 /// Sap25	N/A	N/A
10526687	2010007H12Rik	N/A	N/A
10526712	Azgp1	N/A	N/A
10526718	Smok3a /// Smok3b	N/A	N/A
10526720	Smok3a /// Smok3b	N/A	N/A
10526735	Zscan21	N/A	N/A
10526743	Cops6	N/A	N/A
10526754	Ap4m1 /// Mcm7	N/A	N/A
10526772	Cnpy4	N/A	N/A
10526781	Mblac1	N/A	N/A
10526792	0910001L09Rik	N/A	N/A
10526838	Got2 /// Gm10874	N/A	N/A
10526842	Zfp157	N/A	N/A
10526848	A430033K04Rik	N/A	N/A
10526853	Fam20c	N/A	N/A
10526880	Pdgfa /// 6330403L08Rik	N/A	N/A
10526882	Heatr2	N/A	N/A
10526897	Sun1	N/A	N/A
10526923	Get4	N/A	N/A
10526941	D830046C22Rik	N/A	N/A
10526952	Gpr30	N/A	N/A
10526966	---	N/A	N/A
10526968	Elfn1	N/A	N/A
10526972	Nudt1	N/A	N/A
10526977	Eif3b	N/A	N/A
10527009	Chst12	N/A	N/A
10527024	Gm10091	N/A	N/A
10527026	AA881470	N/A	N/A
10527043	Amz1	N/A	N/A
10527099	---	N/A	N/A

10527101	Foxk1	N/A	N/A
10527148	Slc29a4	N/A	N/A
10527158	Fscn1	N/A	N/A
10527172	Zfp12	N/A	N/A
10527182	E130309D02Rik	N/A	N/A
10527184	0610040B10Rik	N/A	N/A
10527213	Daglb	N/A	N/A
10527229	2810453I06Rik	N/A	N/A
10527252	Eif2ak1	N/A	N/A
10527306	Lmtk2	N/A	N/A
10527340	Trrap	N/A	N/A
10527423	Trrap	N/A	N/A
10527425	LOC280487	N/A	N/A
10527430	Arpc1a	N/A	N/A
10527441	Arpc1b	N/A	N/A
10527455	Cpsf4	N/A	N/A
10527465	Zkscan5	N/A	N/A
10527475	Zfp655	N/A	N/A
10527483	Zfp498	N/A	N/A
10527494	Cyp3a25 /// Cyp3a59 /// Cyp3a57	N/A	N/A
10527508	Cdk8	N/A	N/A
10527516	Wasf3	N/A	N/A
10527530	Rpl21 /// Gm9104 /// Rpl21-ps7 /// Gm16415 /// Gm16416 /// Rpl21-ps12 /// Rpl21-ps14 /// Rpl21-ps10 /// Gm8054	N/A	N/A
10527547	Gtf3a /// Mtif3	N/A	N/A
10527559	Polr1d	N/A	N/A
10527562	Gsx1	N/A	N/A
10527571	---	N/A	N/A
10527573	---	N/A	N/A
10527575	Pan3	N/A	N/A
10527598	Pomp	N/A	N/A
10527605	Mtus2	N/A	N/A
10527624	Uspl1	N/A	N/A
10527646	---	N/A	N/A
10527694	B3galtl	N/A	N/A
10527732	Fry	N/A	N/A
10527799	Gm10850	N/A	N/A
10527832	Pds5b	N/A	N/A
10527870	Kl	N/A	N/A
10527878	Gm4741 /// V1rg10 /// Vmn1r2 /// Vmn1r3 /// Vmn1r238	N/A	N/A
10527880	Pex1 /// C030048B08Rik	N/A	N/A
10527886	Pex1 /// Gatad1	N/A	N/A
10527888	Gatad1	N/A	N/A
10527896	Ankib1	N/A	N/A
10527920	Cyp51	N/A	N/A
10527934	---	N/A	N/A
10527936	Fzd1	N/A	N/A
10527940	Cdk14	N/A	N/A
10527959	---	N/A	N/A
10527961	---	N/A	N/A
10527963	Gm10484	N/A	N/A
10527970	Gtpbp10	N/A	N/A
10528008	Steap2	N/A	N/A
10528021	Hspa8 /// LOC624853	N/A	N/A
10528027	---	N/A	N/A
10528029	---	N/A	N/A
10528031	Zfp804b	N/A	N/A
10528036	---	N/A	N/A
10528038	Adam22	N/A	N/A
10528090	Rundc3b	N/A	N/A
10528102	Crot	N/A	N/A
10528120	Dmtf1	N/A	N/A
10528143	Ppp1r14b	N/A	N/A
10528145	Grm3	N/A	N/A
10528161	Gm10482	N/A	N/A
10528163	---	N/A	N/A
10528165	LOC280487	N/A	N/A
10528167	Gapdh /// Gm16374 /// Gm4609 /// Gm3200 /// Gm2451 /// Gm10293	N/A	N/A
10528170	Cycs	N/A	N/A
10528172	---	N/A	N/A
10528177	Gm9758 /// Speer4e /// Speer4d /// Speer4c /// Gm7361	N/A	N/A
10528183	Gm9758 /// Speer4e /// Speer4d /// Speer4c	N/A	N/A
10528198	---	N/A	N/A
10528200	Hnrnpa3	N/A	N/A
10528205	LOC280487	N/A	N/A
10528207	Cd36	N/A	N/A
10528227	Gnai1	N/A	N/A
10528238	Phtf2	N/A	N/A
10528257	Rsbn1l	N/A	N/A
10528268	Ptpn12	N/A	N/A
10528287	Gm6718	N/A	N/A
10528474	Rpl22l1 /// LOC100042049	N/A	N/A
10528476	---	N/A	N/A
10528478	Rpl17 /// Gm10268 /// Rpl17-ps3	N/A	N/A
10528480	---	N/A	N/A

10528482	5031425E22Rik	N/A	N/A
10528484	Srpk2 /// Mll5	N/A	N/A
10528519	---	N/A	N/A
10528521	---	N/A	N/A
10528527	Fam126a	N/A	N/A
10528546	Gabarapl2	N/A	N/A
10528548	Kcnh2	N/A	N/A
10528583	Cdk5	N/A	N/A
10528598	Fastk	N/A	N/A
10528615	Gm10472	N/A	N/A
10528648	Abcf2	N/A	N/A
10528662	Atp5l	N/A	N/A
10528691	Rheb	N/A	N/A
10528702	Prkag2	N/A	N/A
10528721	Galnt11 /// E130116L18Rik	N/A	N/A
10528723	Mll3	N/A	N/A
10528788	Gm10062	N/A	N/A
10528794	Gm10471	N/A	N/A
10528804	Gm10471	N/A	N/A
10528810	Speer4b /// Gm10471 /// Gm10220 /// Speer4a /// 5031410I06Rik /// Gm5862 /// Gm7347 /// Gm7361	N/A	N/A
10528815	Speer4b	N/A	N/A
10528821	Speer4b //	N/A	N/A
10528832	---	N/A	N/A
10528840	Paxip1	N/A	N/A
10528880	Lmbr1	N/A	N/A
10528901	C79130	N/A	N/A
10528909	---	N/A	N/A
10528911	---	N/A	N/A
10528913	Dnajb6 /// Gm5129	N/A	N/A
10528925	3110082J24Rik	N/A	N/A
10528929	Hadha	N/A	N/A
10528970	---	N/A	N/A
10529026	LOC100502829	N/A	N/A
10529028	Ost4 /// Agbl5	N/A	N/A
10529034	Cgref1	N/A	N/A
10529041	Preb	N/A	N/A
10529082	Mpv17	N/A	N/A
10529091	Gtf3c2	N/A	N/A
10529093	Gtf3c2	N/A	N/A
10529133	Zfp513	N/A	N/A
10529142	Ppm1g	N/A	N/A
10529154	Nrbp1 /// Gm9970	N/A	N/A
10529156	Ift172	N/A	N/A
10529206	Fndc4	N/A	N/A
10529215	---	N/A	N/A
10529226	Rbks	N/A	N/A
10529235	Gm10463	N/A	N/A
10529237	Yes1 /// Gm10461	N/A	N/A
10529252	C330019G07Rik	N/A	N/A
10529260	C330019G07Rik	N/A	N/A
10529273	Ctbp1	N/A	N/A
10529285	Nkx1-1	N/A	N/A
10529287	Fam53a	N/A	N/A
10529299	Slbp	N/A	N/A
10529305	Tmem129	N/A	N/A
10529311	G630022F23Rik	N/A	N/A
10529313	Letm1	N/A	N/A
10529375	Mxd4	N/A	N/A
10529402	Tnip2	N/A	N/A
10529410	Mfsd10	N/A	N/A
10529425	Nop14	N/A	N/A
10529445	Lrpap1	N/A	N/A
10529454	E130018O15Rik	N/A	N/A
10529468	2310079F23Rik	N/A	N/A
10529480	Acox3	N/A	N/A
10529544	---	N/A	N/A
10529547	Tada2b	N/A	N/A
10529567	D5Ertd579e	N/A	N/A
10529577	Cno	N/A	N/A
10529581	Mrfap1	N/A	N/A
10529584	Man2b2	N/A	N/A
10529605	Wfs1	N/A	N/A
10529636	Stk32b	N/A	N/A
10529656	Nsg1	N/A	N/A
10529689	Wdr1	N/A	N/A
10529706	4930421P07Rik	N/A	N/A
10529708	Zfp518b	N/A	N/A
10529730	---	N/A	N/A
10529739	---	N/A	N/A
10529741	Rab28	N/A	N/A
10529758	Bod1l	N/A	N/A
10529792	---	N/A	N/A
10529797	---	N/A	N/A
10529799	Cpeb2 /// Gm7854	N/A	N/A

10529801	Fbxl5	N/A	N/A
10529815	LOC100505155	N/A	N/A
10529817	Rpl15 /// Gm5292 /// Gm4294	N/A	N/A
10529858	Tapt1	N/A	N/A
10529873	Rab2a	N/A	N/A
10529875	Ldb2	N/A	N/A
10529887	---	N/A	N/A
10529895	Qdpr	N/A	N/A
10529921	---	N/A	N/A
10529923	Lcorl	N/A	N/A
10529937	Kcnip4	N/A	N/A
10529953	Gm10048	N/A	N/A
10529957	Gpr125	N/A	N/A
10529977	Ppargc1a	N/A	N/A
10529979	Ppargc1a	N/A	N/A
10529995	Dhx15	N/A	N/A
10530013	Gm447	N/A	N/A
10530017	---	N/A	N/A
10530029	Lgi2	N/A	N/A
10530045	Sepsecs	N/A	N/A
10530087	Gm10441	N/A	N/A
10530096	---	N/A	N/A
10530098	---	N/A	N/A
10530100	Arap2	N/A	N/A
10530128	Gm9954 /// 3110047P20Rik	N/A	N/A
10530140	---	N/A	N/A
10530194	Rpl9 /// Gm5451	N/A	N/A
10530201	Ugdh	N/A	N/A
10530215	1110003E01Rik	N/A	N/A
10530225	Pds5a	N/A	N/A
10530257	N4bp2	N/A	N/A
10530269	Rbm47	N/A	N/A
10530278	---	N/A	N/A
10530283	---	N/A	N/A
10530287	Apbb2	N/A	N/A
10530319	Atp8a1	N/A	N/A
10530369	---	N/A	N/A
10530391	---	N/A	N/A
10530393	Gabrg1	N/A	N/A
10530421	Gabra4	N/A	N/A
10530467	Nfxl1	N/A	N/A
10530477	Nfxl1	N/A	N/A
10530492	Nfxl1	N/A	N/A
10530496	Zar1	N/A	N/A
10530499	Slc10a4 /// Gm5868	N/A	N/A
10530536	Tec	N/A	N/A
10530558	Slain2	N/A	N/A
10530560	Slain2	N/A	N/A
10530563	Fryl	N/A	N/A
10530592	Fryl	N/A	N/A
10530612	Fryl	N/A	N/A
10530615	Ociad2	N/A	N/A
10530625	---	N/A	N/A
10530633	Sgcb	N/A	N/A
10530641	Usp46	N/A	N/A
10530652	Scfd2	N/A	N/A
10530683	Chic2	N/A	N/A
10530722	A430089I19Rik /// BC061212 /// BC080696 /// LOC665755 /// Gm3147 /// Gm10424	N/A	N/A
10530726	A430089I19Rik /// BC061212 /// BC080696 /// LOC665755 /// Gm3147 /// Gm10424	N/A	N/A
10530731	Srd5a3	N/A	N/A
10530759	Ube2n	N/A	N/A
10530783	Gm10430	N/A	N/A
10530787	Aasdh	N/A	N/A
10530806	Ppat	N/A	N/A
10530841	Igfbp7	N/A	N/A
10530847	Pea15b	N/A	N/A
10530849	---	N/A	N/A
10530851	Mtch2	N/A	N/A
10530868	---	N/A	N/A
10530870	Epha5	N/A	N/A
10530910	Uba6	N/A	N/A
10531034	Ugt2b34	N/A	N/A
10531049	---	N/A	N/A
10531057	Ugt2b5	N/A	N/A
10531061	Ugt2b37	N/A	N/A
10531066	Ugt2a3	N/A	N/A
10531073	Ugt2b38	N/A	N/A
10531087	Sult1b1	N/A	N/A
10531100	Sult1d1	N/A	N/A
10531133	Grsf1	N/A	N/A
10531144	Rpl21 /// Gm6813 /// Gm9104 /// Rpl21-ps4 /// Rpl21-ps7 /// Gm16416 /// Rpl21-ps12 /// Rpl21-ps10 /// Gm8054	N/A	N/A
10531149	Gc	N/A	N/A
10531166	Adamts3	N/A	N/A
10531173	Adamts3	N/A	N/A

10531175	Adamts3	N/A	N/A
10531177	Adamts3	N/A	N/A
10531179	Adamts3	N/A	N/A
10531181	Adamts3	N/A	N/A
10531183	Adamts3	N/A	N/A
10531185	Adamts3	N/A	N/A
10531187	Adamts3	N/A	N/A
10531189	Adamts3	N/A	N/A
10531191	Adamts3	N/A	N/A
10531193	Adamts3	N/A	N/A
10531195	Adamts3	N/A	N/A
10531197	Adamts3	N/A	N/A
10531199	D130050E23Rik	N/A	N/A
10531201	Adamts3	N/A	N/A
10531203	Adamts3	N/A	N/A
10531208	Cox18	N/A	N/A
10531215	Ankrd17	N/A	N/A
10531259	Gm10426	N/A	N/A
10531261	Rassf6	N/A	N/A
10531284	Trmt112	N/A	N/A
10531286	Vdac2	N/A	N/A
10531288	---	N/A	N/A
10531290	Rchy1 /// Thap6	N/A	N/A
10531323	G3bp2	N/A	N/A
10531338	---	N/A	N/A
10531370	Naaa	N/A	N/A
10531383	Sdad1	N/A	N/A
10531407	Cxcl9	N/A	N/A
10531415	Cxcl10	N/A	N/A
10531437	Scarb2	N/A	N/A
10531488	Ccni	N/A	N/A
10531497	Gm16367 /// D5Ertd577e /// E330014E10Rik /// Gm16427 /// Gm7792 /// Gm3106	N/A	N/A
10531507	A430089I19Rik /// BC061212 /// BC080696 /// LOC665755 /// Gm3147 /// Gm10424	N/A	N/A
10531512	A430089I19Rik /// BC061212 /// BC080696 /// LOC665755 /// Gm3147 /// Gm10424	N/A	N/A
10531518	A430089I19Rik /// BC061212 /// BC080696 /// LOC665755 /// Gm3147 /// Gm10424	N/A	N/A
10531523	A430089I19Rik /// BC061212 /// BC080696 /// LOC665755 /// Gm3147 /// Gm10424	N/A	N/A
10531529	Cnot6l	N/A	N/A
10531544	Paqr3	N/A	N/A
10531560	Antxr2	N/A	N/A
10531579	Hmgb1	N/A	N/A
10531610	Rasgef1b	N/A	N/A
10531627	A930011G23Rik	N/A	N/A
10531633	Hnrnpd	N/A	N/A
10531675	Sec31a	N/A	N/A
10531707	Lin54	N/A	N/A
10531722	---	N/A	N/A
10531724	Plac8	N/A	N/A
10531752	Helq	N/A	N/A
10531776	Fam175a	N/A	N/A
10531794	Wdfy3	N/A	N/A
10531796	Wdfy3	N/A	N/A
10531866	Mapk10	N/A	N/A
10531869	Mapk10	N/A	N/A
10531899	Klhl8	N/A	N/A
10531910	Hsd17b13	N/A	N/A
10531919	Hsd17b11	N/A	N/A
10531928	---	N/A	N/A
10531931	Sparcl1	N/A	N/A
10531944	Gm10047	N/A	N/A
10531950	---	N/A	N/A
10531970	---	N/A	N/A
10531987	Gbp4	N/A	N/A
10531994	Gbp10 /// Mpa2l /// Gbp11 /// Gbp8	N/A	N/A
10532019	Gbp11 /// Mpa2l	N/A	N/A
10532025	Rps15a	N/A	N/A
10532027	Gapdh /// Gm16374 /// Gm2606 /// Gm4609 /// Gm3200 /// Gm2451 /// Gm10293	N/A	N/A
10532030	---	N/A	N/A
10532040	Zfp644	N/A	N/A
10532085	Tgfbr3	N/A	N/A
10532133	Evi5	N/A	N/A
10532150	Fam69a	N/A	N/A
10532157	Tmed5	N/A	N/A
10532164	Atp5k	N/A	N/A
10532180	Cplx1	N/A	N/A
10532187	Gak	N/A	N/A
10532241	Slc26a1 /// Idua	N/A	N/A
10532301	---	N/A	N/A
10532305	2310001H12Rik /// 4930522L14Rik	N/A	N/A
10532308	---	N/A	N/A
10532310	2310001H12Rik /// 4930522L14Rik	N/A	N/A
10532313	AB010352	N/A	N/A
10532315	---	N/A	N/A
10532317	Gtpbp6	N/A	N/A
10532330	Ankle2	N/A	N/A

10532332	Pgam5	N/A	N/A
10532339	Pxmp2	N/A	N/A
10532368	Fbrsl1	N/A	N/A
10532390	Noc4l	N/A	N/A
10532407	Ep400	N/A	N/A
10532461	---	N/A	N/A
10532534	Gm6583	N/A	N/A
10532538	Asphd2	N/A	N/A
10532542	Sez6l	N/A	N/A
10532574	Myo18b	N/A	N/A
10532576	Myo18b	N/A	N/A
10532578	Myo18b	N/A	N/A
10532580	Myo18b	N/A	N/A
10532582	Myo18b	N/A	N/A
10532584	Myo18b	N/A	N/A
10532586	Myo18b	N/A	N/A
10532588	Myo18b	N/A	N/A
10532592	Myo18b	N/A	N/A
10532594	Myo18b	N/A	N/A
10532596	Myo18b	N/A	N/A
10532616	Myo18b	N/A	N/A
10532618	Myo18b	N/A	N/A
10532620	Myo18b	N/A	N/A
10532624	Myo18b	N/A	N/A
10532626	Myo18b	N/A	N/A
10532630	Adrbk2	N/A	N/A
10532669	2900026A02Rik	N/A	N/A
10532678	F830115B05Rik	N/A	N/A
10532680	Sgsm1	N/A	N/A
10532709	---	N/A	N/A
10532744	Selplg	N/A	N/A
10532753	Coro1c	N/A	N/A
10532765	---	N/A	N/A
10532767	Ssh1	N/A	N/A
10532784	Svop	N/A	N/A
10532802	Alkbh2	N/A	N/A
10532857	Gltp	N/A	N/A
10532865	Git2	N/A	N/A
10532892	1500011B03Rik	N/A	N/A
10532896	2610524H06Rik	N/A	N/A
10532901	---	N/A	N/A
10532903	Mir469	N/A	N/A
10532907	Hnf1a	N/A	N/A
10532919	Gm5148 /// Rps23	N/A	N/A
10532921	Sppl3 /// Gm10401	N/A	N/A
10532926	Acads	N/A	N/A
10532937	Unc119b	N/A	N/A
10532944	Mlec	N/A	N/A
10532954	Mlec	N/A	N/A
10532984	Dynll1	N/A	N/A
10532989	Gatc	N/A	N/A
10532993	Cox6a1	N/A	N/A
10532997	4930430O22Rik	N/A	N/A
10532999	Sirt4	N/A	N/A
10533003	1110006O24Rik	N/A	N/A
10533007	Ccdc64	N/A	N/A
10533023	Rpl29 /// Gm3550 /// Rpl29-ps2	N/A	N/A
10533026	Prkab1	N/A	N/A
10533050	Hspb8	N/A	N/A
10533055	Srrm4	N/A	N/A
10533071	Suds3	N/A	N/A
10533085	Pebp1	N/A	N/A
10533088	Gm10399	N/A	N/A
10533090	Rfc5	N/A	N/A
10533095	Fbxw8 /// Gm9754	N/A	N/A
10533120	Rbm19	N/A	N/A
10533122	Sdsl	N/A	N/A
10533131	Plbd2	N/A	N/A
10533145	Tpcn1	N/A	N/A
10533176	1110008J03Rik	N/A	N/A
10533180	Ddx54	N/A	N/A
10533182	Dtx1	N/A	N/A
10533261	Rph3a	N/A	N/A
10533285	Ptpn11	N/A	N/A
10533304	Trafd1	N/A	N/A
10533316	Erp29	N/A	N/A
10533320	Adam1b	N/A	N/A
10533323	Adam1a	N/A	N/A
10533345	Aldh2	N/A	N/A
10533386	Sh2b3	N/A	N/A
10533401	Cux2	N/A	N/A
10533403	Cux2	N/A	N/A
10533444	4930565B19Rik	N/A	N/A
10533446	Tctn1 /// Hvcn1	N/A	N/A

10533462	Rad9b	N/A	N/A
10533474	1500011H22Rik	N/A	N/A
10533483	Atp2a2	N/A	N/A
10533504	Ift81	N/A	N/A
10533526	---	N/A	N/A
10533549	Anapc5	N/A	N/A
10533569	Kdm2b	N/A	N/A
10533603	Rhof /// Tmem120b	N/A	N/A
10533612	Hpd	N/A	N/A
10533626	Rpl35a /// Ino80 /// Gm10247	N/A	N/A
10533633	Diablo /// B3gnt4	N/A	N/A
10533644	Vps33a	N/A	N/A
10533659	Clip1	N/A	N/A
10533687	Zcchc8	N/A	N/A
10533703	Rsrc2	N/A	N/A
10533725	Gpr81	N/A	N/A
10533729	Vps37b	N/A	N/A
10533781	Mphosph9	N/A	N/A
10533807	Cdk2ap1	N/A	N/A
10533812	Sbno1	N/A	N/A
10533849	Rilpl1	N/A	N/A
10533858	Eif2b1	N/A	N/A
10533869	Ccdc92	N/A	N/A
10533929	Scarb1	N/A	N/A
10533945	Ubc	N/A	N/A
10533961	Dhx37	N/A	N/A
10533989	Gm4868	N/A	N/A
10533991	---	N/A	N/A
10533993	Slc15a4	N/A	N/A
10534002	Tmem132d	N/A	N/A
10534021	Rimbp2	N/A	N/A
10534056	Hpvc-ps	N/A	N/A
10534059	Rps2 /// Rps2-ps6 /// Gm5921 /// Gm6139	N/A	N/A
10534075	Psph	N/A	N/A
10534085	Phkg1 /// Sumf2	N/A	N/A
10534096	Chchd2	N/A	N/A
10534120	Asl	N/A	N/A
10534140	0610007L01Rik /// Gm6598	N/A	N/A
10534142	Sbds	N/A	N/A
10534152	Wbscr17	N/A	N/A
10534168	Auts2	N/A	N/A
10534216	Gtf2i	N/A	N/A
10534281	Clip2	N/A	N/A
10534301	Gm52	N/A	N/A
10534316	Eif4h	N/A	N/A
10534324	Limk1	N/A	N/A
10534384	Wbscr28	N/A	N/A
10534395	Cldn4	N/A	N/A
10534405	Wbscr22 /// Dnajc30	N/A	N/A
10534420	Vps37d	N/A	N/A
10534426	Fzd9	N/A	N/A
10534441	Pom121 /// Nsun5	N/A	N/A
10534501	Rhbdd2	N/A	N/A
10534504	Tmem120a	N/A	N/A
10534531	Ywhag	N/A	N/A
10534549	---	N/A	N/A
10534570	Orai2	N/A	N/A
10534575	Prkrip1	N/A	N/A
10534583	---	N/A	N/A
10534585	Sh2b2	N/A	N/A
10534596	Cux1	N/A	N/A
10534640	Emid2	N/A	N/A
10534660	Ap1s1	N/A	N/A
10534667	Serpine1	N/A	N/A
10534679	Trim56	N/A	N/A
10534694	Srrt	N/A	N/A
10534728	Slc12a9	N/A	N/A
10534839	Pop7	N/A	N/A
10534842	Gnb2	N/A	N/A
10534854	Mospd3	N/A	N/A
10534909	Sp110 /// LOC100041885	N/A	N/A
10534921	Mepce	N/A	N/A
10534927	Pilra	N/A	N/A
10534945	Cyp3a13	N/A	N/A
10534960	Gjc3	N/A	N/A
10534964	Rps25 /// Gm4963	N/A	N/A
10534966	Zfp113	N/A	N/A
10534984	Mir25	N/A	N/A
10534988	Mir106b	N/A	N/A
10534990	Taf6	N/A	N/A
10535006	BC037034	N/A	N/A
10535017	Gal3st4 /// Gpc2	N/A	N/A
10535025	Got2 /// Gm10874	N/A	N/A
10535034	Zfp68	N/A	N/A

10535043	Pdgfa	N/A	N/A
10535053	Prkar1b	N/A	N/A
10535080	Cox19	N/A	N/A
10535084	3110082I17Rik	N/A	N/A
10535091	4930432F04Rik /// Mir339	N/A	N/A
10535095	Zfand2a	N/A	N/A
10535174	Tmem184a	N/A	N/A
10535208	Ftsj2 /// Nudt1	N/A	N/A
10535213	Snx8	N/A	N/A
10535231	Ttyh3	N/A	N/A
10535247	Iqce	N/A	N/A
10535310	Foxk1	N/A	N/A
10535329	Papolb	N/A	N/A
10535331	Mmd2	N/A	N/A
10535340	Wipi2	N/A	N/A
10535369	---	N/A	N/A
10535372	Fbxl18	N/A	N/A
10535378	D430018E03Rik	N/A	N/A
10535381	Actb	N/A	N/A
10535389	Rnf216	N/A	N/A
10535410	---	N/A	N/A
10535413	---	N/A	N/A
10535418	Rbak	N/A	N/A
10535449	E130309D02Rik	N/A	N/A
10535458	Zdhhc4	N/A	N/A
10535467	9530056K15Rik	N/A	N/A
10535471	Rac1 /// Daglb	N/A	N/A
10535477	Usp42	N/A	N/A
10535497	Ankrd61	N/A	N/A
10535502	Aimp2	N/A	N/A
10535508	AU022870	N/A	N/A
10535532	Tecpr1	N/A	N/A
10535559	Baiap2l1	N/A	N/A
10535575	Gm6272	N/A	N/A
10535577	Tmem130	N/A	N/A
10535586	Smurf1	N/A	N/A
10535626	Rpl29	N/A	N/A
10535629	Pdap1	N/A	N/A
10535637	Ptcd1	N/A	N/A
10535647	Atp5j2	N/A	N/A
10535653	Zkscan14	N/A	N/A
10535704	Cyp3a11	N/A	N/A
10535714	Cyp3a25	N/A	N/A
10535725	Rnf6	N/A	N/A
10535732	Gpr12	N/A	N/A
10535739	Usp12	N/A	N/A
10535747	Gm10858	N/A	N/A
10535750	Mtif3	N/A	N/A
10535776	Prhoxnb	N/A	N/A
10535807	Flt1	N/A	N/A
10535841	Slc46a3	N/A	N/A
10535849	---	N/A	N/A
10535852	Slc7a1	N/A	N/A
10535881	---	N/A	N/A
10535883	Katnal1	N/A	N/A
10535894	Hmgb1	N/A	N/A
10535900	---	N/A	N/A
10535904	Hsph1	N/A	N/A
10535927	Dscaml1 /// Nme2	N/A	N/A
10535938	N4bp2l1	N/A	N/A
10535946	N4bp2l2	N/A	N/A
10535954	Gm8675	N/A	N/A
10535956	Stard13	N/A	N/A
10535972	D730045B01Rik	N/A	N/A
10535979	Rfc3	N/A	N/A
10535989	A430089I19Rik /// BC061212 /// BC080696 /// LOC665755 /// Gm3147 /// Gm10424	N/A	N/A
10536002	C87414 /// AA792892	N/A	N/A
10536021	Gm16367 /// D5Ertd577e /// E330014E10Rik /// Gm16427 /// Gm7792 /// Gm3106	N/A	N/A
10536025	LOC624931 /// EG665031 /// LOC625240 /// Gm7682	N/A	N/A
10536037	Gm16367 /// D5Ertd577e /// E330014E10Rik /// Gm16427 /// Gm7792 /// Gm3106	N/A	N/A
10536041	C87414 /// AA792892	N/A	N/A
10536048	Gm16367 /// D5Ertd577e /// E330014E10Rik /// Gm16427 /// Gm7792 /// Gm3106	N/A	N/A
10536052	LOC624931 /// EG665031 /// Gm7682	N/A	N/A
10536061	Zfp141	N/A	N/A
10536068	Zfp788	N/A	N/A
10536079	Gprc2a-rs5	N/A	N/A
10536083	Gprc2a-rs5 /// Vmn2r58 /// Vmn2r60 /// Vmn2r61 /// Vmn2r-ps60	N/A	N/A
10536085	A430089I19Rik /// BC061212 /// BC080696 /// LOC665755 /// Gm3147 /// Gm10424	N/A	N/A
10536090	---	N/A	N/A
10536103	LOC624931 /// EG665031 /// LOC625240 /// Gm7682	N/A	N/A
10536122	---	N/A	N/A
10536136	A430089I19Rik /// BC061212 /// BC080696 /// LOC665755 /// Gm3147 /// Gm10424	N/A	N/A
10536141	Dullard	N/A	N/A
10536143	LOC624931 /// EG665031 /// LOC625240 /// Gm7682	N/A	N/A

10536147	Gm16367 /// D5Ertd577e /// E330014E10Rik /// Gm16427 /// Gm7792 /// Gm3106	N/A	N/A
10536151	LOC624931 /// EG665031 /// LOC625240 /// Gm7682	N/A	N/A
10536155	D5Ertd577e /// Gm16367 /// E330014E10Rik /// Gm16427 /// Gm7792 /// Gm3106	N/A	N/A
10536159	Gm16367 /// D5Ertd577e /// E330014E10Rik /// Gm16427 /// Gm7792 /// Gm3106	N/A	N/A
10536170	Rn18s	N/A	N/A
10536176	Ccdc132	N/A	N/A
10536216	Gng11	N/A	N/A
10536273	Casd1	N/A	N/A
10536294	Peg10	N/A	N/A
10536297	Ppp1r9a	N/A	N/A
10536334	Dync1i1	N/A	N/A
10536353	Dlx6	N/A	N/A
10536359	Acn9	N/A	N/A
10536363	Tac1	N/A	N/A
10536369	C1galt1	N/A	N/A
10536376	Mios	N/A	N/A
10536401	Nxph1	N/A	N/A
10536405	Nxph1	N/A	N/A
10536407	Phf14	N/A	N/A
10536425	---	N/A	N/A
10536429	Tmem106b	N/A	N/A
10536440	Gm725	N/A	N/A
10536442	---	N/A	N/A
10536444	Foxp2	N/A	N/A
10536481	---	N/A	N/A
10536483	Tes	N/A	N/A
10536494	Cav2	N/A	N/A
10536499	Cav1	N/A	N/A
10536505	Met	N/A	N/A
10536541	St7	N/A	N/A
10536593	Tsen15	N/A	N/A
10536595	Naa38	N/A	N/A
10536609	---	N/A	N/A
10536611	Kcnd2	N/A	N/A
10536620	Ing3	N/A	N/A
10536635	A430107O13Rik	N/A	N/A
10536667	Ptprz1	N/A	N/A
10536743	---	N/A	N/A
10536746	Arf5	N/A	N/A
10536762	Snd1	N/A	N/A
10536787	Mir129-1	N/A	N/A
10536818	Calu	N/A	N/A
10536827	Ccdc136	N/A	N/A
10536895	Atp6v1f	N/A	N/A
10536898	Irf5	N/A	N/A
10536908	Tspan33	N/A	N/A
10536917	Smo	N/A	N/A
10536931	Ahcyl2	N/A	N/A
10536949	Fam40b	N/A	N/A
10536973	---	N/A	N/A
10536994	---	N/A	N/A
10536996	Klhdc10	N/A	N/A
10537062	Mest /// Copg2	N/A	N/A
10537076	Mir335	N/A	N/A
10537078	Mkln1	N/A	N/A
10537098	1700012A03Rik	N/A	N/A
10537102	Exoc4	N/A	N/A
10537157	Akr1b10	N/A	N/A
10537184	Cald1	N/A	N/A
10537227	Tmem140 /// 3110062M04Rik	N/A	N/A
10537244	Rpl17 /// Rpl17-ps3	N/A	N/A
10537246	Nup205	N/A	N/A
10537290	---	N/A	N/A
10537292	1810058I24Rik	N/A	N/A
10537296	Mir490	N/A	N/A
10537298	Chrm2	N/A	N/A
10537306	Akr1d1	N/A	N/A
10537316	Atp6v0c /// Atp6v0c-ps2	N/A	N/A
10537343	Tmem213 /// Atp6v0a4	N/A	N/A
10537347	---	N/A	N/A
10537349	Rpl30 /// Gm12191 /// Gm6570 /// Gm6109	N/A	N/A
10537353	Ttc26	N/A	N/A
10537375	Ubn2	N/A	N/A
10537394	1110001J03Rik	N/A	N/A
10537397	Luc7l2	N/A	N/A
10537406	Clec2l	N/A	N/A
10537426	---	N/A	N/A
10537437	Mkrn1	N/A	N/A
10537441	Adck2	N/A	N/A
10537452	Ndufb2	N/A	N/A
10537458	Gm5567	N/A	N/A
10537463	Agk	N/A	N/A
10537494	Ssbp1	N/A	N/A
10537499	Tas2r137	N/A	N/A

10537501	Tas2r108	N/A	N/A
10537504	---	N/A	N/A
10537506	Olfr460	N/A	N/A
10537509	Mgam	N/A	N/A
10537558	Gm6273	N/A	N/A
10537657	Ephb6	N/A	N/A
10537710	Tas2r144	N/A	N/A
10537712	Gstk1	N/A	N/A
10537728	Casp2	N/A	N/A
10537770	Zyx	N/A	N/A
10537785	Tas2r143	N/A	N/A
10537789	Tas2r126	N/A	N/A
10537791	Olfr453	N/A	N/A
10537797	---	N/A	N/A
10537799	Olfr450	N/A	N/A
10537801	Olfr449	N/A	N/A
10537803	Olfr448	N/A	N/A
10537805	Olfr447	N/A	N/A
10537807	Olfr446	N/A	N/A
10537817	Olfr237-ps1 /// Olfr444 /// Olfr441	N/A	N/A
10537821	Olfr437	N/A	N/A
10537826	Olfr435	N/A	N/A
10537828	Olfr434	N/A	N/A
10537849	Arhgef5	N/A	N/A
10537851	Cntnap2	N/A	N/A
10537880	---	N/A	N/A
10537909	Rny3	N/A	N/A
10537911	Zfp398	N/A	N/A
10537934	Zfp212	N/A	N/A
10537938	AI894139 /// Zfp783	N/A	N/A
10537957	Krba1	N/A	N/A
10538080	Rpl35a /// Ino80 /// Gm10247	N/A	N/A
10538082	Atp6v0e2	N/A	N/A
10538087	Lrrc61	N/A	N/A
10538106	Zfp775	N/A	N/A
10538109	AI854703	N/A	N/A
10538135	Gimap7	N/A	N/A
10538138	Gimap1	N/A	N/A
10538150	Tmem176a	N/A	N/A
10538159	Gm7932	N/A	N/A
10538201	2410003K15Rik	N/A	N/A
10538247	Npy	N/A	N/A
10538253	Mpp6	N/A	N/A
10538269	Dfna5	N/A	N/A
10538271	---	N/A	N/A
10538273	BC022713	N/A	N/A
10538275	Nfe2l3	N/A	N/A
10538282	Cbx3 /// Gm6901	N/A	N/A
10538305	5730596B20Rik	N/A	N/A
10538318	Tax1bp1	N/A	N/A
10538352	---	N/A	N/A
10538354	1200009O22Rik	N/A	N/A
10538394	Plekha8	N/A	N/A
10538408	2410066E13Rik	N/A	N/A
10538413	Znrf2	N/A	N/A
10538420	Gars	N/A	N/A
10538459	Aqp1	N/A	N/A
10538482	Adcyap1r1	N/A	N/A
10538519	Gsbs	N/A	N/A
10538526	Avl9	N/A	N/A
10538545	Gm10209	N/A	N/A
10538547	Fkbp9	N/A	N/A
10538569	Vmn1r9 /// Vmn1r10	N/A	N/A
10538571	Vmn1r9 /// Vmn1r10	N/A	N/A
10538582	Vmn1r15	N/A	N/A
10538584	---	N/A	N/A
10538586	Vmn1r19	N/A	N/A
10538588	Vmn1r20 /// Vmn1r27	N/A	N/A
10538617	Lancl2	N/A	N/A
10538629	Rps15	N/A	N/A
10538636	Vmn1r29	N/A	N/A
10538638	---	N/A	N/A
10538640	Abcg2	N/A	N/A
10538658	Herc3	N/A	N/A
10538684	Tigd2	N/A	N/A
10538704	---	N/A	N/A
10538732	Grid2	N/A	N/A
10538749	---	N/A	N/A
10538751	Atoh1	N/A	N/A
10538753	---	N/A	N/A
10538781	Gm8479	N/A	N/A
10538783	C130060K24Rik	N/A	N/A
10538802	A930038C07Rik	N/A	N/A
10538811	Prdm5	N/A	N/A

10538832	Mad2l1	N/A	N/A
10538840	Vmn1r35 /// Vmn1r37 /// Vmn1r36 /// Vmn1r34	N/A	N/A
10538842	Gng12	N/A	N/A
10538848	---	N/A	N/A
10538850	---	N/A	N/A
10538852	A430010J10Rik	N/A	N/A
10538855	---	N/A	N/A
10538857	Serbp1	N/A	N/A
10538871	Gm4964	N/A	N/A
10538878	Gm10877	N/A	N/A
10538885	---	N/A	N/A
10538887	Gm5153	N/A	N/A
10538890	LOC641050	N/A	N/A
10538901	BC005685	N/A	N/A
10538921	---	N/A	N/A
10538924	LOC100046496	N/A	N/A
10538929	---	N/A	N/A
10538934	---	N/A	N/A
10538939	Eif2ak3	N/A	N/A
10538957	---	N/A	N/A
10538963	Rpl34 /// Gm6404 /// Gm10154	N/A	N/A
10538965	Fabp1	N/A	N/A
10538970	Krcc1	N/A	N/A
10538975	Gm1070	N/A	N/A
10539002	Rnf103	N/A	N/A
10539015	---	N/A	N/A
10539017	Reep1	N/A	N/A
10539026	Immt	N/A	N/A
10539042	Polr1a	N/A	N/A
10539104	0610030E20Rik	N/A	N/A
10539111	Tmem150a	N/A	N/A
10539119	Ggcx	N/A	N/A
10539143	Retsat	N/A	N/A
10539156	Gm15401	N/A	N/A
10539159	Suclg1	N/A	N/A
10539177	Eif4a3 /// Gm8994	N/A	N/A
10539209	---	N/A	N/A
10539211	Lrrtm4	N/A	N/A
10539238	Fam176a	N/A	N/A
10539242	---	N/A	N/A
10539244	Tacr1	N/A	N/A
10539310	Pcgf1	N/A	N/A
10539337	Mogs	N/A	N/A
10539358	1700003E16Rik	N/A	N/A
10539364	Dctn1	N/A	N/A
10539421	Mobkl1b	N/A	N/A
10539433	Mobkl1b	N/A	N/A
10539435	Bola3	N/A	N/A
10539441	B230319C09Rik	N/A	N/A
10539472	Nagk	N/A	N/A
10539484	Rpl18	N/A	N/A
10539486	Zfml	N/A	N/A
10539574	Npm3 /// Npm3-ps1	N/A	N/A
10539577	Spr	N/A	N/A
10539579	Gm5878	N/A	N/A
10539581	---	N/A	N/A
10539584	Emx1	N/A	N/A
10539592	Smyd5	N/A	N/A
10539606	Cct7	N/A	N/A
10539617	Alms1	N/A	N/A
10539632	Alms1	N/A	N/A
10539640	Alms1	N/A	N/A
10539649	Ptges3	N/A	N/A
10539653	Tprkb	N/A	N/A
10539669	Add2	N/A	N/A
10539700	Rps28	N/A	N/A
10539702	Fam136a	N/A	N/A
10539710	Tia1	N/A	N/A
10539727	C87436	N/A	N/A
10539739	Asprv1	N/A	N/A
10539741	Aak1	N/A	N/A
10539766	Aak1	N/A	N/A
10539773	Gfpt1	N/A	N/A
10539802	Bmp10	N/A	N/A
10539810	Ccdc48	N/A	N/A
10539813	Ccdc48	N/A	N/A
10539822	Copg	N/A	N/A
10539850	8430410A17Rik	N/A	N/A
10539857	Gm5577 /// Rab7	N/A	N/A
10539861	Rpn1	N/A	N/A
10539880	Dnajb8 /// 1700031F10Rik	N/A	N/A
10539882	Ruvbl1	N/A	N/A
10539894	Mgll	N/A	N/A
10539905	Podxl2	N/A	N/A

10539907	Tpra1	N/A	N/A
10539927	Gm1965	N/A	N/A
10539931	Gm1965	N/A	N/A
10539952	Vmn1r40	N/A	N/A
10539957	Vmn1r44	N/A	N/A
10539963	Vmn1r46	N/A	N/A
10539965	Vmn1r47	N/A	N/A
10539967	Vmn1r50	N/A	N/A
10539973	Vmn1r48 /// Vmn1r45	N/A	N/A
10539975	Vmn1r52	N/A	N/A
10539977	V1ra8	N/A	N/A
10539979	---	N/A	N/A
10539981	Vmn1r54	N/A	N/A
10539990	Uroc1	N/A	N/A
10540012	Zxdc	N/A	N/A
10540034	Aldh1l1	N/A	N/A
10540059	Slc41a3	N/A	N/A
10540072	Ppp1r2	N/A	N/A
10540075	Hdac11	N/A	N/A
10540105	Tmem43	N/A	N/A
10540118	Lsm3	N/A	N/A
10540122	Slc6a6	N/A	N/A
10540141	C130022K22Rik	N/A	N/A
10540191	Nr2c2	N/A	N/A
10540213	---	N/A	N/A
10540215	Slc25a26	N/A	N/A
10540227	Kbtbd8	N/A	N/A
10540231	---	N/A	N/A
10540241	Arl6ip5	N/A	N/A
10540265	Mitf	N/A	N/A
10540267	---	N/A	N/A
10540269	Gpr27	N/A	N/A
10540271	---	N/A	N/A
10540273	Ube2v2	N/A	N/A
10540283	Ppp4r2	N/A	N/A
10540287	Gm10009	N/A	N/A
10540298	Chl1	N/A	N/A
10540333	Cntn6	N/A	N/A
10540359	Cntn4	N/A	N/A
10540391	Trnt1 /// Crbn	N/A	N/A
10540401	Lrrn1	N/A	N/A
10540405	Setmar	N/A	N/A
10540408	Itpr1	N/A	N/A
10540493	Edem1	N/A	N/A
10540507	H3f3a	N/A	N/A
10540531	LOC280487	N/A	N/A
10540537	---	N/A	N/A
10540540	0610010K06Rik	N/A	N/A
10540542	LOC100503669	N/A	N/A
10540544	Thumpd3	N/A	N/A
10540554	Setd5	N/A	N/A
10540579	Mtmr14	N/A	N/A
10540650	Arpc4 /// Ttll3	N/A	N/A
10540659	Ttll3 /// Arpc4	N/A	N/A
10540676	Jagn1	N/A	N/A
10540727	Creld1	N/A	N/A
10540785	6720456B07Rik	N/A	N/A
10540790	Vhl	N/A	N/A
10540795	Irak2	N/A	N/A
10540812	Tatdn2	N/A	N/A
10540822	Slc6a11	N/A	N/A
10540855	Hrh1	N/A	N/A
10540860	Atg7 /// LOC100043926	N/A	N/A
10540880	Syn2	N/A	N/A
10540911	Tsen2	N/A	N/A
10540923	Mkrn2	N/A	N/A
10540931	Raf1 /// D830050J10Rik	N/A	N/A
10540991	Rho	N/A	N/A
10540999	H2afz	N/A	N/A
10541002	D6Wsu116e	N/A	N/A
10541034	Anubl1	N/A	N/A
10541049	March8	N/A	N/A
10541061	Olfr211	N/A	N/A
10541063	Olfr212	N/A	N/A
10541065	Olfr213	N/A	N/A
10541067	Eif4a3 /// Gm8994	N/A	N/A
10541069	Olfr214	N/A	N/A
10541075	Cxcl12	N/A	N/A
10541089	2900097C17Rik /// Gm7292	N/A	N/A
10541091	Gemin6	N/A	N/A
10541094	Zfp637	N/A	N/A
10541098	Zfp239	N/A	N/A
10541104	Hnrnpf	N/A	N/A
10541112	Lasp1	N/A	N/A

10541114	Rasgef1a	N/A	N/A
10541127	---	N/A	N/A
10541129	LOC280487	N/A	N/A
10541182	Fbxl14	N/A	N/A
10541184	---	N/A	N/A
10541186	3110021A11Rik	N/A	N/A
10541188	---	N/A	N/A
10541214	B4galnt3	N/A	N/A
10541216	Kdm5a	N/A	N/A
10541246	Il17ra	N/A	N/A
10541260	Cecr2	N/A	N/A
10541268	Slc25a18	N/A	N/A
10541279	Bcl2l13	N/A	N/A
10541288	Gm9878	N/A	N/A
10541301	Tuba8	N/A	N/A
10541307	Usp18	N/A	N/A
10541318	Slc6a13	N/A	N/A
10541333	Slc6a12	N/A	N/A
10541349	---	N/A	N/A
10541410	Mug1	N/A	N/A
10541441	Gm10319	N/A	N/A
10541446	Mug2	N/A	N/A
10541448	Mug-ps1 /// Mug2 /// Mug1	N/A	N/A
10541480	Mug-ps1 /// Mug1	N/A	N/A
10541482	Nhp2l1	N/A	N/A
10541484	M6pr /// M6pr-ps	N/A	N/A
10541494	Rps27a	N/A	N/A
10541513	---	N/A	N/A
10541520	Rps15	N/A	N/A
10541522	Rps2 /// Rps2-ps6 /// Gm5921 /// Gm6139	N/A	N/A
10541532	Foxj2	N/A	N/A
10541545	Necap1	N/A	N/A
10541625	---	N/A	N/A
10541627	---	N/A	N/A
10541632	Vmn2r23	N/A	N/A
10541634	Vmn2r24	N/A	N/A
10541642	---	N/A	N/A
10541670	C1rl	N/A	N/A
10541678	C1ra	N/A	N/A
10541683	C1ra /// C1rb	N/A	N/A
10541695	Lpcat3	N/A	N/A
10541711	Phb2	N/A	N/A
10541721	Spsb2	N/A	N/A
10541738	A230083G16Rik	N/A	N/A
10541741	Mlf2	N/A	N/A
10541751	---	N/A	N/A
10541753	C530028O21Rik	N/A	N/A
10541762	Zfp384	N/A	N/A
10541771	Ing4	N/A	N/A
10541799	Lpar5	N/A	N/A
10541803	Chd4	N/A	N/A
10541845	Nop2	N/A	N/A
10541862	Iffo1	N/A	N/A
10541873	Mrpl51	N/A	N/A
10541877	Vamp1	N/A	N/A
10541885	Scnn1a	N/A	N/A
10541895	Tnfrsf1a	N/A	N/A
10542006	D6Wsu163e	N/A	N/A
10542021	---	N/A	N/A
10542034	---	N/A	N/A
10542036	Gm10414	N/A	N/A
10542038	Hmgb1	N/A	N/A
10542040	Parp11	N/A	N/A
10542050	Efcab4b	N/A	N/A
10542060	---	N/A	N/A
10542077	Rpl18	N/A	N/A
10542093	Nrip2	N/A	N/A
10542104	Gm10069	N/A	N/A
10542108	Tom1	N/A	N/A
10542112	Clec2h	N/A	N/A
10542156	Clec2d	N/A	N/A
10542164	Clec12a	N/A	N/A
10542172	Clec1b	N/A	N/A
10542200	Gabarapl1	N/A	N/A
10542221	Mir680-1	N/A	N/A
10542229	---	N/A	N/A
10542237	---	N/A	N/A
10542239	Prp2	N/A	N/A
10542241	Tas2r120	N/A	N/A
10542245	Tas2r102	N/A	N/A
10542249	Tas2r123	N/A	N/A
10542251	Tas2r116	N/A	N/A
10542253	Tas2r110	N/A	N/A
10542255	Tas2r113	N/A	N/A

10542257	Tas2r125	N/A	N/A
10542264	2700089E24Rik	N/A	N/A
10542275	Etv6	N/A	N/A
10542297	Loh12cr1	N/A	N/A
10542302	Crebl2	N/A	N/A
10542317	Cdkn1b	N/A	N/A
10542319	Apold1	N/A	N/A
10542321	Ddx47	N/A	N/A
10542340	8430419L09Rik	N/A	N/A
10542365	---	N/A	N/A
10542367	---	N/A	N/A
10542369	Gm8994	N/A	N/A
10542371	---	N/A	N/A
10542374	---	N/A	N/A
10542376	1110034A24Rik /// Rpl36al	N/A	N/A
10542395	Atf7ip	N/A	N/A
10542414	Ptpro	N/A	N/A
10542445	Strap	N/A	N/A
10542460	Dera	N/A	N/A
10542470	Mgst1	N/A	N/A
10542518	---	N/A	N/A
10542520	Atp5h /// Gm4953 /// Gm5048	N/A	N/A
10542555	MGC7817	N/A	N/A
10542573	BC005705	N/A	N/A
10542592	Gm10400	N/A	N/A
10542594	Gm10210	N/A	N/A
10542596	Slco1c1	N/A	N/A
10542615	Slco1b2	N/A	N/A
10542636	Pyroxd1 /// Recql	N/A	N/A
10542650	Golt1b	N/A	N/A
10542656	B230216G23Rik	N/A	N/A
10542665	Cmas	N/A	N/A
10542674	---	N/A	N/A
10542677	Etnk1	N/A	N/A
10542714	Lyrm5 /// Kras	N/A	N/A
10542722	Rps25 /// Gm4963	N/A	N/A
10542731	Rassf8	N/A	N/A
10542738	Rassf8	N/A	N/A
10542740	Sspn	N/A	N/A
10542745	Fgfr1op2	N/A	N/A
10542750	Med21	N/A	N/A
10542757	Stk38l	N/A	N/A
10542762	---	N/A	N/A
10542764	Arntl2	N/A	N/A
10542824	Mrps35	N/A	N/A
10542834	Gm5887	N/A	N/A
10542836	Klhdc5	N/A	N/A
10542840	E330012B07Rik	N/A	N/A
10542857	Far2	N/A	N/A
10542875	3010003L21Rik	N/A	N/A
10542878	---	N/A	N/A
10542880	4833442J19Rik	N/A	N/A
10542885	2810474O19Rik	N/A	N/A
10542892	Nacc1	N/A	N/A
10542894	---	N/A	N/A
10542896	Bicd1	N/A	N/A
10542911	Samd9l	N/A	N/A
10542929	Calcr	N/A	N/A
10542951	Mir489	N/A	N/A
10542953	Tfpi2	N/A	N/A
10542959	Bet1	N/A	N/A
10542965	Sgce	N/A	N/A
10542981	Gmfg	N/A	N/A
10542983	Pon1	N/A	N/A
10542993	Pon3	N/A	N/A
10543004	Pon2	N/A	N/A
10543029	LOC280487	N/A	N/A
10543031	Slc25a13	N/A	N/A
10543052	Rps27a	N/A	N/A
10543054	Shfm1	N/A	N/A
10543058	Dlx5	N/A	N/A
10543067	Asns	N/A	N/A
10543080	Rnps1	N/A	N/A
10543118	Glcci1	N/A	N/A
10543120	Ica1	N/A	N/A
10543134	Ndufa4	N/A	N/A
10543145	Thsd7a	N/A	N/A
10543213	B630005N14Rik	N/A	N/A
10543226	2610001J05Rik	N/A	N/A
10543231	---	N/A	N/A
10543302	---	N/A	N/A
10543304	---	N/A	N/A
10543306	Tspan12	N/A	N/A
10543317	Rpl21	N/A	N/A

10543333	Aass	N/A	N/A
10543358	---	N/A	N/A
10543360	---	N/A	N/A
10543362	Fezf1	N/A	N/A
10543402	Rnf133	N/A	N/A
10543407	Rnf148	N/A	N/A
10543409	Tas2r118	N/A	N/A
10543448	Wasl	N/A	N/A
10543460	Tmem229a	N/A	N/A
10543464	Rpl7a	N/A	N/A
10543471	Pot1a	N/A	N/A
10543492	---	N/A	N/A
10543494	Grm8	N/A	N/A
10543510	Mir592	N/A	N/A
10543522	---	N/A	N/A
10543524	Gcc1 /// Fscn3	N/A	N/A
10543544	Lrrc4	N/A	N/A
10543549	---	N/A	N/A
10543572	Impdh1	N/A	N/A
10543650	Tnpo3	N/A	N/A
10543676	1700080G18Rik	N/A	N/A
10543678	---	N/A	N/A
10543682	Mir96	N/A	N/A
10543695	---	N/A	N/A
10543697	Zc3hc1	N/A	N/A
10543725	Tsga14	N/A	N/A
10543737	Copg2	N/A	N/A
10543781	Mir29b-1	N/A	N/A
10543783	Psma3	N/A	N/A
10543785	AB041803	N/A	N/A
10543802	Plxna4	N/A	N/A
10543835	Chchd3	N/A	N/A
10543846	Slc35b4	N/A	N/A
10543859	Akr1b3 /// Gm6644	N/A	N/A
10543870	Npn2	N/A	N/A
10543872	3110062M04Rik /// Tmem140	N/A	N/A
10543904	Cnot4	N/A	N/A
10543944	Mtpn	N/A	N/A
10543967	Dgki	N/A	N/A
10544002	Creb3l2	N/A	N/A
10544015	Ybx1	N/A	N/A
10544062	D630045J12Rik	N/A	N/A
10544084	Zc3hav1l	N/A	N/A
10544089	Zc3hav1	N/A	N/A
10544106	---	N/A	N/A
10544114	Hipk2	N/A	N/A
10544133	Parp12	N/A	N/A
10544148	Jhdm1d	N/A	N/A
10544150	Jhdm1d	N/A	N/A
10544171	Slc37a3	N/A	N/A
10544186	Mkrn1	N/A	N/A
10544197	---	N/A	N/A
10544199	Dennd2a	N/A	N/A
10544219	Braf	N/A	N/A
10544248	---	N/A	N/A
10544252	E330009J07Rik	N/A	N/A
10544271	Olfr461	N/A	N/A
10544284	Tas2r138	N/A	N/A
10544406	Fam131b	N/A	N/A
10544417	Epha1	N/A	N/A
10544436	Olfr458	N/A	N/A
10544438	Olfr457 /// Olfr456 /// Olfr458	N/A	N/A
10544440	Olfr457	N/A	N/A
10544442	Olfr455	N/A	N/A
10544462	Fam115a	N/A	N/A
10544497	Tarbp2	N/A	N/A
10544499	Cul1	N/A	N/A
10544501	Ezh2	N/A	N/A
10544523	Rny1	N/A	N/A
10544525	Pdia4 /// Mir704	N/A	N/A
10544538	---	N/A	N/A
10544540	Rpl35a /// Ino80 /// Gm10247	N/A	N/A
10544547	Zfp777	N/A	N/A
10544555	Zfp746	N/A	N/A
10544570	Pcnp	N/A	N/A
10544573	Lrrc61 /// Rarres2	N/A	N/A
10544583	Gimap6	N/A	N/A
10544596	Tmem176b	N/A	N/A
10544608	---	N/A	N/A
10544629	Tra2a	N/A	N/A
10544638	Tra2a	N/A	N/A
10544640	Tra2a	N/A	N/A
10544642	Gm10464	N/A	N/A
10544644	Dfna5	N/A	N/A

10544687	Cycs	N/A	N/A
10544702	---	N/A	N/A
10544720	Hnrnpa2b1	N/A	N/A
10544774	Hoxa6	N/A	N/A
10544795	Mir196b	N/A	N/A
10544798	Hoxa10	N/A	N/A
10544812	Rpl38	N/A	N/A
10544815	Hibadh	N/A	N/A
10544823	---	N/A	N/A
10544827	---	N/A	N/A
10544829	Jazf1	N/A	N/A
10544837	1200009O22Rik /// Gm16499	N/A	N/A
10544855	Rpl29	N/A	N/A
10544875	Scrn1	N/A	N/A
10544906	Ggct	N/A	N/A
10544932	Inmt	N/A	N/A
10544939	---	N/A	N/A
10544941	Pde1c	N/A	N/A
10544971	Lsm5	N/A	N/A
10544976	Kbtbd2	N/A	N/A
10544982	Nt5c3	N/A	N/A
10544993	Vmn1r8 /// Vmn1r7	N/A	N/A
10544999	Vmn1r18	N/A	N/A
10545001	Ppm1k	N/A	N/A
10545009	Pigy	N/A	N/A
10545014	Vopp1	N/A	N/A
10545028	Vmn1r24	N/A	N/A
10545030	Vmn1r25	N/A	N/A
10545032	Vmn1r26	N/A	N/A
10545034	---	N/A	N/A
10545036	Vmn1r27	N/A	N/A
10545041	Nap1l5	N/A	N/A
10545045	Fam13a	N/A	N/A
10545065	Gprin3	N/A	N/A
10545075	---	N/A	N/A
10545096	Mageb16 /// Mageb16-ps1	N/A	N/A
10545099	---	N/A	N/A
10545101	Hpgds	N/A	N/A
10545117	Vmn1r35 /// Vmn1r37 /// Vmn1r36 /// Vmn1r34	N/A	N/A
10545123	Vmn1r38	N/A	N/A
10545125	Rpl23	N/A	N/A
10545127	Gng12	N/A	N/A
10545130	Gadd45a	N/A	N/A
10545173	LOC672291	N/A	N/A
10545175	LOC672291 /// Igk-J1	N/A	N/A
10545177	---	N/A	N/A
10545182	Gm459	N/A	N/A
10545184	Gm10880	N/A	N/A
10545187	Gm1502 /// Gm8760 /// Igkv4-71	N/A	N/A
10545192	Rprl1	N/A	N/A
10545194	Gm8760 /// Igkv4-71	N/A	N/A
10545196	Igk-C /// Gm1419 /// Gm10880 /// Igkv4-71 /// Gm1524	N/A	N/A
10545198	Gm8760 /// Gm1499 /// Igkv4-71	N/A	N/A
10545200	---	N/A	N/A
10545208	Gm189	N/A	N/A
10545226	---	N/A	N/A
10545229	U29423	N/A	N/A
10545231	---	N/A	N/A
10545233	Gm10883	N/A	N/A
10545255	Rpia	N/A	N/A
10545271	Thnsl2	N/A	N/A
10545298	Rmnd5a	N/A	N/A
10545337	Ppia	N/A	N/A
10545339	Mrpl35	N/A	N/A
10545346	Ptcd3	N/A	N/A
10545372	Atoh8	N/A	N/A
10545379	Usp39	N/A	N/A
10545394	Rnf181	N/A	N/A
10545409	Vamp8	N/A	N/A
10545417	Mat2a	N/A	N/A
10545436	Elmod3	N/A	N/A
10545458	Tcf7l1	N/A	N/A
10545471	Kcmf1	N/A	N/A
10545479	Tmsb10	N/A	N/A
10545502	Dnahc6	N/A	N/A
10545513	---	N/A	N/A
10545528	Pigp	N/A	N/A
10545530	Gm10450	N/A	N/A
10545534	Rnf26	N/A	N/A
10545538	Ctnna2	N/A	N/A
10545576	Mrpl19 /// AW146020	N/A	N/A
10545583	Pole4	N/A	N/A
10545608	Sema4f	N/A	N/A
10545629	Htra2 /// Loxl3	N/A	N/A

10545644	Wbp1	N/A	N/A
10545651	Ino80b	N/A	N/A
10545658	Wdr54 /// Rtkn	N/A	N/A
10545672	Mthfd2	N/A	N/A
10545682	Tet3	N/A	N/A
10545692	Tet3	N/A	N/A
10545697	Dguok	N/A	N/A
10545720	Stambp	N/A	N/A
10545751	Tex261	N/A	N/A
10545758	---	N/A	N/A
10545760	Paip2b	N/A	N/A
10545765	Gapdh /// Gm16374 /// Gm2606 /// Gm4609 /// Gm3200 /// Gm2451 /// Gm10293	N/A	N/A
10545768	Gm10445	N/A	N/A
10545780	Exoc6b	N/A	N/A
10545812	Sfxn5	N/A	N/A
10545827	Rab11fip5	N/A	N/A
10545862	Cml3	N/A	N/A
10545865	Cml3	N/A	N/A
10545874	Cml3 /// Cml5	N/A	N/A
10545881	Cml2	N/A	N/A
10545891	Cml1	N/A	N/A
10545895	Nat8b	N/A	N/A
10545910	Pcyox1	N/A	N/A
10545917	Pcbp1	N/A	N/A
10545921	Mxd1	N/A	N/A
10545930	---	N/A	N/A
10545932	Snrnp27	N/A	N/A
10545940	Gmcl1	N/A	N/A
10545974	Antxr1	N/A	N/A
10546047	LOC100503261	N/A	N/A
10546054	Rpl3	N/A	N/A
10546056	Rab43	N/A	N/A
10546066	Isy1	N/A	N/A
10546086	---	N/A	N/A
10546088	H1fx	N/A	N/A
10546092	Rab7	N/A	N/A
10546104	Eefsec	N/A	N/A
10546113	Sec61a1	N/A	N/A
10546137	Abtb1	N/A	N/A
10546152	Podxl2	N/A	N/A
10546184	Plxna1	N/A	N/A
10546217	Chchd6	N/A	N/A
10546227	---	N/A	N/A
10546229	---	N/A	N/A
10546231	Vmn1r42	N/A	N/A
10546233	Vmn1r43	N/A	N/A
10546238	Vmn1r48	N/A	N/A
10546240	Vmn1r49	N/A	N/A
10546242	Vmn1r53	N/A	N/A
10546272	Iqsec1	N/A	N/A
10546292	Rpl21 /// Rpl21-ps4 /// Rpl21-ps12 /// Gm9104 /// Rpl21-ps14 /// Rpl21-ps10	N/A	N/A
10546337	---	N/A	N/A
10546396	Mrps25	N/A	N/A
10546402	Zfyve20	N/A	N/A
10546417	Trh	N/A	N/A
10546421	Prickle2	N/A	N/A
10546430	Adamts9	N/A	N/A
10546432	Adamts9	N/A	N/A
10546450	Adamts9	N/A	N/A
10546452	Adamts9	N/A	N/A
10546476	Magi1	N/A	N/A
10546508	---	N/A	N/A
10546510	Lrig1	N/A	N/A
10546533	---	N/A	N/A
10546538	Suclg2	N/A	N/A
10546551	1700123L14Rik	N/A	N/A
10546567	A130022J15Rik	N/A	N/A
10546586	Tmf1	N/A	N/A
10546606	Uba3	N/A	N/A
10546631	Frmd4b	N/A	N/A
10546657	---	N/A	N/A
10546659	---	N/A	N/A
10546685	Eif4e3	N/A	N/A
10546695	Prok2	N/A	N/A
10546702	Tpt1 /// Tpt1p	N/A	N/A
10546706	Rybp	N/A	N/A
10546710	Shq1	N/A	N/A
10546736	Cntn3	N/A	N/A
10546760	Ddx3x	N/A	N/A
10546775	Crbn	N/A	N/A
10546791	Sumf1	N/A	N/A
10546801	Rpl36 /// Gm13611	N/A	N/A
10546803	Gm10429 /// Itpr1	N/A	N/A
10546805	Ddx18	N/A	N/A

10546807	---	N/A	N/A
10546829	Oxtr	N/A	N/A
10546832	---	N/A	N/A
10546853	Srgap3	N/A	N/A
10546855	Srgap3	N/A	N/A
10546884	Lhfpl4	N/A	N/A
10546889	---	N/A	N/A
10546891	Camk1	N/A	N/A
10546919	Rpusd3	N/A	N/A
10546944	Tmem111	N/A	N/A
10546960	---	N/A	N/A
10546962	Ghrl	N/A	N/A
10546967	Sec13	N/A	N/A
10546977	Atp2b2	N/A	N/A
10547009	Vgll4	N/A	N/A
10547015	1500001M20Rik	N/A	N/A
10547022	Timp4	N/A	N/A
10547030	2510049J12Rik	N/A	N/A
10547054	---	N/A	N/A
10547073	Snora7a	N/A	N/A
10547075	BC060267	N/A	N/A
10547088	Mbd4 /// Ift122	N/A	N/A
10547140	Tmcc1	N/A	N/A
10547151	Rpl27a /// Gm5908	N/A	N/A
10547171	Olfr215	N/A	N/A
10547173	Zfp422	N/A	N/A
10547200	---	N/A	N/A
10547202	---	N/A	N/A
10547204	Hnrnpf /// 4933440N22Rik	N/A	N/A
10547206	Fxyd4	N/A	N/A
10547217	Csgalnact2	N/A	N/A
10547251	Bms1	N/A	N/A
10547274	Zfp248	N/A	N/A
10547282	Zfp9	N/A	N/A
10547288	Ankrd26	N/A	N/A
10547322	Cacna1c	N/A	N/A
10547381	Lrtm2	N/A	N/A
10547386	Adipor2	N/A	N/A
10547396	Wnt5b	N/A	N/A
10547404	Erc1	N/A	N/A
10547408	Erc1	N/A	N/A
10547410	Erc1	N/A	N/A
10547436	Wnk1	N/A	N/A
10547469	Wnk1	N/A	N/A
10547513	Cecr5	N/A	N/A
10547521	Atp6v1e1	N/A	N/A
10547531	Bid	N/A	N/A
10547540	Mical3	N/A	N/A
10547553	Mical3	N/A	N/A
10547575	Iqsec3	N/A	N/A
10547597	Phc1	N/A	N/A
10547613	Rimklb	N/A	N/A
10547641	Slc2a3	N/A	N/A
10547655	---	N/A	N/A
10547662	Gm5316	N/A	N/A
10547674	Vmn2r22	N/A	N/A
10547682	Vmn2r22	N/A	N/A
10547684	Vmn2r22	N/A	N/A
10547689	Vmn2r26	N/A	N/A
10547695	Vmn2r27	N/A	N/A
10547701	Pex5	N/A	N/A
10547719	Clstn3	N/A	N/A
10547752	Gm5077	N/A	N/A
10547758	Emg1 /// Lpcat3	N/A	N/A
10547765	Mir141	N/A	N/A
10547767	Mir200c	N/A	N/A
10547789	Grcc10 /// Rnu7 /// Atn1	N/A	N/A
10547795	Atn1 /// Rnu7	N/A	N/A
10547807	Eno2	N/A	N/A
10547830	Tpi1	N/A	N/A
10547837	Usp5	N/A	N/A
10547888	Gpr162	N/A	N/A
10547916	Ptms	N/A	N/A
10547924	---	N/A	N/A
10547926	Cops7a	N/A	N/A
10547936	Gapdh /// Gm2606	N/A	N/A
10547976	Tapbpl	N/A	N/A
10547993	---	N/A	N/A
10548000	Ltbr	N/A	N/A
10548011	Plekhg6	N/A	N/A
10548030	Cd9	N/A	N/A
10548038	Ntf3	N/A	N/A
10548041	Gm10415	N/A	N/A
10548043	Kcna5	N/A	N/A

10548047	Kcna1	N/A	N/A
10548051	Kcna6	N/A	N/A
10548057	Ndufa9	N/A	N/A
10548105	Ccnd2	N/A	N/A
10548116	---	N/A	N/A
10548118	Prmt8	N/A	N/A
10548128	Tspan9	N/A	N/A
10548139	---	N/A	N/A
10548141	Gm10010	N/A	N/A
10548143	Gapdh /// Gm16374 /// Gm4609 /// Gm3200 /// Gm2451 /// Gm10293	N/A	N/A
10548176	5930416I19Rik	N/A	N/A
10548180	Itfg2	N/A	N/A
10548194	Fkbp4	N/A	N/A
10548207	Pzp	N/A	N/A
10548244	---	N/A	N/A
10548246	Rpl21	N/A	N/A
10548563	Ptp4a1	N/A	N/A
10548598	---	N/A	N/A
10548600	Hmgb1 /// Hmgb1l	N/A	N/A
10548606	Tas2r107	N/A	N/A
10548610	Tas2r104	N/A	N/A
10548612	Tas2r105	N/A	N/A
10548614	Tas2r114	N/A	N/A
10548661	---	N/A	N/A
10548663	Tas2r121	N/A	N/A
10548665	Tas2r115	N/A	N/A
10548669	Tas2r131	N/A	N/A
10548671	Tas2r109	N/A	N/A
10548673	Tas2r117	N/A	N/A
10548675	Tas2r103	N/A	N/A
10548677	Tas2r140	N/A	N/A
10548679	---	N/A	N/A
10548681	---	N/A	N/A
10548684	---	N/A	N/A
10548697	---	N/A	N/A
10548699	---	N/A	N/A
10548701	Lrp6	N/A	N/A
10548727	D230041D01Rik /// LOC100502769	N/A	N/A
10548735	Dusp16	N/A	N/A
10548752	---	N/A	N/A
10548761	Hebp1	N/A	N/A
10548785	Dynlt1a /// Dynlt1b /// Dynlt1c /// Dynlt1f	N/A	N/A
10548788	---	N/A	N/A
10548791	Grin2b	N/A	N/A
10548808	---	N/A	N/A
10548815	Coq2	N/A	N/A
10548817	Plbd1	N/A	N/A
10548857	Hist4h4	N/A	N/A
10548859	Wbp11	N/A	N/A
10548875	Art4	N/A	N/A
10548879	Mgp	N/A	N/A
10548905	Eps8	N/A	N/A
10548940	Lmo3	N/A	N/A
10548948	Igbp1b	N/A	N/A
10548976	Rpl38	N/A	N/A
10548978	Slco1a1	N/A	N/A
10548996	Slco1a4	N/A	N/A
10549079	Gys2	N/A	N/A
10549162	St8sia1	N/A	N/A
10549171	5730419I09Rik	N/A	N/A
10549219	---	N/A	N/A
10549222	Bcat1	N/A	N/A
10549276	Bhlhe41	N/A	N/A
10549282	Itpr2	N/A	N/A
10549341	4933424B01Rik	N/A	N/A
10549361	Tm7sf3	N/A	N/A
10549375	Rps26 /// Gm6654	N/A	N/A
10549402	Ergic2 /// Far2	N/A	N/A
10549420	Tmtc1	N/A	N/A
10549445	---	N/A	N/A
10549447	Ipo8	N/A	N/A
10549495	Rps29	N/A	N/A
10549497	Fam60a	N/A	N/A
10549504	Dennd5b	N/A	N/A
10549506	Dennd5b	N/A	N/A
10549530	---	N/A	N/A
10549532	---	N/A	N/A
10549534	---	N/A	N/A
10549536	Amn1	N/A	N/A
10549544	Gm10388	N/A	N/A
10549552	Prpf31	N/A	N/A
10549569	Cnot3 /// Leng1	N/A	N/A
10549582	Tsen34	N/A	N/A
10549588	Rps9	N/A	N/A

10549592	---	N/A	N/A
10549594	Ttyh1	N/A	N/A
10549615	Leng8	N/A	N/A
10549633	Cdc42ep5	N/A	N/A
10549653	Atp6v0c	N/A	N/A
10549679	Brsk1 /// Tmem150b	N/A	N/A
10549700	Suv420h2	N/A	N/A
10549714	Rpl28	N/A	N/A
10549721	---	N/A	N/A
10549730	Nat14	N/A	N/A
10549748	Zfp524	N/A	N/A
10549758	4632433K11Rik /// Zfp784	N/A	N/A
10549760	Zfp580	N/A	N/A
10549770	U2af2	N/A	N/A
10549780	Epn1	N/A	N/A
10549802	Rps18 /// Gm5321 /// Gm10260	N/A	N/A
10549813	Zfp444	N/A	N/A
10549849	Zfp78	N/A	N/A
10549854	Zfp28	N/A	N/A
10549862	Olfr1344 /// Gm16532	N/A	N/A
10549871	Olfr1346	N/A	N/A
10549875	Olfr1350	N/A	N/A
10549877	---	N/A	N/A
10549879	Usp29 /// Zim3	N/A	N/A
10549899	Zfp418	N/A	N/A
10549917	Vmn2r43 /// Vmn2r35 /// Vmn2r33 /// Vmn2r39 /// Vmn2r50	N/A	N/A
10549921	Vmn2r43 /// Vmn2r35 /// Vmn2r39 /// Vmn2r50	N/A	N/A
10549932	2810047C21Rik1	N/A	N/A
10549938	Vmn2r42	N/A	N/A
10549945	Vmn1r67	N/A	N/A
10549947	Vmn1r70	N/A	N/A
10549962	---	N/A	N/A
10549964	Zscan4d /// Zscan4c /// Zscan4f /// Zscan4b /// Zscan4a /// Zscan4-ps2	N/A	N/A
10549972	Zscan4d /// Zscan4c /// Zscan4f /// Zscan4b /// Zscan4a /// Zscan4-ps2	N/A	N/A
10549976	---	N/A	N/A
10549979	---	N/A	N/A
10549984	Vmn1r74	N/A	N/A
10549986	Vmn1r75	N/A	N/A
10549990	Gm4741 /// V1rg10 /// Vmn1r2 /// Vmn1r3 /// Vmn1r238	N/A	N/A
10549996	Vmn1r82	N/A	N/A
10550003	Zfp606	N/A	N/A
10550029	Zfp110	N/A	N/A
10550039	Zfp128	N/A	N/A
10550059	2310014L17Rik	N/A	N/A
10550062	Zfp324	N/A	N/A
10550067	Zfp446	N/A	N/A
10550076	Trim28	N/A	N/A
10550098	Wdr12	N/A	N/A
10550100	Vmn1r89	N/A	N/A
10550161	---	N/A	N/A
10550167	---	N/A	N/A
10550172	Phf20	N/A	N/A
10550179	---	N/A	N/A
10550181	---	N/A	N/A
10550197	Obox1	N/A	N/A
10550200	---	N/A	N/A
10550237	Napa	N/A	N/A
10550274	Meis3	N/A	N/A
10550296	Zc3h4	N/A	N/A
10550316	Tmem160	N/A	N/A
10550320	---	N/A	N/A
10550345	Strn4	N/A	N/A
10550383	Dact3	N/A	N/A
10550400	Pnmal2	N/A	N/A
10550402	Pnmal1	N/A	N/A
10550451	---	N/A	N/A
10550482	Igfl3	N/A	N/A
10550494	---	N/A	N/A
10550509	Pglyrp1	N/A	N/A
10550514	Nova2	N/A	N/A
10550519	Nanos2	N/A	N/A
10550521	Mypop	N/A	N/A
10550527	Irf2bp1	N/A	N/A
10550531	Sympk	N/A	N/A
10550564	Dmwd	N/A	N/A
10550574	Dmpk	N/A	N/A
10550597	Fbxo46	N/A	N/A
10550601	Snrpd2 /// Gm10120	N/A	N/A
10550605	Eml2	N/A	N/A
10550625	Mir330	N/A	N/A
10550627	Gpr4	N/A	N/A
10550632	Opa3	N/A	N/A
10550636	---	N/A	N/A
10550650	Ercc1 /// Cd3eap	N/A	N/A

10550660	Ppp1r13l	N/A	N/A
10550674	Ercc2 /// Klc3	N/A	N/A
10550698	Ckm	N/A	N/A
10550730	Zfp296	N/A	N/A
10550734	Tomm40	N/A	N/A
10550738	Rpl7a-ps8	N/A	N/A
10550760	Vmn1r148	N/A	N/A
10550768	Vmn1r151	N/A	N/A
10550770	Vmn1r-ps79	N/A	N/A
10550772	Gm5156 /// Gm6882 /// Gm6176	N/A	N/A
10550782	Vmn1r148	N/A	N/A
10550805	Vmn1r177 /// Vmn1r168	N/A	N/A
10550824	Vmn1r180	N/A	N/A
10550826	Vmn1r181	N/A	N/A
10550831	Vmn1r183	N/A	N/A
10550833	Zfp180	N/A	N/A
10550847	Zfp235	N/A	N/A
10550860	Zfp108	N/A	N/A
10550865	Zfp93	N/A	N/A
10550915	Cadm4	N/A	N/A
10550931	Irgq	N/A	N/A
10550972	Phldb3	N/A	N/A
10550978	Phldb3	N/A	N/A
10550994	Ceacam10	N/A	N/A
10551009	Tmsb10	N/A	N/A
10551065	D930028M14Rik	N/A	N/A
10551077	Zfp526 /// 9130221H12Rik /// Gsk3a	N/A	N/A
10551080	Cic	N/A	N/A
10551102	Prr19	N/A	N/A
10551104	Tmem145	N/A	N/A
10551120	Megf8	N/A	N/A
10551155	Lipe /// 4732471J01Rik	N/A	N/A
10551162	Atp5sl	N/A	N/A
10551181	B9d2	N/A	N/A
10551195	---	N/A	N/A
10551207	---	N/A	N/A
10551215	Rnf170	N/A	N/A
10551226	Cyp2a5 /// Cyp2a4	N/A	N/A
10551250	---	N/A	N/A
10551282	Cyp2a5	N/A	N/A
10551287	Cyp2a12	N/A	N/A
10551293	Cyp2f2	N/A	N/A
10551314	BC024978	N/A	N/A
10551319	Adck4	N/A	N/A
10551336	Numbl	N/A	N/A
10551393	Akt2	N/A	N/A
10551401	Ttc9b	N/A	N/A
10551410	Zfp60	N/A	N/A
10551417	Zfp780b /// 1700049G17Rik /// Gm10046	N/A	N/A
10551421	Zfp60	N/A	N/A
10551423	4933426I21Rik	N/A	N/A
10551426	Zfp59	N/A	N/A
10551431	Zfp607	N/A	N/A
10551462	---	N/A	N/A
10551469	Dyrk1b	N/A	N/A
10551483	Eid2	N/A	N/A
10551487	Eid2b	N/A	N/A
10551489	Rps16 /// Rps16-ps2	N/A	N/A
10551496	Paf1	N/A	N/A
10551529	LOC100503763	N/A	N/A
10551531	Sycn	N/A	N/A
10551570	Sirt2	N/A	N/A
10551600	Hnrnpl	N/A	N/A
10551614	Ech1	N/A	N/A
10551714	---	N/A	N/A
10551716	Ggn	N/A	N/A
10551724	Yif1b	N/A	N/A
10551734	---	N/A	N/A
10551741	Dpf1	N/A	N/A
10551750	4932431P20Rik	N/A	N/A
10551752	4932431P20Rik	N/A	N/A
10551758	6330444E15Rik	N/A	N/A
10551760	Zfp84	N/A	N/A
10551770	Zfp30	N/A	N/A
10551777	Zfp790	N/A	N/A
10551801	C230062I16Rik	N/A	N/A
10551803	Zfp568	N/A	N/A
10551815	Zfp260	N/A	N/A
10551822	Zfp382	N/A	N/A
10551828	Gm5113	N/A	N/A
10551836	Cox7a1	N/A	N/A
10551841	Polr2i	N/A	N/A
10551852	Clip3	N/A	N/A
10551865	Alkbh6	N/A	N/A

10551872	AI428936	N/A	N/A
10551881	Sdhaf1 /// E130208F15Rik	N/A	N/A
10551905	Nphs1	N/A	N/A
10551939	Prodh2	N/A	N/A
10551966	Hspb6	N/A	N/A
10551971	U2af1l4 /// Tmem149	N/A	N/A
10551981	Tmem149 /// U2af1l4	N/A	N/A
10551989	Tmem149	N/A	N/A
10551996	---	N/A	N/A
10551998	---	N/A	N/A
10552006	Gm4883	N/A	N/A
10552071	Fam187b	N/A	N/A
10552088	Gm10640	N/A	N/A
10552094	Gm5326	N/A	N/A
10552100	---	N/A	N/A
10552104	Gm6666 /// Gm5116	N/A	N/A
10552106	---	N/A	N/A
10552108	---	N/A	N/A
10552118	LOC100504758	N/A	N/A
10552121	Dym /// Abpa	N/A	N/A
10552125	Pepd	N/A	N/A
10552140	Cebpa	N/A	N/A
10552143	Slc7a10 /// Lrp3	N/A	N/A
10552208	Nudt19	N/A	N/A
10552210	Ankrd27	N/A	N/A
10552240	E130304I02Rik /// Zfp507	N/A	N/A
10552242	Rpl17 /// Gm10268 /// Rpl17-ps3	N/A	N/A
10552245	Tshz3	N/A	N/A
10552249	---	N/A	N/A
10552252	1600014C10Rik	N/A	N/A
10552258	---	N/A	N/A
10552260	---	N/A	N/A
10552262	---	N/A	N/A
10552264	9430025M13Rik	N/A	N/A
10552276	Ube2h /// Gm2058	N/A	N/A
10552284	Pin4	N/A	N/A
10552288	Vstm2b	N/A	N/A
10552311	---	N/A	N/A
10552314	Zfp141	N/A	N/A
10552320	Zfp788	N/A	N/A
10552331	Gprc2a-rs5	N/A	N/A
10552335	Gprc2a-rs5 /// Vmn2r58 /// Vmn2r60 /// Vmn2r61 /// Vmn2r-ps60	N/A	N/A
10552339	Vmn2r60	N/A	N/A
10552341	Vmn2r60 /// Vmn2r61 /// Vmn2r59 /// Vmn2r-ps60	N/A	N/A
10552343	---	N/A	N/A
10552348	---	N/A	N/A
10552351	---	N/A	N/A
10552353	---	N/A	N/A
10552358	EU599041	N/A	N/A
10552363	EU599041	N/A	N/A
10552418	Etfb	N/A	N/A
10552440	Zfp719	N/A	N/A
10552458	Ctu1	N/A	N/A
10552613	Klk1b4	N/A	N/A
10552632	Shank1	N/A	N/A
10552656	Syt3	N/A	N/A
10552668	Lrrc4b	N/A	N/A
10552672	Aspdh	N/A	N/A
10552681	Josd2	N/A	N/A
10552725	Vrk3	N/A	N/A
10552740	Nup62	N/A	N/A
10552752	Akt1s1	N/A	N/A
10552758	Mir707	N/A	N/A
10552760	Pnkp	N/A	N/A
10552812	Irf3	N/A	N/A
10552824	Rras	N/A	N/A
10552843	Mir150	N/A	N/A
10552871	Pth2	N/A	N/A
10552929	Mtag2 /// Lin7b	N/A	N/A
10552942	Lhb	N/A	N/A
10552945	Gys1	N/A	N/A
10552964	Ftl2 /// Ftl1 /// Gm10252	N/A	N/A
10553015	Bcat2	N/A	N/A
10553080	Car11	N/A	N/A
10553115	Lmtk3	N/A	N/A
10553131	Kdelr1	N/A	N/A
10553140	Tmem143	N/A	N/A
10553163	Nomo1	N/A	N/A
10553197	Ush1c /// Gm9860	N/A	N/A
10553261	Kcnc1	N/A	N/A
10553274	Saa2	N/A	N/A
10553280	Gtf2h1	N/A	N/A
10553299	Ifitm2	N/A	N/A
10553301	Ldha	N/A	N/A

10553324	Tmem86a	N/A	N/A
10553334	Mrgprb8	N/A	N/A
10553336	Zdhhc13	N/A	N/A
10553399	---	N/A	N/A
10553401	F830223B06Rik	N/A	N/A
10553403	Htatip2	N/A	N/A
10553450	Nell1	N/A	N/A
10553471	4933405O20Rik	N/A	N/A
10553473	---	N/A	N/A
10553475	Rps27a	N/A	N/A
10553501	Slc17a6	N/A	N/A
10553516	Gapdh	N/A	N/A
10553533	Gm6181	N/A	N/A
10553537	Luzp2	N/A	N/A
10553555	---	N/A	N/A
10553559	Siglech	N/A	N/A
10553598	Cyfip1	N/A	N/A
10553635	Nipa1 /// A230056P14Rik	N/A	N/A
10553644	---	N/A	N/A
10553646	Herc2	N/A	N/A
10553741	---	N/A	N/A
10553769	Gabra5 /// Gm9962	N/A	N/A
10553773	Gabrb3	N/A	N/A
10553786	---	N/A	N/A
10553788	Atp10a	N/A	N/A
10553811	---	N/A	N/A
10553813	Ube3a	N/A	N/A
10553829	Fam100b /// Gm7367	N/A	N/A
10553831	Fam100b /// Gm7367	N/A	N/A
10553833	Ndn	N/A	N/A
10553835	Magel2	N/A	N/A
10553840	Atp5l	N/A	N/A
10553842	Otud7a	N/A	N/A
10553857	Hmgb1	N/A	N/A
10553859	---	N/A	N/A
10553895	Mir211	N/A	N/A
10553897	Mtmr10	N/A	N/A
10553913	Mcee	N/A	N/A
10553917	Apba2	N/A	N/A
10553956	Tm2d3 /// Tarsl2	N/A	N/A
10553967	Pcsk6	N/A	N/A
10553993	Snrpa1	N/A	N/A
10554005	H47	N/A	N/A
10554013	Chsy1	N/A	N/A
10554057	Adamts17	N/A	N/A
10554059	Gm10622	N/A	N/A
10554061	Adamts17	N/A	N/A
10554074	Adamts17	N/A	N/A
10554076	Lysmd4	N/A	N/A
10554081	Ttc23	N/A	N/A
10554094	Igf1r	N/A	N/A
10554118	Fam169b	N/A	N/A
10554127	Gm5334	N/A	N/A
10554129	B130024G19Rik	N/A	N/A
10554140	Zic4	N/A	N/A
10554142	2310037I24Rik	N/A	N/A
10554150	Rgma	N/A	N/A
10554156	Fam174b	N/A	N/A
10554160	Gm10618	N/A	N/A
10554162	---	N/A	N/A
10554164	---	N/A	N/A
10554166	Akap13	N/A	N/A
10554198	Klhl25	N/A	N/A
10554221	Gm9885	N/A	N/A
10554231	Mir7-2	N/A	N/A
10554269	Abhd2	N/A	N/A
10554321	Gm10616	N/A	N/A
10554323	Mir9-3	N/A	N/A
10554349	Kif7 /// 9330171B17Rik	N/A	N/A
10554370	Zfp710	N/A	N/A
10554375	Sema4b	N/A	N/A
10554392	D330012F22Rik	N/A	N/A
10554413	Ngrn	N/A	N/A
10554419	Vps33b	N/A	N/A
10554463	Hddc3	N/A	N/A
10554468	---	N/A	N/A
10554475	Zfp592	N/A	N/A
10554521	Pde8a	N/A	N/A
10554547	Mir1839	N/A	N/A
10554549	Whamm	N/A	N/A
10554574	Tm6sf1 /// Hdgfrp3	N/A	N/A
10554586	4833418N17Rik	N/A	N/A
10554629	Eftud1	N/A	N/A
10554655	Mex3b	N/A	N/A

10554693	Stard5	N/A	N/A
10554701	Hnrnpk	N/A	N/A
10554704	Mesdc2	N/A	N/A
10554710	---	N/A	N/A
10554712	---	N/A	N/A
10554714	Olfr291	N/A	N/A
10554716	Olfr290	N/A	N/A
10554721	Olfr301	N/A	N/A
10554727	Olfr297	N/A	N/A
10554729	Olfr295	N/A	N/A
10554743	---	N/A	N/A
10554778	Grm5	N/A	N/A
10554789	Ctsc	N/A	N/A
10554814	Prss23	N/A	N/A
10554817	Gm10291	N/A	N/A
10554819	Me3	N/A	N/A
10554837	Eed	N/A	N/A
10554839	Picalm	N/A	N/A
10554863	Sytl2	N/A	N/A
10554893	Ccdc89	N/A	N/A
10554895	Crebzf	N/A	N/A
10554900	Dlg2	N/A	N/A
10554926	Ccdc90b	N/A	N/A
10554938	Rab30	N/A	N/A
10554945	Prcp	N/A	N/A
10554958	Gm10607	N/A	N/A
10554960	Fam181b /// Gm9934	N/A	N/A
10554963	---	N/A	N/A
10554965	---	N/A	N/A
10554967	Gm9966	N/A	N/A
10554969	Odz4	N/A	N/A
10555007	---	N/A	N/A
10555009	Mir708	N/A	N/A
10555011	Nars2	N/A	N/A
10555027	Gab2	N/A	N/A
10555039	Kctd21	N/A	N/A
10555041	Alg8	N/A	N/A
10555055	Ndufc2	N/A	N/A
10555063	Ints4	N/A	N/A
10555087	---	N/A	N/A
10555089	Rsf1	N/A	N/A
10555108	Clns1a	N/A	N/A
10555116	Gm9990	N/A	N/A
10555118	Pak1	N/A	N/A
10555174	Lrrc32	N/A	N/A
10555177	---	N/A	N/A
10555179	Prkrir	N/A	N/A
10555197	Mtap6	N/A	N/A
10555233	---	N/A	N/A
10555235	Arrb1	N/A	N/A
10555254	Mir326	N/A	N/A
10555256	Gm10605	N/A	N/A
10555258	Olfr520	N/A	N/A
10555260	Olfr521	N/A	N/A
10555293	Lipt2	N/A	N/A
10555303	Pgm2l1	N/A	N/A
10555339	C2cd3	N/A	N/A
10555389	Ucp2	N/A	N/A
10555407	Chchd8	N/A	N/A
10555414	Rab6	N/A	N/A
10555425	Fam168a	N/A	N/A
10555438	Fchsd2	N/A	N/A
10555460	Stard10	N/A	N/A
10555510	Pde2a	N/A	N/A
10555548	Mir139	N/A	N/A
10555550	Clpb	N/A	N/A
10555568	Rpl31	N/A	N/A
10555574	Inppl1	N/A	N/A
10555586	2400001E08Rik	N/A	N/A
10555590	Numa1	N/A	N/A
10555671	Pgap2	N/A	N/A
10555681	Stim1	N/A	N/A
10555695	Rrm1	N/A	N/A
10555716	Olfr547	N/A	N/A
10555718	---	N/A	N/A
10555720	Olfr549	N/A	N/A
10555725	Olfr552	N/A	N/A
10555727	Olfr554	N/A	N/A
10555729	Olfr555	N/A	N/A
10555734	Olfr557	N/A	N/A
10555744	Olfr564	N/A	N/A
10555749	Olfr570	N/A	N/A
10555751	Olfr572	N/A	N/A
10555756	Olfr576	N/A	N/A

10555758	Olfr582	N/A	N/A
10555771	Olfr592	N/A	N/A
10555773	Olfr593	N/A	N/A
10555775	Olfr594	N/A	N/A
10555777	---	N/A	N/A
10555781	Olfr603 /// Olfr596	N/A	N/A
10555783	Olfr597	N/A	N/A
10555787	Olfr599	N/A	N/A
10555789	Usp17l5	N/A	N/A
10555791	Olfr606	N/A	N/A
10555793	Olfr608	N/A	N/A
10555799	Olfr615	N/A	N/A
10555801	Olfr617	N/A	N/A
10555803	Olfr618	N/A	N/A
10555805	Olfr619	N/A	N/A
10555807	Olfr617	N/A	N/A
10555818	---	N/A	N/A
10555832	Olfr632	N/A	N/A
10555834	Olfr633	N/A	N/A
10555836	---	N/A	N/A
10555838	Olfr635	N/A	N/A
10555840	Olfr638	N/A	N/A
10555842	Olfr641	N/A	N/A
10555844	Olfr646	N/A	N/A
10555846	---	N/A	N/A
10555870	Olfr651	N/A	N/A
10555873	Olfr652	N/A	N/A
10555875	Olfr653	N/A	N/A
10555877	Olfr654	N/A	N/A
10555879	Olfr656	N/A	N/A
10555881	Olfr657	N/A	N/A
10555883	Olfr659	N/A	N/A
10555885	---	N/A	N/A
10555887	Olfr661	N/A	N/A
10555892	Twf1	N/A	N/A
10555899	Olfr665	N/A	N/A
10555901	Olfr669	N/A	N/A
10555903	Olfr676	N/A	N/A
10555905	Olfr677	N/A	N/A
10555907	Olfr678	N/A	N/A
10555909	Olfr679	N/A	N/A
10555911	Olfr681 /// Olfr679	N/A	N/A
10555913	Olfr689 /// Olfr688 /// Olfr687	N/A	N/A
10555915	Olfr689 /// Olfr688 /// Olfr687	N/A	N/A
10555917	Olfr689	N/A	N/A
10555919	Olfr692	N/A	N/A
10555921	Gm5901	N/A	N/A
10555935	Cckbr	N/A	N/A
10555946	Smpd1	N/A	N/A
10556018	---	N/A	N/A
10556029	Olfr703	N/A	N/A
10556033	Olfr707 /// Olfr708	N/A	N/A
10556035	Olfr713	N/A	N/A
10556041	Olfr716	N/A	N/A
10556059	Rbmxl2	N/A	N/A
10556076	Olfml1	N/A	N/A
10556111	Olfr467	N/A	N/A
10556117	Olfr473	N/A	N/A
10556121	Olfr476	N/A	N/A
10556123	Olfr477	N/A	N/A
10556125	---	N/A	N/A
10556127	Olfr479	N/A	N/A
10556129	Olfr481	N/A	N/A
10556131	Olfr483	N/A	N/A
10556133	Styx	N/A	N/A
10556135	---	N/A	N/A
10556139	Olfr494	N/A	N/A
10556141	Olfr495	N/A	N/A
10556143	Olfr497	N/A	N/A
10556145	Olfr498	N/A	N/A
10556149	Olfr658 /// Olfr503	N/A	N/A
10556154	Olfr506	N/A	N/A
10556156	Olfr507	N/A	N/A
10556160	Olfr510	N/A	N/A
10556165	Olfr513	N/A	N/A
10556167	---	N/A	N/A
10556178	Tub /// Ric3	N/A	N/A
10556200	Rpl27a /// Gm5908 /// Rpl27a-ps1 /// Gm5453	N/A	N/A
10556206	Snora3	N/A	N/A
10556208	D930014E17Rik	N/A	N/A
10556214	---	N/A	N/A
10556216	Ipo7	N/A	N/A
10556242	---	N/A	N/A
10556246	Zfp143	N/A	N/A

10556295	---	N/A	N/A
10556302	Ampd3	N/A	N/A
10556350	Usp47	N/A	N/A
10556381	Mical2	N/A	N/A
10556426	Parva	N/A	N/A
10556442	Tead1	N/A	N/A
10556456	Tead1	N/A	N/A
10556509	Spon1	N/A	N/A
10556528	Pde3b	N/A	N/A
10556571	Rpl19	N/A	N/A
10556573	1110006G14Rik	N/A	N/A
10556576	1110004F10Rik	N/A	N/A
10556581	Gm10589	N/A	N/A
10556583	Nucb2	N/A	N/A
10556598	Xylt1	N/A	N/A
10556611	---	N/A	N/A
10556613	4930583K01Rik	N/A	N/A
10556658	9030624J02Rik	N/A	N/A
10556701	Acsm5	N/A	N/A
10556734	Acsm1	N/A	N/A
10556764	Ppp1cc	N/A	N/A
10556769	Acsm3 /// Eri2	N/A	N/A
10556812	Lyrm1	N/A	N/A
10556820	Tmem159	N/A	N/A
10556828	Anks4b	N/A	N/A
10556938	E130201H02Rik	N/A	N/A
10556940	Uqcrc2 /// Pdzd9	N/A	N/A
10556957	BC030336	N/A	N/A
10557009	Eef2k	N/A	N/A
10557033	Eef2k	N/A	N/A
10557035	Polr3e	N/A	N/A
10557058	Polr3e	N/A	N/A
10557106	Hs3st2	N/A	N/A
10557109	1700069B07Rik	N/A	N/A
10557177	Prkcb	N/A	N/A
10557201	Cacng3	N/A	N/A
10557206	Rbbp6	N/A	N/A
10557211	Rbbp6	N/A	N/A
10557213	Rbbp6	N/A	N/A
10557229	LOC100134980 /// LOC100504538	N/A	N/A
10557233	Tnrc6a	N/A	N/A
10557285	Lcmt1	N/A	N/A
10557308	Hs3st4	N/A	N/A
10557311	Gm16496	N/A	N/A
10557313	4930533L02Rik	N/A	N/A
10557315	4930571K23Rik	N/A	N/A
10557317	Jmjd5	N/A	N/A
10557326	Il4ra	N/A	N/A
10557353	D430042O09Rik	N/A	N/A
10557355	D430042O09Rik	N/A	N/A
10557357	D430042O09Rik	N/A	N/A
10557359	D430042O09Rik	N/A	N/A
10557361	D430042O09Rik	N/A	N/A
10557372	D430042O09Rik	N/A	N/A
10557374	D430042O09Rik	N/A	N/A
10557394	D430042O09Rik	N/A	N/A
10557397	D430042O09Rik	N/A	N/A
10557432	Eif3c	N/A	N/A
10557439	Ccdc101	N/A	N/A
10557450	Bola2	N/A	N/A
10557455	---	N/A	N/A
10557459	Mapk3	N/A	N/A
10557508	Doc2a /// Ino80e	N/A	N/A
10557519	Hirip3	N/A	N/A
10557528	Kctd13	N/A	N/A
10557535	Sez6l2	N/A	N/A
10557560	Cdipt	N/A	N/A
10557585	Zfp553	N/A	N/A
10557587	Zfp771	N/A	N/A
10557624	LOC100048345	N/A	N/A
10557626	---	N/A	N/A
10557628	Zfp689	N/A	N/A
10557631	Prr14	N/A	N/A
10557644	Fbrs	N/A	N/A
10557703	Snora30	N/A	N/A
10557705	Phkg2 /// Gm166	N/A	N/A
10557716	Rnf40	N/A	N/A
10557738	Ctf1	N/A	N/A
10557742	Fbxl19	N/A	N/A
10557754	Orai3	N/A	N/A
10557758	Setd1a	N/A	N/A
10557780	Setd1a	N/A	N/A
10557793	Stx4a	N/A	N/A
10557806	---	N/A	N/A

10557808	Zfp646	N/A	N/A
10557831	Myst1 /// Prss8	N/A	N/A
10557843	Fus	N/A	N/A
10557853	B230325K18Rik	N/A	N/A
10557925	Itgad	N/A	N/A
10557951	Armc5	N/A	N/A
10557992	Bag3	N/A	N/A
10558029	Sec23ip	N/A	N/A
10558049	Ppapdc1a	N/A	N/A
10558057	Wdr11	N/A	N/A
10558088	---	N/A	N/A
10558134	Plekha1	N/A	N/A
10558150	Htra1	N/A	N/A
10558220	Pstk	N/A	N/A
10558227	Acadsb	N/A	N/A
10558239	Hmx3	N/A	N/A
10558248	Bub3	N/A	N/A
10558257	Gpr26	N/A	N/A
10558263	Gm10584	N/A	N/A
10558265	Lhpp	N/A	N/A
10558274	Fam175b	N/A	N/A
10558285	Zranb1	N/A	N/A
10558295	Zranb1	N/A	N/A
10558297	2700050L05Rik	N/A	N/A
10558325	Bccip	N/A	N/A
10558345	Dock1	N/A	N/A
10558398	5430417C01Rik	N/A	N/A
10558403	4930544L04Rik	N/A	N/A
10558436	BC005624	N/A	N/A
10558439	Phgdh	N/A	N/A
10558468	Jakmip3	N/A	N/A
10558481	Dpysl4	N/A	N/A
10558515	Inpp5a	N/A	N/A
10558533	---	N/A	N/A
10558548	Kndc1	N/A	N/A
10558583	Zfp511	N/A	N/A
10558600	Mtg1	N/A	N/A
10558612	Olfr523	N/A	N/A
10558643	Olfr527	N/A	N/A
10558645	Olfr530	N/A	N/A
10558647	Olfr535	N/A	N/A
10558649	Olfr46 /// Olfr538 /// GA_x5J8B7TT63N-1148-873	N/A	N/A
10558653	Olfr46	N/A	N/A
10558655	Olfr46	N/A	N/A
10558657	Olfr46 /// Olfr538 /// GA_x5J8B7TT63N-1148-873	N/A	N/A
10558659	Olfr46	N/A	N/A
10558664	Olfr53	N/A	N/A
10558666	Olfr539	N/A	N/A
10558668	Olfr45	N/A	N/A
10558673	Cyp2e1	N/A	N/A
10558687	1190003J15Rik	N/A	N/A
10558707	Ric8	N/A	N/A
10558740	---	N/A	N/A
10558742	Nlrp6	N/A	N/A
10558754	Athl1	N/A	N/A
10558769	Ifitm1	N/A	N/A
10558773	B4galnt4	N/A	N/A
10558811	Ptdss2	N/A	N/A
10558840	Rassf7	N/A	N/A
10558847	Phrf1	N/A	N/A
10558872	Tmem80	N/A	N/A
10558880	Eps8l2 /// B230206H07Rik	N/A	N/A
10558903	Taldo1	N/A	N/A
10558910	Rangrf	N/A	N/A
10558914	Rplp2	N/A	N/A
10558921	Pnpla2	N/A	N/A
10558948	Cd151	N/A	N/A
10558971	Ap2a2	N/A	N/A
10559172	Krtap5-3	N/A	N/A
10559175	Krtap5-5 /// Krtap5-4	N/A	N/A
10559179	E330027M22Rik	N/A	N/A
10559200	Tnni2	N/A	N/A
10559233	Mrpl23	N/A	N/A
10559238	Igf2 /// Igf2as	N/A	N/A
10559261	Cd81	N/A	N/A
10559270	Tssc4	N/A	N/A
10559297	Slc22a18	N/A	N/A
10559310	---	N/A	N/A
10559312	Dhcr7	N/A	N/A
10559333	Shank2	N/A	N/A
10559341	Shank2	N/A	N/A
10559357	---	N/A	N/A
10559359	Gm10152	N/A	N/A
10559385	Mrgprf	N/A	N/A

10559389	Mrgprd	N/A	N/A
10559406	Tfpt	N/A	N/A
10559420	Tmc4	N/A	N/A
10559436	Mboat7	N/A	N/A
10559486	Lair1	N/A	N/A
10559498	9430041J12Rik	N/A	N/A
10559500	D030047H15Rik	N/A	N/A
10559504	Leng9	N/A	N/A
10559509	Cdc42ep5	N/A	N/A
10559513	Cab39	N/A	N/A
10559516	Rdh13	N/A	N/A
10559524	Ppp1r12c	N/A	N/A
10559568	6030429G01Rik /// Tnni3	N/A	N/A
10559580	Syt5	N/A	N/A
10559606	Tmem86b	N/A	N/A
10559635	Hspbp1	N/A	N/A
10559644	Brsk1	N/A	N/A
10559673	2210411K11Rik	N/A	N/A
10559676	Ube2s	N/A	N/A
10559681	Shisa7	N/A	N/A
10559687	Isoc2b	N/A	N/A
10559708	Fiz1	N/A	N/A
10559713	4632433K11Rik /// Zfp784	N/A	N/A
10559720	Rasl2-9-ps	N/A	N/A
10559728	---	N/A	N/A
10559743	Vmn1r56	N/A	N/A
10559754	Vmn1r64	N/A	N/A
10559762	---	N/A	N/A
10559766	Zfp583	N/A	N/A
10559782	Olfr5	N/A	N/A
10559784	Olfr1347	N/A	N/A
10559786	Olfr1348	N/A	N/A
10559788	Olfr1349	N/A	N/A
10559790	Zim1	N/A	N/A
10559796	Peg3	N/A	N/A
10559816	---	N/A	N/A
10559820	5730403M16Rik	N/A	N/A
10559825	Zfp773	N/A	N/A
10559837	Vmn2r29	N/A	N/A
10559853	Clcn4-2	N/A	N/A
10559871	Vmn2r42	N/A	N/A
10559878	2810047C21Rik1	N/A	N/A
10559883	Vmn2r42	N/A	N/A
10559890	Vmn2r43 /// Vmn2r35 /// Vmn2r39 /// Vmn2r50	N/A	N/A
10559894	Vmn2r37 /// Vmn2r51 /// Vmn2r48 /// Vmn2r34 /// Vmn2r35	N/A	N/A
10559901	Vmn2r42	N/A	N/A
10559908	Vmn2r43 /// Vmn2r29 /// Vmn2r35 /// Vmn2r33 /// Vmn2r39 /// Vmn2r50	N/A	N/A
10559916	---	N/A	N/A
10559919	Zfp772 /// 2810047C21Rik1	N/A	N/A
10559928	Vmn2r51 /// Vmn2r48 /// Vmn2r34 /// Vmn2r29	N/A	N/A
10559931	Vmn2r43 /// Vmn2r35 /// Vmn2r33 /// Vmn2r39 /// Vmn2r50	N/A	N/A
10559964	Zik1	N/A	N/A
10559983	---	N/A	N/A
10559996	---	N/A	N/A
10560000	Tpm3	N/A	N/A
10560013	Vmn1r76	N/A	N/A
10560019	Vmn1r83	N/A	N/A
10560021	Vmn1r84	N/A	N/A
10560023	Zfp551	N/A	N/A
10560028	Vmn2r54	N/A	N/A
10560035	Zscan18	N/A	N/A
10560043	Zfp329	N/A	N/A
10560045	Slc27a5	N/A	N/A
10560063	Chmp2a	N/A	N/A
10560070	Ube2m	N/A	N/A
10560087	Vmn1r85	N/A	N/A
10560089	Vmn1r86	N/A	N/A
10560094	---	N/A	N/A
10560097	6330408A02Rik /// Lig1	N/A	N/A
10560103	Rps8	N/A	N/A
10560131	2810007J24Rik	N/A	N/A
10560160	Obox5 /// Obox3 /// Gm4745 /// Gm5889	N/A	N/A
10560174	Sepw1	N/A	N/A
10560202	Gltscr1	N/A	N/A
10560260	Sae1	N/A	N/A
10560282	Grlf1	N/A	N/A
10560292	---	N/A	N/A
10560294	Ceacam15	N/A	N/A
10560298	---	N/A	N/A
10560300	Fkrp	N/A	N/A
10560304	Calm3	N/A	N/A
10560311	---	N/A	N/A
10560315	Ppp5c	N/A	N/A
10560354	Kctd20	N/A	N/A

10560356	---	N/A	N/A
10560399	---	N/A	N/A
10560429	Mypop /// Gm10676	N/A	N/A
10560431	Foxa3	N/A	N/A
10560434	Qpctl	N/A	N/A
10560487	Cd3eap	N/A	N/A
10560510	Mark4	N/A	N/A
10560528	---	N/A	N/A
10560530	Bloc1s3	N/A	N/A
10560532	Lrrc68	N/A	N/A
10560551	Clasrp	N/A	N/A
10560592	Clptm1	N/A	N/A
10560608	Apoc2	N/A	N/A
10560614	Apoc4 /// Apoc2	N/A	N/A
10560624	Apoe	N/A	N/A
10560630	Tomm40	N/A	N/A
10560655	Bcam	N/A	N/A
10560685	Bcl3	N/A	N/A
10560709	Pvr	N/A	N/A
10560726	---	N/A	N/A
10560728	Vmn1r122 /// Vmn1r101 /// Vmn1r158 /// Vmn1r113 /// Vmn1r117 /// Vmn1r129 /// Vmn1r149 /// Vmn1r119	N/A	N/A
10560742	Vmn1r151	N/A	N/A
10560744	Vmn1r158 /// Vmn1r113 /// Vmn1r117 /// Vmn1r129 /// Vmn1r119	N/A	N/A
10560752	Vmn1r148	N/A	N/A
10560758	Gm4141 /// Vmn1r111 /// Vmn1r118	N/A	N/A
10560760	Gm5891 /// Gm5156 /// Gm6882 /// Gm6176	N/A	N/A
10560764	Gm5156 /// Gm6882 /// Gm6176	N/A	N/A
10560771	Gm4141 /// Vmn1r111 /// Vmn1r118	N/A	N/A
10560773	Gm5891 /// Gm5156 /// Gm6882 /// Gm6176	N/A	N/A
10560777	Gm5156 /// Gm6882 /// Gm6176	N/A	N/A
10560780	Vmn1r122 /// Vmn1r101 /// Vmn1r158 /// Vmn1r113 /// Vmn1r117 /// Vmn1r129 /// Vmn1r149 /// Vmn1r119	N/A	N/A
10560789	Gm4141	N/A	N/A
10560795	Vmn1r101 /// Vmn1r158 /// Vmn1r113 /// Vmn1r117 /// Vmn1r129 /// Vmn1r149 /// Vmn1r119	N/A	N/A
10560801	Gm4141 /// Vmn1r111 /// Vmn1r118	N/A	N/A
10560803	Gm5891 /// Gm5156 /// Gm6882	N/A	N/A
10560807	Gm5156 /// Gm6882 /// Gm6176	N/A	N/A
10560810	Vmn1r171 /// Vmn1r170 /// Vmn1r175 /// Vmn1r169 /// Vmn1r176 /// Vmn1r167	N/A	N/A
10560814	Vmn1r177 /// Vmn1r168	N/A	N/A
10560816	Atp5g2	N/A	N/A
10560826	Zfp109	N/A	N/A
10560842	Zfp94	N/A	N/A
10560868	Zfp575	N/A	N/A
10560908	Gm4881	N/A	N/A
10560919	Atp1a3	N/A	N/A
10560945	Grik5	N/A	N/A
10560964	Pou2f2	N/A	N/A
10560993	9130221H12Rik /// Gsk3a	N/A	N/A
10561004	Erf	N/A	N/A
10561008	Ceacam1	N/A	N/A
10561017	Pafah1b3	N/A	N/A
10561031	Lipe	N/A	N/A
10561063	Bckdha	N/A	N/A
10561073	Tmem91	N/A	N/A
10561078	Ccdc97	N/A	N/A
10561085	Hnrnpul1	N/A	N/A
10561128	Cyp2s1	N/A	N/A
10561138	Rpl37a	N/A	N/A
10561140	Mrpl41	N/A	N/A
10561166	---	N/A	N/A
10561178	Rab4b	N/A	N/A
10561194	Snrpa	N/A	N/A
10561204	Itpkc	N/A	N/A
10561302	Pgam1	N/A	N/A
10561306	Pld3	N/A	N/A
10561323	Map3k10	N/A	N/A
10561335	Prkcz	N/A	N/A
10561337	1700049G17Rik	N/A	N/A
10561343	Josd1	N/A	N/A
10561356	Psmc4	N/A	N/A
10561388	Timm50	N/A	N/A
10561401	Supt5h	N/A	N/A
10561456	Med29	N/A	N/A
10561461	Samd4b	N/A	N/A
10561474	Il28a	N/A	N/A
10561485	Pak4	N/A	N/A
10561513	Mrps12	N/A	N/A
10561516	Nfkbib	N/A	N/A
10561525	Gm10648	N/A	N/A
10561527	Actn4 /// Capn12	N/A	N/A
10561552	Eif3k	N/A	N/A
10561664	Fam98c	N/A	N/A
10561673	Spred3	N/A	N/A
10561679	Psmd8	N/A	N/A
10561702	Kcnk6	N/A	N/A

10561706	Yif1b /// 2200002D01Rik	N/A	N/A
10561712	Spint2	N/A	N/A
10561721	Sipa1l3	N/A	N/A
10561777	BC027344	N/A	N/A
10561787	Zfp27	N/A	N/A
10561799	Zfp74	N/A	N/A
10561810	Zfp14	N/A	N/A
10561831	Zfp566 /// LOC100504880 /// Zfp82	N/A	N/A
10561842	Capns1	N/A	N/A
10561854	Tbcb	N/A	N/A
10561907	Sdhaf1 /// E130208F15Rik	N/A	N/A
10561918	---	N/A	N/A
10561927	Aplp1	N/A	N/A
10561956	Rps12 /// Gm10063 /// Rps12-ps2	N/A	N/A
10561958	Arhgap33	N/A	N/A
10561983	BC053749	N/A	N/A
10562000	Psenen	N/A	N/A
10562044	Zbtb32	N/A	N/A
10562059	Cox6b1	N/A	N/A
10562093	4930479M11Rik	N/A	N/A
10562096	Tmem147	N/A	N/A
10562152	Mag	N/A	N/A
10562166	Hamp2	N/A	N/A
10562169	Hamp	N/A	N/A
10562172	Usf2	N/A	N/A
10562181	Lsr	N/A	N/A
10562192	Fxyd5	N/A	N/A
10562204	Fxyd7	N/A	N/A
10562211	Fxyd1	N/A	N/A
10562234	Hpn	N/A	N/A
10562260	Gramd1a	N/A	N/A
10562285	---	N/A	N/A
10562287	Gm5329	N/A	N/A
10562289	Gm5329	N/A	N/A
10562323	Wtip	N/A	N/A
10562331	Uba2	N/A	N/A
10562358	---	N/A	N/A
10562360	Gpi1	N/A	N/A
10562368	4931406P16Rik	N/A	N/A
10562382	Lsm14a	N/A	N/A
10562397	---	N/A	N/A
10562408	Chst8	N/A	N/A
10562416	Cebpg	N/A	N/A
10562439	Gpatch1	N/A	N/A
10562461	C230052I12Rik /// Rhpn2	N/A	N/A
10562480	Nudt19	N/A	N/A
10562491	Pdcd5	N/A	N/A
10562500	Dpy19l3	N/A	N/A
10562523	Zfp507 /// E130304I02Rik	N/A	N/A
10562546	---	N/A	N/A
10562576	Plekhf1	N/A	N/A
10562578	Pop4	N/A	N/A
10562599	Gm5590	N/A	N/A
10562601	---	N/A	N/A
10562605	AI987944	N/A	N/A
10562627	Dullard	N/A	N/A
10562632	Vmn2r-ps57	N/A	N/A
10562634	---	N/A	N/A
10562637	Ccnb1	N/A	N/A
10562639	Gapdh	N/A	N/A
10562641	Vmn2r-ps57 /// Gprc2a-rs5 /// Vmn2r58 /// Vmn2r60 /// Vmn2r61 /// Vmn2r-ps60	N/A	N/A
10562643	4933421I07Rik	N/A	N/A
10562647	Rpl29	N/A	N/A
10562649	---	N/A	N/A
10562651	Gm5595 /// EU599041 /// C330019L16Rik	N/A	N/A
10562657	Gm5595	N/A	N/A
10562663	Rps4x	N/A	N/A
10562665	---	N/A	N/A
10562667	---	N/A	N/A
10562685	---	N/A	N/A
10562709	Cd33	N/A	N/A
10562729	---	N/A	N/A
10562754	---	N/A	N/A
10562761	Clec11a	N/A	N/A
10562774	2310044H10Rik	N/A	N/A
10562847	Nr1h2	N/A	N/A
10562905	Atf5 /// Nup62-il4i1	N/A	N/A
10562911	Tbc1d17	N/A	N/A
10562942	Med25	N/A	N/A
10562963	Fuz	N/A	N/A
10562965	Ap2a1	N/A	N/A
10562989	Cpt1c	N/A	N/A
10563014	Prmt1	N/A	N/A
10563050	Prr12	N/A	N/A

10563085	Fcgrt	N/A	N/A
10563094	Rps11	N/A	N/A
10563099	Snord35b	N/A	N/A
10563101	Rpl13a	N/A	N/A
10563110	Rpl13a /// Snord34	N/A	N/A
10563112	Snord33	N/A	N/A
10563114	Rpl13a /// Snord32a	N/A	N/A
10563130	Aldh16a1	N/A	N/A
10563176	---	N/A	N/A
10563253	Lin7b /// Mtag2	N/A	N/A
10563260	Snrnp70	N/A	N/A
10563275	Ruvbl2 /// Gys1	N/A	N/A
10563295	Ftl2 /// Ftl1 /// Mir692-1	N/A	N/A
10563314	Dhdh	N/A	N/A
10563323	Nucb1	N/A	N/A
10563362	Sphk2	N/A	N/A
10563373	Spaca4	N/A	N/A
10563387	Gm5897	N/A	N/A
10563411	Grwd1	N/A	N/A
10563421	Grin2d	N/A	N/A
10563434	---	N/A	N/A
10563436	Syngr4	N/A	N/A
10563441	Emp3	N/A	N/A
10563558	Sergef /// Prf1	N/A	N/A
10563583	Saal1	N/A	N/A
10563597	Saa3	N/A	N/A
10563602	Saa4	N/A	N/A
10563608	---	N/A	N/A
10563611	Saa2 /// Saa1	N/A	N/A
10563615	Hps5	N/A	N/A
10563641	Gm9392 /// Rap1a	N/A	N/A
10563643	Tsg101	N/A	N/A
10563657	---	N/A	N/A
10563659	Spty2d1	N/A	N/A
10563685	Ptpn5	N/A	N/A
10563712	Mrgpra2a /// Mrgpra2b	N/A	N/A
10563722	Mrgpra3 /// Mrgpra2a	N/A	N/A
10563743	---	N/A	N/A
10563745	Mrgprb5	N/A	N/A
10563764	Mrgprb3	N/A	N/A
10563766	---	N/A	N/A
10563768	---	N/A	N/A
10563770	Csrp3	N/A	N/A
10563802	---	N/A	N/A
10563804	---	N/A	N/A
10563806	---	N/A	N/A
10563808	---	N/A	N/A
10563810	---	N/A	N/A
10563812	---	N/A	N/A
10563814	---	N/A	N/A
10563816	Fancf /// Gas2	N/A	N/A
10563820	Svip	N/A	N/A
10563825	---	N/A	N/A
10563829	Mrps33	N/A	N/A
10563834	---	N/A	N/A
10563838	Nipa2 /// Cyfip1	N/A	N/A
10563852	Nipa1 /// A230056P14Rik	N/A	N/A
10563858	Gabrg3	N/A	N/A
10563872	Gabra5	N/A	N/A
10563891	Nck2	N/A	N/A
10563893	---	N/A	N/A
10563895	---	N/A	N/A
10563897	---	N/A	N/A
10563899	---	N/A	N/A
10563901	---	N/A	N/A
10563903	---	N/A	N/A
10563905	---	N/A	N/A
10563907	---	N/A	N/A
10563909	---	N/A	N/A
10563911	---	N/A	N/A
10563913	---	N/A	N/A
10563915	---	N/A	N/A
10563917	---	N/A	N/A
10563919	---	N/A	N/A
10563921	---	N/A	N/A
10563923	---	N/A	N/A
10563925	---	N/A	N/A
10563927	---	N/A	N/A
10563929	---	N/A	N/A
10563931	---	N/A	N/A
10563933	---	N/A	N/A
10563935	---	N/A	N/A
10563937	Snord115	N/A	N/A
10563939	---	N/A	N/A

10563941	---	N/A	N/A
10563943	---	N/A	N/A
10563945	---	N/A	N/A
10563947	Snord115	N/A	N/A
10563949	---	N/A	N/A
10563951	Snord115	N/A	N/A
10563953	Snord115	N/A	N/A
10563955	---	N/A	N/A
10563957	Snord115	N/A	N/A
10563959	---	N/A	N/A
10563961	---	N/A	N/A
10563963	---	N/A	N/A
10563965	---	N/A	N/A
10563967	---	N/A	N/A
10563969	---	N/A	N/A
10563971	---	N/A	N/A
10563973	---	N/A	N/A
10563975	---	N/A	N/A
10563977	---	N/A	N/A
10563979	---	N/A	N/A
10563981	---	N/A	N/A
10563983	---	N/A	N/A
10563985	---	N/A	N/A
10563987	---	N/A	N/A
10563989	---	N/A	N/A
10563991	---	N/A	N/A
10563993	---	N/A	N/A
10563995	Snord115	N/A	N/A
10563997	Snord115	N/A	N/A
10563999	Snord115	N/A	N/A
10564001	Snord115	N/A	N/A
10564003	Snord115	N/A	N/A
10564005	---	N/A	N/A
10564007	Snord115	N/A	N/A
10564009	---	N/A	N/A
10564011	Snord115	N/A	N/A
10564013	Snord115	N/A	N/A
10564015	---	N/A	N/A
10564017	Snord115	N/A	N/A
10564019	---	N/A	N/A
10564021	---	N/A	N/A
10564023	---	N/A	N/A
10564025	---	N/A	N/A
10564027	---	N/A	N/A
10564029	Snord115	N/A	N/A
10564031	Snord115	N/A	N/A
10564033	---	N/A	N/A
10564035	---	N/A	N/A
10564037	---	N/A	N/A
10564039	---	N/A	N/A
10564041	---	N/A	N/A
10564043	---	N/A	N/A
10564045	---	N/A	N/A
10564047	---	N/A	N/A
10564049	---	N/A	N/A
10564051	---	N/A	N/A
10564053	---	N/A	N/A
10564055	---	N/A	N/A
10564057	---	N/A	N/A
10564059	---	N/A	N/A
10564061	---	N/A	N/A
10564063	---	N/A	N/A
10564065	---	N/A	N/A
10564067	---	N/A	N/A
10564069	---	N/A	N/A
10564071	---	N/A	N/A
10564073	---	N/A	N/A
10564075	---	N/A	N/A
10564077	---	N/A	N/A
10564079	---	N/A	N/A
10564081	---	N/A	N/A
10564083	---	N/A	N/A
10564085	---	N/A	N/A
10564087	---	N/A	N/A
10564089	---	N/A	N/A
10564091	---	N/A	N/A
10564093	---	N/A	N/A
10564095	---	N/A	N/A
10564097	---	N/A	N/A
10564099	---	N/A	N/A
10564101	---	N/A	N/A
10564103	---	N/A	N/A
10564105	---	N/A	N/A
10564107	---	N/A	N/A

10564109	---	N/A	N/A
10564111	---	N/A	N/A
10564113	---	N/A	N/A
10564115	---	N/A	N/A
10564117	---	N/A	N/A
10564119	---	N/A	N/A
10564121	---	N/A	N/A
10564123	---	N/A	N/A
10564125	---	N/A	N/A
10564127	---	N/A	N/A
10564129	---	N/A	N/A
10564131	---	N/A	N/A
10564133	---	N/A	N/A
10564135	---	N/A	N/A
10564137	---	N/A	N/A
10564139	---	N/A	N/A
10564141	---	N/A	N/A
10564143	---	N/A	N/A
10564145	---	N/A	N/A
10564147	---	N/A	N/A
10564149	---	N/A	N/A
10564151	---	N/A	N/A
10564153	---	N/A	N/A
10564155	---	N/A	N/A
10564157	---	N/A	N/A
10564159	---	N/A	N/A
10564161	Snord116 /// Snord116l1 /// Snord116l2	N/A	N/A
10564163	Snord116 /// Snord116l1 /// Snord116l2	N/A	N/A
10564165	---	N/A	N/A
10564167	Snord116 /// Snord116l1 /// Snord116l2	N/A	N/A
10564169	---	N/A	N/A
10564171	Snord116 /// Snord116l1 /// Snord116l2	N/A	N/A
10564173	Snord116 /// Snord116l1 /// Snord116l2	N/A	N/A
10564175	Snord116 /// Snord116l1 /// Snord116l2	N/A	N/A
10564177	Snord116	N/A	N/A
10564179	Snord116 /// Snord116l1 /// Snord116l2	N/A	N/A
10564181	Snord116 /// Snord116l1 /// Snord116l2	N/A	N/A
10564183	Snord116	N/A	N/A
10564185	Snord116 /// Snord116l1 /// Snord116l2	N/A	N/A
10564187	Snord116 /// Snord116l1 /// Snord116l2	N/A	N/A
10564189	Snord116 /// Snord116l1 /// Snord116l2	N/A	N/A
10564191	Snord116 /// Snord116l1 /// Snord116l2	N/A	N/A
10564193	Snord116 /// Snord116l1 /// Snord116l2	N/A	N/A
10564195	Snord116 /// Snord116l1 /// Snord116l2	N/A	N/A
10564197	Snord116 /// Snord116l1 /// Snord116l2	N/A	N/A
10564199	Snord116 /// Snord116l1 /// Snord116l2	N/A	N/A
10564201	Snord116	N/A	N/A
10564203	---	N/A	N/A
10564205	Snord116	N/A	N/A
10564207	Snord116	N/A	N/A
10564209	---	N/A	N/A
10564211	Snurf /// Snrpn	N/A	N/A
10564220	Gkap1	N/A	N/A
10564231	---	N/A	N/A
10564233	Mir344 /// Mir344-2	N/A	N/A
10564235	Mir344 /// Mir344-2	N/A	N/A
10564237	Gm9801	N/A	N/A
10564260	---	N/A	N/A
10564272	Chrna7	N/A	N/A
10564313	Mphosph10	N/A	N/A
10564326	Fam189a1	N/A	N/A
10564343	Tjp1	N/A	N/A
10564373	Gm10624	N/A	N/A
10564375	---	N/A	N/A
10564434	---	N/A	N/A
10564436	Asb7	N/A	N/A
10564448	Asb7	N/A	N/A
10564451	Mef2a	N/A	N/A
10564467	Lrrc28	N/A	N/A
10564482	Synm	N/A	N/A
10564500	---	N/A	N/A
10564520	Snora21	N/A	N/A
10564525	---	N/A	N/A
10564527	Nr2f2	N/A	N/A
10564535	---	N/A	N/A
10564537	Gm10295	N/A	N/A
10564539	Mctp2	N/A	N/A
10564563	Rpl17 /// Gm10268 /// Rpl17-ps3	N/A	N/A
10564565	---	N/A	N/A
10564567	---	N/A	N/A
10564570	---	N/A	N/A
10564573	Chd2	N/A	N/A
10564616	Gm4971	N/A	N/A
10564618	Fam174b	N/A	N/A

10564622	---	N/A	N/A
10564624	St8sia2	N/A	N/A
10564631	Slco3a1	N/A	N/A
10564646	Sv2b	N/A	N/A
10564663	Gm9855 /// Tdg /// Gm5806	N/A	N/A
10564667	Ntrk3	N/A	N/A
10564692	Mrpl46	N/A	N/A
10564736	Polg	N/A	N/A
10564791	Kif7 /// 9330171B17Rik	N/A	N/A
10564805	Pex11a	N/A	N/A
10564809	Eif1 /// Gm16378 /// Gm6900	N/A	N/A
10564818	Anpep	N/A	N/A
10564839	Ap3s2	N/A	N/A
10564849	2610034B18Rik	N/A	N/A
10564857	Idh2	N/A	N/A
10564869	Cib1	N/A	N/A
10564888	Unc45a	N/A	N/A
10564909	Man2a2	N/A	N/A
10564960	Furin	N/A	N/A
10565057	Wdr73	N/A	N/A
10565067	Nmb	N/A	N/A
10565081	Timm17a	N/A	N/A
10565083	Rps17 /// Gm6402	N/A	N/A
10565089	Cpeb1	N/A	N/A
10565102	Ap3b2	N/A	N/A
10565170	3110040N11Rik	N/A	N/A
10565178	Btbd1	N/A	N/A
10565193	Hdgfrp3 /// Tm6sf1	N/A	N/A
10565210	Fam154b	N/A	N/A
10565216	Gm10610	N/A	N/A
10565250	Mesdc1	N/A	N/A
10565288	Fam108c	N/A	N/A
10565292	Arnt2	N/A	N/A
10565315	Fah	N/A	N/A
10565330	Zfand6	N/A	N/A
10565341	Gm6155	N/A	N/A
10565343	Vmn2r65	N/A	N/A
10565353	Vmn2r68-ps	N/A	N/A
10565360	---	N/A	N/A
10565379	Olfr310	N/A	N/A
10565381	---	N/A	N/A
10565383	Olfr309	N/A	N/A
10565389	Olfr307	N/A	N/A
10565391	Olfr305	N/A	N/A
10565395	Olfr303	N/A	N/A
10565399	Olfr294	N/A	N/A
10565401	Folh1	N/A	N/A
10565434	Rps13	N/A	N/A
10565437	Tmem135	N/A	N/A
10565461	Me3	N/A	N/A
10565479	l7Rn6	N/A	N/A
10565486	Eed	N/A	N/A
10565499	Gm5341	N/A	N/A
10565514	Tmem126a	N/A	N/A
10565519	Tmem126b	N/A	N/A
10565525	---	N/A	N/A
10565528	---	N/A	N/A
10565532	Ankrd42	N/A	N/A
10565547	Pcf11	N/A	N/A
10565565	---	N/A	N/A
10565567	4632427E13Rik	N/A	N/A
10565582	Rps8 /// Gm5121 /// Gm11353 /// Rps8-ps1	N/A	N/A
10565587	Gm5037 /// Fxn	N/A	N/A
10565591	---	N/A	N/A
10565596	---	N/A	N/A
10565598	LOC280487	N/A	N/A
10565607	---	N/A	N/A
10565609	Thrsp	N/A	N/A
10565612	1810020D17Rik	N/A	N/A
10565689	Capn5	N/A	N/A
10565727	Tsku	N/A	N/A
10565735	A630091E08Rik	N/A	N/A
10565738	2210018M11Rik	N/A	N/A
10565759	Uvrag	N/A	N/A
10565775	Dgat2	N/A	N/A
10565802	Rps3	N/A	N/A
10565811	Snord15b	N/A	N/A
10565813	Snord15a	N/A	N/A
10565815	Gm4980	N/A	N/A
10565817	---	N/A	N/A
10565819	Slco2b1	N/A	N/A
10565846	Spcs2	N/A	N/A
10565852	Rnf169	N/A	N/A
10565858	Gm5115	N/A	N/A

10565862	Pold3	N/A	N/A
10565873	Ppme1	N/A	N/A
10565888	Gm10603	N/A	N/A
10565900	D630004N19Rik /// Mrpl48	N/A	N/A
10565904	Mrpl48	N/A	N/A
10565921	Gapdh /// Gm3200	N/A	N/A
10565935	Arhgef17	N/A	N/A
10565958	P2ry6	N/A	N/A
10565962	P2ry2	N/A	N/A
10565994	Art2b	N/A	N/A
10565996	Inppl1	N/A	N/A
10566050	Il18bp	N/A	N/A
10566067	Rnf121	N/A	N/A
10566097	Nup98	N/A	N/A
10566132	Rhog	N/A	N/A
10566136	Olfr543	N/A	N/A
10566140	Olfr545	N/A	N/A
10566155	---	N/A	N/A
10566159	Olfr553	N/A	N/A
10566182	Olfr566	N/A	N/A
10566184	Olfr569	N/A	N/A
10566186	Olfr571	N/A	N/A
10566188	---	N/A	N/A
10566190	Olfr575	N/A	N/A
10566194	Olfr578	N/A	N/A
10566199	Olfr586	N/A	N/A
10566201	Olfr589	N/A	N/A
10566205	Dub2a /// Usp-ps	N/A	N/A
10566207	Olfr600	N/A	N/A
10566209	Olfr601	N/A	N/A
10566211	Olfr603	N/A	N/A
10566215	Olfr607	N/A	N/A
10566217	Olfr609	N/A	N/A
10566219	Olfr610	N/A	N/A
10566223	Olfr611 /// Olfr612	N/A	N/A
10566225	Olfr616	N/A	N/A
10566227	Olfr620	N/A	N/A
10566229	Olfr620 /// Olfr624	N/A	N/A
10566231	Olfr622	N/A	N/A
10566239	Olfr629	N/A	N/A
10566249	Olfr68	N/A	N/A
10566281	Dnajc19	N/A	N/A
10566292	Olfr639	N/A	N/A
10566294	Olfr640	N/A	N/A
10566296	Olfr642	N/A	N/A
10566300	Olfr644	N/A	N/A
10566302	Olfr645	N/A	N/A
10566322	Olfr648	N/A	N/A
10566324	Olfr649	N/A	N/A
10566333	Trim12a /// Trim5 /// 9230105E10Rik	N/A	N/A
10566346	9230105E10Rik	N/A	N/A
10566366	Trim30d	N/A	N/A
10566395	Dub1a	N/A	N/A
10566398	Olfr666	N/A	N/A
10566403	Olfr668	N/A	N/A
10566405	Mapksp1	N/A	N/A
10566409	Olfr670	N/A	N/A
10566411	Olfr671	N/A	N/A
10566413	Olfr672	N/A	N/A
10566417	Olfr675	N/A	N/A
10566419	---	N/A	N/A
10566422	---	N/A	N/A
10566425	Olfr683	N/A	N/A
10566430	Olfr685	N/A	N/A
10566432	Olfr686	N/A	N/A
10566434	Olfr690	N/A	N/A
10566436	Olfr691	N/A	N/A
10566438	Fam160a2	N/A	N/A
10566452	Gm4972	N/A	N/A
10566454	Prkcdbp	N/A	N/A
10566457	Apbb1	N/A	N/A
10566477	Hpx	N/A	N/A
10566488	Trim3	N/A	N/A
10566502	Arfip2	N/A	N/A
10566512	---	N/A	N/A
10566514	Mrps36	N/A	N/A
10566516	Rrp8	N/A	N/A
10566525	Taf10 /// Ilk	N/A	N/A
10566529	Tpp1	N/A	N/A
10566543	Dchs1	N/A	N/A
10566564	Mrpl17	N/A	N/A
10566574	Gvin1	N/A	N/A
10566578	Gvin1 /// Gm8979	N/A	N/A
10566580	Gm4759	N/A	N/A

10566583	Gm8995	N/A	N/A
10566585	Gm1966	N/A	N/A
10566587	Olfr693	N/A	N/A
10566589	Olfr694 /// Olfr704	N/A	N/A
10566593	---	N/A	N/A
10566595	Olfr697	N/A	N/A
10566599	Olfr699	N/A	N/A
10566601	Olfr700	N/A	N/A
10566607	Olfr705	N/A	N/A
10566609	Olfr706	N/A	N/A
10566611	Olfr707	N/A	N/A
10566615	Olfr710	N/A	N/A
10566618	Olfr6	N/A	N/A
10566620	Olfr711	N/A	N/A
10566622	Olfr2	N/A	N/A
10566626	Olfr715	N/A	N/A
10566630	5330417H12Rik	N/A	N/A
10566662	Olfr469	N/A	N/A
10566664	Olfr470	N/A	N/A
10566666	Olfr471	N/A	N/A
10566668	Socs6	N/A	N/A
10566676	Olfr484	N/A	N/A
10566678	Olfr485	N/A	N/A
10566682	Olfr486 /// Olfr487	N/A	N/A
10566684	Olfr488	N/A	N/A
10566688	Olfr492	N/A	N/A
10566690	Olfr493	N/A	N/A
10566692	Olfr502	N/A	N/A
10566694	Olfr504	N/A	N/A
10566696	Olfr509	N/A	N/A
10566698	Olfr514	N/A	N/A
10566705	Olfr518	N/A	N/A
10566707	Olfr518 /// Olfr516 /// Olfr519	N/A	N/A
10566714	Ric3	N/A	N/A
10566743	Trim66	N/A	N/A
10566804	Tmem9b	N/A	N/A
10566846	Dennd5a	N/A	N/A
10566870	Tmem41b	N/A	N/A
10566875	AA474408	N/A	N/A
10566877	Sbf2	N/A	N/A
10566922	B430319F04Rik	N/A	N/A
10566926	Rnf141	N/A	N/A
10566934	Lyve1	N/A	N/A
10566966	Eif4g2	N/A	N/A
10567010	Dkk3	N/A	N/A
10567020	Rpl7a /// Rpl7a-ps3 /// Rpl7a-ps10	N/A	N/A
10567041	Gm5600	N/A	N/A
10567043	Rras2	N/A	N/A
10567049	Copb1	N/A	N/A
10567072	Psma1	N/A	N/A
10567084	---	N/A	N/A
10567086	Cyp2r1	N/A	N/A
10567095	Calca	N/A	N/A
10567106	Rps4y2 /// Gm6816	N/A	N/A
10567108	Sox6	N/A	N/A
10567131	Nup35	N/A	N/A
10567134	Plekha7	N/A	N/A
10567163	Rps13	N/A	N/A
10567171	Rps13 /// Snord14a	N/A	N/A
10567173	Pik3c2a	N/A	N/A
10567213	---	N/A	N/A
10567216	Rps15a	N/A	N/A
10567219	Arl6ip1	N/A	N/A
10567229	Smg1	N/A	N/A
10567289	Syt17	N/A	N/A
10567297	Itpripl2	N/A	N/A
10567299	Itpripl2	N/A	N/A
10567316	Tmc7	N/A	N/A
10567355	Gprc5b	N/A	N/A
10567361	Gpr139	N/A	N/A
10567394	Pdilt	N/A	N/A
10567407	Thumpd1	N/A	N/A
10567412	Eri2 /// Acsm3	N/A	N/A
10567423	Dcun1d3	N/A	N/A
10567428	---	N/A	N/A
10567430	---	N/A	N/A
10567432	Dnahc3	N/A	N/A
10567442	---	N/A	N/A
10567444	Dnahc3	N/A	N/A
10567446	Dnahc3	N/A	N/A
10567448	Dnahc3	N/A	N/A
10567450	Dnahc3	N/A	N/A
10567452	---	N/A	N/A
10567454	---	N/A	N/A

10567456	---	N/A	N/A
10567458	---	N/A	N/A
10567460	---	N/A	N/A
10567462	---	N/A	N/A
10567464	---	N/A	N/A
10567466	---	N/A	N/A
10567490	---	N/A	N/A
10567492	---	N/A	N/A
10567494	---	N/A	N/A
10567496	---	N/A	N/A
10567498	---	N/A	N/A
10567500	---	N/A	N/A
10567502	---	N/A	N/A
10567504	---	N/A	N/A
10567506	---	N/A	N/A
10567508	---	N/A	N/A
10567510	---	N/A	N/A
10567512	---	N/A	N/A
10567514	---	N/A	N/A
10567516	---	N/A	N/A
10567518	---	N/A	N/A
10567520	---	N/A	N/A
10567522	---	N/A	N/A
10567564	Cdr2	N/A	N/A
10567574	Ppia	N/A	N/A
10567576	Mettl9 /// Gm9905	N/A	N/A
10567578	---	N/A	N/A
10567587	---	N/A	N/A
10567589	Usp31	N/A	N/A
10567591	Usp31	N/A	N/A
10567626	Gga2	N/A	N/A
10567657	Ndufab1	N/A	N/A
10567702	Arhgap17	N/A	N/A
10567725	Zkscan2	N/A	N/A
10567735	---	N/A	N/A
10567737	---	N/A	N/A
10567750	Gtf3c1	N/A	N/A
10567788	Gsg1l	N/A	N/A
10567797	Xpo6	N/A	N/A
10567823	Rpl21	N/A	N/A
10567903	Sh2b1	N/A	N/A
10567919	Atxn2l	N/A	N/A
10567995	Nupr1	N/A	N/A
10567999	Ccdc101	N/A	N/A
10568001	Sult1a1	N/A	N/A
10568011	Giyd2	N/A	N/A
10568038	Gm9967	N/A	N/A
10568050	Aldoa	N/A	N/A
10568078	Taok2	N/A	N/A
10568099	Tmem219	N/A	N/A
10568107	---	N/A	N/A
10568115	Mvp /// 2900092E17Rik	N/A	N/A
10568131	2900092E17Rik	N/A	N/A
10568135	2900092E17Rik /// Prrt2	N/A	N/A
10568139	Maz	N/A	N/A
10568169	Qprt	N/A	N/A
10568174	Spn	N/A	N/A
10568188	Tbc1d10b	N/A	N/A
10568215	Gm4532 /// Zfp553	N/A	N/A
10568217	Dctpp1	N/A	N/A
10568221	Sephs2	N/A	N/A
10568239	E430018J23Rik	N/A	N/A
10568243	Zfp764	N/A	N/A
10568252	Zfp689	N/A	N/A
10568258	Srcap /// 1700008J07Rik	N/A	N/A
10568260	Zfp629	N/A	N/A
10568278	1700120K04Rik	N/A	N/A
10568294	---	N/A	N/A
10568296	Hsd3b7	N/A	N/A
10568298	Stx1b	N/A	N/A
10568318	Prss53 /// Vkorc1	N/A	N/A
10568328	Vkorc1	N/A	N/A
10568332	Prss8	N/A	N/A
10568355	Pycard	N/A	N/A
10568361	Yipf5	N/A	N/A
10568363	Armcx3	N/A	N/A
10568367	---	N/A	N/A
10568369	Cox6a2	N/A	N/A
10568374	9130023H24Rik /// Armc5	N/A	N/A
10568376	Slc5a2	N/A	N/A
10568390	---	N/A	N/A
10568392	Rgs10	N/A	N/A
10568399	Tial1	N/A	N/A
10568417	1110007A13Rik	N/A	N/A

10568432	---	N/A	N/A
10568434	---	N/A	N/A
10568436	Fgfr2	N/A	N/A
10568461	Rfwd3	N/A	N/A
10568464	Ate1	N/A	N/A
10568480	Nsmce4a	N/A	N/A
10568521	2310057M21Rik	N/A	N/A
10568529	Ikzf5	N/A	N/A
10568532	Rpl7a	N/A	N/A
10568534	LOC280487	N/A	N/A
10568553	Chst15	N/A	N/A
10568568	Oat	N/A	N/A
10568586	Fam53b	N/A	N/A
10568605	Ctbp2	N/A	N/A
10568622	---	N/A	N/A
10568638	Uros	N/A	N/A
10568651	Dhx32	N/A	N/A
10568705	Fam196a	N/A	N/A
10568731	---	N/A	N/A
10568733	Gm10579	N/A	N/A
10568758	9430038I01Rik	N/A	N/A
10568765	Tcerg1l	N/A	N/A
10568780	Mapk1ip1	N/A	N/A
10568792	Stk32c	N/A	N/A
10568805	Inpp5a /// Nkx6-2	N/A	N/A
10568810	E030019B06Rik	N/A	N/A
10568859	Ndufab1	N/A	N/A
10568861	---	N/A	N/A
10568897	Tubgcp2	N/A	N/A
10568921	Caly	N/A	N/A
10568930	1810014F10Rik	N/A	N/A
10568939	Echs1	N/A	N/A
10568948	Sprn /// Mtg1	N/A	N/A
10568954	Olfr524	N/A	N/A
10568958	Olfr530	N/A	N/A
10568962	Olfr532	N/A	N/A
10568982	BC066028	N/A	N/A
10568988	Bet1l	N/A	N/A
10569008	Cox8b	N/A	N/A
10569011	Ifitm5	N/A	N/A
10569014	Ifitm2	N/A	N/A
10569017	Ifitm3	N/A	N/A
10569057	Rnh1	N/A	N/A
10569069	Rps9	N/A	N/A
10569100	Mir210	N/A	N/A
10569102	Irf7	N/A	N/A
10569113	Cdhr5	N/A	N/A
10569129	Sct	N/A	N/A
10569134	Deaf1	N/A	N/A
10569149	Eps8l2 /// B230206H07Rik	N/A	N/A
10569152	Pddc1	N/A	N/A
10569163	Cend1	N/A	N/A
10569168	Slc25a22	N/A	N/A
10569198	Cd151	N/A	N/A
10569200	Polr2l /// Usp50	N/A	N/A
10569265	Tollip	N/A	N/A
10569273	2700078K21Rik	N/A	N/A
10569278	Dusp8 /// 5530400B01Rik	N/A	N/A
10569280	Dusp8	N/A	N/A
10569288	Krtap5-4 /// Krtap5-2	N/A	N/A
10569291	Krtap5-2	N/A	N/A
10569296	Krtap5-3 /// Krtap5-2 /// Krtap5-1	N/A	N/A
10569300	Krtap5-5	N/A	N/A
10569303	---	N/A	N/A
10569306	Krtap5-4 /// Krtap5-1 /// Gm4559	N/A	N/A
10569308	Krtap5-1	N/A	N/A
10569311	---	N/A	N/A
10569313	6330512M04Rik	N/A	N/A
10569319	Ctsd	N/A	N/A
10569341	Mir675 /// H19	N/A	N/A
10569356	Mir483	N/A	N/A
10569368	---	N/A	N/A
10569370	Th	N/A	N/A
10569389	---	N/A	N/A
10569393	R74862	N/A	N/A
10569429	Cdkn1c	N/A	N/A
10569437	Phlda2	N/A	N/A
10569441	Nap1l4	N/A	N/A
10569456	---	N/A	N/A
10569494	Tnfrsf22	N/A	N/A
10569539	Mrgprg	N/A	N/A
10569569	Cttn	N/A	N/A
10569583	Ppfia1	N/A	N/A
10569611	Fadd	N/A	N/A

10569654	1810010D01Rik	N/A	N/A
10569702	Cacng8	N/A	N/A
10569714	Cacng7	N/A	N/A
10569717	---	N/A	N/A
10569733	Arhgef18	N/A	N/A
10569767	Zfp358	N/A	N/A
10569771	Mcoln1	N/A	N/A
10569786	Pnpla6	N/A	N/A
10569848	Stxbp2	N/A	N/A
10569870	Retn	N/A	N/A
10569886	Trappc5	N/A	N/A
10569890	Aida	N/A	N/A
10569896	Cd209g	N/A	N/A
10569898	Evi5l	N/A	N/A
10569927	Map2k7 /// Gm14378	N/A	N/A
10569953	Snapc2	N/A	N/A
10569958	Ccl25	N/A	N/A
10569962	Ccl25	N/A	N/A
10569972	Lass4	N/A	N/A
10569985	BC003267	N/A	N/A
10569992	LOC674846	N/A	N/A
10569996	Rpl21 /// Rpl21-ps14	N/A	N/A
10570000	Gpi1	N/A	N/A
10570002	2410089E03Rik	N/A	N/A
10570005	4921522P10Rik	N/A	N/A
10570008	B930078G14Rik	N/A	N/A
10570119	Carkd	N/A	N/A
10570139	---	N/A	N/A
10570141	---	N/A	N/A
10570144	Arhgef7	N/A	N/A
10570178	A230072I06Rik	N/A	N/A
10570180	Gm5607	N/A	N/A
10570189	Sox1	N/A	N/A
10570199	---	N/A	N/A
10570201	Atp11a	N/A	N/A
10570278	---	N/A	N/A
10570280	F7	N/A	N/A
10570291	F10	N/A	N/A
10570308	Proz	N/A	N/A
10570321	Cul4a	N/A	N/A
10570344	Lamp1	N/A	N/A
10570356	Tmco3	N/A	N/A
10570397	Cdc16	N/A	N/A
10570429	Zfp828	N/A	N/A
10570432	Snora3	N/A	N/A
10570434	Ifitm1	N/A	N/A
10570437	Fbxo25	N/A	N/A
10570448	Gm10699	N/A	N/A
10570450	Dlgap2	N/A	N/A
10570470	Hmgb1	N/A	N/A
10570513	Kbtbd11	N/A	N/A
10570516	Kbtbd11	N/A	N/A
10570556	Mcph1	N/A	N/A
10570573	Agpat5	N/A	N/A
10570585	Xkr5	N/A	N/A
10570587	Defb34	N/A	N/A
10570604	---	N/A	N/A
10570618	Defb46	N/A	N/A
10570621	Defb3	N/A	N/A
10570630	---	N/A	N/A
10570634	Gm15319 /// 4930467E23Rik /// Trim44 /// Gm7827	N/A	N/A
10570639	2610005L07Rik	N/A	N/A
10570641	AY761185	N/A	N/A
10570647	---	N/A	N/A
10570651	Defa21 /// Defa22	N/A	N/A
10570656	Defa23	N/A	N/A
10570660	Defa4 /// Gm14850 /// Gm15315 /// Defa5 /// Gm10104 /// Gm15293	N/A	N/A
10570663	Defa23 /// Defa25 /// Gm6696 /// Defa-ps1 /// Gm15299	N/A	N/A
10570668	Defa6 /// Defa23 /// Defa24 /// Defa-rs7 /// Defa17 /// Gm15284 /// Defa3	N/A	N/A
10570671	Defa21 /// Defa22	N/A	N/A
10570676	Defa24	N/A	N/A
10570680	---	N/A	N/A
10570687	---	N/A	N/A
10570690	Defa13	N/A	N/A
10570693	Gm14850 /// Gm15315 /// Defa5 /// Gm10104 /// Gm15293	N/A	N/A
10570700	Defa23 /// Defa25 /// Gm6696 /// Defa-ps1 /// Gm15299	N/A	N/A
10570706	Defa20 /// Defa3 /// Gm15308	N/A	N/A
10570711	Defa20 /// Defa3 /// Gm15308	N/A	N/A
10570717	Defa4 /// Gm14850 /// Gm15315 /// Defa5 /// Gm10104 /// Gm15293	N/A	N/A
10570726	Defa26 /// Defa-rs7 /// Defa23	N/A	N/A
10570729	Defa13	N/A	N/A
10570732	Defa4 /// Gm14850 /// Gm15315 /// Defa5 /// Gm10104 /// Gm15293	N/A	N/A
10570738	---	N/A	N/A
10570758	Defb13	N/A	N/A

10570764	Alg11	N/A	N/A
10570771	Vps36	N/A	N/A
10570786	Thsd1	N/A	N/A
10570855	Plat	N/A	N/A
10570875	Myst3	N/A	N/A
10570951	Nkx6-3	N/A	N/A
10570955	Golga7 /// 4930518F22Rik	N/A	N/A
10570957	Sfrp1	N/A	N/A
10570975	Tm2d2	N/A	N/A
10570980	Rps23 /// Gm8618	N/A	N/A
10570982	Fgfr1	N/A	N/A
10571005	Letm2 /// D830025C05Rik	N/A	N/A
10571007	Whsc1l1	N/A	N/A
10571036	Ppapdc1b	N/A	N/A
10571045	Bag4 /// Ddhd2	N/A	N/A
10571048	Lsm1	N/A	N/A
10571093	Rnf170	N/A	N/A
10571111	Zfp703	N/A	N/A
10571114	Erlin2	N/A	N/A
10571128	Prosc	N/A	N/A
10571162	Eif4ebp1	N/A	N/A
10571205	---	N/A	N/A
10571207	Dusp26	N/A	N/A
10571214	Rnf122	N/A	N/A
10571221	Mak16 /// BC019943	N/A	N/A
10571241	Purg	N/A	N/A
10571246	5930422O12Rik	N/A	N/A
10571248	---	N/A	N/A
10571250	Hmgb1-rs17	N/A	N/A
10571266	Ppp2cb /// Ubxn8	N/A	N/A
10571274	Gsr	N/A	N/A
10571288	Gtf2e2	N/A	N/A
10571297	---	N/A	N/A
10571300	Mboat4	N/A	N/A
10571302	Tmem66	N/A	N/A
10571310	Rpl29	N/A	N/A
10571319	Tnks	N/A	N/A
10571321	Ppp1r3b	N/A	N/A
10571325	Mfhas1	N/A	N/A
10571342	Rps12 /// Gm10063	N/A	N/A
10571344	D8Ertd82e	N/A	N/A
10571362	AI429214	N/A	N/A
10571364	---	N/A	N/A
10571371	Tusc3	N/A	N/A
10571382	---	N/A	N/A
10571399	Zdhhc2	N/A	N/A
10571439	4930529F22Rik	N/A	N/A
10571444	Slc7a2	N/A	N/A
10571474	Pcm1	N/A	N/A
10571514	---	N/A	N/A
10571528	---	N/A	N/A
10571530	Fat1	N/A	N/A
10571567	Sorbs2	N/A	N/A
10571599	---	N/A	N/A
10571621	Ufsp2	N/A	N/A
10571634	Lrp2bp /// Ankrd37	N/A	N/A
10571647	4933411K20Rik	N/A	N/A
10571653	Actg1	N/A	N/A
10571657	Acsl1	N/A	N/A
10571705	Irf2	N/A	N/A
10571724	Gm10083	N/A	N/A
10571728	Rwdd4a	N/A	N/A
10571737	AA386476	N/A	N/A
10571739	Cdkn2aip	N/A	N/A
10571741	---	N/A	N/A
10571745	Cldn22 /// Wwc2	N/A	N/A
10571747	---	N/A	N/A
10571761	---	N/A	N/A
10571763	---	N/A	N/A
10571768	---	N/A	N/A
10571774	Aga	N/A	N/A
10571788	Vegfc	N/A	N/A
10571815	Gpm6a	N/A	N/A
10571824	Gm10674 /// Gm7360	N/A	N/A
10571829	Glra3	N/A	N/A
10571840	Hpgd	N/A	N/A
10571849	Fbxo8	N/A	N/A
10571857	---	N/A	N/A
10571860	Hand2	N/A	N/A
10571865	Scrg1	N/A	N/A
10571876	Gapdh	N/A	N/A
10571878	BC030500	N/A	N/A
10571889	---	N/A	N/A
10571911	2700029M09Rik	N/A	N/A

10571920	B230317F23Rik	N/A	N/A
10571922	Nek1	N/A	N/A
10571975	---	N/A	N/A
10571978	Cbr4	N/A	N/A
10572024	Spock3	N/A	N/A
10572038	Tmem192	N/A	N/A
10572046	---	N/A	N/A
10572048	---	N/A	N/A
10572050	March1	N/A	N/A
10572064	---	N/A	N/A
10572070	Npy1r	N/A	N/A
10572077	Naf1	N/A	N/A
10572083	---	N/A	N/A
10572109	Ints10	N/A	N/A
10572146	Atp6v1b2	N/A	N/A
10572161	D10627	N/A	N/A
10572170	D130040H23Rik	N/A	N/A
10572212	Gmip	N/A	N/A
10572241	Pbx4	N/A	N/A
10572251	Tssk6 /// Ndufa13	N/A	N/A
10572253	Sugp1	N/A	N/A
10572271	Tm6sf2	N/A	N/A
10572282	Hapln4	N/A	N/A
10572332	Sugp2	N/A	N/A
10572357	Cope	N/A	N/A
10572368	Gdf1 /// Lass1	N/A	N/A
10572408	Fkbp8	N/A	N/A
10572419	Ell	N/A	N/A
10572449	Lsm4	N/A	N/A
10572456	Jund /// Gm11175	N/A	N/A
10572485	Rab3a	N/A	N/A
10572514	A230052G05Rik	N/A	N/A
10572516	Mtap1s	N/A	N/A
10572525	Gm10654	N/A	N/A
10572527	Gm15319 /// 4930467E23Rik /// Trim44 /// Gm7827	N/A	N/A
10572533	Myo9b	N/A	N/A
10572580	Use1	N/A	N/A
10572591	Ocel1	N/A	N/A
10572596	5430437P03Rik	N/A	N/A
10572613	Mrpl34	N/A	N/A
10572616	Dda1	N/A	N/A
10572622	Gtpbp3	N/A	N/A
10572635	Sfn	N/A	N/A
10572637	Fam125a	N/A	N/A
10572647	Slc27a1	N/A	N/A
10572663	Pgls	N/A	N/A
10572722	---	N/A	N/A
10572724	Zfp709	N/A	N/A
10572727	Zfp882	N/A	N/A
10572730	Zfp617	N/A	N/A
10572733	BC049349	N/A	N/A
10572739	6430601O08Rik	N/A	N/A
10572741	Olfr372	N/A	N/A
10572743	Olfr373	N/A	N/A
10572745	Olfr374	N/A	N/A
10572755	---	N/A	N/A
10572757	Rab8a	N/A	N/A
10572779	Fam32a	N/A	N/A
10572786	Ap1m1	N/A	N/A
10572804	1700030K09Rik	N/A	N/A
10572813	Usmg5	N/A	N/A
10572815	Tmem38a /// Nwd1	N/A	N/A
10572838	Sin3b	N/A	N/A
10572870	Hmgxb4	N/A	N/A
10572880	Tom1	N/A	N/A
10572928	Rasd2	N/A	N/A
10572932	Naa20	N/A	N/A
10572949	Nr3c2	N/A	N/A
10572958	---	N/A	N/A
10572960	0610038B21Rik /// Arhgap10	N/A	N/A
10572962	Prmt10	N/A	N/A
10573008	Zfp827	N/A	N/A
10573027	Otud4	N/A	N/A
10573048	Anapc10	N/A	N/A
10573075	---	N/A	N/A
10573077	Usp38	N/A	N/A
10573082	Inpp4b	N/A	N/A
10573110	---	N/A	N/A
10573115	Rnf150	N/A	N/A
10573128	Tbc1d9	N/A	N/A
10573194	Ndufb7	N/A	N/A
10573198	Dnajb1	N/A	N/A
10573203	Gipc1	N/A	N/A
10573232	---	N/A	N/A

10573234	Lphn1	N/A	N/A
10573261	Asf1b	N/A	N/A
10573295	Rfx1	N/A	N/A
10573317	Mir709	N/A	N/A
10573342	Mir23a	N/A	N/A
10573344	Mir27a	N/A	N/A
10573346	Mir24-2	N/A	N/A
10573348	Cacna1a	N/A	N/A
10573401	Trmt1	N/A	N/A
10573427	Nfix /// G430095P16Rik	N/A	N/A
10573430	Gadd45gip1 /// Rad23a	N/A	N/A
10573434	Farsa	N/A	N/A
10573451	Syce2	N/A	N/A
10573457	Klf1	N/A	N/A
10573483	Prdx2	N/A	N/A
10573515	2310036O22Rik	N/A	N/A
10573519	Tnpo2	N/A	N/A
10573549	Fbxw9	N/A	N/A
10573566	Dhps	N/A	N/A
10573578	BC056474	N/A	N/A
10573583	Man2b1	N/A	N/A
10573613	Olfr371	N/A	N/A
10573626	Gpt2	N/A	N/A
10573637	Phkb	N/A	N/A
10573673	---	N/A	N/A
10573675	Lonp2	N/A	N/A
10573691	Gm10638 /// Siah1a	N/A	N/A
10573693	Rps13	N/A	N/A
10573703	Tmem188	N/A	N/A
10573713	Heatr3	N/A	N/A
10573731	9430002A10Rik	N/A	N/A
10573733	Papd5	N/A	N/A
10573803	Cyld	N/A	N/A
10573821	Rps6 /// Rps6-ps2 /// Gm16409	N/A	N/A
10573823	Chd9	N/A	N/A
10573865	Gm3579	N/A	N/A
10573867	Rbl2	N/A	N/A
10573893	Fto	N/A	N/A
10573908	---	N/A	N/A
10573954	Capns2	N/A	N/A
10573975	Ces5a	N/A	N/A
10573979	Gnao1	N/A	N/A
10573996	Amfr	N/A	N/A
10573998	Ogfod1	N/A	N/A
10574018	Mt3	N/A	N/A
10574023	Mt2	N/A	N/A
10574027	Mt1	N/A	N/A
10574033	Nup93	N/A	N/A
10574057	Mir138-2	N/A	N/A
10574087	Herpud1	N/A	N/A
10574096	Ap3s1	N/A	N/A
10574098	Nlrc5	N/A	N/A
10574100	Nlrc5	N/A	N/A
10574102	Nlrc5	N/A	N/A
10574133	Nlrc5	N/A	N/A
10574135	Nlrc5	N/A	N/A
10574139	Nlrc5	N/A	N/A
10574141	Nlrc5	N/A	N/A
10574143	Nlrc5	N/A	N/A
10574145	Nlrc5	N/A	N/A
10574147	---	N/A	N/A
10574149	Nlrc5	N/A	N/A
10574151	Nlrc5	N/A	N/A
10574153	Nlrc5	N/A	N/A
10574155	Nlrc5	N/A	N/A
10574157	Nlrc5	N/A	N/A
10574159	Nlrc5	N/A	N/A
10574161	Nlrc5	N/A	N/A
10574163	Nlrc5	N/A	N/A
10574166	Cpne2	N/A	N/A
10574184	Rspry1	N/A	N/A
10574204	Arl2bp	N/A	N/A
10574220	Cx3cl1	N/A	N/A
10574226	Ccl17	N/A	N/A
10574230	---	N/A	N/A
10574232	Coq9	N/A	N/A
10574242	Polr2c /// Dok4	N/A	N/A
10574259	Gpr56	N/A	N/A
10574308	Katnb1	N/A	N/A
10574342	AA960436	N/A	N/A
10574350	Mmp15	N/A	N/A
10574375	---	N/A	N/A
10574384	Ndrg4	N/A	N/A
10574404	Setd6	N/A	N/A

10574410	Cnot1 /// 4930513N10Rik	N/A	N/A
10574412	Sap18 /// Gm10094	N/A	N/A
10574421	Gm10632	N/A	N/A
10574427	Impdh2	N/A	N/A
10574432	LOC280487	N/A	N/A
10574434	LOC280487	N/A	N/A
10574436	Khdrbs1	N/A	N/A
10574438	Cdh5	N/A	N/A
10574451	Bean1	N/A	N/A
10574456	Cklf	N/A	N/A
10574471	Cmtm3	N/A	N/A
10574478	Dync1li2	N/A	N/A
10574498	Ces2a	N/A	N/A
10574545	Ces2e	N/A	N/A
10574572	Ces2g	N/A	N/A
10574595	---	N/A	N/A
10574598	Ces3a	N/A	N/A
10574607	Ces3b	N/A	N/A
10574617	Ces4a	N/A	N/A
10574632	Cbfb	N/A	N/A
10574641	D230025D16Rik	N/A	N/A
10574682	E2f4	N/A	N/A
10574694	Elmo3	N/A	N/A
10574718	Tmem208 /// Fhod1	N/A	N/A
10574789	Fam65a /// Mir1966	N/A	N/A
10574812	Ctcf	N/A	N/A
10574897	Thap11	N/A	N/A
10574939	Pskh1	N/A	N/A
10574944	Dus2l /// Ddx28	N/A	N/A
10574962	Nfatc3	N/A	N/A
10574976	Pla2g15	N/A	N/A
10574985	Slc7a6	N/A	N/A
10574996	Prmt7	N/A	N/A
10575019	---	N/A	N/A
10575021	Zfp90	N/A	N/A
10575074	Tmco7	N/A	N/A
10575102	Cirh1a	N/A	N/A
10575120	Sntb2	N/A	N/A
10575129	Vps4a	N/A	N/A
10575142	Cog8 /// Pdf /// Gm10627	N/A	N/A
10575151	C630050I24Rik	N/A	N/A
10575153	Cyb5b	N/A	N/A
10575160	Nfat5	N/A	N/A
10575184	Wwp2	N/A	N/A
10575209	LOC100505227	N/A	N/A
10575213	Zfhx3	N/A	N/A
10575249	Txnl4b	N/A	N/A
10575291	Zfp821	N/A	N/A
10575302	Ap1g1	N/A	N/A
10575326	---	N/A	N/A
10575328	Phlpp2	N/A	N/A
10575349	Tat	N/A	N/A
10575363	Zfp612	N/A	N/A
10575376	Ftsjd1	N/A	N/A
10575473	Gm9558	N/A	N/A
10575497	Mtss1l	N/A	N/A
10575512	Cog4	N/A	N/A
10575534	St3gal2	N/A	N/A
10575548	---	N/A	N/A
10575550	Exosc6 /// Aars	N/A	N/A
10575578	Pdpr	N/A	N/A
10575596	---	N/A	N/A
10575598	Znrf1	N/A	N/A
10575616	Gabarapl2	N/A	N/A
10575619	Terf2ip	N/A	N/A
10575628	---	N/A	N/A
10575630	Cntnap4	N/A	N/A
10575662	Syce1l /// Mon1b	N/A	N/A
10575685	Nudt7	N/A	N/A
10575693	Vat1l	N/A	N/A
10575706	Wwox	N/A	N/A
10575745	Atmin	N/A	N/A
10575750	Bcmo1	N/A	N/A
10575763	Gan	N/A	N/A
10575775	4933407C03Rik	N/A	N/A
10575777	4933407C03Rik	N/A	N/A
10575833	Hsd17b2	N/A	N/A
10575840	LOC100504406	N/A	N/A
10575842	---	N/A	N/A
10575844	Cdh13	N/A	N/A
10575861	Hsbp1	N/A	N/A
10575867	Mlycd	N/A	N/A
10575873	Osgin1	N/A	N/A
10575880	Necab2	N/A	N/A

10575961	Usp10	N/A	N/A
10575993	6430548M08Rik	N/A	N/A
10576010	Gse1 /// Gins2	N/A	N/A
10576027	Gm10614	N/A	N/A
10576029	Cox4i1	N/A	N/A
10576046	Foxf1a	N/A	N/A
10576049	Foxf1a	N/A	N/A
10576051	Foxc2	N/A	N/A
10576056	Map1lc3b	N/A	N/A
10576062	Jph3	N/A	N/A
10576073	Banp	N/A	N/A
10576088	Gm22	N/A	N/A
10576090	Zfpm1	N/A	N/A
10576115	Il17c	N/A	N/A
10576152	Trappc2l	N/A	N/A
10576158	Cbfa2t3	N/A	N/A
10576160	Acsf3	N/A	N/A
10576173	Gm16378	N/A	N/A
10576189	---	N/A	N/A
10576191	Spg7	N/A	N/A
10576216	Rpl13 /// Snord68	N/A	N/A
10576218	Cpne7	N/A	N/A
10576246	Rps12 /// Gm10063 /// Rps12-ps2	N/A	N/A
10576258	Cdk10	N/A	N/A
10576288	Spire2	N/A	N/A
10576305	Tcf25	N/A	N/A
10576328	Mc1r	N/A	N/A
10576335	Def8	N/A	N/A
10576354	Afg3l1	N/A	N/A
10576373	Gas8	N/A	N/A
10576386	Rhou	N/A	N/A
10576401	1700054N08Rik	N/A	N/A
10576403	Urb2	N/A	N/A
10576413	---	N/A	N/A
10576437	---	N/A	N/A
10576439	Cog2	N/A	N/A
10576482	Arv1	N/A	N/A
10576493	---	N/A	N/A
10576506	Gnpat	N/A	N/A
10576532	Tsnax	N/A	N/A
10576556	---	N/A	N/A
10576559	4933403G14Rik	N/A	N/A
10576581	Kcnk1	N/A	N/A
10576598	1810063B05Rik	N/A	N/A
10576608	---	N/A	N/A
10576610	Pard3	N/A	N/A
10576639	Nrp1	N/A	N/A
10576657	---	N/A	N/A
10576659	---	N/A	N/A
10576661	Itgb1	N/A	N/A
10576692	Insr	N/A	N/A
10576696	Insr	N/A	N/A
10576719	Pex11c	N/A	N/A
10576726	Rps23 /// Gm8618	N/A	N/A
10576728	Xab2	N/A	N/A
10576772	Clec4g	N/A	N/A
10576774	Clec4g	N/A	N/A
10576797	---	N/A	N/A
10576844	BC068157	N/A	N/A
10576854	Ctxn1	N/A	N/A
10576857	Timm44	N/A	N/A
10576881	Gm7461	N/A	N/A
10576896	Gm9457	N/A	N/A
10576899	---	N/A	N/A
10576901	Slc10a2	N/A	N/A
10576909	---	N/A	N/A
10576911	Efnb2	N/A	N/A
10576934	Fam155a	N/A	N/A
10576940	Fam155a /// LOC100504132	N/A	N/A
10576942	---	N/A	N/A
10576944	Gm10067	N/A	N/A
10576946	Lig4	N/A	N/A
10576951	Tnfsf13b	N/A	N/A
10576971	Irs2	N/A	N/A
10577048	Ankrd10	N/A	N/A
10577065	---	N/A	N/A
10577070	Tubgcp3	N/A	N/A
10577093	Gm15348	N/A	N/A
10577096	Pcid2	N/A	N/A
10577114	Grtp1	N/A	N/A
10577144	Dcun1d2 /// Tmco3	N/A	N/A
10577190	Rasa3	N/A	N/A
10577217	---	N/A	N/A
10577222	2410022L05Rik	N/A	N/A

10577226	2610019F03Rik	N/A	N/A
10577230	Erich1	N/A	N/A
10577240	Csmd1	N/A	N/A
10577349	Defb39	N/A	N/A
10577356	---	N/A	N/A
10577359	Defb8	N/A	N/A
10577388	Gm15319 /// 4930467E23Rik /// Trim44 /// Gm7827	N/A	N/A
10577395	2610005L07Rik /// 6820431F20Rik	N/A	N/A
10577412	2610005L07Rik /// 6820431F20Rik	N/A	N/A
10577419	---	N/A	N/A
10577421	Defa-rs1	N/A	N/A
10577426	Defa-rs1	N/A	N/A
10577429	Defa-rs1 /// Gm14851 /// AY761184 /// Gm7861 /// Gm7849	N/A	N/A
10577432	---	N/A	N/A
10577434	Defa-rs1 /// Gm14851 /// AY761184 /// Gm7861 /// Gm7849	N/A	N/A
10577444	Defb11	N/A	N/A
10577446	Defb15	N/A	N/A
10577449	Atp7b	N/A	N/A
10577517	Slc25a15	N/A	N/A
10577528	AI316807 /// Slc20a2	N/A	N/A
10577534	Vdac3	N/A	N/A
10577544	Polb	N/A	N/A
10577560	Ikbkb	N/A	N/A
10577586	Ap3m2	N/A	N/A
10577602	Mir486	N/A	N/A
10577604	Agpat6	N/A	N/A
10577641	1810011O10Rik	N/A	N/A
10577645	Ido2	N/A	N/A
10577757	Adam9	N/A	N/A
10577808	Tacc1	N/A	N/A
10577824	Letm2	N/A	N/A
10577838	Ddhd2	N/A	N/A
10577858	Bag4	N/A	N/A
10577866	Ash2l	N/A	N/A
10577882	Hgsnat	N/A	N/A
10577903	4930444A02Rik	N/A	N/A
10577910	Fnta	N/A	N/A
10577922	Hook3	N/A	N/A
10577946	Prosc	N/A	N/A
10577948	Brf2 /// Gpr124	N/A	N/A
10577954	Rab11fip1	N/A	N/A
10577990	---	N/A	N/A
10577992	---	N/A	N/A
10577996	Unc5d	N/A	N/A
10577999	Unc5d	N/A	N/A
10578017	Gm9911	N/A	N/A
10578019	Nudc	N/A	N/A
10578025	---	N/A	N/A
10578027	Mak16 /// BC019943	N/A	N/A
10578037	Eif3h	N/A	N/A
10578040	---	N/A	N/A
10578042	Tgs1	N/A	N/A
10578045	Nrg1	N/A	N/A
10578064	Rpl27a /// Gm5908 /// Gm5453	N/A	N/A
10578069	Gtpbp10	N/A	N/A
10578109	Ubxn8	N/A	N/A
10578123	Rbpms	N/A	N/A
10578138	Dctn6	N/A	N/A
10578145	Erh	N/A	N/A
10578149	Leprotl1	N/A	N/A
10578153	---	N/A	N/A
10578155	---	N/A	N/A
10578157	Tnks	N/A	N/A
10578191	---	N/A	N/A
10578193	Eri1	N/A	N/A
10578207	Lonrf1	N/A	N/A
10578219	---	N/A	N/A
10578241	Dlc1 /// A730069N07Rik	N/A	N/A
10578249	Gm10683	N/A	N/A
10578251	---	N/A	N/A
10578262	Mir383	N/A	N/A
10578277	---	N/A	N/A
10578279	Gm10682	N/A	N/A
10578287	Cnot7	N/A	N/A
10578320	---	N/A	N/A
10578322	Pdgfrl /// Gm9868	N/A	N/A
10578324	Mtus1	N/A	N/A
10578352	Fgl1	N/A	N/A
10578361	Asah1	N/A	N/A
10578377	Frg1	N/A	N/A
10578399	---	N/A	N/A
10578405	---	N/A	N/A
10578421	Adam34	N/A	N/A
10578423	Gm9908	N/A	N/A

10578425	AY512931	N/A	N/A
10578448	Cyp4v3 /// Klkb1	N/A	N/A
10578477	Fam149a	N/A	N/A
10578504	1700029J07Rik	N/A	N/A
10578515	Ankrd37	N/A	N/A
10578521	Snx25	N/A	N/A
10578539	Slc25a4	N/A	N/A
10578545	Gm12070 /// Gm2606 /// Gm2451	N/A	N/A
10578572	Stox2	N/A	N/A
10578574	Stox2	N/A	N/A
10578582	D030016E14Rik	N/A	N/A
10578613	Rps16 /// Rps16-ps2	N/A	N/A
10578619	Cdkn2aip	N/A	N/A
10578623	Wwc2	N/A	N/A
10578647	---	N/A	N/A
10578649	Odz3	N/A	N/A
10578679	Gm10675	N/A	N/A
10578681	---	N/A	N/A
10578683	Eif2s2 /// Gm9892	N/A	N/A
10578685	---	N/A	N/A
10578688	Rnu2	N/A	N/A
10578703	Spcs3	N/A	N/A
10578709	Wdr17	N/A	N/A
10578757	Fbxo8 /// BC088983	N/A	N/A
10578763	Sap30	N/A	N/A
10578768	---	N/A	N/A
10578771	Galnt7	N/A	N/A
10578786	Galntl6	N/A	N/A
10578794	Galntl6	N/A	N/A
10578796	Galntl6	N/A	N/A
10578810	Clcn3	N/A	N/A
10578872	---	N/A	N/A
10578902	Mir710	N/A	N/A
10578904	Cpe	N/A	N/A
10578916	Sc4mol	N/A	N/A
10578922	Klhl2	N/A	N/A
10578950	---	N/A	N/A
10578952	BC030870	N/A	N/A
10578955	---	N/A	N/A
10578957	---	N/A	N/A
10578959	---	N/A	N/A
10578962	Gm10661	N/A	N/A
10578964	1810029B16Rik	N/A	N/A
10578979	---	N/A	N/A
10578981	---	N/A	N/A
10578984	Tufm	N/A	N/A
10578986	Psd3	N/A	N/A
10578989	Psd3	N/A	N/A
10579012	Csgalnact1	N/A	N/A
10579043	Lzts1	N/A	N/A
10579047	---	N/A	N/A
10579049	Gm10033	N/A	N/A
10579052	Gm10033	N/A	N/A
10579054	Gm15319 /// 4930467E23Rik /// Trim44	N/A	N/A
10579060	Gm15319 /// 4930467E23Rik /// Trim44	N/A	N/A
10579066	Gm3365	N/A	N/A
10579070	Zfp868	N/A	N/A
10579079	Zfp869	N/A	N/A
10579089	Zfp866	N/A	N/A
10579119	Ndufa13 /// Tssk6	N/A	N/A
10579142	---	N/A	N/A
10579144	9130404D08Rik	N/A	N/A
10579165	Ncan	N/A	N/A
10579181	Rfxank /// Nr2c2ap	N/A	N/A
10579199	Slc25a42	N/A	N/A
10579234	Upf1	N/A	N/A
10579262	Crtc1	N/A	N/A
10579277	Klhl26	N/A	N/A
10579287	Tmem59l	N/A	N/A
10579296	2810428I15Rik	N/A	N/A
10579302	Uba52 /// Gm5239 /// Gm7866	N/A	N/A
10579335	Pgpep1	N/A	N/A
10579341	Mpv17l2	N/A	N/A
10579347	Ifi30	N/A	N/A
10579356	Pik3r2	N/A	N/A
10579373	Mast3	N/A	N/A
10579437	Ccdc124	N/A	N/A
10579468	Haus8	N/A	N/A
10579479	Nr2f6	N/A	N/A
10579500	Abhd8	N/A	N/A
10579508	Ano8 /// Dda1	N/A	N/A
10579525	Plvap	N/A	N/A
10579532	Bst2	N/A	N/A
10579538	Tmem221	N/A	N/A

10579548	Glt25d1	N/A	N/A
10579550	Unc13a	N/A	N/A
10579554	Unc13a	N/A	N/A
10579600	Jak3 /// Gm9933	N/A	N/A
10579607	Cnot8	N/A	N/A
10579659	Hmgn2 /// Gm6724	N/A	N/A
10579663	Eps15l1	N/A	N/A
10579724	Slc35e1	N/A	N/A
10579736	9130011J15Rik	N/A	N/A
10579744	Large	N/A	N/A
10579763	---	N/A	N/A
10579765	---	N/A	N/A
10579767	---	N/A	N/A
10579769	Gapdh /// Gm16374 /// Gm4609 /// Gm3200 /// Gm2451 /// Gm10293	N/A	N/A
10579772	---	N/A	N/A
10579774	Gm10649	N/A	N/A
10579799	Tmem184c	N/A	N/A
10579823	---	N/A	N/A
10579828	Rps25 /// Gm4963	N/A	N/A
10579830	Rbmxrt	N/A	N/A
10579833	Lsm6 /// Gm10043	N/A	N/A
10579839	Rpl21 /// Gm6813 /// Gm16416 /// Rpl21-ps12 /// Rpl21-ps14 /// Rpl21-ps10 /// Gm8054	N/A	N/A
10579852	Mmaa	N/A	N/A
10579860	Smad1	N/A	N/A
10579872	Tpd52	N/A	N/A
10579910	Zc3h15	N/A	N/A
10579913	---	N/A	N/A
10579915	Smarca5	N/A	N/A
10579925	Gab1	N/A	N/A
10579952	---	N/A	N/A
10579974	Tbc1d9 /// Gm10645	N/A	N/A
10579987	Scoc	N/A	N/A
10579991	---	N/A	N/A
10579993	Adam4 /// Gm4787	N/A	N/A
10579996	Tecr	N/A	N/A
10580010	Pkn1	N/A	N/A
10580033	Cd97	N/A	N/A
10580056	Gm10644 /// Lphn1	N/A	N/A
10580058	2210011C24Rik	N/A	N/A
10580077	Rln3	N/A	N/A
10580080	Gm10643	N/A	N/A
10580135	Mir181d	N/A	N/A
10580137	Mir181c	N/A	N/A
10580155	D8Ertd738e	N/A	N/A
10580160	Mri1	N/A	N/A
10580169	Ccdc130	N/A	N/A
10580183	Ier2	N/A	N/A
10580188	Nacc1	N/A	N/A
10580191	Nfix	N/A	N/A
10580219	Calr	N/A	N/A
10580231	---	N/A	N/A
10580233	Gcdh	N/A	N/A
10580247	Mast1	N/A	N/A
10580274	Rnaseh2a	N/A	N/A
10580300	Asna1	N/A	N/A
10580309	Wdr83	N/A	N/A
10580331	Vps35	N/A	N/A
10580382	Neto2	N/A	N/A
10580391	Itfg1	N/A	N/A
10580411	---	N/A	N/A
10580450	---	N/A	N/A
10580452	Siah1a /// Gm10638	N/A	N/A
10580457	N4bp1	N/A	N/A
10580467	Gm10637	N/A	N/A
10580469	Cbln1	N/A	N/A
10580473	Zfp423	N/A	N/A
10580486	Brd7	N/A	N/A
10580504	Snx20	N/A	N/A
10580516	Gm6625	N/A	N/A
10580519	---	N/A	N/A
10580522	Tox3	N/A	N/A
10580534	---	N/A	N/A
10580537	Aktip /// Rbl2	N/A	N/A
10580577	Irx3	N/A	N/A
10580590	Gapdh /// Gm16374 /// Gm2606 /// Gm4609 /// Gm3200 /// Gm2451 /// Gm10293	N/A	N/A
10580622	Ces1b	N/A	N/A
10580624	Ces1c	N/A	N/A
10580635	Ces1d	N/A	N/A
10580649	Ces1e	N/A	N/A
10580663	Ces1f	N/A	N/A
10580678	Ces1g	N/A	N/A
10580704	Amfr	N/A	N/A
10580722	Nudt21	N/A	N/A
10580733	Bbs2	N/A	N/A

10580752	9330175E14Rik	N/A	N/A
10580754	9330175E14Rik	N/A	N/A
10580756	Fam192a	N/A	N/A
10580765	Pllp	N/A	N/A
10580782	Polr2c /// Dok4	N/A	N/A
10580805	Gm10286	N/A	N/A
10580807	Kifc3	N/A	N/A
10580850	Cngb1	N/A	N/A
10580852	Cngb1	N/A	N/A
10580868	---	N/A	N/A
10580870	Zfp319	N/A	N/A
10580872	Zfp319	N/A	N/A
10580875	Gtl3	N/A	N/A
10580885	Csnk2a2	N/A	N/A
10580903	---	N/A	N/A
10580905	Cnot1	N/A	N/A
10580957	Slc38a7	N/A	N/A
10580969	Got2 /// Gm10874	N/A	N/A
10580981	---	N/A	N/A
10580984	---	N/A	N/A
10580986	Rbmxrt	N/A	N/A
10580990	Cdh8	N/A	N/A
10581009	LOC280487	N/A	N/A
10581013	Cdh11	N/A	N/A
10581027	Rplp0	N/A	N/A
10581031	---	N/A	N/A
10581033	---	N/A	N/A
10581036	Tk2	N/A	N/A
10581061	Cmtm4	N/A	N/A
10581069	Gm9853	N/A	N/A
10581073	Dync1li2	N/A	N/A
10581111	Nae1	N/A	N/A
10581181	Tradd	N/A	N/A
10581188	4931428F04Rik /// Nol3	N/A	N/A
10581212	Mir328	N/A	N/A
10581214	Lrrc29	N/A	N/A
10581266	Tppp3	N/A	N/A
10581271	Zdhhc1	N/A	N/A
10581289	Atp6v0d1	N/A	N/A
10581306	Rps2	N/A	N/A
10581308	Myl6 /// Gm8894	N/A	N/A
10581336	Gfod2	N/A	N/A
10581340	Ranbp10	N/A	N/A
10581355	Ctrl	N/A	N/A
10581363	Cenpt	N/A	N/A
10581388	Lcat	N/A	N/A
10581395	Slc12a4	N/A	N/A
10581448	---	N/A	N/A
10581450	Ddx28	N/A	N/A
10581455	Esrp2	N/A	N/A
10581473	Slc7a6os	N/A	N/A
10581491	A930006D01Rik	N/A	N/A
10581493	Pdxp	N/A	N/A
10581495	Rplp1	N/A	N/A
10581499	Chtf8	N/A	N/A
10581505	Rpl10	N/A	N/A
10581507	Cog8 /// Pdf /// Gm10627	N/A	N/A
10581523	Terf2	N/A	N/A
10581535	Rps18 /// Gm10260	N/A	N/A
10581547	Nob1	N/A	N/A
10581558	Rps26 /// Gm6654	N/A	N/A
10581560	Psmd7	N/A	N/A
10581569	---	N/A	N/A
10581571	4922502B01Rik	N/A	N/A
10581573	---	N/A	N/A
10581575	Wdr70 /// Gm1943	N/A	N/A
10581605	Hp	N/A	N/A
10581625	2400003C14Rik	N/A	N/A
10581636	Atxn1l	N/A	N/A
10581640	---	N/A	N/A
10581643	---	N/A	N/A
10581645	Marveld3	N/A	N/A
10581654	Calb2	N/A	N/A
10581674	Sf3b3	N/A	N/A
10581729	Ddx19a	N/A	N/A
10581737	Ddx19b	N/A	N/A
10581772	Glg1	N/A	N/A
10581800	Rfwd3	N/A	N/A
10581813	Mlkl	N/A	N/A
10581865	Ldhd	N/A	N/A
10581902	Cfdp1	N/A	N/A
10581910	Tmem170	N/A	N/A
10581917	Tmem231	N/A	N/A
10581926	Adat1	N/A	N/A

10581940	Kars	N/A	N/A
10581957	0610007P22Rik	N/A	N/A
10581990	---	N/A	N/A
10581992	Maf	N/A	N/A
10581996	Cdyl2	N/A	N/A
10582006	Gm10620	N/A	N/A
10582008	2310061C15Rik	N/A	N/A
10582069	Sdr42e1	N/A	N/A
10582074	Mphosph6	N/A	N/A
10582078	---	N/A	N/A
10582080	---	N/A	N/A
10582094	Mbtps1	N/A	N/A
10582162	Cotl1	N/A	N/A
10582171	Zdhhc7	N/A	N/A
10582188	Gse1 /// 1700016A09Rik	N/A	N/A
10582229	1110003O08Rik	N/A	N/A
10582231	Fbxo31	N/A	N/A
10582241	Zcchc14	N/A	N/A
10582260	---	N/A	N/A
10582275	Slc7a5	N/A	N/A
10582287	Car5a /// BC048644	N/A	N/A
10582295	Odc1	N/A	N/A
10582299	Trhr2	N/A	N/A
10582303	Cyba	N/A	N/A
10582310	Mvd	N/A	N/A
10582330	Rnf166	N/A	N/A
10582376	Fam38a	N/A	N/A
10582403	Galns	N/A	N/A
10582427	Cbfa2t3	N/A	N/A
10582445	Ankrd11	N/A	N/A
10582464	---	N/A	N/A
10582466	Sult5a1	N/A	N/A
10582477	Spata2L	N/A	N/A
10582549	---	N/A	N/A
10582551	Dbndd1	N/A	N/A
10582556	---	N/A	N/A
10582558	---	N/A	N/A
10582562	---	N/A	N/A
10582564	---	N/A	N/A
10582568	---	N/A	N/A
10582572	---	N/A	N/A
10582574	---	N/A	N/A
10582578	---	N/A	N/A
10582580	---	N/A	N/A
10582582	---	N/A	N/A
10582584	---	N/A	N/A
10582592	Acta1	N/A	N/A
10582599	Nup133	N/A	N/A
10582642	Taf5l	N/A	N/A
10582647	---	N/A	N/A
10582649	Pgbd5	N/A	N/A
10582658	Agt	N/A	N/A
10582664	2310022B05Rik	N/A	N/A
10582669	Ttc13	N/A	N/A
10582694	Fam89a	N/A	N/A
10582699	2810004N23Rik /// Gnpat	N/A	N/A
10582708	Exoc8	N/A	N/A
10582712	Egln1	N/A	N/A
10582719	Sipa1l2	N/A	N/A
10582743	Pcnxl2	N/A	N/A
10582809	Tk1	N/A	N/A
10582814	Tomm20	N/A	N/A
10582821	---	N/A	N/A
10582823	Rbm34	N/A	N/A
10582837	---	N/A	N/A
10582839	Gm9909	N/A	N/A
10582843	Itgb1	N/A	N/A
10582845	---	N/A	N/A
10582860	A530040E14Rik	N/A	N/A
10582862	Sp140 /// Arhgef12	N/A	N/A
10582874	Sp110	N/A	N/A
10582877	A530040E14Rik	N/A	N/A
10582879	Csprs /// Gm7609 /// Gm7592	N/A	N/A
10582882	---	N/A	N/A
10582884	---	N/A	N/A
10582888	---	N/A	N/A
10582890	---	N/A	N/A
10582896	---	N/A	N/A
10582916	---	N/A	N/A
10582918	---	N/A	N/A
10582925	Alkbh8	N/A	N/A
10582939	---	N/A	N/A
10582941	Cwf19l2	N/A	N/A
10582978	8430410K20Rik	N/A	N/A

10582983	LOC280487	N/A	N/A
10583021	Pdgfd	N/A	N/A
10583032	---	N/A	N/A
10583034	Dcun1d5	N/A	N/A
10583142	Rpl29 /// Gm3550 /// Rpl29-ps2	N/A	N/A
10583145	Tmem123	N/A	N/A
10583163	Trpc6	N/A	N/A
10583179	Pgr	N/A	N/A
10583195	Gm16485	N/A	N/A
10583197	---	N/A	N/A
10583199	---	N/A	N/A
10583201	---	N/A	N/A
10583203	Phxr4	N/A	N/A
10583207	Maml2	N/A	N/A
10583228	Fam76b	N/A	N/A
10583242	Sesn3	N/A	N/A
10583254	Cwc15	N/A	N/A
10583262	---	N/A	N/A
10583264	Mre11a	N/A	N/A
10583286	Gpr83	N/A	N/A
10583297	Taf1d	N/A	N/A
10583310	Taf1d	N/A	N/A
10583312	Taf1d	N/A	N/A
10583314	Taf1d	N/A	N/A
10583316	Taf1d	N/A	N/A
10583318	Taf1d	N/A	N/A
10583320	BC017612	N/A	N/A
10583324	---	N/A	N/A
10583340	Npm1	N/A	N/A
10583343	---	N/A	N/A
10583345	---	N/A	N/A
10583376	Olfr829	N/A	N/A
10583380	Olfr832	N/A	N/A
10583384	---	N/A	N/A
10583386	Olfr835	N/A	N/A
10583390	Olfr837	N/A	N/A
10583394	Olfr845	N/A	N/A
10583396	Olfr849	N/A	N/A
10583398	Olfr851	N/A	N/A
10583400	Olfr855	N/A	N/A
10583402	Zfp317	N/A	N/A
10583416	---	N/A	N/A
10583432	---	N/A	N/A
10583436	Olfr869	N/A	N/A
10583444	Olfr39	N/A	N/A
10583446	Olfr873	N/A	N/A
10583448	Rpl21	N/A	N/A
10583450	Zfp846	N/A	N/A
10583459	5730577I03Rik	N/A	N/A
10583465	Ubl5 /// Fbxl12	N/A	N/A
10583472	Pin1	N/A	N/A
10583485	Angptl6 /// A230050P20Rik	N/A	N/A
10583508	Mrpl4	N/A	N/A
10583519	Icam1	N/A	N/A
10583535	Icam5	N/A	N/A
10583548	Raver1	N/A	N/A
10583573	Kri1 /// Atg4d	N/A	N/A
10583586	Slc44a2	N/A	N/A
10583610	Ilf3	N/A	N/A
10583634	Qtrt1	N/A	N/A
10583647	Dnm2 /// Tmed1	N/A	N/A
10583676	Carm1 /// Yipf2	N/A	N/A
10583694	1810026J23Rik	N/A	N/A
10583697	Smarca4	N/A	N/A
10583732	Ldlr	N/A	N/A
10583753	Gm6484	N/A	N/A
10583759	Ccdc159	N/A	N/A
10583773	BC018242	N/A	N/A
10583785	2310047B19Rik	N/A	N/A
10583788	Prkcsh	N/A	N/A
10583806	---	N/A	N/A
10583820	Zfp872	N/A	N/A
10583825	Zfp809	N/A	N/A
10583847	Bbs9	N/A	N/A
10583887	Npsr1	N/A	N/A
10583898	Gm10701	N/A	N/A
10583900	Cypt4 /// Cypt9 /// Cypt10	N/A	N/A
10583903	---	N/A	N/A
10583905	Sep 07	N/A	N/A
10583920	Eepd1	N/A	N/A
10583929	B3gat1	N/A	N/A
10583942	Thyn1	N/A	N/A
10583952	Ncapd3	N/A	N/A
10583992	Igsf9b	N/A	N/A

10584013	---	N/A	N/A
10584024	Opcml	N/A	N/A
10584034	Snx19	N/A	N/A
10584057	Zbtb44	N/A	N/A
10584067	Zbtb44	N/A	N/A
10584069	---	N/A	N/A
10584071	Prdm10	N/A	N/A
10584095	Nfrkb	N/A	N/A
10584120	---	N/A	N/A
10584122	Rpl21	N/A	N/A
10584124	Arhgap32	N/A	N/A
10584162	Tmed2	N/A	N/A
10584165	Kirrel3	N/A	N/A
10584194	Srpr	N/A	N/A
10584200	Rpusd4	N/A	N/A
10584208	Cdon	N/A	N/A
10584229	---	N/A	N/A
10584231	Pus3	N/A	N/A
10584252	Gm9513	N/A	N/A
10584259	Fez1	N/A	N/A
10584271	Tmem218	N/A	N/A
10584276	Sec61g	N/A	N/A
10584280	Hepacam	N/A	N/A
10584309	BC024479	N/A	N/A
10584315	---	N/A	N/A
10584325	Vsig2	N/A	N/A
10584334	Siae	N/A	N/A
10584350	Tpt1p /// Tpt1 /// Gm6790	N/A	N/A
10584352	Olfr874	N/A	N/A
10584354	Olfr875	N/A	N/A
10584356	Olfr876	N/A	N/A
10584358	Olfr877	N/A	N/A
10584360	Olfr145	N/A	N/A
10584362	Olfr878	N/A	N/A
10584364	Olfr881	N/A	N/A
10584366	Olfr883	N/A	N/A
10584368	Olfr885 /// Olfr884	N/A	N/A
10584370	Olfr885 /// Olfr884	N/A	N/A
10584372	Olfr887	N/A	N/A
10584374	Olfr888	N/A	N/A
10584376	Olfr889	N/A	N/A
10584380	---	N/A	N/A
10584384	Olfr894	N/A	N/A
10584386	Olfr143	N/A	N/A
10584399	Olfr898	N/A	N/A
10584405	Olfr147	N/A	N/A
10584407	Olfr901	N/A	N/A
10584409	Olfr902	N/A	N/A
10584414	Olfr904	N/A	N/A
10584416	Olfr905	N/A	N/A
10584418	Olfr906	N/A	N/A
10584420	Olfr907	N/A	N/A
10584422	Olfr908	N/A	N/A
10584431	Olfr913	N/A	N/A
10584433	Olfr914	N/A	N/A
10584435	Vwa5a	N/A	N/A
10584458	Olfr920	N/A	N/A
10584460	Olfr921	N/A	N/A
10584466	Olfr923	N/A	N/A
10584470	Olfr933 /// Olfr26	N/A	N/A
10584479	Olfr933	N/A	N/A
10584481	---	N/A	N/A
10584484	Olfr242 /// Olfr27	N/A	N/A
10584488	Olfr944	N/A	N/A
10584492	Olfr954	N/A	N/A
10584494	Olfr148	N/A	N/A
10584498	Olfr961	N/A	N/A
10584502	Olfr965	N/A	N/A
10584504	Olfr150 /// Olfr919	N/A	N/A
10584506	Olfr967	N/A	N/A
10584508	Olfr969	N/A	N/A
10584510	Olfr970	N/A	N/A
10584512	Olfr971	N/A	N/A
10584514	Olfr972	N/A	N/A
10584518	Olfr974	N/A	N/A
10584520	Olfr978	N/A	N/A
10584522	Olfr981	N/A	N/A
10584526	Olfr982	N/A	N/A
10584535	---	N/A	N/A
10584549	Scn3b	N/A	N/A
10584561	9030425E11Rik	N/A	N/A
10584572	Hspa8 /// LOC624853	N/A	N/A
10584576	Hspa8 /// Snord14d /// Snord14c	N/A	N/A
10584578	Hspa8 /// Snord14d /// Snord14c	N/A	N/A

10584580	Snord14e	N/A	N/A
10584582	Gm10694	N/A	N/A
10584589	Mir100	N/A	N/A
10584591	Mirlet7a-2	N/A	N/A
10584593	---	N/A	N/A
10584595	2610203C20Rik	N/A	N/A
10584598	Mir125b-1	N/A	N/A
10584600	---	N/A	N/A
10584602	Gm10690	N/A	N/A
10584615	Pvrl1	N/A	N/A
10584628	Thy1	N/A	N/A
10584674	Mcam	N/A	N/A
10584698	Nlrx1	N/A	N/A
10584700	Dpagt1	N/A	N/A
10584710	H2afx	N/A	N/A
10584712	Hyou1	N/A	N/A
10584741	Slc37a4	N/A	N/A
10584752	Rps25 /// Gm4963	N/A	N/A
10584760	Gm10023	N/A	N/A
10584762	Bcl9l	N/A	N/A
10584777	Ddx6	N/A	N/A
10584803	Ift46	N/A	N/A
10584819	---	N/A	N/A
10584827	Mpzl2	N/A	N/A
10584835	Mpzl3	N/A	N/A
10584855	Scn2b	N/A	N/A
10584883	Fxyd6	N/A	N/A
10584941	Bace1	N/A	N/A
10584954	Pcsk7	N/A	N/A
10584977	Sik3	N/A	N/A
10585005	Apoa1	N/A	N/A
10585010	Apoa4	N/A	N/A
10585015	Apoa5	N/A	N/A
10585022	Zfp259	N/A	N/A
10585037	Bud13	N/A	N/A
10585048	Cadm1	N/A	N/A
10585068	Fam55d	N/A	N/A
10585091	Nhp2l1	N/A	N/A
10585097	Gm5617	N/A	N/A
10585129	Zw10	N/A	N/A
10585169	Drd2	N/A	N/A
10585186	1600029D21Rik	N/A	N/A
10585201	Timm8b /// AU019823	N/A	N/A
10585206	Pih1d2	N/A	N/A
10585233	Alg9	N/A	N/A
10585249	Ppp2r1b	N/A	N/A
10585282	2010007H06Rik	N/A	N/A
10585284	---	N/A	N/A
10585286	Arhgap20	N/A	N/A
10585301	9830163H01Rik /// Rdx	N/A	N/A
10585325	---	N/A	N/A
10585328	4930510E17Rik	N/A	N/A
10585331	Exph5	N/A	N/A
10585338	Kdelc2	N/A	N/A
10585358	Npat	N/A	N/A
10585377	---	N/A	N/A
10585395	Siva1	N/A	N/A
10585398	Gldn	N/A	N/A
10585417	Idh3a	N/A	N/A
10585428	Dnaja4	N/A	N/A
10585438	Crabp1	N/A	N/A
10585444	Ireb2	N/A	N/A
10585467	Agphd1	N/A	N/A
10585474	Psma4 /// AY074887	N/A	N/A
10585494	Ube2q2	N/A	N/A
10585509	Fbxo22	N/A	N/A
10585533	Dnajb6	N/A	N/A
10585545	Rcn2	N/A	N/A
10585572	Hmg20a	N/A	N/A
10585586	Ube2s	N/A	N/A
10585588	Cspg4	N/A	N/A
10585599	Imp3	N/A	N/A
10585610	Ptpn9	N/A	N/A
10585625	Sin3a	N/A	N/A
10585680	---	N/A	N/A
10585697	Gm5121	N/A	N/A
10585699	Fabp5 /// Fabp5l2	N/A	N/A
10585759	Edc3	N/A	N/A
10585767	Ubl7	N/A	N/A
10585823	1600029O15Rik	N/A	N/A
10585825	Stoml1	N/A	N/A
10585840	Cd276 /// Gm10657	N/A	N/A
10585842	Nptn	N/A	N/A
10585851	Hcn4	N/A	N/A

10585860	Adpgk	N/A	N/A
10585874	Hexa	N/A	N/A
10585889	Celf6	N/A	N/A
10585905	Parp6	N/A	N/A
10585932	Pkm2	N/A	N/A
10585940	4933407I18Rik	N/A	N/A
10585956	Myo9a	N/A	N/A
10585970	Myo9a	N/A	N/A
10585972	Myo9a	N/A	N/A
10585974	Myo9a	N/A	N/A
10585976	Myo9a	N/A	N/A
10585978	Myo9a	N/A	N/A
10585980	Myo9a	N/A	N/A
10585982	Myo9a	N/A	N/A
10585984	Myo9a	N/A	N/A
10585986	Myo9a	N/A	N/A
10585988	Myo9a	N/A	N/A
10585990	Myo9a	N/A	N/A
10585992	Myo9a	N/A	N/A
10586011	Larp6	N/A	N/A
10586015	1700036A12Rik	N/A	N/A
10586076	---	N/A	N/A
10586110	Cln6	N/A	N/A
10586126	---	N/A	N/A
10586157	Rpl4	N/A	N/A
10586166	---	N/A	N/A
10586168	Snord16a	N/A	N/A
10586170	---	N/A	N/A
10586172	---	N/A	N/A
10586174	---	N/A	N/A
10586176	Snapc5	N/A	N/A
10586180	Uchl3 /// Uchl4	N/A	N/A
10586184	Tipin	N/A	N/A
10586227	Dennd4a	N/A	N/A
10586244	Dennd4a	N/A	N/A
10586246	Dennd4a	N/A	N/A
10586248	Dennd4a	N/A	N/A
10586250	Dennd4a	N/A	N/A
10586252	Dennd4a	N/A	N/A
10586254	Dennd4a	N/A	N/A
10586284	Dpp8	N/A	N/A
10586306	Igdcc4	N/A	N/A
10586384	Pdcd7	N/A	N/A
10586397	Mtfmt	N/A	N/A
10586405	Spg21	N/A	N/A
10586433	Rbpms2	N/A	N/A
10586446	---	N/A	N/A
10586454	D030028M11Rik	N/A	N/A
10586458	Csnk1g1	N/A	N/A
10586477	Ppib /// Snx22	N/A	N/A
10586484	Fam96a	N/A	N/A
10586505	Herc1	N/A	N/A
10586602	Gm10647	N/A	N/A
10586604	Rps27l	N/A	N/A
10586609	---	N/A	N/A
10586614	C2cd4b	N/A	N/A
10586616	Vps13c	N/A	N/A
10586700	Rora	N/A	N/A
10586718	9530091C08Rik	N/A	N/A
10586722	F830001A07Rik	N/A	N/A
10586724	Narg2	N/A	N/A
10586759	Bnip2	N/A	N/A
10586781	Myo1e	N/A	N/A
10586812	---	N/A	N/A
10586814	---	N/A	N/A
10586816	Sltm	N/A	N/A
10586842	Fam63b /// Gm10642	N/A	N/A
10586844	Adam10	N/A	N/A
10586863	LOC280487	N/A	N/A
10586880	Zfp280d	N/A	N/A
10586920	Rfx7	N/A	N/A
10586933	Nedd4	N/A	N/A
10586967	Gm7265	N/A	N/A
10587000	Dyx1c1	N/A	N/A
10587010	---	N/A	N/A
10587012	Ccpg1	N/A	N/A
10587023	Rab27a	N/A	N/A
10587038	2410004A20Rik	N/A	N/A
10587042	Rsl24d1	N/A	N/A
10587049	---	N/A	N/A
10587075	---	N/A	N/A
10587077	Onecut1	N/A	N/A
10587082	Onecut1	N/A	N/A
10587085	BC031353	N/A	N/A

10587104	Arpp19	N/A	N/A
10587107	Myo5a	N/A	N/A
10587194	Gnb5	N/A	N/A
10587226	Lysmd2	N/A	N/A
10587231	Bmp5	N/A	N/A
10587241	Hmgcll1	N/A	N/A
10587266	Gclc	N/A	N/A
10587284	Elovl5	N/A	N/A
10587299	Ick	N/A	N/A
10587315	Gsta4	N/A	N/A
10587339	Gsta2	N/A	N/A
10587368	Mto1	N/A	N/A
10587419	Senp6	N/A	N/A
10587446	Myo6	N/A	N/A
10587486	Set /// BC085271	N/A	N/A
10587488	Mei4	N/A	N/A
10587495	Irak1bp1	N/A	N/A
10587501	Rps27a	N/A	N/A
10587503	Sh3bgrl2	N/A	N/A
10587532	Gm5919	N/A	N/A
10587534	Bckdhb	N/A	N/A
10587550	---	N/A	N/A
10587552	---	N/A	N/A
10587554	Tpbg	N/A	N/A
10587558	Dopey1 /// Pgm3	N/A	N/A
10587610	A330041J22Rik	N/A	N/A
10587616	Prss35	N/A	N/A
10587621	Ripply2	N/A	N/A
10587627	Cyb5r4	N/A	N/A
10587633	Mrap2	N/A	N/A
10587639	Nt5e	N/A	N/A
10587651	Rpl21	N/A	N/A
10587653	Snx14	N/A	N/A
10587655	4930422I07Rik	N/A	N/A
10587683	Bcl2a1b	N/A	N/A
10587686	---	N/A	N/A
10587688	---	N/A	N/A
10587690	Bcl2a1b	N/A	N/A
10587695	Gm2382 /// Mthfs	N/A	N/A
10587699	Rasgrf1	N/A	N/A
10587733	Ctsh	N/A	N/A
10587746	Tmem41b	N/A	N/A
10587776	---	N/A	N/A
10587780	Tuba1b /// Gm6682 /// Gm5620	N/A	N/A
10587782	Zic4	N/A	N/A
10587792	Plscr1	N/A	N/A
10587818	Plscr4	N/A	N/A
10587829	Plod2	N/A	N/A
10587852	---	N/A	N/A
10587854	Slc9a9	N/A	N/A
10587871	Paqr9	N/A	N/A
10587873	---	N/A	N/A
10587878	---	N/A	N/A
10587880	Pcolce2	N/A	N/A
10587892	Atr	N/A	N/A
10587940	---	N/A	N/A
10587942	Xrn1	N/A	N/A
10587988	Gk5	N/A	N/A
10588007	Tfdp2	N/A	N/A
10588024	Ppia	N/A	N/A
10588026	Rpl7a	N/A	N/A
10588035	---	N/A	N/A
10588043	Rbp2	N/A	N/A
10588049	Copb2	N/A	N/A
10588075	Prr23a /// Gm6406	N/A	N/A
10588077	2410012M07Rik	N/A	N/A
10588079	7420426K07Rik	N/A	N/A
10588091	Cep70	N/A	N/A
10588137	Dzip1l	N/A	N/A
10588154	Stag1	N/A	N/A
10588192	Msl2	N/A	N/A
10588201	---	N/A	N/A
10588223	Anapc13	N/A	N/A
10588226	Amotl2	N/A	N/A
10588243	Ryk	N/A	N/A
10588263	Slco2a1	N/A	N/A
10588283	Rab6b	N/A	N/A
10588324	---	N/A	N/A
10588357	Acad11 /// Ccrl1	N/A	N/A
10588403	Mrpl3	N/A	N/A
10588419	Aste1	N/A	N/A
10588429	Pik3r4	N/A	N/A
10588452	Glyctk /// Mir135a-1	N/A	N/A
10588454	Wdr82	N/A	N/A

10588464	Mirlet7g	N/A	N/A
10588482	Poc1a	N/A	N/A
10588495	Dusp7	N/A	N/A
10588499	Rpl29 /// Gm8580 /// Rpl29-ps2	N/A	N/A
10588505	Abhd14b	N/A	N/A
10588509	Pcbp4	N/A	N/A
10588542	Iqcf5	N/A	N/A
10588545	---	N/A	N/A
10588547	Vprbp	N/A	N/A
10588592	Cacna2d2	N/A	N/A
10588642	Nprl2	N/A	N/A
10588691	Hyal1 /// Nat6	N/A	N/A
10588696	Nat6 /// Hyal1 /// Hyal3	N/A	N/A
10588707	Ifrd2	N/A	N/A
10588722	Mon1a	N/A	N/A
10588755	Camkv	N/A	N/A
10588819	Cdhr4	N/A	N/A
10588826	Ip6k1	N/A	N/A
10588849	Amigo3	N/A	N/A
10588855	Mst1	N/A	N/A
10588874	Bsn	N/A	N/A
10588876	Nicn1	N/A	N/A
10588893	Rhoa	N/A	N/A
10588899	Gpx1	N/A	N/A
10588903	Usp4	N/A	N/A
10588975	Usp19	N/A	N/A
10589004	Qars	N/A	N/A
10589030	Qrich1	N/A	N/A
10589059	Mir425	N/A	N/A
10589061	Dalrd3 /// Wdr6	N/A	N/A
10589074	Arih2 /// Gm10621	N/A	N/A
10589087	Prkar2a	N/A	N/A
10589099	Ip6k2	N/A	N/A
10589128	---	N/A	N/A
10589130	Celsr3	N/A	N/A
10589190	---	N/A	N/A
10589192	Tmem89	N/A	N/A
10589196	Uqcrc1	N/A	N/A
10589327	Ucn2	N/A	N/A
10589368	Plxnb1	N/A	N/A
10589413	Nme6	N/A	N/A
10589420	Cdc25a	N/A	N/A
10589438	Mtap4	N/A	N/A
10589462	Mtap4	N/A	N/A
10589464	Gm10615	N/A	N/A
10589466	Smarcc1	N/A	N/A
10589494	Cspg5	N/A	N/A
10589503	2610002I17Rik	N/A	N/A
10589511	Scap	N/A	N/A
10589565	Setd2	N/A	N/A
10589587	Setd2	N/A	N/A
10589695	Rtp3	N/A	N/A
10589756	Epm2aip1	N/A	N/A
10589761	Trank1	N/A	N/A
10589773	Trank1	N/A	N/A
10589784	Dclk3	N/A	N/A
10589793	---	N/A	N/A
10589798	---	N/A	N/A
10589800	Clasp2	N/A	N/A
10589846	---	N/A	N/A
10589848	Ubp1 /// Fbxl2	N/A	N/A
10589870	---	N/A	N/A
10589884	Bcl2a1c	N/A	N/A
10589886	---	N/A	N/A
10589889	Glb1	N/A	N/A
10589907	Glb1	N/A	N/A
10589909	Trim71	N/A	N/A
10589911	---	N/A	N/A
10589913	Dync1li1	N/A	N/A
10589927	---	N/A	N/A
10589929	Cmtm6	N/A	N/A
10589938	Gpd1l	N/A	N/A
10589940	Osbpl10	N/A	N/A
10589955	Stt3b /// Gm9757	N/A	N/A
10589958	---	N/A	N/A
10589974	LOC280487	N/A	N/A
10589982	Azi2	N/A	N/A
10590004	Golga4	N/A	N/A
10590029	---	N/A	N/A
10590060	Ctdspl	N/A	N/A
10590071	Mir26a-1	N/A	N/A
10590135	LOC100038738	N/A	N/A
10590149	Xylb	N/A	N/A
10590169	Acvr2b	N/A	N/A

10590182	Exog	N/A	N/A
10590191	Wdr48 /// Gorasp1	N/A	N/A
10590245	Slc25a38	N/A	N/A
10590253	Rpsa /// Rpsa-ps10	N/A	N/A
10590263	---	N/A	N/A
10590265	---	N/A	N/A
10590267	Snora62	N/A	N/A
10590269	Mobp	N/A	N/A
10590277	---	N/A	N/A
10590279	Myrip	N/A	N/A
10590298	Eif1b	N/A	N/A
10590304	---	N/A	N/A
10590306	Entpd3	N/A	N/A
10590323	5830454E08Rik	N/A	N/A
10590325	Ctnnb1	N/A	N/A
10590343	Trak1	N/A	N/A
10590381	Vipr1	N/A	N/A
10590383	Deb1	N/A	N/A
10590407	Zfp651	N/A	N/A
10590433	1700048O20Rik	N/A	N/A
10590445	Snrk	N/A	N/A
10590452	Abhd5	N/A	N/A
10590462	Mir138-1	N/A	N/A
10590467	D9Ertd402e	N/A	N/A
10590479	Zfp167	N/A	N/A
10590487	Gm8008	N/A	N/A
10590529	Tmem42	N/A	N/A
10590549	Exosc7	N/A	N/A
10590563	Lars2	N/A	N/A
10590586	Limd1	N/A	N/A
10590597	Sacm1l	N/A	N/A
10590628	Ccr3	N/A	N/A
10590646	---	N/A	N/A
10590654	Aasdhppt	N/A	N/A
10590663	Gria4	N/A	N/A
10590690	Dync2h1	N/A	N/A
10590779	---	N/A	N/A
10590791	Birc2	N/A	N/A
10590799	C330006D17Rik	N/A	N/A
10590808	Yap1	N/A	N/A
10590821	9230110C19Rik	N/A	N/A
10590830	AK129341	N/A	N/A
10590844	Arhgap42	N/A	N/A
10590860	Arhgap42	N/A	N/A
10590865	Cntn5	N/A	N/A
10590888	---	N/A	N/A
10590890	---	N/A	N/A
10590909	Endod1	N/A	N/A
10590918	Amotl1	N/A	N/A
10590972	Mif	N/A	N/A
10591009	Med17	N/A	N/A
10591022	4931406C07Rik	N/A	N/A
10591035	5830418K08Rik	N/A	N/A
10591090	Fat3	N/A	N/A
10591092	Fat3	N/A	N/A
10591094	Fat3	N/A	N/A
10591110	Fat3	N/A	N/A
10591112	Fat3	N/A	N/A
10591114	Fat3	N/A	N/A
10591116	Fat3	N/A	N/A
10591118	Fat3	N/A	N/A
10591120	Fat3	N/A	N/A
10591123	Fat3	N/A	N/A
10591125	Fat3	N/A	N/A
10591127	Fat3	N/A	N/A
10591129	Fat3	N/A	N/A
10591131	Fat3	N/A	N/A
10591135	Fat3	N/A	N/A
10591164	Zfp558	N/A	N/A
10591180	Olfr828	N/A	N/A
10591182	---	N/A	N/A
10591184	---	N/A	N/A
10591190	Olfr846	N/A	N/A
10591194	Olfr850	N/A	N/A
10591198	Olfr854	N/A	N/A
10591200	Olfr856-ps1	N/A	N/A
10591208	Uba52 /// Gm5239 /// Gm7866	N/A	N/A
10591210	Olfr866	N/A	N/A
10591214	Olfr867	N/A	N/A
10591216	Olfr870	N/A	N/A
10591218	---	N/A	N/A
10591228	Zfp26	N/A	N/A
10591241	Zfp426	N/A	N/A
10591253	5730601F06Rik	N/A	N/A

10591263	Fbxl12	N/A	N/A
10591270	Olfm2	N/A	N/A
10591369	Dnmt1	N/A	N/A
10591416	---	N/A	N/A
10591430	Raver1	N/A	N/A
10591472	Cdc37	N/A	N/A
10591494	S1pr5	N/A	N/A
10591497	Kri1 /// Atg4d	N/A	N/A
10591517	Cdkn2d /// Gm4694	N/A	N/A
10591535	Mir199a-1	N/A	N/A
10591537	Tmed1	N/A	N/A
10591544	Yipf2 /// Carm1	N/A	N/A
10591563	Kank2	N/A	N/A
10591608	Dock6	N/A	N/A
10591610	Dock6	N/A	N/A
10591612	Dock6	N/A	N/A
10591614	Dock6	N/A	N/A
10591616	Dock6	N/A	N/A
10591618	Dock6	N/A	N/A
10591620	Dock6	N/A	N/A
10591624	Dock6	N/A	N/A
10591626	Dock6	N/A	N/A
10591628	Dock6	N/A	N/A
10591630	Dock6	N/A	N/A
10591634	Dock6	N/A	N/A
10591653	Tmem205	N/A	N/A
10591658	2310047B19Rik	N/A	N/A
10591706	Elavl3	N/A	N/A
10591715	Zfp653	N/A	N/A
10591726	Ecsit	N/A	N/A
10591735	Elof1	N/A	N/A
10591739	Acp5	N/A	N/A
10591747	Rpl15 /// Gm4294	N/A	N/A
10591749	1810064F22Rik	N/A	N/A
10591754	Zfp599	N/A	N/A
10591773	Hmgn2 /// Gm6724	N/A	N/A
10591781	Anln	N/A	N/A
10591806	rp9	N/A	N/A
10591814	---	N/A	N/A
10591867	Rpl41	N/A	N/A
10591869	Herpud2	N/A	N/A
10591881	Hmgb1	N/A	N/A
10591947	Acad8	N/A	N/A
10591960	Vps26b	N/A	N/A
10591978	Ntm	N/A	N/A
10591997	Adamts8 /// Gm10700	N/A	N/A
10591999	Gm9957	N/A	N/A
10592023	Aplp2	N/A	N/A
10592058	Tuba1b /// Gm5620	N/A	N/A
10592061	Kcnj5	N/A	N/A
10592099	Dcps	N/A	N/A
10592106	Tirap	N/A	N/A
10592114	Foxred1	N/A	N/A
10592138	Rpl36 /// Gm5614	N/A	N/A
10592154	Hyls1	N/A	N/A
10592160	Gm6762	N/A	N/A
10592162	---	N/A	N/A
10592186	Gm9896	N/A	N/A
10592217	Stt3a	N/A	N/A
10592237	Ei24	N/A	N/A
10592248	---	N/A	N/A
10592251	Pknox2	N/A	N/A
10592330	Nrgn	N/A	N/A
10592336	Spa17	N/A	N/A
10592342	Tbrg1	N/A	N/A
10592365	Olfr151	N/A	N/A
10592369	Olfr891	N/A	N/A
10592374	Olfr916	N/A	N/A
10592376	Olfr917	N/A	N/A
10592380	Olfr919	N/A	N/A
10592382	Olfr934	N/A	N/A
10592384	Olfr935	N/A	N/A
10592391	Olfr937	N/A	N/A
10592393	Olfr938	N/A	N/A
10592397	Olfr144	N/A	N/A
10592406	Olfr952	N/A	N/A
10592408	Olfr952 /// Olfr955	N/A	N/A
10592410	Olfr44	N/A	N/A
10592412	Olfr957	N/A	N/A
10592414	Olfr958	N/A	N/A
10592420	AW551984	N/A	N/A
10592447	---	N/A	N/A
10592449	Olfr149	N/A	N/A
10592451	Olfr968	N/A	N/A

10592455	Olfr975	N/A	N/A
10592457	Olfr976	N/A	N/A
10592459	---	N/A	N/A
10592461	Olfr979	N/A	N/A
10592463	Olfr980	N/A	N/A
10592465	Olfr983	N/A	N/A
10592467	Olfr984	N/A	N/A
10592471	Gramd1b	N/A	N/A
10592515	Ubash3b	N/A	N/A
10592531	---	N/A	N/A
10592533	---	N/A	N/A
10592535	Sorl1	N/A	N/A
10592585	Sc5d	N/A	N/A
10592618	Tbcel	N/A	N/A
10592629	Grik4	N/A	N/A
10592652	9530085L02Rik	N/A	N/A
10592719	Oaf	N/A	N/A
10592725	Gm10688	N/A	N/A
10592727	Rnf26	N/A	N/A
10592731	Gm10687	N/A	N/A
10592734	Cbl	N/A	N/A
10592772	Abcg4	N/A	N/A
10592790	Hinfp	N/A	N/A
10592802	C2cd2l	N/A	N/A
10592816	Hmbs	N/A	N/A
10592830	Vps11	N/A	N/A
10592847	Myl6 /// Gm8894	N/A	N/A
10592850	Trappc4	N/A	N/A
10592856	Ccdc84 /// Rps25	N/A	N/A
10592886	Gm10023	N/A	N/A
10592917	---	N/A	N/A
10592919	Arcn1	N/A	N/A
10592926	Tmem25	N/A	N/A
10592938	Ttc36	N/A	N/A
10592942	Mll1	N/A	N/A
10592983	Atp5l /// Gm5426	N/A	N/A
10592988	Ube4a	N/A	N/A
10593058	---	N/A	N/A
10593101	---	N/A	N/A
10593103	Rnf214	N/A	N/A
10593130	Sidt2	N/A	N/A
10593159	Pafah1b2	N/A	N/A
10593167	---	N/A	N/A
10593169	Apoc3	N/A	N/A
10593174	Apoa4	N/A	N/A
10593191	---	N/A	N/A
10593193	---	N/A	N/A
10593196	Gm10677	N/A	N/A
10593205	Rexo2	N/A	N/A
10593213	Rbm7	N/A	N/A
10593233	Htr3a	N/A	N/A
10593258	---	N/A	N/A
10593293	Ncam1	N/A	N/A
10593316	---	N/A	N/A
10593318	---	N/A	N/A
10593320	Rpl10	N/A	N/A
10593323	---	N/A	N/A
10593325	Pts	N/A	N/A
10593332	Bco2	N/A	N/A
10593356	Sdhd	N/A	N/A
10593361	AU019823	N/A	N/A
10593367	Dlat	N/A	N/A
10593413	2310030G06Rik	N/A	N/A
10593418	Cryab /// Hspb2	N/A	N/A
10593421	1110032A03Rik	N/A	N/A
10593430	Sik2 /// Ppp2r1b	N/A	N/A
10593469	Mir34c	N/A	N/A
10593471	Mir34b	N/A	N/A
10593473	1810046K07Rik	N/A	N/A
10593483	Fdx1	N/A	N/A
10593490	Gm6981 /// Gapdh	N/A	N/A
10593492	Zc3h12c	N/A	N/A
10593497	Zc3h12c	N/A	N/A
10593499	AI593442	N/A	N/A
10593506	---	N/A	N/A
10593508	Ddx10	N/A	N/A
10593526	Atm	N/A	N/A
10593589	Gm9963	N/A	N/A
10593591	Acat1	N/A	N/A
10593605	Cul5	N/A	N/A
10593634	Elmod1	N/A	N/A
10593668	Dmxl2	N/A	N/A
10593671	Dmxl2	N/A	N/A
10593713	Cib2 /// Sh2d7	N/A	N/A

10593723	Acsbg1	N/A	N/A
10593740	Wdr61	N/A	N/A
10593751	AY074887	N/A	N/A
10593756	Chrna3	N/A	N/A
10593789	Etfa	N/A	N/A
10593799	Scaper	N/A	N/A
10593832	---	N/A	N/A
10593842	Tspan3	N/A	N/A
10593856	C230081A13Rik	N/A	N/A
10593865	LOC236598	N/A	N/A
10593867	Lingo1	N/A	N/A
10593872	Odf3l1	N/A	N/A
10593878	Snx33	N/A	N/A
10593882	---	N/A	N/A
10593884	Ptpn9	N/A	N/A
10593887	Neil1	N/A	N/A
10593903	Commd4	N/A	N/A
10593913	1700017B05Rik	N/A	N/A
10593918	Ppcdc	N/A	N/A
10593927	Scamp5	N/A	N/A
10593937	Mpi	N/A	N/A
10593947	Cplx3	N/A	N/A
10593953	Lman1l	N/A	N/A
10593966	Csk	N/A	N/A
10593981	Cyp1a2	N/A	N/A
10593988	Clk3	N/A	N/A
10594001	Arid3b	N/A	N/A
10594015	Ccdc33	N/A	N/A
10594044	Islr	N/A	N/A
10594048	Islr2	N/A	N/A
10594053	Pml	N/A	N/A
10594066	Loxl1	N/A	N/A
10594080	Tbc1d21	N/A	N/A
10594092	Cd276	N/A	N/A
10594103	2410076I21Rik	N/A	N/A
10594110	Neo1	N/A	N/A
10594144	Bbs4	N/A	N/A
10594161	Arih1	N/A	N/A
10594177	Tmem202	N/A	N/A
10594183	Senp8	N/A	N/A
10594186	---	N/A	N/A
10594188	Nr2e3	N/A	N/A
10594199	Thsd4	N/A	N/A
10594221	Lrrc49	N/A	N/A
10594241	Gm9869	N/A	N/A
10594244	---	N/A	N/A
10594246	Gm10655	N/A	N/A
10594248	Rplp1	N/A	N/A
10594251	Kif23	N/A	N/A
10594277	Paqr5	N/A	N/A
10594289	Glce	N/A	N/A
10594297	Spesp1	N/A	N/A
10594301	Coro2b	N/A	N/A
10594315	Fem1b	N/A	N/A
10594320	Gm10653 /// Rps2 /// Rps2-ps6	N/A	N/A
10594322	Pias1	N/A	N/A
10594340	Skor1	N/A	N/A
10594353	Map2k5	N/A	N/A
10594377	2300009A05Rik	N/A	N/A
10594381	Iqch	N/A	N/A
10594418	Smad6	N/A	N/A
10594426	Zwilch /// Lctl	N/A	N/A
10594447	Map2k1	N/A	N/A
10594460	Dis3l	N/A	N/A
10594480	Rab11a	N/A	N/A
10594490	Slc24a1	N/A	N/A
10594513	---	N/A	N/A
10594517	Kbtbd13	N/A	N/A
10594519	Ostb	N/A	N/A
10594524	Ankdd1a	N/A	N/A
10594538	Plekho2	N/A	N/A
10594540	Plekho2	N/A	N/A
10594549	Gm10144	N/A	N/A
10594551	Zfp609	N/A	N/A
10594561	Trip4	N/A	N/A
10594582	Snx22 /// Ppib	N/A	N/A
10594590	Snx1	N/A	N/A
10594607	---	N/A	N/A
10594609	Fbxl22	N/A	N/A
10594613	Usp3	N/A	N/A
10594631	Aph1b	N/A	N/A
10594636	Ppp1r2	N/A	N/A
10594638	Aph1c	N/A	N/A
10594645	Rab8b	N/A	N/A

10594652	Lactb	N/A	N/A
10594661	Tpm1	N/A	N/A
10594679	Tln2	N/A	N/A
10594745	Mir190	N/A	N/A
10594747	C2cd4b	N/A	N/A
10594750	C2cd4a	N/A	N/A
10594752	Rpl7a /// Gm4978 /// Gm16477	N/A	N/A
10594754	Foxb1	N/A	N/A
10594758	Gcnt3	N/A	N/A
10594762	Fam81a	N/A	N/A
10594774	Ccnb2	N/A	N/A
10594785	Rnf111	N/A	N/A
10594798	---	N/A	N/A
10594800	Fam63b	N/A	N/A
10594802	Fam63b	N/A	N/A
10594812	Lipc	N/A	N/A
10594825	Aqp9	N/A	N/A
10594835	Grinl1a	N/A	N/A
10594840	Gcom1	N/A	N/A
10594855	Cgnl1	N/A	N/A
10594879	Tcf12	N/A	N/A
10594904	Uba52 /// Gm7866	N/A	N/A
10594907	Rpl7a	N/A	N/A
10594909	Gm10641	N/A	N/A
10594911	Tex9	N/A	N/A
10594928	Pigb /// Ccpg1	N/A	N/A
10594946	Rpl36 /// Gm13611 /// Gm5745	N/A	N/A
10594948	Unc13c	N/A	N/A
10594963	Unc13c	N/A	N/A
10594965	Unc13c	N/A	N/A
10594967	Unc13c	N/A	N/A
10594969	Unc13c	N/A	N/A
10594971	Unc13c	N/A	N/A
10594973	Unc13c	N/A	N/A
10594986	Rpl17	N/A	N/A
10594998	Leo1	N/A	N/A
10595000	Tmod3	N/A	N/A
10595013	Tmod2	N/A	N/A
10595033	Scg3	N/A	N/A
10595046	---	N/A	N/A
10595048	---	N/A	N/A
10595050	Gfral	N/A	N/A
10595059	Hcrtr2	N/A	N/A
10595070	Fam83b	N/A	N/A
10595079	---	N/A	N/A
10595081	Tinag	N/A	N/A
10595094	2310046A06Rik	N/A	N/A
10595109	Lrrc1	N/A	N/A
10595126	Fbxo9	N/A	N/A
10595140	Rn7sk	N/A	N/A
10595142	Omt2a	N/A	N/A
10595145	---	N/A	N/A
10595148	Gsta1	N/A	N/A
10595156	Dppa5a	N/A	N/A
10595159	Ooep	N/A	N/A
10595165	---	N/A	N/A
10595169	Ddx43	N/A	N/A
10595183	Eef1a1	N/A	N/A
10595189	Slc17a5	N/A	N/A
10595202	---	N/A	N/A
10595205	2410127L17Rik	N/A	N/A
10595209	Gm10635	N/A	N/A
10595211	Col12a1	N/A	N/A
10595280	Cox7a2	N/A	N/A
10595288	Tmem30a	N/A	N/A
10595298	Filip1	N/A	N/A
10595306	Impg1	N/A	N/A
10595324	Htr1b	N/A	N/A
10595327	Phip	N/A	N/A
10595371	Hmgn3	N/A	N/A
10595382	Lca5	N/A	N/A
10595392	Elovl4	N/A	N/A
10595400	---	N/A	N/A
10595404	Fam46a	N/A	N/A
10595407	Ibtk	N/A	N/A
10595439	---	N/A	N/A
10595441	Rpl7a /// Rpl7a-ps3 /// Rpl7a-ps5 /// Gm5619 /// Gm16477 /// Rpl7a-ps10	N/A	N/A
10595443	Tpbg	N/A	N/A
10595447	---	N/A	N/A
10595452	Ube2cbp	N/A	N/A
10595466	Pgm3 /// Dopey1	N/A	N/A
10595480	Me1	N/A	N/A
10595496	Snap91	N/A	N/A
10595529	4922501C03Rik	N/A	N/A

10595560	Tbx18	N/A	N/A
10595570	Slc25a40	N/A	N/A
10595573	Snx14	N/A	N/A
10595604	Syncrip	N/A	N/A
10595614	2810026P18Rik	N/A	N/A
10595620	---	N/A	N/A
10595622	---	N/A	N/A
10595624	---	N/A	N/A
10595626	---	N/A	N/A
10595628	---	N/A	N/A
10595630	Gm2382 /// Mthfs	N/A	N/A
10595633	Bcl2a1b	N/A	N/A
10595636	---	N/A	N/A
10595638	4921528I07Rik /// Gm10634 /// 4930579C12Rik	N/A	N/A
10595657	AF529169	N/A	N/A
10595664	Tmed3	N/A	N/A
10595668	Ankrd34c	N/A	N/A
10595671	Mir184	N/A	N/A
10595673	Morf4l1	N/A	N/A
10595680	Tbc1d2b	N/A	N/A
10595695	Zic1	N/A	N/A
10595702	1190002N15Rik	N/A	N/A
10595718	Chst2	N/A	N/A
10595723	2610101N10Rik	N/A	N/A
10595753	Trpc1	N/A	N/A
10595768	Pls1	N/A	N/A
10595787	1700065D16Rik /// Atr	N/A	N/A
10595791	---	N/A	N/A
10595793	Atp1b3	N/A	N/A
10595798	BC043934	N/A	N/A
10595803	Rnf7	N/A	N/A
10595805	Rasa2	N/A	N/A
10595831	Zbtb38	N/A	N/A
10595836	E030011O05Rik /// Zbtb38	N/A	N/A
10595840	Acpl2	N/A	N/A
10595848	Spsb4	N/A	N/A
10595856	Slc25a36	N/A	N/A
10595863	Trim42	N/A	N/A
10595871	Clstn2	N/A	N/A
10595892	2610303G11Rik	N/A	N/A
10595895	4930579K19Rik	N/A	N/A
10595900	Mrps22	N/A	N/A
10595909	---	N/A	N/A
10595911	Foxl2os	N/A	N/A
10595914	Gm1123	N/A	N/A
10595924	Pik3cb	N/A	N/A
10595948	Gm6432	N/A	N/A
10595953	Esyt3	N/A	N/A
10595979	Mras	N/A	N/A
10595981	Mras	N/A	N/A
10595990	Armc8	N/A	N/A
10596014	Cldn18	N/A	N/A
10596023	Sox14	N/A	N/A
10596027	Il20rb	N/A	N/A
10596034	Il20rb	N/A	N/A
10596041	Il20rb	N/A	N/A
10596043	Nck1	N/A	N/A
10596051	Tmem22	N/A	N/A
10596053	Pccb	N/A	N/A
10596070	---	N/A	N/A
10596072	Ppp2r3a	N/A	N/A
10596093	Gm5161	N/A	N/A
10596095	Ephb1	N/A	N/A
10596113	---	N/A	N/A
10596115	Ephb1	N/A	N/A
10596117	Cep63	N/A	N/A
10596119	Cep63	N/A	N/A
10596135	---	N/A	N/A
10596137	Srprb	N/A	N/A
10596148	Trf	N/A	N/A
10596166	1300017J02Rik	N/A	N/A
10596185	Cdv3	N/A	N/A
10596190	Bfsp2	N/A	N/A
10596200	Tmem108	N/A	N/A
10596207	Uba5	N/A	N/A
10596222	Ccrl1 /// Acad11	N/A	N/A
10596231	Dnajc13	N/A	N/A
10596255	Dnajc13	N/A	N/A
10596257	Dnajc13	N/A	N/A
10596259	Dnajc13	N/A	N/A
10596261	Dnajc13	N/A	N/A
10596263	Dnajc13	N/A	N/A
10596265	Dnajc13	N/A	N/A
10596267	Dnajc13	N/A	N/A

10596269	Dnajc13	N/A	N/A
10596271	Dnajc13	N/A	N/A
10596273	Dnajc13	N/A	N/A
10596275	Dnajc13	N/A	N/A
10596277	Dnajc13	N/A	N/A
10596279	Dnajc13	N/A	N/A
10596281	Dnajc13	N/A	N/A
10596303	Acpp	N/A	N/A
10596318	Nudt16	N/A	N/A
10596322	1700080E11Rik	N/A	N/A
10596327	Nek11	N/A	N/A
10596347	Atp2c1	N/A	N/A
10596377	---	N/A	N/A
10596379	Rpl10	N/A	N/A
10596381	---	N/A	N/A
10596383	Col6a6	N/A	N/A
10596394	Col6a6	N/A	N/A
10596403	Gm7455	N/A	N/A
10596409	Gm7455	N/A	N/A
10596428	Col6a4	N/A	N/A
10596433	Glyctk	N/A	N/A
10596442	Ppm1m	N/A	N/A
10596454	Alas1	N/A	N/A
10596465	Acy1	N/A	N/A
10596481	Abhd14a	N/A	N/A
10596489	Gpr62	N/A	N/A
10596492	Parp3	N/A	N/A
10596506	4930517N10Rik	N/A	N/A
10596509	Iqcf3	N/A	N/A
10596517	Iqcf4	N/A	N/A
10596521	Grm2	N/A	N/A
10596533	Tex264	N/A	N/A
10596543	Rad54l2	N/A	N/A
10596545	Rad54l2	N/A	N/A
10596568	Rbm15b	N/A	N/A
10596570	Rbm15b	N/A	N/A
10596575	Manf	N/A	N/A
10596583	Dock3	N/A	N/A
10596637	Mapkapk3	N/A	N/A
10596652	Hemk1	N/A	N/A
10596664	Cyb561d2	N/A	N/A
10596671	Rassf1 /// Gm9917 /// Hyal2	N/A	N/A
10596675	BY080835	N/A	N/A
10596680	Sema3b	N/A	N/A
10596704	Gnai2	N/A	N/A
10596718	Slc38a3	N/A	N/A
10596737	Gnat1	N/A	N/A
10596747	Sema3f	N/A	N/A
10596769	Rbm5	N/A	N/A
10596796	Rbm6	N/A	N/A
10596810	Rbm6	N/A	N/A
10596812	6230427J02Rik	N/A	N/A
10596815	Rnf123	N/A	N/A
10596857	Apeh	N/A	N/A
10596880	Bsn	N/A	N/A
10596893	Dag1	N/A	N/A
10596900	Tcta	N/A	N/A
10596904	Ccdc36	N/A	N/A
10596912	Klhdc8b	N/A	N/A
10596925	Ndufaf3	N/A	N/A
10596931	Wdr6	N/A	N/A
10596940	P4htm	N/A	N/A
10596951	Arih2	N/A	N/A
10596958	---	N/A	N/A
10596960	Trex1 /// Atrip	N/A	N/A
10596967	Atrip /// Trex1	N/A	N/A
10596982	Ccdc72	N/A	N/A
10596988	Plxnb1	N/A	N/A
10596990	Fbxw21	N/A	N/A
10597011	Fbxw20	N/A	N/A
10597020	Fbxw14 /// Fbxw22 /// Fbxw28	N/A	N/A
10597031	Fbxw22	N/A	N/A
10597036	Fbxw16 /// Fbxw19	N/A	N/A
10597064	Fbxw24	N/A	N/A
10597074	---	N/A	N/A
10597076	Fbxw18	N/A	N/A
10597088	Fbxw26	N/A	N/A
10597095	3000002C10Rik	N/A	N/A
10597098	Camp	N/A	N/A
10597103	Dhx30	N/A	N/A
10597132	---	N/A	N/A
10597134	Scap	N/A	N/A
10597136	Ptpn23	N/A	N/A
10597162	Klhl18	N/A	N/A

10597173	Nradd	N/A	N/A
10597239	Pth1r	N/A	N/A
10597266	Als2cl	N/A	N/A
10597268	Tdgf1	N/A	N/A
10597273	Rtp3	N/A	N/A
10597279	Ccrl2	N/A	N/A
10597288	Mlh1	N/A	N/A
10597309	Stac	N/A	N/A
10597323	Arpp21	N/A	N/A
10597352	Mir128-2	N/A	N/A
10597354	Pdcd6ip	N/A	N/A
10597377	Fbxl2 /// Ubp1	N/A	N/A
10597395	4921528I07Rik	N/A	N/A
10597413	Crtap	N/A	N/A
10597420	Ccr4	N/A	N/A
10597427	Trim71	N/A	N/A
10597432	Cnot10	N/A	N/A
10597459	---	N/A	N/A
10597461	Cmtm7	N/A	N/A
10597470	Cmtm8	N/A	N/A
10597477	Gpd1l	N/A	N/A
10597490	Rps27 /// Gm9846	N/A	N/A
10597493	Stt3b	N/A	N/A
10597511	Gm5921	N/A	N/A
10597513	---	N/A	N/A
10597515	Rpl24	N/A	N/A
10597518	Tgfbr2 /// Mib1	N/A	N/A
10597531	Rbms3	N/A	N/A
10597554	Zcwpw2	N/A	N/A
10597564	Cmc1	N/A	N/A
10597571	---	N/A	N/A
10597573	Eif1	N/A	N/A
10597575	Plcd1	N/A	N/A
10597592	Acaa1b	N/A	N/A
10597598	Slc22a14	N/A	N/A
10597612	Slc22a13	N/A	N/A
10597619	9330176C04Rik /// Slc22a13	N/A	N/A
10597627	Oxsr1	N/A	N/A
10597648	Myd88	N/A	N/A
10597656	Scn5a	N/A	N/A
10597686	Scn10a	N/A	N/A
10597714	Scn11a	N/A	N/A
10597743	Cx3cr1	N/A	N/A
10597748	Gorasp1 /// Wdr48	N/A	N/A
10597758	Csrnp1	N/A	N/A
10597764	Xirp1	N/A	N/A
10597770	Gm5922	N/A	N/A
10597773	---	N/A	N/A
10597775	Ulk4	N/A	N/A
10597817	Cck	N/A	N/A
10597823	Lyzl4	N/A	N/A
10597833	Sec22c	N/A	N/A
10597841	Hhatl	N/A	N/A
10597854	Ccdc13	N/A	N/A
10597871	Higd1a	N/A	N/A
10597875	Cyp8b1	N/A	N/A
10597878	C85492	N/A	N/A
10597883	Ano10	N/A	N/A
10597898	---	N/A	N/A
10597900	Zfp445	N/A	N/A
10597913	1110059G10Rik	N/A	N/A
10597918	Gm9856	N/A	N/A
10597920	Zdhhc3	N/A	N/A
10597933	---	N/A	N/A
10597935	Cdcp1	N/A	N/A
10597945	Tmem158	N/A	N/A
10597949	Slc6a20b	N/A	N/A
10597960	Slc6a20a	N/A	N/A
10597969	Hnrnpa1 /// Gm10052	N/A	N/A
10597973	Lztfl1	N/A	N/A
10597978	Fyco1	N/A	N/A
10597996	Xcr1	N/A	N/A
10597999	---	N/A	N/A
10598004	Ccr1	N/A	N/A
10598010	Ccr1l1	N/A	N/A
10598013	Ccr5 /// Ccr2	N/A	N/A
10598018	---	N/A	N/A
10598020	---	N/A	N/A
10598023	---	N/A	N/A
10598025	---	N/A	N/A
10598027	---	N/A	N/A
10598029	ND1	N/A	N/A
10598032	---	N/A	N/A
10598036	COX1	N/A	N/A

10598038	Prf1 /// COX2	N/A	N/A
10598041	---	N/A	N/A
10598043	ATP6 /// Gm10925 /// ATP8 /// COX3	N/A	N/A
10598049	COX3 /// ND3	N/A	N/A
10598053	---	N/A	N/A
10598055	ND3	N/A	N/A
10598057	---	N/A	N/A
10598059	ND4L /// ND4	N/A	N/A
10598062	---	N/A	N/A
10598064	---	N/A	N/A
10598067	ND5	N/A	N/A
10598069	CYTB	N/A	N/A
10598071	---	N/A	N/A
10598073	---	N/A	N/A
10598075	---	N/A	N/A
10598077	---	N/A	N/A
10598079	---	N/A	N/A
10598081	---	N/A	N/A
10598083	LOC100503984 /// LOC100504272	N/A	N/A
10598085	---	N/A	N/A
10598087	ND6	N/A	N/A
10598089	---	N/A	N/A
10598091	---	N/A	N/A
10598093	Tarm1	N/A	N/A
10598101	Maml2	N/A	N/A
10598105	Olfr239	N/A	N/A
10598107	Taf1a	N/A	N/A
10598111	Dhrsx	N/A	N/A
10598126	Hhipl2	N/A	N/A
10598138	Spry3	N/A	N/A
10598146	Tcstv3	N/A	N/A
10598152	LOC625360	N/A	N/A
10598154	AF067063	N/A	N/A
10598164	0610010K06Rik	N/A	N/A
10598169	4930449I24Rik /// Gm6370 /// Gm3402 /// Gm3409 /// Gm3415	N/A	N/A
10598175	Ear2	N/A	N/A
10598178	Disp1	N/A	N/A
10598180	BC085271	N/A	N/A
10598192	4930408F14Rik /// Gm2790 /// Gm5923 /// Gm5926 /// Gm2940	N/A	N/A
10598198	Mia3	N/A	N/A
10598203	Ccl28	N/A	N/A
10598212	---	N/A	N/A
10598216	---	N/A	N/A
10598222	4930408F14Rik /// Gm2790 /// Gm5923	N/A	N/A
10598227	4930408F14Rik /// Gm2790 /// Gm5923 /// Gm5926 /// Gm2940	N/A	N/A
10598232	---	N/A	N/A
10598236	Nudt11	N/A	N/A
10598238	---	N/A	N/A
10598240	Shroom4	N/A	N/A
10598251	Dgkk	N/A	N/A
10598279	Akap4	N/A	N/A
10598287	LOC100270707	N/A	N/A
10598289	4930524L23Rik	N/A	N/A
10598292	Foxp3	N/A	N/A
10598309	Cacna1f	N/A	N/A
10598359	Syp	N/A	N/A
10598369	Prickle3	N/A	N/A
10598381	Gpkow	N/A	N/A
10598389	Wdr45	N/A	N/A
10598403	Praf2	N/A	N/A
10598407	---	N/A	N/A
10598409	Tcfe3	N/A	N/A
10598422	Gripap1	N/A	N/A
10598448	Kcnd1	N/A	N/A
10598467	Pim2	N/A	N/A
10598474	Slc35a2	N/A	N/A
10598482	Timm17b	N/A	N/A
10598491	Gm10490	N/A	N/A
10598493	Pcsk1n	N/A	N/A
10598499	---	N/A	N/A
10598505	Rbm3 /// 2900002K06Rik	N/A	N/A
10598507	Slc38a5	N/A	N/A
10598524	Ssxb10	N/A	N/A
10598528	Ssxb3 /// Ssxb2 /// Ssxb1 /// Ssx9 /// Ssxb5 /// Ssxb9 /// Ssxb10 /// Ssxb11 /// Ssxb12 /// Ssxb8 /// Gm14459 /// Gm6592	N/A	N/A
10598538	Ssxb3	N/A	N/A
10598547	---	N/A	N/A
10598549	Ssx9	N/A	N/A
10598555	Ssxb5	N/A	N/A
10598562	B630019K06Rik	N/A	N/A
10598565	Fthl17 /// Gm5635	N/A	N/A
10598567	Fthl17	N/A	N/A
10598569	Gm5635	N/A	N/A
10598571	4930402K13Rik	N/A	N/A
10598573	---	N/A	N/A

10598575	Lancl3	N/A	N/A
10598592	Gm14501 /// Gm14484 /// Gm14483 /// 1700012L04Rik	N/A	N/A
10598594	---	N/A	N/A
10598596	Gm14501 /// Gm14484 /// Gm14483 /// 1700012L04Rik	N/A	N/A
10598598	1700054O13Rik	N/A	N/A
10598601	4930557A04Rik	N/A	N/A
10598612	Otc	N/A	N/A
10598626	Tspan7	N/A	N/A
10598636	Ube2i	N/A	N/A
10598638	Mid1ip1	N/A	N/A
10598644	Gm14501 /// Gm14484 /// Gm14483 /// 1700012L04Rik	N/A	N/A
10598646	Gm14501 /// Gm14484 /// Gm14483 /// 1700012L04Rik	N/A	N/A
10598648	Gm14501 /// Gm14484 /// Gm14483 /// 1700012L04Rik	N/A	N/A
10598650	Gm14501 /// Gm14484 /// Gm14483 /// 1700012L04Rik	N/A	N/A
10598652	Gm14501 /// Gm14484 /// Gm14483 /// 1700012L04Rik	N/A	N/A
10598654	Gm14501 /// Gm14484 /// Gm14483 /// 1700012L04Rik	N/A	N/A
10598656	Gm14501 /// Gm14484 /// Gm14483 /// 1700012L04Rik	N/A	N/A
10598658	Gm14501 /// Gm14484 /// Gm14483 /// 1700012L04Rik	N/A	N/A
10598660	Gm14501 /// Gm14484 /// Gm14483 /// 1700012L04Rik	N/A	N/A
10598662	---	N/A	N/A
10598664	Atp6ap2	N/A	N/A
10598674	LOC634012	N/A	N/A
10598678	Usp9x	N/A	N/A
10598721	Rpl3	N/A	N/A
10598723	Ddx3x	N/A	N/A
10598743	Nyx	N/A	N/A
10598750	Gpr34	N/A	N/A
10598757	Gpr82	N/A	N/A
10598763	Gm14501 /// Gm14484 /// Gm5382 /// 1700012L04Rik	N/A	N/A
10598765	Cypt1 /// Cypt8 /// Cypt7	N/A	N/A
10598769	---	N/A	N/A
10598771	Maoa	N/A	N/A
10598789	---	N/A	N/A
10598791	Gm5073	N/A	N/A
10598794	---	N/A	N/A
10598796	Dusp21	N/A	N/A
10598798	Kdm6a	N/A	N/A
10598827	---	N/A	N/A
10598831	Rpl23a /// Gm10335	N/A	N/A
10598833	Chst7	N/A	N/A
10598837	---	N/A	N/A
10598839	Rp2h	N/A	N/A
10598848	Phf16	N/A	N/A
10598863	Rgn	N/A	N/A
10598872	Rbm10	N/A	N/A
10598882	Uba1	N/A	N/A
10598912	Cdk16	N/A	N/A
10598933	Usp11	N/A	N/A
10598956	Araf	N/A	N/A
10598976	Timp1	N/A	N/A
10598983	Spaca5	N/A	N/A
10598989	Ssxa1	N/A	N/A
10598994	Atp1b3	N/A	N/A
10598996	Gm5124	N/A	N/A
10599001	Agtr2	N/A	N/A
10599005	---	N/A	N/A
10599008	Slc6a14	N/A	N/A
10599026	---	N/A	N/A
10599028	1700023I07Rik	N/A	N/A
10599030	---	N/A	N/A
10599032	Wdr44	N/A	N/A
10599054	Gm4907	N/A	N/A
10599058	---	N/A	N/A
10599060	---	N/A	N/A
10599062	---	N/A	N/A
10599073	---	N/A	N/A
10599084	4930408F14Rik /// Gm2790 /// Gm5923 /// Gm5926 /// Gm2940	N/A	N/A
10599088	4930408F14Rik /// Gm2790 /// Gm5923 /// Gm5926 /// Gm2940	N/A	N/A
10599094	---	N/A	N/A
10599105	---	N/A	N/A
10599116	---	N/A	N/A
10599118	---	N/A	N/A
10599120	Dock11	N/A	N/A
10599174	Il13ra1	N/A	N/A
10599187	Zcchc12	N/A	N/A
10599192	Lonrf3	N/A	N/A
10599200	Pgrmc1	N/A	N/A
10599207	Slc25a43	N/A	N/A
10599213	---	N/A	N/A
10599215	Slc25a5	N/A	N/A
10599222	Ube2a	N/A	N/A
10599229	Ankrd58	N/A	N/A
10599232	Nkap	N/A	N/A
10599243	Rnf113a1	N/A	N/A

10599246	Rhox2a /// Rhox2e /// Rhox2f /// Rhox2c /// Rhox2h /// Rhox2g /// Rhox2b /// Rhox2d	N/A	N/A
10599251	Rhox3c	N/A	N/A
10599263	Rhox4b	N/A	N/A
10599269	Rhox4b /// Rhox4e /// Rhox4d /// Rhox4a /// Rhox4f /// Rhox4g	N/A	N/A
10599274	Rhox4b /// Rhox4e /// Rhox4a /// Rhox4d /// Rhox4c /// Rhox4g	N/A	N/A
10599281	Rhox4b /// Rhox4e /// Rhox4d /// Rhox4a /// Rhox4g	N/A	N/A
10599286	Rhox2a	N/A	N/A
10599291	Rhox4b /// Rhox4e /// Rhox4d /// Rhox4a /// Rhox4f /// Rhox4c /// Rhox4g	N/A	N/A
10599296	Rhox6	N/A	N/A
10599300	Rhox7	N/A	N/A
10599321	Zbtb33	N/A	N/A
10599324	Atp1b4	N/A	N/A
10599333	Atp5l	N/A	N/A
10599335	Mcts1	N/A	N/A
10599342	6030498E09Rik	N/A	N/A
10599346	Polr2k	N/A	N/A
10599348	Gria3	N/A	N/A
10599369	Xiap	N/A	N/A
10599377	Stag2	N/A	N/A
10599411	Sh2d1a	N/A	N/A
10599416	Gm10483	N/A	N/A
10599418	---	N/A	N/A
10599420	Gm362 /// LOC637093	N/A	N/A
10599422	1110059M19Rik	N/A	N/A
10599425	Actrt1	N/A	N/A
10599427	---	N/A	N/A
10599430	---	N/A	N/A
10599433	Rpl21	N/A	N/A
10599435	Ocrl	N/A	N/A
10599461	Calm2	N/A	N/A
10599463	Xpnpep2	N/A	N/A
10599487	Sash3	N/A	N/A
10599496	---	N/A	N/A
10599530	Rab33a	N/A	N/A
10599537	Slc25a14	N/A	N/A
10599554	Rbmx2	N/A	N/A
10599560	---	N/A	N/A
10599562	Arhgap36	N/A	N/A
10599576	Ppdpf	N/A	N/A
10599579	Olfr1320	N/A	N/A
10599581	2610018G03Rik	N/A	N/A
10599595	---	N/A	N/A
10599598	---	N/A	N/A
10599604	Ccdc160	N/A	N/A
10599607	Rps2	N/A	N/A
10599612	Phf6	N/A	N/A
10599627	Hprt	N/A	N/A
10599637	Fam122c	N/A	N/A
10599648	---	N/A	N/A
10599650	Etd	N/A	N/A
10599654	Cxx1c	N/A	N/A
10599658	4933416I08Rik /// LOC100504039	N/A	N/A
10599670	---	N/A	N/A
10599673	Slxl1 /// Gm16405 /// Gm16430	N/A	N/A
10599680	3830403N18Rik	N/A	N/A
10599686	Zfp449	N/A	N/A
10599693	Ncrna00086	N/A	N/A
10599696	Ddx26b	N/A	N/A
10599717	---	N/A	N/A
10599719	Slc9a6	N/A	N/A
10599736	Fhl1	N/A	N/A
10599747	Gpr112	N/A	N/A
10599773	Gpr112	N/A	N/A
10599776	Brs3	N/A	N/A
10599781	Htatsf1	N/A	N/A
10599792	Vgll1	N/A	N/A
10599802	Cd40lg	N/A	N/A
10599810	---	N/A	N/A
10599812	Zic3	N/A	N/A
10599822	4930550L24Rik	N/A	N/A
10599826	F9	N/A	N/A
10599835	---	N/A	N/A
10599839	---	N/A	N/A
10599841	C230004F18Rik	N/A	N/A
10599849	Gm4988	N/A	N/A
10599853	Ldoc1	N/A	N/A
10599855	Eif4e	N/A	N/A
10599858	4933402E13Rik	N/A	N/A
10599863	4931400O07Rik	N/A	N/A
10599866	3830417A13Rik	N/A	N/A
10599871	---	N/A	N/A
10599874	---	N/A	N/A
10599876	Ctag2	N/A	N/A
10599880	Slitrk2	N/A	N/A

10599884	Slitrk2	N/A	N/A
10599888	---	N/A	N/A
10599893	Fmr1	N/A	N/A
10599917	Fmr1nb	N/A	N/A
10599925	Styx	N/A	N/A
10599927	Aff2	N/A	N/A
10599948	1700020N15Rik /// Gm6812	N/A	N/A
10599951	1110012L19Rik	N/A	N/A
10599956	---	N/A	N/A
10599958	Gm16441	N/A	N/A
10599960	---	N/A	N/A
10599962	Mamld1	N/A	N/A
10599972	Hnrnpa3	N/A	N/A
10599976	Mtm1	N/A	N/A
10599997	Mtmr1	N/A	N/A
10600017	Hmgb3	N/A	N/A
10600024	Gpr50	N/A	N/A
10600027	Rpl30	N/A	N/A
10600034	Gm1141	N/A	N/A
10600044	Prrg3	N/A	N/A
10600047	Fate1	N/A	N/A
10600057	Cnga2	N/A	N/A
10600065	Magea4	N/A	N/A
10600067	---	N/A	N/A
10600069	Gabrq	N/A	N/A
10600080	Magea9-ps	N/A	N/A
10600082	Nsdhl	N/A	N/A
10600093	Zfp185	N/A	N/A
10600114	Pnma3	N/A	N/A
10600116	Gm14685 /// Gm5640 /// DXBay18	N/A	N/A
10600122	Xlr3a /// Xlr3c /// Xlr3b	N/A	N/A
10600131	Xlr4b /// Xlr4c /// Xlr4a	N/A	N/A
10600144	F8a	N/A	N/A
10600148	Gm14685 /// Gm5640 /// DXBay18	N/A	N/A
10600150	Zfp275	N/A	N/A
10600159	Zfp92	N/A	N/A
10600169	Bgn	N/A	N/A
10600180	Atp2b3	N/A	N/A
10600205	Dusp9	N/A	N/A
10600210	Slc6a8	N/A	N/A
10600235	Abcd1	N/A	N/A
10600247	---	N/A	N/A
10600249	Plxnb3	N/A	N/A
10600284	Srpk3	N/A	N/A
10600301	Ssr4	N/A	N/A
10600308	---	N/A	N/A
10600310	Avpr2 /// Arhgap4	N/A	N/A
10600317	Opn1mw	N/A	N/A
10600324	Rpl3	N/A	N/A
10600326	Tktl1	N/A	N/A
10600341	Emd	N/A	N/A
10600349	Rpl10	N/A	N/A
10600355	Snora70	N/A	N/A
10600357	Taz	N/A	N/A
10600372	B230340J04Rik	N/A	N/A
10600377	Atp6ap1	N/A	N/A
10600390	Gdi1	N/A	N/A
10600403	Fam50a	N/A	N/A
10600419	Plxna3	N/A	N/A
10600453	Ikbkg	N/A	N/A
10600471	---	N/A	N/A
10600474	---	N/A	N/A
10600476	---	N/A	N/A
10600478	Olfr1325	N/A	N/A
10600480	4930408F14Rik /// Gm2790 /// Gm5923 /// Gm5926 /// Gm2940	N/A	N/A
10600482	---	N/A	N/A
10600485	Dkc1	N/A	N/A
10600500	2810453I06Rik	N/A	N/A
10600502	2810453I06Rik	N/A	N/A
10600504	Fundc2	N/A	N/A
10600512	Brcc3	N/A	N/A
10600524	Vbp1	N/A	N/A
10600529	---	N/A	N/A
10600531	---	N/A	N/A
10600533	---	N/A	N/A
10600536	Gm4937	N/A	N/A
10600539	Magea7-ps	N/A	N/A
10600541	4930428D18Rik	N/A	N/A
10600547	Tbl1x	N/A	N/A
10600566	---	N/A	N/A
10600568	Gm14744	N/A	N/A
10600576	Gm14743 /// Obp1b	N/A	N/A
10600581	4930480E11Rik	N/A	N/A
10600584	---	N/A	N/A

10600586	Fam47a-ps	N/A	N/A
10600588	---	N/A	N/A
10600593	Hnrnpa3	N/A	N/A
10600597	Tmem47	N/A	N/A
10600602	---	N/A	N/A
10600604	Dmd	N/A	N/A
10600685	1600014K23Rik	N/A	N/A
10600688	Tab3	N/A	N/A
10600698	5430427O19Rik	N/A	N/A
10600703	1700072E05Rik	N/A	N/A
10600710	---	N/A	N/A
10600712	---	N/A	N/A
10600714	Smek3-ps	N/A	N/A
10600718	Sec61g	N/A	N/A
10600720	---	N/A	N/A
10600723	---	N/A	N/A
10600730	Gm5072	N/A	N/A
10600733	Gm5072	N/A	N/A
10600736	Mageb17-ps	N/A	N/A
10600741	Gm5941	N/A	N/A
10600744	---	N/A	N/A
10600746	1700003E24Rik /// 1700084M14Rik	N/A	N/A
10600750	1700003E24Rik /// 1700084M14Rik	N/A	N/A
10600753	---	N/A	N/A
10600755	Arx	N/A	N/A
10600763	Gm6985	N/A	N/A
10600765	Pcyt1b	N/A	N/A
10600777	AU015836	N/A	N/A
10600781	Gm4990	N/A	N/A
10600786	Klhl15	N/A	N/A
10600797	Apoo /// Apoo-ps	N/A	N/A
10600810	---	N/A	N/A
10600814	Gspt2	N/A	N/A
10600819	Zxda /// Zxdb	N/A	N/A
10600821	---	N/A	N/A
10600823	LOC675747	N/A	N/A
10600825	Zc3h12b	N/A	N/A
10600836	Msn	N/A	N/A
10600852	Mir223 /// F630028O10Rik	N/A	N/A
10600857	Heph	N/A	N/A
10600884	Rpl17 /// Gm10268 /// Rpl17-ps3	N/A	N/A
10600886	Gpr165	N/A	N/A
10600892	Pgr15l	N/A	N/A
10600899	---	N/A	N/A
10600901	Ar	N/A	N/A
10600911	Yipf6	N/A	N/A
10600921	Stard8	N/A	N/A
10600936	Efnb1	N/A	N/A
10600947	Tmem28	N/A	N/A
10600953	Eda	N/A	N/A
10600969	Mir676	N/A	N/A
10600971	Otud6a	N/A	N/A
10600973	Igbp1	N/A	N/A
10600980	Dgat2l6	N/A	N/A
10600988	Awat1	N/A	N/A
10600994	Arr3	N/A	N/A
10601011	Kif4	N/A	N/A
10601044	Gdpd2	N/A	N/A
10601062	Dlg3	N/A	N/A
10601086	---	N/A	N/A
10601088	---	N/A	N/A
10601091	Foxo4	N/A	N/A
10601150	---	N/A	N/A
10601152	Nlgn3	N/A	N/A
10601161	Gjb1	N/A	N/A
10601164	Nono	N/A	N/A
10601178	Itgb1bp2	N/A	N/A
10601192	Taf1	N/A	N/A
10601235	Ogt	N/A	N/A
10601264	Tpt1 /// Tpt1p	N/A	N/A
10601266	8030474K03Rik	N/A	N/A
10601270	Gm10456	N/A	N/A
10601272	Pin4	N/A	N/A
10601277	---	N/A	N/A
10601280	Dmrtc1b	N/A	N/A
10601285	Dmrtc1c /// Dmrtc1c2	N/A	N/A
10601296	Pabpc1l2b-ps	N/A	N/A
10601298	Cdx4	N/A	N/A
10601303	Chic1	N/A	N/A
10601312	Chic1	N/A	N/A
10601314	Tsx	N/A	N/A
10601322	---	N/A	N/A
10601326	Uprt	N/A	N/A
10601328	Uprt	N/A	N/A

10601335	2610029G23Rik	N/A	N/A
10601343	Magee1	N/A	N/A
10601347	Cypt2-ps	N/A	N/A
10601350	Fgf16	N/A	N/A
10601354	---	N/A	N/A
10601356	Cox7b	N/A	N/A
10601360	Atp7a	N/A	N/A
10601385	Tlr13	N/A	N/A
10601390	Pgk1	N/A	N/A
10601404	Gm5127	N/A	N/A
10601412	Lpar4	N/A	N/A
10601416	P2ry10	N/A	N/A
10601424	Gpr174	N/A	N/A
10601430	4933401B06Rik	N/A	N/A
10601433	Tbx22	N/A	N/A
10601447	Fam46d	N/A	N/A
10601449	Sh3bgrl	N/A	N/A
10601456	Gm6377	N/A	N/A
10601459	Pou3f4	N/A	N/A
10601461	Cylc1	N/A	N/A
10601466	Tex16	N/A	N/A
10601473	Apool	N/A	N/A
10601484	2010106E10Rik	N/A	N/A
10601492	Zfp711	N/A	N/A
10601503	Dach2	N/A	N/A
10601519	Klhl4	N/A	N/A
10601531	Ube2dnl	N/A	N/A
10601534	4930524E20Rik	N/A	N/A
10601537	---	N/A	N/A
10601539	---	N/A	N/A
10601541	Cpxcr1	N/A	N/A
10601547	---	N/A	N/A
10601549	---	N/A	N/A
10601551	---	N/A	N/A
10601553	Gm14920	N/A	N/A
10601555	Tgif2lx1 /// Tgif2lx2	N/A	N/A
10601559	---	N/A	N/A
10601561	Pabpc5	N/A	N/A
10601567	Gapdh /// Gm12070 /// Gm2606 /// Gm4609 /// Gm2451 /// Gm10291	N/A	N/A
10601569	Pcdh11x	N/A	N/A
10601578	---	N/A	N/A
10601581	9230105E10Rik /// Trim5	N/A	N/A
10601583	Mthfd2l	N/A	N/A
10601585	---	N/A	N/A
10601588	3110007F17Rik /// Gm6604	N/A	N/A
10601593	Gm5167	N/A	N/A
10601595	3110007F17Rik /// Gm6604	N/A	N/A
10601598	3110007F17Rik /// Gm6604	N/A	N/A
10601601	Gm5167	N/A	N/A
10601603	Gm382	N/A	N/A
10601607	4921511C20Rik	N/A	N/A
10601610	---	N/A	N/A
10601612	Atrn	N/A	N/A
10601616	Diap2	N/A	N/A
10601648	Tnmd	N/A	N/A
10601659	Srpx2	N/A	N/A
10601673	Cstf2	N/A	N/A
10601691	Arl13a	N/A	N/A
10601701	Tmem35	N/A	N/A
10601729	Drp2	N/A	N/A
10601753	---	N/A	N/A
10601755	Rpl36a	N/A	N/A
10601760	Hnrnph2	N/A	N/A
10601764	---	N/A	N/A
10601768	Armcx4	N/A	N/A
10601771	Armcx1	N/A	N/A
10601778	Armcx3	N/A	N/A
10601786	Pramel	N/A	N/A
10601790	Pramel3 /// Gm5128 /// AV320801	N/A	N/A
10601797	Pramel3 /// Gm5128 /// AV320801	N/A	N/A
10601805	Pramel3 /// Gm5128 /// AV320801	N/A	N/A
10601813	Pramel3 /// Gm5128 /// AV320801	N/A	N/A
10601819	Gprasp1 /// Armcx5	N/A	N/A
10601834	Gprasp2 /// Gprasp1	N/A	N/A
10601844	Bhlhb9	N/A	N/A
10601846	2900062L11Rik	N/A	N/A
10601848	6530401D17Rik	N/A	N/A
10601850	Bex1 /// Bex4	N/A	N/A
10601854	Wbp5	N/A	N/A
10601857	Ngfrap1	N/A	N/A
10601861	---	N/A	N/A
10601863	---	N/A	N/A
10601865	---	N/A	N/A
10601867	Kir3dl1	N/A	N/A

10601874	Tceal6 /// Tceal5 /// Tceal3	N/A	N/A
10601878	Tceal1	N/A	N/A
10601880	BC065397 /// Morf4l2	N/A	N/A
10601882	BC065397	N/A	N/A
10601888	Plp1	N/A	N/A
10601901	1700014N06Rik	N/A	N/A
10601903	Zcchc18	N/A	N/A
10601915	Fam199x	N/A	N/A
10601922	---	N/A	N/A
10601925	---	N/A	N/A
10601927	Il1rapl2	N/A	N/A
10601942	Nrk	N/A	N/A
10601973	4930513O06Rik	N/A	N/A
10601978	4933428M09Rik	N/A	N/A
10601980	Mum1l1	N/A	N/A
10601993	D330045A20Rik	N/A	N/A
10602009	Rnf128	N/A	N/A
10602020	Tbc1d8b	N/A	N/A
10602033	Cldn2	N/A	N/A
10602038	E230019M04Rik	N/A	N/A
10602044	Frmpd3	N/A	N/A
10602062	Prps1	N/A	N/A
10602068	Mid2	N/A	N/A
10602081	Vsig1	N/A	N/A
10602090	Atg4a /// Syt6	N/A	N/A
10602105	Col4a5	N/A	N/A
10602164	Rbbp7	N/A	N/A
10602166	Nxt2	N/A	N/A
10602173	Hnrnpa1 /// Gm10052 /// Gm5643	N/A	N/A
10602176	---	N/A	N/A
10602178	---	N/A	N/A
10602180	Tmem164	N/A	N/A
10602192	Mir652	N/A	N/A
10602196	Rgag1	N/A	N/A
10602198	Pak3	N/A	N/A
10602221	Mir680-2	N/A	N/A
10602223	Alg13	N/A	N/A
10602251	Gm15070	N/A	N/A
10602253	---	N/A	N/A
10602255	Zcchc16	N/A	N/A
10602258	---	N/A	N/A
10602261	Htr2c	N/A	N/A
10602269	Mir448	N/A	N/A
10602271	---	N/A	N/A
10602277	Gm15107	N/A	N/A
10602284	Gm15107	N/A	N/A
10602293	Gm15107	N/A	N/A
10602300	Gm15107	N/A	N/A
10602307	Gm15107	N/A	N/A
10602327	Gm15107	N/A	N/A
10602332	Gm15107	N/A	N/A
10602341	Gm15107	N/A	N/A
10602350	Gm15107	N/A	N/A
10602359	Gm15107	N/A	N/A
10602367	Gm15107	N/A	N/A
10602372	Alas2	N/A	N/A
10602385	Pfkfb1	N/A	N/A
10602401	Fgd1 /// Tsr2	N/A	N/A
10602426	---	N/A	N/A
10602428	Wnk3-ps	N/A	N/A
10602454	Fam120c	N/A	N/A
10602474	Phf8	N/A	N/A
10602499	---	N/A	N/A
10602501	Huwe1	N/A	N/A
10602586	Mirlet7f-2	N/A	N/A
10602590	---	N/A	N/A
10602592	Hsd17b10	N/A	N/A
10602599	Smc1a	N/A	N/A
10602625	Iqsec2	N/A	N/A
10602644	Kdm5c	N/A	N/A
10602675	---	N/A	N/A
10602677	Gpr143	N/A	N/A
10602688	Usp51	N/A	N/A
10602690	Foxr2	N/A	N/A
10602692	Rragb	N/A	N/A
10602704	Klf8	N/A	N/A
10602716	Ubqln2	N/A	N/A
10602719	Cypt3	N/A	N/A
10602722	Spin2	N/A	N/A
10602725	4930542N07Rik	N/A	N/A
10602729	---	N/A	N/A
10602731	---	N/A	N/A
10602733	---	N/A	N/A
10602735	---	N/A	N/A

10602737	---	N/A	N/A
10602739	Acot9	N/A	N/A
10602744	Gm15155	N/A	N/A
10602749	4930503H13Rik	N/A	N/A
10602754	---	N/A	N/A
10602756	Smpx	N/A	N/A
10602765	Klhl34	N/A	N/A
10602770	---	N/A	N/A
10602772	Rps6ka3	N/A	N/A
10602795	Eif1ax	N/A	N/A
10602805	Mtap7d2	N/A	N/A
10602825	---	N/A	N/A
10602827	A830080D01Rik	N/A	N/A
10602840	Sh3kbp1	N/A	N/A
10602865	Pdha1 /// Map3k15	N/A	N/A
10602893	---	N/A	N/A
10602896	Gpr64	N/A	N/A
10602925	Phka2	N/A	N/A
10602964	---	N/A	N/A
10602975	---	N/A	N/A
10602977	Scml2	N/A	N/A
10603000	Rai2	N/A	N/A
10603003	---	N/A	N/A
10603005	---	N/A	N/A
10603009	Uba52 /// Gm5239 /// Gm7866	N/A	N/A
10603011	Rbbp7	N/A	N/A
10603023	---	N/A	N/A
10603026	Ctps2	N/A	N/A
10603051	Ap1s2	N/A	N/A
10603059	Tmem27	N/A	N/A
10603066	Ace2	N/A	N/A
10603087	Pir	N/A	N/A
10603099	Figf	N/A	N/A
10603109	Piga	N/A	N/A
10603116	Asb11	N/A	N/A
10603125	Asb9	N/A	N/A
10603135	Fancb	N/A	N/A
10603147	Gemin8	N/A	N/A
10603151	Gpm6b	N/A	N/A
10603166	Trappc2	N/A	N/A
10603172	Rpl7a	N/A	N/A
10603175	---	N/A	N/A
10603177	---	N/A	N/A
10603180	Chchd5	N/A	N/A
10603182	Arhgap6	N/A	N/A
10603206	BC022960	N/A	N/A
10603208	Mid1	N/A	N/A
10603230	4930408F14Rik /// Gm2790 /// Gm5923 /// Gm5926 /// Gm2940	N/A	N/A
10603242	4930408F14Rik /// Gm2790 /// Gm5923 /// Gm5926 /// Gm2940	N/A	N/A
10603245	---	N/A	N/A
10603247	---	N/A	N/A
10603249	Mycs	N/A	N/A
10603252	Larp4	N/A	N/A
10603254	Larp4	N/A	N/A
10603256	---	N/A	N/A
10603262	AU022751	N/A	N/A
10603266	Nudt10	N/A	N/A
10603270	Bmp15	N/A	N/A
10603275	Ccnb3	N/A	N/A
10603289	Clcn5	N/A	N/A
10603302	Mir500	N/A	N/A
10603304	Mir501	N/A	N/A
10603306	Mir362	N/A	N/A
10603308	Mir188	N/A	N/A
10603310	Mir532	N/A	N/A
10603312	Usp27x	N/A	N/A
10603316	2010204K13Rik	N/A	N/A
10603321	---	N/A	N/A
10603323	Ppp1r3f	N/A	N/A
10603328	Ccdc22 /// Foxp3	N/A	N/A
10603346	Plp2 /// Prickle3	N/A	N/A
10603354	Magix	N/A	N/A
10603362	Ccdc120	N/A	N/A
10603373	Pqbp1	N/A	N/A
10603387	Hdac6	N/A	N/A
10603417	Gata1	N/A	N/A
10603425	Glod5	N/A	N/A
10603431	Suv39h1	N/A	N/A
10603440	Was	N/A	N/A
10603453	Wdr13	N/A	N/A
10603485	Ebp	N/A	N/A
10603492	Porcn	N/A	N/A
10603508	Ftsj1	N/A	N/A
10603525	Ssxb2 /// Ssxb8	N/A	N/A

10603533	Ssxb3	N/A	N/A
10603538	Ssxb3 /// Ssxb2 /// Ssxb1 /// Ssxb9 /// Ssxb10 /// Ssx9	N/A	N/A
10603541	---	N/A	N/A
10603543	Fthl17 /// Gm5635	N/A	N/A
10603545	Fthl17 /// Gm5635	N/A	N/A
10603547	---	N/A	N/A
10603549	LOC280487	N/A	N/A
10603551	Cybb	N/A	N/A
10603567	Dynlt3	N/A	N/A
10603573	Sytl5	N/A	N/A
10603583	Srpx	N/A	N/A
10603598	Rpgr	N/A	N/A
10603618	---	N/A	N/A
10603620	Gm10489	N/A	N/A
10603623	---	N/A	N/A
10603625	---	N/A	N/A
10603627	Bcor	N/A	N/A
10603649	---	N/A	N/A
10603651	1810030O07Rik	N/A	N/A
10603659	Med14	N/A	N/A
10603698	Gm1549	N/A	N/A
10603700	---	N/A	N/A
10603702	Llph	N/A	N/A
10603704	---	N/A	N/A
10603706	Med4	N/A	N/A
10603708	Cask	N/A	N/A
10603736	Rnu2	N/A	N/A
10603738	4930403L05Rik	N/A	N/A
10603742	---	N/A	N/A
10603744	---	N/A	N/A
10603746	Maob	N/A	N/A
10603764	Ndp	N/A	N/A
10603768	Efhc2	N/A	N/A
10603785	Fundc1	N/A	N/A
10603791	Gm5073	N/A	N/A
10603794	---	N/A	N/A
10603796	4930578C19Rik	N/A	N/A
10603803	LOC280487	N/A	N/A
10603805	Mir221	N/A	N/A
10603807	Mir222	N/A	N/A
10603809	---	N/A	N/A
10603811	BC049702	N/A	N/A
10603814	Slc9a7	N/A	N/A
10603833	Usmg5	N/A	N/A
10603835	---	N/A	N/A
10603837	Ndufb11	N/A	N/A
10603841	---	N/A	N/A
10603843	Syn1	N/A	N/A
10603860	Cfp	N/A	N/A
10603870	Elk1	N/A	N/A
10603878	Uxt	N/A	N/A
10603881	Zfp182	N/A	N/A
10603887	Zfp300	N/A	N/A
10603894	---	N/A	N/A
10603896	Klhl13	N/A	N/A
10603909	---	N/A	N/A
10603911	---	N/A	N/A
10603962	---	N/A	N/A
10603973	---	N/A	N/A
10603984	---	N/A	N/A
10603995	---	N/A	N/A
10604006	---	N/A	N/A
10604017	---	N/A	N/A
10604019	1810037I17Rik	N/A	N/A
10604021	---	N/A	N/A
10604032	---	N/A	N/A
10604036	---	N/A	N/A
10604038	Akap17b	N/A	N/A
10604046	C330007P06Rik	N/A	N/A
10604053	Nkrf	N/A	N/A
10604057	Sep 06	N/A	N/A
10604072	Rpl39	N/A	N/A
10604076	Snora69	N/A	N/A
10604078	Upf3b	N/A	N/A
10604094	Akap14	N/A	N/A
10604100	Ndufa1	N/A	N/A
10604106	Gm9	N/A	N/A
10604111	Rhox1	N/A	N/A
10604119	Rhox4b	N/A	N/A
10604124	Rhox4b	N/A	N/A
10604129	Rhox3c /// Rhox3g-ps /// Rhox3f /// Rhox3a /// Rhox3b /// Rhox3h /// Rhox3e	N/A	N/A
10604143	Gm6310	N/A	N/A
10604148	Rhox8	N/A	N/A
10604154	Rhox9	N/A	N/A

10604157	4930525M21Rik	N/A	N/A
10604159	4930430D24Rik	N/A	N/A
10604165	Rhox11	N/A	N/A
10604169	Rhox12	N/A	N/A
10604175	Fam70a	N/A	N/A
10604187	Lamp2	N/A	N/A
10604199	Cul4b	N/A	N/A
10604226	C1galt1c1	N/A	N/A
10604230	Ap3s1	N/A	N/A
10604232	---	N/A	N/A
10604234	---	N/A	N/A
10604236	---	N/A	N/A
10604240	Rpl17 /// Gm10268 /// Rpl17-ps3	N/A	N/A
10604242	---	N/A	N/A
10604245	---	N/A	N/A
10604248	Thoc2	N/A	N/A
10604290	---	N/A	N/A
10604292	Odz1	N/A	N/A
10604327	Dcaf12l2	N/A	N/A
10604333	Dcaf12l1	N/A	N/A
10604337	---	N/A	N/A
10604340	---	N/A	N/A
10604342	Gm4987	N/A	N/A
10604344	---	N/A	N/A
10604347	Smarca1	N/A	N/A
10604375	Apln	N/A	N/A
10604380	Zdhhc9	N/A	N/A
10604393	Elf4	N/A	N/A
10604403	Elf4	N/A	N/A
10604405	Aifm1	N/A	N/A
10604424	Zfp280c	N/A	N/A
10604447	Gpr119	N/A	N/A
10604449	---	N/A	N/A
10604451	Enox2	N/A	N/A
10604470	Olfr1321	N/A	N/A
10604473	Igsf1	N/A	N/A
10604497	Olfr1322	N/A	N/A
10604499	Olfr1323	N/A	N/A
10604501	Hnrnpf	N/A	N/A
10604503	Olfr1324	N/A	N/A
10604505	6720401G13Rik	N/A	N/A
10604508	Frmd7	N/A	N/A
10604523	Rap2c	N/A	N/A
10604528	Mbnl3	N/A	N/A
10604542	Hs6st2	N/A	N/A
10604551	Usp26	N/A	N/A
10604557	1700080O16Rik	N/A	N/A
10604564	Gpc4	N/A	N/A
10604576	Gpc3	N/A	N/A
10604585	Mir717	N/A	N/A
10604587	Mir363	N/A	N/A
10604589	Mir92-2	N/A	N/A
10604593	Mir20b	N/A	N/A
10604597	Rps17 /// Gm6402 /// Gm12034	N/A	N/A
10604599	---	N/A	N/A
10604602	Mir450b	N/A	N/A
10604604	Mir450-1	N/A	N/A
10604606	Mir450-2	N/A	N/A
10604608	Mir542	N/A	N/A
10604610	Mir351	N/A	N/A
10604612	Mir503	N/A	N/A
10604614	Mir322	N/A	N/A
10604616	Plac1	N/A	N/A
10604620	Fam122b	N/A	N/A
10604630	Mospd1	N/A	N/A
10604633	Cxx1a /// Cxx1b /// Cxx1c	N/A	N/A
10604637	Cxx1a /// Cxx1b /// Cxx1c	N/A	N/A
10604641	---	N/A	N/A
10604643	1700013H16Rik	N/A	N/A
10604653	Zfp36l3	N/A	N/A
10604656	Xlr	N/A	N/A
10604661	---	N/A	N/A
10604665	---	N/A	N/A
10604669	---	N/A	N/A
10604671	---	N/A	N/A
10604674	Gm773	N/A	N/A
10604682	Gm648	N/A	N/A
10604687	Mmgt1	N/A	N/A
10604694	Mtap7d3	N/A	N/A
10604713	Arhgef6	N/A	N/A
10604735	Rbmx	N/A	N/A
10604743	Snord61	N/A	N/A
10604745	Gpr101	N/A	N/A
10604749	---	N/A	N/A

10604751	Fgf13	N/A	N/A
10604761	Mir504	N/A	N/A
10604763	Arpc1b	N/A	N/A
10604765	Mcf2	N/A	N/A
10604799	Atp11c	N/A	N/A
10604832	Mir505	N/A	N/A
10604834	---	N/A	N/A
10604837	Sox3	N/A	N/A
10604840	---	N/A	N/A
10604842	---	N/A	N/A
10604844	Sms /// Gm8234	N/A	N/A
10604846	---	N/A	N/A
10604850	---	N/A	N/A
10604853	Slitrk4	N/A	N/A
10604859	---	N/A	N/A
10604861	---	N/A	N/A
10604864	4930447F04Rik	N/A	N/A
10604867	Mir471	N/A	N/A
10604869	Mir463	N/A	N/A
10604871	Mir470	N/A	N/A
10604873	Mir465b-1 /// Mir465b-2 /// Mir465c-1 /// Mir465c-2	N/A	N/A
10604875	Mir465b-1 /// Mir465b-2 /// Mir465c-1 /// Mir465c-2	N/A	N/A
10604877	Mir465	N/A	N/A
10604879	---	N/A	N/A
10604882	---	N/A	N/A
10604885	4933436I01Rik	N/A	N/A
10604889	Mir201	N/A	N/A
10604891	Mir547	N/A	N/A
10604893	Gm4910	N/A	N/A
10604895	Gm10474	N/A	N/A
10604897	---	N/A	N/A
10604899	1700020N15Rik /// Gm6812	N/A	N/A
10604902	---	N/A	N/A
10604904	Rps6 /// Gm16409	N/A	N/A
10604906	Ids	N/A	N/A
10604918	4930567H17Rik	N/A	N/A
10604922	BC023829	N/A	N/A
10604930	---	N/A	N/A
10604932	Cd99l2	N/A	N/A
10604944	Gabre	N/A	N/A
10604954	Mir224	N/A	N/A
10604956	Mir452	N/A	N/A
10604958	Magea10	N/A	N/A
10604961	Gabra3	N/A	N/A
10604974	Cetn2	N/A	N/A
10604982	Pnma5	N/A	N/A
10604984	Xlr4a /// Xlr4b /// Xlr4c	N/A	N/A
10604996	Xlr3c	N/A	N/A
10605007	Xlr3c	N/A	N/A
10605018	Xlr5c /// Xlr5a /// Xlr5b	N/A	N/A
10605028	Gm14685 /// Gm5640 /// DXBay18	N/A	N/A
10605034	Xlr4a /// Xlr4b /// Xlr4c	N/A	N/A
10605044	Xlr5c /// Xlr5a /// Xlr5b	N/A	N/A
10605051	Trex2	N/A	N/A
10605055	Haus7	N/A	N/A
10605067	Pnck	N/A	N/A
10605081	Bcap31	N/A	N/A
10605090	Idh3g	N/A	N/A
10605104	Pdzd4	N/A	N/A
10605113	L1cam	N/A	N/A
10605143	Arhgap4	N/A	N/A
10605172	Arhgap4	N/A	N/A
10605181	Renbp	N/A	N/A
10605195	Hcfc1	N/A	N/A
10605222	Irak1 /// Mir718	N/A	N/A
10605247	Mecp2	N/A	N/A
10605252	Tex28	N/A	N/A
10605256	Flna	N/A	N/A
10605303	Dnase1l1	N/A	N/A
10605315	Lage3	N/A	N/A
10605319	Slc10a3-ubl4 /// Ubl4	N/A	N/A
10605324	Slc10a3-ubl4 /// Slc10a3	N/A	N/A
10605328	Fam3a	N/A	N/A
10605338	G6pdx	N/A	N/A
10605349	Ube2d3	N/A	N/A
10605351	---	N/A	N/A
10605353	4930408F14Rik /// Gm2790 /// Gm5923 /// Gm5926 /// Gm2940	N/A	N/A
10605355	---	N/A	N/A
10605357	Gab3	N/A	N/A
10605370	Mpp1	N/A	N/A
10605386	4930428E23Rik	N/A	N/A
10605392	F8	N/A	N/A
10605421	Mtcp1	N/A	N/A
10605429	---	N/A	N/A

10605431	Rab39b	N/A	N/A
10605455	---	N/A	N/A
10605457	Gm5793	N/A	N/A
10605459	Gm6927	N/A	N/A
10605461	---	N/A	N/A
10605465	Prkx	N/A	N/A
10605475	Pbsn	N/A	N/A
10605482	Gm14744	N/A	N/A
10605488	Obp1a	N/A	N/A
10605493	Prrg1	N/A	N/A
10605499	3426406K10Rik	N/A	N/A
10605501	---	N/A	N/A
10605503	---	N/A	N/A
10605505	---	N/A	N/A
10605507	Zc3h18	N/A	N/A
10605510	---	N/A	N/A
10605512	---	N/A	N/A
10605522	Gm7173	N/A	N/A
10605542	Mageb16 /// Mageb16-ps1	N/A	N/A
10605552	---	N/A	N/A
10605554	Gapdh	N/A	N/A
10605559	---	N/A	N/A
10605561	4930595M18Rik	N/A	N/A
10605566	---	N/A	N/A
10605568	Tsga8	N/A	N/A
10605571	Gyk	N/A	N/A
10605598	Mageb4	N/A	N/A
10605603	Mageb4	N/A	N/A
10605606	Gm41	N/A	N/A
10605612	---	N/A	N/A
10605614	---	N/A	N/A
10605616	Il1rapl1	N/A	N/A
10605629	---	N/A	N/A
10605631	---	N/A	N/A
10605633	Pet2	N/A	N/A
10605644	---	N/A	N/A
10605648	4930415L06Rik	N/A	N/A
10605651	---	N/A	N/A
10605654	Mageb5	N/A	N/A
10605659	---	N/A	N/A
10605662	Gm5072	N/A	N/A
10605666	Mageb18	N/A	N/A
10605674	Pola1	N/A	N/A
10605711	Pdk3	N/A	N/A
10605726	---	N/A	N/A
10605729	Zfx	N/A	N/A
10605740	Eif2s3x	N/A	N/A
10605753	4932442L08Rik	N/A	N/A
10605766	Maged1	N/A	N/A
10605782	---	N/A	N/A
10605784	Gm7061	N/A	N/A
10605786	Gm7061	N/A	N/A
10605788	Zxda /// Zxdb	N/A	N/A
10605790	---	N/A	N/A
10605792	Spin4	N/A	N/A
10605797	Arhgef9	N/A	N/A
10605811	Fam123b	N/A	N/A
10605815	Asb12	N/A	N/A
10605820	Zc4h2	N/A	N/A
10605828	1700010D01Rik	N/A	N/A
10605831	Las1l	N/A	N/A
10605848	Vsig4	N/A	N/A
10605855	Hsf3	N/A	N/A
10605871	Gm9059	N/A	N/A
10605874	Eda2r	N/A	N/A
10605884	Ophn1	N/A	N/A
10605914	---	N/A	N/A
10605917	Gm14812	N/A	N/A
10605919	Pja1	N/A	N/A
10605929	Awat2	N/A	N/A
10605938	P2ry4	N/A	N/A
10605943	Pdzd11 /// Kif4	N/A	N/A
10605952	Gdpd2	N/A	N/A
10605954	Tex11	N/A	N/A
10605986	Slc7a3	N/A	N/A
10605999	---	N/A	N/A
10606001	Snx12	N/A	N/A
10606009	Gm614	N/A	N/A
10606016	Il2rg	N/A	N/A
10606026	Zmym3	N/A	N/A
10606056	2610030H06Rik	N/A	N/A
10606058	Cxcr3	N/A	N/A
10606060	---	N/A	N/A
10606064	Rgag4	N/A	N/A

10606069	4930444G20Rik /// AF366264 /// Gm4831 /// Gm9839	N/A	N/A
10606071	Ercc6l	N/A	N/A
10606075	Rps4x	N/A	N/A
10606083	Cited1	N/A	N/A
10606088	Hdac8	N/A	N/A
10606102	Phka1	N/A	N/A
10606138	Dmrtc1c /// Dmrtc1c2	N/A	N/A
10606146	1700031F05Rik	N/A	N/A
10606149	Dmrtc1a	N/A	N/A
10606158	Pabpc1l2b-ps	N/A	N/A
10606160	Rfwd2	N/A	N/A
10606163	4930519F16Rik	N/A	N/A
10606174	Nap1l2	N/A	N/A
10606178	Xist	N/A	N/A
10606180	Gm9785	N/A	N/A
10606182	Mir421	N/A	N/A
10606184	Mir374	N/A	N/A
10606186	Slc16a2	N/A	N/A
10606195	Rlim	N/A	N/A
10606206	C77370	N/A	N/A
10606215	Mir672	N/A	N/A
10606217	Abcb7	N/A	N/A
10606235	Zdhhc15	N/A	N/A
10606248	Magee2	N/A	N/A
10606252	---	N/A	N/A
10606255	Rpl30	N/A	N/A
10606259	Mir325	N/A	N/A
10606261	Rpl12 /// Rpl12-ps1	N/A	N/A
10606263	Atrx	N/A	N/A
10606301	Magt1	N/A	N/A
10606315	Taf9b	N/A	N/A
10606323	Fnd3c2	N/A	N/A
10606355	Cysltr1	N/A	N/A
10606362	---	N/A	N/A
10606364	---	N/A	N/A
10606366	Zcchc5	N/A	N/A
10606369	Itm2a	N/A	N/A
10606376	2610002M06Rik	N/A	N/A
10606386	Gm732	N/A	N/A
10606389	---	N/A	N/A
10606391	Gm10452	N/A	N/A
10606393	Brwd3	N/A	N/A
10606436	Hmgn5	N/A	N/A
10606439	---	N/A	N/A
10606441	---	N/A	N/A
10606443	---	N/A	N/A
10606445	Rps6ka6	N/A	N/A
10606473	Gm9927 /// Hdx	N/A	N/A
10606475	Hdx	N/A	N/A
10606488	Satl1	N/A	N/A
10606495	Pof1b	N/A	N/A
10606513	Chm	N/A	N/A
10606530	Mir361	N/A	N/A
10606535	---	N/A	N/A
10606538	Rpl30 /// Gm12191 /// Gm6570 /// Gm6109	N/A	N/A
10606540	---	N/A	N/A
10606542	---	N/A	N/A
10606544	---	N/A	N/A
10606548	Tgif2lx1 /// Tgif2lx2	N/A	N/A
10606552	Gm14920	N/A	N/A
10606554	Nap1l3	N/A	N/A
10606559	Vmn2r121	N/A	N/A
10606565	Gm10063	N/A	N/A
10606567	Rps12 /// Gm10063 /// Rps12-ps2	N/A	N/A
10606569	Rps12 /// Gm10063 /// Rps12-ps2	N/A	N/A
10606571	Gm10063	N/A	N/A
10606575	Gm10063	N/A	N/A
10606577	Rps12 /// Gm10063 /// Rps12-ps2	N/A	N/A
10606579	Rps12 /// Gm10063 /// Rps12-ps2	N/A	N/A
10606581	Rps12 /// Gm10063 /// Rps12-ps2	N/A	N/A
10606583	4932411N23Rik	N/A	N/A
10606589	4930412D23Rik	N/A	N/A
10606592	---	N/A	N/A
10606595	---	N/A	N/A
10606600	Pcdh19	N/A	N/A
10606609	Tspan6	N/A	N/A
10606619	Sytl4	N/A	N/A
10606640	Nox1	N/A	N/A
10606654	Xkrx	N/A	N/A
10606658	Trmt2b	N/A	N/A
10606674	Taf7l	N/A	N/A
10606689	Timm8a1	N/A	N/A
10606694	Btk	N/A	N/A
10606714	Gla	N/A	N/A

10606725	---	N/A	N/A
10606728	Gm10344	N/A	N/A
10606730	Armcx6	N/A	N/A
10606735	Armcx2	N/A	N/A
10606744	Rps2 /// Rps2-ps6	N/A	N/A
10606746	Nxf2	N/A	N/A
10606770	Zmat1	N/A	N/A
10606783	Pramel3 /// Gm5128 /// AV320801	N/A	N/A
10606789	Tceal6	N/A	N/A
10606792	Nxf7	N/A	N/A
10606816	Prame	N/A	N/A
10606820	1700008I05Rik	N/A	N/A
10606831	Tmsb15a	N/A	N/A
10606835	Bex2	N/A	N/A
10606837	Nxf3	N/A	N/A
10606858	Tceal8	N/A	N/A
10606864	Tceal5	N/A	N/A
10606868	Bex1	N/A	N/A
10606870	Kir3dl2	N/A	N/A
10606876	Morf4l2	N/A	N/A
10606880	Glra4	N/A	N/A
10606893	Rab9b	N/A	N/A
10606902	Tmsb15l /// Tmsb15b2 /// Tmsb15b1	N/A	N/A
10606910	Mcart6	N/A	N/A
10606912	---	N/A	N/A
10606914	Esx1	N/A	N/A
10606924	---	N/A	N/A
10606926	Tex13a	N/A	N/A
10606928	Serpina7	N/A	N/A
10606936	---	N/A	N/A
10606939	Rps4x	N/A	N/A
10606941	Ripply1	N/A	N/A
10606948	Morc4	N/A	N/A
10606969	Rbm41	N/A	N/A
10606979	Nup62cl	N/A	N/A
10606989	Tsc22d3	N/A	N/A
10606997	Tex13	N/A	N/A
10607002	---	N/A	N/A
10607004	Psmd10	N/A	N/A
10607012	Col4a6	N/A	N/A
10607059	Irs4	N/A	N/A
10607064	Gucy2f	N/A	N/A
10607085	Kcne1l	N/A	N/A
10607089	Acsl4	N/A	N/A
10607111	---	N/A	N/A
10607113	Rgs3	N/A	N/A
10607116	Ammecr1	N/A	N/A
10607124	Chrdl1	N/A	N/A
10607143	Capn6	N/A	N/A
10607156	Dcx	N/A	N/A
10607169	Trpc5	N/A	N/A
10607183	Lhfpl1	N/A	N/A
10607189	Amot	N/A	N/A
10607202	---	N/A	N/A
10607204	Gm8261	N/A	N/A
10607206	Il13ra2	N/A	N/A
10607222	Vmn1r41 /// Vmn1r239-ps	N/A	N/A
10607225	Lrch2	N/A	N/A
10607244	---	N/A	N/A
10607246	Tmem29	N/A	N/A
10607250	Apex2	N/A	N/A
10607259	Tro	N/A	N/A
10607280	---	N/A	N/A
10607283	Maged2 /// LOC675577	N/A	N/A
10607302	Gnl3l	N/A	N/A
10607317	Tsr2	N/A	N/A
10607324	Rpl21	N/A	N/A
10607328	---	N/A	N/A
10607330	Fam120c /// A230072E10Rik	N/A	N/A
10607332	---	N/A	N/A
10607334	---	N/A	N/A
10607336	Ribc1	N/A	N/A
10607346	Tspyl2	N/A	N/A
10607356	Gpr173	N/A	N/A
10607361	---	N/A	N/A
10607363	---	N/A	N/A
10607366	Shroom2	N/A	N/A
10607388	Rpl7a /// Gm16477	N/A	N/A
10607391	Rps7 /// Gm9493	N/A	N/A
10607393	---	N/A	N/A
10607395	Mageh1	N/A	N/A
10607398	---	N/A	N/A
10607401	---	N/A	N/A
10607403	Kctd12b	N/A	N/A

10607407	---	N/A	N/A
10607409	Gm15144 /// Gm5646 /// Gm15140	N/A	N/A
10607412	Gm15144 /// Gm5646 /// Gm15140	N/A	N/A
10607415	Gm15144 /// Gm15140	N/A	N/A
10607419	---	N/A	N/A
10607421	4930524N10Rik	N/A	N/A
10607425	Gm5647 /// 4930524N10Rik	N/A	N/A
10607429	LOC280487	N/A	N/A
10607431	Magea6	N/A	N/A
10607433	Magea3	N/A	N/A
10607437	Magea3 /// Magea8	N/A	N/A
10607443	Magea2 /// Magea6 /// Magea5 /// Magea1 /// Magea3 /// Magea8	N/A	N/A
10607450	Magea5	N/A	N/A
10607454	Magea2 /// Magea6 /// Magea5 /// Magea1 /// Magea3 /// Magea8	N/A	N/A
10607459	1700042B14Rik	N/A	N/A
10607465	Gm4997	N/A	N/A
10607467	Sat1	N/A	N/A
10607473	---	N/A	N/A
10607475	Prdx4	N/A	N/A
10607484	Ptchd1	N/A	N/A
10607486	Ptchd1	N/A	N/A
10607493	---	N/A	N/A
10607495	---	N/A	N/A
10607497	Suclg2	N/A	N/A
10607499	Phex	N/A	N/A
10607524	Sms /// Gm8234	N/A	N/A
10607539	Mbtps2 /// Yy2	N/A	N/A
10607557	---	N/A	N/A
10607560	---	N/A	N/A
10607562	Cnksr2	N/A	N/A
10607585	---	N/A	N/A
10607587	Pdha1 /// Map3k15	N/A	N/A
10607600	---	N/A	N/A
10607602	Ppef1	N/A	N/A
10607619	Cdkl5	N/A	N/A
10607639	Gja6	N/A	N/A
10607643	---	N/A	N/A
10607646	Nhs	N/A	N/A
10607658	Reps2	N/A	N/A
10607679	Txlng	N/A	N/A
10607694	Syap1	N/A	N/A
10607705	S100g	N/A	N/A
10607710	---	N/A	N/A
10607712	Grpr	N/A	N/A
10607718	1700045I19Rik	N/A	N/A
10607724	Zrsr2	N/A	N/A
10607738	Car5b	N/A	N/A
10607747	Siah1b	N/A	N/A
10607752	Bmx	N/A	N/A
10607774	Mospd2	N/A	N/A
10607792	Glra2	N/A	N/A
10607804	---	N/A	N/A
10607806	Ofd1	N/A	N/A
10607838	---	N/A	N/A
10607841	Tceanc	N/A	N/A
10607848	Egfl6	N/A	N/A
10607862	---	N/A	N/A
10607865	Tmsb4x	N/A	N/A
10607868	Tlr8	N/A	N/A
10607870	Tlr7	N/A	N/A
10607877	Prps2	N/A	N/A
10607886	Gm6744	N/A	N/A
10607888	Frmpd4	N/A	N/A
10607910	Msl3	N/A	N/A
10607924	Amelx	N/A	N/A
10607933	Hccs	N/A	N/A
10607943	---	N/A	N/A
10607945	4933400A11Rik	N/A	N/A
10607950	G530011O06Rik	N/A	N/A
10607952	Vamp7	N/A	N/A
10607962	Ube1y1	N/A	N/A
10607972	Kdm5d /// LOC100040412	N/A	N/A
10608001	Eif2s3y	N/A	N/A
10608016	---	N/A	N/A
10608018	---	N/A	N/A
10608024	---	N/A	N/A
10608033	Gm6026 /// Gm16501	N/A	N/A
10608035	Ube1y1	N/A	N/A
10608041	Rbmy1a1	N/A	N/A
10608062	Rbmy1a1	N/A	N/A
10608073	Rbmy1a1	N/A	N/A
10608083	---	N/A	N/A
10608085	---	N/A	N/A
10608087	---	N/A	N/A

10608089	Ube1y1	N/A	N/A
10608100	Zfy1	N/A	N/A
10608107	Uty	N/A	N/A
10608136	---	N/A	N/A
10608138	Ddx3y	N/A	N/A
10608156	Usp9y	N/A	N/A
10608182	---	N/A	N/A
10608184	Zfy2	N/A	N/A
10608187	Ube1y1	N/A	N/A
10608193	Sry	N/A	N/A
10608196	Gm6026 /// Gm16501	N/A	N/A
10608198	---	N/A	N/A
10608200	---	N/A	N/A
10608202	---	N/A	N/A
10608204	---	N/A	N/A
10608212	Sly /// LOC100041897	N/A	N/A
10608237	Sly /// LOC100042331 /// LOC380994	N/A	N/A
10608251	LOC380994	N/A	N/A
10608260	Srsy	N/A	N/A
10608263	Sly /// LOC100041897	N/A	N/A
10608280	---	N/A	N/A
10608286	---	N/A	N/A
10608288	---	N/A	N/A
10608290	---	N/A	N/A
10608298	---	N/A	N/A
10608300	---	N/A	N/A
10608304	---	N/A	N/A
10608311	---	N/A	N/A
10608325	---	N/A	N/A
10608330	Sly /// LOC380994 /// LOC100041897	N/A	N/A
10608352	LOC380994 /// LOC100042331	N/A	N/A
10608385	Sly	N/A	N/A
10608397	Sly /// LOC100042331 /// LOC380994 /// LOC100041897	N/A	N/A
10608407	Srsy	N/A	N/A
10608410	Sly /// LOC100041897	N/A	N/A
10608422	Rmi1 /// LOC665550	N/A	N/A
10608429	LOC380994	N/A	N/A
10608438	---	N/A	N/A
10608442	LOC380994	N/A	N/A
10608464	LOC100042331 /// LOC380994	N/A	N/A
10608492	LOC380994	N/A	N/A
10608499	---	N/A	N/A
10608504	Rbm31y	N/A	N/A
10608509	---	N/A	N/A
10608523	LOC380994	N/A	N/A
10608533	---	N/A	N/A
10608535	---	N/A	N/A
10608539	---	N/A	N/A
10608557	Sly	N/A	N/A
10608579	---	N/A	N/A
10608586	LOC380994 /// Sly /// LOC100042331 /// LOC100041897	N/A	N/A
10608600	---	N/A	N/A
10608615	LOC100042331 /// LOC380994	N/A	N/A
Liver Class V			
Probe ID	**Gene ID**	**Con Peak**	**TSR Peak**
10344931	Rpl5	N/A	N/A
10345423	Plekhb2	N/A	N/A
10345504	Cox5b	N/A	N/A
10345620	Mrpl30	N/A	N/A
10345698	Tbc1d8 /// Rpl31 /// Gm16382	N/A	N/A
10346105	Rpl23a /// Rpl23a-ps1	N/A	N/A
10346328	---	N/A	N/A
10346410	Aox3	N/A	N/A
10346533	Nif3l1 /// Ppil3	N/A	N/A
10346808	Rpl17	N/A	N/A
10346867	Eef1b2	N/A	N/A
10346874	---	N/A	N/A
10347218	---	N/A	N/A
10347273	Rpl37a /// Mir682	N/A	N/A
10347873	Agfg1	N/A	N/A
10347917	Rpl19	N/A	N/A
10348775	Ppp1r7	N/A	N/A
10348924	Rpl18a /// Gm15427	N/A	N/A
10349694	Pm20d1	N/A	N/A
10350614	1190005F20Rik	N/A	N/A
10350816	Rpl35 /// Gm10269	N/A	N/A
10350948	---	N/A	N/A
10350990	Rpl23a /// Gm6177	N/A	N/A
10350992	Rc3h1	N/A	N/A
10351043	Snord47	N/A	N/A
10351504	---	N/A	N/A
10351546	Apoa2	N/A	N/A
10352092	Zfp238	N/A	N/A

10352348	Cnih4	N/A	N/A
10352396	Trp53bp2	N/A	N/A
10352503	Bpnt1	N/A	N/A
10352756	Lpgat1	N/A	N/A
10353061	---	N/A	N/A
10353064	Arfgef1	N/A	N/A
10353265	Rpl7 /// Gm5045	N/A	N/A
10353341	Hmgb1	N/A	N/A
10353545	1110058L19Rik	N/A	N/A
10353689	Phf3	N/A	N/A
10353729	Gm5415 /// 4930444G20Rik /// Gm9839	N/A	N/A
10353731	Gm5415 /// 4930444G20Rik /// Gm9839	N/A	N/A
10353947	Tmem131	N/A	N/A
10353991	Rpl12 /// Rpl12-ps1	N/A	N/A
10354372	Myl6 /// Gm8894	N/A	N/A
10354470	Rpl15 /// Gm4294	N/A	N/A
10354739	Atp5l	N/A	N/A
10354768	Akr1b3 /// Gm6644	N/A	N/A
10355844	Dnpep	N/A	N/A
10356194	Trip12	N/A	N/A
10356482	---	N/A	N/A
10356778	Rpl17	N/A	N/A
10357124	Tsn	N/A	N/A
10357292	Ddx18	N/A	N/A
10357478	Rpl31 /// Gm16382	N/A	N/A
10357514	---	N/A	N/A
10358609	Hmcn1	N/A	N/A
10358709	Cox7b	N/A	N/A
10359422	Prdx6	N/A	N/A
10359961	Uhmk1	N/A	N/A
10360128	Rpl27	N/A	N/A
10360460	Chml	N/A	N/A
10360522	Adss	N/A	N/A
10360679	H3f3a /// Gm12657	N/A	N/A
10360840	Mosc1	N/A	N/A
10361065	Mfsd7b	N/A	N/A
10361640	---	N/A	N/A
10361816	Rpl23a /// Gm3940 /// Gm10335	N/A	N/A
10362273	Tmem200a /// Gm9767	N/A	N/A
10362892	Pam16	N/A	N/A
10363000	Gpx4	N/A	N/A
10363368	Timm23	N/A	N/A
10363622	3110049J23Rik	N/A	N/A
10363699	Rps6 /// Gm16409	N/A	N/A
10364038	Upb1	N/A	N/A
10364102	Chchd10	N/A	N/A
10364293	Ube2g2	N/A	N/A
10364373	Lsm7	N/A	N/A
10364455	Cdc34	N/A	N/A
10364696	Atp5d	N/A	N/A
10364744	Ndufs7	N/A	N/A
10365337	---	N/A	N/A
10365601	Gnptab	N/A	N/A
10365891	Tmcc3	N/A	N/A
10365983	Lum	N/A	N/A
10366004	Atp2b1	N/A	N/A
10366229	Lin7a	N/A	N/A
10366546	Cpm	N/A	N/A
10366593	---	N/A	N/A
10367059	BC089597	N/A	N/A
10367091	Naca	N/A	N/A
10367106	Atp5b	N/A	N/A
10367215	Apon	N/A	N/A
10368220	---	N/A	N/A
10368343	Arg1	N/A	N/A
10368504	Rpl12	N/A	N/A
10368720	Slc16a10	N/A	N/A
10368886	Foxo3	N/A	N/A
10369154	Man1a	N/A	N/A
10369206	Rps8 /// Gm11353 /// Rps8-ps1	N/A	N/A
10369210	Serinc1	N/A	N/A
10369295	Anapc16	N/A	N/A
10369774	Nrbf2	N/A	N/A
10369877	Ube2d1	N/A	N/A
10369989	Ddt	N/A	N/A
10370777	2310011J03Rik	N/A	N/A
10371002	Lsm7	N/A	N/A
10371037	Slc39a3	N/A	N/A
10371265	Rpl23a /// Gm3940 /// Gm6177 /// Gm8290	N/A	N/A
10371267	Rpl23a /// Gm3940 /// Gm6177 /// Gm8290	N/A	N/A
10371269	Rpl23a /// Gm3940 /// Gm6177 /// Gm8290	N/A	N/A
10371275	Rpl23a /// Gm3940 /// Gm6177 /// Gm8290	N/A	N/A
10371293	1190007I07Rik	N/A	N/A
10371307	Nfyb	N/A	N/A

10371811	Scyl2	N/A	N/A
10371842	Uhrf1bp1l	N/A	N/A
10372114	Gm8671	N/A	N/A
10373027	Tspan31 /// Cdk4	N/A	N/A
10373396	Myl6	N/A	N/A
10373448	Rpl41	N/A	N/A
10373498	Rps26	N/A	N/A
10373519	Rpl12	N/A	N/A
10373651	---	N/A	N/A
10374175	Ppia	N/A	N/A
10374476	Rps17 /// Gm12034	N/A	N/A
10374840	---	N/A	N/A
10375229	---	N/A	N/A
10375461	Rpl7 /// Gm5045	N/A	N/A
10375926	Ppp2ca	N/A	N/A
10376314	Cnot8	N/A	N/A
10376461	Trim11	N/A	N/A
10376885	Snord49b	N/A	N/A
10377429	Snord118	N/A	N/A
10377541	Lsmd1 /// Tmem88	N/A	N/A
10377689	Gabarap	N/A	N/A
10378126	Ankfy1	N/A	N/A
10378637	Scarf1	N/A	N/A
10379736	1100001G20Rik	N/A	N/A
10379875	Rpl13	N/A	N/A
10380670	Rpl17 /// Gm10268 /// Rpl17-ps3	N/A	N/A
10380815	Psmb3	N/A	N/A
10380821	Atp5l	N/A	N/A
10380833	Rpl19	N/A	N/A
10381187	Atp6v0a1	N/A	N/A
10381381	---	N/A	N/A
10381402	Rpl27	N/A	N/A
10381458	Rnu2	N/A	N/A
10381460	Rnu2	N/A	N/A
10381470	Rnu2	N/A	N/A
10381472	Rnu2	N/A	N/A
10381939	Tanc2	N/A	N/A
10382081	---	N/A	N/A
10382104	Snord104	N/A	N/A
10382189	Apoh	N/A	N/A
10382369	Rpl38	N/A	N/A
10382470	Tmem104	N/A	N/A
10383850	---	N/A	N/A
10383887	Uqcr10	N/A	N/A
10384100	Rps15a /// Gm11968	N/A	N/A
10384552	Gapdh /// Gm2606 /// Gm2451 /// Gm10293	N/A	N/A
10384577	---	N/A	N/A
10384603	Mdh1	N/A	N/A
10385034	Rpsa	N/A	N/A
10385043	Npm1	N/A	N/A
10385426	Hmgb1	N/A	N/A
10385557	Cnot6	N/A	N/A
10385570	Rpl30 /// Gm12191 /// Gm6570 /// Gm6109	N/A	N/A
10385716	0610009B22Rik	N/A	N/A
10385719	Sec24a	N/A	N/A
10385790	Hspa4	N/A	N/A
10385818	Uqcrq	N/A	N/A
10385926	Cdc42se2	N/A	N/A
10386005	Atp5f1	N/A	N/A
10386187	Rpl24	N/A	N/A
10386442	Cops3	N/A	N/A
10386527	---	N/A	N/A
10386529	---	N/A	N/A
10386533	---	N/A	N/A
10386535	---	N/A	N/A
10386537	---	N/A	N/A
10386541	---	N/A	N/A
10386546	---	N/A	N/A
10386949	Hmgb1	N/A	N/A
10387559	Senp3	N/A	N/A
10387983	---	N/A	N/A
10388225	---	N/A	N/A
10388461	Cox7c /// Gm10012	N/A	N/A
10388520	Glod4	N/A	N/A
10388579	Gosr1	N/A	N/A
10388591	Cpd	N/A	N/A
10388682	Taok1	N/A	N/A
10388776	Rpl23a /// Gm3940 /// Gm10335	N/A	N/A
10388884	Nlk	N/A	N/A
10389062	1700071K01Rik	N/A	N/A
10389162	Rpl12	N/A	N/A
10389331	Znhit3 /// Myo19	N/A	N/A
10389339	Usp32	N/A	N/A
10389373	Appbp2	N/A	N/A

10389858	Dscaml1 /// Nme2	N/A	N/A
10389875	Rpl27	N/A	N/A
10390237	Atp5g1	N/A	N/A
10390283	Cdk5rap3	N/A	N/A
10390381	Npepps	N/A	N/A
10390595	Med1	N/A	N/A
10391488	Rnu2	N/A	N/A
10391963	Nsf	N/A	N/A
10392881	Fdxr	N/A	N/A
10392930	Atp5h /// Gm4953 /// Gm5048	N/A	N/A
10393823	P4hb	N/A	N/A
10393926	Dcxr	N/A	N/A
10394142	Hmgb1	N/A	N/A
10394353	0610009D07Rik	N/A	N/A
10394532	Ube2f /// Gm5434	N/A	N/A
10394558	Rpl29 /// Gm3550	N/A	N/A
10394591	Rpl15 /// Gm4294	N/A	N/A
10394776	---	N/A	N/A
10394778	Hpcal1	N/A	N/A
10395005	Kidins220	N/A	N/A
10395250	---	N/A	N/A
10395312	Twistnb	N/A	N/A
10395737	Rpl21 /// Gm7555	N/A	N/A
10395925	Mia2	N/A	N/A
10396030	Fancm	N/A	N/A
10396059	Rpl17 /// Gm10268 /// Rpl17-ps3	N/A	N/A
10396193	Psma3	N/A	N/A
10396278	Daam1	N/A	N/A
10396421	Hif1a	N/A	N/A
10396694	Churc1	N/A	N/A
10396896	Slc39a9	N/A	N/A
10396919	4933426M11Rik	N/A	N/A
10397094	Psen1	N/A	N/A
10397699	Rpl30 /// Gm12191 /// Gm6570 /// Gm5481 /// Gm6109	N/A	N/A
10397741	Psmc1	N/A	N/A
10398147	Papola	N/A	N/A
10398173	Vrk1	N/A	N/A
10398368	---	N/A	N/A
10398451	Rps25 /// Gm4963	N/A	N/A
10398599	Rps19 /// Rps19-ps3 /// Gm6636	N/A	N/A
10398678	Eif5	N/A	N/A
10398693	Snora28	N/A	N/A
10399428	Snord118	N/A	N/A
10399760	Rps7 /// Gm9493	N/A	N/A
10399823	---	N/A	N/A
10400153	Hmgb1	N/A	N/A
10400210	Hectd1	N/A	N/A
10400334	Rpl31 /// Gm16382	N/A	N/A
10400470	Cox6c	N/A	N/A
10400515	Sec23a	N/A	N/A
10400570	---	N/A	N/A
10400581	Fkbp3 /// Prpf39	N/A	N/A
10400984	Tmem30b	N/A	N/A
10401023	Wdr89 /// Rplp2	N/A	N/A
10401172	Vti1b /// Arg2	N/A	N/A
10401924	Rpl31 /// Rpl31-ps1 /// Gm16382	N/A	N/A
10401958	Naca	N/A	N/A
10401997	Ptpn21	N/A	N/A
10402394	Serpina1d	N/A	N/A
10402648	Brp44l	N/A	N/A
10402750	Hmgb1	N/A	N/A
10402835	Nudt14	N/A	N/A
10403052	---	N/A	N/A
10403253	Rps4x	N/A	N/A
10403322	Akr1c6	N/A	N/A
10403394	---	N/A	N/A
10403706	Psma2	N/A	N/A
10403750	2810021B07Rik	N/A	N/A
10403911	Gpx6	N/A	N/A
10403957	Hist1h4m /// Hist1h4b /// Hist1h4f /// Hist1h4a /// Hist1h4i /// Gm11275	N/A	N/A
10404067	Hist1h4m /// Hist1h4c /// Hist1h4a /// Hist1h4b /// Hist1h4f /// Gm11275	N/A	N/A
10404077	Slc17a2	N/A	N/A
10404262	Rps8 /// Gm11353 /// Rps8-ps1	N/A	N/A
10404354	Rps18 /// Gm10260	N/A	N/A
10404731	Tmem14c	N/A	N/A
10404928	C78339	N/A	N/A
10404996	Ninj1	N/A	N/A
10405462	BC038268 /// Pfn3	N/A	N/A
10405535	B230219D22Rik	N/A	N/A
10405539	Txndc15	N/A	N/A
10405587	Tgfbi	N/A	N/A
10405626	Rpl17 /// Gm10268 /// Rpl17-ps3	N/A	N/A
10405729	Selk	N/A	N/A
10405853	Zfp369	N/A	N/A

10406024	Rps28	N/A	N/A
10406250	Rpl37a /// Mir682	N/A	N/A
10406777	Gcnt4	N/A	N/A
10407120	Rps3a /// Gm9000	N/A	N/A
10407319	Rpl34 /// Gm6404 /// Gm10154	N/A	N/A
10407346	---	N/A	N/A
10407507	Rpl35a /// Ino80 /// Gm10247	N/A	N/A
10407570	Zmynd11	N/A	N/A
10407907	Rala	N/A	N/A
10408074	Hist1h4m /// Hist1h4k /// Gm11275 /// Hist1h4j /// Hist1h4a /// Hist1h4b	N/A	N/A
10408092	Hist1h4i /// Hist1h4m /// Hist1h4f /// Hist1h4a /// Hist1h4b /// Gm11275	N/A	N/A
10408113	Hist1h4i	N/A	N/A
10408121	---	N/A	N/A
10408200	Hist1h4f	N/A	N/A
10408243	Hist1h4m /// Hist1h4a /// Hist1h4f /// Hist1h4b /// Gm11275	N/A	N/A
10408600	Serpinb6a	N/A	N/A
10408687	Hmgb1	N/A	N/A
10409014	---	N/A	N/A
10409212	---	N/A	N/A
10409220	Hist2h2aa1 /// Hist2h2aa2 /// Hist2h3c1 /// Hist2h2ac	N/A	N/A
10409449	F12	N/A	N/A
10409592	Lect2	N/A	N/A
10409689	Hnrnpk	N/A	N/A
10409968	Rps18	N/A	N/A
10410506	Rpl9 /// Gm5451	N/A	N/A
10410590	Exoc3	N/A	N/A
10410721	Arsk	N/A	N/A
10410921	Cox7c /// Gm10012	N/A	N/A
10411393	Rps18 /// Gm10260	N/A	N/A
10411454	Sec61b	N/A	N/A
10411491	Tnpo1	N/A	N/A
10411882	Nln	N/A	N/A
10412378	H3f3a	N/A	N/A
10413008	Fut11	N/A	N/A
10413014	Chchd1	N/A	N/A
10413174	Rps24 /// Gm6030	N/A	N/A
10413304	Arf4	N/A	N/A
10413542	Tkt	N/A	N/A
10413752	Bap1	N/A	N/A
10414078	Rpl23a /// Gm3940 /// Gm10335	N/A	N/A
10414767	Rps19 /// Rps19-ps3 /// Gm6636	N/A	N/A
10414874	Rps19 /// Rps19-ps3 /// Gm6636	N/A	N/A
10415640	Rpl12 /// Snora65	N/A	N/A
10415660	Rpl13	N/A	N/A
10415955	---	N/A	N/A
10415960	Ints9	N/A	N/A
10416437	Lcp1	N/A	N/A
10416496	Tpt1 /// Tpt1p	N/A	N/A
10416732	Snora30	N/A	N/A
10416793	Uchl3	N/A	N/A
10416835	Rpl17 /// Gm10268 /// Rpl17-ps3	N/A	N/A
10417664	Rpl19	N/A	N/A
10417730	---	N/A	N/A
10417745	Rpl15 /// Gm4294	N/A	N/A
10417794	1810063B07Rik	N/A	N/A
10418164	---	N/A	N/A
10418169	1700054O19Rik	N/A	N/A
10418498	Rpl7a	N/A	N/A
10418804	Timm23	N/A	N/A
10418991	Gcap14	N/A	N/A
10419469	---	N/A	N/A
10419729	Nrbf2	N/A	N/A
10419731	Nrbf2	N/A	N/A
10420011	Myl6 /// Gm8894	N/A	N/A
10420080	Mdp1	N/A	N/A
10420657	Rps12 /// Gm10063 /// Rps12-ps2	N/A	N/A
10420837	Extl3	N/A	N/A
10421526	Rb1	N/A	N/A
10421571	Rps6 /// Rps6-ps2 /// Gm16409	N/A	N/A
10421707	Rpl23a /// Gm3940 /// Gm10335	N/A	N/A
10421972	Rpl36a	N/A	N/A
10422024	---	N/A	N/A
10422057	Rpl7a /// Gm16477 /// Rpl7a-ps10	N/A	N/A
10422161	Gm10293	N/A	N/A
10422512	A2ld1	N/A	N/A
10422699	---	N/A	N/A
10422701	Rpl37	N/A	N/A
10422707	Prkaa1	N/A	N/A
10422852	Rpl19	N/A	N/A
10422962	1110020G09Rik	N/A	N/A
10422980	Lmbrd2	N/A	N/A
10423172	Golph3	N/A	N/A
10423289	---	N/A	N/A
10423293	Myo10	N/A	N/A

10423556	Pgcp	N/A	N/A
10424413	Hmgb1	N/A	N/A
10424439	Efr3a	N/A	N/A
10424559	Khdrbs3	N/A	N/A
10424686	BC025446	N/A	N/A
10424823	Rps6 /// Gm16409	N/A	N/A
10424979	Gpt	N/A	N/A
10425024	1110038F14Rik	N/A	N/A
10425211	Gcat	N/A	N/A
10425628	Gm5805	N/A	N/A
10425721	Mir33	N/A	N/A
10425763	Cyp2d9	N/A	N/A
10425799	Rnu12	N/A	N/A
10425880	Prr5	N/A	N/A
10426435	Gapdh /// Gm12070 /// Gm2606 /// Gm4609 /// Gm10290 /// Gm10291	N/A	N/A
10426437	Rn7sk	N/A	N/A
10426648	Rpl36 /// Gm13611 /// Gm8973	N/A	N/A
10426889	Sec61b	N/A	N/A
10427199	Pfdn5	N/A	N/A
10427205	Myg1	N/A	N/A
10427266	Rpl39	N/A	N/A
10427459	---	N/A	N/A
10427521	Wdr70	N/A	N/A
10427814	Golph3	N/A	N/A
10427849	6030458C11Rik	N/A	N/A
10427995	---	N/A	N/A
10428119	Cox6c	N/A	N/A
10428169	Rpl7a /// Rpl7a-ps3 /// Rpl7a-ps5 /// Gm5619 /// Gm16477 /// Rpl7a-ps10	N/A	N/A
10428338	Dpys	N/A	N/A
10428396	---	N/A	N/A
10428398	Eif3e	N/A	N/A
10428447	---	N/A	N/A
10428576	Rpl15 /// Gm4294	N/A	N/A
10428602	---	N/A	N/A
10428690	Mrpl13	N/A	N/A
10429389	Rpl17 /// Gm10268 /// Rpl17-ps3	N/A	N/A
10429455	Hmgb1 /// Hmg1l1	N/A	N/A
10429588	9030619P08Rik	N/A	N/A
10429666	Pycrl	N/A	N/A
10429957	Fbxl6	N/A	N/A
10430618	Dnalc4	N/A	N/A
10430681	Rps19bp1	N/A	N/A
10430768	---	N/A	N/A
10430851	Cyp2d22	N/A	N/A
10431282	2810001A02Rik	N/A	N/A
10431656	Rpl31 /// Gm16382	N/A	N/A
10432918	Krt8	N/A	N/A
10433049	Atp5g2	N/A	N/A
10433262	Rpl7a /// Gm5766	N/A	N/A
10433562	Rpl30 /// Gm12191 /// Gm6570 /// Gm5481 /// Gm6109	N/A	N/A
10433575	Gm10832	N/A	N/A
10433902	Rpl30 /// Gm12191 /// Gm5481	N/A	N/A
10433937	---	N/A	N/A
10433940	Mapk1	N/A	N/A
10433988	Serpind1	N/A	N/A
10434233	Ufd1l	N/A	N/A
10434643	Psmb3	N/A	N/A
10434645	Senp2	N/A	N/A
10434664	Ndufa11	N/A	N/A
10434743	---	N/A	N/A
10435455	Rpl35a /// Ino80 /// Gm10247	N/A	N/A
10435784	Ndufs5 /// BC002163	N/A	N/A
10435980	Rps24 /// Gm6030	N/A	N/A
10436048	Prdx1	N/A	N/A
10436182	Cd47	N/A	N/A
10436232	Rpl24	N/A	N/A
10436392	Cpox	N/A	N/A
10436402	Cldnd1	N/A	N/A
10436456	Pros1	N/A	N/A
10436748	2310034C09Rik	N/A	N/A
10436783	Sod1	N/A	N/A
10436830	Ifnar2	N/A	N/A
10436890	Gm10785	N/A	N/A
10437594	Usp7	N/A	N/A
10437627	Rpl35a /// Ino80 /// Gm10247	N/A	N/A
10437934	Rpsa /// Rpsa-ps10	N/A	N/A
10438313	Dgcr8 /// Trmt2a	N/A	N/A
10438517	Alg3	N/A	N/A
10438564	Rps10	N/A	N/A
10438583	Rpl12	N/A	N/A
10438911	Atp13a3	N/A	N/A
10439016	Gm5415 /// 4930444G20Rik /// AF366264 /// Gm9839	N/A	N/A
10439204	Rpl15 /// Gm4294	N/A	N/A
10439402	Rpl9 /// Gm5451	N/A	N/A

10439500	Upk1b	N/A	N/A
10439642	Slc35a5	N/A	N/A
10439878	Psmc1	N/A	N/A
10440037	Nit2	N/A	N/A
10440206	Arl6	N/A	N/A
10440414	Fau	N/A	N/A
10440469	---	N/A	N/A
10440543	Ltn1	N/A	N/A
10440661	Rpl13	N/A	N/A
10440849	Synj1	N/A	N/A
10441038	Hlcs	N/A	N/A
10441494	Tulp4	N/A	N/A
10442120	---	N/A	N/A
10442396	Abca3	N/A	N/A
10442454	Pgp	N/A	N/A
10442736	BC003965	N/A	N/A
10442914	0610011F06Rik	N/A	N/A
10442922	Cox7c /// Gm10012	N/A	N/A
10443193	Rpl30 /// Gm12191	N/A	N/A
10443808	Ndufv3	N/A	N/A
10444041	Ndufa7	N/A	N/A
10444780	H2-L /// H2-D1	N/A	N/A
10445430	Mrpl14	N/A	N/A
10446013	Mpnd	N/A	N/A
10446309	Cntnap5c	N/A	N/A
10446615	Rps24 /// Gm6030	N/A	N/A
10446804	Slc30a6	N/A	N/A
10446821	Rps6 /// Gm16409	N/A	N/A
10447286	Rpl31	N/A	N/A
10447412	---	N/A	N/A
10447429	Gm4832	N/A	N/A
10447515	U2af1	N/A	N/A
10447566	Atp5g2	N/A	N/A
10447675	Rnaset2a /// Rnaset2b	N/A	N/A
10447699	---	N/A	N/A
10447799	Igf2r	N/A	N/A
10448048	Psmb1	N/A	N/A
10448380	Tceb2	N/A	N/A
10448865	Gnptg	N/A	N/A
10449312	---	N/A	N/A
10449370	Rps10	N/A	N/A
10449549	Rpl35a /// Ino80 /// Gm10247	N/A	N/A
10449602	1110021J02Rik	N/A	N/A
10449731	U2af1	N/A	N/A
10449955	Cyp4f13	N/A	N/A
10450050	Rps28	N/A	N/A
10450063	Rps18 /// Gm10260	N/A	N/A
10450103	H2-Ke6	N/A	N/A
10450614	2310061I04Rik	N/A	N/A
10450694	H2-T22 /// H2-T10 /// H2-T9	N/A	N/A
10450744	---	N/A	N/A
10451301	Rpl11 /// Gm10288	N/A	N/A
10451677	---	N/A	N/A
10452085	Safb2	N/A	N/A
10452384	Rpl7a /// Rpl7a-ps3 /// Rpl7a-ps5 /// Gm5619 /// Gm16477 /// Rpl7a-ps10	N/A	N/A
10452468	Mrps10	N/A	N/A
10452854	Srd5a2	N/A	N/A
10452890	Tceb2	N/A	N/A
10452935	Heatr5b	N/A	N/A
10453252	Hmgb1	N/A	N/A
10453373	Prepl /// Slc3a1	N/A	N/A
10453690	Rpl27	N/A	N/A
10454057	---	N/A	N/A
10454097	Rpl11 /// Gm7589 /// Gm10288 /// Gm10036	N/A	N/A
10454286	Mapre2	N/A	N/A
10454296	Rpl19	N/A	N/A
10455015	Vaultrc5	N/A	N/A
10455017	Gm6756	N/A	N/A
10455210	Rpl17 /// Gm10268 /// Rpl17-ps3	N/A	N/A
10455227	Rnf14	N/A	N/A
10455439	Hmgb1	N/A	N/A
10456001	Rps14	N/A	N/A
10456423	Seh1l	N/A	N/A
10456579	Mex3c	N/A	N/A
10456709	Rpl17 /// Gm10268 /// Rpl17-ps3	N/A	N/A
10456719	---	N/A	N/A
10456721	---	N/A	N/A
10456812	Hdhd2	N/A	N/A
10457071	Cyb5	N/A	N/A
10457400	---	N/A	N/A
10457686	Dsc2	N/A	N/A
10457731	Rpl35 /// Gm10269	N/A	N/A
10457920	Rps25 /// Gm4963	N/A	N/A
10457927	---	N/A	N/A

10458016	Proc	N/A	N/A
10458386	Ndufa2	N/A	N/A
10458828	Cdo1	N/A	N/A
10459669	Rpl5	N/A	N/A
10459768	---	N/A	N/A
10460118	Socs6	N/A	N/A
10460626	Rnaseh2c	N/A	N/A
10460726	Fau	N/A	N/A
10461017	Bad /// Gpr137	N/A	N/A
10461108	Iscu	N/A	N/A
10461156	Snhg1	N/A	N/A
10461160	---	N/A	N/A
10461162	Snord22	N/A	N/A
10461389	Rps7 /// Gm9493	N/A	N/A
10461640	---	N/A	N/A
10461671	Patl1	N/A	N/A
10461775	Gm5244	N/A	N/A
10461898	Rfk	N/A	N/A
10462140	Dock8	N/A	N/A
10462361	Hmgb1	N/A	N/A
10462363	Jak2	N/A	N/A
10462473	Mbl2	N/A	N/A
10462683	Pcgf5	N/A	N/A
10462861	---	N/A	N/A
10463043	Cyp2c37	N/A	N/A
10463153	Morf4l1	N/A	N/A
10463158	AI606181	N/A	N/A
10463803	Slk	N/A	N/A
10463904	---	N/A	N/A
10464388	Rps12 /// Gm10063 /// Rps12-ps2	N/A	N/A
10464469	Rpl7a /// Gm16477	N/A	N/A
10464825	Ccs	N/A	N/A
10464877	Dpp3	N/A	N/A
10465043	Gm6293	N/A	N/A
10465686	Rtn3	N/A	N/A
10465812	Ttc9c	N/A	N/A
10465831	5730408K05Rik	N/A	N/A
10466127	AW112010	N/A	N/A
10466314	---	N/A	N/A
10466402	Eif4a1	N/A	N/A
10466771	Fxn	N/A	N/A
10466839	---	N/A	N/A
10466923	Rpl26 /// Gm10136 /// Gm15772	N/A	N/A
10466947	Ermp1	N/A	N/A
10467162	Pank1	N/A	N/A
10467256	Rpl10	N/A	N/A
10467380	Cyp2c67	N/A	N/A
10467385	Cyp2c68	N/A	N/A
10467390	Cyp2c40 /// Cyp2c67 /// Cyp2c68 /// Cyp2c69	N/A	N/A
10467400	Cyp2c54	N/A	N/A
10468016	Ndufb8	N/A	N/A
10468131	9130011E15Rik	N/A	N/A
10468789	Pdzd8	N/A	N/A
10468929	Nmt2	N/A	N/A
10469035	Sephs1	N/A	N/A
10469151	Itih5	N/A	N/A
10469571	Otud1	N/A	N/A
10470283	Egfl7	N/A	N/A
10470305	Rpl7a /// Rpl7a-ps3 /// Rpl7a-ps5 /// Gm5619 /// Gm16477 /// Rpl7a-ps10	N/A	N/A
10470316	---	N/A	N/A
10470834	Spna2	N/A	N/A
10470893	BC085271 /// Set	N/A	N/A
10471036	Dolpp1	N/A	N/A
10471655	Gsn	N/A	N/A
10471951	---	N/A	N/A
10471953	Acvr2a	N/A	N/A
10472212	Pkp4	N/A	N/A
10472587	Rpl13	N/A	N/A
10472686	Rpl9 /// Gm5451	N/A	N/A
10472764	Dync1i2	N/A	N/A
10473160	Ssfa2	N/A	N/A
10473230	Nup35	N/A	N/A
10473281	Itgav	N/A	N/A
10473414	Ssrp1	N/A	N/A
10473547	Srp9	N/A	N/A
10473564	Olfr1143	N/A	N/A
10474112	Traf6	N/A	N/A
10474379	Hadhb	N/A	N/A
10474541	Nop10	N/A	N/A
10474545	Slc12a6	N/A	N/A
10474577	Slc12a6 /// 2410042D21Rik	N/A	N/A
10474867	---	N/A	N/A
10474915	Gchfr	N/A	N/A
10475435	Rps12 /// Gm10063 /// Rps12-ps2	N/A	N/A

10475514	---	N/A	N/A
10476287	Pank2	N/A	N/A
10476383	Crls1	N/A	N/A
10476592	Macrod2	N/A	N/A
10476648	Dstn	N/A	N/A
10476725	Gm561	N/A	N/A
10477100	---	N/A	N/A
10477167	Mcts2	N/A	N/A
10477264	Tm9sf4	N/A	N/A
10477543	Cbfa2t2	N/A	N/A
10477581	Rpl5	N/A	N/A
10478341	Ift52	N/A	N/A
10479172	---	N/A	N/A
10479174	Rps8 /// Gm11353 /// Rps8-ps1	N/A	N/A
10479187	Rps8 /// Gm11353 /// Rps8-ps1	N/A	N/A
10479215	Rps8 /// Gm11353 /// Rps8-ps1	N/A	N/A
10479809	Rps28	N/A	N/A
10479996	Atp5c1	N/A	N/A
10480032	Gapdh /// Gm16374 /// Gm2606 /// Gm4609 /// Gm3200 /// Gm2451 /// Gm10293	N/A	N/A
10480345	---	N/A	N/A
10480421	Pdss1	N/A	N/A
10480579	Mrpl41 /// Wdr85	N/A	N/A
10481962	Hc	N/A	N/A
10482109	Rbm18 /// Mrrf	N/A	N/A
10482119	---	N/A	N/A
10482267	Rpl35 /// Gm10269	N/A	N/A
10482920	Cd302	N/A	N/A
10483322	---	N/A	N/A
10483563	Tlk1	N/A	N/A
10483786	Mrpl23	N/A	N/A
10483806	Rps6 /// Gm16409	N/A	N/A
10483865	Fkbp7	N/A	N/A
10484357	Rps4x	N/A	N/A
10484925	Gm13777	N/A	N/A
10485294	Hsd17b12	N/A	N/A
10485654	Rpl10	N/A	N/A
10485685	Rpl35a /// Ino80 /// Gm10247	N/A	N/A
10486107	Srp14	N/A	N/A
10486595	Ttbk2	N/A	N/A
10487513	Anapc1	N/A	N/A
10487711	Ddrgk1	N/A	N/A
10487925	Rpl18	N/A	N/A
10488020	Tmx4	N/A	N/A
10488892	Trpc4ap	N/A	N/A
10489045	Rpl37a /// Mir682	N/A	N/A
10489235	9430008C03Rik	N/A	N/A
10489553	Acot8 /// Snx21	N/A	N/A
10490256	Rps8 /// Gm11353 /// Rps8-ps1	N/A	N/A
10490350	Ppp1r3d	N/A	N/A
10491058	Rprl2	N/A	N/A
10491064	---	N/A	N/A
10491091	Tnfsf10	N/A	N/A
10491406	Ndufb5	N/A	N/A
10491967	Gapdh /// Gm4609 /// Gm10293	N/A	N/A
10492229	Rpl13	N/A	N/A
10492310	Mbnl1	N/A	N/A
10492558	Smc4	N/A	N/A
10492582	Mir15b	N/A	N/A
10492590	Ppm1l	N/A	N/A
10493409	Scamp3	N/A	N/A
10494296	Rps10	N/A	N/A
10494335	Otud7b	N/A	N/A
10494411	Rnu1b1 /// Rnu1b6 /// Rnu1b2	N/A	N/A
10494417	Rnu1b1 /// Rnu1b6 /// Rnu1b2	N/A	N/A
10494445	Lix1l	N/A	N/A
10494527	Rnf115	N/A	N/A
10494924	Trim33	N/A	N/A
10495518	Olfm3	N/A	N/A
10495659	Cnn3	N/A	N/A
10495820	Fabp2	N/A	N/A
10495989	Rpl7a /// Gm5619 /// Gm16477 /// Rpl7a-ps10	N/A	N/A
10496295	Ube2d3	N/A	N/A
10496379	H2afz	N/A	N/A
10496475	Adh5	N/A	N/A
10496490	Mir1956 /// Eif4e	N/A	N/A
10496626	Gapdh /// Gm16374 /// Gm2606 /// Gm4609 /// Gm3200 /// Gm2451 /// Gm10293	N/A	N/A
10496629	Sep 15	N/A	N/A
10496735	Rpl36a	N/A	N/A
10496835	Hmgb1	N/A	N/A
10497173	Pxmp3	N/A	N/A
10497237	Pag1	N/A	N/A
10497296	Slc10a5	N/A	N/A
10497325	Hmgb1	N/A	N/A
10497483	Hmgb1	N/A	N/A

10497673	Zmat3	N/A	N/A
10498405	Gapdh /// Gm12070	N/A	N/A
10498775	Golim4	N/A	N/A
10498871	Tmem144	N/A	N/A
10499198	Rpl30 /// Gm12191 /// Gm6570 /// Gm6109	N/A	N/A
10499748	Rps27 /// Gm9846	N/A	N/A
10499854	S100a1	N/A	N/A
10499879	Rpl3	N/A	N/A
10499881	Rpl3	N/A	N/A
10499937	---	N/A	N/A
10499996	Snx27	N/A	N/A
10500009	Rpl31 /// Gm16382	N/A	N/A
10500293	---	N/A	N/A
10500345	Terc	N/A	N/A
10500356	Rnu1b1 /// Rnu1b6 /// Rnu1b2	N/A	N/A
10500543	Hmgb1	N/A	N/A
10500736	Vangl1	N/A	N/A
10500990	Atp5f1	N/A	N/A
10501179	Rpl13	N/A	N/A
10501742	Rpl7a-ps3	N/A	N/A
10501811	Slc44a3	N/A	N/A
10501857	Gapdh /// Gm16374 /// Gm2606 /// Gm4609 /// Gm3200 /// Gm2451 /// Gm10293	N/A	N/A
10502196	Rpl34	N/A	N/A
10502375	Mttp	N/A	N/A
10502714	Rpf1	N/A	N/A
10503249	Rpl13	N/A	N/A
10503374	---	N/A	N/A
10503399	Myl6 /// Gm8894	N/A	N/A
10503508	Ggh	N/A	N/A
10503523	Ggh	N/A	N/A
10503551	Usp45	N/A	N/A
10503833	Rplp1	N/A	N/A
10503915	Chchd2	N/A	N/A
10504008	Chmp5	N/A	N/A
10504056	Ubap1	N/A	N/A
10504316	Tesk1	N/A	N/A
10504398	Serf2	N/A	N/A
10504564	Dcaf10	N/A	N/A
10504751	---	N/A	N/A
10504849	Stx17	N/A	N/A
10505109	BC026590	N/A	N/A
10505917	---	N/A	N/A
10506397	Mier1	N/A	N/A
10506496	---	N/A	N/A
10506680	Tmem48	N/A	N/A
10506767	Echdc2	N/A	N/A
10507171	Cyp4a31	N/A	N/A
10507177	Cyp4a10 /// Cyp4a31 /// Cyp4a32	N/A	N/A
10507286	Ipp /// Tmem69	N/A	N/A
10507328	Prdx1	N/A	N/A
10507520	Hyi	N/A	N/A
10507784	Ppt1	N/A	N/A
10507870	---	N/A	N/A
10507872	---	N/A	N/A
10508721	Snora44	N/A	N/A
10508723	Snora61	N/A	N/A
10509014	D4Wsu53e	N/A	N/A
10509275	Rpl31 /// Gm16382	N/A	N/A
10509635	Akr7a5	N/A	N/A
10510025	Agmat	N/A	N/A
10510464	Lzic	N/A	N/A
10511149	Mrpl20	N/A	N/A
10511190	Dvl1	N/A	N/A
10511269	Sdf4	N/A	N/A
10511325	Rps20	N/A	N/A
10511444	Gm11810	N/A	N/A
10511498	Plekhf2	N/A	N/A
10511629	Rpl26 /// Gm10136 /// Gm15772	N/A	N/A
10511739	Cpne3	N/A	N/A
10512022	Mobkl2b	N/A	N/A
10512063	---	N/A	N/A
10512195	Ubap2	N/A	N/A
10512274	Enho	N/A	N/A
10512487	Rmrp	N/A	N/A
10512701	Tomm5	N/A	N/A
10512728	Tstd2 /// Tmod1	N/A	N/A
10512939	2610030H06Rik	N/A	N/A
10512949	Abca1	N/A	N/A
10513006	---	N/A	N/A
10513145	Ptpn3	N/A	N/A
10513154	Ptpn3	N/A	N/A
10513158	Ptpn3	N/A	N/A
10513529	Mup3	N/A	N/A
10513737	Rpl17 /// Gm10268 /// Rpl17-ps3	N/A	N/A

10513878	Rps18 /// Gm10260	N/A	N/A
10514173	Rpl34 /// Gm6404 /// Gm10154	N/A	N/A
10514201	Haus6	N/A	N/A
10514233	Rps6 /// Gm16409	N/A	N/A
10514902	Lrrc42	N/A	N/A
10514985	Zyg11b	N/A	N/A
10515154	Rpl3	N/A	N/A
10515326	Tmem69	N/A	N/A
10515416	Rps8 /// Gm5121 /// Gm11353 /// Rps8-ps1	N/A	N/A
10515427	---	N/A	N/A
10515429	Snord55	N/A	N/A
10515696	Szt2	N/A	N/A
10516211	Ndufs5	N/A	N/A
10516371	Eif2c1	N/A	N/A
10516479	Rps28	N/A	N/A
10516640	Eif3i	N/A	N/A
10516765	Serinc2	N/A	N/A
10517457	Rpl11 /// Gm7589 /// Gm10288 /// Gm10036	N/A	N/A
10517600	Pink1	N/A	N/A
10517948	Spen	N/A	N/A
10518031	Dnajc16	N/A	N/A
10518453	Chchd2	N/A	N/A
10518679	Nmnat1	N/A	N/A
10518841	Thap3	N/A	N/A
10519096	Rer1	N/A	N/A
10519136	---	N/A	N/A
10520467	Hadhb	N/A	N/A
10520842	Bre	N/A	N/A
10521036	Rpl35a /// Ino80 /// Gm10247	N/A	N/A
10521593	Rpl7 /// Gm5045	N/A	N/A
10521709	Lap3	N/A	N/A
10521813	Nsfl1c	N/A	N/A
10521913	Rbpj	N/A	N/A
10521969	Immp1l	N/A	N/A
10522396	Ociad1	N/A	N/A
10522782	Rpl7 /// Gm5045	N/A	N/A
10523275	---	N/A	N/A
10523354	Gapdh /// Gm2606 /// Gm10293	N/A	N/A
10523756	---	N/A	N/A
10523893	Rpl5	N/A	N/A
10523960	Pigg	N/A	N/A
10524018	Rpl34 /// Gm6404 /// Gm10154	N/A	N/A
10524310	Ttc28	N/A	N/A
10524621	Oasl2	N/A	N/A
10525195	Gm15800	N/A	N/A
10525406	Anapc7	N/A	N/A
10525471	Ndufb11	N/A	N/A
10525983	Ran	N/A	N/A
10526085	---	N/A	N/A
10526215	Rps28	N/A	N/A
10526381	Mdh2	N/A	N/A
10526792	0910001L09Rik	N/A	N/A
10527306	Lmtk2	N/A	N/A
10527448	Bud31 /// Ptcd1	N/A	N/A
10528167	Gapdh /// Gm16374 /// Gm4609 /// Gm3200 /// Gm2451 /// Gm10293	N/A	N/A
10528227	Gnai1	N/A	N/A
10528478	Rpl17 /// Gm10268 /// Rpl17-ps3	N/A	N/A
10528523	Tomm7	N/A	N/A
10528544	Rpl11 /// Gm10288 /// Gm10036	N/A	N/A
10528662	Atp5l	N/A	N/A
10528970	---	N/A	N/A
10529794	---	N/A	N/A
10529858	Tapt1	N/A	N/A
10530140	---	N/A	N/A
10530283	---	N/A	N/A
10530287	Apbb2	N/A	N/A
10530563	Fryl	N/A	N/A
10530592	Fryl	N/A	N/A
10531340	Rpl13	N/A	N/A
10531910	Hsd17b13	N/A	N/A
10532025	Rps15a	N/A	N/A
10532027	Gapdh /// Gm16374 /// Gm2606 /// Gm4609 /// Gm3200 /// Gm2451 /// Gm10293	N/A	N/A
10532030	---	N/A	N/A
10532339	Pxmp2	N/A	N/A
10532767	Ssh1	N/A	N/A
10532993	Cox6a1	N/A	N/A
10533526	---	N/A	N/A
10533549	Anapc5	N/A	N/A
10533612	Hpd	N/A	N/A
10533626	Rpl35a /// Ino80 /// Gm10247	N/A	N/A
10534096	Chchd2	N/A	N/A
10534405	Wbscr22 /// Dnajc30	N/A	N/A
10534583	---	N/A	N/A
10534660	Ap1s1	N/A	N/A

10534694	Srrt	N/A	N/A
10534964	Rps25 /// Gm4963	N/A	N/A
10535471	Rac1 /// Daglb	N/A	N/A
10535575	Gm6272	N/A	N/A
10535647	Atp5j2	N/A	N/A
10536505	Met	N/A	N/A
10536996	Klhdc10	N/A	N/A
10537244	Rpl17 /// Rpl17-ps3	N/A	N/A
10537349	Rpl30 /// Gm12191 /// Gm6570 /// Gm6109	N/A	N/A
10537394	1110001J03Rik	N/A	N/A
10537452	Ndufb2	N/A	N/A
10537504	---	N/A	N/A
10537880	---	N/A	N/A
10537909	Rny3	N/A	N/A
10538080	Rpl35a /// Ino80 /// Gm10247	N/A	N/A
10538420	Gars	N/A	N/A
10538638	---	N/A	N/A
10538842	Gng12	N/A	N/A
10538963	Rpl34 /// Gm6404 /// Gm10154	N/A	N/A
10539159	Suclg1	N/A	N/A
10539484	Rpl18	N/A	N/A
10539700	Rps28	N/A	N/A
10540507	H3f3a	N/A	N/A
10540542	LOC100503669	N/A	N/A
10540999	H2afz	N/A	N/A
10541094	Zfp637	N/A	N/A
10541494	Rps27a	N/A	N/A
10542038	Hmgb1	N/A	N/A
10542077	Rpl18	N/A	N/A
10542470	Mgst1	N/A	N/A
10542677	Etnk1	N/A	N/A
10542722	Rps25 /// Gm4963	N/A	N/A
10542872	Rps4y2	N/A	N/A
10543052	Rps27a	N/A	N/A
10543118	Glcci1	N/A	N/A
10543134	Ndufa4	N/A	N/A
10543464	Rpl7a	N/A	N/A
10543549	---	N/A	N/A
10543859	Akr1b3 /// Gm6644	N/A	N/A
10544540	Rpl35a /// Ino80 /// Gm10247	N/A	N/A
10544573	Lrrc61 /// Rarres2	N/A	N/A
10544640	Tra2a	N/A	N/A
10544812	Rpl38	N/A	N/A
10544932	Inmt	N/A	N/A
10545125	Rpl23	N/A	N/A
10545192	Rprl1	N/A	N/A
10545200	---	N/A	N/A
10545337	Ppia	N/A	N/A
10545339	Mrpl35	N/A	N/A
10545765	Gapdh /// Gm16374 /// Gm2606 /// Gm4609 /// Gm3200 /// Gm2451 /// Gm10293	N/A	N/A
10545881	Cml2	N/A	N/A
10546066	Isy1	N/A	N/A
10546079	Cnbp	N/A	N/A
10546702	Tpt1 /// Tpt1p	N/A	N/A
10546919	Rpusd3	N/A	N/A
10547068	Rpl32	N/A	N/A
10547073	Snora7a	N/A	N/A
10547386	Adipor2	N/A	N/A
10547638	Rpl15 /// Gm4294	N/A	N/A
10548143	Gapdh /// Gm16374 /// Gm4609 /// Gm3200 /// Gm2451 /// Gm10293	N/A	N/A
10548600	Hmgb1 /// Hmgb1l	N/A	N/A
10548879	Mgp	N/A	N/A
10549375	Rps26 /// Gm6654	N/A	N/A
10549582	Tsen34	N/A	N/A
10549588	Rps9	N/A	N/A
10549802	Rps18 /// Gm5321 /// Gm10260	N/A	N/A
10550052	Rps5	N/A	N/A
10551287	Cyp2a12	N/A	N/A
10551347	Blvrb	N/A	N/A
10551489	Rps16 /// Rps16-ps2	N/A	N/A
10551529	LOC100503763	N/A	N/A
10551760	Zfp84	N/A	N/A
10551998	---	N/A	N/A
10552242	Rpl17 /// Gm10268 /// Rpl17-ps3	N/A	N/A
10552343	---	N/A	N/A
10553475	Rps27a	N/A	N/A
10553840	Atp5l	N/A	N/A
10553857	Hmgb1	N/A	N/A
10554005	H47	N/A	N/A
10554701	Hnrnpk	N/A	N/A
10555063	Ints4	N/A	N/A
10555568	Rpl31	N/A	N/A
10556206	Snora3	N/A	N/A
10556244	Snora23	N/A	N/A

10556571	Rpl19	N/A	N/A
10556940	Uqcrc2 /// Pdzd9	N/A	N/A
10557703	Snora30	N/A	N/A
10558227	Acadsb	N/A	N/A
10558687	1190003J15Rik	N/A	N/A
10558903	Taldo1	N/A	N/A
10559233	Mrpl23	N/A	N/A
10560045	Slc27a5	N/A	N/A
10560103	Rps8	N/A	N/A
10560614	Apoc4 /// Apoc2	N/A	N/A
10560816	Atp5g2	N/A	N/A
10561140	Mrpl41	N/A	N/A
10561679	Psmd8	N/A	N/A
10562096	Tmem147	N/A	N/A
10562289	Gm5329	N/A	N/A
10562663	Rps4x	N/A	N/A
10562667	---	N/A	N/A
10563026	Bcl2l12	N/A	N/A
10563099	Snord35b	N/A	N/A
10563114	Rpl13a /// Snord32a	N/A	N/A
10564563	Rpl17 /// Gm10268 /// Rpl17-ps3	N/A	N/A
10564570	---	N/A	N/A
10564622	---	N/A	N/A
10564857	Idh2	N/A	N/A
10565083	Rps17 /// Gm6402	N/A	N/A
10565434	Rps13	N/A	N/A
10565530	---	N/A	N/A
10565570	4632434I11Rik	N/A	N/A
10565589	Hmgb1	N/A	N/A
10565813	Snord15a	N/A	N/A
10566142	Hmgb1	N/A	N/A
10566477	Hpx	N/A	N/A
10567106	Rps4y2 /// Gm6816	N/A	N/A
10568532	Rpl7a /// Rpl7a-ps3 /// Rpl7a-ps5 /// Gm5619 /// Gm16477 /// Rpl7a-ps10	N/A	N/A
10578557	Ccdc111 /// Mlf1ip	N/A	N/A
10588026	Rpl7a /// Rpl7a-ps3 /// Rpl7a-ps5 /// Gm5619 /// Gm16477 /// Rpl7a-ps10	N/A	N/A
10594907	Rpl7a /// Rpl7a-ps3 /// Rpl7a-ps5 /// Gm5619 /// Gm16477 /// Rpl7a-ps10	N/A	N/A
10595441	Rpl7a /// Rpl7a-ps3 /// Rpl7a-ps5 /// Gm5619 /// Gm16477 /// Rpl7a-ps10	N/A	N/A
10597515	Rpl24	N/A	N/A
10600027	Rpl30 /// Gm12191 /// Gm6570 /// Gm5481 /// Gm6109	N/A	N/A
10606369	Itm2a	N/A	N/A
Liver Class VI			
Probe ID	**Gene ID**	**Con Peak**	**TSR Peak**
10344707	Pcmtd1	N/A	1
10344789	Cspp1	N/A	1
10344799	Cspp1	N/A	1
10344801	Cspp1	N/A	1
10344805	Cspp1	N/A	1
10344811	Cspp1	N/A	1
10344815	Cspp1	N/A	1
10344817	Cspp1	N/A	1
10344819	Cspp1	N/A	1
10344924	Xkr9	N/A	19
10344939	Terf1	N/A	1
10344952	Rdh10	N/A	13
10345025	Iars	N/A	19
10345089		N/A	13
10345436	LOC280487	N/A	7
10345546	LOC100046859	N/A	1
10345928		N/A	13
10345930	Tpp2	N/A	1
10345967	Bivm	N/A	1
10346222	LOC280487	N/A	7
10346310	Mobkl3	N/A	1
10346340	9430016H08Rik	N/A	13
10346374	Aox1	N/A	1
10346544		N/A	13
10346551	Cflar	N/A	1
10346695	Nbeal1	N/A	1
10346722	Nbeal1	N/A	1
10346882	Adam23	N/A	13
10346970	Pikfyve	N/A	1
10347106	Rpe	N/A	1
10347297	Arpc2	N/A	13
10347417	Bcs1l	N/A	7
10347481	Cyp27a1	N/A	13
10347508	Fam134a	N/A	7
10347531	Ankzf1	N/A	1
10347552	Stk16	N/A	13
10347639	Gmppa	N/A	1
10347724	Slc4a3	N/A	13
10347779	9830004L10Rik	N/A	13
10347790		N/A	13

10348004	Psmd1	N/A	1
10348201	Gigyf2	N/A	1
10348632	Twist2	N/A	19
10349016	2310035C23Rik	N/A	1
10349249	Clasp1	N/A	1
10349442	Ccnt2	N/A	1
10349478	Rpl28	N/A	7
10349826	Gm10538	N/A	13
10350046	Kdm5b	N/A	1
10350136	Csrp1	N/A	7
10350341		N/A	13
10350590	Fam33a	N/A	7
10350800	Tor1aip2	N/A	13
10350923	Rfwd2	N/A	1
10351035	Gas5	N/A	1
10351037	Gas5	N/A	1
10351390	Tada1l	N/A	13
10351463	Rgs5	N/A	13
10351487	Nos1ap	N/A	19
10351489	Gm7299	N/A	13
10351533	Nr1i3	N/A	19
10351574	Dedd	N/A	7
10351788	Pigm	N/A	1
10351857		N/A	13
10352194	Cdc42bpa	N/A	1
10352281	Acbd3	N/A	1
10352320	Tmem63a	N/A	19
10352514	Eprs	N/A	1
10352562	Gpatch2	N/A	1
10352777	Slc30a1	N/A	19
10352914	A330023F24Rik	N/A	1
10352916	C030002C11Rik	N/A	1
10352918	C030002C11Rik	N/A	1
10353028	Vcpip1	N/A	1
10353115		N/A	13
10353303		N/A	7
10353415	Il17f	N/A	13
10353549	Fam135a	N/A	1
10353989		N/A	1
10354203		N/A	1
10354307	Txn1	N/A	13
10354368		N/A	13
10354432	Myo1b	N/A	1
10354542	Pms1	N/A	1
10354737	Gm5147	N/A	7
10354765	Gm7098	N/A	7
10354816	Clk1	N/A	1
10355138		N/A	13
10355141	Klf7	N/A	19
10355266	Lancl1	N/A	13
10355534	Tns1	N/A	19
10355742	Abcb6	N/A	13
10355996	Slc25a5	N/A	19
10356248	C130026I21Rik	N/A	13
10356278	Sp110	N/A	13
10356423	Usp40	N/A	1
10356512	Iqca	N/A	13
10356762	LOC280487	N/A	7
10356771	Mterfd2	N/A	7
10356968	Pam	N/A	13
10357001		N/A	13
10357008	Pign	N/A	1
10357298	LOC280487	N/A	7
10357773	Mdm4	N/A	1
10357875	Btg2	N/A	7
10357950	Ppp1r12b	N/A	19
10357954	Ppp1r12b	N/A	1
10358324	Cfhr3	N/A	1
10358339	Cfh	N/A	1
10358430	Gm9931	N/A	13
10358533	Hmcn1	N/A	19
10358591	Hmcn1	N/A	1
10358613	Hmcn1	N/A	13
10358627	Hmcn1	N/A	13
10358631	Hmcn1	N/A	19
10358658	Hmcn1	N/A	19
10358668	Hmcn1	N/A	1
10358677	1200016B10Rik	N/A	1
10358757	Smg7	N/A	1
10358982	Mr1	N/A	19
10359078		N/A	1
10359086		N/A	1
10359097	Tor1aip1	N/A	7
10359181	Tor3a	N/A	19

10359375	Gpr52	N/A	1
10359405	Klhl20	N/A	1
10359561	Fmo4	N/A	19
10359642	LOC280487	N/A	7
10359644	Mettl11b	N/A	13
10359826	Uqcr	N/A	7
10360076	Ndufs2	N/A	7
10360130	Nit1	N/A	7
10360443	Fh1	N/A	13
10360479	Cep170	N/A	1
10360542	Fam36a	N/A	1
10360544	Hnrnpu	N/A	1
10360804	Gm10517	N/A	13
10360832	1700056E22Rik	N/A	13
10360848	Mosc2	N/A	13
10360884	Iars2	N/A	1
10361023	Prox1	N/A	1
10361078	Tatdn3	N/A	19
10361091	Atf3	N/A	13
10361682	Ppil4	N/A	1
10361698	Zc3h12d	N/A	7
10361765		N/A	13
10361977	Gm6251	N/A	19
10362160		N/A	7
10362162	Taar7d	N/A	13
10362387		N/A	19
10362462	Trdn	N/A	7
10362487	Tspyl1	N/A	1
10362520	Hdac2	N/A	1
10362676	Cdc2l6	N/A	7
10362837	Sec63	N/A	1
10362876	Pdss2	N/A	1
10363005	LOC280487	N/A	7
10363007	Ascc3	N/A	1
10363054		N/A	1
10363144		N/A	13
10363173	Gja1	N/A	13
10363181	Stard6	N/A	13
10363190		N/A	13
10363403	Ascc1	N/A	13
10363561	LOC280487	N/A	7
10363641	Herc4	N/A	1
10363703		N/A	1
10363773		N/A	19
10364194	Lss	N/A	19
10364675	Gpx4	N/A	13
10364888	Dot1l	N/A	13
10364909	Oaz1	N/A	13
10365056	Apba3	N/A	13
10365217	Gm10776	N/A	1
10365225	Gm4924	N/A	1
10365286	Eid3	N/A	1
10365471	Fbxo7	N/A	13
10365559	Igf1	N/A	19
10365572		N/A	13
10365574	Pmch	N/A	13
10365714		N/A	1
10365729	Pctk2	N/A	1
10365933	Eea1	N/A	1
10366043	Dusp6	N/A	13
10366310	Osbpl8	N/A	1
10366409	Zfc3h1	N/A	1
10366512		N/A	1
10366519	5330438D12Rik	N/A	19
10366649	4921513I03Rik	N/A	19
10367050	Rdh18	N/A	13
10367066	Sdr9c7	N/A	1
10367120		N/A	1
10367252	Usp52	N/A	1
10367335		N/A	13
10367479	Olfr1518	N/A	13
10367485	Olfr780	N/A	13
10367746	Sash1	N/A	7
10367803		N/A	1
10367843	Utrn	N/A	1
10368064	Ect2l	N/A	13
10368162	Pex7	N/A	19
10368173	4933406P04Rik	N/A	19
10368575		N/A	13
10368713	AI317395	N/A	1
10368739		N/A	19
10368762	Cdc40	N/A	1
10368990		N/A	13
10369116	Mcm9	N/A	1

10369171	9530009G21Rik	N/A	1
10369223	Dux	N/A	13
10369225	Dux	N/A	13
10369228	Gm4981	N/A	7
10369661	Ccar1	N/A	1
10369738	Sirt1	N/A	1
10369833	Slc16a9	N/A	13
10369885	Cisd1	N/A	13
10370174	A130042E20Rik	N/A	13
10370544	2610008E11Rik	N/A	1
10370559	Mier2	N/A	13
10370610	Polrmt	N/A	13
10370708	Polr2e	N/A	13
10370833	Uqcr	N/A	7
10370920	Btbd2	N/A	7
10371277	B230315N10Rik	N/A	1
10371482	Hsp90b1	N/A	1
10371616	Chpt1	N/A	19
10371770	Gas2l3	N/A	19
10371784	Nr1h4	N/A	19
10371888	Tmpo	N/A	1
10371904	1110012L19Rik	N/A	13
10372028	Plxnc1	N/A	13
10372082	Nudt4	N/A	1
10372121	Tmtc3	N/A	1
10372385	Zdhhc17	N/A	1
10372441		N/A	13
10372497	Thap2	N/A	1
10372534	Cnot2	N/A	1
10372583	Rab3ip	N/A	13
10372600	Cct2	N/A	13
10372629	Yeats4	N/A	7
10372668	Mdm2	N/A	1
10372766	Helb	N/A	19
10372831	Tbc1d30	N/A	7
10373569		N/A	7
10373600	Mettl7b	N/A	7
10373626	Olfr796	N/A	13
10373702	Pisd-ps1	N/A	19
10374032		N/A	13
10374035	Xbp1	N/A	7
10374183	Gm4638	N/A	7
10374185	Ccm2	N/A	13
10374348	Eef2	N/A	13
10374350		N/A	19
10374352	Gm4638	N/A	7
10374354		N/A	13
10374364	Akt2	N/A	7
10374485	Peli1	N/A	1
10374551		N/A	13
10374560	Zrsr1	N/A	1
10374621	Usp34	N/A	1
10374821	Smek2	N/A	1
10375051	Hba-a1	N/A	1
10375058	Hba-a1	N/A	1
10375324	LOC280487	N/A	7
10375501		N/A	1
10375547	Olfr10	N/A	7
10375574	Olfr1373	N/A	13
10375578	Flt4	N/A	13
10375704	3010026O09Rik	N/A	7
10375713	Mgat4b	N/A	13
10375735	Hnrnph1	N/A	1
10375791	Olfr51	N/A	13
10375880	Nhp2	N/A	13
10375941	Vdac1	N/A	13
10376324	Gm12250	N/A	7
10376358	Sh3bp5l	N/A	13
10376425	Gm12258	N/A	1
10376474	Mrpl55	N/A	7
10376864	Ubb	N/A	7
10377416	2310047M10Rik	N/A	13
10377550		N/A	1
10377790	Slc16a11	N/A	7
10378013	Rpain	N/A	13
10378334	Tax1bp3	N/A	13
10378429	Olfr402	N/A	13
10378547		N/A	13
10378572	Tlcd2	N/A	19
10378785	Bhlha9	N/A	13
10378802	Blmh	N/A	13
10379068	Sdf2	N/A	13
10379215	Ift20	N/A	1
10380116	Rnf43	N/A	1

10380129	Supt4h1	N/A	13
10380549		N/A	1
10380558		N/A	1
10380689	Cbx1	N/A	13
10380719	Sp6	N/A	13
10380871	Stard3	N/A	13
10381226	Coasy	N/A	13
10381387	G6pc	N/A	1
10381408	Ifi35	N/A	13
10381526	Ppih	N/A	13
10381588	Grn	N/A	13
10381934	Tanc2	N/A	1
10382049	Ddx42	N/A	1
10382069	Psmc5	N/A	13
10382257	Amz2	N/A	13
10382435	Gprc5c	N/A	7
10382516	Kctd2	N/A	13
10382565	Mrps7	N/A	13
10382795	Gm11741	N/A	13
10383010	Socs3	N/A	13
10383194		N/A	13
10383289	Baiap2	N/A	19
10383409	Gcgr	N/A	13
10383564	Fn3k	N/A	7
10384145	H2afv	N/A	7
10384448	Sec61g	N/A	1
10384486	Etaa1	N/A	7
10384504	Meis1	N/A	1
10384566		N/A	1
10384579	Ugp2	N/A	7
10384715	LOC100134990	N/A	1
10384737	Papolg	N/A	1
10384795	Cyb5d1	N/A	7
10385239	Mat2b	N/A	7
10385271	Ccng1	N/A	1
10385477		N/A	7
10385616	Rufy1	N/A	1
10386070	Atox1	N/A	13
10386095	Fam114a2	N/A	1
10386123		N/A	7
10386169	Gm9900	N/A	1
10386551	Flii	N/A	7
10386582	Top3a	N/A	7
10386604	Shmt1	N/A	19
10386652	Aldh3a2	N/A	19
10386683	Slc47a1	N/A	1
10386743	Epn2	N/A	13
10386992	1700086D15Rik	N/A	13
10387111	2310004I24Rik	N/A	1
10387363	Cyb5d1	N/A	13
10387514		N/A	13
10387659	Nlgn2	N/A	13
10387768	Acadvl	N/A	13
10388033	Derl2	N/A	1
10388278		N/A	13
10388286	Olfr391	N/A	13
10388613	Ccdc55	N/A	1
10388718	Pipox	N/A	1
10389010	5730455P16Rik	N/A	1
10389070		N/A	1
10389245	Tada2l	N/A	1
10389308	Ggnbp2	N/A	1
10389522		N/A	7
10389524		N/A	1
10389717		N/A	7
10389882	3300001P08Rik	N/A	1
10390473	Pip4k2b	N/A	19
10391277	Ghdc	N/A	1
10391407	Ccdc56	N/A	13
10391732	Gpatch8	N/A	7
10391744	Gpatch8	N/A	1
10392012	Cdc27	N/A	1
10392183	Ern1	N/A	1
10392221	Pecam1	N/A	7
10392297	Memo1	N/A	13
10392449	Wipi1	N/A	7
10393395	Sfrs2	N/A	19
10393569	RP23-394O9.3	N/A	13
10393642	Eif4a3	N/A	13
10393877	Mafg	N/A	7
10394040	Csnk1d	N/A	13
10394080		N/A	1
10394344		N/A	19
10394366	Atad2b	N/A	1

10394392	Nfyc	N/A	19
10394394	Apob	N/A	19
10394527	Ttc32	N/A	7
10394611	Nbas	N/A	1
10394674	Socs2	N/A	7
10394731	Taf1d	N/A	19
10394819		N/A	13
10394821		N/A	13
10394922	Gm6950	N/A	1
10394926		N/A	13
10395275	LOC280487	N/A	7
10395414	Tmem195	N/A	19
10395520	Immp2l	N/A	19
10395628	Scfd1	N/A	19
10395682	Gm5785	N/A	1
10395692	Arhgap5	N/A	1
10395739	Srp54b	N/A	1
10395788	Srp54a	N/A	1
10395910	Pnn	N/A	1
10396008	Prpf39	N/A	1
10396121	4930512B01Rik	N/A	13
10396205		N/A	1
10396383	Slc38a6	N/A	1
10396485	Syne2	N/A	1
10396608	Syne2	N/A	1
10397153	Acot4	N/A	19
10397216	Coq6	N/A	13
10397428	1700020O03Rik	N/A	19
10397543	EG435337	N/A	1
10397642		N/A	13
10397683	Ttc8	N/A	7
10398315		N/A	13
10398374	AF357341	N/A	13
10398430		N/A	19
10398483	Dync1h1	N/A	13
10398721	2810002N01Rik	N/A	1
10398727	Klc1	N/A	19
10398812		N/A	13
10399005	Crip1	N/A	7
10399038	Zfp386	N/A	19
10399061	Esyt2	N/A	19
10399389	1700034J04Rik	N/A	13
10399636	Mrto4	N/A	13
10399642		N/A	13
10400143	Stxbp6	N/A	1
10400357	Baz1a	N/A	1
10400413	Garnl1	N/A	1
10400460	Mbip	N/A	1
10400483	Slc25a21	N/A	1
10400510	Clec14a	N/A	1
10400639	Rpl36al	N/A	13
10400668	Sdccag1	N/A	1
10400710	Gm71	N/A	1
10400762	Map4k5	N/A	1
10401028	Sgpp1	N/A	1
10401063	Zbtb25	N/A	1
10401309	Synj2bp	N/A	1
10401317	Gm4787	N/A	1
10401320	Adam4	N/A	1
10401322	Synj2bp	N/A	1
10401441		N/A	13
10401795	Alkbh1	N/A	1
10401824	Oog1	N/A	13
10401829	EG435337	N/A	1
10402195	Tc2n	N/A	1
10402225	Trip11	N/A	1
10402435	Serpina3c	N/A	13
10402997	LOC630837	N/A	13
10403015		N/A	7
10403018		N/A	13
10403021		N/A	13
10403034		N/A	13
10403043		N/A	13
10403048		N/A	13
10403054		N/A	13
10403060		N/A	13
10403079		N/A	7
10403246	Gm8598	N/A	13
10403258	Gdi2	N/A	1
10403348	Gm5444	N/A	13
10403604	Lyst	N/A	19
10403680	Arid4b	N/A	1
10403743	Inhba	N/A	1
10403754	Bat4	N/A	7

10403756	1600012F09Rik	N/A	1
10403765	Vps41	N/A	1
10403842	Elmo1	N/A	1
10403917	Olfr1367	N/A	13
10403982	V1rh17	N/A	13
10404020		N/A	13
10404022		N/A	13
10404024	Hist1h4h	N/A	13
10404026	Hist1h2af	N/A	13
10404113	Slc17a1	N/A	19
10404127	Hist1h2aa	N/A	7
10404538	Prpf4b	N/A	1
10404630	Riok1	N/A	1
10404763	Tmem170b	N/A	1
10404848	Jarid2	N/A	1
10404895	5033430I15Rik	N/A	13
10405094	Iars	N/A	1
10405291	Faf2	N/A	1
10405400	Nsd1	N/A	1
10405619	5133401N09Rik	N/A	13
10405725		N/A	13
10405727	2410127L17Rik	N/A	19
10405785	0610007P08Rik	N/A	1
10405847		N/A	19
10405870		N/A	13
10405876	Gm10324	N/A	13
10405916	A530054K11Rik	N/A	1
10405938	Zfp273	N/A	1
10406067	Clptm1l	N/A	13
10406111	Slc12a7	N/A	19
10406193	Ccdc127	N/A	1
10406205	Erap1	N/A	19
10406270	Glrx	N/A	19
10406399	Pou5f2	N/A	1
10406419	Lysmd3	N/A	1
10406466	Tmem161b	N/A	1
10406501		N/A	19
10406546		N/A	13
10406710	Tbca	N/A	13
10406905	Ccdc125	N/A	1
10406953	Trim23	N/A	1
10407042	Dimt1	N/A	1
10407122		N/A	19
10407281	Esm1	N/A	13
10407291	Arl15	N/A	1
10407392	BC016423	N/A	1
10407916	1600012F09Rik	N/A	1
10407924	Bat4	N/A	7
10408010	Zkscan3	N/A	1
10408049	Zfp192	N/A	1
10408062	Olfr1362	N/A	7
10408072	Hist1h2ao	N/A	13
10408085	Hist1h2an	N/A	13
10408094	RP23-480B19.10	N/A	13
10408280	Lrrc16a	N/A	19
10408329	Taf1d	N/A	19
10408331	Acot13	N/A	19
10408346	Gm9983	N/A	1
10408523	Foxq1	N/A	7
10408574		N/A	13
10408935	Gm10786	N/A	1
10408937	Atxn1	N/A	1
10409021	Tpmt	N/A	1
10409061		N/A	1
10409539		N/A	19
10409545		N/A	13
10409970	8430426H19Rik	N/A	1
10410039	Ptch1	N/A	7
10410078	Slc35d2	N/A	19
10410099	Cdc14b	N/A	7
10410115	1110018J18Rik	N/A	7
10410211		N/A	13
10410264	Mterfd1	N/A	1
10410295	Zfp595	N/A	1
10410317	Zfp459	N/A	13
10410351	AA987161	N/A	1
10410355	A530054K11Rik	N/A	1
10410362	6720487G11Rik	N/A	1
10410370	Zfp71-rs1	N/A	1
10410465	BC018507	N/A	13
10410650	Zfp825	N/A	1
10410656	Cast	N/A	13
10410889		N/A	13
10410892	Rasa1	N/A	1

10410927	LOC280487	N/A	7
10411126	Jmy	N/A	1
10411229	F2r	N/A	1
10411306	Polk	N/A	1
10411464	Fcho2	N/A	1
10411646	Gtf2h2	N/A	1
10411839	Sfrs12	N/A	1
10411915	Ppwd1	N/A	1
10411945	Fam159b	N/A	13
10412082	Gpbp1	N/A	1
10412258		N/A	1
10412481	2410127L17Rik	N/A	19
10412491		N/A	13
10412517	Gm10021	N/A	13
10412537	B930046C15Rik	N/A	13
10412543	Gm5458	N/A	13
10412549	D830030K20Rik	N/A	13
10412699	Gm10404	N/A	1
10412755	Gm8471	N/A	7
10412882	Thrb	N/A	7
10412981	Sec24c	N/A	1
10413086	Adk	N/A	1
10413216	4931406H21Rik	N/A	19
10413434	D14Abb1e	N/A	1
10413492	Lrtm1	N/A	19
10413670	Pbrm1	N/A	1
10413710		N/A	13
10413771	Capn7	N/A	1
10413897	Ercc6	N/A	1
10413993	Gm626	N/A	13
10414093	Glud1	N/A	1
10414218	4930503E14Rik	N/A	13
10414241		N/A	13
10414313		N/A	7
10414355	Mapk1ip1l	N/A	1
10414460	Nat12	N/A	1
10414706		N/A	13
10414726		N/A	13
10415119	Pabpn1	N/A	19
10415262	Dcaf11	N/A	13
10415377	2610027L16Rik	N/A	13
10415576	Zmym2	N/A	1
10415952		N/A	1
10416057	Clu	N/A	13
10416584	Gm4821	N/A	7
10416657	Elf1	N/A	1
10416696	Gm4638	N/A	7
10416698	Gm4638	N/A	7
10416725	LOC630896	N/A	13
10416989	Gpr180	N/A	13
10417183	Pcca	N/A	1
10417235	Gm3264	N/A	13
10417258	544988,00000000	N/A	13
10417264	544988,00000000	N/A	13
10417281		N/A	13
10417302	544988,00000000	N/A	13
10417315	Hn1l	N/A	13
10417319	D830030K20Rik	N/A	13
10417359	Gm3264	N/A	13
10417371	4930555G01Rik	N/A	13
10417415	Gm5458	N/A	13
10417446	4930555G01Rik	N/A	13
10417452	4930555G01Rik	N/A	13
10417492		N/A	13
10417504	1700001E04Rik	N/A	13
10417708	Oxsm	N/A	1
10417773		N/A	13
10418218	Slmap	N/A	1
10418796	Dph3	N/A	13
10418879	Mapk8	N/A	1
10418901	Gprin2	N/A	19
10418927	Bmpr1a	N/A	1
10419108		N/A	13
10419111	Gm10375	N/A	13
10419119	Gm8020	N/A	13
10419128	Gm10375	N/A	13
10419132	Gm10375	N/A	13
10419147	Gm10375	N/A	13
10419156	Ear10	N/A	13
10419162		N/A	13
10419343	D14Ertd436e	N/A	7
10419354		N/A	7
10419638	Chd8	N/A	1
10419742	Olfr49	N/A	13

10419779	Haus4	N/A	13
10419810	Psmb5	N/A	13
10420503	Setdb2	N/A	1
10420637	Kpna3	N/A	19
10421361	Bmp1	N/A	19
10421486	F630201L12Rik	N/A	13
10421488	Fndc3a	N/A	1
10421581	Lrch1	N/A	7
10421648	Slc25a30	N/A	7
10421661	Gtf2f2	N/A	1
10421758		N/A	1
10421768	Akap11	N/A	1
10421922		N/A	13
10421981	Dis3	N/A	1
10422022	Gm10843	N/A	19
10422075	Mycbp2	N/A	1
10422247	LOC280487	N/A	7
10422259	Tgds	N/A	13
10422760	Fyb	N/A	1
10422781	Rictor	N/A	1
10422854	Nup155	N/A	1
10422892	2410089E03Rik	N/A	1
10423053	Agxt2	N/A	1
10423577	Mtdh	N/A	1
10423663	Vps13b	N/A	1
10423731		N/A	1
10423825	Fzd6	N/A	7
10423917		N/A	13
10424126	Gm9920	N/A	1
10424211	Gm10370	N/A	13
10424252	Wdyhv1	N/A	7
10424377	Gm4638	N/A	7
10424485	Phf20l1	N/A	1
10424894	Heatr7a	N/A	13
10425263	Gm10864	N/A	19
10425335	Syngr1	N/A	13
10425651	4930407I10Rik	N/A	13
10426079		N/A	13
10426225	Ncaph2	N/A	13
10426292	Alg10b	N/A	1
10426298		N/A	13
10426477	Snrpc	N/A	7
10426507	1700124K17Rik	N/A	1
10426891	Mettl7a1	N/A	13
10426909	Letmd1	N/A	19
10427255	Tarbp2	N/A	13
10427389	LOC280487	N/A	7
10427428	AW549877	N/A	1
10427538	Nipbl	N/A	1
10427659		N/A	13
10428018	Ube2v2	N/A	1
10428192	Pabpc1	N/A	1
10428368		N/A	7
10428554	Eif3h	N/A	13
10428579	Ext1	N/A	1
10428744	9130401M01Rik	N/A	13
10428827	Tmem65	N/A	1
10429081	Snrpc	N/A	7
10429197		N/A	13
10429385		N/A	1
10429754	Nrbp2	N/A	19
10429968		N/A	1
10430145	Rbm9	N/A	19
10430489	Pla2g6	N/A	13
10430536	Ddx17	N/A	1
10430593	Josd1	N/A	13
10430679		N/A	1
10430711	Slc25a17	N/A	19
10430725	St13	N/A	13
10430846	Ndufa6	N/A	7
10430866	Cyp2d10	N/A	13
10430883	Cyp2d13	N/A	1
10430892	Pcdhb11	N/A	19
10430929	Tbrg3	N/A	1
10431284	Brd1	N/A	13
10431300	Alg12	N/A	1
10431912	Rpl36	N/A	13
10432152	Olfr284	N/A	13
10432281	Prkag1	N/A	13
10432573	Slc11a2	N/A	1
10432986	Aaas	N/A	7
10433214	Zfp174	N/A	1
10433228	Cluap1	N/A	19
10433352	Ubn1	N/A	1

10433389	Alg1	N/A	13
10433428		N/A	1
10433573		N/A	1
10433797	Prkdc	N/A	1
10433887	Pkp2	N/A	19
10433929	Ppm1f	N/A	13
10434302	Klhl24	N/A	7
10434348	Eif2b5	N/A	13
10434467	Psmd2	N/A	13
10434741		N/A	1
10434745		N/A	1
10434888	Opa1	N/A	1
10435237	Zfp148	N/A	1
10435453		N/A	13
10435470	Kpna1	N/A	1
10435565	Hcls1	N/A	13
10435641	Fstl1	N/A	7
10435693	Cox17	N/A	7
10435789	Zbtb20	N/A	1
10435802	2610015P09Rik	N/A	1
10436498	4933411O13Rik	N/A	13
10436590	2810055G20Rik	N/A	1
10436594		N/A	1
10436596	2810055G20Rik	N/A	19
10436598	2810055G20Rik	N/A	1
10436602		N/A	1
10436636	Ncam2	N/A	1
10436658	7120432I05Rik	N/A	19
10436773		N/A	13
10436775	1110057P08Rik	N/A	19
10436802		N/A	19
10436841	Il10rb	N/A	7
10437195	Igsf5	N/A	19
10437270	Cluap1	N/A	13
10437392	Magmas	N/A	7
10437561	Fam86	N/A	1
10437590	Carhsp1	N/A	13
10438109	Ube2l3	N/A	13
10438169		N/A	1
10438672	Tbccd1	N/A	1
10438708	Masp1	N/A	1
10438726	Abce1	N/A	1
10438738	Bcl6	N/A	19
10438749		N/A	7
10438753	Leprel1	N/A	1
10438815	1600021P15Rik	N/A	7
10439018	0610012G03Rik	N/A	13
10439218	Pdia5	N/A	13
10439520	Gm10809	N/A	13
10439547	Gramd1c	N/A	1
10439710	Phldb2	N/A	1
10439732	Phldb2	N/A	1
10439766	Pvrl3	N/A	7
10439788		N/A	13
10439989		N/A	7
10440050	Tbc1d23	N/A	1
10440149	Olfr186	N/A	13
10440164	Olfr194	N/A	13
10440288	Zfp654	N/A	1
10440292	Gm5488	N/A	1
10440307	Chmp2b	N/A	7
10440425		N/A	13
10440463	LOC436427	N/A	13
10440471	Mrpl39	N/A	13
10440491	App	N/A	13
10440534	Adamts5	N/A	1
10440564	Rnf160	N/A	1
10440593	Rwdd2b	N/A	7
10440881	1810007M14Rik	N/A	1
10440926	Dnajc28	N/A	1
10441344	Rbm16	N/A	1
10441394		N/A	13
10441933	Gm3435	N/A	19
10441952	2210404J11Rik	N/A	19
10441973	Tbp	N/A	1
10442087	4930546H06Rik	N/A	19
10442104	Fpr-rs4	N/A	13
10442206	Zfp51	N/A	1
10442231		N/A	1
10442236	3110052M02Rik	N/A	19
10442238		N/A	1
10442250	Zfp229	N/A	1
10442258	4930432O21Rik	N/A	1
10442262	Zfp758	N/A	1

10442270	1300003B13Rik	N/A	1
10442292	Vmn2r113	N/A	13
10442893	Wdr24	N/A	13
10442948	Mrpl28	N/A	13
10443199	C130040N14Rik	N/A	13
10443244	Anks1	N/A	7
10443817	Pknox1	N/A	7
10444595	Lsm2	N/A	7
10444717	D17H6S53E	N/A	13
10444936	Dhx16	N/A	13
10445141	Olfr111	N/A	13
10445183	Olfr136	N/A	13
10445251	Gpr110	N/A	7
10445434	Mrps18a	N/A	7
10445729	Tcfeb	N/A	13
10445826	Mocs1	N/A	19
10445898	Rab5a	N/A	7
10446341	4930505H01Rik	N/A	13
10446425		N/A	13
10446833	Birc6	N/A	1
10447004	Hdac1	N/A	13
10447056	Qpct	N/A	13
10447065	Fam82a1	N/A	1
10447079	4921513D11Rik	N/A	19
10447100	Dhx57	N/A	13
10447120	Tmem178	N/A	13
10447141	Eml4	N/A	1
10447254	Ppm1b	N/A	1
10447513		N/A	1
10447551	5730437N04Rik	N/A	7
10447589	Fbl	N/A	13
10447594	Dynlt1	N/A	13
10447629	Tagap1	N/A	1
10447702	Ppih	N/A	13
10447880	Mrpl18	N/A	1
10448089	Oaz1	N/A	13
10448127	Ncaph2	N/A	13
10448214	6330416L07Rik	N/A	1
10448230	LOC280487	N/A	7
10448416	Kctd5	N/A	13
10448755	Eme2	N/A	13
10449142	9530058B02Rik	N/A	13
10449266	Itfg3	N/A	13
10449363	Nudt3	N/A	13
10449527	Stk38	N/A	19
10449631	Btbd9	N/A	1
10450089	Ring1	N/A	7
10450444	Apom	N/A	13
10450646	Rbx1	N/A	13
10450648	Abcf1	N/A	13
10450856	Olfr92	N/A	19
10450858	Olfr93	N/A	13
10450876	Olfr102	N/A	19
10451193	E030047D23Rik	N/A	1
10451198	Vegfa	N/A	19
10451220	Mad2l1bp	N/A	13
10451238	Polr1c	N/A	13
10451451	Gnmt	N/A	7
10451736	Tbc1d5	N/A	1
10451856	Vmn2r118	N/A	13
10451860	Pot1b	N/A	1
10452110	2410015M20Rik	N/A	13
10452118	Lonp1	N/A	13
10452213	Gtf2f1	N/A	1
10452404	Nudt12	N/A	1
10452470	Vapa	N/A	7
10452701	Smchd1	N/A	1
10452907	Fez2	N/A	13
10453102	Sfrs7	N/A	1
10453141	Sos1	N/A	1
10453602		N/A	13
10453632	Rpl7a	N/A	7
10453676		N/A	1
10453678	Zeb1	N/A	1
10453692	Vps24	N/A	13
10453766	Thoc1	N/A	1
10454093		N/A	13
10454099	Morf4l1	N/A	13
10454103	Gm2889	N/A	13
10454298	Zfp397	N/A	1
10454332	Elp2	N/A	1
10454512		N/A	1
10454655	Apc	N/A	1
10454807	Snora74a	N/A	13

10454828	Pnet-ps	N/A	1
10454912	Ankhd1	N/A	1
10455312	Rbm27	N/A	1
10455372	C330007P06Rik	N/A	13
10455533	Eif1a	N/A	1
10455602	Dmxl1	N/A	1
10455954	Gm4951	N/A	1
10455957	Gm5970	N/A	19
10456120	Csnk1a1	N/A	1
10456291		N/A	1
10456346	Sec11c	N/A	1
10456490		N/A	1
10456501	Rnmt	N/A	1
10456619		N/A	1
10456622	Mbd1	N/A	13
10456798	Hdhd2	N/A	13
10456972	Gm10265	N/A	7
10456976	Gm10526	N/A	7
10456978	Gm10526	N/A	7
10456980	Gm10526	N/A	7
10456982	Gm10526	N/A	7
10456984	Gm10526	N/A	7
10456986	Gm10526	N/A	7
10457048	Gm10523	N/A	13
10457203		N/A	1
10457223	Cdh11	N/A	1
10457273	Kif5b	N/A	1
10457429	Rock1	N/A	1
10457665	4921533I20Rik	N/A	1
10457667	Gm4638	N/A	7
10457776		N/A	1
10457959	Sft2d3	N/A	1
10458044	Osgep	N/A	13
10458262	Slc23a1	N/A	1
10458285	5133400G04Rik	N/A	19
10458349	Sra1	N/A	13
10458607	Lars	N/A	1
10458641	Gpr151	N/A	13
10458757		N/A	13
10458784	Pggt1b	N/A	1
10458823		N/A	1
10458834	Atg12	N/A	1
10459183	Slc26a2	N/A	19
10459496	Ccbe1	N/A	13
10459590	Ptpn2	N/A	1
10459602	Ptpn2	N/A	1
10459643	4930503L19Rik	N/A	1
10459912	Adnp2	N/A	1
10459918	1110032A13Rik	N/A	13
10460070		N/A	1
10460102	1700034H14Rik	N/A	1
10460194		N/A	19
10460202	Suv420h1	N/A	1
10460344	Tmem134	N/A	13
10460392	Pold4	N/A	7
10460423	Spnb3	N/A	13
10460461	Rbm4b	N/A	7
10460492	Mrpl11	N/A	13
10460706	Syvn1	N/A	1
10461707	Olfr232	N/A	1
10461713	Olfr1440	N/A	13
10461826		N/A	7
10461921	2410127L17Rik	N/A	19
10462102	Ptar1	N/A	1
10462309	C030016D13Rik	N/A	1
10462333	Cdc37l1	N/A	1
10462454	Uhrf2	N/A	1
10462484	A1cf	N/A	1
10462507	Papss2	N/A	1
10462521	Pten	N/A	1
10462535	B430203M17Rik	N/A	1
10462702	Hectd2	N/A	1
10462724	Tnks2	N/A	1
10462752	Btaf1	N/A	1
10463027	Cyp2c29	N/A	1
10463138	Dppa2	N/A	13
10463200	Dhdpsl	N/A	1
10463254	D19Ertd386e	N/A	19
10463404	Fam178a	N/A	1
10463410	Fam178a	N/A	1
10463505	Dpcd	N/A	13
10463515	Hps6	N/A	13
10463643		N/A	19
10463687	D19Wsu162e	N/A	13

10463911	Add3	N/A	13
10463930	Mxi1	N/A	13
10464107	Ppnr	N/A	19
10464363	4930442E04Rik	N/A	13
10464448	Gm6020	N/A	1
10464529	Tcirg1	N/A	19
10464603	Aip	N/A	7
10464896	Peli3	N/A	13
10464974	Sf3b2	N/A	7
10465045	Drap1	N/A	7
10465379	Snx15	N/A	7
10465726	BC014805	N/A	19
10465742	C730048C13Rik	N/A	1
10465764	C730048C13Rik	N/A	1
10466272	Olfr1419	N/A	13
10466290	Olfr76	N/A	1
10466340	Olfr1448	N/A	13
10466357	Olfr1472	N/A	13
10466622	C730037M02Rik	N/A	19
10466624	Aldh1a7	N/A	19
10466682	Smc5	N/A	1
10466835	Snora19	N/A	1
10466837		N/A	13
10467139	Lipa	N/A	19
10467230	Ide	N/A	1
10467859	Cox15	N/A	1
10467871	Dnmbp	N/A	7
10467907	Erlin1	N/A	7
10468307	Gm6970	N/A	13
10468413	D19Ertd652e	N/A	13
10468487	LOC280487	N/A	7
10468519	Smndc1	N/A	7
10468653	A630007B06Rik	N/A	1
10468851	Snora19	N/A	1
10468881	Zfp826	N/A	1
10469083	Upf2	N/A	1
10469127	LOC280487	N/A	7
10469503		N/A	19
10469732	Yme1l1	N/A	1
10469856	Wdr85	N/A	19
10470182	Bmyc	N/A	7
10470322	Surf1	N/A	13
10470390	Gm13397	N/A	7
10470954	Lrrc8a	N/A	13
10470959	Phyhd1	N/A	13
10471018	Fam73b	N/A	19
10471503	Taf1d	N/A	1
10471555	Angptl2	N/A	19
10471749	Olfr345	N/A	13
10471772	Olfr360	N/A	19
10471784	Rabgap1	N/A	1
10471814	Gpr21	N/A	1
10471967	Mbd5	N/A	1
10472058	Rif1	N/A	1
10472277	March7	N/A	1
10472289	Tank	N/A	1
10472350	Gca	N/A	19
10472372		N/A	1
10472440	Tax1bp3	N/A	13
10472630	Ubr3	N/A	1
10472794		N/A	1
10473242		N/A	13
10473487	Olfr1031	N/A	19
10473491	Olfr1033	N/A	19
10473494	Olfr1034	N/A	19
10473496		N/A	1
10473578	Olfr1153	N/A	7
10473604		N/A	13
10473606	Olfr1193	N/A	13
10473643	Olfr1274	N/A	13
10473793	Psmc3	N/A	13
10473965	Arhgap1	N/A	1
10473976	Harbi1	N/A	1
10474207	Fbxo3	N/A	19
10474229	Cd59a	N/A	1
10474243	Cstf3	N/A	1
10474477	Gm13961	N/A	1
10474497		N/A	13
10474671	Spred1	N/A	7
10474870	Rpusd2	N/A	1
10475000	Rtf1	N/A	13
10475051	Mga	N/A	1
10475280	Lcmt2	N/A	1
10475378	Casc4	N/A	1

10475394	Ctdspl2	N/A	1
10475544	Sema6d	N/A	1
10475665	Usp8	N/A	1
10475686	Ap4e1	N/A	1
10475841		N/A	13
10476297		N/A	1
10476399	Gm4638	N/A	7
10476493	BC034902	N/A	13
10476728	Dtd1	N/A	13
10476819	Gm114	N/A	7
10476868	6430503K07Rik	N/A	19
10476880	A530006G24Rik	N/A	13
10476989	Gins1	N/A	13
10477122	Defb20	N/A	13
10477777	Ergic3	N/A	13
10477920	Myl9	N/A	7
10477929	1110008F13Rik	N/A	13
10478073	Snhg11	N/A	13
10478077	B230339M05Rik	N/A	1
10478746	LOC280487	N/A	7
10478778	Arfgef2	N/A	19
10478892	A530013C23Rik	N/A	7
10478926	1700101G07Rik	N/A	13
10479087	Stx16	N/A	1
10479625	BC050777	N/A	7
10479649	Prpf6	N/A	1
10479726	Pcmtd2	N/A	7
10479887	Sec61a2	N/A	1
10479988	Taf3	N/A	1
10480121	Fam188a	N/A	19
10480254	Cacnb2	N/A	13
10480379	Mrps5	N/A	13
10480804	Phpt1	N/A	13
10481111		N/A	1
10481135	Surf1	N/A	13
10481420	D2Wsu81e	N/A	7
10481531	BC005624	N/A	13
10481920	Psmd5	N/A	19
10482017	Rab14	N/A	1
10482172	Zbtb26	N/A	1
10482177	Strbp	N/A	1
10482323	Ppp6c	N/A	1
10482486	Mmadhc	N/A	1
10482687	Arl5a	N/A	1
10482731	Prpf40a	N/A	1
10483046	Dpp4	N/A	1
10483150	Fign	N/A	19
10483161	Gm4638	N/A	7
10483324	Scn9a	N/A	19
10483633	Sp3	N/A	1
10483646	Sp3	N/A	13
10483737	Atf2	N/A	1
10483770	Lnp	N/A	1
10483809	Nfe2l2	N/A	19
10484195	Ttn	N/A	13
10484197		N/A	19
10484201	2610301F02Rik	N/A	1
10484203	2610301F02Rik	N/A	19
10484205	2610301F02Rik	N/A	19
10484207	2610301F02Rik	N/A	19
10484355	LOC280487	N/A	7
10484461		N/A	19
10484547	Olfr1016	N/A	13
10484608	Olfr228	N/A	13
10484630	Olfr1090	N/A	13
10484679	Olfr1132	N/A	13
10484718	Olfr1165	N/A	19
10484739	Olfr1179	N/A	13
10484803	Olfr1228	N/A	13
10484816	Olfr1234	N/A	13
10484875	Olfr1506	N/A	13
10484880	Olfr142	N/A	13
10485027	F2	N/A	13
10485048	D2Ertd391e	N/A	1
10485309	E530001K10Rik	N/A	13
10485355	Gm4638	N/A	7
10485395	Trim44	N/A	1
10485483	Nat10	N/A	19
10485546	D430041D05Rik	N/A	13
10485594	Pin1l	N/A	13
10485633	Gm10796	N/A	19
10485667	Dnajc24	N/A	1
10485771	Olfr1286	N/A	13
10485807	Olfr1311	N/A	13

10485989	Aqr	N/A	1
10486041	Meis2	N/A	1
10486499	Tmem87a	N/A	1
10486712	Zscan29	N/A	1
10486898	Spg11	N/A	1
10487622	Snrpb	N/A	13
10487629	Idh3b	N/A	19
10488090	Tasp1	N/A	1
10488106	Gm14072	N/A	13
10488195	Rrbp1	N/A	7
10488291	Rbbp9	N/A	1
10488449		N/A	1
10488507	Abhd12	N/A	7
10488636	Defb26	N/A	7
10488860		N/A	1
10488959	6430550D23Rik	N/A	19
10489051	5730471H19Rik	N/A	1
10489053	4930518I15Rik	N/A	1
10489195	2610036D13Rik	N/A	7
10489266	Chd6	N/A	1
10489413	Tomm34	N/A	7
10489542	Wfdc3	N/A	7
10489721	LOC280487	N/A	7
10489831	Stau1	N/A	1
10490097	Cbln4	N/A	13
10490159	Pmepa1	N/A	7
10490221	Atp5e	N/A	13
10490302	2810021G02Rik	N/A	1
10490370	Psma7	N/A	13
10490510	Dido1	N/A	1
10490852		N/A	13
10490931	Ythdf3	N/A	1
10490989	Cp	N/A	1
10491014	Hltf	N/A	1
10491083	Nceh1	N/A	7
10491191	Rpl22l1	N/A	1
10491231	Mynn	N/A	1
10491279	Prkci	N/A	1
10491438	Ttc14	N/A	1
10491699	Fgf2	N/A	13
10491848	Larp2	N/A	1
10491858		N/A	1
10491958	Gm10729	N/A	7
10492220	2810407C02Rik	N/A	1
10492888		N/A	19
10492890	Lrba	N/A	1
10493094	Mrpl24	N/A	13
10493120	Gpatch4	N/A	19
10493177	Mef2d	N/A	1
10493474	Muc1	N/A	13
10493662	Nup210l	N/A	7
10493891	Ywhaz	N/A	13
10493921	Sf3a3	N/A	13
10494005		N/A	13
10494039	Lingo4	N/A	19
10494306	Mcl1	N/A	7
10494460	Pex11b	N/A	13
10494643	Hmgcs2	N/A	7
10494662	Ywhah	N/A	13
10494735	Gdap2	N/A	1
10495054	Rhoc	N/A	7
10495173	4933421E11Rik	N/A	1
10495183	Kcna3	N/A	13
10495530	Dph5	N/A	1
10495594		N/A	13
10495625	Dpyd	N/A	19
10495657	Alg14	N/A	19
10496130	A630047E20Rik	N/A	1
10496167	LOC280487	N/A	7
10496302	Manba	N/A	19
10496336	LOC280487	N/A	7
10496357		N/A	13
10496462	Adh6-ps1	N/A	1
10496892	Fubp1	N/A	1
10496919	Usp33	N/A	1
10496956	Pigk	N/A	1
10497001	Cryz	N/A	13
10497077		N/A	1
10497199	Tmem189	N/A	13
10497327	LOC280487	N/A	7
10497399	Pde7a	N/A	19
10497587	1600012F09Rik	N/A	1
10497648	Phc3	N/A	1
10497931	Cflar	N/A	1

10498000	Gm10730	N/A	13
10498307	Gm6394	N/A	7
10498415	Dhx36	N/A	1
10498485	Slc33a1	N/A	1
10498519	Ssr3	N/A	1
10498531	Ccnl1	N/A	1
10498566		N/A	1
10498591	Chmp1a	N/A	13
10498595	Snrpd2	N/A	13
10498710	Bche	N/A	19
10498837	Etfdh	N/A	1
10499132	Mab21l2	N/A	19
10499160	Cd1d1	N/A	19
10499309	Apoa1bp	N/A	13
10499420	Robld3	N/A	7
10499504	Mtx1	N/A	13
10499639	Cks1b	N/A	13
10499652	4632404H12Rik	N/A	1
10499876	Lelp1	N/A	13
10499902	Sprr4	N/A	13
10499969		N/A	13
10499979		N/A	13
10500034	Psmb4	N/A	13
10500103	Gabpb2	N/A	1
10500218	Tars2	N/A	13
10500360	Gm15441	N/A	13
10500388	Polr3c	N/A	7
10500469	Pde4dip	N/A	1
10500527	LOC280487	N/A	7
10500780	Nr1h5	N/A	1
10500796		N/A	1
10500798		N/A	1
10500813	Hipk1	N/A	1
10500845	A130049A11Rik	N/A	1
10500847	Magi3	N/A	1
10500876	Lrig2	N/A	1
10500976	Rap1a	N/A	1
10501420	Stxbp3a	N/A	7
10501447	Prpf38b	N/A	1
10501591	A930005H10Rik	N/A	19
10501690	Slc35a3	N/A	1
10501802	Tmem56	N/A	1
10501832	Abcd3	N/A	19
10501879	Usp53	N/A	1
10502440		N/A	7
10502770	Lphn2	N/A	1
10502774	Lphn2	N/A	1
10502780	Lphn2	N/A	1
10502787		N/A	7
10503085	Tgs1	N/A	1
10503232		N/A	1
10503251	2610301B20Rik	N/A	1
10503283	1110037F02Rik	N/A	1
10503410	Tmem64	N/A	19
10503431	Nbn	N/A	7
10503484	Fam82b	N/A	1
10503502	Ttpa	N/A	1
10503534	Ccnc	N/A	1
10503570	Sfrs18	N/A	1
10503615		N/A	19
10503656		N/A	13
10503695	Bach2	N/A	7
10504137	4933409K07Rik	N/A	19
10504148	4933409K07Rik	N/A	13
10504178	4933409K07Rik	N/A	13
10504201	4933409K07Rik	N/A	19
10504329	RP23-195K8.6	N/A	13
10504456	Ccin	N/A	13
10504499	Zcchc7	N/A	1
10504514	1700055D18Rik	N/A	1
10504613	E230008N13Rik	N/A	13
10504646		N/A	13
10504660		N/A	13
10504672	Tdrd7	N/A	7
10504753	LOC641050	N/A	13
10504761	LOC641050	N/A	13
10504902	Murc	N/A	13
10504926	Rnf20	N/A	1
10504955	Gm12466	N/A	13
10505200	Hsdl2	N/A	13
10505537	Gm428	N/A	13
10505543	Gm428	N/A	13
10505550	Gm428	N/A	13
10505556	Gm428	N/A	13

10505562	Gm428	N/A	13
10505623	D4Bwg0951e	N/A	1
10505630	Snapc3	N/A	1
10505749	Dennd4c	N/A	1
10505881		N/A	7
10505982	Fggy	N/A	19
10505994	Fggy	N/A	1
10505996	Fggy	N/A	19
10506031	Nfia	N/A	19
10506118	Usp1	N/A	1
10506125	Angptl3	N/A	19
10506134	Atg4c	N/A	1
10506146	Gm10305	N/A	1
10506431	Gm10304	N/A	13
10506498		N/A	13
10506500	Usp24	N/A	1
10506569	Usp24	N/A	13
10506571	Dhcr24	N/A	19
10506668	Yipf1	N/A	13
10507334	0610037D15Rik	N/A	7
10507594	Slc2a1	N/A	13
10507671	Guca2a	N/A	13
10507804	Trit1	N/A	1
10507894	Rragc	N/A	13
10508249	OTTMUSG00000009332	N/A	13
10508759	Rpa2	N/A	13
10508887	Nr0b2	N/A	7
10508972	Gm5589	N/A	13
10509023	Syf2	N/A	13
10509113	Fusip1	N/A	1
10509137	Hmgcl	N/A	13
10509204	Tcea3	N/A	7
10509228	Hnrnpr	N/A	1
10509246	Luzp1	N/A	19
10509253	4930549C01Rik	N/A	7
10509562	Mul1	N/A	13
10509596	Rnf186	N/A	7
10510125	Mcrs1	N/A	13
10510162	Ppp2r5a	N/A	1
10510176	Smarca5	N/A	1
10510194	Hmgb1	N/A	13
10510197	Ppp2r5a	N/A	19
10510212	Hmgb1	N/A	13
10510215	OTTMUSG00000010657	N/A	1
10510227	Hmgb1	N/A	13
10510957	Pank4	N/A	13
10511084	Nadk	N/A	7
10511139	Ssu72	N/A	13
10511366	Gm11780	N/A	19
10511501	2310030N02Rik	N/A	13
10511725	Cyb5r4	N/A	13
10511843	1810074P20Rik	N/A	1
10511952	Orc3l	N/A	1
10512125	Psenen	N/A	7
10512136	Bag1	N/A	13
10512350	4933409K07Rik	N/A	19
10512352	4933409K07Rik	N/A	19
10512463	LOC280487	N/A	7
10512867	Tex10	N/A	1
10512999	AI427809	N/A	13
10513002	Gm12471	N/A	13
10513082	D730040F13Rik	N/A	1
10513143	Ptpn3	N/A	13
10513162	Ptpn3	N/A	7
10513268	AI314180	N/A	1
10513622	Pole3	N/A	13
10514070	2310067E19Rik	N/A	19
10514158	Psip1	N/A	1
10514292	Ifna14	N/A	13
10514441	Mysm1	N/A	1
10514473		N/A	13
10514510	Cyp2j6	N/A	1
10514520	Cyp2j9	N/A	19
10514590	Dock7	N/A	19
10514645		N/A	13
10515086	9630013D21Rik	N/A	19
10515164	Cmpk1	N/A	1
10515253	Uqcrh	N/A	13
10515399	Plk3	N/A	7
10515528	Dph2	N/A	13
10515706	BC059842	N/A	13
10515771	Tie1	N/A	13
10515826	Olfr1337	N/A	13
10515828	Olfr1335	N/A	13

10515841	Gm12866	N/A	13
10515884	Ppih	N/A	13
10515930	AA415398	N/A	1
10516079	C130018J17Rik	N/A	13
10516103	Macf1	N/A	1
10516219	Gm10572	N/A	13
10516275	Rpl28	N/A	7
10516348	Eif2c3	N/A	1
10516576	Rbbp4	N/A	7
10516637	Fam167b	N/A	13
10516735	Tinagl1	N/A	13
10517053	Trnp1	N/A	13
10517687	Gm16287	N/A	1
10517706	Mrto4	N/A	13
10518216	Gm9944	N/A	13
10518228	Vps13d	N/A	19
10518329	Rpl28	N/A	7
10518341	Ppp2r5a	N/A	1
10518568	Gm13208	N/A	13
10518726	Slc25a33	N/A	7
10518847	Phf13	N/A	13
10518857	Zbtb48	N/A	7
10519052	2810405K02Rik	N/A	13
10519181		N/A	13
10519203	A230069A22Rik	N/A	1
10519211	Tas1r3	N/A	19
10519333	5830415L20Rik	N/A	1
10519354	Pex1	N/A	19
10519392	Krit1	N/A	1
10519420	Akap9	N/A	1
10519475		N/A	13
10519497	Steap4	N/A	7
10519640	Anapc5	N/A	13
10519655	Anapc5	N/A	13
10519657	Anapc5	N/A	13
10519667	Anapc5	N/A	13
10519669	Anapc5	N/A	13
10519671	Speer1-ps1	N/A	13
10519675	Anapc5	N/A	13
10519684	Anapc5	N/A	13
10519686	Anapc5	N/A	13
10519811	Speer7-ps1	N/A	13
10519857	Hgf	N/A	19
10520046		N/A	13
10520111	Nupl2	N/A	1
10520211	Agap3	N/A	13
10520271	2900005J15Rik	N/A	19
10520360		N/A	13
10520638		N/A	13
10520763	Gckr	N/A	1
10520815	Slc4a1ap	N/A	1
10520860		N/A	1
10520948	Plb1	N/A	19
10520965	Yes1	N/A	1
10520982	Depdc5	N/A	19
10521261	Htt	N/A	1
10521461	Grpel1	N/A	13
10521543	Stx18	N/A	1
10521602	Cpeb2	N/A	1
10521690	Ppih	N/A	13
10521811		N/A	13
10521822	Gm8012	N/A	13
10521848	Zcchc4	N/A	1
10521863	Anapc4	N/A	19
10522285	C330024D21Rik	N/A	13
10522303	Guf1	N/A	1
10522558		N/A	13
10522585		N/A	13
10522676	Srp72	N/A	1
10522742	LOC280487	N/A	7
10522908	4931407G18Rik	N/A	13
10522976	Rufy3	N/A	19
10523060	Gm9958	N/A	1
10523195	Thap6	N/A	1
10523316	Gm7682	N/A	13
10523337	Gm7682	N/A	13
10523346	Gm7682	N/A	13
10523468	Bmp2k	N/A	19
10523479	Slc25a5	N/A	19
10523746	BC005561	N/A	1
10523752	4932441K18Rik	N/A	1
10523785	Zfp326	N/A	19
10523905	Mtf2	N/A	1
10523955	Dr1	N/A	1

10524415		N/A	7
10524681	Triap1	N/A	13
10524844	Taok3	N/A	7
10525076	Sds	N/A	13
10525185		N/A	13
10525236	AU042671	N/A	7
10525336	Fam109a	N/A	7
10525397	Arpc3	N/A	7
10525553		N/A	1
10525835		N/A	13
10526133	Rabgef1	N/A	13
10526179	Rpl28	N/A	7
10526520	Plod3	N/A	13
10526726	Zkscan1	N/A	7
10527425	LOC280487	N/A	7
10527483		N/A	19
10527494	Cyp3a25	N/A	1
10527547	Gtf3a	N/A	13
10527571		N/A	7
10527832	Pds5b	N/A	1
10527878	LOC171266	N/A	13
10527880	C030048B08Rik	N/A	1
10528120	Dmtf1	N/A	1
10528161	Anapc5	N/A	13
10528165	LOC280487	N/A	7
10528198		N/A	13
10528202		N/A	13
10528205	LOC280487	N/A	7
10528257	Rsbn1l	N/A	1
10528268	Ptpn12	N/A	1
10528474		N/A	1
10528484	Srpk2	N/A	1
10528583	Cdk5	N/A	13
10528804	5031410I06Rik	N/A	13
10528810		N/A	13
10528815	5031410I06Rik	N/A	13
10528821	Gm10220	N/A	13
10528929	Hadha	N/A	13
10529091		N/A	19
10529118	Eif2b4	N/A	13
10529133	Zfp513	N/A	13
10529260	C330019G07Rik	N/A	1
10529273	Ctbp1	N/A	13
10529375	Mxd4	N/A	7
10529425	Nop14	N/A	1
10529741	Rab28	N/A	1
10529801	Fbxl5	N/A	1
10529923	Lcorl	N/A	1
10530278		N/A	1
10530625	Gm15653	N/A	13
10531034	Ugt2b34	N/A	19
10531197	Adamts3	N/A	13
10531199	D130050E23Rik	N/A	1
10531437	Scarb2	N/A	13
10531501	C87414	N/A	13
10531579		N/A	13
10531675	Sec31a	N/A	1
10531919	Hsd17b11	N/A	13
10531980	BC057170	N/A	7
10532157	Tmed5	N/A	1
10532164	Atp5k	N/A	13
10532313		N/A	1
10532588	Myo18b	N/A	13
10532716	1700069L16Rik	N/A	13
10532896	2610524H06Rik	N/A	13
10532926	Acads	N/A	13
10533071	Suds3	N/A	13
10533844	Rilpl2	N/A	13
10534504	Tmem120a	N/A	7
10534654	Znhit1	N/A	13
10534679	Trim56	N/A	7
10534889		N/A	7
10534909	Sp110	N/A	13
10535084	3110082I17Rik	N/A	7
10535189	Mad1l1	N/A	19
10535413		N/A	13
10535449	E130309D02Rik	N/A	13
10535477	Usp42	N/A	1
10535714	Cyp3a25	N/A	1
10535747	Gm10858	N/A	13
10535927		N/A	13
10535946	N4bp2l2	N/A	1
10536002	C87414	N/A	13
10536025	Gm7682	N/A	13

10536029	AA792892	N/A	13
10536033	Gm7682	N/A	13
10536041	AA792892	N/A	13
10536052	Gm7682	N/A	13
10536103	LOC624931	N/A	13
10536107	LOC625240	N/A	13
10536143	LOC624931	N/A	13
10536151	Gm7682	N/A	13
10536273	Casd1	N/A	1
10536294	Peg10	N/A	19
10536297	Ppp1r9a	N/A	1
10536369	C1galt1	N/A	1
10536376	Mios	N/A	1
10536440	Gm725	N/A	13
10536541	St7	N/A	19
10536620	Ing3	N/A	1
10536787		N/A	19
10537078	Mkln1	N/A	19
10537184	Cald1	N/A	1
10537494	Ssbp1	N/A	1
10537509	Mgam	N/A	1
10537828	Olfr434	N/A	13
10538586	V1rc27	N/A	13
10538755	Smarcad1	N/A	1
10538890	LOC641050	N/A	13
10539143	Retsat	N/A	13
10539177	Eif4a3	N/A	13
10539337	Mogs	N/A	13
10539710	Tia1	N/A	1
10539741	Aak1	N/A	1
10539882	Ruvbl1	N/A	1
10540122	Slc6a6	N/A	19
10540273	Ube2v2	N/A	1
10540531	LOC280487	N/A	7
10540705	Il17rc	N/A	13
10540790	Vhl	N/A	19
10540855	Hrh1	N/A	13
10541067	Eif4a3	N/A	13
10541075	Cxcl12	N/A	19
10541089	2900097C17Rik	N/A	7
10541129	LOC280487	N/A	7
10541246	Il17ra	N/A	7
10541318	Slc6a13	N/A	19
10541410	Mug1	N/A	1
10541426	Cpamd8	N/A	1
10541448	Cpamd8	N/A	1
10541873	Mrpl51	N/A	13
10542181	Clec9a	N/A	1
10542275	Etv6	N/A	1
10542310	Cdkn1b	N/A	1
10542376	Rpl36al	N/A	13
10542555	MGC7817	N/A	1
10542714	Lyrm5	N/A	1
10542824	Mrps35	N/A	13
10542993	Pon3	N/A	19
10543029	LOC280487	N/A	7
10543140	Gm6578	N/A	13
10543409	Tas2r118	N/A	13
10543512	Zfp800	N/A	1
10543678		N/A	7
10543686	Ube2h	N/A	7
10543697	Zc3hc1	N/A	7
10543709	Tmem209	N/A	1
10543904	Cnot4	N/A	1
10543944	Mtpn	N/A	1
10544002	Creb3l2	N/A	1
10544252	E330009J07Rik	N/A	13
10544497	Tarbp2	N/A	13
10544555	Zfp746	N/A	19
10544638	Tra2a	N/A	1
10544823		N/A	1
10544827		N/A	1
10544939		N/A	13
10544999	V1rc26	N/A	13
10545001	Ppm1k	N/A	7
10545130	Gadd45a	N/A	7
10545184		N/A	13
10545187		N/A	13
10545194		N/A	13
10545196		N/A	13
10545198		N/A	13
10545271	Thnsl2	N/A	7
10545346	Ptcd3	N/A	19
10545401	Vamp5	N/A	13

10545528		N/A	7
10545771	Cyp26b1	N/A	7
10545780	Exoc6b	N/A	1
10546092	Rab7	N/A	1
10546231	V1ra6	N/A	13
10546508		N/A	19
10546604		N/A	1
10546725	Pdzrn3	N/A	1
10546891	Camk1	N/A	13
10547009	Vgll4	N/A	13
10547015	1500001M20Rik	N/A	13
10547200		N/A	13
10547469	Hsn2	N/A	1
10547553	Mical3	N/A	1
10547689	Vmn2r26	N/A	13
10547758	Lpcat3	N/A	13
10547789	Grcc10	N/A	7
10548043	Kcna5	N/A	7
10548105	Ccnd2	N/A	13
10548661		N/A	13
10548761	Hebp1	N/A	13
10548785	Dynlt1	N/A	13
10549219	Gm10396	N/A	19
10549341	4933424B01Rik	N/A	1
10549402	Ergic2	N/A	1
10549530		N/A	1
10549532		N/A	1
10549536	Amn1	N/A	1
10549714	Rpl28	N/A	7
10549875	Olfr1350	N/A	13
10549899	Zfp418	N/A	1
10549964	Zscan4c	N/A	13
10549972	Zscan4c	N/A	13
10549990	LOC171266	N/A	13
10550200		N/A	13
10550316	Tmem160	N/A	13
10550531	Sympk	N/A	7
10550601		N/A	13
10550760	V1rd14	N/A	13
10550765	V1rd14	N/A	13
10550768	V1rd21	N/A	13
10550770	V1rd21	N/A	13
10550782	V1rd14	N/A	13
10550822	V1rd17	N/A	13
10550829	V1rd18	N/A	13
10550935	Xrcc1	N/A	7
10551102	Prr19	N/A	13
10551181	B9d2	N/A	13
10551215	Rnf170	N/A	1
10551393	Akt2	N/A	13
10551736	Ppp1r14a	N/A	13
10551815	Zfp260	N/A	1
10551836	Cox7a1	N/A	13
10551971	U2af1l4	N/A	7
10551981	U2af1l4	N/A	13
10551996		N/A	7
10552249	Gm6795	N/A	13
10552252	1600014C10Rik	N/A	7
10552260		N/A	13
10552276	Ube2h	N/A	7
10552363	EU599041	N/A	1
10552752	Akt1s1	N/A	7
10553741		N/A	1
10553813	Ube3a	N/A	1
10553967	Pcsk6	N/A	13
10554059	Gm10622	N/A	1
10554166	Akap13	N/A	1
10554323		N/A	13
10554665	Gm7957	N/A	7
10554893	Ccdc89	N/A	13
10554895	Crebzf	N/A	1
10555089	Rsf1	N/A	1
10555260	Olfr521	N/A	13
10555339	C2cd3	N/A	7
10555550	Clpb	N/A	13
10555681	Stim1	N/A	13
10555793	Olfr608	N/A	13
10555905	Olfr677	N/A	13
10555913	Olfr688	N/A	13
10555915	Olfr688	N/A	13
10556018		N/A	13
10556037	Olfr714	N/A	7
10556125	Gm6702	N/A	13
10556165	Olfr513	N/A	13

10556208	D930014E17Rik	N/A	13
10556442	Tead1	N/A	19
10556583	Nucb2	N/A	1
10556734	Acsm1	N/A	19
10557420	Tufm	N/A	13
10557628	Zfp689	N/A	1
10557780		N/A	1
10558001	Inpp5f	N/A	1
10558134	Plekha1	N/A	1
10558248	Bub3	N/A	13
10558515	Inpp5a	N/A	13
10558653	Olfr538	N/A	13
10558657	Olfr538	N/A	13
10558742	Nlrp6	N/A	19
10559357	Gm10574	N/A	19
10559454	Pira1	N/A	13
10559513		N/A	1
10559676	Ube2s	N/A	13
10559745		N/A	13
10559754	V1rd11	N/A	13
10559786	Olfr1348	N/A	13
10559978	Zscan4f	N/A	13
10560043	Zfp329	N/A	1
10560153	Obox3	N/A	13
10560315	Ppp5c	N/A	13
10560630	Tomm40	N/A	13
10560728	V1rd14	N/A	13
10560730	V1rd21	N/A	13
10560740	V1rd21	N/A	13
10560742	V1rd21	N/A	13
10560744	V1rd15	N/A	13
10560752	V1rd14	N/A	13
10560758	LOC100042966	N/A	13
10560771	Gm6239	N/A	13
10560780		N/A	13
10560783	V1rd21	N/A	13
10560785	V1rd21	N/A	13
10560787	V1rd21	N/A	13
10560789	V1rd21	N/A	13
10560797	V1rd14	N/A	13
10560801	Gm6239	N/A	13
10561170	Egln2	N/A	13
10561335	Prkcz	N/A	13
10561343	Josd1	N/A	7
10561345	Zfp780b	N/A	1
10561356	Psmc4	N/A	13
10561453	Zfp36	N/A	7
10561552	Eif3k	N/A	7
10561854	Tbcb	N/A	13
10562000	Psenen	N/A	7
10562059	Cox6b1	N/A	13
10562130	Ffar1	N/A	13
10562349	Pdcd2l	N/A	13
10562439	Gpatch1	N/A	1
10562576	Plekhf1	N/A	7
10562637	Ccnb1	N/A	13
10562685		N/A	13
10563085	Fcgrt	N/A	13
10563094	Rps11	N/A	13
10563101	Rpl13a	N/A	13
10563260	Snrnp70	N/A	1
10563712	Mrgpra2	N/A	13
10563802	Rbbp6	N/A	13
10563804	Rbbp6	N/A	13
10563806	Rbbp6	N/A	13
10563808	Rbbp6	N/A	13
10563810	Rbbp6	N/A	13
10563812	Rbbp6	N/A	13
10563899		N/A	13
10563959		N/A	13
10564089		N/A	13
10564109		N/A	13
10564117		N/A	13
10564539	Mctp2	N/A	1
10564573	Chd2	N/A	19
10565499	Gm5341	N/A	13
10565528	Gm2509	N/A	7
10565547	Pcf11	N/A	1
10565567	4632427E13Rik	N/A	1
10565587	Fxn	N/A	13
10565596		N/A	13
10565598	LOC280487	N/A	7
10565802	Rps3	N/A	13
10565815	Gm4980	N/A	13

10566201	Olfr589	N/A	13
10566227	Olfr620	N/A	13
10566249	Olfr68	N/A	13
10566322	Olfr648	N/A	13
10566409	Olfr670	N/A	13
10566599	Olfr699	N/A	13
10566607	Olfr705	N/A	1
10566707	Olfr519	N/A	13
10567072	Psma1	N/A	13
10567229	Smg1	N/A	1
10567394	Pdilt	N/A	19
10567446	Dnahc3	N/A	13
10567458	Dnahc3	N/A	19
10567518	Dnahc3	N/A	13
10567589	Usp31	N/A	13
10567702	Arhgap17	N/A	1
10568078	Taok2	N/A	19
10568107		N/A	1
10568217	Dctpp1	N/A	13
10568328	Vkorc1	N/A	7
10568392	Rgs10	N/A	13
10568534	Gm4638	N/A	7
10568638	Uros	N/A	19
10568861	Gm4974	N/A	13
10568958	Olfr530	N/A	13
10569011	Ifitm5	N/A	13
10569057	Rnh1	N/A	13
10569441	Nap1l4	N/A	13
10569958	Ccl25	N/A	19
10570139		N/A	13
10570280	F7	N/A	13
10570308	Proz	N/A	1
10570614	Defb6	N/A	13
10570639	6820431F20Rik	N/A	1
10570647	LOC100039037	N/A	13
10570656		N/A	13
10570676		N/A	13
10570690		N/A	13
10570706	Defa20	N/A	13
10570711	Defa20	N/A	13
10570729		N/A	13
10570975	Tm2d2	N/A	7
10571093	Rnf170	N/A	1
10571114	Erlin2	N/A	13
10571162	Eif4ebp1	N/A	7
10571241	Purg	N/A	1
10571599		N/A	1
10571824	Gm10674	N/A	13
10571826		N/A	13
10571857		N/A	13
10571920	B230317F23Rik	N/A	19
10571922	Nek1	N/A	1
10572077	Naf1	N/A	7
10572332	Sfrs14	N/A	19
10572449	Lsm4	N/A	13
10572724	Zfp709	N/A	1
10572730	Zfp617	N/A	1
10573194	Ndufb7	N/A	13
10573637	Phkb	N/A	1
10574135	Nlrc5	N/A	1
10574184	Rspry1	N/A	1
10574232	Coq9	N/A	13
10574350	Mmp15	N/A	7
10574375		N/A	1
10574429		N/A	13
10574432	Gm4638	N/A	7
10574434	LOC280487	N/A	7
10574456	Cklf	N/A	1
10574572	2210023G05Rik	N/A	1
10574641	D230025D16Rik	N/A	7
10574718	Tmem208	N/A	13
10575160	Nfat5	N/A	1
10575363	Zfp612	N/A	1
10575512	Cog4	N/A	13
10575619	Terf2ip	N/A	1
10575763	Gan	N/A	1
10576029	Cox4i1	N/A	13
10576056	Map1lc3b	N/A	7
10576160	Acsf3	N/A	13
10576403	Urb2	N/A	1
10576490		N/A	7
10576639	Nrp1	N/A	1
10576692	Insr	N/A	1
10577048	Ankrd10	N/A	1

10577065	Gm7606	N/A	13
10577070	Tubgcp3	N/A	1
10577312		N/A	13
10577395	6820431F20Rik	N/A	1
10577412	Cdh11	N/A	1
10577602		N/A	13
10577623	Gins4	N/A	13
10577922	Hook3	N/A	1
10578025		N/A	13
10578037	Eif3h	N/A	13
10578189	Chd6	N/A	1
10578241		N/A	1
10578251		N/A	19
10578324	Mtus1	N/A	7
10578421	Adam34	N/A	13
10578572	Stox2	N/A	19
10578619	Cdkn2aip	N/A	1
10578794		N/A	13
10578962	Gm10661	N/A	13
10578984	Tufm	N/A	13
10578989	Psd3	N/A	19
10579049	Gm10033	N/A	1
10579052	Gm10033	N/A	1
10579347	Ifi30	N/A	13
10579356	Pik3r2	N/A	13
10579437	Ccdc124	N/A	13
10579812	Ednra	N/A	1
10579915	Smarca5	N/A	1
10579993	Gm4787	N/A	1
10580010	Pkn1	N/A	13
10580080	Gm10643	N/A	13
10580183		N/A	7
10580300	Asna1	N/A	13
10580486	Brd7	N/A	1
10580537	Aktip	N/A	13
10580622	Gm5158	N/A	13
10580663	AU018778	N/A	19
10580805	Gm10286	N/A	13
10580870	Zfp319	N/A	13
10580905	Cnot1	N/A	1
10581009	LOC280487	N/A	7
10581499	Chtf8	N/A	13
10581571	4922502B01Rik	N/A	13
10581926	Adat1	N/A	1
10581959		N/A	13
10582123	Hsdl1	N/A	13
10582427	Cbfa2t3	N/A	13
10582839	Gm9909	N/A	19
10582918		N/A	13
10582925	Alkbh8	N/A	1
10582941	Cwf19l2	N/A	1
10582983	LOC280487	N/A	7
10583021	Pdgfd	N/A	19
10583203	Phxr4	N/A	1
10583450	Zfp846	N/A	1
10583519	Icam1	N/A	13
10583905	Sept7	N/A	1
10583952	Ncapd3	N/A	1
10584095	Nfrkb	N/A	19
10584276	Sec61g	N/A	1
10584403	Olfr251	N/A	13
10584433	Olfr914	N/A	13
10584504	Olfr919	N/A	13
10584510	Olfr970	N/A	13
10584514	Olfr972	N/A	7
10585325		N/A	13
10585331	Exph5	N/A	19
10585444	Ireb2	N/A	7
10585586	Ube2s	N/A	13
10585980	Myo9a	N/A	19
10585982	Myo9a	N/A	1
10585988	Myo9a	N/A	1
10585990	Myo9a	N/A	1
10586110	Cln6	N/A	13
10586170		N/A	1
10586172		N/A	1
10586174		N/A	1
10586227	Dennd4a	N/A	1
10586244	Dennd4a	N/A	1
10586246	Dennd4a	N/A	1
10586248	Dennd4a	N/A	7
10586250	Dennd4a	N/A	1
10586252	Dennd4a	N/A	1
10586254	Dennd4a	N/A	1

10586505	Herc1	N/A	1
10586616	Vps13c	N/A	1
10586781	Myo1e	N/A	1
10586863	LOC280487	N/A	7
10586880	Suhw4	N/A	1
10586967	Gm7265	N/A	1
10587082	Onecut1	N/A	7
10587104	Arpp19	N/A	1
10587419	Senp6	N/A	1
10587446	Myo6	N/A	1
10587776		N/A	1
10587892	Atr	N/A	1
10587942	Xrn1	N/A	1
10588049	Copb2	N/A	1
10588482	Wdr51a	N/A	7
10588577	Cish	N/A	13
10588722	Mon1a	N/A	13
10588893	Rhoa	N/A	7
10589061	Dalrd3	N/A	13
10589464	Gm10615	N/A	1
10589565	Setd2	N/A	1
10589756	Epm2aip1	N/A	1
10589974	LOC280487	N/A	7
10590253	Rpsa	N/A	7
10590381	Vipr1	N/A	13
10590389	Nktr	N/A	1
10590479	Zfp167	N/A	1
10590654	Aasdhppt	N/A	1
10590799	C330006D17Rik	N/A	1
10590844	9030420J04Rik	N/A	1
10591116	Fat3	N/A	13
10591203	Olfr860	N/A	13
10591228	Zfp26	N/A	1
10591241	Zfp426	N/A	1
10591253	5730601F06Rik	N/A	1
10591472	Cdc37	N/A	13
10591537	Tmed1	N/A	13
10591612	Dock6	N/A	19
10591614	Dock6	N/A	19
10591726	Ecsit	N/A	13
10591735	Elof1	N/A	7
10591881		N/A	13
10592099	Dcps	N/A	7
10592397	Olfr144	N/A	13
10592585	Sc5d	N/A	19
10592850	Trappc4	N/A	13
10592926	Tmem25	N/A	1
10593032	Amica1	N/A	13
10593198	Fam55b	N/A	1
10593219	Nnmt	N/A	13
10593325	Pts	N/A	19
10593367	Dlat	N/A	19
10593430	Ppp2r1b	N/A	1
10593497	Zc3h12c	N/A	1
10593508	Ddx10	N/A	1
10593668	Dmxl2	N/A	19
10593671	Dmxl2	N/A	19
10593713	Cib2	N/A	13
10593799	Scaper	N/A	1
10593882	Gm10658	N/A	19
10593884	Ptpn9	N/A	1
10593903	Commd4	N/A	13
10594289	Glce	N/A	7
10594322	Pias1	N/A	1
10594517	5430433E21Rik	N/A	13
10595327	Phip	N/A	1
10595723	2610101N10Rik	N/A	1
10595990	Armc8	N/A	19
10596072	Ppp2r3a	N/A	19
10596115	Ephb1	N/A	13
10596261	Dnajc13	N/A	1
10596263	Dnajc13	N/A	19
10596265	Dnajc13	N/A	1
10596267	Dnajc13	N/A	1
10596273	Dnajc13	N/A	1
10596275	Dnajc13	N/A	1
10596277	Dnajc13	N/A	1
10596281	Dnajc13	N/A	1
10596857	Apeh	N/A	13
10596893	Dag1	N/A	19
10596900	Tcta	N/A	19
10597095	3000002C10Rik	N/A	1
10597273	Rtp3	N/A	19
10597354	Pdcd6ip	N/A	1

10597898		N/A	13
10597913	1110059G10Rik	N/A	7
10598064		N/A	7
10598071		N/A	7
10598073		N/A	7
10598212		N/A	13
10598218	Gmcl1l	N/A	13
10598225	Gmcl1l	N/A	13
10598229		N/A	13
10598422	Gripap1	N/A	1
10598501	EG627782	N/A	13
10598565		N/A	13
10598612	Otc	N/A	1
10598646	Gm14484	N/A	13
10598648	OTTMUSG00000016789	N/A	7
10598650	OTTMUSG00000016789	N/A	13
10598652	Gm14484	N/A	13
10598654	OTTMUSG00000016789	N/A	13
10598678	Usp9x	N/A	1
10599058		N/A	13
10599060		N/A	13
10599062	RP23-110D11.1	N/A	13
10599064	RP23-110D11.1	N/A	13
10599073	RP23-110D11.1	N/A	13
10599075	RP23-110D11.1	N/A	13
10599092		N/A	13
10599094	RP23-110D11.1	N/A	13
10599096	RP23-110D11.1	N/A	13
10599105	RP23-110D11.1	N/A	13
10599107	RP23-110D11.1	N/A	13
10599213		N/A	1
10599215	Slc25a5	N/A	19
10599232	Nkap	N/A	1
10599321	Zbtb33	N/A	1
10599377	Stag2	N/A	1
10599416	Gm10483	N/A	7
10599498	Utp14a	N/A	1
10599696	Ddx26b	N/A	1
10600017	Hmgb3	N/A	13
10600357	Taz	N/A	1
10600372	B230340J04Rik	N/A	19
10600453	Ikbkg	N/A	19
10600512	Brcc3	N/A	19
10600566		N/A	1
10600718		N/A	1
10600823	LOC675747	N/A	13
10600911	Yipf6	N/A	1
10600971	Otud6a	N/A	13
10601084		N/A	13
10601235	Ogt	N/A	19
10601328	Uprt	N/A	1
10601449	Sh3bgrl	N/A	1
10601581	9230105E10Rik	N/A	7
10601595	3110007F17Rik	N/A	7
10601598	3110007F17Rik	N/A	7
10602020	Tbc1d8b	N/A	1
10602068	Mid2	N/A	1
10602192		N/A	19
10602372	Alas2	N/A	7
10603023		N/A	13
10603228		N/A	13
10603545		N/A	13
10603549	LOC280487	N/A	7
10603625		N/A	13
10603651	1810030O07Rik	N/A	1
10603659	Med14	N/A	19
10603744		N/A	1
10603803	LOC280487	N/A	7
10603809	Gm14636	N/A	1
10603878	Uxt	N/A	1
10603953		N/A	13
10603962	RP23-110D11.1	N/A	13
10603964	RP23-110D11.1	N/A	13
10603973	RP23-110D11.1	N/A	13
10603975	RP23-110D11.1	N/A	13
10603984	RP23-110D11.1	N/A	13
10603986	RP23-110D11.1	N/A	13
10603995	RP23-110D11.1	N/A	13
10603997	RP23-110D11.1	N/A	13
10604006	RP23-110D11.1	N/A	13
10604008	RP23-110D11.1	N/A	13
10604017	RP23-110D11.1	N/A	13
10604021	RP23-110D11.1	N/A	13
10604023	RP23-110D11.1	N/A	13

10604038	Sfrs17b	N/A	1
10604078	Upf3b	N/A	1
10604187	Lamp2	N/A	1
10604199	Cul4b	N/A	1
10604234	Gm7189	N/A	13
10604245		N/A	1
10604248	Thoc2	N/A	1
10604337		N/A	1
10604505	6720401G13Rik	N/A	1
10604610		N/A	13
10604735	Rbmx	N/A	1
10604832		N/A	19
10605081	Bcap31	N/A	13
10605319	Ubl4	N/A	13
10605349		N/A	1
10605392	F8	N/A	1
10605421	Mtcp1	N/A	1
10605437	Pls3	N/A	1
10605571	Gyk	N/A	1
10605740	Eif2s3x	N/A	1
10605782		N/A	7
10605952	Gdpd2	N/A	13
10606088	Hdac8	N/A	1
10606160	Rfwd2	N/A	1
10606263	Atrx	N/A	1
10606376	2610002M06Rik	N/A	1
10606391	Gm10452	N/A	1
10606393	Brwd3	N/A	1
10606513	Chm	N/A	1
10606969	Rbm41	N/A	7
10607113	Rgs3	N/A	13
10607116	Ammecr1	N/A	1
10607246	Tmem29	N/A	19
10607346	Tspyl2	N/A	1
10607366	Shroom2	N/A	7
10607391		N/A	13
10607398		N/A	19
10607429	LOC280487	N/A	7
10607658	Reps2	N/A	1
10607679	4932441K18Rik	N/A	1
10607952	Vamp7	N/A	1
10607972	Kdm5d	N/A	1
10608001	Eif2s3y	N/A	1
10608085	Gm2889	N/A	13
10608107		N/A	1
10608209		N/A	13
10608212		N/A	13
10608222		N/A	13
10608237	Sly	N/A	13
10608247	LOC100042196	N/A	13
10608249		N/A	13
10608251	LOC380994	N/A	13
10608263	Sly	N/A	13
10608273		N/A	13
10608277	LOC100041207	N/A	13
10608280		N/A	13
10608282	LOC100041256	N/A	13
10608290		N/A	1
10608293		N/A	13
10608295		N/A	13
10608302		N/A	13
10608308		N/A	13
10608313	Ssty1	N/A	13
10608317	Ssty1	N/A	13
10608321		N/A	13
10608325		N/A	13
10608327	LOC100041256	N/A	13
10608339	Ssty2	N/A	13
10608342	LOC100041233	N/A	13
10608346	LOC665346	N/A	13
10608348		N/A	13
10608350		N/A	13
10608361		N/A	13
10608365	Ssty2	N/A	13
10608368	LOC100041256	N/A	13
10608371		N/A	13
10608373	Ssty2	N/A	13
10608377	LOC382133	N/A	13
10608382		N/A	13
10608385	Sly	N/A	13
10608394		N/A	13
10608397	Sly	N/A	13
10608410	Sly	N/A	13
10608420		N/A	13

10608422	Rmi1	N/A	1
10608424		N/A	13
10608426		N/A	13
10608429	LOC380994	N/A	13
10608438	Srsy	N/A	13
10608448	LOC621831	N/A	13
10608452		N/A	13
10608454		N/A	13
10608457		N/A	13
10608460	LOC665698	N/A	13
10608464	Sly	N/A	13
10608474	Srsy	N/A	13
10608477		N/A	13
10608480	LOC100039147	N/A	13
10608482		N/A	13
10608484		N/A	13
10608488		N/A	13
10608499		N/A	13
10608501		N/A	13
10608506		N/A	13
10608513		N/A	13
10608517		N/A	13
10608521		N/A	13
10608531		N/A	13
10608549		N/A	13
10608567		N/A	13
10608573		N/A	13
10608576		N/A	13
10608583	Srsy	N/A	13
10608586	Sly	N/A	13
10608596		N/A	13
10608608	Ssty2	N/A	13
10608613		N/A	13
10608615	LOC380994	N/A	13
10608625	LOC100040188	N/A	13
10608628	LOC100041704	N/A	13
10608630	Ssty2	N/A	13
