# Supplementary material for: Circadian Desynchrony Promotes Metabolic Disruption in a Mouse Model of Shiftwork
Source: PLoS One. 2012 May 21;7(5):e37150. doi: 10.1371/journal.pone.0037150 (PMC3357388; doi:10.1371/journal.pone.0037150)
Supplement: Table S2 — Category 1 enriched GO processes (DAVID) of Class III TSR-regulated genes ( Figure 3E ) in the liver. (PDF) [file pone.0037150.s005.pdf]

Class III Liver Ontology GOTERM_BP_1

| Term | No. of Genes | P-value |
|---|---|---|
| GO:0008152 metabolic process | 1033 | 2.90E-06 |
| GO:0009987 cellular process | 1287 | 1.33E-05 |
| GO:0044085 cellular component biogenesis | 103 | 4.00E-03 |
| GO:0051234 establishment of localization | 334 | 3.10E-03 |
| GO:0051179 localization | 365 | 1.20E-02 |
| GO:0016043 cellular component organization | 265 | 1.50E-02 |
